# Analysis of variability in high throughput screening data: applications to melanoma cell lines and drug responses

**DOI:** 10.18632/oncotarget.15347

**Published:** 2017-02-15

**Authors:** Kuan-Fu Ding, Darren Finlay, Hongwei Yin, William P.D. Hendricks, Chris Sereduk, Jeffrey Kiefer, Aleksandar Sekulic, Patricia M. LoRusso, Kristiina Vuori, Jeffrey M. Trent, Nicholas J. Schork

**Affiliations:** ^1^ J. Craig Venter Institute, La Jolla, San Diego, CA, USA; ^2^ University of California, San Diego, CA, USA; ^3^ The Translational Genomics Research Institute, Phoenix, AZ, USA; ^4^ Sanford Burnham Prebys Medical Discovery Institute, La Jolla, San Diego, CA, USA; ^5^ Yale University, New Haven, CT, USA

**Keywords:** melanoma, high-throughput screening, drug screens, computational modeling, variability

## Abstract

High-throughput screening (HTS) strategies and protocols have undergone significant development in the last decade. It is now possible to screen hundreds of thousands of compounds, each exploring multiple biological phenotypes and parameters, against various cell lines or model systems in a single setting. However, given the vast amount of data such studies generate, the fact that they use multiple reagents, and are often technician-intensive, questions have been raised about the variability, reliability and reproducibility of HTS results. Assessments of the impact of the multiple factors in HTS studies could arguably lead to more compelling insights into the robustness of the results of a particular screen, as well as the overall quality of the study. We leveraged classical, yet highly flexible, analysis of variance (ANOVA)-based linear models to explore how different factors contribute to the variation observed in a screening study of four different melanoma cell lines and 120 drugs over nine dosages studied in two independent academic laboratories. We find that factors such as plate effects, appropriate dosing ranges, and to a lesser extent, the laboratory performing the screen, are significant predictors of variation in drug responses across the cell lines. Further, we show that when sources of variation are quantified and controlled for, they contextualize claims of inconsistencies and reveal the overall quality of the HTS studies performed at each participating laboratory. In the context of the broader screening study, we show that our analysis can also elucidate the robust effects of drugs, even those within specific cell lines.

## INTRODUCTION

High-throughput screening (HTS) strategies allow researchers to assess the effects of thousands of compounds on drug responses in one large experimental setting. Over the past decade, applications of HTS for drug discovery and drug effect characterization studies have steadily increased, ranging from studies focusing on the assessment of multiple phenotypic endpoints in high-content screening [1], the evaluation of drugs on traits such as lifespan through the sophisticated use of model species such as *Caenorhabditis elegans* [2] as well as drug response pattern identification using massive amounts of genomic information made available for crowd-sourcing efforts and community driven challenges [3]. HTS studies have also been pursued to advance personalized medicine, especially in oncology settings, since tumor-derived cell lines can be used in the screening studies to identify compounds that are active against them or some subset of them. For example, large HTS databases – such as the NCI-60 [4], the Cancer Cell Line Encyclopedia [5] (CCLE) and the Genomics of Drug Sensitivity in Cancer [6] (GDSC) – have been made available to researchers for the express purpose of uncovering drugs that exhibit unique effects against tumor-generated cell lines with specific genomic profiles. In addition, very recent work involving the Connectivity Map [7] has exploited genetic network and pathway reconstruction methods to identify sets of genes that mediate specific drug responses in subsets of cancers. In this light, the Cancer Cell Map Initiative [8] (CCMI) and related initiatives have not only drastically reduced drug discovery costs, but have also guided efforts to identify genomically-informed, patient-specific cancer treatment strategies. Unfortunately, as timely and as sophisticated as these efforts have been, very recent studies comparing the quality of different HTS studies meant to advance insights into personalized cancer care have raised questions and concerns about their reliability and reproducibility as well as the interpretation of the data they generated [9, 10].

Assessing the reliability of HTS studies is not trivial given the number of compounds typically considered, the number of reagents used, the way in which constructs such as plating schemes and distributed robotic handlers are set up, the manner in which dose response curves are constructed, and the fact that different labs likely follow slightly, if not overtly, different protocols; i.e., the sources of HTS cells, tissues or organisms, cell culture and assay conditions, reagents, consumables and instrumentation are not standardized within the community. This is particularly true for, e.g., tumor-derived cell line-based screening studies where the nature of the source cell lines, their procurement and sustenance as well as the responses measured on them may vary widely between different laboratories. In addition, although diverse in execution, many cancer-oriented HTS studies focus on cell counts upon stimulation with a drug that reflect that drug's ability to kill cells derived from a specific cell lines across differing drug concentrations. These concentrations often range from inducing no response (i.e., no cells are killed) to a very strong response (e.g., all the cells are killed). The dose ranges necessary to achieve variation in the number of cells killed and establish a dose response curve are very hard to anticipate, often leading to different labs using different concentrations and numbers of concentrations.

After having established the drug concentrations or doses to be used and applying them to cells derived from a single cancer cell line, sigmoidal curves are often fit to the cell counts associated with each drug dose to generate drug-specific dose response curves (DRCs). This is repeated for each cell line. The half minimal inhibitory concentration (IC_50_) is then extracted from these curves [4, 5]. These IC_50_ values, which are often coupled with related response measures such as the area under each dose response curve (AUC), are then used to determine the sensitivity or resistance of each cell line to the different drugs. Recently, the IC_50_ and AUC results from two large cancer cell line HTS studies, the Cancer Cell Line Encyclopedia [11] (CCLE) and the Genomics of Drug Sensitivity in Cancer [12] (GDSC) studies, were used to assess the variability of HTS assays pursued in this manner [7, 8]. The results of an assessment of the comparability and reproducibility of CCLE and GDSC data sets by two different research teams yielded opposing interpretations, which underscores the complexity of HTS studies and their interpreration [7, 8, 13]. A third research team recently reevaluated the CCLE and GDSC data and came to yet a different a conclusion [14].

In order to assess the reliability of HTS data, we conducted a study of intra- and inter-site experimental variability across melanoma cell lines treated with 120 different drugs that are either in use in clinical trials or have been FDA-approved for use in treating cancers. Our study was motivated by not only the controversies surrounding the reliability of the CCLE and GDSC data sets, but also by our engagement in a large clinical trial exploring the utility of personalized treatment for late-stage BRAF wild-type melanoma [15]. We first measured variability across replicated dose and drug applications to 29 melanoma cell lines pursued within a single institution, the Sanford Burnham Prebys (SBP) Medical Discovery Institute in La Jolla, California. The SBP studies were pursued using two independent HTS formats and screens: a nine-concentration and a three-concentration dose-response screens. Using the same 120 drugs and four of the 29 cell lines, we performed an independent nine-concentration dose response screen at the Translational Genomics Research Institute (TGen) in Phoenix, Arizona. To enable analysis of inter-site experimental variability, two copies of the master drug plates were generated at SBP. One was then ultimately used onsite at SBP and the other was provided to TGen for their respective screens. Furthermore, the two sites used the same final dosing concentrations and the same cell lines. All other aspects of the screen were independent, resulting in variation in the environments in the which the screens were pursued, personnel, compound freeze/thaw cycles, cell passages, culture conditions, plating density, actual plates and other consumables, and instrumentation.

To analyze the data produced from the two nine-concentration HTS studies, and to assess the variability of the results, we used flexible linear models and analysis of variance (ANOVA) techniques. These traditional techniques allowed us to examine how variation in drug responses (i.e., variation in the fraction of cells killed for a particular cell line, drug and dose) is impacted by different factors, such as the laboratory, the drug used, the plate on which specific assays were conducted. We also considered interaction terms in the models (e.g., dose x drug interactions). We chose not to generate dose-response curves and extract IC50 values for use in our analyses, since our interests were in quantifying as many sources of variation as possible and not condensing or obscuring any of them in dose-response relationships reflected in single derived value. Thus, we modeled the dosage effects of each drug as a separate independent or explanatory variable for drug response variation. It is well-known that models that are “saturated” in that they exhaustively model the effects of independent variables and their interactions are inherently linear. This fact is exploited in many contexts, most notably econometrics, to help draw causal inferences between independent and dependent variables [16]. Although we did not consider all possible interaction terms in our models, we did consider most of them. Ultimately, our linear modeling and ANOVA analyses allowed us to make comprehensive claims about that effects of particular drugs and dosages on specific cell lines while accounting for factors built into the design of the HTS, such a plate effects, that could induce variation in drug responses. Thus, we believe our analyses can help identify signals of truly statistically-significant drug effects over-and-above the “noise” created by various factors, including individual laboratories and/or the individual plates upon which cells were placed for drug effect characterization. We firmly believe that more sophisticated analytical methods, careful analyses, and interpretations of drug effect claims in HTS experiments are necessary and will likely lead to the identification and characterization of correctable sources of variation that may obscure HTS results and shed light on claims about their lack of reproducibility.

## RESULTS

### Variation of cell viability data across dose and drug replicates

**Figure 1:**
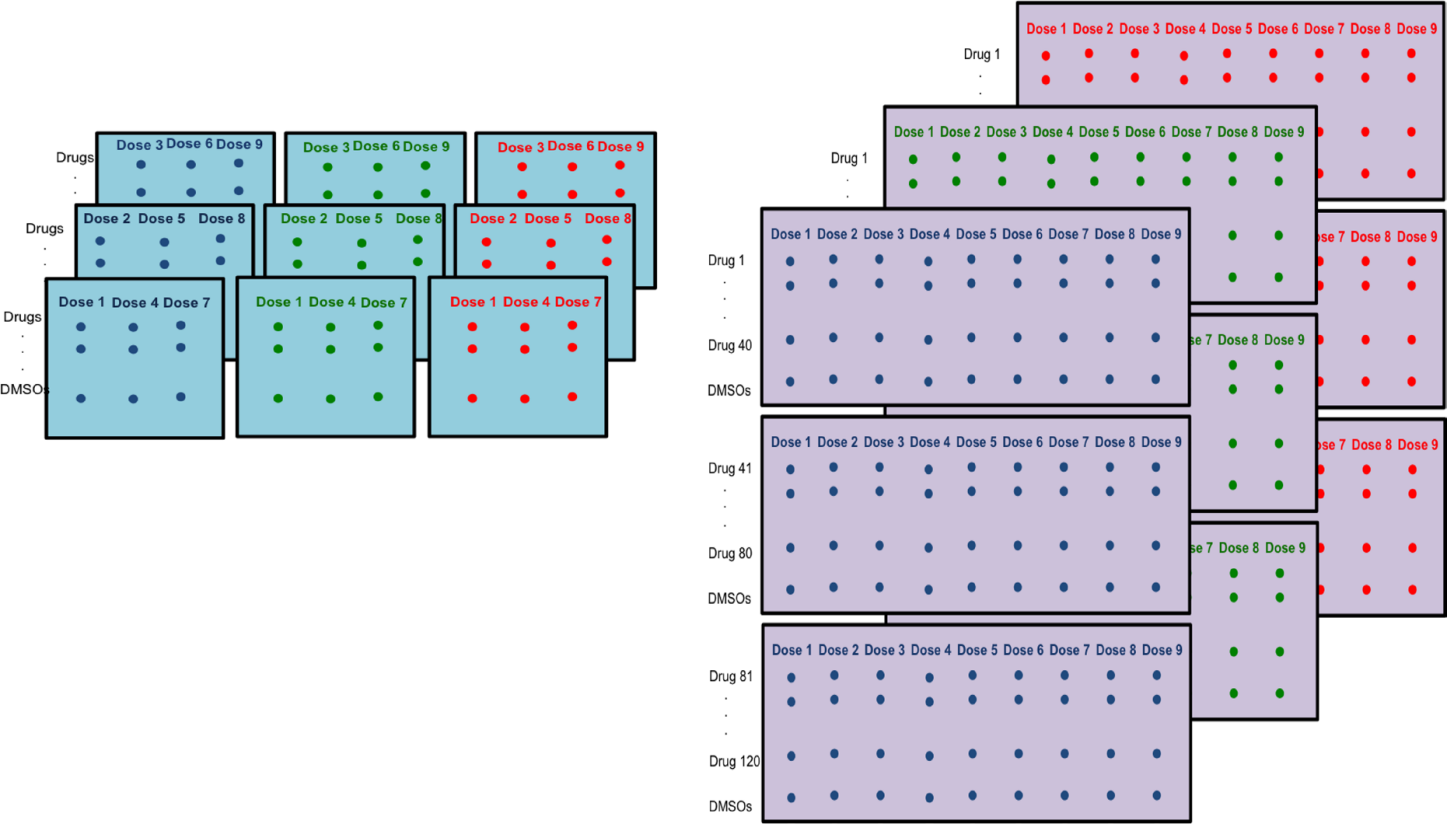
Differences in HTS plating schemes Nine 384-well plates are used for each cell line. Left: TGen/SBP plating scheme. Each plate consists of triplicates of three doses across 120 drugs and 8 DMSOs. Right: Alternate plating scheme with each plate including all 9 doses.

Table 1 provides a brief summary of the data sets we considered in our analyses. As an initial assessment of the consistency of drug-by-dose effects across the 29 cell lines in common between the SBP nine-concentration and three-concentration HTS studies (Figure 1), which comprise all HTS data obtained at matching doses within the SBP HTS data, we considered the use of simple correlation analyses. We found that the cell viability data are not normally distributed (Shapiro-Wilk's test *p-value* < 2.2e-16 for drug responses for each drug) and that the non-parametric Spearman correlation coefficient, rather than the standard Pearson correlation coefficient, would be more appropriate for use in assessing the consistency between the two data sets. We computed pairwise Spearman correlations for the replicates at 0.1, 1.0, and 10.0 μM concentrations across each drug and cell line (Figure 2A, Supplementary Figure 1). Additionally, we calculated the correlation coefficients using all available concentrations. As expected, the pairwise correlations at the three concentration points suggested that a subset of the cell lines showed greater evidence for reproducibility. These cell lines were identified as those most likely to be sensitive to the drugs. This makes sense since the cell lines exhibiting no response (i.e., does response curve) contributed only noise to the correlations. In addition, within each dose, the distribution of the correlation coefficients for each drug was skewed left (Figure 2B), with a long tail towards negative correlations. This was the same when considering all dosages together, although there was an observed improvement in the correlation coefficient, which may reflect a larger sample size (Figure 2C). This highlights the advantage of considering the overall pattern of consistency for drug sensitivity profiles, as opposed to considering each dose individually. Thus, studies exploring the influence of different factors on HTS results should pursue analyses reflecting variation in the entire experiment, instead of just focusing on each individual drug, cell line or dose in isolation.

**Table 1:** Datasets used in HTS study

Data Set	Doses *(um)*	MCLs	OCLs	# Drugs	CCLs	Common Drugs
SBPMDI 1 dose	10	49	0	747	37	89
SBPMDI 3 doses	.1, 1, 10	30	0	120	29	120
SBPMDI 9 doses	.02, .04, .1, .2, .4, 1, 2, 4, 10	40	0	120	40	120
TGen 9 doses	.02, .04, .1, .2, .4, 1, 2, 4, 10	4	0	120	4	120
CCLE 8 doses	.0025, .008, .025, .08, .25, .8, 2.53, 8	59	888	24	4	9
GDSC 8 doses	varied	45	1209	139	4	6

Key: CLs = Cell Lines; MCLs = Melanoma Cell Lines; OCLs = Other Cell Lines; CCLs = Melanoma cell lines in common with the SBP nine-point data set; Common drugs = drugs in common with the SBP nine-point data set; SBPMDI = Sanford Burnham Prebys Medical Discovery Institute; TGen = Translational Genomics Research Institute; CCLE = Cancer Cell Line Encyclopedia; GDSC = Genomics of Drug Sensitivity in Cancer.

**Figure 2A:**
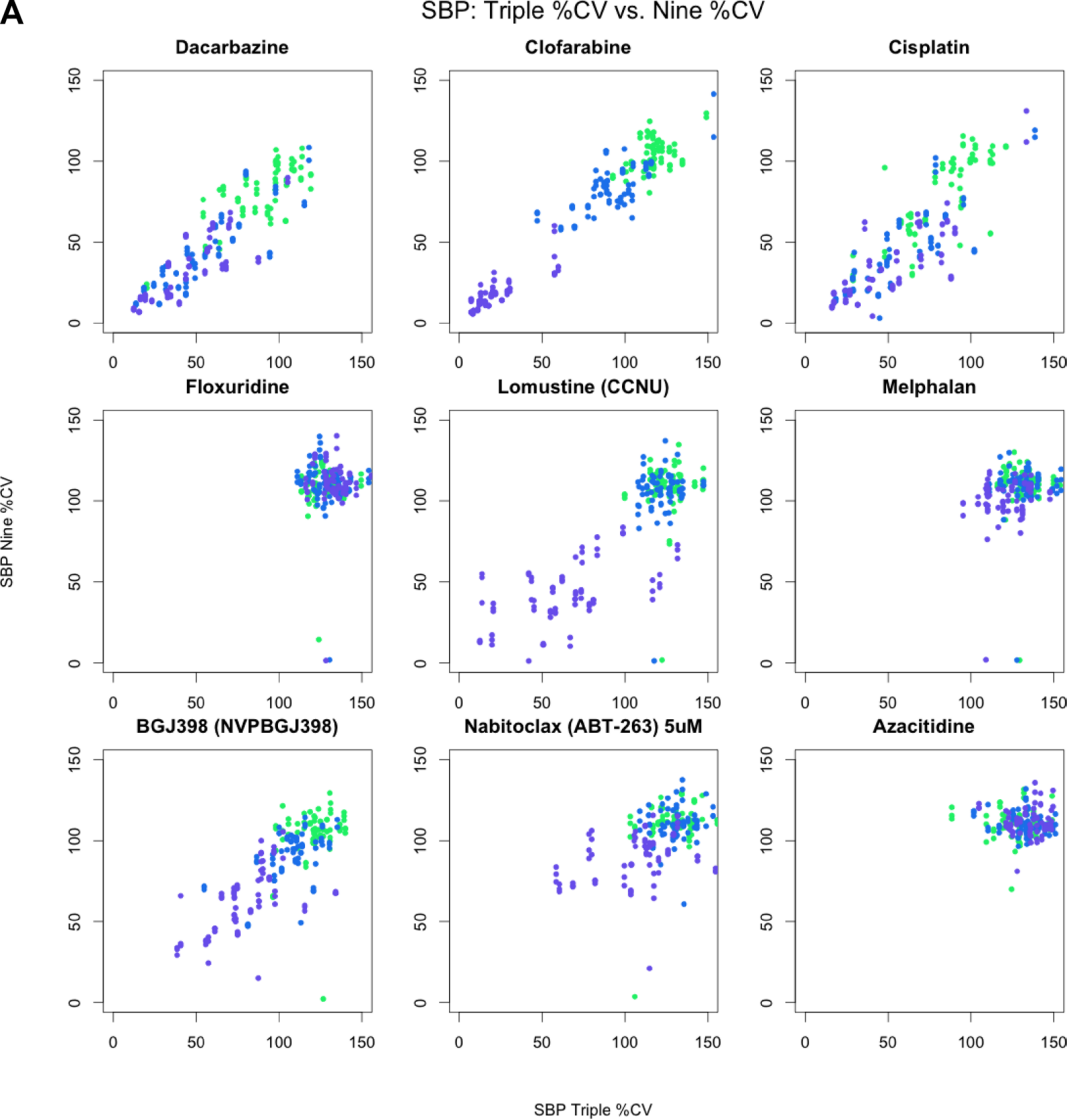
Pairwise scatterplots across nine drugs comparing SBP 3-point screen against same three concentrations from SBP 9-point screen All 128 pairwise scatterplots are provided in the supplemental figures. Colors indicate concentration: 0.1 (green), 1.0 (blue), and 10.0 μM (purple).

**Figure 2 (B, C):**
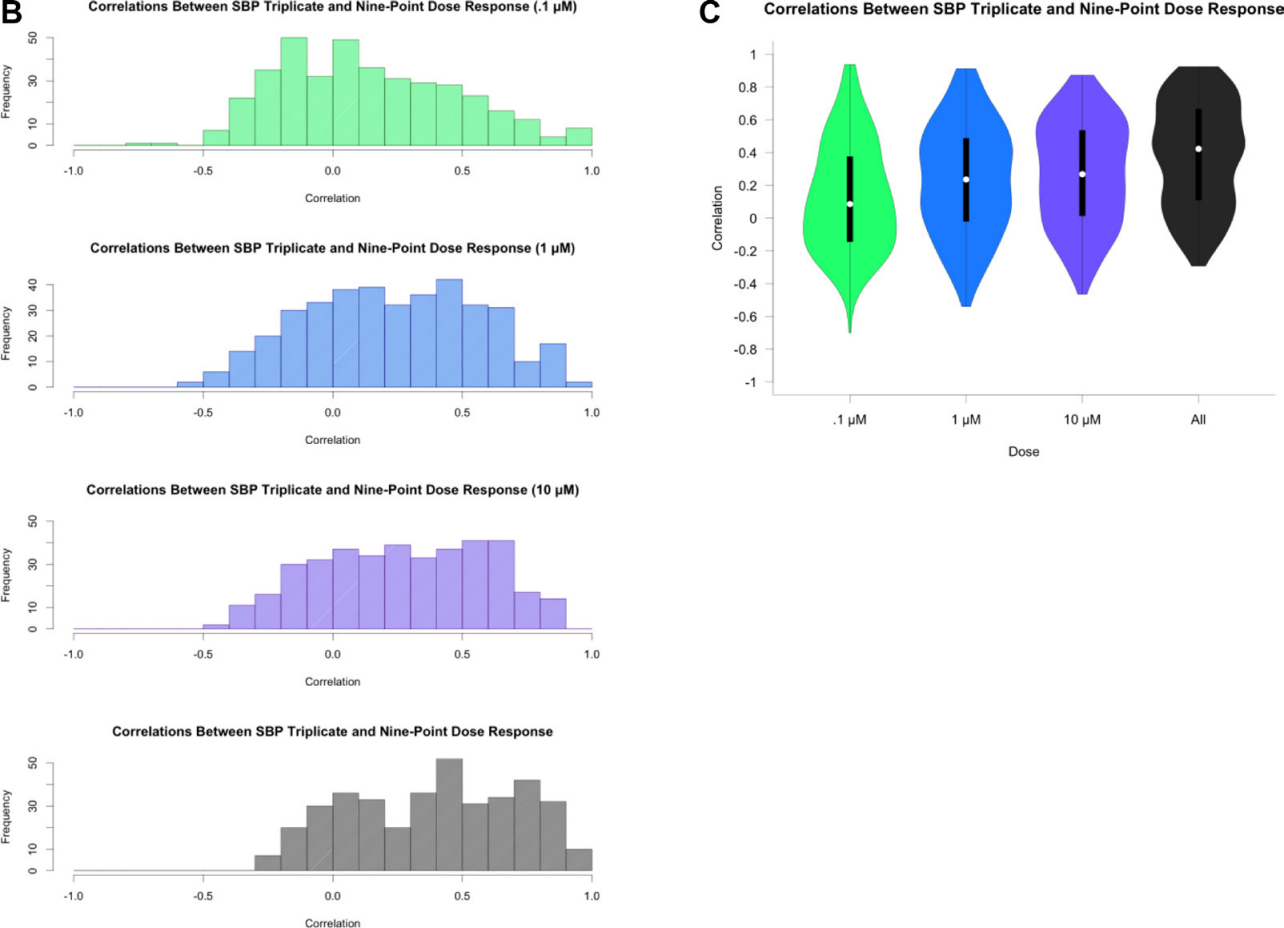
Correlation coefficients from pairwise spearman correlation across same three concentrations Colors indicate concentration: 0.1 (green), 1.0 (blue), 10.0 μM (purple), and across all three concentrations. Figure 2**B**: Histogram of correlation coefficients. Figure 2**C**: Violin plot of correlation coefficients.

**Figure 2 (D, E):**
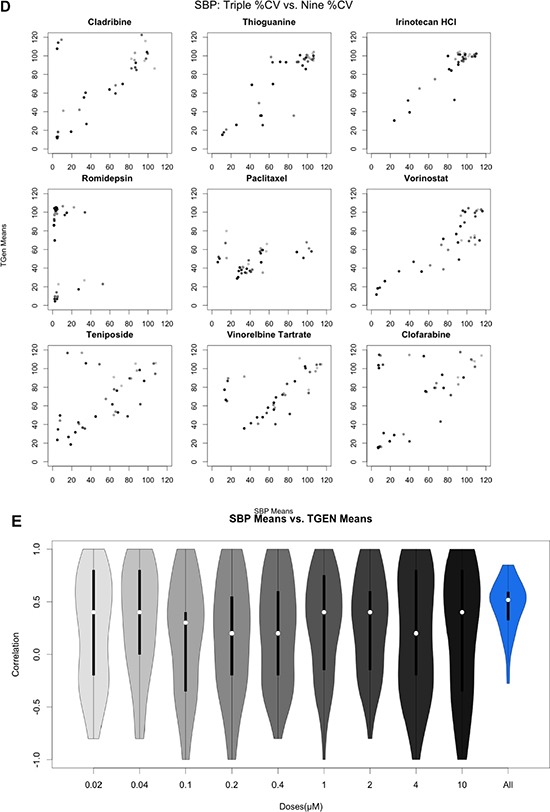
Pairwise analysis of mean Cell Viability across nine drugs exhibiting at least one CV less than 20% Gradient indicates concentration from 0.02 μM (light) to 10 μM (dark). All 46 pairwise scatterplots for drugs with at least one CV less than 20% is provided in the supplemental figures. Figure 2**D**: Pairwise scatterplots comparing SBP 9-point HTS against TGen 9-point HTS. Figure 2**E**: Violin plot of correlation coefficients.

### Variation between two laboratories

For drugs with at least one cell line exhibiting a 20% cell viability at higher doses, which is consistent with a drug response (*n* = 46), we also calculated the Spearman correlation coefficient between the SBP and TGen response data for each of the nine concentrations and also across all nine concentrations (Figure 2D, Figure 2E, Supplementary Figure 2). The correlation coefficients across all available dosages were greater than 0.0 in 44 out of the 46 drugs (Bonferroni-adjusted *p-value* < .05 in 22 of the 44 drugs). Individual pairwise scatterplots for each dose across the 46 drugs indicate that for a large number of them, there is a high degree of between-laboratory consistency. As in the three-concentration drug response analysis performed, the drug responses were more consistent at higher doses and across all dosages when considered together (Figure 2E, Supplementary Figure 2). The stronger correlation at higher dosages, especially in the context of lower doses that do not induce an effect or response, reveal technical variation and “noise” that should be considered in analyses seeking to identify bona fide drug-induced effects producing signals that rise above this experiment-wise noise. Likewise, the improved correlation coefficients observed when comparing the nine-concentration dose-response curves (DRCs) against the three-concentration DRCs suggest that a full range of DRCs may be better at revealing true biological variation in the HTS data.

### Comprehensive analysis considering the entire HTS experimental setting via ANOVA modeling

For a more comprehensive assessment of the factors contributing to the variation in drug response associated with our HTS studies, we applied flexible linear regression modeling within an ANOVA context. We ultimately wanted to partition the variation in cell viability data arising from the entire HTS study into factors representing different experimental and biological conditions (i.e., across all plates on which the samples were arranged, drugs, drug concentrations, cell lines, and laboratories; Supplementary Table 1). We limited this analysis to the four cell lines in common at the two independent laboratories. In order to conduct the modeling appropriately, we initially had to choose a comparator to be contrasted with all the other subgroupings for each of the different factors we studied. This was achieved by creating simple zero (absence) or one (presence) dummy variables for each factor subgroup. We randomly selected SBP as the comparator laboratory, Cladribine as the comparator drug, plate 36 (from the SBP experiment) as the comparator plate, cell line A375 as the comparator cell line, and the lowest dose (0.02 μM) as the comparator dose. Our analysis found that the laboratory used explained 0.028% of the variation in drug response, whereas plate (3.23%) and other biological factors such as drugs (45.5%), concentration (5.24%), and cell lines (4.94%) explained approximately 60% of the variation (Table 2). To identify the individual factors that were most statistically significant sources of variation, we carried out simple *t*-tests on each factor's regression coefficient. We used a conservative Bonferroni-correction to accommodate the multiple tests. The results suggested that laboratory was only marginally significant factor relative to the others (Figure 3A, Supplementary Figure 3). Analysis of the cell lines indicated that two of the four cell lines, MeWo and SK-MEL-2, had an effect on drug responses that were statistically significantly different from the comparator cell line A375. This could be due to the BRAF mutation status in the cell lines: A375 (the comparator cell line) and UACC-0257 are cell lines with the BRAF V600E mutation, whereas MeWo and SK-Mel-2 are BRAF wild type cell lines. Obviously, more work on this hypothesis is needed before attributing differences to the presence of the BRAF V600E mutation. Additionally, the higher concentrations (i.e., 2.0, 4.0, and 10.0 μM) were the most statistically significant, consistent with the existence of overall dose-response relationships in the experiment.

**Table 2:** Percentage of variance explained by experimental factors

	Df	Sum Sq	Mean Sq	*F* value	Pr(> F)	% Variance explained
Cell Lines	3	1409075	469692	1064.446	< 2.2e-16	4.94
Lab (SBP)	1	7975	7975	18.0742	2.13E-05	0.03
Log Dose	8	1493445	186681	423.0677	< 2.2e-16	5.24
Drug	119	12966053	108958	246.9287	< 2.2e-16	45.46
Plates	65	921052	14170	34.236	< 2.2e-16	3.23
Residuals	25723	11721774	441			

Percentage of variance explained by Cell Lines, Site, Dose, Drug, and Plates.

Interestingly, we also found that a large number of SBP plates were moderately statistically different from other plates both used at SBP and TGen; however, a single TGen plate (plate 25) was highly significant. Furthermore, only one SBP plate yielded a more significant t-statistic (plate 27) than the TGen plate 25 (Supplementary Table 2). As expected, the outlying plates produced DRCs with greater variability (e.g., Mitoxantrone DRCs for UACC-0257 at 0.1, 1.0, and 10.0 μM for SBP plate 27 and at 0.02, 0.2, and 2.0 μM for TGen plate 25, Figure 3B). Although plate-effects explained 3.23% of the variation in cell viability, the small number of highly significant plates suggests that plate analysis and ways of accommodating plate effects in HTS data analyses, or subsequent removal of outlying plates, should be performed to assess the overall quality of the HTS data and potentially lead to explanations for why some drugs don't replicate across site-specific studies (e.g., because some plates were outlying and should be removed as opposed to a more global analysis and rationale). The reasons for plate effects should be explored, but could reflect contamination, technician, or robot error when setting up the plate or experiment in question. Importantly, if the drugs, concentrations and cell lines used on the plate led to biologically meaningful effects, then one would not be able to separate the biological significance from a potential technical artifact, which suggests the use of controls and designed plating schemes are necessary. Finally, we found that most drugs in the study exhibited strong, statistically significant *p*-values, far beyond what would be expected by chance alone.

**Figure 3A:**
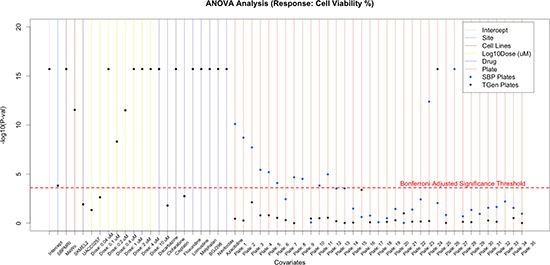
Application of flexible linear models, using ANOVA methods, to explore the variation in CV that is explained by site, cell lines, dose, drug, and plates Sites explained a small proportion of the variation, whereas drugs, dose, and cell lines explained a majority of the variation observed in CV. Nine of the 120 drugs are plotted; however, plots with all drugs are available in supplemental figures.

**Figure 3B:**
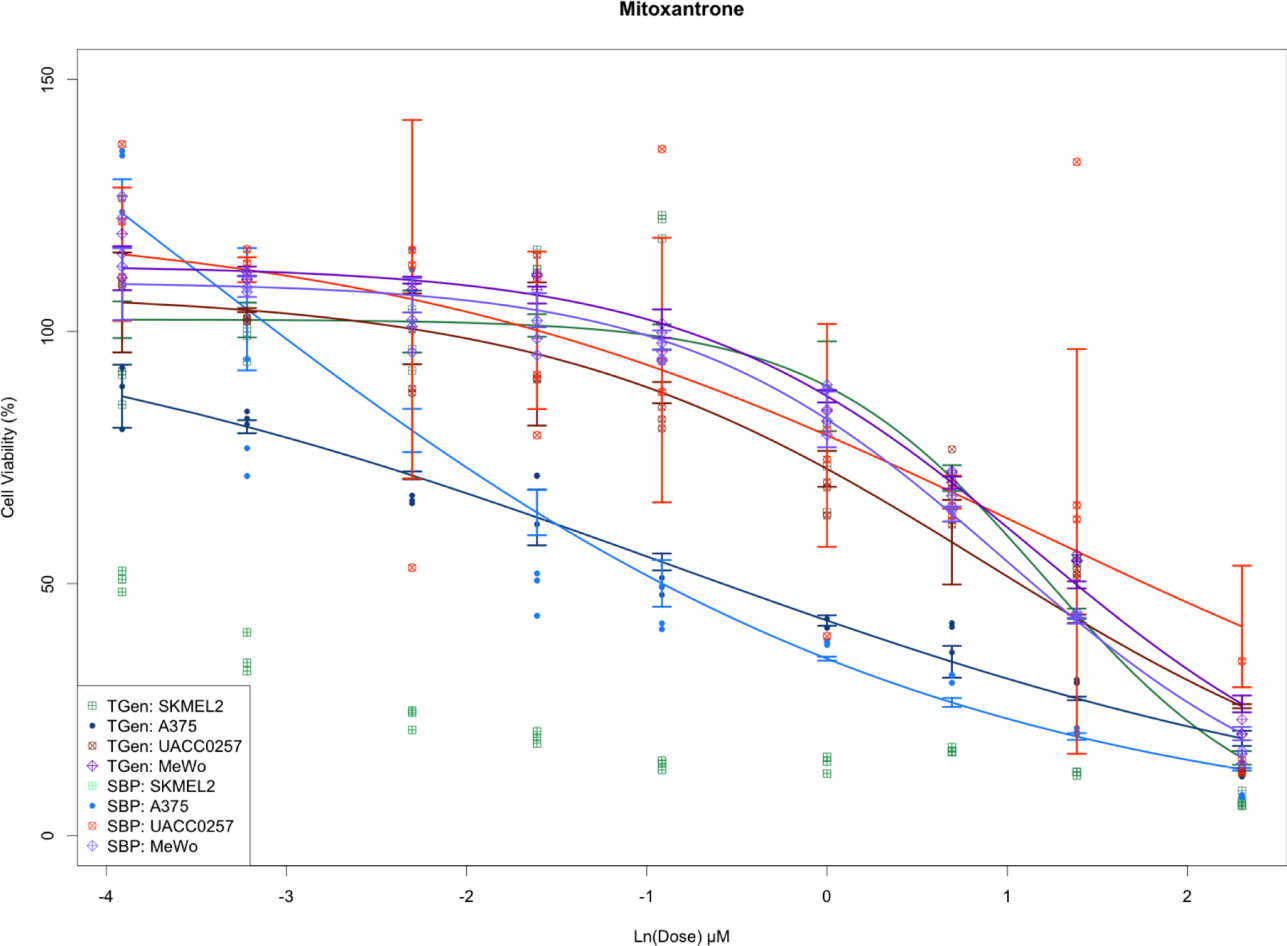
Plots of dose response curves for each of the cell line and site combinations Greater variance is observed in concentration from outlying plates detected by ANOVA-like methods.

**Figure 3C:**
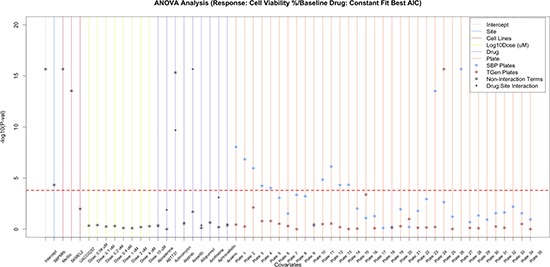
Assessing the variation in CV that is explained by site, cell lines, dose, drug, and drug-site interaction To further assess the reproducibility of HTS, we examined the drug-site interaction terms and found that 19 of the significant drugs did not have a significant drug-site interaction term. Nine of the 120 drugs are plotted; however, plots with all drugs are available in supplemental figures.

### Exploring interaction effects

To identify whether specific drugs, cell lines, doses, and sites were significant predictors of drug response (i.e., cell viability) while considering other factors, we added interaction terms to the linear models. When we accounted for laboratory x drug interactions, we found that many of the drugs exhibited significant interaction term *p*-values; in fact many more than would be expected by chance alone. These results indicate that although drugs may influence variation in cell viability across the experiment as a whole, some of the cell viability variation may be laboratory-specific (Supplementary Table 3). 19 drugs yielded significant drug *p*-values, yet non-significant drug-laboratory interaction effects (Figure 3C, Supplementary Figure 4), indicating that these drugs exhibited overtly reproducible effects (Supplementary Table 4). As expected, when we incorporated the drug x dose interaction terms into our analysis models, we found that a majority of the variation significantly accounted for by dose was limited to higher concentrations, which of course makes sense (Supplementary Figure 5). Importantly, drug x laboratory interaction effects only explained an additional 3.94% of the cell viability variation, whereas drug x dose interaction effects explained an additional 11.02% of the variation in cell viability.

The consideration of three-way interaction effects led to additional insights into the factors contributing to cell viability in a pronounced enough way to rise above the “noise” in cell viability across the HTS experiments as a whole. Laboratory × drug x dose effects explained 2.5% of cell viability variance and drug x dose x cell line explained 3.6% of the variance (Supplementary Table 5). These findings provided further evidence that laboratory effects were not as influential on the experiment as a whole relative to other factors. While nine drugs appeared to have laboratory-specific effects (i.e., a non-significant effect when considering the drug alone, but a significant drug x laboratory interaction effect), drug x dose interactions had the largest impact on the drug-associated cell viability, which it to be expected (Supplementary Figure 6). Again, we identified a subset of drug x dose x cell line interaction terms that were significant (Supplementary Table 6), suggesting that there may be some heterogeneity among the cell lines (likely due to genetic differences) affecting the drug response. This is important in that it suggests unique cell-line features may indicate efficacy of certain drugs, which is consistent with the goal of personalized medicine. The significant differences in cell viability across the cell lines generally appear in the intermediate concentration ranges (Supplementary Figure 7).

### Re-analysis with a broader set of comparator drugs and cell lines

To investigate whether the use of an arbitrary drug as the comparator drug impacted our initial analyses in any way, we set out to identify a group of cell line and drug combinations that did not exhibit any dose-response or general drug effect. These combinations would then effectively act as a group of controls whose cell viabilities represent simple noise and technical variation when compared to the other cell line-drug combinations. These analyses would allow us to see if compelling drug or drug x cell line effects can be identified that rise above the noise and variation exhibited in the HTS experiment as a whole. As noted in the Methods section, we fit a three-parameter sigmoidal, a four-parameter sigmoidal, a linear, and a constant model to each of the cell lines for each drugs and dosages to characterize dose response curves. We then selected the drug-cell line pairs where the constant model fit the data the best based on the Akaike Information Criterion (AIC), since this would be indicative of no evidence of a drug or dose-dependent response. The set of drug x cell line combinations that suggested no evidence for a drug or dose-dependent response was then used as the comparator group in a re-analysis of the cell viability data. The re-analysis suggested that the laboratory in which the assays were done explained a minimal proportion of the variance (0.03%). However, drug effects explained 41.2%, dose 5.24%, cell line 4.94%, and plates 3.23% of the variation in cell viability across the experiment (Supplementary Table 7). Interestingly, this re-analysis greatly reduced the number of statistically significant drug effects, suggesting that only a small subset of drugs studied may be exhibiting actual effects that rise above the “noise” in cell viability across the experimental setting as a whole (Supplementary Figures 8–11 and Supplementary Tables 7, 8, 9, 10).

### Comparing the results of models that accommodate plate-specific effects to those that do not

To assess the impact that technically-deficient or outlying plates have on the interpretation of HTS experiments, we assessed the statistical significance of the effects on cell viability of each drug in the context of the entire experiment using our linear model. We used the drugs that did not exhibit evidence for a dose response relationship as a comparator group, as discussed in the previous section. When not accommodating any site, plate, and dose (since doses were plated in triplicates on each plate) effects in the model, we found 54 significant drugs with drug-effect *p*-values surpassed Bonferroni-adjusted significance thresholds (Supplementary Table 11). When the site, plate and dose effects were considered in the model, we found 44 drugs with drug effect *p*-values that surpassed Bonferroni-adjusted significance thresholds (Supplementary Table 11). Strikingly, only 28 of the 44 drugs (64%) resulting from these analyses were in common, further confirming that experimental factors ultimately affect downstream analyses as well as the interpretation of analyses meant to identify drugs with pronounced effects (Supplementary Table 11).

### Analysis of CCLE and GDSC data

**Figure 4:**
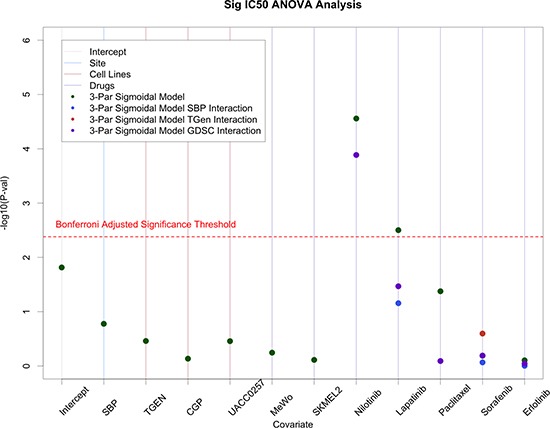
ANOVA analysis on IC50 values from SBP, TGen, GDSC, and CCLE Site effects were generally minimal; however, site specific effects were observed in Nilotinib.

We used ANOVA analyses on IC_50_ values provided in the CCLE and GDSC data sets along with our SBP and TGen data on the four cell lines and six common drugs they had in common. In these models, we randomly selected CCLE as the comparator laboratory, the drug Crizotinib as the comparator drug, and cell line A375 as the comparator cell line. Similar to the analysis comparing the raw SBP and TGen data, we found that laboratories were not significant predictors of IC_50_ values when we also accommodated drug, cell line, and laboratory effects in the model. We note, however, that we did observe some laboratory-specific effects for one of the six drugs, Nilotinib (Figure 4).

## DISCUSSION

Although there has been a considerable amount of interest and development in HTS technologies, there have been few attempts at standardizing protocols and ensuring the reliability and reproducibility of the resulting data. Many sources of variation implicated in HTS studies, such as the laboratory used to pursue the experiment, technicians, reagents and version of reagents used, plating schemes, cell culture conditions and dosing ranges, will inevitably have an effect on the resulting data. However, properly recording and accommodating these factors in the analysis of HTS data can aide in not only an assessment of the quality of the data, but also the interpretation of the results.

We sought to quantify the sources of variability in an HTS setting in order to gauge the, reliability and reproducibility of the resulting data. We did this using HTS data from a study on melanoma cell lines and simple correlation analyses coupled with often-used ANOVA modeling methods. The methods we discussed and applied can be used with any HTS data set including many of those in the public domain, although many studies have either used methods like we have proposed or different methods for assessing the concordance between datasets [9] of all sorts (e.g., gene expression microarray, next generation sequencing, reverse phase protein arrays, etc.). Using the ANOVA setting, we, for example, identified evidence that certain plates used in the experiment were problematic and thus could undermine the reliability of the HTS experiment as whole if not accounted for properly.

We chose not to construct dose-response curves (DRCs) for each cell line for each drug and then compare, e.g., IC_50_ values extracted from those curves across sites or among different conditions, since this would ultimately ignore variation introduced by different technical factors impacting dose effects on cell viability (e.g., plate effects) and hence the DRCs. In this context, once outlying plates have been identified, it is possible to reassess the reliability of the HTS data using the traditional methods while excluding those outlying plates (alternatively, one could obtain coefficients associated with significant experimental effects and weight them accordingly). Additionally, ANOVA methodology allows for the simultaneous analysis of all cell viability data across all drugs to identify true and very compelling signals that have been obtained in a HTS experiment. Although the specific plating scheme in our experiments (Figure 1) allowed for the simultaneous analysis of various factors, it could also lead to confounding effects between plate and dose because triplicate concentrations were constrained to individual plates (i.e., one of three concentrations sets were used for each plate: concentrations 1, 4, and 7; concentrations 2, 5, and 8; and concentrations 3, 6, and 9).

In terms of specific findings, we observed that laboratory effects only explained a small fraction (0.03%) of the cell viability variation in our HTS setting. This contrasts with the results of studies described in recent publications about the reliability of the data associated with two very large cancer cell line screens pursued by different groups at different sites [5, 6, 8, 9]. We also observed that the main source of variation in our study could be attributed to the drugs used–further indicating that in certain settings, HTS data may indeed be reliable. By considering site x drug interactions in our analyses, we identified drugs with reproducible effects. Our consideration of three-way interaction terms allowed us to identify some heterogeneity among the cell line response to drugs, suggesting that genetic differences among cell lines may explain why some cell lines are responsive to certain drugs. This is consistent with the notion that *in vitro* drug screening can shed light on the potential for personalized medicine [17]. We also find that the downstream analyses of drug association tests are greatly impacted by whether one accommodates or does not accommodate experimental and technical factors, such a plating scheme, in the analysis models. In this light, we found that many of the drugs identified without experimental factors were false positives. Similarly, we find that many drugs that were significantly associated with drug response were missed when the models did not accommodate experimental factors. Our experience suggests that interaction terms in ANOVA-like analysis settings should be considered in order to tease out important and compelling drug associations that would otherwise go undetected due to the masking of particular drug effects by “noise.”

Ultimately, assessing the variability, reliability and reproducibility of HTS data by designing the study to contrast different experimental conditions could add to the overall experimental costs. As a result, alternative methods that can account for sources of variation are needed. Our analysis at the very least highlights the importance of using consistent dosing and plating schemes that include specific controls, which would allow researchers to not only measure and test for the impact of experimental factors on the outcomes, but also adjust for these specific experimental effects when making claims about, e.g., drug and cell-line specific effects. Thus, ANOVA-like methods can accommodate different experimental factors that may influence the HTS assays in pronounced ways and also reveal compelling signals attributable to drugs and experimental compounds. Although our analysis was performed only within the context of melanoma, and, importantly, only within the context of four melanoma cell lines and 120 drugs, our overall ANOVA-based approach can be used to reassess the quality of publicly available data as well as additional HTS data.

## MATERIALS AND METHODS

### Data

We initially performed a nine-concentration (i.e., dose) HTS study (drug concentrations: 0.02, 0.04, 0.1, 0.2, 0.4, 1.0, 2.0, 4.0, and 10.0 μM) on 40 melanoma cell lines across 120 drugs (Table 1) at Sanford Burnham Prebys Medical Discovery Institute (SBP). We used 384-well plates with three concentrations of a drug assigned to each plate across all 120 drugs in triplicate (Figure 1). Drugs were spotted on 384-well clear bottom tissue culture treated plates (Greiner Bio-One, #781098) using an Echo Liquid Handler (Labcyte Inc., Sunnyvale, CA) such that addition of 25 μLs of cells (100 k cells/mL in RPMI + 10% FBS +Pen./ Strep./ Glut., Omega Scientific, Tarzana, CA) resulted in the above described final drug concentrations and 2.5 k cells/well. Upon plating, the cells were gently spun down at 1 k rpm for one minute and incubated with drugs for 96 hours at 37°C in a standard tissue culture incubator. After this time course, plates were allowed to equilibrate to room temperature for 30 minutes before 10 μLs per well of freshly prepared CellTiterGlo reagent (G7571, Promega Corp., Madison WI) were added. Samples were incubated for ten minutes with gentle agitation (100 rpm) before luminescence was read on a BioTek Synergy2 plate reader using Gen5 software (BioTek, Winooski, VT).

Each plate was assayed in triplicate and included 24 vehicle only DMSO controls. In total, nine plates were used for each cell line. For each cell line, drug, and drug concentration combination, cell viability (CV) measures were obtained post drug administration and were normalized to the plate-specific average DMSO cell:

$$CV_{norm}=\frac{100*cellCount_{Drug}}{cellCount_{DMSO}}$$

We also pursued an independent three-concentration HTS study (drug concentrations: 0.1, 1.0, and 10.0 μM) at SBP across 30 melanoma cell lines (29 in common with the nine-point screen) on all 120 drugs as described above. Finally, we performed the primary nine-concentration HTS study across all 120 drugs for four melanoma cell lines in common with the initial nine-point SBP screen (four screened cell lines: UACC-0257, MeWo, SK-Mel-2, and A375) at the Translational Genomics Research Institute (TGen). For the TGen HTS study, the experimental protocols were similar to the SBP screen, with minor differences. Specifically, the compounds were pre-spotted to white, solid-bottom 384-well assay plates (Greiner Bio-One) using ATS (Biosero, San Diego, CA). Additionally, prior to measuring the luminescence using an Analyst GT plate reader (Molecular Devices, Sunnyvale, CA), 25 uL CellTiterGlo reagent (G7571, Promega Corp., Madison WI) was added to assay plates and incubated at room temperature for one hour (as opposed to ten minutes at SBP).

MeWo and A-375 cell lines were obtained directly from American Type Culture Collection, all SK- cell lines were received directly from Memorial Sloan Kettering Cancer Center, TGen generated all UACC- cell lines, and all were of low passage number. Cells were maintained according to the manufacturer's or collaborator's instructions: A-375 cells are grown using DMEM medium, MeWo and Sk-Mel-2 cells are grown using EMEM medium, and UACC-0257 cells are grown using RPMI1640 medium. All media have 10% FBS and 1%AA added to final growth media. All cell lines were banked at low passages in multiple aliquots as liquid nitrogen stocks to reduce risk of phenotypic drift. All cells were cultured for less than three months before reinitiating culture from the frozen stock. All cells were routinely inspected for identity by morphology and growth curve analysis and validated to be mycoplasma free. All cell lines were free of contaminants.

### Data analysis

We assessed the variability of the HTS data by first examining the correlations between measures obtained at matching doses using the two SBP screens (i.e., nine-concentration and three-concentration screens) and then across the SBP and TGen screens (i.e., the two nine-concentration screens). We calculated the Spearman correlation of the CV_norm_ between the data obtained from each experimental pair setting at each dose (i.e., single concentration pairs) and the correlation coefficient across all available doses (i.e., all three-concentration doses when comparing the two SBP screens or all nine-concentration doses when comparing SBP and TGen screens). Since the Spearman correlation is robust to outliers, is non-parametric, and does not necessarily assume linear relationships, we chose use it as implemented in base stats package [18] in R. We assessed the normality of the data using Shapiro-Wilk test in the base stat package [11] for R

For a more comprehensive assessment of the assays’ variability and reliability on the four common cell lines across institutes, we used flexible linear models and ANOVA (also within the base stats [11] package in R) to simultaneously assess drug effects (across all drugs) as well as all other experimental (e.g., plate and lab) and biological (e.g., drug, dose, and cell line) factor effects by creating (0, 1) dummy variables for each factor (e.g., we had 120–1 = 119 dummy variables for the drugs, 9–1 = 8 for doses, 4–1 = 3 for cell lines, 72–1 = 71 for plates, and one for site). After combining the lab, plate, drug, dose, and cell line factors, there were a total of 202 factor effects. Notably, we performed a multi-factorial analysis in an unbalanced experimental design. Under this approach, ANOVA in the context of linear regression models was used to assess the proportion of variation that can be attributed to each factor (e.g., laboratory, plate, drug, dose, and cell line). Interaction terms between the factors were also used to assess the non-additive influence and combinations of different factors. We then fit dose-response curves (DRCs) to each of the SBP and TGen nine-concentration HTS experiments using four separate models: a four-parameter sigmoidal model, a three-parameter sigmoidal model, a linear model, and a constant model (i.e., no dose-response effect). We fit these DRCs using the nplr [19] package in R. We used the Akaike Information Criterion [20] (AIC) from the base stats [11] package in R to identify the “best” model for each cell line and drug combination. The AIC provides an assessment of model performance while considering the number of parameters used for the model. Thus, this approach allowed us to assess the fit of each model while adjusting for the number of parameters assumed within those models. We did this to identify individual drugs that exhibited no evidence of a dose-response relationship. The drugs and cell lines that exhibited no evidence of a dose response effect were then treated as a set of “controls” for the other drugs because the variation they exhibited across the different doses reflect noise-induced and technical variation. We used this set of control drug-cell line combinations in re-analysis with the ANOVA model, coding the controls as the baseline drug (i.e. for the “controls”, binary variables for each drug were set to 0 in the model). To evaluate the impact of controlling for plate-specific effects, we performed drug association tests using linear models with interaction terms. We compared models that included and excluded plate effects. The significance of each drug's effects in the context of the entire experiment was determined based on a Bonferroni-adjusted [21] significance threshold.

Because we were unable to access the raw experimental data for the Cancer Cell Line Encyclopedia (CCLE) data, we explored the consistency of the IC_50_ values provided from the CCLE [5] repository along with IC_50_ values from the Genomics of Drug Sensitivity in Cancer [6] (GDSC) data repository. We also computed the IC_50_ values from our SBP and TGen data using three-parameter sigmoidal curve fits. The four cell lines in common between the SBP and TGen data sets were also available for the CCLE and GDSC data, but only six drugs were in common. Further, replicates were only available in the GDSC dataset, plating schemes used between the sites were inconsistent, and there were differences in dose-concentrations between same cell line and drug combinations from the various datasets. Nevertheless, we explored the variation in the HTS data using information on the different sites (*n* = 4), cell lines (*n* = 4), and drugs (*n* = 6). All analyses were performed in R and all figures were generated using the graphics^11^ package in R.

## SUPPLEMENTARY MATERIALS FIGURES AND TABLES














