# Supplementary material for: Analysis of variability in high throughput screening data: applications to melanoma cell lines and drug responses
Source: Oncotarget. 2017 Feb 15;8(17):27786–99. doi: 10.18632/oncotarget.15347 (PMC5438608; doi:10.18632/oncotarget.15347)
Supplement: Supplementary file 1 [file oncotarget-08-27786-s001.pdf]

Analysis of variability in high throughput screening data: applications to melanoma cell lines and drug responses

Supplementary Materials

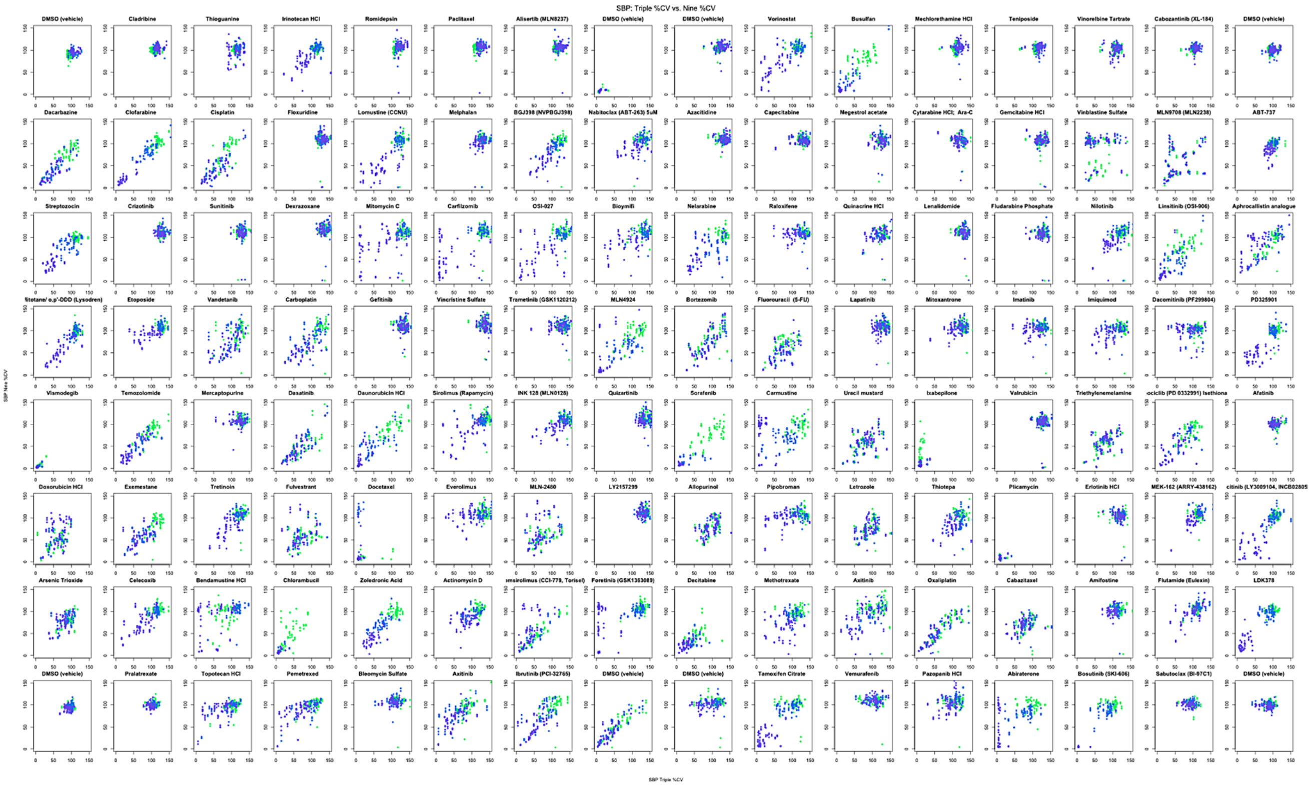

Supplementary Figure 1: Pairwise scatterplots across all 128 drugs comparing SBP 3-point screen against same three concentrations from SBP 9-point screen. Colors indicate concentration: 0.1 (green), 1.0 (blue), and 10.0 µM (purple).

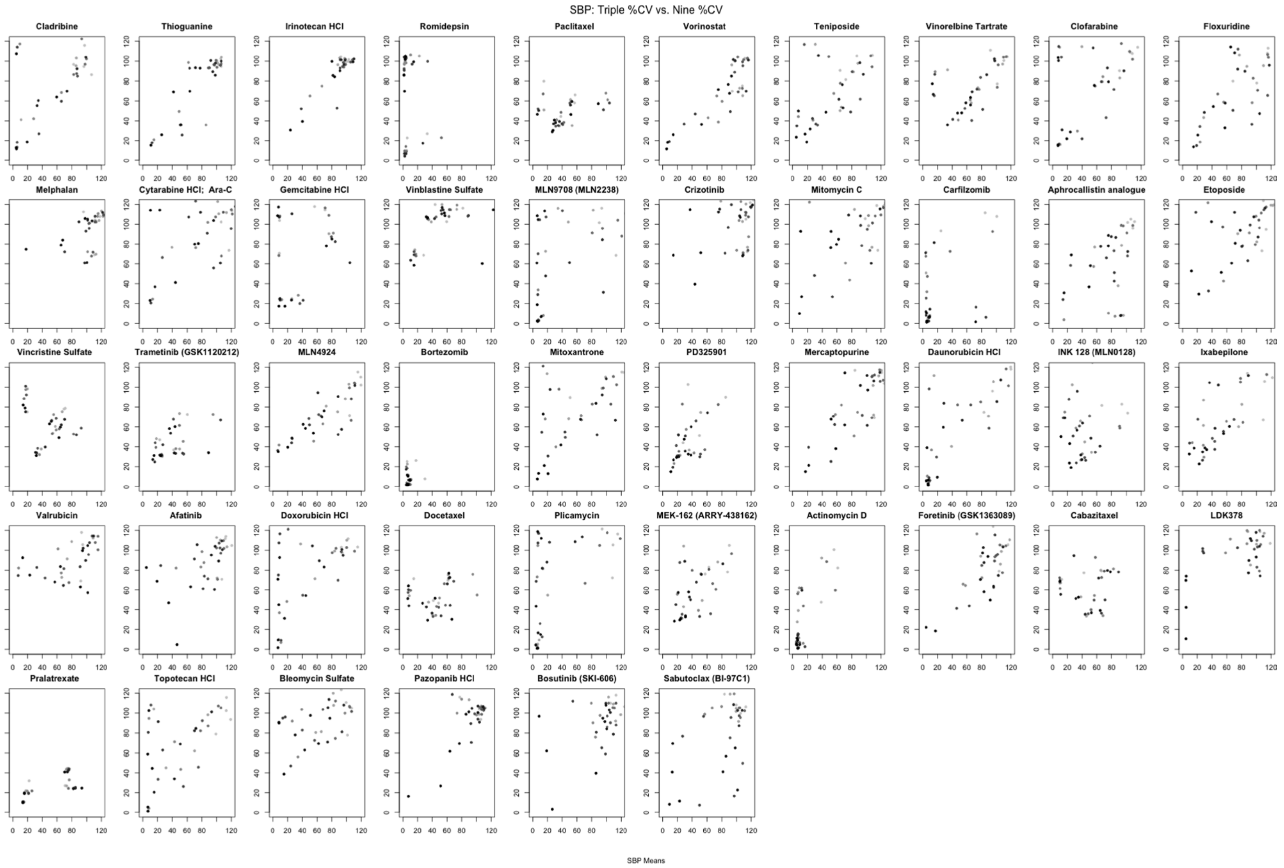

Supplementary Figure 2: Pairwise analysis of mean Cell Viability across 46 drugs exhibiting at least one CV less than 20%. Gradient indicates concentration from 0.02 μM (light) to 10 μM (dark).

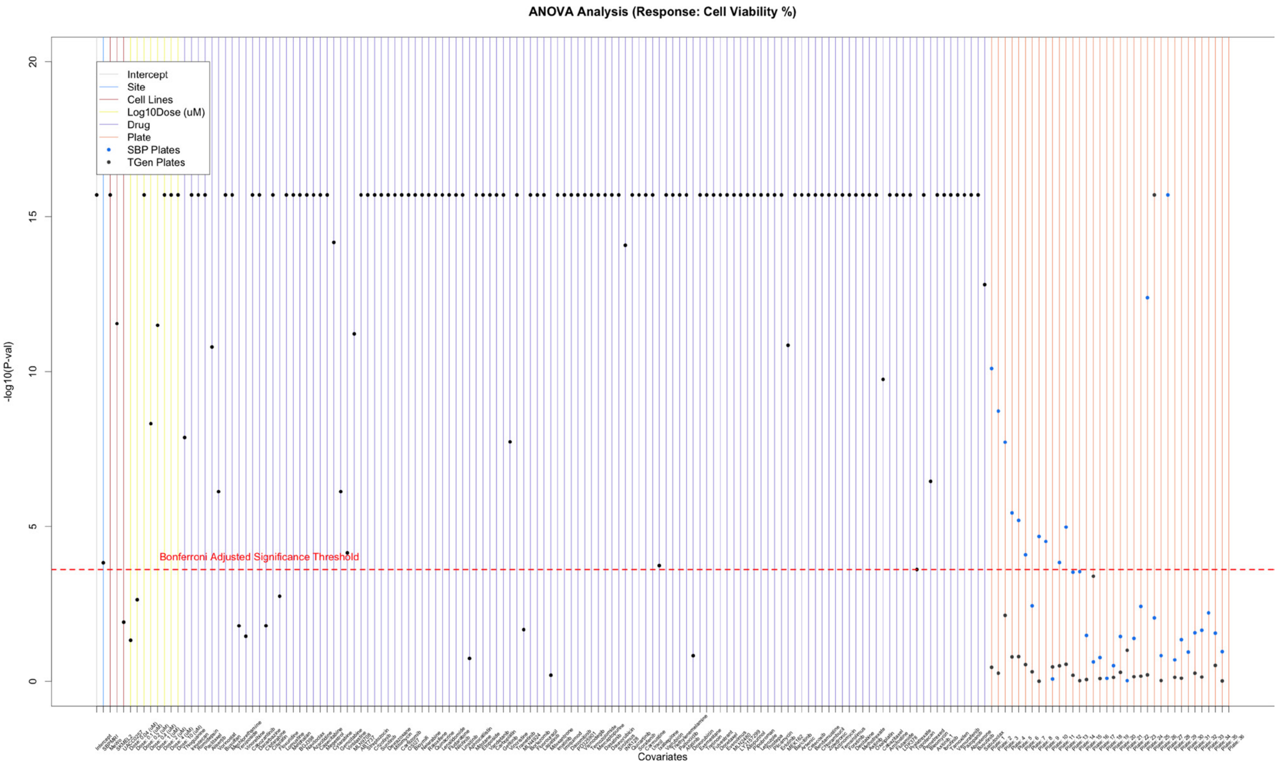

Supplementary Figure 3: Application of flexible linear models, using ANOVA methods, to explore the variation in CV that is explained by site, cell lines, dose, drug, and plates. Sites explained a small proportion of the variation, whereas drugs, dose, and cell lines explained a majority of the variation observed in CV.

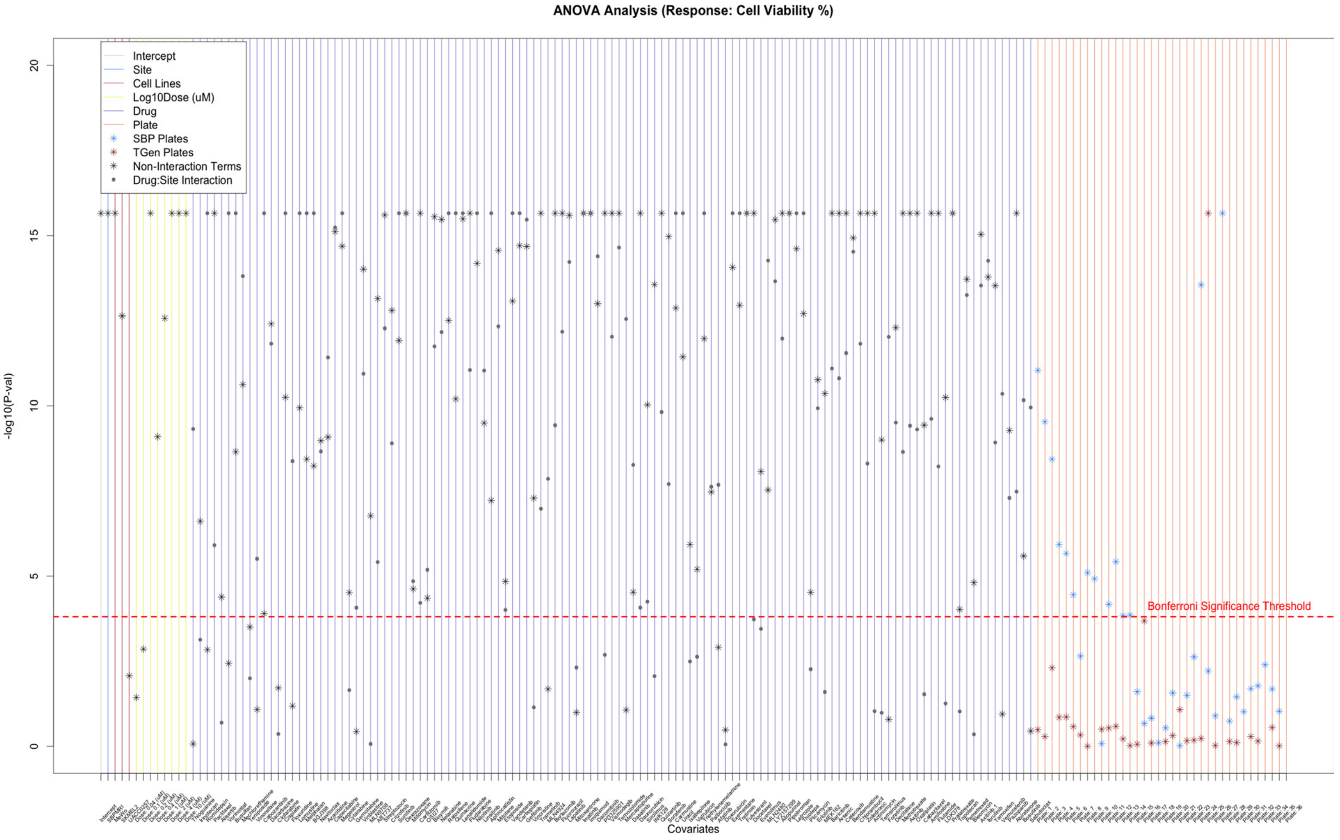

Supplementary Figure 4: Assessing the variation in CV that is explained by site, cell lines, dose, drug, and drug-site interaction. To further assess the reproducibility of HTS, we examined the drug-site interaction terms and found that 19 of the significant drugs did not have a significant drug-site interaction term.

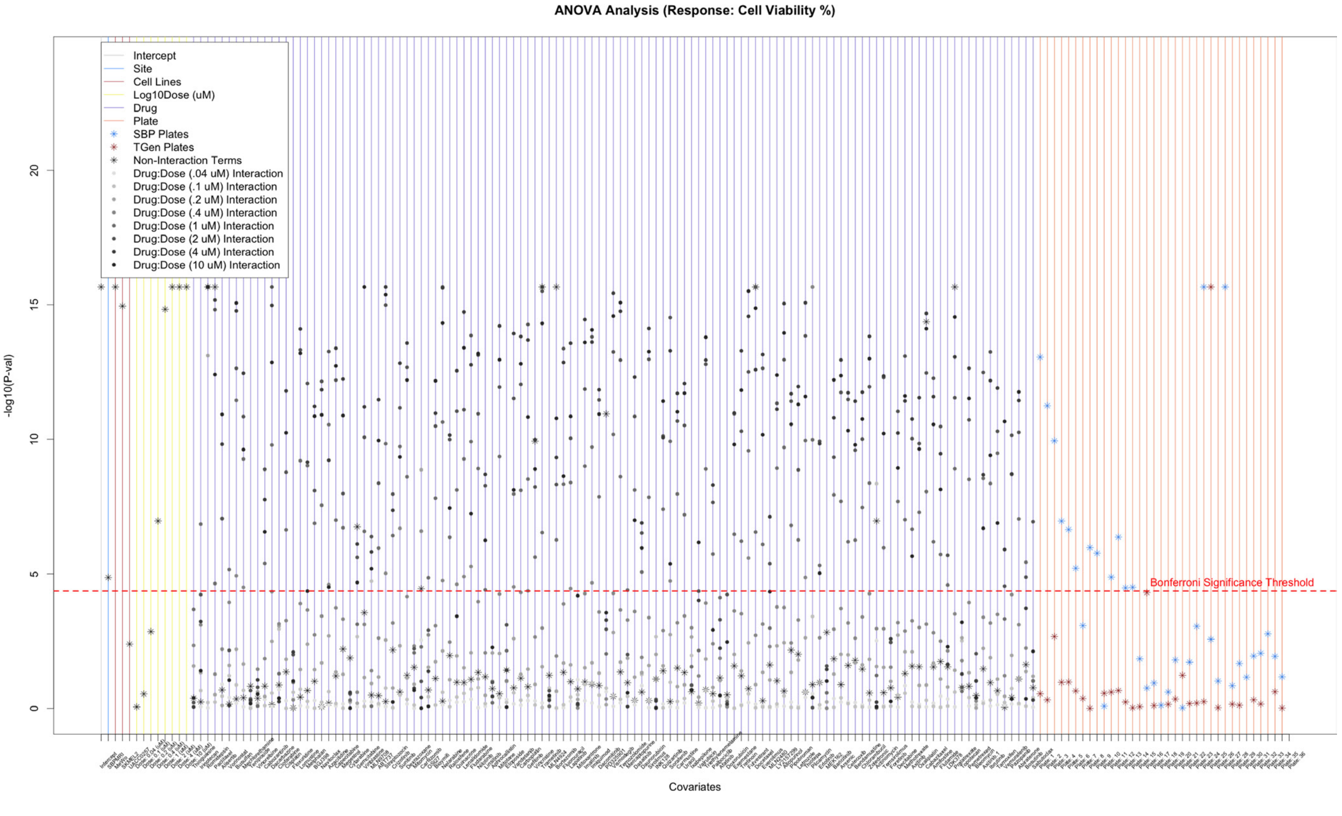

Supplementary Figure 5: Assessing the variation in CV that is explained by site, cell lines, dose, drug, and drug-dose interaction. We find that many of the significant drug-dose interaction terms were from higher concentrations, indicating that a majority of the variation significantly accounted for by dose was limited to higher concentrations.

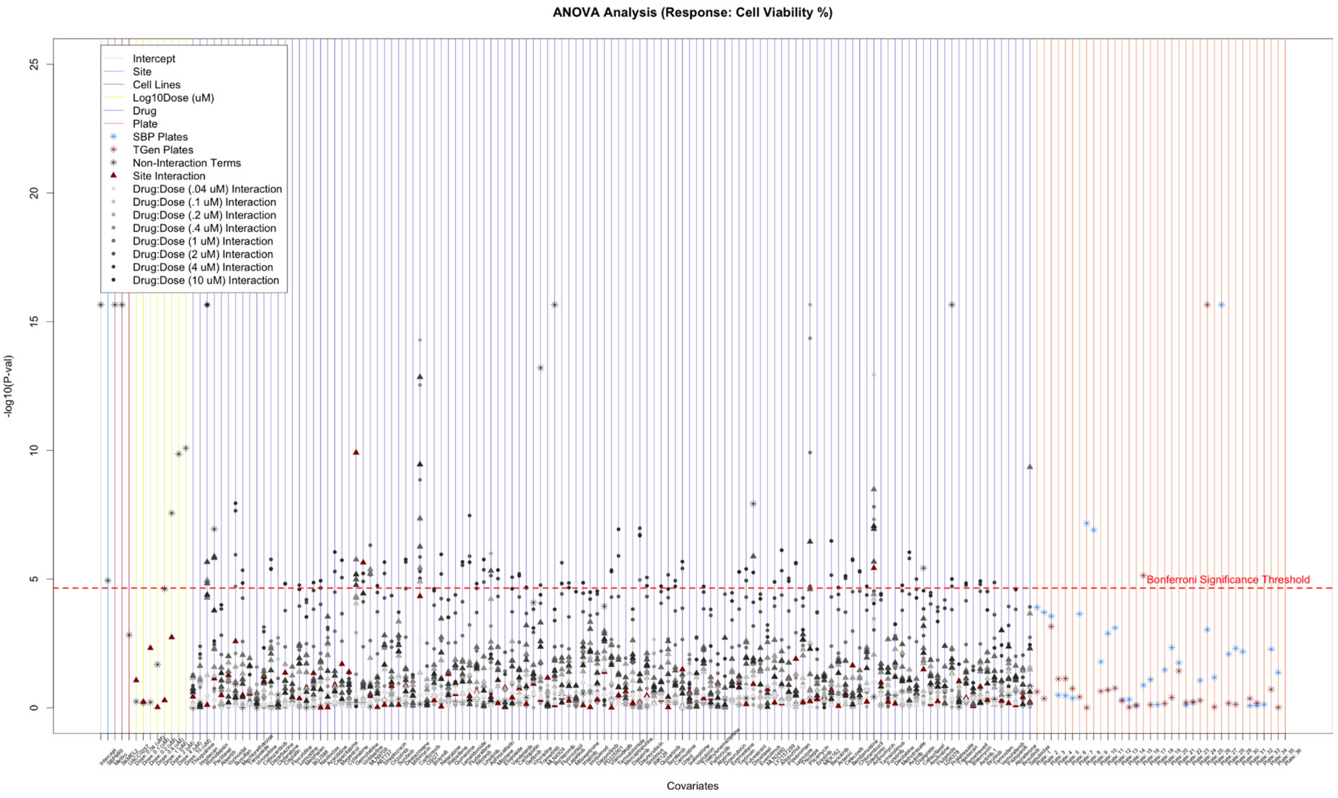

Supplementary Figure 6: Assessing the variation in CV that is explained by site, cell lines, dose, drug, and 3-way interaction terms. Site-by-drug-by-dose effects explained 2.49% of cell viability variance.

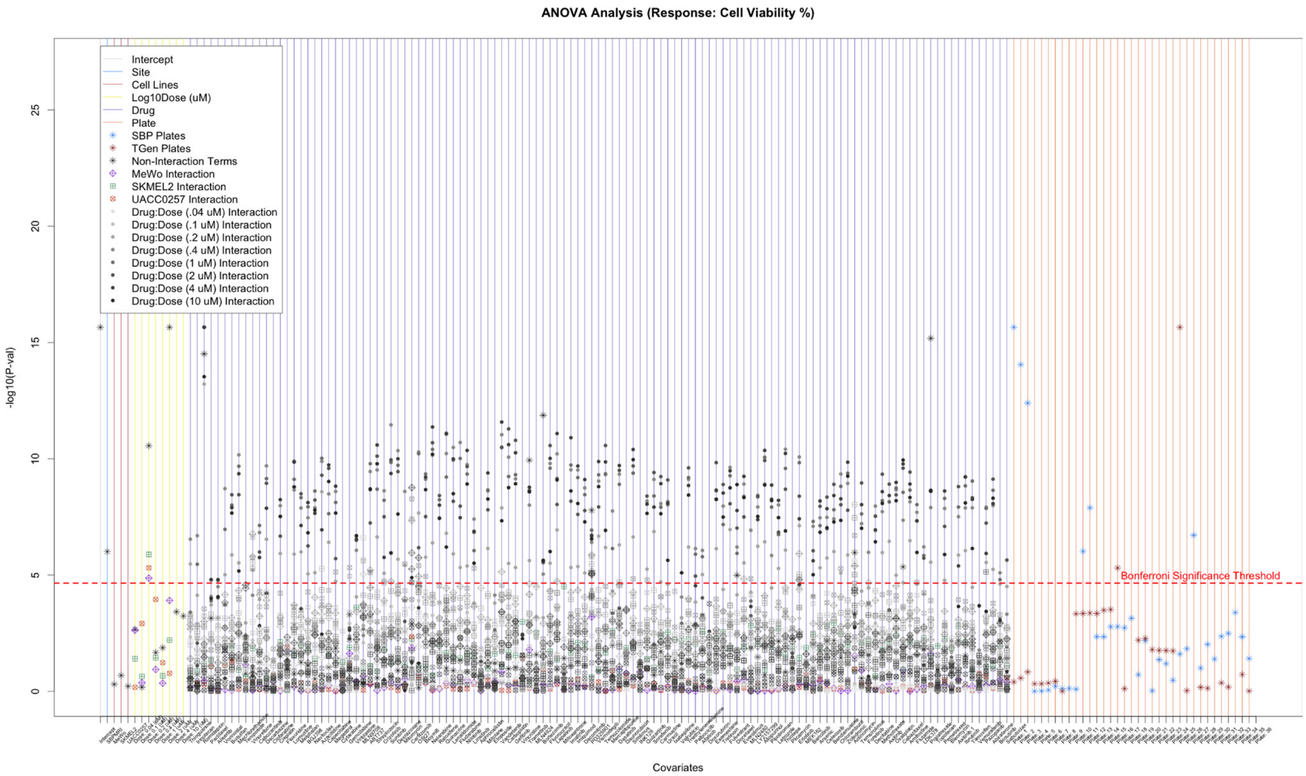

Supplementary Figure 7: Assessing the variation in CV that is explained by site, cell lines, dose, drug, and 3-way interaction terms. Drug-by-dose-by-cell line explained 3.6% of the variance.

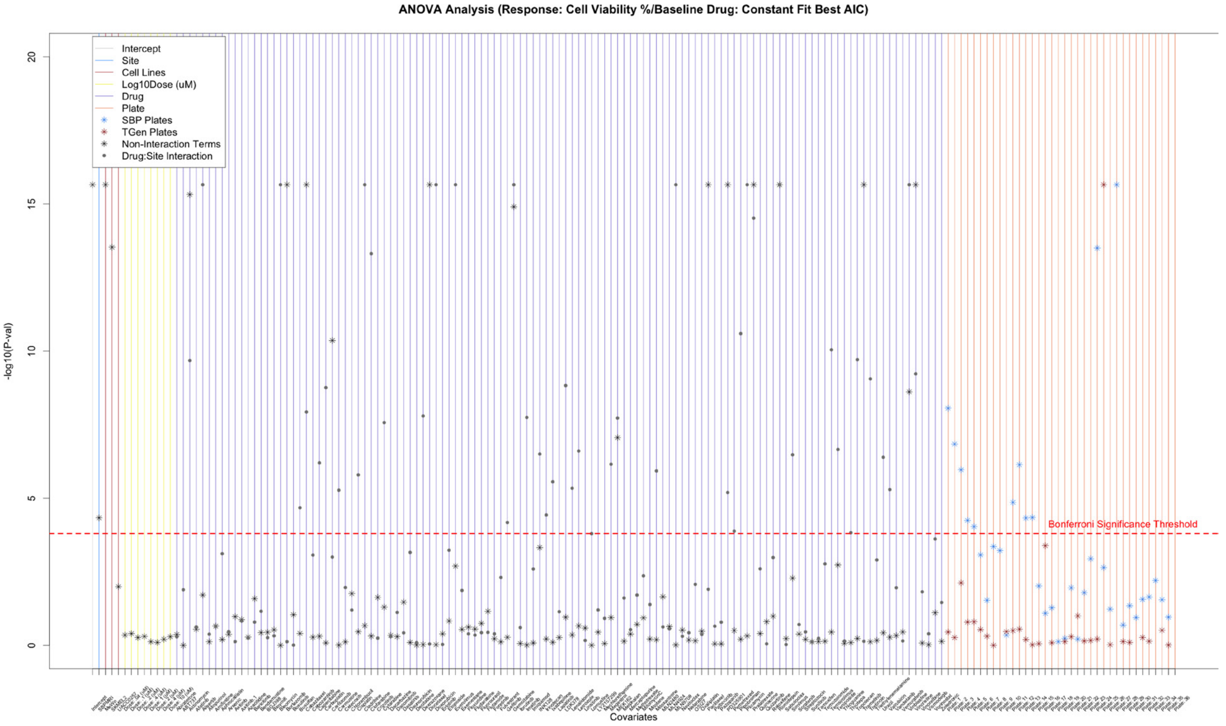

Supplementary Figure 8: Re-assessing the variation in CV that is explained by site, cell lines, dose, and drug when using AIC-defined control drugs. Results were similar from using random baseline drug in terms of proportion of variation explained by sites, cell lines, dose, and drug. However, a smaller number of statistically significant drug effects were observed.

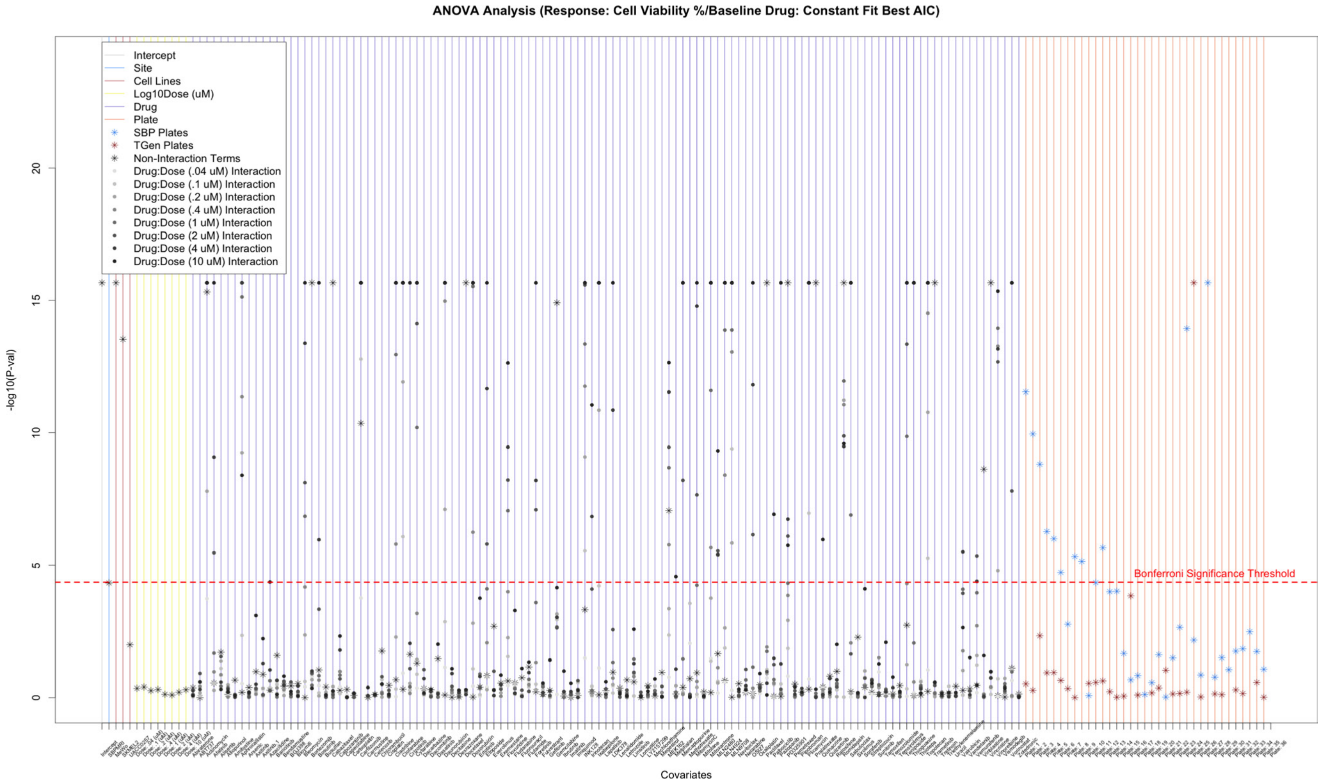

Supplementary Figure 9: Re-assessing the variation in CV that is explained by site, cell lines, dose, drug, and dose-drug interaction when using AIC-defined control drugs.

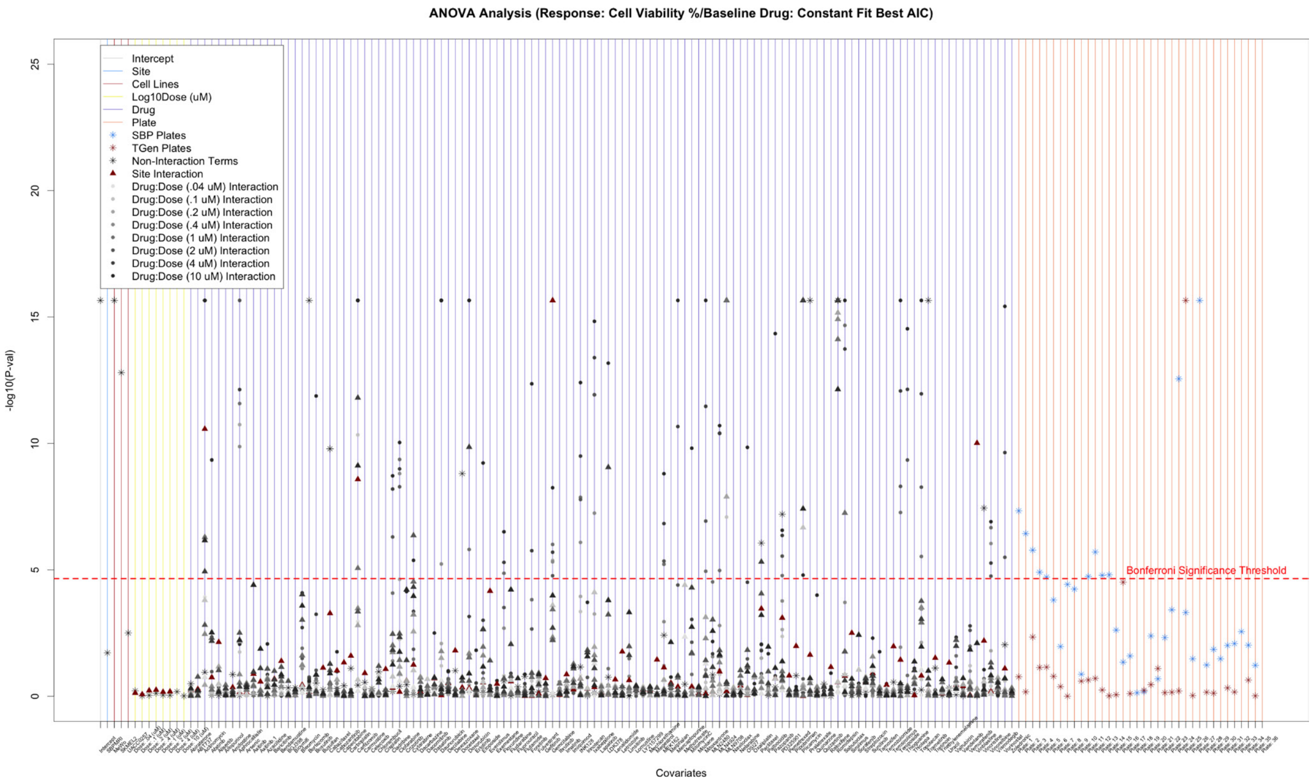

Supplementary Figure 10: Re-assessing the variation in CV that is explained by site, cell lines, dose, drug, and site-dose-drug interaction when using AIC-defined control drugs.

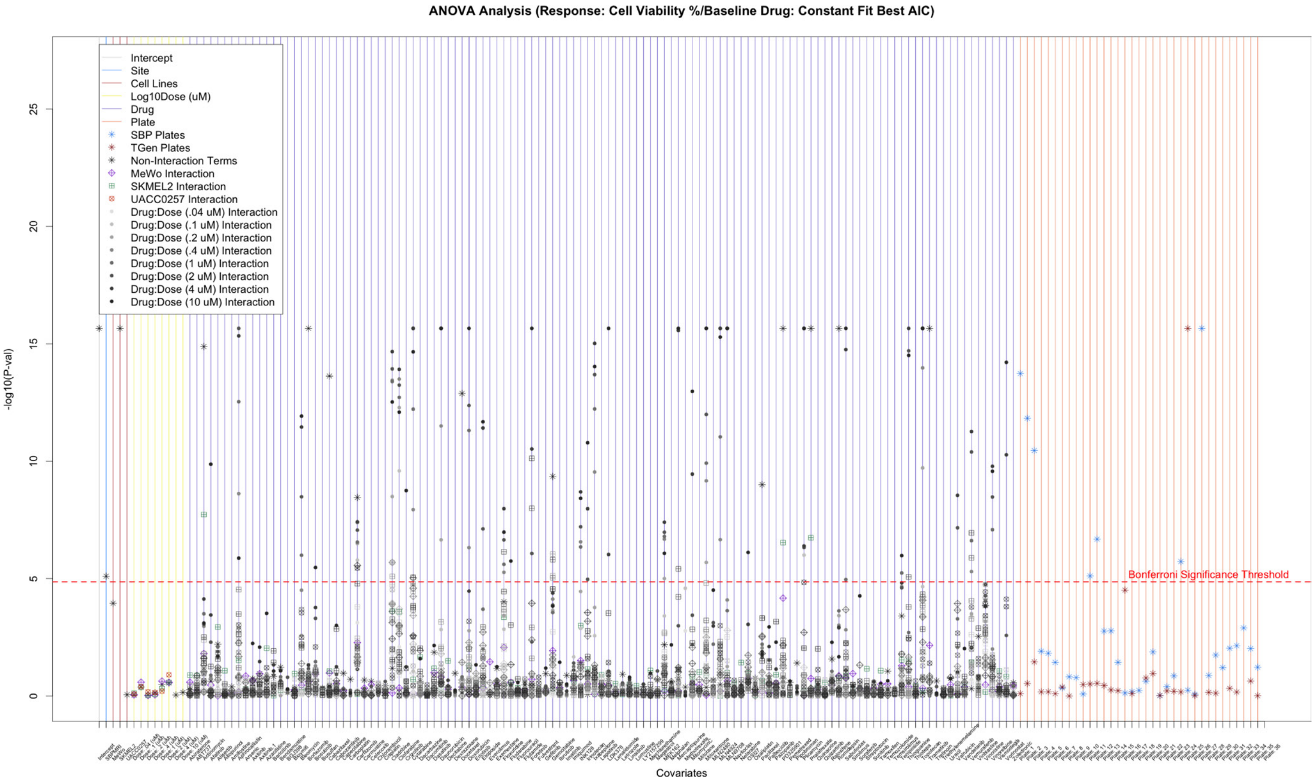

Supplementary Figure 11: Re-assessing the variation in CV that is explained by site, cell lines, dose, drug, and cell line-dose-drug interaction when using AIC-defined control drugs.

Supplementary Table 1: Factors availale in SBP and TGen datasets. See Supplementary_Table_1

Supplementary Table 2: Significance of Each Covariate. See Supplementary_Table_2

Supplementary Table 3: Percentage of variance explained by experimental factors with interaction

	Df	Sum Sq	Mean Sq	F value	Pr(> F)	% Var Explained
CellLine	3	1409075	469692	1134.805	< 2.2e-16	4.94
SBP	1	7975	7975	19.269	1.14E-05	0.03
Drug	119	12966053	108958	263.25	< 2.2e-16	45.46
Ldose	8	1493445	186681	451.032	< 2.2e-16	5.24
Plates	65	921052	14170	34.236	< 2.2e-16	3.23
SBP:Drug Interaction	119	1124367	9448	22.828	< 2.2e-16	3.94
Residuals	25604	10597407	414			

Percentage of Variance Explained by Experimental Factors, including Site:Drug Interaction.

Supplementary Table 4: Factor significance in model with drug:site interaction. See Supplementary_Table_4

Supplementary Table 5: Percentage of variance explained by experimental factors with 3-Way interactions. See Supplementary_Table_5

	Df	Sum Sq	Mean Sq	F value	Pr(> F)	% Var Explained
SBP	1	7975	7975	29.1919	6.63E-08	0.03
CellLine	3	1409075	469692	1719.2045	< 2.2e-16	4.94
Drug	119	12966053	108958	398.8186	< 2.2e-16	45.46
Ldose	8	1493445	186681	683.3036	< 2.2e-16	5.24
Plates	65	921052	14170	51.8663	< 2.2e-16	3.23
CellLine:Drug Interaction	357	1600815	4484	16.413	< 2.2e-16	5.61
CellLine:Ldose Interaction	18	66789	3711	13.5815	< 2.2e-16	0.23
Drug:Ldose Interaction	952	3143874	3302	12.0877	< 2.2e-16	11.02
CellLine:Drug:Ldose Interaction	2856	1025504	359	1.3143	< 2.2e-16	3.60
Residuals	21540	5884792	273			

Percentage of variance explained by the experimental factors, including cell line:drug:dose 3-way interaction.

Supplementary Table 6: Factor significance in ANOVA analysis for site, dose, cell line, plate, and drug-dose-cell line interaction effects. See Supplementary_Table_6

Supplementary Table 7: Percentage of variance explained by the experimental factors, including drug:site interaction using an AIC derived set of control drugs as baseline

	Df	Sum Sq	Mean Sq	F value	Pr(> F)	% Var Explained
CellLine	3	1409075	469692	1028.647	< 2.2e-16	4.94
SBP	1	7975	7975	17.466	2.93E-05	0.03
Drug	119	11741176	98665	216.082	< 2.2e-16	41.17
Ldose	8	1493445	186681	408.839	< 2.2e-16	5.24
Plates	65	921052	14170	31.033	< 2.2e-16	3.23
SBP:DrugInteraction	119	1255580	10551	23.107	< 2.2e-16	4.40
Residuals	25604	11691071	457			

Supplementary Table 8: Factor significance in analysis with site, dose, cell line, plate, and drug-site interaction. See Supplementary_Table_8

Supplementary Table 9: Percentage of variance explained by the experimental factors, including cell line:drug:dose 3-way interaction using an AIC derived set of control drugs as baseline

	Df	Sum Sq	Mean Sq	F value	Pr(> F)	% Variance Explained
SBP	1	7975	7975	24.311	8.26E-07	0.03
CellLine	3	1409075	469692	1431.763	< 2.2e-16	4.94
Drug	119	11741176	98665	300.762	< 2.2e-16	41.17
Ldose	8	1493445	186681	569.059	< 2.2e-16	5.24
Plates	65	921052	14170	43.195	< 2.2e-16	3.23
CellLine:Drug Interaction	273	1496897	5483	16.714	< 2.2e-16	5.25
CellLine:Ldose Interaction	18	66789	3711	11.311	< 2.2e-16	0.23
Drug:Ldose Interaction	952	3173127	3333	10.16	< 2.2e-16	11.13
CellLine:Drug:Ldose Interaction	2184	895607	410	1.25	1.86E-13	3.14
Residuals	22296	7314232	328			

Supplementary Table 10: Factor significance in analysis for site, dose, cell line, plate, and drug-dose-cell line interaction effects using an AIC derived set of control drugs as baseline. See Supplementary_Table_10

Supplementary Table 11: Drugs significantly associated with dose response with and without adjusting for experimental factors

Drugs Sig w/o Exp Factors Correction	Drugs Sig w/ Exp Factors Correction	Drugs Significant Both
Actinomycin.D	Actinomycin.D	Actinomycin.D
Afatinib	Alisertib..MLN8237.	Alisertib..MLN8237.
Alisertib..MLN8237.	Bleomycin.Sulfate	Bleomycin.Sulfate
Aphrocallistin.analogue	Busulfan	Cabazitaxel
Bleomycin.Sulfate	Cabazitaxel	Cladribine
Bortezomib	Capecitabine	Clofarabine
Bosutinib..SKI.606.	Carboplatin	Cytarabine.HCl...Ara.C
Cabazitaxel	Carmustine	Doxorubicin.HCl
Carfilzomib	Cisplatin	Everolimus
Chlorambucil	Cladribine	Gemcitabine.HCl
Cladribine	Clofarabine	INK.128..MLN0128.
Clofarabine	Cytarabine.HCl...Ara.C	Irinotecan.HCl
Cytarabine.HCl...Ara.C	Dexrazoxane	Ixabepilone
Dasatinib	Doxorubicin.HCl	LDK378
Daunorubicin.HCl	Everolimus	Megestrol.acetate
Docetaxel	Gefitinib	MEK.162..ARRY.438162.
Doxorubicin.HCl	Gemcitabine.HCl	Mitoxantrone
Etoposide	Imatinib	MLN9708..MLN2238.
Everolimus	INK.128..MLN0128.	PD325901
Floxuridine	Irinotecan.HCl	Plicamycin
Foretinib..GSK1363089.	Ixabepilone	Pralatrexate
Gemcitabine.HCl	LDK378	Romidepsin
INK.128..MLN0128.	Lenalidomide	Sirolimus..Rapamycin.
Irinotecan.HCl	Letrozole	Teniposide
Ixabepilone	Megestrol.acetate	Topotecan.HCl
LDK378	MEK.162..ARRY.438162.	Valrubicin
Megestrol.acetate	Mitoxantrone	Vinblastine.Sulfate
MEK.162..ARRY.438162.	MLN9708..MLN2238.	Vincristine.Sulfate
Mercaptopurine	PD325901	
Mitomycin.C	Pemetrexed	
Mitoxantrone	Pipobroman	
MLN.2480	Plicamycin	
MLN4924	Pralatrexate	
MLN9708..MLN2238.	Romidepsin	
OSI.027	Sirolimus..Rapamycin.	
Paclitaxel	Temsirolimus..CCI.779..Torisel.	
Palbociclib..PD.0332991..Isethionate	Teniposide	
Pazopanib.HCl	Thiotepa	
PD325901	Topotecan.HCl	
Plicamycin	Tretinoin	
Pralatrexate	Uracil.mustard	
Romidepsin	Valrubicin	
Sabutoclax..BI.97C1.	Vinblastine.Sulfate	
Sirolimus..Rapamycin.	Vincristine.Sulfate	
Teniposide		
Thioguanine		
Topotecan.HCl		
Trametinib..GSK1120212.		
Valrubicin		
Vemurafenib		
Vinblastine.Sulfate		
Vincristine.Sulfate		
Vinorelbine.Tartrate		
Vorinostat		
