# Supplementary material for: Analysis of variability in high throughput screening data: applications to melanoma cell lines and drug responses
Source: Oncotarget. 2017 Feb 15;8(17):27786–99. doi: 10.18632/oncotarget.15347 (PMC5438608; doi:10.18632/oncotarget.15347)
Supplement: Supplementary file 2 [file oncotarget-08-27786-s002.docx]

**Supplemental Table 1:** Factors Availale in SBP and TGen Datasets.

| Factors in SBP and TGen Dataset: | | | | |
| --- | --- | --- | --- | --- |
| Drugs | Plates | Doses (μM ) | Cell Lines | Sites |
| Abiraterone | TGen Plate 1 | 0.02 | MeWo | SBPMDI |
| ABT737 | TGen Plate 2 | 0.04 | SK-MEL-2 | TGen |
| Actinomycin | TGen Plate 3 | 0.1 | A-375 |  |
| Afatinib | TGen Plate 4 | 0.2 | UACC-0257 |  |
| Alisertib | TGen Plate 5 | 0.4 |  |  |
| Allopurinol | TGen Plate 6 | 1 |  |  |
| Amifostine | TGen Plate 7 | 2 |  |  |
| Aphrocallistin | TGen Plate 8 | 4 |  |  |
| Arsenic | TGen Plate 9 | 10 |  |  |
| Axitinib | TGen Plate 10 |  |  |  |
| Axitinib | TGen Plate 11 |  |  |  |
| Azacitidine | TGen Plate 12 |  |  |  |
| Baricitinib | TGen Plate 13 |  |  |  |
| Bendamustine | TGen Plate 14 |  |  |  |
| BGJ398 | TGen Plate 15 |  |  |  |
| Bioymifi | TGen Plate 16 |  |  |  |
| Bleomycin | TGen Plate 17 |  |  |  |
| Bortezomib | TGen Plate 18 |  |  |  |
| Bosutinib | TGen Plate 19 |  |  |  |
| Busulfan | TGen Plate 20 |  |  |  |
| Cabazitaxel | TGen Plate 21 |  |  |  |
| Cabozantinib | TGen Plate 22 |  |  |  |
| Capecitabine | TGen Plate 23 |  |  |  |
| Carboplatin | TGen Plate 24 |  |  |  |
| Carfilzomib | TGen Plate 25 |  |  |  |
| Carmustine | TGen Plate 26 |  |  |  |
| Celecoxib | TGen Plate 27 |  |  |  |
| Chlorambucil | TGen Plate 28 |  |  |  |
| Cisplatin | TGen Plate 29 |  |  |  |
| Cladribine | TGen Plate 30 |  |  |  |
| Clofarabine | TGen Plate 31 |  |  |  |
| Crizotinib | TGen Plate 32 |  |  |  |
| Cytarabine | TGen Plate 33 |  |  |  |
| Dacarbazine | TGen Plate 34 |  |  |  |
| Dacomitinib | TGen Plate 35 |  |  |  |
| Dasatinib | TGen Plate 36 |  |  |  |
| Daunorubicin | SBPMDI Plate 1 |  |  |  |
| Decitabine | SBPMDI Plate 2 |  |  |  |
| Dexrazoxane | SBPMDI Plate 3 |  |  |  |
| Docetaxel | SBPMDI Plate 4 |  |  |  |
| Doxorubicin | SBPMDI Plate 5 |  |  |  |
| Erlotinib | SBPMDI Plate 6 |  |  |  |
| Etoposide | SBPMDI Plate 7 |  |  |  |
| Everolimus | SBPMDI Plate 8 |  |  |  |
| Exemestane | SBPMDI Plate 9 |  |  |  |
| Floxuridine | SBPMDI Plate 10 |  |  |  |
| Fludarabine | SBPMDI Plate 11 |  |  |  |
| Fluorouracil | SBPMDI Plate 12 |  |  |  |
| Flutamide | SBPMDI Plate 13 |  |  |  |
| Foretinib | SBPMDI Plate 14 |  |  |  |
| Fulvestrant | SBPMDI Plate 15 |  |  |  |
| Gefitinib | SBPMDI Plate 16 |  |  |  |
| Gemcitabine | SBPMDI Plate 17 |  |  |  |
| Ibrutinib | SBPMDI Plate 18 |  |  |  |
| Imatinib | SBPMDI Plate 19 |  |  |  |
| Imiquimod | SBPMDI Plate 20 |  |  |  |
| INK128 | SBPMDI Plate 21 |  |  |  |
| Irinotecan | SBPMDI Plate 22 |  |  |  |
| Ixabepilone | SBPMDI Plate 23 |  |  |  |
| Lapatinib | SBPMDI Plate 24 |  |  |  |
| LDK378 | SBPMDI Plate 25 |  |  |  |
| Lenalidomide | SBPMDI Plate 26 |  |  |  |
| Letrozole | SBPMDI Plate 27 |  |  |  |
| Linsitinib | SBPMDI Plate 28 |  |  |  |
| Lomustine | SBPMDI Plate 29 |  |  |  |
| LY2157299 | SBPMDI Plate 30 |  |  |  |
| Mechlorethamine | SBPMDI Plate 31 |  |  |  |
| Megestrol | SBPMDI Plate 32 |  |  |  |
| MEK162 | SBPMDI Plate 33 |  |  |  |
| Melphalan | SBPMDI Plate 34 |  |  |  |
| Mercaptopurine | SBPMDI Plate 35 |  |  |  |
| Methotrexate | SBPMDI Plate 36 |  |  |  |
| Mitomycin |  |  |  |  |
| Mitotane |  |  |  |  |
| Mitoxantrone |  |  |  |  |
| MLN2480 |  |  |  |  |
| MLN4924 |  |  |  |  |
| MLN9708 |  |  |  |  |
| Navitoclax |  |  |  |  |
| Nelarabine |  |  |  |  |
| Nilotinib |  |  |  |  |
| OSI27 |  |  |  |  |
| Oxaliplatin |  |  |  |  |
| Paclitaxel |  |  |  |  |
| Palbociclib |  |  |  |  |
| Pazopanib |  |  |  |  |
| PD325901 |  |  |  |  |
| Pemetrexed |  |  |  |  |
| Pipobroman |  |  |  |  |
| Plicamycin |  |  |  |  |
| Pralatrexate |  |  |  |  |
| Quinacrine |  |  |  |  |
| Quizartinib |  |  |  |  |
| Raloxifene |  |  |  |  |
| Romidepsin |  |  |  |  |
| Sabutoclax |  |  |  |  |
| Sirolimus |  |  |  |  |
| Sorafenib |  |  |  |  |
| Streptozocin |  |  |  |  |
| Sunitinib |  |  |  |  |
| Tamoxifen |  |  |  |  |
| Temozolomide |  |  |  |  |
| Temsirolimus |  |  |  |  |
| Teniposide |  |  |  |  |
| Thioguanine |  |  |  |  |
| Thiotepa |  |  |  |  |
| Topotecan |  |  |  |  |
| Trametinib |  |  |  |  |
| Tretinoin |  |  |  |  |
| Triethylenemelamine |  |  |  |  |
| Uracil |  |  |  |  |
| Valrubicin |  |  |  |  |
| Vandetanib |  |  |  |  |
| Vemurafenib |  |  |  |  |
| Vinblastine |  |  |  |  |
| Vincristine |  |  |  |  |
| Vinorelbine |  |  |  |  |
| Vismodegib |  |  |  |  |
| Vorinostat |  |  |  |  |
| Zoledronic |  |  |  |  |

Supplemental Table 1: A list of all drugs, plates, doses, cell lines, and sites available in the HTS study.
