# Supplementary material for: Analysis of variability in high throughput screening data: applications to melanoma cell lines and drug responses
Source: Oncotarget. 2017 Feb 15;8(17):27786–99. doi: 10.18632/oncotarget.15347 (PMC5438608; doi:10.18632/oncotarget.15347)
Supplement: Supplementary file 3 [file oncotarget-08-27786-s003.docx]

**Supplemental Table 2:** Significance of Each Covariate

| Covariate | Estimate | StErr | Tstat | Pval | Significance |
| --- | --- | --- | --- | --- | --- |
| Intercept | 75.12463 | 1.861022 | 40.367 | < 2e-16 | *** |
| MeWo | 13.457613 | 1.591108 | 8.458 | < 2e-16 | *** |
| SKMEL2 | -11.121113 | 1.591108 | -6.99 | 2.83E-12 | *** |
| UACC0257 | 3.986111 | 1.591108 | 2.505 | 0.012243 | * |
| SBP | -6.033357 | 1.591108 | -3.792 | 0.00015 | *** |
| Ldose:-1.397940009 | -3.29065 | 1.656078 | -1.987 | 0.046931 | * |
| Ldose:-1 | -5.043579 | 1.656078 | -3.045 | 0.002325 | ** |
| Ldose:-0.698970004 | -6.169655 | 0.562542 | -10.967 | < 2e-16 | *** |
| Ldose:-0.397940009 | -9.698519 | 1.656078 | -5.856 | 4.79E-09 | *** |
| Ldose:0 | -11.545576 | 1.656078 | -6.972 | 3.21E-12 | *** |
| Ldose:0.301029996 | -13.397892 | 0.562542 | -23.817 | < 2e-16 | *** |
| Ldose:0.602059991 | -17.203974 | 1.656078 | -10.388 | < 2e-16 | *** |
| Ldose:1 | -22.468612 | 1.656078 | -13.567 | < 2e-16 | *** |
| Thioguanine | 11.673965 | 2.054111 | 5.683 | 1.34E-08 | *** |
| Irinotecan | 20.892411 | 2.054111 | 10.171 | < 2e-16 | *** |
| Romidepsin | -27.781036 | 2.054111 | -13.525 | < 2e-16 | *** |
| Paclitaxel | -20.700191 | 2.054111 | -10.077 | < 2e-16 | *** |
| Alisertib | 13.84424 | 2.054111 | 6.74 | 1.62E-11 | *** |
| Vorinostat | 10.164373 | 2.054111 | 4.948 | 7.53E-07 | *** |
| Busulfan | 37.333099 | 2.054111 | 18.175 | < 2e-16 | *** |
| Mechlorethamine | 33.553269 | 2.054111 | 16.335 | < 2e-16 | *** |
| Teniposide | -4.941464 | 2.054111 | -2.406 | 0.016151 | * |
| Vinorelbine | 4.33192 | 2.054111 | 2.109 | 0.034963 | * |
| Cabozantinib | 28.320453 | 2.054111 | 13.787 | < 2e-16 | *** |
| Dacarbazine | 33.961259 | 2.054111 | 16.533 | < 2e-16 | *** |
| Clofarabine | -4.94547 | 2.054111 | -2.408 | 0.016065 | * |
| Cisplatin | 36.206087 | 2.054111 | 17.626 | < 2e-16 | *** |
| Floxuridine | 6.41117 | 2.054111 | 3.121 | 0.001804 | ** |
| Lomustine | 35.527643 | 2.054111 | 17.296 | < 2e-16 | *** |
| Melphalan | 32.417813 | 2.054111 | 15.782 | < 2e-16 | *** |
| BGJ398 | 33.518964 | 2.054111 | 16.318 | < 2e-16 | *** |
| Navitoclax | 28.612003 | 2.054111 | 13.929 | < 2e-16 | *** |
| Azacitidine | 30.604597 | 2.054111 | 14.899 | < 2e-16 | *** |
| Capecitabine | 38.185406 | 2.054111 | 18.59 | < 2e-16 | *** |
| Megestrol | 39.918631 | 2.054111 | 19.434 | < 2e-16 | *** |
| Cytarabine | 16.006078 | 2.054111 | 7.792 | 6.83E-15 | *** |
| Gemcitabine | -10.164972 | 2.054111 | -4.949 | 7.52E-07 | *** |
| Vinblastine | 8.161225 | 2.054111 | 3.973 | 7.11E-05 | *** |
| MLN9708 | -14.13435 | 2.054111 | -6.881 | 6.08E-12 | *** |
| ABT737 | 29.780285 | 2.054111 | 14.498 | < 2e-16 | *** |
| Streptozocin | 36.84304 | 2.054111 | 17.936 | < 2e-16 | *** |
| Crizotinib | 32.335272 | 2.054111 | 15.742 | < 2e-16 | *** |
| Sunitinib | 37.345264 | 2.054111 | 18.181 | < 2e-16 | *** |
| Dexrazoxane | 39.666992 | 2.054111 | 19.311 | < 2e-16 | *** |
| Mitomycin | 20.193791 | 2.054111 | 9.831 | < 2e-16 | *** |
| Carfilzomib | -41.547265 | 2.054111 | -20.226 | < 2e-16 | *** |
| OSI27 | 20.131798 | 2.054111 | 9.801 | < 2e-16 | *** |
| Bioymifi | 36.475576 | 2.054111 | 17.757 | < 2e-16 | *** |
| Nelarabine | 36.67642 | 2.054111 | 17.855 | < 2e-16 | *** |
| Raloxifene | 37.056167 | 2.054111 | 18.04 | < 2e-16 | *** |
| Quinacrine | 35.345359 | 2.054111 | 17.207 | < 2e-16 | *** |
| Lenalidomide | 39.588564 | 2.054111 | 19.273 | < 2e-16 | *** |
| Fludarabine | 39.825365 | 2.054111 | 19.388 | < 2e-16 | *** |
| Nilotinib | 40.184848 | 2.054111 | 19.563 | < 2e-16 | *** |
| Linsitinib | 30.768399 | 2.054111 | 14.979 | < 2e-16 | *** |
| Aphrocallistin | 2.745456 | 2.054111 | 1.337 | 0.181376 |  |
| Mitotane | 36.064431 | 2.054111 | 17.557 | < 2e-16 | *** |
| Etoposide | 19.637318 | 2.054111 | 9.56 | < 2e-16 | *** |
| Vandetanib | 36.793782 | 2.054111 | 17.912 | < 2e-16 | *** |
| Carboplatin | 39.037476 | 2.054111 | 19.005 | < 2e-16 | *** |
| Gefitinib | 37.971129 | 2.054111 | 18.485 | < 2e-16 | *** |
| Vincristine | -11.5562 | 2.054111 | -5.626 | 1.86E-08 | *** |
| Trametinib | -25.791404 | 2.054111 | -12.556 | < 2e-16 | *** |
| MLN4924 | 4.718982 | 2.054111 | 2.297 | 0.021608 | * |
| Bortezomib | -58.838398 | 2.054111 | -28.644 | < 2e-16 | *** |
| Fluorouracil | 38.726023 | 2.054111 | 18.853 | < 2e-16 | *** |
| Lapatinib | 37.990367 | 2.054111 | 18.495 | < 2e-16 | *** |
| Mitoxantrone | 0.991545 | 2.054111 | 0.483 | 0.629304 |  |
| Imatinib | 39.975577 | 2.054111 | 19.461 | < 2e-16 | *** |
| Imiquimod | 40.324245 | 2.054111 | 19.631 | < 2e-16 | *** |
| Dacomitinib | 35.998179 | 2.054111 | 17.525 | < 2e-16 | *** |
| PD325901 | -27.320246 | 2.054111 | -13.3 | < 2e-16 | *** |
| Vismodegib | 37.384935 | 2.054111 | 18.2 | < 2e-16 | *** |
| Temozolomide | 40.939851 | 2.054111 | 19.931 | < 2e-16 | *** |
| Mercaptopurine | 19.05959 | 2.054111 | 9.279 | < 2e-16 | *** |
| Dasatinib | 22.982502 | 2.054111 | 11.189 | < 2e-16 | *** |
| Daunorubicin | -17.086396 | 2.054111 | -8.318 | < 2e-16 | *** |
| Sirolimus | 25.825896 | 2.054111 | 12.573 | < 2e-16 | *** |
| INK128 | -15.950895 | 2.054111 | -7.765 | 8.44E-15 | *** |
| Quizartinib | 36.711413 | 2.054111 | 17.872 | < 2e-16 | *** |
| Sorafenib | 33.202064 | 2.054111 | 16.164 | < 2e-16 | *** |
| Carmustine | 38.251379 | 2.054111 | 18.622 | < 2e-16 | *** |
| Uracil | 37.377948 | 2.054111 | 18.197 | < 2e-16 | *** |
| Ixabepilone | -7.683557 | 2.054111 | -3.741 | 0.000184 | *** |
| Valrubicin | 18.457582 | 2.054111 | 8.986 | < 2e-16 | *** |
| Triethylenemelamine | 37.275447 | 2.054111 | 18.147 | < 2e-16 | *** |
| Palbociclib | 26.213575 | 2.054111 | 12.762 | < 2e-16 | *** |
| Afatinib | 19.916855 | 2.054111 | 9.696 | < 2e-16 | *** |
| Doxorubicin | -2.972556 | 2.054111 | -1.447 | 0.147874 |  |
| Exemestane | 38.38916 | 2.054111 | 18.689 | < 2e-16 | *** |
| Tretinoin | 37.437 | 2.054111 | 18.225 | < 2e-16 | *** |
| Fulvestrant | 39.94196 | 2.054111 | 19.445 | < 2e-16 | *** |
| Docetaxel | -18.46839 | 2.054111 | -8.991 | < 2e-16 | *** |
| Everolimus | 22.931169 | 2.054111 | 11.164 | < 2e-16 | *** |
| MLN2480 | 30.66758 | 2.054111 | 14.93 | < 2e-16 | *** |
| LY2157299 | 37.567695 | 2.054111 | 18.289 | < 2e-16 | *** |
| Allopurinol | 37.651551 | 2.054111 | 18.33 | < 2e-16 | *** |
| Pipobroman | 39.946834 | 2.054111 | 19.447 | < 2e-16 | *** |
| Letrozole | 39.8326 | 2.054111 | 19.392 | < 2e-16 | *** |
| Thiotepa | 38.750271 | 2.054111 | 18.865 | < 2e-16 | *** |
| Plicamycin | -16.986882 | 2.054111 | -8.27 | < 2e-16 | *** |
| Erlotinib | 31.252315 | 2.054111 | 15.215 | < 2e-16 | *** |
| MEK162 | -13.881054 | 2.054111 | -6.758 | 1.43E-11 | *** |
| Baricitinib | 38.593795 | 2.054111 | 18.789 | < 2e-16 | *** |
| Arsenic | 36.754654 | 2.054111 | 17.893 | < 2e-16 | *** |
| Celecoxib | 37.816213 | 2.054111 | 18.41 | < 2e-16 | *** |
| Bendamustine | 37.643125 | 2.054111 | 18.326 | < 2e-16 | *** |
| Chlorambucil | 37.606517 | 2.054111 | 18.308 | < 2e-16 | *** |
| Zoledronic | 35.827106 | 2.054111 | 17.442 | < 2e-16 | *** |
| Actinomycin | -42.753826 | 2.054111 | -20.814 | < 2e-16 | *** |
| Temsirolimus | 20.098025 | 2.054111 | 9.784 | < 2e-16 | *** |
| Foretinib | 17.85076 | 2.054111 | 8.69 | < 2e-16 | *** |
| Decitabine | 32.334116 | 2.054111 | 15.741 | < 2e-16 | *** |
| Methotrexate | 35.885363 | 2.054111 | 17.47 | < 2e-16 | *** |
| Axitinib | 36.238548 | 2.054111 | 17.642 | < 2e-16 | *** |
| Oxaliplatin | 36.89309 | 2.054111 | 17.961 | < 2e-16 | *** |
| Cabazitaxel | -13.104848 | 2.054111 | -6.38 | 1.80E-10 | *** |
| Amifostine | 37.968917 | 2.054111 | 18.484 | < 2e-16 | *** |
| Flutamide | 35.56271 | 2.054111 | 17.313 | < 2e-16 | *** |
| LDK378 | 21.889006 | 2.054111 | 10.656 | < 2e-16 | *** |
| Pralatrexate | -30.733792 | 2.054111 | -14.962 | < 2e-16 | *** |
| Topotecan | -7.533448 | 2.054111 | -3.667 | 0.000245 | *** |
| Pemetrexed | 35.936064 | 2.054111 | 17.495 | < 2e-16 | *** |
| Bleomycin | 10.465324 | 2.054111 | 5.095 | 3.52E-07 | *** |
| Axitinib.1 | 37.158681 | 2.054111 | 18.09 | < 2e-16 | *** |
| Ibrutinib | 36.572295 | 2.054111 | 17.804 | < 2e-16 | *** |
| Tamoxifen | 32.96471 | 2.054111 | 16.048 | < 2e-16 | *** |
| Vemurafenib | 17.27205 | 2.054111 | 8.409 | < 2e-16 | *** |
| Pazopanib | 27.875965 | 2.054111 | 13.571 | < 2e-16 | *** |
| Abiraterone | 35.658004 | 2.054111 | 17.359 | < 2e-16 | *** |
| Bosutinib | 25.794763 | 2.054111 | 12.558 | < 2e-16 | *** |
| Sabutoclax | 15.172258 | 2.054111 | 7.386 | 1.56E-13 | *** |
| Plate: -35 | 2.5423 | 1.591108 | 1.598 | 0.110096 |  |
| Plate: -34 | 3.498033 | 1.591108 | 2.198 | 0.027923 | * |
| Plate: -33 | 6.157534 | 2.250166 | 2.736 | 0.006214 | ** |
| Plate: -32 | 5.129357 | 2.250166 | 2.28 | 0.022643 | * |
| Plate: -31 | 4.968405 | 2.250166 | 2.208 | 0.027252 | * |
| Plate: -30 | 3.561967 | 2.250166 | 1.583 | 0.113438 |  |
| Plate: -29 | 4.51169 | 2.250166 | 2.005 | 0.044968 | * |
| Plate: -28 | 2.864528 | 2.250166 | 1.273 | 0.203019 |  |
| Plate: -27 | -34.360242 | 2.250166 | -15.27 | < 2e-16 | *** |
| Plate: -26 | 3.260706 | 2.250166 | 1.449 | 0.147323 |  |
| Plate: -25 | 5.880522 | 2.250166 | 2.613 | 0.008971 | ** |
| Plate: -24 | 19.99294 | 2.75588 | 7.255 | 4.14E-13 | *** |
| Plate: -23 | 7.979931 | 2.75588 | 2.896 | 0.003788 | ** |
| Plate: -22 | 5.633082 | 2.75588 | 2.044 | 0.040961 | * |
| Plate: -21 | 0.119387 | 2.250166 | 0.053 | 0.957687 |  |
| Plate: -20 | 4.728863 | 2.250166 | 2.102 | 0.035601 | * |
| Plate: -19 | -2.269292 | 2.250166 | -1.008 | 0.313224 |  |
| Plate: -18 | -0.562341 | 2.250166 | -0.25 | 0.802658 |  |
| Plate: -17 | 3.091143 | 2.250166 | 1.374 | 0.169534 |  |
| Plate: -16 | 2.655494 | 2.250166 | 1.18 | 0.237958 |  |
| Plate: -15 | 5.877087 | 2.75588 | 2.133 | 0.03297 | * |
| Plate: -14 | 9.999689 | 2.75588 | 3.628 | 0.000286 | *** |
| Plate: -13 | 9.97251 | 2.75588 | 3.619 | 0.000297 | *** |
| Plate: -12 | 9.915747 | 2.250166 | 4.407 | 1.05E-05 | *** |
| Plate: -11 | 8.542084 | 2.250166 | 3.796 | 0.000147 | *** |
| Plate: -10 | 0.4293 | 2.250166 | 0.191 | 0.848695 |  |
| Plate: -9 | 9.384956 | 2.250166 | 4.171 | 3.05E-05 | *** |
| Plate: -8 | 9.575084 | 2.250166 | 4.255 | 2.10E-05 | *** |
| Plate: -7 | 6.545418 | 2.250166 | 2.909 | 0.003631 | ** |
| Plate: -6 | 10.858216 | 2.75588 | 3.94 | 8.17E-05 | *** |
| Plate: -5 | 12.442361 | 2.75588 | 4.515 | 6.36E-06 | *** |
| Plate: -4 | 12.759179 | 2.75588 | 4.63 | 3.68E-06 | *** |
| Plate: -3 | 12.650668 | 2.250166 | 5.622 | 1.91E-08 | *** |
| Plate: -2 | 13.517878 | 2.250166 | 6.008 | 1.91E-09 | *** |
| Plate: -1 | 14.634887 | 2.250166 | 6.504 | 7.97E-11 | *** |
| Plate: 1 | 2.088091 | 2.250166 | 0.928 | 0.353431 |  |
| Plate: 2 | -1.369853 | 2.250166 | -0.609 | 0.542677 |  |
| Plate: 3 | -6.020652 | 2.250166 | -2.676 | 0.007463 | ** |
| Plate: 4 | 3.151328 | 2.250166 | 1.4 | 0.16138 |  |
| Plate: 5 | 3.175642 | 2.250166 | 1.411 | 0.158171 |  |
| Plate: 6 | 2.382005 | 2.250166 | 1.059 | 0.289796 |  |
| Plate: 7 | 1.098032 | 1.591108 | 0.69 | 0.490134 |  |
| Plate: 8 | -0.005717 | 1.591108 | -0.004 | 0.997133 |  |
| Plate: 9 | NA | NA | NA | NA |  |
| Plate: 10 | -2.135263 | 2.250166 | -0.949 | 0.342662 |  |
| Plate: 11 | -2.254601 | 2.250166 | -1.002 | 0.316367 |  |
| Plate: 12 | -2.41568 | 2.250166 | -1.074 | 0.283032 |  |
| Plate: 13 | 1.074383 | 2.250166 | 0.477 | 0.633033 |  |
| Plate: 14 | -0.116427 | 2.250166 | -0.052 | 0.958735 |  |
| Plate: 15 | -0.334813 | 2.250166 | -0.149 | 0.881717 |  |
| Plate: 16 | -5.626775 | 1.591108 | -3.536 | 0.000406 | *** |
| Plate: 17 | 0.366361 | 1.591108 | 0.23 | 0.817895 |  |
| Plate: 18 | NA | NA | NA | NA |  |
| Plate: 19 | -0.728745 | 2.250166 | -0.324 | 0.746044 |  |
| Plate: 20 | -1.48549 | 2.250166 | -0.66 | 0.509151 |  |
| Plate: 21 | 3.705302 | 2.250166 | 1.647 | 0.099636 | . |
| Plate: 22 | 0.835384 | 2.250166 | 0.371 | 0.710451 |  |
| Plate: 23 | 0.919319 | 2.250166 | 0.409 | 0.682869 |  |
| Plate: 24 | 1.136611 | 2.250166 | 0.505 | 0.613477 |  |
| Plate: 25 | 22.874691 | 1.591108 | 14.377 | < 2e-16 | *** |
| Plate: 26 | -0.095872 | 1.591108 | -0.06 | 0.951953 |  |
| Plate: 27 | NA | NA | NA | NA |  |
| Plate: 28 | -0.525181 | 1.591108 | -0.33 | 0.741348 |  |
| Plate: 29 | -0.413643 | 1.591108 | -0.26 | 0.794888 |  |
| Plate: 30 | NA | NA | NA | NA |  |
| Plate: 31 | 0.969505 | 1.591108 | 0.609 | 0.542313 |  |
| Plate: 32 | 0.557125 | 1.591108 | 0.35 | 0.72623 |  |
| Plate: 33 | NA | NA | NA | NA |  |
| Plate: 34 | -1.622432 | 1.591108 | -1.02 | 0.307886 |  |
| Plate: 35 | 0.043811 | 1.591108 | 0.028 | 0.978033 |  |
| Plate: 36 | NA | NA | NA | NA |  |

Supplemental Table 2: ANOVA analysis for site, dose, cell line, and plate effects. Signif. codes: 0 ‘***’ 0.001 ‘**’ 0.01 ‘*’ 0.05 ‘.’ 0.1 ‘ ’ 1
