# Supplementary material for: Analysis of variability in high throughput screening data: applications to melanoma cell lines and drug responses
Source: Oncotarget. 2017 Feb 15;8(17):27786–99. doi: 10.18632/oncotarget.15347 (PMC5438608; doi:10.18632/oncotarget.15347)
Supplement: Supplementary file 4 [file oncotarget-08-27786-s004.docx]

**Supplemental Table 4:** Factor Significance in Model with Drug:Site Interaction

| Covariate | Estimate | StErr | Tstat | Pval | Estimate_SiteInter | StErr_SiteInter | Tstat_SiteInter | Pval_SiteInter |
| --- | --- | --- | --- | --- | --- | --- | --- | --- |
| Intercept | 86.40403302 | 2.246321685 | 38.46467476 | 9.82600141798009e-315 |  |  |  |  |
| SBP | -28.59216394 | 3.146474765 | -9.087046955 | 1.09E-19 |  |  |  |  |
| MeWo | 13.45761327 | 1.51638561 | 8.874796212 | 7.45E-19 |  |  |  |  |
| SKMEL2 | -11.12111256 | 1.51638561 | -7.333960761 | 2.30E-13 |  |  |  |  |
| UACC0257 | 3.986110915 | 1.51638561 | 2.628692128 | 0.00857651 |  |  |  |  |
| Thioguanine | -0.519120002 | 2.768528681 | -0.18750754 | 0.851264198 | 24.38617093 | 3.915290809 | 6.228444353 | 4.78E-10 |
| Irinotecan | 14.2890756 | 2.768528681 | 5.16125251 | 2.47E-07 | 13.20667162 | 3.915290809 | 3.373101071 | 0.000744368 |
| Romidepsin | -8.812209092 | 2.768528681 | -3.182993607 | 0.001459351 | -37.93765295 | 3.915290809 | -9.689613058 | 3.64E-22 |
| Paclitaxel | -30.19169669 | 2.768528681 | -10.90532198 | 1.25E-27 | 18.9830115 | 3.915290809 | 4.848429512 | 1.25E-06 |
| Alisertib | 11.35065237 | 2.768528681 | 4.099886141 | 4.15E-05 | 4.987174483 | 3.915290809 | 1.273768598 | 0.202757 |
| Vorinostat | -8.039127312 | 2.768528681 | -2.90375439 | 0.003690323 | 36.40700046 | 3.915290809 | 9.298670837 | 1.53E-20 |
| Busulfan | 16.56011221 | 2.768528681 | 5.981557034 | 2.24E-09 | 41.54597391 | 3.915290809 | 10.61121024 | 3.00E-26 |
| Mechlorethamine | 18.50433043 | 2.768528681 | 6.68381388 | 2.38E-11 | 30.09787791 | 3.915290809 | 7.687264977 | 1.56E-14 |
| Teniposide | -9.972879491 | 2.768528681 | -3.602230874 | 0.000316095 | 10.06283042 | 3.915290809 | 2.570136143 | 0.010171457 |
| Vinorelbine | -4.797502655 | 2.768528681 | -1.732870852 | 0.083130667 | 18.25884617 | 3.915290809 | 4.663471263 | 3.12E-06 |
| Cabozantinib | 10.6143663 | 2.768528681 | 3.833937635 | 0.00012641 | 35.41217351 | 3.915290809 | 9.04458321 | 1.61E-19 |
| Dacarbazine | 20.10250413 | 2.768528681 | 7.261078517 | 3.95E-13 | 27.71750976 | 3.915290809 | 7.079297839 | 1.49E-12 |
| Clofarabine | -6.469547359 | 2.768528681 | -2.336817893 | 0.019456324 | 3.048153841 | 3.915290809 | 0.778525527 | 0.436266458 |
| Cisplatin | 18.15044646 | 2.768528681 | 6.555990039 | 5.63E-11 | 36.1112804 | 3.915290809 | 9.22314131 | 3.10E-20 |
| Floxuridine | -5.095138509 | 2.768528681 | -1.840377723 | 0.065724367 | 23.01261721 | 3.915290809 | 5.877626551 | 4.21E-09 |
| Lomustine | 17.85110002 | 2.768528681 | 6.447865303 | 1.15E-10 | 35.35308609 | 3.915290809 | 9.029491759 | 1.84E-19 |
| Melphalan | 16.33104836 | 2.768528681 | 5.898818556 | 3.71E-09 | 32.17352942 | 3.915290809 | 8.217404784 | 2.18E-16 |
| BGJ398 | 16.12615281 | 2.768528681 | 5.824809734 | 5.79E-09 | 34.78562311 | 3.915290809 | 8.884556682 | 6.83E-19 |
| Navitoclax | 16.89348772 | 2.768528681 | 6.101973166 | 1.06E-09 | 23.4370308 | 3.915290809 | 5.986025546 | 2.18E-09 |
| Azacitidine | 17.00179109 | 2.768528681 | 6.141092633 | 8.32E-10 | 27.20561262 | 3.915290809 | 6.948554768 | 3.78E-12 |
| Capecitabine | 22.33062303 | 2.768528681 | 8.065881053 | 7.59E-16 | 31.70956678 | 3.915290809 | 8.098904611 | 5.79E-16 |
| Megestrol | 21.99529416 | 2.768528681 | 7.944759361 | 2.03E-15 | 35.84667344 | 3.915290809 | 9.155558345 | 5.80E-20 |
| Cytarabine | 11.54025583 | 2.768528681 | 4.168371419 | 3.08E-05 | 8.931645098 | 3.915290809 | 2.281221379 | 0.022543524 |
| Gemcitabine | -2.471484685 | 2.768528681 | -0.89270691 | 0.372022539 | -15.38697457 | 3.915290809 | -3.929969784 | 8.52E-05 |
| Vinblastine | 21.45294437 | 2.768528681 | 7.748861163 | 9.61E-15 | -26.58343974 | 3.915290809 | -6.789646298 | 1.15E-11 |
| MLN9708 | -14.48318391 | 2.768528681 | -5.231364951 | 1.70E-07 | 0.697668166 | 3.915290809 | 0.178190638 | 0.858574672 |
| ABT737 | 20.7373932 | 2.768528681 | 7.490402155 | 7.09E-14 | 18.08578365 | 3.915290809 | 4.619269561 | 3.87E-06 |
| Streptozocin | 22.70601881 | 2.768528681 | 8.201475016 | 2.48E-16 | 28.2740423 | 3.915290809 | 7.221441186 | 5.29E-13 |
| Crizotinib | 20.445278 | 2.768528681 | 7.384889361 | 1.57E-13 | 23.77998774 | 3.915290809 | 6.07361979 | 1.27E-09 |
| Sunitinib | 19.68285102 | 2.768528681 | 7.109498685 | 1.20E-12 | 35.32482676 | 3.915290809 | 9.022274075 | 1.97E-19 |
| Dexrazoxane | 22.95177238 | 2.768528681 | 8.290241867 | 1.19E-16 | 33.43043966 | 3.915290809 | 8.538430806 | 1.43E-17 |
| MitomycinC | 11.69711334 | 2.768528681 | 4.225028774 | 2.40E-05 | 16.99335611 | 3.915290809 | 4.340253876 | 1.43E-05 |
| Carfilzomib | -49.39229557 | 2.768528681 | -17.8406299 | 9.17E-71 | 15.69006039 | 3.915290809 | 4.007380589 | 6.16E-05 |
| OSI27 | 11.30856297 | 2.768528681 | 4.084683336 | 4.43E-05 | 17.64647036 | 3.915290809 | 4.50706505 | 6.60E-06 |
| Bioymifi | 22.66716874 | 2.768528681 | 8.187442266 | 2.79E-16 | 27.61681367 | 3.915290809 | 7.053579164 | 1.79E-12 |
| Nelarabine | 22.60615667 | 2.768528681 | 8.165404545 | 3.35E-16 | 28.14052758 | 3.915290809 | 7.187340344 | 6.79E-13 |
| Raloxifene | 20.19294737 | 2.768528681 | 7.293746858 | 3.10E-13 | 33.72643967 | 3.915290809 | 8.614031835 | 7.45E-18 |
| Quinacrine | 18.10527356 | 2.768528681 | 6.539673468 | 6.28E-11 | 34.48017181 | 3.915290809 | 8.806541709 | 1.37E-18 |
| Lenalidomide | 22.620031 | 2.768528681 | 8.170415989 | 3.21E-16 | 33.9370653 | 3.915290809 | 8.667827488 | 4.66E-18 |
| Fludarabine | 26.46044735 | 2.768528681 | 9.557584696 | 1.31E-21 | 26.72983598 | 3.915290809 | 6.827037196 | 8.86E-12 |
| Nilotinib | 21.58722441 | 2.768528681 | 7.797363471 | 6.56E-15 | 37.19524782 | 3.915290809 | 9.499996203 | 2.28E-21 |
| Linsitinib | 17.41523191 | 2.768528681 | 6.29042857 | 3.22E-10 | 26.70633475 | 3.915290809 | 6.821034774 | 9.24E-12 |
| Aphrocallistin | -15.0020982 | 2.768528681 | -5.418798188 | 6.05E-08 | 35.49510746 | 3.915290809 | 9.065765277 | 1.32E-19 |
| Mitotane | 21.89258211 | 2.768528681 | 7.907659496 | 2.73E-15 | 28.34369752 | 3.915290809 | 7.239231746 | 4.64E-13 |
| Etoposide | 12.01208564 | 2.768528681 | 4.338797615 | 1.44E-05 | 15.25046529 | 3.915290809 | 3.895104102 | 9.84E-05 |
| Vandetanib | 20.67493245 | 2.768528681 | 7.467841166 | 8.41E-14 | 32.23769995 | 3.915290809 | 8.233794507 | 1.90E-16 |
| Carboplatin | 22.00619095 | 2.768528681 | 7.948695313 | 1.96E-15 | 34.06256923 | 3.915290809 | 8.699882307 | 3.52E-18 |
| Gefitinib | 21.98725606 | 2.768528681 | 7.941855978 | 2.07E-15 | 31.9677458 | 3.915290809 | 8.164845821 | 3.37E-16 |
| Vincristine | -15.08153504 | 2.768528681 | -5.447490989 | 5.16E-08 | 7.050670228 | 3.915290809 | 1.800803714 | 0.071745586 |
| Trametinib | -36.20304692 | 2.768528681 | -13.07663784 | 5.97E-39 | 20.82328673 | 3.915290809 | 5.318452128 | 1.06E-07 |
| MLN4924 | -6.392572444 | 2.768528681 | -2.309014347 | 0.020950707 | 22.22310856 | 3.915290809 | 5.675979038 | 1.39E-08 |
| Bortezomib | -71.1082199 | 2.768528681 | -25.68448013 | 1.15E-143 | 24.53964305 | 3.915290809 | 6.267642494 | 3.72E-10 |
| Fluorouracil | 24.6498826 | 2.768528681 | 8.903603841 | 5.76E-19 | 28.15228129 | 3.915290809 | 7.190342344 | 6.64E-13 |
| Lapatinib | 22.70188736 | 2.768528681 | 8.199982726 | 2.52E-16 | 30.57695894 | 3.915290809 | 7.809626521 | 5.95E-15 |
| Mitoxantrone | -4.51964582 | 2.768528681 | -1.632508217 | 0.102584766 | 11.02238101 | 3.915290809 | 2.815213874 | 0.0048782 |
| Imatinib | 23.85778827 | 2.768528681 | 8.617497241 | 7.23E-18 | 32.23557654 | 3.915290809 | 8.23325217 | 1.91E-16 |
| Imiquimod | 23.46618449 | 2.768528681 | 8.476048901 | 2.45E-17 | 33.716122 | 3.915290809 | 8.61139661 | 7.63E-18 |
| Dacomitinib | 20.61297604 | 2.768528681 | 7.445462344 | 9.97E-14 | 30.77040544 | 3.915290809 | 7.859034474 | 4.02E-15 |
| PD325901 | -33.3473221 | 2.768528681 | -12.04514237 | 2.53E-33 | 12.05415266 | 3.915290809 | 3.078737506 | 0.002080996 |
| Vismodegib | 23.40144593 | 2.768528681 | 8.45266516 | 3.00E-17 | 27.96697722 | 3.915290809 | 7.143014042 | 9.37E-13 |
| Temozolomide | 25.41061892 | 2.768528681 | 9.178383844 | 4.70E-20 | 31.05846349 | 3.915290809 | 7.932607055 | 2.23E-15 |
| Mercaptopurine | 4.754078934 | 2.768528681 | 1.717186088 | 0.08595727 | 28.61102199 | 3.915290809 | 7.307508788 | 2.80E-13 |
| Dasatinib | 11.55776063 | 2.768528681 | 4.174694201 | 2.99E-05 | 22.84948289 | 3.915290809 | 5.835960598 | 5.41E-09 |
| Daunorubicin | -24.78171324 | 2.768528681 | -8.951221423 | 3.75E-19 | 15.39063361 | 3.915290809 | 3.930904335 | 8.48E-05 |
| Sirolimus | 17.9427865 | 2.768528681 | 6.480982706 | 9.28E-11 | 15.76621813 | 3.915290809 | 4.026831951 | 5.67E-05 |
| INK128 | -21.08176035 | 2.768528681 | -7.614788496 | 2.73E-14 | 10.26173072 | 3.915290809 | 2.620937045 | 0.008774026 |
| Quizartinib | 24.17078163 | 2.768528681 | 8.730551284 | 2.69E-18 | 25.08126281 | 3.915290809 | 6.405976986 | 1.52E-10 |
| Sorafenib | 22.21230197 | 2.768528681 | 8.023143167 | 1.07E-15 | 21.97952488 | 3.915290809 | 5.613765607 | 2.00E-08 |
| Carmustine | 20.50466876 | 2.768528681 | 7.406341462 | 1.34E-13 | 35.49342013 | 3.915290809 | 9.065334318 | 1.33E-19 |
| Uracil | 19.25059059 | 2.768528681 | 6.953365056 | 3.65E-12 | 36.25471404 | 3.915290809 | 9.259775535 | 2.20E-20 |
| Ixabepilone | -13.44927236 | 2.768528681 | -4.857913322 | 1.19E-06 | 11.53142977 | 3.915290809 | 2.945229443 | 0.003230055 |
| Valrubicin | 12.50301269 | 2.768528681 | 4.516121787 | 6.33E-06 | 11.90913823 | 3.915290809 | 3.041699535 | 0.002354846 |
| Triethylenemelamine | 19.73014753 | 2.768528681 | 7.126582312 | 1.06E-12 | 35.09059969 | 3.915290809 | 8.962450406 | 3.39E-19 |
| Palbociclib | 15.28501811 | 2.768528681 | 5.52098962 | 3.40E-08 | 21.85711337 | 3.915290809 | 5.582500621 | 2.39E-08 |
| Afatinib | 8.942734722 | 2.768528681 | 3.230139815 | 0.001238858 | 21.94824028 | 3.915290809 | 5.605775242 | 2.09E-08 |
| Doxorubicin | -2.676699438 | 2.768528681 | -0.966831031 | 0.333637641 | -0.591712599 | 3.915290809 | -0.151128646 | 0.879875426 |
| Exemestane | 21.49424486 | 2.768528681 | 7.763779008 | 8.55E-15 | 33.78983014 | 3.915290809 | 8.630222324 | 6.47E-18 |
| Tretinoin | 20.57440974 | 2.768528681 | 7.431532091 | 1.11E-13 | 33.72518008 | 3.915290809 | 8.613710124 | 7.47E-18 |
| Fulvestrant | 23.62224997 | 2.768528681 | 8.532420173 | 1.51E-17 | 32.63941948 | 3.915290809 | 8.336397237 | 8.04E-17 |
| Docetaxel | -25.78430539 | 2.768528681 | -9.313360401 | 1.33E-20 | 14.63183131 | 3.915290809 | 3.737099498 | 0.00018656 |
| Everolimus | 15.9442272 | 2.768528681 | 5.759097716 | 8.55E-09 | 13.97388298 | 3.915290809 | 3.569053658 | 0.000358928 |
| MLN2480 | 15.3543967 | 2.768528681 | 5.546049353 | 2.95E-08 | 30.62636719 | 3.915290809 | 7.822245827 | 5.39E-15 |
| LY2157299 | 22.60299435 | 2.768528681 | 8.164262305 | 3.38E-16 | 29.92940195 | 3.915290809 | 7.644234722 | 2.18E-14 |
| Allopurinol | 23.7020082 | 2.768528681 | 8.561229062 | 1.18E-17 | 27.89908624 | 3.915290809 | 7.125674083 | 1.06E-12 |
| Pipobroman | 22.8721143 | 2.768528681 | 8.261469155 | 1.51E-16 | 34.14944033 | 3.915290809 | 8.722069955 | 2.89E-18 |
| Letrozole | 21.93170014 | 2.768528681 | 7.921789033 | 2.44E-15 | 35.80179986 | 3.915290809 | 9.144097235 | 6.45E-20 |
| Thiotepa | 20.3618583 | 2.768528681 | 7.354757942 | 1.97E-13 | 36.77682595 | 3.915290809 | 9.393127549 | 6.29E-21 |
| Plicamycin | -11.54910683 | 2.768528681 | -4.171568427 | 3.04E-05 | -10.87554965 | 3.915290809 | -2.777711843 | 0.005478297 |
| Erlotinib | 18.63681522 | 2.768528681 | 6.731667745 | 1.71E-11 | 25.23100052 | 3.915290809 | 6.444221324 | 1.18E-10 |
| MEK162 | -18.2559634 | 2.768528681 | -6.594103044 | 4.36E-11 | 8.749818959 | 3.915290809 | 2.234781371 | 0.025440297 |
| Baricitinib | 25.20021997 | 2.768528681 | 9.10238718 | 9.46E-20 | 26.78714947 | 3.915290809 | 6.841675569 | 8.00E-12 |
| Arsenic | 23.54850586 | 2.768528681 | 8.505783602 | 1.90E-17 | 26.41229599 | 3.915290809 | 6.745934666 | 1.55E-11 |
| Celecoxib | 24.13218156 | 2.768528681 | 8.716608836 | 3.04E-18 | 27.36806195 | 3.915290809 | 6.99004577 | 2.82E-12 |
| Bendamustine | 22.1845509 | 2.768528681 | 8.013119406 | 1.17E-15 | 30.91714725 | 3.915290809 | 7.896513634 | 2.98E-15 |
| Chlorambucil | 23.75064065 | 2.768528681 | 8.578795231 | 1.01E-17 | 27.71175195 | 3.915290809 | 7.077827242 | 1.50E-12 |
| Zoledronic | 24.37416157 | 2.768528681 | 8.804012665 | 1.40E-18 | 22.90588935 | 3.915290809 | 5.85036731 | 4.96E-09 |
| Actinomycin | -46.03674936 | 2.768528681 | -16.62859759 | 9.13E-62 | 6.565847511 | 3.915290809 | 1.676975692 | 0.093559442 |
| Temsirolimus | 16.92041419 | 2.768528681 | 6.111699079 | 1.00E-09 | 6.35522192 | 3.915290809 | 1.623180047 | 0.104563233 |
| Foretinib | 3.870181176 | 2.768528681 | 1.397919842 | 0.16214925 | 27.96115771 | 3.915290809 | 7.141527687 | 9.48E-13 |
| Decitabine | 20.01156142 | 2.768528681 | 7.228229766 | 5.03E-13 | 24.64510895 | 3.915290809 | 6.294579421 | 3.13E-10 |
| Methotrexate | 24.17608149 | 2.768528681 | 8.732465607 | 2.64E-18 | 23.41856388 | 3.915290809 | 5.981308931 | 2.24E-09 |
| Axitinib | 23.97965323 | 2.768528681 | 8.661515192 | 4.92E-18 | 24.51778936 | 3.915290809 | 6.262060869 | 3.86E-10 |
| Oxaliplatin | 24.70921348 | 2.768528681 | 8.925034314 | 4.75E-19 | 24.36775319 | 3.915290809 | 6.2237403 | 4.93E-10 |
| Cabazitaxel | -17.35715175 | 2.768528681 | -6.269449859 | 3.68E-10 | 8.504608231 | 3.915290809 | 2.172152376 | 0.029853317 |
| Amifostine | 25.56833619 | 2.768528681 | 9.235351746 | 2.77E-20 | 24.80116196 | 3.915290809 | 6.334436743 | 2.42E-10 |
| Flutamide | 24.1739321 | 2.768528681 | 8.731689242 | 2.66E-18 | 22.77755679 | 3.915290809 | 5.817590034 | 6.04E-09 |
| LDK378 | 18.14521931 | 2.768528681 | 6.554101979 | 5.70E-11 | 7.487574211 | 3.915290809 | 1.912392866 | 0.055836968 |
| Pralatrexate | -53.46752442 | 2.768528681 | -19.31261351 | 1.63E-82 | 45.46746516 | 3.915290809 | 11.61279389 | 4.25E-31 |
| Topotecan | -10.80254401 | 2.768528681 | -3.90190793 | 9.57E-05 | 6.538192162 | 3.915290809 | 1.66991227 | 0.094948946 |
| Pemetrexed | 21.20926136 | 2.768528681 | 7.660842204 | 1.91E-14 | 29.45360531 | 3.915290809 | 7.52271204 | 5.54E-14 |
| Bleomycin | 11.95933506 | 2.768528681 | 4.319743964 | 1.57E-05 | -2.988023022 | 3.915290809 | -0.763167583 | 0.445370491 |
| Axitinib.1 | 22.26811145 | 2.768528681 | 8.043301703 | 9.12E-16 | 29.78113855 | 3.915290809 | 7.606366935 | 2.92E-14 |
| Ibrutinib | 21.26243663 | 2.768528681 | 7.680049254 | 1.65E-14 | 30.61971707 | 3.915290809 | 7.820547329 | 5.46E-15 |
| Tamoxifen | 21.05385014 | 2.768528681 | 7.604707251 | 2.95E-14 | 23.82172024 | 3.915290809 | 6.08427864 | 1.19E-09 |
| Vemurafenib | 4.367937748 | 2.768528681 | 1.577710853 | 0.114644406 | 25.80822521 | 3.915290809 | 6.591649631 | 4.43E-11 |
| Pazopanib | 17.20598837 | 2.768528681 | 6.21484924 | 5.22E-10 | 21.33995237 | 3.915290809 | 5.450413115 | 5.07E-08 |
| Abiraterone | 24.83986937 | 2.768528681 | 8.972227572 | 3.10E-19 | 21.63626952 | 3.915290809 | 5.526095143 | 3.31E-08 |
| Bosutinib | 13.01594181 | 2.768528681 | 4.701393163 | 2.60E-06 | 25.55764152 | 3.915290809 | 6.527648333 | 6.81E-11 |
| Sabutoclax | 2.541879128 | 2.768528681 | 0.918133572 | 0.358557589 | 25.26075791 | 3.915290809 | 6.451821625 | 1.12E-10 |
| Ldose: -1.397940009 (uM) | -3.290650486 | 1.578304183 | -2.084927938 | 0.037085725 |  |  |  |  |
| Ldose: -1 (uM) | -5.043578603 | 1.578304183 | -3.195568166 | 0.001397251 |  |  |  |  |
| Ldose: -0.698970004 (uM) | -6.169655059 | 0.536123274 | -11.5079038 | 1.43E-30 |  |  |  |  |
| Ldose: -0.397940009 (uM) | -9.698519416 | 1.578304183 | -6.144898759 | 8.12E-10 |  |  |  |  |
| Ldose: 0 (uM) | -11.54557581 | 1.578304183 | -7.315177856 | 2.65E-13 |  |  |  |  |
| Ldose: 0.301029996 (uM) | -13.39789184 | 0.536123274 | -24.99031938 | 3.34E-136 |  |  |  |  |
| Ldose: 0.602059991 (uM) | -17.20397433 | 1.578304183 | -10.90029065 | 1.32E-27 |  |  |  |  |
| Ldose: 1 (uM) | -22.4686118 | 1.578304183 | -14.23591982 | 8.20E-46 |  |  |  |  |
| Plate: -35 | 2.542300278 | 1.51638561 | 1.676552627 | 0.093642205 |  |  |  |  |
| Plate: -34 | 3.4980325 | 1.51638561 | 2.306822537 | 0.021072657 |  |  |  |  |
| Plate: -33 | 6.157534063 | 2.144493095 | 2.8713238 | 0.004090918 |  |  |  |  |
| Plate: -32 | 5.129357119 | 2.144493095 | 2.391873926 | 0.016769768 |  |  |  |  |
| Plate: -31 | 4.968404619 | 2.144493095 | 2.316820059 | 0.020521386 |  |  |  |  |
| Plate: -30 | 3.561967451 | 2.144493095 | 1.660983409 | 0.096729023 |  |  |  |  |
| Plate: -29 | 4.511690229 | 2.144493095 | 2.103849268 | 0.035401324 |  |  |  |  |
| Plate: -28 | 2.864528284 | 2.144493095 | 1.335760088 | 0.181639575 |  |  |  |  |
| Plate: -27 | -34.36024154 | 2.144493095 | -16.02254706 | 1.69E-57 |  |  |  |  |
| Plate: -26 | 3.260705684 | 2.144493095 | 1.520501834 | 0.128397247 |  |  |  |  |
| Plate: -25 | 5.88052235 | 2.144493095 | 2.742150284 | 0.00610808 |  |  |  |  |
| Plate: -24 | 19.99293975 | 2.62645692 | 7.612133135 | 2.79E-14 |  |  |  |  |
| Plate: -23 | 7.979931136 | 2.62645692 | 3.038287465 | 0.002381666 |  |  |  |  |
| Plate: -22 | 5.633081969 | 2.62645692 | 2.144745618 | 0.031982573 |  |  |  |  |
| Plate: -21 | 0.119387301 | 2.144493095 | 0.055671572 | 0.955603881 |  |  |  |  |
| Plate: -20 | 4.728863412 | 2.144493095 | 2.20511944 | 0.02745457 |  |  |  |  |
| Plate: -19 | -2.269292421 | 2.144493095 | -1.058195257 | 0.289976417 |  |  |  |  |
| Plate: -18 | -0.562341179 | 2.144493095 | -0.262225689 | 0.793149559 |  |  |  |  |
| Plate: -17 | 3.09114271 | 2.144493095 | 1.441432811 | 0.149474676 |  |  |  |  |
| Plate: -16 | 2.655494099 | 2.144493095 | 1.238285218 | 0.215621676 |  |  |  |  |
| Plate: -15 | 5.877087051 | 2.62645692 | 2.237648372 | 0.02525256 |  |  |  |  |
| Plate: -14 | 9.999688718 | 2.62645692 | 3.807292114 | 0.000140824 |  |  |  |  |
| Plate: -13 | 9.972509829 | 2.62645692 | 3.796943995 | 0.000146829 |  |  |  |  |
| Plate: -12 | 9.91574655 | 2.144493095 | 4.623818362 | 3.79E-06 |  |  |  |  |
| Plate: -11 | 8.54208405 | 2.144493095 | 3.983264889 | 6.82E-05 |  |  |  |  |
| Plate: -10 | 0.429300439 | 2.144493095 | 0.200187373 | 0.841335632 |  |  |  |  |
| Plate: -9 | 9.384956382 | 2.144493095 | 4.376305246 | 1.21E-05 |  |  |  |  |
| Plate: -8 | 9.575083882 | 2.144493095 | 4.464963727 | 8.04E-06 |  |  |  |  |
| Plate: -7 | 6.545418048 | 2.144493095 | 3.052198239 | 0.002274048 |  |  |  |  |
| Plate: -6 | 10.85821572 | 2.62645692 | 4.134168598 | 3.57E-05 |  |  |  |  |
| Plate: -5 | 12.44236128 | 2.62645692 | 4.737317861 | 2.18E-06 |  |  |  |  |
| Plate: -4 | 12.75917933 | 2.62645692 | 4.857943505 | 1.19E-06 |  |  |  |  |
| Plate: -3 | 12.65066772 | 2.144493095 | 5.899141271 | 3.70E-09 |  |  |  |  |
| Plate: -2 | 13.51787772 | 2.144493095 | 6.303530542 | 2.96E-10 |  |  |  |  |
| Plate: -1 | 14.63488661 | 2.144493095 | 6.824403699 | 9.03E-12 |  |  |  |  |
| Plate: 1 | 2.08809055 | 2.144493095 | 0.973698892 | 0.330215266 |  |  |  |  |
| Plate: 2 | -1.369852595 | 2.144493095 | -0.638776874 | 0.522973809 |  |  |  |  |
| Plate: 3 | -6.020651782 | 2.144493095 | -2.807494133 | 0.004996634 |  |  |  |  |
| Plate: 4 | 3.151328017 | 2.144493095 | 1.469497861 | 0.141710088 |  |  |  |  |
| Plate: 5 | 3.175641904 | 2.144493095 | 1.480835686 | 0.138662669 |  |  |  |  |
| Plate: 6 | 2.382005383 | 2.144493095 | 1.110754513 | 0.266684461 |  |  |  |  |
| Plate: 7 | 1.098031908 | 1.51638561 | 0.724111269 | 0.469004043 |  |  |  |  |
| Plate: 8 | -0.005717464 | 1.51638561 | -0.003770455 | 0.996991648 |  |  |  |  |
| Plate: 10 | -2.135263217 | 2.144493095 | -0.995696009 | 0.319407279 |  |  |  |  |
| Plate: 11 | -2.254600746 | 2.144493095 | -1.051344372 | 0.293110377 |  |  |  |  |
| Plate: 12 | -2.415679551 | 2.144493095 | -1.126457137 | 0.259982631 |  |  |  |  |
| Plate: 13 | 1.074382663 | 2.144493095 | 0.500996093 | 0.616378179 |  |  |  |  |
| Plate: 14 | -0.116427445 | 2.144493095 | -0.054291359 | 0.956703457 |  |  |  |  |
| Plate: 15 | -0.334813242 | 2.144493095 | -0.156126985 | 0.875934159 |  |  |  |  |
| Plate: 16 | -5.626774521 | 1.51638561 | -3.710648851 | 0.000207166 |  |  |  |  |
| Plate: 17 | 0.366361283 | 1.51638561 | 0.241601661 | 0.809090769 |  |  |  |  |
| Plate: 19 | -0.72874536 | 2.144493095 | -0.339821733 | 0.733993568 |  |  |  |  |
| Plate: 20 | -1.485489878 | 2.144493095 | -0.692699772 | 0.488504265 |  |  |  |  |
| Plate: 21 | 3.705302017 | 2.144493095 | 1.727821846 | 0.084032228 |  |  |  |  |
| Plate: 22 | 0.835383545 | 2.144493095 | 0.389548256 | 0.696873861 |  |  |  |  |
| Plate: 23 | 0.919318518 | 2.144493095 | 0.428688029 | 0.66815388 |  |  |  |  |
| Plate: 24 | 1.136611096 | 2.144493095 | 0.530013875 | 0.596106907 |  |  |  |  |
| Plate: 25 | 22.87469134 | 1.51638561 | 15.0850095 | 3.37E-51 |  |  |  |  |
| Plate: 26 | -0.095872012 | 1.51638561 | -0.063224032 | 0.949588602 |  |  |  |  |
| Plate: 28 | -0.525180938 | 1.51638561 | -0.346337327 | 0.729092066 |  |  |  |  |
| Plate: 29 | -0.413642737 | 1.51638561 | -0.272782025 | 0.785022978 |  |  |  |  |
| Plate: 31 | 0.969504552 | 1.51638561 | 0.63935225 | 0.522599524 |  |  |  |  |
| Plate: 32 | 0.557125075 | 1.51638561 | 0.367403298 | 0.713321248 |  |  |  |  |
| Plate: 34 | -1.622432408 | 1.51638561 | -1.069933925 | 0.284659137 |  |  |  |  |
| Plate: 35 | 0.043810796 | 1.51638561 | 0.028891593 | 0.976951276 |  |  |  |  |

Supplemental Table 4: ANOVA analysis for site, dose, cell line, plate, and drug-site interaction effects. Signif. codes: 0 ‘***’ 0.001 ‘**’ 0.01 ‘*’ 0.05 ‘.’ 0.1 ‘ ’ 1
