# Supplementary material for: Analysis of variability in high throughput screening data: applications to melanoma cell lines and drug responses
Source: Oncotarget. 2017 Feb 15;8(17):27786–99. doi: 10.18632/oncotarget.15347 (PMC5438608; doi:10.18632/oncotarget.15347)
Supplement: Supplementary file 5 [file oncotarget-08-27786-s005.docx]

**Supplemental Table 6:** Factor Significance in ANOVA analysis for site, dose, cell line, plate, and drug-dose-cell line interaction effects.

| Covariate | Estimate | StErr | Tstat | Pval |
| --- | --- | --- | --- | --- |
| Intercept | 90.44336582 | 6.794574441 | 13.3111156 | 2.88E-40 |
| SBP | -6.033357009 | 1.231987927 | -4.897253355 | 9.79E-07 |
| MeWo | 6.529306364 | 9.608979326 | 0.679500511 | 0.496828086 |
| SKMEL2 | 12.14676157 | 9.608979326 | 1.264105287 | 0.206205918 |
| UACC0257 | 4.977244819 | 9.608979326 | 0.517978513 | 0.604478581 |
| Thioguanine | 10.75805293 | 9.542937448 | 1.127331389 | 0.259614954 |
| Irinotecan | 12.6239766 | 9.542937448 | 1.322860667 | 0.185895761 |
| Romidepsin | -75.32092065 | 9.542937448 | -7.892844427 | 3.09E-15 |
| Paclitaxel | -32.30443684 | 9.542937448 | -3.38516699 | 0.000712619 |
| Alisertib | -6.759788249 | 9.542937448 | -0.708355083 | 0.478732444 |
| Vorinostat | 9.173347335 | 9.542937448 | 0.961270823 | 0.336426805 |
| Busulfan | 19.34586857 | 9.542937448 | 2.027244617 | 0.042649692 |
| Mechlorethamine | 8.57381167 | 9.542937448 | 0.898445758 | 0.368957993 |
| Teniposide | -12.89096135 | 9.542937448 | -1.350837875 | 0.176761562 |
| Vinorelbine | 15.66527213 | 9.542937448 | 1.641556618 | 0.100696523 |
| Cabozantinib | 18.20324587 | 9.542937448 | 1.907509712 | 0.0564679 |
| Dacarbazine | 4.982020468 | 9.542937448 | 0.52206362 | 0.601631404 |
| Clofarabine | 0.934928761 | 9.542937448 | 0.097970752 | 0.921956429 |
| Cisplatin | 20.77510255 | 9.542937448 | 2.177013384 | 0.029490389 |
| Floxuridine | -15.57783507 | 9.542937448 | -1.63239413 | 0.102611102 |
| Lomustine | 12.82786503 | 9.542937448 | 1.344226042 | 0.17888946 |
| Melphalan | 18.176568 | 9.542937448 | 1.904714151 | 0.056830545 |
| BGJ398 | 16.99285337 | 9.542937448 | 1.780673242 | 0.074979937 |
| Navitoclax | 9.999295328 | 9.542937448 | 1.047821531 | 0.294732597 |
| Azacitidine | 3.160291335 | 9.542937448 | 0.331165467 | 0.740522725 |
| Capecitabine | 16.06134684 | 9.542937448 | 1.683061104 | 0.092377755 |
| Megestrol | 23.50600797 | 9.542937448 | 2.463183699 | 0.013778695 |
| Cytarabine | 14.54674804 | 9.542937448 | 1.524346997 | 0.127436734 |
| Gemcitabine | -33.26214887 | 9.542937448 | -3.485525191 | 0.000492149 |
| Vinblastine | -8.608035682 | 9.542937448 | -0.902032076 | 0.367049915 |
| MLN9708 | 6.218296273 | 9.542937448 | 0.651612389 | 0.514658205 |
| ABT737 | 6.181217968 | 9.542937448 | 0.647726971 | 0.51716846 |
| Streptozocin | 10.66471178 | 9.542937448 | 1.117550214 | 0.263771607 |
| Crizotinib | 19.331784 | 9.542937448 | 2.025768702 | 0.042800796 |
| Sunitinib | 11.02292728 | 9.542937448 | 1.15508745 | 0.24806743 |
| Dexrazoxane | 14.53420201 | 9.542937448 | 1.523032304 | 0.127765304 |
| MitomycinC | 12.31328108 | 9.542937448 | 1.290303027 | 0.196959317 |
| Carfilzomib | -30.75815741 | 9.542937448 | -3.223133084 | 0.001269853 |
| OSI27 | 3.749571601 | 9.542937448 | 0.392915873 | 0.694385506 |
| Bioymifi | 9.942620541 | 9.542937448 | 1.041882606 | 0.297477803 |
| Nelarabine | 7.730613018 | 9.542937448 | 0.810087361 | 0.417898917 |
| Raloxifene | 20.30720554 | 9.542937448 | 2.127982673 | 0.033349842 |
| Quinacrine | 15.63097098 | 9.542937448 | 1.637962217 | 0.101444182 |
| Lenalidomide | 14.52651753 | 9.542937448 | 1.522227051 | 0.127966879 |
| Fludarabine | 13.41192158 | 9.542937448 | 1.405429057 | 0.159908154 |
| Nilotinib | 13.9918099 | 9.542937448 | 1.466195286 | 0.14260969 |
| Linsitinib | 12.12231803 | 9.542937448 | 1.270292097 | 0.203994323 |
| Aphrocallistin | 12.18434846 | 9.542937448 | 1.276792238 | 0.201689371 |
| Mitotane | 16.08273391 | 9.542937448 | 1.685302246 | 0.091944758 |
| Etoposide | 14.3582595 | 9.542937448 | 1.50459537 | 0.132442799 |
| Vandetanib | 7.79591668 | 9.542937448 | 0.816930502 | 0.413977181 |
| Carboplatin | 15.43004523 | 9.542937448 | 1.616907301 | 0.105912936 |
| Gefitinib | 15.98966371 | 9.542937448 | 1.675549462 | 0.093840989 |
| Vincristine | -14.8692708 | 9.542937448 | -1.558144008 | 0.119213806 |
| Trametinib | -61.545353 | 9.542937448 | -6.449309066 | 1.15E-10 |
| MLN4924 | 21.18059658 | 9.542937448 | 2.219504917 | 0.026462768 |
| Bortezomib | -67.69744214 | 9.542937448 | -7.093983641 | 1.34E-12 |
| Fluorouracil | 17.83263725 | 9.542937448 | 1.868673807 | 0.061681773 |
| Lapatinib | 15.22557767 | 9.542937448 | 1.59548124 | 0.110619332 |
| Mitoxantrone | 19.79004831 | 9.542937448 | 2.073790007 | 0.038110672 |
| Imatinib | 16.63910837 | 9.542937448 | 1.743604467 | 0.08124235 |
| Imiquimod | 18.50619708 | 9.542937448 | 1.93925583 | 0.052483236 |
| Dacomitinib | 16.83041153 | 9.542937448 | 1.763651038 | 0.077804924 |
| PD325901 | -53.90358114 | 9.542937448 | -5.648531328 | 1.64E-08 |
| Vismodegib | 14.38589555 | 9.542937448 | 1.507491339 | 0.131699428 |
| Temozolomide | 18.28693861 | 9.542937448 | 1.916279836 | 0.055342706 |
| Mercaptopurine | 24.37044845 | 9.542937448 | 2.553768018 | 0.010663247 |
| Dasatinib | -4.696436309 | 9.542937448 | -0.492137388 | 0.622627239 |
| Daunorubicin | 5.724944023 | 9.542937448 | 0.599914235 | 0.548569705 |
| Sirolimus | 6.39651154 | 9.542937448 | 0.670287485 | 0.50268173 |
| INK128 | -1.750945365 | 9.542937448 | -0.183480755 | 0.854422545 |
| Quizartinib | 15.35690203 | 9.542937448 | 1.609242659 | 0.107577946 |
| Sorafenib | 12.2268586 | 9.542937448 | 1.281246856 | 0.200120766 |
| Carmustine | 22.28487428 | 9.542937448 | 2.335221666 | 0.019540977 |
| Uracil | 24.02328402 | 9.542937448 | 2.517388818 | 0.011830023 |
| Ixabepilone | 18.86472412 | 9.542937448 | 1.976825712 | 0.048074067 |
| Valrubicin | 10.46210107 | 9.542937448 | 1.09631873 | 0.272951572 |
| Triethylenemelamine | 19.07996558 | 9.542937448 | 1.999380766 | 0.04557971 |
| Palbociclib | 16.598055 | 9.542937448 | 1.739302504 | 0.081995852 |
| Afatinib | 22.96801718 | 9.542937448 | 2.406807894 | 0.016100994 |
| Doxorubicin | 8.361651651 | 9.542937448 | 0.876213608 | 0.380923691 |
| Exemestane | 21.44747462 | 9.542937448 | 2.247470943 | 0.024620023 |
| Tretinoin | 23.87216789 | 9.542937448 | 2.501553429 | 0.012372338 |
| Fulvestrant | 17.53068847 | 9.542937448 | 1.837032734 | 0.066218832 |
| Docetaxel | -42.11626174 | 9.542937448 | -4.413343582 | 1.02E-05 |
| Everolimus | 7.199769673 | 9.542937448 | 0.754460533 | 0.450580983 |
| MLN2480 | 23.42765136 | 9.542937448 | 2.454972747 | 0.014097375 |
| LY2157299 | 20.27037332 | 9.542937448 | 2.124123042 | 0.033671216 |
| Allopurinol | 12.37422385 | 9.542937448 | 1.296689192 | 0.19475204 |
| Pipobroman | 23.68030016 | 9.542937448 | 2.481447698 | 0.01309256 |
| Letrozole | 25.72741269 | 9.542937448 | 2.695963672 | 0.007023984 |
| Thiotepa | 13.77414292 | 9.542937448 | 1.443386064 | 0.14892629 |
| Plicamycin | 17.45197276 | 9.542937448 | 1.828784151 | 0.06744577 |
| Erlotinib | 11.98709967 | 9.542937448 | 1.256122629 | 0.209085137 |
| MEK162 | -6.621668437 | 9.542937448 | -0.693881572 | 0.487763964 |
| Baricitinib | 24.8904532 | 9.542937448 | 2.608259075 | 0.009106686 |
| Arsenic | 22.8300313 | 9.542937448 | 2.392348417 | 0.016749458 |
| Celecoxib | 25.81675487 | 9.542937448 | 2.705325798 | 0.00682911 |
| Bendamustine | 25.90848734 | 9.542937448 | 2.714938402 | 0.00663409 |
| Chlorambucil | 20.01846762 | 9.542937448 | 2.09772596 | 0.035940982 |
| Zoledronic | 17.3282672 | 9.542937448 | 1.815821103 | 0.069411716 |
| Actinomycin | -46.55492546 | 9.542937448 | -4.8784691 | 1.08E-06 |
| Temsirolimus | 10.38382211 | 9.542937448 | 1.088115915 | 0.276556113 |
| Foretinib | 20.95316103 | 9.542937448 | 2.195672051 | 0.028126051 |
| Decitabine | 10.90837465 | 9.542937448 | 1.143083533 | 0.253016615 |
| Methotrexate | 20.08724943 | 9.542937448 | 2.104933575 | 0.035308642 |
| Axitinib | 23.90424269 | 9.542937448 | 2.504914532 | 0.012255423 |
| Oxaliplatin | 22.15328018 | 9.542937448 | 2.321431981 | 0.020272823 |
| Cabazitaxel | -43.80591223 | 9.542937448 | -4.590401276 | 4.45E-06 |
| Amifostine | 20.20527825 | 9.542937448 | 2.11730176 | 0.034245674 |
| Flutamide | 21.12100778 | 9.542937448 | 2.213260634 | 0.026890123 |
| LDK378 | 23.10411837 | 9.542937448 | 2.421069874 | 0.015483111 |
| Pralatrexate | -77.15121143 | 9.542937448 | -8.08463975 | 6.56E-16 |
| Topotecan | 16.74096131 | 9.542937448 | 1.75427759 | 0.079397172 |
| Pemetrexed | 22.35852562 | 9.542937448 | 2.342939555 | 0.019141532 |
| Bleomycin | 16.22311485 | 9.542937448 | 1.700012699 | 0.089142974 |
| Axitinib.1 | 25.89940688 | 9.542937448 | 2.713986865 | 0.006653169 |
| Ibrutinib | 20.80316023 | 9.542937448 | 2.179953536 | 0.029271696 |
| Tamoxifen | 13.95770883 | 9.542937448 | 1.462621851 | 0.143585471 |
| Vemurafenib | 8.672427816 | 9.542937448 | 0.908779698 | 0.363476593 |
| Pazopanib | 16.43887635 | 9.542937448 | 1.722622247 | 0.084971213 |
| Abiraterone | 18.48466625 | 9.542937448 | 1.936999624 | 0.052758448 |
| Bosutinib | 25.74463267 | 9.542937448 | 2.697768146 | 0.00698604 |
| Sabutoclax | 19.95333205 | 9.542937448 | 2.090900434 | 0.036548679 |
| Ldose: -1.397940009 (uM) | 41.52245631 | 13.54253074 | 3.066078055 | 0.002171553 |
| Ldose: -1 (uM) | 5.736359373 | 13.54253074 | 0.423581049 | 0.671875608 |
| Ldose: -0.698970004 (uM) | -63.59085099 | 9.542937448 | -6.663655854 | 2.74E-11 |
| Ldose: -0.397940009 (uM) | -31.13235829 | 13.54253074 | -2.298858233 | 0.021522516 |
| Ldose: 0 (uM) | -33.49920407 | 13.54253074 | -2.473629539 | 0.013382482 |
| Ldose: 0.301029996 (uM) | -80.1766676 | 9.542937448 | -8.401675902 | 4.67E-17 |
| Ldose: 0.602059991 (uM) | -34.16172465 | 9.608979326 | -3.555187652 | 0.000378518 |
| Ldose: 1 (uM) | -33.07799235 | 9.608979326 | -3.442404362 | 0.000577663 |
| Plate: -35 | 2.542300278 | 1.231987927 | 2.063575642 | 0.039069921 |
| Plate: -34 | 3.4980325 | 1.231987927 | 2.839339918 | 0.004524929 |
| Plate: -33 | 6.157534063 | 1.742294035 | 3.534153214 | 0.000409941 |
| Plate: -32 | 5.129357119 | 1.742294035 | 2.944024955 | 0.003243198 |
| Plate: -31 | 4.968404619 | 1.742294035 | 2.851645313 | 0.004353496 |
| Plate: -30 | 3.561967451 | 1.742294035 | 2.044412355 | 0.040924959 |
| Plate: -29 | 4.511690229 | 1.742294035 | 2.589511379 | 0.00961769 |
| Plate: -28 | 2.864528284 | 1.742294035 | 1.644113007 | 0.100167455 |
| Plate: -27 | -70.76110322 | 13.58914888 | -5.207176978 | 1.93E-07 |
| Plate: -26 | -33.14015599 | 13.58914888 | -2.438721974 | 0.014747324 |
| Plate: -25 | -30.52033933 | 13.58914888 | -2.245934575 | 0.024718293 |
| Plate: -24 | -13.19668989 | 13.64488042 | -0.967153209 | 0.333478308 |
| Plate: -23 | -25.2096985 | 13.64488042 | -1.847557305 | 0.064680113 |
| Plate: -22 | -27.55654766 | 13.64488042 | -2.019552156 | 0.043442221 |
| Plate: -21 | 0.119387301 | 1.742294035 | 0.06852305 | 0.945369908 |
| Plate: -20 | 4.728863412 | 1.742294035 | 2.714159216 | 0.00664971 |
| Plate: -19 | -2.269292421 | 1.742294035 | -1.302473851 | 0.192768377 |
| Plate: -18 | -45.99312435 | 13.58914888 | -3.384547829 | 0.000714227 |
| Plate: -17 | -42.33964046 | 13.58914888 | -3.115694797 | 0.001837531 |
| Plate: -16 | -42.77528908 | 13.58914888 | -3.147753361 | 0.001647548 |
| Plate: -15 | -42.88522401 | 13.64488042 | -3.142953452 | 0.001674793 |
| Plate: -14 | -38.76262234 | 13.64488042 | -2.840818032 | 0.004504019 |
| Plate: -13 | -38.78980123 | 13.64488042 | -2.842809907 | 0.004475978 |
| Plate: -12 | 9.91574655 | 1.742294035 | 5.691201571 | 1.28E-08 |
| Plate: -11 | 8.54208405 | 1.742294035 | 4.902779828 | 9.52E-07 |
| Plate: -10 | 0.429300439 | 1.742294035 | 0.246399534 | 0.805375315 |
| Plate: -9 | -4.291972901 | 13.58914888 | -0.315838243 | 0.752128349 |
| Plate: -8 | -4.101845401 | 13.58914888 | -0.301847116 | 0.762771526 |
| Plate: -7 | -7.131511234 | 13.58914888 | -0.524794547 | 0.599731423 |
| Plate: -6 | -2.037031837 | 13.64488042 | -0.149289094 | 0.881326918 |
| Plate: -5 | -0.452886281 | 13.64488042 | -0.033190931 | 0.973522638 |
| Plate: -4 | -0.136068225 | 13.64488042 | -0.009972108 | 0.992043633 |
| Plate: -3 | 12.65066772 | 1.742294035 | 7.260925806 | 3.98E-13 |
| Plate: -2 | 13.51787772 | 1.742294035 | 7.758666132 | 8.96E-15 |
| Plate: -1 | 14.63488661 | 1.742294035 | 8.399780012 | 4.75E-17 |
| Plate: 1 | -11.58883873 | 13.58914888 | -0.852800925 | 0.393779193 |
| Plate: 2 | -15.04678188 | 13.58914888 | -1.10726448 | 0.268191961 |
| Plate: 3 | -19.69758106 | 13.58914888 | -1.449508077 | 0.147210305 |
| Plate: 4 | -9.743919543 | 13.58914888 | -0.717036779 | 0.473359178 |
| Plate: 5 | -9.719605655 | 13.58914888 | -0.715247565 | 0.474463835 |
| Plate: 6 | -10.51324218 | 13.58914888 | -0.77364979 | 0.43914641 |
| Plate: 7 | 1.098031908 | 1.231987927 | 0.8912684 | 0.372795152 |
| Plate: 8 | -0.005717464 | 1.231987927 | -0.004640844 | 0.996297198 |
| Plate: 10 | -47.56604639 | 13.58914888 | -3.500296214 | 0.000465681 |
| Plate: 11 | -47.68538392 | 13.58914888 | -3.50907804 | 0.00045058 |
| Plate: 12 | -47.84646273 | 13.58914888 | -3.520931527 | 0.000430922 |
| Plate: 13 | -47.68792839 | 13.58914888 | -3.509265283 | 0.000450263 |
| Plate: 14 | -48.8787385 | 13.58914888 | -3.596894767 | 0.000322759 |
| Plate: 15 | -49.0971243 | 13.58914888 | -3.612965368 | 0.000303404 |
| Plate: 16 | -5.626774521 | 1.231987927 | -4.567231868 | 4.97E-06 |
| Plate: 17 | 0.366361283 | 1.231987927 | 0.297374085 | 0.766183794 |
| Plate: 19 | -37.12960704 | 13.58914888 | -2.7322982 | 0.006294545 |
| Plate: 20 | -37.88635156 | 13.58914888 | -2.787985611 | 0.005308338 |
| Plate: 21 | -32.69555966 | 13.58914888 | -2.406004963 | 0.016136416 |
| Plate: 22 | -32.35424609 | 13.58914888 | -2.380888337 | 0.017279586 |
| Plate: 23 | -32.27031112 | 13.58914888 | -2.37471172 | 0.017571367 |
| Plate: 24 | -32.05301854 | 13.58914888 | -2.358721566 | 0.018346888 |
| Plate: 25 | 22.87469134 | 1.231987927 | 18.56730154 | 2.33E-76 |
| Plate: 26 | -0.095872012 | 1.231987927 | -0.077818954 | 0.937972792 |
| Plate: 28 | -0.525180938 | 1.231987927 | -0.426287407 | 0.669902693 |
| Plate: 29 | -0.413642737 | 1.231987927 | -0.335752265 | 0.73706096 |
| Plate: 31 | 0.969504552 | 1.231987927 | 0.786943225 | 0.431323761 |
| Plate: 32 | 0.557125075 | 1.231987927 | 0.452216343 | 0.65111769 |
| Plate: 34 | -1.622432408 | 1.231987927 | -1.316922327 | 0.187878672 |
| Plate: 35 | 0.043810796 | 1.231987927 | 0.03556106 | 0.971632688 |
| MeWo:Thioguanine Interaction | -5.439340815 | 13.49575156 | -0.403040971 | 0.686922084 |
| SKMEL2:Thioguanine Interaction | -16.71577077 | 13.49575156 | -1.238595027 | 0.215509008 |
| UACC0257:Thioguanine Interaction | -3.333721048 | 13.49575156 | -0.247020037 | 0.804895072 |
| MeWo:Irinotecan.HCl Interaction | -7.37964437 | 13.49575156 | -0.546812405 | 0.584513284 |
| SKMEL2:Irinotecan.HCl Interaction | -20.29566068 | 13.49575156 | -1.503855534 | 0.13263323 |
| UACC0257:Irinotecan.HCl Interaction | -10.75561309 | 13.49575156 | -0.796962884 | 0.425481372 |
| MeWo:Romidepsin Interaction | -12.44876259 | 13.49575156 | -0.922420847 | 0.356319431 |
| SKMEL2:Romidepsin Interaction | -24.79747662 | 13.49575156 | -1.837428357 | 0.06616045 |
| UACC0257:Romidepsin Interaction | 8.771200951 | 13.49575156 | 0.649923119 | 0.515748815 |
| MeWo:Paclitaxel Interaction | -1.486587921 | 13.49575156 | -0.110152289 | 0.912289626 |
| SKMEL2:Paclitaxel Interaction | -26.76171474 | 13.49575156 | -1.98297328 | 0.047383095 |
| UACC0257:Paclitaxel Interaction | -23.09861726 | 13.49575156 | -1.711547308 | 0.086994522 |
| MeWo:Alisertib..MLN8237. Interaction | 3.713111554 | 13.49575156 | 0.275131884 | 0.783217556 |
| SKMEL2:Alisertib..MLN8237. Interaction | -4.571483968 | 13.49575156 | -0.338735042 | 0.734812642 |
| UACC0257:Alisertib..MLN8237. Interaction | 0.749624629 | 13.49575156 | 0.05554523 | 0.955704612 |
| MeWo:Vorinostat Interaction | 2.570346462 | 13.49575156 | 0.190455971 | 0.848953637 |
| SKMEL2:Vorinostat Interaction | -24.12207448 | 13.49575156 | -1.787382819 | 0.073889692 |
| UACC0257:Vorinostat Interaction | -23.74326032 | 13.49575156 | -1.759313678 | 0.07853844 |
| MeWo:Busulfan Interaction | -1.63592484 | 13.49575156 | -0.121217765 | 0.903519684 |
| SKMEL2:Busulfan Interaction | -33.28312622 | 13.49575156 | -2.466192865 | 0.013663508 |
| UACC0257:Busulfan Interaction | -26.09533644 | 13.49575156 | -1.933596385 | 0.053175858 |
| MeWo:Mechlorethamine.HCl Interaction | 7.771392242 | 13.49575156 | 0.575839901 | 0.564729417 |
| SKMEL2:Mechlorethamine.HCl Interaction | -20.96426235 | 13.49575156 | -1.553397175 | 0.120342969 |
| UACC0257:Mechlorethamine.HCl Interaction | -3.106971273 | 13.49575156 | -0.23021847 | 0.817924197 |
| MeWo:Teniposide Interaction | 24.33710832 | 13.49575156 | 1.803316266 | 0.071352528 |
| SKMEL2:Teniposide Interaction | -16.16891919 | 13.49575156 | -1.198074751 | 0.2309011 |
| UACC0257:Teniposide Interaction | 12.36269796 | 13.49575156 | 0.916043682 | 0.359654239 |
| MeWo:Vinorelbine.Tartrate Interaction | -1.58152395 | 13.49575156 | -0.117186801 | 0.906713119 |
| SKMEL2:Vinorelbine.Tartrate Interaction | -32.97635537 | 13.49575156 | -2.443461946 | 0.014555074 |
| UACC0257:Vinorelbine.Tartrate Interaction | -10.8489875 | 13.49575156 | -0.803881684 | 0.42147418 |
| MeWo:Cabozantinib..XL.184. Interaction | -7.566547068 | 13.49575156 | -0.560661408 | 0.575034212 |
| SKMEL2:Cabozantinib..XL.184. Interaction | -31.60810219 | 13.49575156 | -2.342077952 | 0.019185768 |
| UACC0257:Cabozantinib..XL.184. Interaction | -2.560147485 | 13.49575156 | -0.189700253 | 0.84954581 |
| MeWo:Dacarbazine Interaction | 1.60229408 | 13.49575156 | 0.118725813 | 0.905493692 |
| SKMEL2:Dacarbazine Interaction | -14.91316596 | 13.49575156 | -1.10502671 | 0.269160358 |
| UACC0257:Dacarbazine Interaction | 0.841621087 | 13.49575156 | 0.062361928 | 0.950275192 |
| MeWo:Clofarabine Interaction | 11.52011033 | 13.49575156 | 0.853610136 | 0.393330533 |
| SKMEL2:Clofarabine Interaction | -0.208817878 | 13.49575156 | -0.01547286 | 0.98765508 |
| UACC0257:Clofarabine Interaction | 17.11317757 | 13.49575156 | 1.268041834 | 0.204796717 |
| MeWo:Cisplatin Interaction | -0.523986873 | 13.49575156 | -0.038826061 | 0.969029427 |
| SKMEL2:Cisplatin Interaction | -36.04447861 | 13.49575156 | -2.670801877 | 0.007572714 |
| UACC0257:Cisplatin Interaction | -3.245291355 | 13.49575156 | -0.240467627 | 0.809970045 |
| MeWo:Floxuridine Interaction | 31.65095105 | 13.49575156 | 2.345252941 | 0.0190232 |
| SKMEL2:Floxuridine Interaction | -1.729123496 | 13.49575156 | -0.128123542 | 0.898052402 |
| UACC0257:Floxuridine Interaction | 33.50978322 | 13.49575156 | 2.482987558 | 0.013036117 |
| MeWo:Lomustine..CCNU. Interaction | 6.27270941 | 13.49575156 | 0.464791411 | 0.642085537 |
| SKMEL2:Lomustine..CCNU. Interaction | -30.05184162 | 13.49575156 | -2.226763103 | 0.025973408 |
| UACC0257:Lomustine..CCNU. Interaction | -8.612761588 | 13.49575156 | -0.638183175 | 0.523361232 |
| MeWo:Melphalan Interaction | -0.81112204 | 13.49575156 | -0.060102028 | 0.952074932 |
| SKMEL2:Melphalan Interaction | -34.69658272 | 13.49575156 | -2.570926306 | 0.010149342 |
| UACC0257:Melphalan Interaction | -10.74720305 | 13.49575156 | -0.796339722 | 0.42584338 |
| MeWo:BGJ398..NVPBGJ398. Interaction | -0.804937306 | 13.49575156 | -0.059643755 | 0.952439922 |
| SKMEL2:BGJ398..NVPBGJ398. Interaction | -33.00631688 | 13.49575156 | -2.445682015 | 0.014465792 |
| UACC0257:BGJ398..NVPBGJ398. Interaction | 1.255040425 | 13.49575156 | 0.092995223 | 0.925908221 |
| MeWo:Navitoclax..ABT.263..5uM Interaction | 1.9208291 | 13.49575156 | 0.142328428 | 0.886821924 |
| SKMEL2:Navitoclax..ABT.263..5uM Interaction | -27.80277393 | 13.49575156 | -2.060113051 | 0.039399719 |
| UACC0257:Navitoclax..ABT.263..5uM Interaction | -10.58425606 | 13.49575156 | -0.784265775 | 0.432892802 |
| MeWo:Azacitidine Interaction | 6.115892767 | 13.49575156 | 0.453171707 | 0.650429669 |
| SKMEL2:Azacitidine Interaction | -11.5963214 | 13.49575156 | -0.859257178 | 0.390208206 |
| UACC0257:Azacitidine Interaction | 2.742250234 | 13.49575156 | 0.203193592 | 0.838985641 |
| MeWo:Capecitabine Interaction | 8.104019624 | 13.49575156 | 0.600486722 | 0.548188225 |
| SKMEL2:Capecitabine Interaction | -29.4683267 | 13.49575156 | -2.183526168 | 0.029007837 |
| UACC0257:Capecitabine Interaction | -2.863215558 | 13.49575156 | -0.212156807 | 0.83198671 |
| MeWo:Megestrol.acetate Interaction | 0.763060862 | 13.49575156 | 0.05654082 | 0.954911502 |
| SKMEL2:Megestrol.acetate Interaction | -36.38499737 | 13.49575156 | -2.696033429 | 0.007022514 |
| UACC0257:Megestrol.acetate Interaction | 0.320983064 | 13.49575156 | 0.023784008 | 0.981025116 |
| MeWo:Cytarabine.HCl...Ara.C Interaction | 7.040424202 | 13.49575156 | 0.521677075 | 0.601900553 |
| SKMEL2:Cytarabine.HCl...Ara.C Interaction | -25.84311409 | 13.49575156 | -1.914907367 | 0.055517548 |
| UACC0257:Cytarabine.HCl...Ara.C Interaction | 13.72024292 | 13.49575156 | 1.016634224 | 0.309338886 |
| MeWo:Gemcitabine.HCl Interaction | 30.47450721 | 13.49575156 | 2.258081521 | 0.023950542 |
| SKMEL2:Gemcitabine.HCl Interaction | -33.53234638 | 13.49575156 | -2.484659429 | 0.012975078 |
| UACC0257:Gemcitabine.HCl Interaction | 23.8863461 | 13.49575156 | 1.769915961 | 0.076755286 |
| MeWo:Vinblastine.Sulfate Interaction | 11.98354101 | 13.49575156 | 0.887949141 | 0.374578024 |
| SKMEL2:Vinblastine.Sulfate Interaction | -49.44189258 | 13.49575156 | -3.663515318 | 0.000249374 |
| UACC0257:Vinblastine.Sulfate Interaction | -6.193613405 | 13.49575156 | -0.458930603 | 0.646288632 |
| MeWo:MLN9708..MLN2238. Interaction | 16.43337581 | 13.49575156 | 1.217670296 | 0.223362628 |
| SKMEL2:MLN9708..MLN2238. Interaction | -22.03534778 | 13.49575156 | -1.632761812 | 0.102533718 |
| UACC0257:MLN9708..MLN2238. Interaction | 2.008726574 | 13.49575156 | 0.148841401 | 0.881680174 |
| MeWo:ABT.737 Interaction | 9.412108844 | 13.49575156 | 0.697412723 | 0.485552069 |
| SKMEL2:ABT.737 Interaction | -23.62498251 | 13.49575156 | -1.750549601 | 0.080037759 |
| UACC0257:ABT.737 Interaction | 10.09579598 | 13.49575156 | 0.748072157 | 0.454424803 |
| MeWo:Streptozocin Interaction | -1.48597484 | 13.49575156 | -0.110106861 | 0.912325653 |
| SKMEL2:Streptozocin Interaction | -20.1118657 | 13.49575156 | -1.490236805 | 0.136176624 |
| UACC0257:Streptozocin Interaction | -8.542600506 | 13.49575156 | -0.632984422 | 0.526750536 |
| MeWo:Crizotinib Interaction | 5.424441657 | 13.49575156 | 0.401936982 | 0.687734393 |
| SKMEL2:Crizotinib Interaction | -32.32301144 | 13.49575156 | -2.395050863 | 0.016626548 |
| UACC0257:Crizotinib Interaction | 7.772756125 | 13.49575156 | 0.575940961 | 0.564661105 |
| MeWo:Sunitinib Interaction | 11.33518294 | 13.49575156 | 0.839907499 | 0.400969575 |
| SKMEL2:Sunitinib Interaction | -25.22515467 | 13.49575156 | -1.869118185 | 0.061619933 |
| UACC0257:Sunitinib Interaction | -5.405577705 | 13.49575156 | -0.400539213 | 0.68876338 |
| MeWo:Dexrazoxane Interaction | 8.821524719 | 13.49575156 | 0.653651979 | 0.513343025 |
| SKMEL2:Dexrazoxane Interaction | -27.96310529 | 13.49575156 | -2.071993187 | 0.038277948 |
| UACC0257:Dexrazoxane Interaction | 1.422345437 | 13.49575156 | 0.105392088 | 0.916065675 |
| MeWo:Mitomycin.C Interaction | 13.98595978 | 13.49575156 | 1.036323151 | 0.300063043 |
| SKMEL2:Mitomycin.C Interaction | -24.1995085 | 13.49575156 | -1.793120478 | 0.072967686 |
| UACC0257:Mitomycin.C Interaction | 7.621203735 | 13.49575156 | 0.564711324 | 0.572276011 |
| MeWo:Carfilzomib Interaction | 33.3263538 | 13.49575156 | 2.469395917 | 0.013541834 |
| SKMEL2:Carfilzomib Interaction | -37.49178344 | 13.49575156 | -2.778043391 | 0.005473463 |
| UACC0257:Carfilzomib Interaction | 38.38569029 | 13.49575156 | 2.844279558 | 0.004455391 |
| MeWo:OSI.027 Interaction | 18.01487213 | 13.49575156 | 1.33485505 | 0.1819379 |
| SKMEL2:OSI.027 Interaction | -22.20298034 | 13.49575156 | -1.645182948 | 0.09994668 |
| UACC0257:OSI.027 Interaction | 16.22271331 | 13.49575156 | 1.202060755 | 0.229353205 |
| MeWo:Bioymifi Interaction | 9.98937837 | 13.49575156 | 0.740186889 | 0.459194669 |
| SKMEL2:Bioymifi Interaction | -22.59190567 | 13.49575156 | -1.674001301 | 0.094144862 |
| UACC0257:Bioymifi Interaction | 10.74527032 | 13.49575156 | 0.796196512 | 0.425926599 |
| MeWo:Nelarabine Interaction | 1.987786674 | 13.49575156 | 0.147289809 | 0.882904654 |
| SKMEL2:Nelarabine Interaction | -17.2801141 | 13.49575156 | -1.280411396 | 0.200414276 |
| UACC0257:Nelarabine Interaction | -2.458189111 | 13.49575156 | -0.182145403 | 0.855470332 |
| MeWo:Raloxifene Interaction | 3.63881554 | 13.49575156 | 0.269626743 | 0.787450007 |
| SKMEL2:Raloxifene Interaction | -35.0224716 | 13.49575156 | -2.595073823 | 0.009463471 |
| UACC0257:Raloxifene Interaction | 4.904649842 | 13.49575156 | 0.363421764 | 0.716293413 |
| MeWo:Quinacrine.HCl Interaction | 10.2087843 | 13.49575156 | 0.756444296 | 0.449391131 |
| SKMEL2:Quinacrine.HCl Interaction | -30.85329222 | 13.49575156 | -2.286148502 | 0.022255235 |
| UACC0257:Quinacrine.HCl Interaction | -7.298574468 | 13.49575156 | -0.540805337 | 0.588647356 |
| MeWo:Lenalidomide Interaction | 8.228094834 | 13.49575156 | 0.609680372 | 0.54207999 |
| SKMEL2:Lenalidomide Interaction | -28.53065509 | 13.49575156 | -2.114047147 | 0.034522701 |
| UACC0257:Lenalidomide Interaction | -3.511293381 | 13.49575156 | -0.260177684 | 0.794729204 |
| MeWo:Fludarabine.Phosphate Interaction | 15.5675978 | 13.49575156 | 1.153518404 | 0.248710467 |
| SKMEL2:Fludarabine.Phosphate Interaction | -28.08382248 | 13.49575156 | -2.080938016 | 0.037451365 |
| UACC0257:Fludarabine.Phosphate Interaction | -3.946368316 | 13.49575156 | -0.292415602 | 0.769971697 |
| MeWo:Nilotinib Interaction | 13.24220125 | 13.49575156 | 0.981212583 | 0.326498941 |
| SKMEL2:Nilotinib Interaction | -30.14445553 | 13.49575156 | -2.233625551 | 0.025517946 |
| UACC0257:Nilotinib Interaction | 3.769541765 | 13.49575156 | 0.279313216 | 0.780007147 |
| MeWo:Linsitinib Interaction | 10.80556389 | 13.49575156 | 0.800664108 | 0.423334957 |
| SKMEL2:Linsitinib Interaction | -29.13280479 | 13.49575156 | -2.15866487 | 0.030887186 |
| UACC0257:Linsitinib Interaction | 9.241750504 | 13.49575156 | 0.684789614 | 0.493484058 |
| MeWo:Aphrocallistin.analogue Interaction | -0.799328905 | 13.49575156 | -0.059228188 | 0.952770908 |
| SKMEL2:Aphrocallistin.analogue Interaction | -16.3176273 | 13.49575156 | -1.209093634 | 0.226640146 |
| UACC0257:Aphrocallistin.analogue Interaction | -3.123738161 | 13.49575156 | -0.231460852 | 0.816958994 |
| MeWo:Mitotane..o.p..DDD..Lysodren. Interaction | -6.931407011 | 13.49575156 | -0.513599186 | 0.607537517 |
| SKMEL2:Mitotane..o.p..DDD..Lysodren. Interaction | -21.76836168 | 13.49575156 | -1.612978839 | 0.106763755 |
| UACC0257:Mitotane..o.p..DDD..Lysodren. Interaction | -12.78540946 | 13.49575156 | -0.947365503 | 0.343463191 |
| MeWo:Etoposide Interaction | 11.78711861 | 13.49575156 | 0.873394753 | 0.382457686 |
| SKMEL2:Etoposide Interaction | -30.42299616 | 13.49575156 | -2.254264686 | 0.024189526 |
| UACC0257:Etoposide Interaction | 5.772260792 | 13.49575156 | 0.427709473 | 0.668866928 |
| MeWo:Vandetanib Interaction | 19.56127433 | 13.49575156 | 1.449439421 | 0.147229465 |
| SKMEL2:Vandetanib Interaction | -23.17815075 | 13.49575156 | -1.717440532 | 0.085913085 |
| UACC0257:Vandetanib Interaction | 1.759474684 | 13.49575156 | 0.130372486 | 0.896272951 |
| MeWo:Carboplatin Interaction | 8.743686849 | 13.49575156 | 0.647884396 | 0.517066629 |
| SKMEL2:Carboplatin Interaction | -31.2346623 | 13.49575156 | -2.314407031 | 0.020654763 |
| UACC0257:Carboplatin Interaction | -1.1162192 | 13.49575156 | -0.082708932 | 0.934083751 |
| MeWo:Gefitinib Interaction | 7.584965504 | 13.49575156 | 0.562026166 | 0.57410404 |
| SKMEL2:Gefitinib Interaction | -29.55847192 | 13.49575156 | -2.190205694 | 0.028520005 |
| UACC0257:Gefitinib Interaction | -11.61632493 | 13.49575156 | -0.860739387 | 0.389391176 |
| MeWo:Vincristine.Sulfate Interaction | -6.869407685 | 13.49575156 | -0.509005197 | 0.610753795 |
| SKMEL2:Vincristine.Sulfate Interaction | -44.3571447 | 13.49575156 | -3.286748758 | 0.001015133 |
| UACC0257:Vincristine.Sulfate Interaction | -27.80368421 | 13.49575156 | -2.0601805 | 0.039393272 |
| MeWo:Trametinib..GSK1120212. Interaction | 32.41895581 | 13.49575156 | 2.402160092 | 0.016306985 |
| SKMEL2:Trametinib..GSK1120212. Interaction | -10.0224634 | 13.49575156 | -0.742638404 | 0.457708729 |
| UACC0257:Trametinib..GSK1120212. Interaction | 4.384328422 | 13.49575156 | 0.324867304 | 0.745284659 |
| MeWo:MLN4924 Interaction | -1.86551514 | 13.49575156 | -0.138229807 | 0.89006009 |
| SKMEL2:MLN4924 Interaction | -39.92108484 | 13.49575156 | -2.95804829 | 0.003099299 |
| UACC0257:MLN4924 Interaction | -2.426279488 | 13.49575156 | -0.179780983 | 0.857326205 |
| MeWo:Bortezomib Interaction | -8.91877696 | 13.49575156 | -0.660858117 | 0.508710376 |
| SKMEL2:Bortezomib Interaction | -23.36450074 | 13.49575156 | -1.731248581 | 0.083421757 |
| UACC0257:Bortezomib Interaction | -21.30040225 | 13.49575156 | -1.578304265 | 0.114510411 |
| MeWo:Fluorouracil...5.FU. Interaction | 6.408006462 | 13.49575156 | 0.47481657 | 0.634922536 |
| SKMEL2:Fluorouracil...5.FU. Interaction | -31.5911855 | 13.49575156 | -2.34082447 | 0.019250283 |
| UACC0257:Fluorouracil...5.FU. Interaction | -3.26237502 | 13.49575156 | -0.241733482 | 0.808988985 |
| MeWo:Lapatinib Interaction | 10.47023387 | 13.49575156 | 0.775817028 | 0.437865546 |
| SKMEL2:Lapatinib Interaction | -27.09117083 | 13.49575156 | -2.007385117 | 0.04472114 |
| UACC0257:Lapatinib Interaction | -9.212938295 | 13.49575156 | -0.682654704 | 0.494832399 |
| MeWo:Mitoxantrone Interaction | 2.637861715 | 13.49575156 | 0.195458675 | 0.84503573 |
| SKMEL2:Mitoxantrone Interaction | -56.23185949 | 13.49575156 | -4.16663416 | 3.10E-05 |
| UACC0257:Mitoxantrone Interaction | 2.681231217 | 13.49575156 | 0.198672242 | 0.842521014 |
| MeWo:Imatinib Interaction | 9.807523662 | 13.49575156 | 0.72671193 | 0.467410341 |
| SKMEL2:Imatinib Interaction | -30.85633594 | 13.49575156 | -2.286374033 | 0.022242046 |
| UACC0257:Imatinib Interaction | -10.72626275 | 13.49575156 | -0.794788101 | 0.426745531 |
| MeWo:Imiquimod Interaction | 8.22070143 | 13.49575156 | 0.60913254 | 0.542443015 |
| SKMEL2:Imiquimod Interaction | -35.80497715 | 13.49575156 | -2.653055443 | 0.007982527 |
| UACC0257:Imiquimod Interaction | -13.70890972 | 13.49575156 | -1.015794463 | 0.309738683 |
| MeWo:Dacomitinib..PF299804. Interaction | 7.396876745 | 13.49575156 | 0.548089279 | 0.583636281 |
| SKMEL2:Dacomitinib..PF299804. Interaction | -34.48115298 | 13.49575156 | -2.554963525 | 0.010626704 |
| UACC0257:Dacomitinib..PF299804. Interaction | -8.569023736 | 13.49575156 | -0.634942315 | 0.525472782 |
| MeWo:PD325901 Interaction | 46.14635792 | 13.49575156 | 3.419324793 | 0.00062893 |
| SKMEL2:PD325901 Interaction | 15.76700949 | 13.49575156 | 1.16829429 | 0.242701008 |
| UACC0257:PD325901 Interaction | 7.770654042 | 13.49575156 | 0.575785202 | 0.564766392 |
| MeWo:Vismodegib Interaction | -5.351701556 | 13.49575156 | -0.39654713 | 0.691705375 |
| SKMEL2:Vismodegib Interaction | -21.22947767 | 13.49575156 | -1.573048938 | 0.115722174 |
| UACC0257:Vismodegib Interaction | -11.39370782 | 13.49575156 | -0.84424404 | 0.398542426 |
| MeWo:Temozolomide Interaction | 1.196903439 | 13.49575156 | 0.088687424 | 0.929331152 |
| SKMEL2:Temozolomide Interaction | -30.94017355 | 13.49575156 | -2.292586182 | 0.021881434 |
| UACC0257:Temozolomide Interaction | -0.215781943 | 13.49575156 | -0.015988879 | 0.987243412 |
| MeWo:Mercaptopurine Interaction | -2.130786553 | 13.49575156 | -0.157885727 | 0.874548325 |
| SKMEL2:Mercaptopurine Interaction | -40.81538578 | 13.49575156 | -3.024313658 | 0.002494914 |
| UACC0257:Mercaptopurine Interaction | -20.32843408 | 13.49575156 | -1.506283958 | 0.132008958 |
| MeWo:Dasatinib Interaction | 21.06596637 | 13.49575156 | 1.560933177 | 0.118554209 |
| SKMEL2:Dasatinib Interaction | -19.89107084 | 13.49575156 | -1.473876482 | 0.140529464 |
| UACC0257:Dasatinib Interaction | 3.047328289 | 13.49575156 | 0.22579908 | 0.821359835 |
| MeWo:Daunorubicin.HCl Interaction | 18.63537476 | 13.49575156 | 1.380832677 | 0.16734474 |
| SKMEL2:Daunorubicin.HCl Interaction | -33.3662237 | 13.49575156 | -2.472350172 | 0.013430461 |
| UACC0257:Daunorubicin.HCl Interaction | 16.65245744 | 13.49575156 | 1.233903674 | 0.217252263 |
| MeWo:Sirolimus..Rapamycin. Interaction | -13.55745729 | 13.49575156 | -1.004572234 | 0.315114158 |
| SKMEL2:Sirolimus..Rapamycin. Interaction | -33.34566916 | 13.49575156 | -2.470827134 | 0.013487777 |
| UACC0257:Sirolimus..Rapamycin. Interaction | 7.287033432 | 13.49575156 | 0.539950176 | 0.589236975 |
| MeWo:INK.128..MLN0128. Interaction | -19.58610024 | 13.49575156 | -1.451278956 | 0.146716762 |
| SKMEL2:INK.128..MLN0128. Interaction | -4.179036418 | 13.49575156 | -0.309655701 | 0.756825788 |
| UACC0257:INK.128..MLN0128. Interaction | -6.614653263 | 13.49575156 | -0.490128559 | 0.624047922 |
| MeWo:Quizartinib Interaction | 1.787975994 | 13.49575156 | 0.132484359 | 0.894602431 |
| SKMEL2:Quizartinib Interaction | -27.72321448 | 13.49575156 | -2.054217904 | 0.039966646 |
| UACC0257:Quizartinib Interaction | 8.572162679 | 13.49575156 | 0.635174902 | 0.525321097 |
| MeWo:Sorafenib Interaction | -3.896264103 | 13.49575156 | -0.28870301 | 0.772811437 |
| SKMEL2:Sorafenib Interaction | -22.58837683 | 13.49575156 | -1.673739823 | 0.094196263 |
| UACC0257:Sorafenib Interaction | -9.923872005 | 13.49575156 | -0.735333039 | 0.462144698 |
| MeWo:Carmustine Interaction | -3.10586184 | 13.49575156 | -0.230136264 | 0.817988072 |
| SKMEL2:Carmustine Interaction | -38.43947276 | 13.49575156 | -2.848264698 | 0.004399997 |
| UACC0257:Carmustine Interaction | -1.410789455 | 13.49575156 | -0.10453582 | 0.916745117 |
| MeWo:Uracil.mustard Interaction | -6.908266273 | 13.49575156 | -0.511884517 | 0.608737082 |
| SKMEL2:Uracil.mustard Interaction | -40.20570672 | 13.49575156 | -2.97913803 | 0.002893828 |
| UACC0257:Uracil.mustard Interaction | -6.280649055 | 13.49575156 | -0.465379718 | 0.641664261 |
| MeWo:Ixabepilone Interaction | 1.313928377 | 13.49575156 | 0.097358667 | 0.922442474 |
| SKMEL2:Ixabepilone Interaction | -37.17863248 | 13.49575156 | -2.754839722 | 0.005877019 |
| UACC0257:Ixabepilone Interaction | -14.47310825 | 13.49575156 | -1.072419582 | 0.283543631 |
| MeWo:Valrubicin Interaction | 6.366242694 | 13.49575156 | 0.471721983 | 0.637130022 |
| SKMEL2:Valrubicin Interaction | -36.49364407 | 13.49575156 | -2.704083867 | 0.006854678 |
| UACC0257:Valrubicin Interaction | -1.535175388 | 13.49575156 | -0.113752493 | 0.909435059 |
| MeWo:Triethylenemelamine Interaction | 1.09323676 | 13.49575156 | 0.081005993 | 0.935437939 |
| SKMEL2:Triethylenemelamine Interaction | -37.32205046 | 13.49575156 | -2.76546662 | 0.005688981 |
| UACC0257:Triethylenemelamine Interaction | -12.58737537 | 13.49575156 | -0.932691693 | 0.350989629 |
| MeWo:Palbociclib..PD.0332991..Isethionate Interaction | 0.214237044 | 13.49575156 | 0.015874406 | 0.987334735 |
| SKMEL2:Palbociclib..PD.0332991..Isethionate Interaction | -35.05865792 | 13.49575156 | -2.597755134 | 0.009389923 |
| UACC0257:Palbociclib..PD.0332991..Isethionate Interaction | -8.610777838 | 13.49575156 | -0.638036185 | 0.523456908 |
| MeWo:Afatinib Interaction | -6.67383799 | 13.49575156 | -0.494513993 | 0.62094827 |
| SKMEL2:Afatinib Interaction | -40.55998319 | 13.49575156 | -3.005388992 | 0.002655467 |
| UACC0257:Afatinib Interaction | -6.249012121 | 13.49575156 | -0.463035504 | 0.643343597 |
| MeWo:Doxorubicin.HCl Interaction | 5.34878191 | 13.49575156 | 0.396330792 | 0.69186494 |
| SKMEL2:Doxorubicin.HCl Interaction | -19.01796796 | 13.49575156 | -1.409181835 | 0.158795844 |
| UACC0257:Doxorubicin.HCl Interaction | 1.825422407 | 13.49575156 | 0.13525904 | 0.892408332 |
| MeWo:Exemestane Interaction | -1.20414001 | 13.49575156 | -0.089223635 | 0.928905012 |
| SKMEL2:Exemestane Interaction | -37.68656732 | 13.49575156 | -2.792476369 | 0.00523524 |
| UACC0257:Exemestane Interaction | 0.860618075 | 13.49575156 | 0.063769555 | 0.949154312 |
| MeWo:Tretinoin Interaction | -4.492811831 | 13.49575156 | -0.332905641 | 0.739208751 |
| SKMEL2:Tretinoin Interaction | -40.4056805 | 13.49575156 | -2.993955565 | 0.002756986 |
| UACC0257:Tretinoin Interaction | -10.47695161 | 13.49575156 | -0.776314795 | 0.437571663 |
| MeWo:Fulvestrant Interaction | 2.732375877 | 13.49575156 | 0.202461928 | 0.839557532 |
| SKMEL2:Fulvestrant Interaction | -33.15263683 | 13.49575156 | -2.456523942 | 0.014036677 |
| UACC0257:Fulvestrant Interaction | -11.18924595 | 13.49575156 | -0.829093949 | 0.407060435 |
| MeWo:Docetaxel Interaction | 12.07727779 | 13.49575156 | 0.894894792 | 0.37085333 |
| SKMEL2:Docetaxel Interaction | -21.34990832 | 13.49575156 | -1.581972536 | 0.113670522 |
| UACC0257:Docetaxel Interaction | -9.119897085 | 13.49575156 | -0.675760593 | 0.499199913 |
| MeWo:Everolimus Interaction | -13.52525468 | 13.49575156 | -1.002186104 | 0.316264976 |
| SKMEL2:Everolimus Interaction | -33.96471462 | 13.49575156 | -2.516696789 | 0.011853275 |
| UACC0257:Everolimus Interaction | 4.872026449 | 13.49575156 | 0.361004456 | 0.718099657 |
| MeWo:MLN.2480 Interaction | 0.135112549 | 13.49575156 | 0.010011488 | 0.992012215 |
| SKMEL2:MLN.2480 Interaction | -42.88573058 | 13.49575156 | -3.177720809 | 0.001486472 |
| UACC0257:MLN.2480 Interaction | -2.469998083 | 13.49575156 | -0.183020417 | 0.854783721 |
| MeWo:LY2157299 Interaction | -0.211079456 | 13.49575156 | -0.015640437 | 0.98752139 |
| SKMEL2:LY2157299 Interaction | -35.17580041 | 13.49575156 | -2.606435087 | 0.009155316 |
| UACC0257:LY2157299 Interaction | -9.867509655 | 13.49575156 | -0.731156735 | 0.464691376 |
| MeWo:Allopurinol Interaction | 0.80816011 | 13.49575156 | 0.059882557 | 0.952249728 |
| SKMEL2:Allopurinol Interaction | -21.03405355 | 13.49575156 | -1.55856852 | 0.11911323 |
| UACC0257:Allopurinol Interaction | -3.439088496 | 13.49575156 | -0.25482749 | 0.798858775 |
| MeWo:Pipobroman Interaction | -0.973436063 | 13.49575156 | -0.072129074 | 0.942499858 |
| SKMEL2:Pipobroman Interaction | -33.61829839 | 13.49575156 | -2.49102825 | 0.012744868 |
| UACC0257:Pipobroman Interaction | -2.111775678 | 13.49575156 | -0.156477071 | 0.875658458 |
| MeWo:Letrozole Interaction | -3.43583106 | 13.49575156 | -0.254586122 | 0.799045209 |
| SKMEL2:Letrozole Interaction | -41.6511535 | 13.49575156 | -3.086241866 | 0.00202961 |
| UACC0257:Letrozole Interaction | -2.262153691 | 13.49575156 | -0.16761969 | 0.866884054 |
| MeWo:Thiotepa Interaction | 7.387358992 | 13.49575156 | 0.547384038 | 0.58412059 |
| SKMEL2:Thiotepa Interaction | -30.15210665 | 13.49575156 | -2.23419248 | 0.02548063 |
| UACC0257:Thiotepa Interaction | -7.702221073 | 13.49575156 | -0.570714498 | 0.568199143 |
| MeWo:Plicamycin Interaction | 2.799198669 | 13.49575156 | 0.207413322 | 0.83568904 |
| SKMEL2:Plicamycin Interaction | -33.88847907 | 13.49575156 | -2.511047933 | 0.012044596 |
| UACC0257:Plicamycin Interaction | -6.320089263 | 13.49575156 | -0.468302134 | 0.639573281 |
| MeWo:Erlotinib.HCl Interaction | 9.057075837 | 13.49575156 | 0.671105703 | 0.502160391 |
| SKMEL2:Erlotinib.HCl Interaction | -28.34611431 | 13.49575156 | -2.100373157 | 0.035707624 |
| UACC0257:Erlotinib.HCl Interaction | 5.716940589 | 13.49575156 | 0.42361039 | 0.671854206 |
| MeWo:MEK.162..ARRY.438162. Interaction | 8.768511482 | 13.49575156 | 0.649723836 | 0.515877552 |
| SKMEL2:MEK.162..ARRY.438162. Interaction | -33.33556911 | 13.49575156 | -2.470078747 | 0.01351602 |
| UACC0257:MEK.162..ARRY.438162. Interaction | -17.16572882 | 13.49575156 | -1.271935745 | 0.203409682 |
| MeWo:Baricitinib..LY3009104..INCB028050. Interaction | -8.130083856 | 13.49575156 | -0.602418014 | 0.546902263 |
| SKMEL2:Baricitinib..LY3009104..INCB028050. Interaction | -39.28953918 | 13.49575156 | -2.911252404 | 0.003603521 |
| UACC0257:Baricitinib..LY3009104..INCB028050. Interaction | 0.337831345 | 13.49575156 | 0.025032422 | 0.980029335 |
| MeWo:Arsenic.Trioxide Interaction | -12.27778917 | 13.49575156 | -0.909752163 | 0.362963407 |
| SKMEL2:Arsenic.Trioxide Interaction | -32.43109088 | 13.49575156 | -2.403059268 | 0.016266954 |
| UACC0257:Arsenic.Trioxide Interaction | -14.19149732 | 13.49575156 | -1.051552946 | 0.293016502 |
| MeWo:Celecoxib Interaction | -9.84051104 | 13.49575156 | -0.729156208 | 0.465914043 |
| SKMEL2:Celecoxib Interaction | -42.57310898 | 13.49575156 | -3.154556364 | 0.001609633 |
| UACC0257:Celecoxib Interaction | -2.857982421 | 13.49575156 | -0.211769045 | 0.832289223 |
| MeWo:Bendamustine.HCl Interaction | -5.065874266 | 13.49575156 | -0.375368074 | 0.707390442 |
| SKMEL2:Bendamustine.HCl Interaction | -43.86658452 | 13.49575156 | -3.250399528 | 0.001154199 |
| UACC0257:Bendamustine.HCl Interaction | -3.194596615 | 13.49575156 | -0.236711279 | 0.812883035 |
| MeWo:Chlorambucil Interaction | -0.74650859 | 13.49575156 | -0.05531434 | 0.955888551 |
| SKMEL2:Chlorambucil Interaction | -39.37210664 | 13.49575156 | -2.917370437 | 0.003533609 |
| UACC0257:Chlorambucil Interaction | 5.424744045 | 13.49575156 | 0.401959388 | 0.687717903 |
| MeWo:Zoledronic.Acid Interaction | -1.143663573 | 13.49575156 | -0.084742489 | 0.932466904 |
| SKMEL2:Zoledronic.Acid Interaction | -34.74296053 | 13.49575156 | -2.574362781 | 0.010049108 |
| UACC0257:Zoledronic.Acid Interaction | -9.654599671 | 13.49575156 | -0.715380661 | 0.474381613 |
| MeWo:Actinomycin.D Interaction | 29.37830502 | 13.49575156 | 2.176855796 | 0.02950215 |
| SKMEL2:Actinomycin.D Interaction | 21.13965163 | 13.49575156 | 1.566393063 | 0.117271316 |
| UACC0257:Actinomycin.D Interaction | 21.52938426 | 13.49575156 | 1.595271235 | 0.110666266 |
| MeWo:Temsirolimus..CCI.779..Torisel. Interaction | -20.65283876 | 13.49575156 | -1.530321499 | 0.125951854 |
| SKMEL2:Temsirolimus..CCI.779..Torisel. Interaction | -36.04436496 | 13.49575156 | -2.670793456 | 0.007572903 |
| UACC0257:Temsirolimus..CCI.779..Torisel. Interaction | -8.847420481 | 13.49575156 | -0.655570788 | 0.512107328 |
| MeWo:Foretinib..GSK1363089. Interaction | -10.37806622 | 13.49575156 | -0.768987646 | 0.441909076 |
| SKMEL2:Foretinib..GSK1363089. Interaction | -36.20400194 | 13.49575156 | -2.68262214 | 0.007310322 |
| UACC0257:Foretinib..GSK1363089. Interaction | -7.859841955 | 13.49575156 | -0.582393794 | 0.560307557 |
| MeWo:Decitabine Interaction | -3.453901806 | 13.49575156 | -0.255925118 | 0.798011103 |
| SKMEL2:Decitabine Interaction | -16.56413105 | 13.49575156 | -1.227358919 | 0.219701133 |
| UACC0257:Decitabine Interaction | -4.643585338 | 13.49575156 | -0.344077565 | 0.730791313 |
| MeWo:Methotrexate Interaction | -5.23061424 | 13.49575156 | -0.387574876 | 0.698334494 |
| SKMEL2:Methotrexate Interaction | -32.5984223 | 13.49575156 | -2.41545809 | 0.0157237 |
| UACC0257:Methotrexate Interaction | -0.522018838 | 13.49575156 | -0.038680235 | 0.969145691 |
| MeWo:Axitinib Interaction | -9.416161533 | 13.49575156 | -0.697713016 | 0.485364217 |
| SKMEL2:Axitinib Interaction | -36.24955705 | 13.49575156 | -2.685997655 | 0.007236903 |
| UACC0257:Axitinib Interaction | -2.481665615 | 13.49575156 | -0.183884951 | 0.854105442 |
| MeWo:Oxaliplatin Interaction | 0.683609777 | 13.49575156 | 0.050653702 | 0.959601939 |
| SKMEL2:Oxaliplatin Interaction | -38.8148407 | 13.49575156 | -2.876078484 | 0.004030445 |
| UACC0257:Oxaliplatin Interaction | -3.387596805 | 13.49575156 | -0.25101209 | 0.801807156 |
| MeWo:Cabazitaxel Interaction | 20.8692032 | 13.49575156 | 1.54635354 | 0.122033899 |
| SKMEL2:Cabazitaxel Interaction | -21.29060027 | 13.49575156 | -1.577577963 | 0.114677283 |
| UACC0257:Cabazitaxel Interaction | 6.956035902 | 13.49575156 | 0.515424122 | 0.606261969 |
| MeWo:Amifostine Interaction | 0.009634327 | 13.49575156 | 0.000713879 | 0.999430414 |
| SKMEL2:Amifostine Interaction | -35.89663115 | 13.49575156 | -2.659846766 | 0.007823408 |
| UACC0257:Amifostine Interaction | 2.044708895 | 13.49575156 | 0.151507597 | 0.879576737 |
| MeWo:Flutamide..Eulexin. Interaction | -3.07882839 | 13.49575156 | -0.228133155 | 0.819544891 |
| SKMEL2:Flutamide..Eulexin. Interaction | -35.55128763 | 13.49575156 | -2.634257711 | 0.008438176 |
| UACC0257:Flutamide..Eulexin. Interaction | 4.848850229 | 13.49575156 | 0.359287158 | 0.719383802 |
| MeWo:LDK378 Interaction | -6.702070606 | 13.49575156 | -0.496605956 | 0.61947202 |
| SKMEL2:LDK378 Interaction | -37.82830548 | 13.49575156 | -2.802978796 | 0.005067824 |
| UACC0257:LDK378 Interaction | -0.819026455 | 13.49575156 | -0.060687725 | 0.951608471 |
| MeWo:Pralatrexate Interaction | 30.50922098 | 13.49575156 | 2.26065372 | 0.023790645 |
| SKMEL2:Pralatrexate Interaction | 29.20042545 | 13.49575156 | 2.163675384 | 0.03050023 |
| UACC0257:Pralatrexate Interaction | 7.385832554 | 13.49575156 | 0.547270933 | 0.58419828 |
| MeWo:Topotecan.HCl Interaction | -4.381208226 | 13.49575156 | -0.324636105 | 0.74545965 |
| SKMEL2:Topotecan.HCl Interaction | -34.24262319 | 13.49575156 | -2.537289089 | 0.011178457 |
| UACC0257:Topotecan.HCl Interaction | 1.312725024 | 13.49575156 | 0.097269501 | 0.922513281 |
| MeWo:Pemetrexed Interaction | -8.386567573 | 13.49575156 | -0.621422789 | 0.534328058 |
| SKMEL2:Pemetrexed Interaction | -37.4668622 | 13.49575156 | -2.776196792 | 0.005504638 |
| UACC0257:Pemetrexed Interaction | -12.67282887 | 13.49575156 | -0.939023574 | 0.34772917 |
| MeWo:Bleomycin.Sulfate Interaction | -6.58252694 | 13.49575156 | -0.487748082 | 0.625733254 |
| SKMEL2:Bleomycin.Sulfate Interaction | -33.13766508 | 13.49575156 | -2.455414575 | 0.014080063 |
| UACC0257:Bleomycin.Sulfate Interaction | -4.482863705 | 13.49575156 | -0.332168511 | 0.739765252 |
| MeWo:Axitinib.1 Interaction | -13.48721996 | 13.49575156 | -0.99936783 | 0.317627764 |
| SKMEL2:Axitinib.1 Interaction | -42.70664491 | 13.49575156 | -3.164451028 | 0.001555919 |
| UACC0257:Axitinib.1 Interaction | -1.905890805 | 13.49575156 | -0.141221539 | 0.887696252 |
| MeWo:Ibrutinib..PCI.32765. Interaction | -6.191656623 | 13.49575156 | -0.458785611 | 0.646392758 |
| SKMEL2:Ibrutinib..PCI.32765. Interaction | -36.00716952 | 13.49575156 | -2.668037371 | 0.007635287 |
| UACC0257:Ibrutinib..PCI.32765. Interaction | -6.751567921 | 13.49575156 | -0.500273578 | 0.616887567 |
| MeWo:Tamoxifen.Citrate Interaction | -7.10829659 | 13.49575156 | -0.526706242 | 0.598403024 |
| SKMEL2:Tamoxifen.Citrate Interaction | -21.02701818 | 13.49575156 | -1.558047218 | 0.119236747 |
| UACC0257:Tamoxifen.Citrate Interaction | -1.479126688 | 13.49575156 | -0.109599431 | 0.912728083 |
| MeWo:Vemurafenib Interaction | -1.683890995 | 13.49575156 | -0.124771932 | 0.900705273 |
| SKMEL2:Vemurafenib Interaction | -17.13372784 | 13.49575156 | -1.269564556 | 0.204253498 |
| UACC0257:Vemurafenib Interaction | -18.37975711 | 13.49575156 | -1.361892076 | 0.17324619 |
| MeWo:Pazopanib.HCl Interaction | -7.985187323 | 13.49575156 | -0.591681559 | 0.554070057 |
| SKMEL2:Pazopanib.HCl Interaction | -23.12940246 | 13.49575156 | -1.713828411 | 0.086574631 |
| UACC0257:Pazopanib.HCl Interaction | -16.28782865 | 13.49575156 | -1.206885632 | 0.227489445 |
| MeWo:Abiraterone Interaction | -8.57907594 | 13.49575156 | -0.635687157 | 0.524987103 |
| SKMEL2:Abiraterone Interaction | -23.32978998 | 13.49575156 | -1.728676604 | 0.08388132 |
| UACC0257:Abiraterone Interaction | -4.553867705 | 13.49575156 | -0.337429722 | 0.73579627 |
| MeWo:Bosutinib..SKI.606. Interaction | -13.31426604 | 13.49575156 | -0.986552396 | 0.323873198 |
| SKMEL2:Bosutinib..SKI.606. Interaction | -34.13362544 | 13.49575156 | -2.52921264 | 0.011438948 |
| UACC0257:Bosutinib..SKI.606. Interaction | -6.898415405 | 13.49575156 | -0.511154593 | 0.60924805 |
| MeWo:Sabutoclax..BI.97C1. Interaction | -11.44533159 | 13.49575156 | -0.848069226 | 0.396408842 |
| SKMEL2:Sabutoclax..BI.97C1. Interaction | -23.71778673 | 13.49575156 | -1.757426152 | 0.078859403 |
| UACC0257:Sabutoclax..BI.97C1. Interaction | -15.13985472 | 13.49575156 | -1.121823757 | 0.261949888 |
| MeWo:Ldose: -1.397940009 (uM) Interaction | -41.01359312 | 13.49575156 | -3.039000305 | 0.002376492 |
| SKMEL2:Ldose: -1.397940009 (uM) Interaction | -27.75707544 | 13.49575156 | -2.056726912 | 0.039724518 |
| UACC0257:Ldose: -1.397940009 (uM) Interaction | -5.815730317 | 13.49575156 | -0.430930452 | 0.666523253 |
| MeWo:Ldose: -1 (uM) Interaction | -10.74352794 | 13.49575156 | -0.796067406 | 0.426001631 |
| SKMEL2:Ldose: -1 (uM) Interaction | 16.29341091 | 13.49575156 | 1.207299263 | 0.227330171 |
| UACC0257:Ldose: -1 (uM) Interaction | 43.67543413 | 13.49575156 | 3.236235783 | 0.001213002 |
| MeWo:Ldose: -0.698970004 (uM) Interaction | 58.71770155 | 13.49575156 | 4.350828576 | 1.36E-05 |
| SKMEL2:Ldose: -0.698970004 (uM) Interaction | 65.33079366 | 13.49575156 | 4.840841456 | 1.30E-06 |
| UACC0257:Ldose: -0.698970004 (uM) Interaction | 61.65920093 | 13.49575156 | 4.568786009 | 4.93E-06 |
| MeWo:Ldose: -0.397940009 (uM) Interaction | 21.26853089 | 13.49575156 | 1.57594268 | 0.115053699 |
| SKMEL2:Ldose: -0.397940009 (uM) Interaction | 28.18902839 | 13.49575156 | 2.0887335 | 0.036743429 |
| UACC0257:Ldose: -0.397940009 (uM) Interaction | 52.16479855 | 13.49575156 | 3.86527555 | 0.000111289 |
| MeWo:Ldose: 0 (uM) Interaction | 10.28018895 | 13.49575156 | 0.761735195 | 0.446226405 |
| SKMEL2:Ldose: 0 (uM) Interaction | 16.73703972 | 13.49575156 | 1.240171 | 0.214925661 |
| UACC0257:Ldose: 0 (uM) Interaction | 25.53474219 | 13.49575156 | 1.892057813 | 0.058496677 |
| MeWo:Ldose: 0.301029996 (uM) Interaction | 51.81723207 | 13.49575156 | 3.83952178 | 0.000123627 |
| SKMEL2:Ldose: 0.301029996 (uM) Interaction | 36.92144581 | 13.49575156 | 2.73578286 | 0.006228303 |
| UACC0257:Ldose: 0.301029996 (uM) Interaction | 18.60295017 | 13.49575156 | 1.378430099 | 0.168084852 |
| Thioguanine:Ldose: -1.397940009 (uM) Interaction | 4.685465172 | 13.49575156 | 0.347180752 | 0.728458922 |
| Irinotecan.HCl:Ldose: -1.397940009 (uM) Interaction | 3.025530714 | 13.49575156 | 0.224183937 | 0.822616308 |
| Romidepsin:Ldose: -1.397940009 (uM) Interaction | -0.228923871 | 13.49575156 | -0.016962662 | 0.98646656 |
| Paclitaxel:Ldose: -1.397940009 (uM) Interaction | -8.9059273 | 13.49575156 | -0.65990599 | 0.509321216 |
| Alisertib..MLN8237.:Ldose: -1.397940009 (uM) Interaction | -0.439016368 | 13.49575156 | -0.032529968 | 0.974049719 |
| Vorinostat:Ldose: -1.397940009 (uM) Interaction | 11.97494473 | 13.49575156 | 0.887312179 | 0.374920757 |
| Busulfan:Ldose: -1.397940009 (uM) Interaction | 3.99107383 | 13.49575156 | 0.295728164 | 0.767440538 |
| Mechlorethamine.HCl:Ldose: -1.397940009 (uM) Interaction | 10.04675266 | 13.49575156 | 0.744438175 | 0.456619552 |
| Teniposide:Ldose: -1.397940009 (uM) Interaction | -0.632734186 | 13.49575156 | -0.046883953 | 0.962606152 |
| Vinorelbine.Tartrate:Ldose: -1.397940009 (uM) Interaction | 6.614781822 | 13.49575156 | 0.490138085 | 0.624041182 |
| Cabozantinib..XL.184.:Ldose: -1.397940009 (uM) Interaction | 3.131451397 | 13.49575156 | 0.232032383 | 0.816515066 |
| Dacarbazine:Ldose: -1.397940009 (uM) Interaction | -1.609421189 | 13.49575156 | -0.119253913 | 0.905075307 |
| Clofarabine:Ldose: -1.397940009 (uM) Interaction | -19.14210677 | 13.49575156 | -1.418380197 | 0.156094261 |
| Cisplatin:Ldose: -1.397940009 (uM) Interaction | 0.351907747 | 13.49575156 | 0.026075446 | 0.979197403 |
| Floxuridine:Ldose: -1.397940009 (uM) Interaction | -11.76099161 | 13.49575156 | -0.871458811 | 0.383513399 |
| Lomustine..CCNU.:Ldose: -1.397940009 (uM) Interaction | 11.16588215 | 13.49575156 | 0.827362751 | 0.408040652 |
| Melphalan:Ldose: -1.397940009 (uM) Interaction | 4.84023113 | 13.49575156 | 0.358648506 | 0.71986157 |
| BGJ398..NVPBGJ398.:Ldose: -1.397940009 (uM) Interaction | 2.349357104 | 13.49575156 | 0.174081235 | 0.861803256 |
| Navitoclax..ABT.263..5uM:Ldose: -1.397940009 (uM) Interaction | 6.190600247 | 13.49575156 | 0.458707336 | 0.646448973 |
| Azacitidine:Ldose: -1.397940009 (uM) Interaction | 11.35628216 | 13.49575156 | 0.841470896 | 0.400093524 |
| Capecitabine:Ldose: -1.397940009 (uM) Interaction | 8.401604077 | 13.49575156 | 0.622536954 | 0.533595442 |
| Megestrol.acetate:Ldose: -1.397940009 (uM) Interaction | 6.269388216 | 13.49575156 | 0.464545319 | 0.642261794 |
| Cytarabine.HCl...Ara.C:Ldose: -1.397940009 (uM) Interaction | 3.886674039 | 13.49575156 | 0.287992412 | 0.773355318 |
| Gemcitabine.HCl:Ldose: -1.397940009 (uM) Interaction | 7.529340799 | 13.49575156 | 0.55790452 | 0.576915383 |
| Vinblastine.Sulfate:Ldose: -1.397940009 (uM) Interaction | 0.20195553 | 13.49575156 | 0.014964378 | 0.988060738 |
| MLN9708..MLN2238.:Ldose: -1.397940009 (uM) Interaction | 18.87080416 | 13.49575156 | 1.398277382 | 0.162044183 |
| ABT.737:Ldose: -1.397940009 (uM) Interaction | 14.12103725 | 13.49575156 | 1.046332039 | 0.295419499 |
| Streptozocin:Ldose: -1.397940009 (uM) Interaction | 7.04337043 | 13.49575156 | 0.521895383 | 0.60174854 |
| Crizotinib:Ldose: -1.397940009 (uM) Interaction | 6.83123511 | 13.49575156 | 0.506176709 | 0.612737784 |
| Sunitinib:Ldose: -1.397940009 (uM) Interaction | 19.01329183 | 13.49575156 | 1.408835346 | 0.158898296 |
| Dexrazoxane:Ldose: -1.397940009 (uM) Interaction | 15.93843232 | 13.49575156 | 1.180996275 | 0.237617243 |
| Mitomycin.C:Ldose: -1.397940009 (uM) Interaction | 17.59269047 | 13.49575156 | 1.30357249 | 0.192393318 |
| Carfilzomib:Ldose: -1.397940009 (uM) Interaction | -26.84623906 | 13.49575156 | -1.989236311 | 0.046687752 |
| OSI.027:Ldose: -1.397940009 (uM) Interaction | 21.87493913 | 13.49575156 | 1.62087595 | 0.105058883 |
| Bioymifi:Ldose: -1.397940009 (uM) Interaction | 11.40595121 | 13.49575156 | 0.845151243 | 0.398035788 |
| Nelarabine:Ldose: -1.397940009 (uM) Interaction | 9.94261763 | 13.49575156 | 0.736722041 | 0.461299424 |
| Raloxifene:Ldose: -1.397940009 (uM) Interaction | 3.866434827 | 13.49575156 | 0.286492739 | 0.77450351 |
| Quinacrine.HCl:Ldose: -1.397940009 (uM) Interaction | 14.08163053 | 13.49575156 | 1.043412104 | 0.29676918 |
| Lenalidomide:Ldose: -1.397940009 (uM) Interaction | 16.7185888 | 13.49575156 | 1.238803836 | 0.215431652 |
| Fludarabine.Phosphate:Ldose: -1.397940009 (uM) Interaction | 19.08374172 | 13.49575156 | 1.414055499 | 0.157360061 |
| Nilotinib:Ldose: -1.397940009 (uM) Interaction | 15.53719483 | 13.49575156 | 1.151265623 | 0.249635754 |
| Linsitinib:Ldose: -1.397940009 (uM) Interaction | 11.45301078 | 13.49575156 | 0.848638234 | 0.396092055 |
| Aphrocallistin.analogue:Ldose: -1.397940009 (uM) Interaction | 0.24581884 | 13.49575156 | 0.018214535 | 0.985467876 |
| Mitotane..o.p..DDD..Lysodren.:Ldose: -1.397940009 (uM) Interaction | 4.577277404 | 13.49575156 | 0.33916432 | 0.734489253 |
| Etoposide:Ldose: -1.397940009 (uM) Interaction | 1.335296232 | 13.49575156 | 0.098941969 | 0.921185267 |
| Vandetanib:Ldose: -1.397940009 (uM) Interaction | 16.54209395 | 13.49575156 | 1.225726027 | 0.220315193 |
| Carboplatin:Ldose: -1.397940009 (uM) Interaction | 12.85787917 | 13.49575156 | 0.952735319 | 0.340734884 |
| Gefitinib:Ldose: -1.397940009 (uM) Interaction | 15.17190001 | 13.49575156 | 1.124198229 | 0.260941469 |
| Vincristine.Sulfate:Ldose: -1.397940009 (uM) Interaction | -16.53351972 | 13.49575156 | -1.225090699 | 0.220554445 |
| Trametinib..GSK1120212.:Ldose: -1.397940009 (uM) Interaction | 5.615667369 | 13.49575156 | 0.416106309 | 0.677336359 |
| MLN4924:Ldose: -1.397940009 (uM) Interaction | 1.77232648 | 13.49575156 | 0.131324771 | 0.895519624 |
| Bortezomib:Ldose: -1.397940009 (uM) Interaction | -12.44203215 | 13.49575156 | -0.921922139 | 0.356579516 |
| Fluorouracil...5.FU.:Ldose: -1.397940009 (uM) Interaction | 6.241698382 | 13.49575156 | 0.462493575 | 0.643732081 |
| Lapatinib:Ldose: -1.397940009 (uM) Interaction | 17.51346101 | 13.49575156 | 1.297701793 | 0.194403724 |
| Mitoxantrone:Ldose: -1.397940009 (uM) Interaction | -22.52973206 | 13.49575156 | -1.669394399 | 0.095053775 |
| Imatinib:Ldose: -1.397940009 (uM) Interaction | 14.26161137 | 13.49575156 | 1.056748214 | 0.290638366 |
| Imiquimod:Ldose: -1.397940009 (uM) Interaction | 13.4002541 | 13.49575156 | 0.992923887 | 0.320758202 |
| Dacomitinib..PF299804.:Ldose: -1.397940009 (uM) Interaction | 12.73006915 | 13.49575156 | 0.94326493 | 0.345555989 |
| PD325901:Ldose: -1.397940009 (uM) Interaction | -1.96333717 | 13.49575156 | -0.145478165 | 0.884334716 |
| Vismodegib:Ldose: -1.397940009 (uM) Interaction | 7.283579614 | 13.49575156 | 0.539694257 | 0.58941348 |
| Temozolomide:Ldose: -1.397940009 (uM) Interaction | 9.889713392 | 13.49575156 | 0.732801974 | 0.463687191 |
| Mercaptopurine:Ldose: -1.397940009 (uM) Interaction | 6.019180867 | 13.49575156 | 0.446005607 | 0.655597679 |
| Dasatinib:Ldose: -1.397940009 (uM) Interaction | 15.07834341 | 13.49575156 | 1.117265929 | 0.2638931 |
| Daunorubicin.HCl:Ldose: -1.397940009 (uM) Interaction | -8.384286035 | 13.49575156 | -0.621253733 | 0.534439265 |
| Sirolimus..Rapamycin.:Ldose: -1.397940009 (uM) Interaction | 11.52270797 | 13.49575156 | 0.853802614 | 0.393223861 |
| INK.128..MLN0128.:Ldose: -1.397940009 (uM) Interaction | -7.779988636 | 13.49575156 | -0.576476871 | 0.564298922 |
| Quizartinib:Ldose: -1.397940009 (uM) Interaction | 10.05506495 | 13.49575156 | 0.745054093 | 0.456247148 |
| Sorafenib:Ldose: -1.397940009 (uM) Interaction | 5.172403717 | 13.49575156 | 0.383261628 | 0.70152957 |
| Carmustine:Ldose: -1.397940009 (uM) Interaction | 6.39401223 | 13.49575156 | 0.473779634 | 0.635661861 |
| Uracil.mustard:Ldose: -1.397940009 (uM) Interaction | 3.184341247 | 13.49575156 | 0.235951383 | 0.813472639 |
| Ixabepilone:Ldose: -1.397940009 (uM) Interaction | -11.63536907 | 13.49575156 | -0.862150508 | 0.388614298 |
| Valrubicin:Ldose: -1.397940009 (uM) Interaction | 12.89039173 | 13.49575156 | 0.955144415 | 0.33951539 |
| Triethylenemelamine:Ldose: -1.397940009 (uM) Interaction | 9.704530597 | 13.49575156 | 0.719080412 | 0.472099176 |
| Palbociclib..PD.0332991..Isethionate:Ldose: -1.397940009 (uM) Interaction | 7.57680522 | 13.49575156 | 0.56142151 | 0.574516065 |
| Afatinib:Ldose: -1.397940009 (uM) Interaction | -1.555691703 | 13.49575156 | -0.115272699 | 0.908230052 |
| Doxorubicin.HCl:Ldose: -1.397940009 (uM) Interaction | -3.877754666 | 13.49575156 | -0.287331509 | 0.773861262 |
| Exemestane:Ldose: -1.397940009 (uM) Interaction | 5.812550277 | 13.49575156 | 0.430694819 | 0.666694596 |
| Tretinoin:Ldose: -1.397940009 (uM) Interaction | 6.108103089 | 13.49575156 | 0.452594512 | 0.65084531 |
| Fulvestrant:Ldose: -1.397940009 (uM) Interaction | 14.46610253 | 13.49575156 | 1.071900476 | 0.283776746 |
| Docetaxel:Ldose: -1.397940009 (uM) Interaction | 2.053551117 | 13.49575156 | 0.152162783 | 0.879059972 |
| Everolimus:Ldose: -1.397940009 (uM) Interaction | 8.944463845 | 13.49575156 | 0.662761448 | 0.50749044 |
| MLN.2480:Ldose: -1.397940009 (uM) Interaction | 6.755437369 | 13.49575156 | 0.500560294 | 0.616685727 |
| LY2157299:Ldose: -1.397940009 (uM) Interaction | 5.74822603 | 13.49575156 | 0.42592856 | 0.670164159 |
| Allopurinol:Ldose: -1.397940009 (uM) Interaction | 5.54602108 | 13.49575156 | 0.410945701 | 0.681116439 |
| Pipobroman:Ldose: -1.397940009 (uM) Interaction | 3.99765099 | 13.49575156 | 0.296215514 | 0.767068357 |
| Letrozole:Ldose: -1.397940009 (uM) Interaction | 4.445755544 | 13.49575156 | 0.329418893 | 0.741842292 |
| Thiotepa:Ldose: -1.397940009 (uM) Interaction | 17.42825463 | 13.49575156 | 1.291388223 | 0.19658295 |
| Plicamycin:Ldose: -1.397940009 (uM) Interaction | -4.302759428 | 13.49575156 | -0.318823254 | 0.749863635 |
| Erlotinib.HCl:Ldose: -1.397940009 (uM) Interaction | 15.84546949 | 13.49575156 | 1.174107971 | 0.240364762 |
| MEK.162..ARRY.438162.:Ldose: -1.397940009 (uM) Interaction | -10.84912251 | 13.49575156 | -0.803891688 | 0.421468402 |
| Baricitinib..LY3009104..INCB028050.:Ldose: -1.397940009 (uM) Interaction | -0.154068386 | 13.49575156 | -0.011416066 | 0.990891601 |
| Arsenic.Trioxide:Ldose: -1.397940009 (uM) Interaction | -3.19288922 | 13.49575156 | -0.236584766 | 0.81298119 |
| Celecoxib:Ldose: -1.397940009 (uM) Interaction | 0.49159983 | 13.49575156 | 0.036426266 | 0.970942809 |
| Bendamustine.HCl:Ldose: -1.397940009 (uM) Interaction | 3.736322937 | 13.49575156 | 0.276851787 | 0.781896571 |
| Chlorambucil:Ldose: -1.397940009 (uM) Interaction | 11.302404 | 13.49575156 | 0.837478665 | 0.402332857 |
| Zoledronic.Acid:Ldose: -1.397940009 (uM) Interaction | 10.1908346 | 13.49575156 | 0.755114271 | 0.450188678 |
| Actinomycin.D:Ldose: -1.397940009 (uM) Interaction | -8.930571465 | 13.49575156 | -0.661732059 | 0.508150034 |
| Temsirolimus..CCI.779..Torisel.:Ldose: -1.397940009 (uM) Interaction | 3.728541027 | 13.49575156 | 0.276275168 | 0.782339378 |
| Foretinib..GSK1363089.:Ldose: -1.397940009 (uM) Interaction | 3.054731647 | 13.49575156 | 0.22634765 | 0.820933189 |
| Decitabine:Ldose: -1.397940009 (uM) Interaction | 7.664993099 | 13.49575156 | 0.567956002 | 0.570070764 |
| Methotrexate:Ldose: -1.397940009 (uM) Interaction | -24.40795195 | 13.49575156 | -1.808565595 | 0.070532456 |
| Axitinib:Ldose: -1.397940009 (uM) Interaction | 2.235984437 | 13.49575156 | 0.165680616 | 0.868409856 |
| Oxaliplatin:Ldose: -1.397940009 (uM) Interaction | 4.769290647 | 13.49575156 | 0.353392001 | 0.72379804 |
| Cabazitaxel:Ldose: -1.397940009 (uM) Interaction | 5.222654897 | 13.49575156 | 0.38698511 | 0.698771055 |
| Amifostine:Ldose: -1.397940009 (uM) Interaction | 7.89470023 | 13.49575156 | 0.584976701 | 0.55856951 |
| Flutamide..Eulexin.:Ldose: -1.397940009 (uM) Interaction | 3.63591833 | 13.49575156 | 0.269412067 | 0.787615181 |
| LDK378:Ldose: -1.397940009 (uM) Interaction | -1.834765353 | 13.49575156 | -0.135951328 | 0.891861028 |
| Pralatrexate:Ldose: -1.397940009 (uM) Interaction | 4.506211361 | 13.49575156 | 0.333898512 | 0.738459393 |
| Topotecan.HCl:Ldose: -1.397940009 (uM) Interaction | -13.41368748 | 13.49575156 | -0.993919265 | 0.320273339 |
| Pemetrexed:Ldose: -1.397940009 (uM) Interaction | 3.445176564 | 13.49575156 | 0.2552786 | 0.798510365 |
| Bleomycin.Sulfate:Ldose: -1.397940009 (uM) Interaction | 4.557561247 | 13.49575156 | 0.337703404 | 0.73559 |
| Axitinib.1:Ldose: -1.397940009 (uM) Interaction | 1.71643858 | 13.49575156 | 0.127183623 | 0.898796254 |
| Ibrutinib..PCI.32765.:Ldose: -1.397940009 (uM) Interaction | 3.780470297 | 13.49575156 | 0.280122991 | 0.779385836 |
| Tamoxifen.Citrate:Ldose: -1.397940009 (uM) Interaction | 1.990963514 | 13.49575156 | 0.147525205 | 0.882718868 |
| Vemurafenib:Ldose: -1.397940009 (uM) Interaction | -4.547319791 | 13.49575156 | -0.336944539 | 0.736161992 |
| Pazopanib.HCl:Ldose: -1.397940009 (uM) Interaction | 6.442178397 | 13.49575156 | 0.477348621 | 0.633118737 |
| Abiraterone:Ldose: -1.397940009 (uM) Interaction | 5.770013114 | 13.49575156 | 0.427542926 | 0.6689882 |
| Bosutinib..SKI.606.:Ldose: -1.397940009 (uM) Interaction | -2.315638953 | 13.49575156 | -0.171582808 | 0.863767136 |
| Sabutoclax..BI.97C1.:Ldose: -1.397940009 (uM) Interaction | 2.285615627 | 13.49575156 | 0.169358158 | 0.865516523 |
| Thioguanine:Ldose: -1 (uM) Interaction | 38.26806601 | 13.49575156 | 2.835563905 | 0.004578748 |
| Irinotecan.HCl:Ldose: -1 (uM) Interaction | 37.05964658 | 13.49575156 | 2.746023177 | 0.006037256 |
| Romidepsin:Ldose: -1 (uM) Interaction | 31.31265301 | 13.49575156 | 2.320185939 | 0.020340116 |
| Paclitaxel:Ldose: -1 (uM) Interaction | 22.70362277 | 13.49575156 | 1.682279247 | 0.092529198 |
| Alisertib..MLN8237.:Ldose: -1 (uM) Interaction | 28.71084363 | 13.49575156 | 2.127398648 | 0.033398302 |
| Vorinostat:Ldose: -1 (uM) Interaction | 42.7031488 | 13.49575156 | 3.164191975 | 0.001557304 |
| Busulfan:Ldose: -1 (uM) Interaction | 36.4990789 | 13.49575156 | 2.704486573 | 0.006846378 |
| Mechlorethamine.HCl:Ldose: -1 (uM) Interaction | 45.46292236 | 13.49575156 | 3.368684 | 0.000756603 |
| Teniposide:Ldose: -1 (uM) Interaction | 27.54908707 | 13.49575156 | 2.041315516 | 0.041231636 |
| Vinorelbine.Tartrate:Ldose: -1 (uM) Interaction | 36.81175426 | 13.49575156 | 2.727655002 | 0.006383795 |
| Cabozantinib..XL.184.:Ldose: -1 (uM) Interaction | 35.1050669 | 13.49575156 | 2.601193919 | 0.009296343 |
| Dacarbazine:Ldose: -1 (uM) Interaction | 40.66750746 | 13.49575156 | 3.01335626 | 0.002586757 |
| Clofarabine:Ldose: -1 (uM) Interaction | -26.57061997 | 13.49575156 | -1.968813655 | 0.048987294 |
| Cisplatin:Ldose: -1 (uM) Interaction | 35.51346753 | 13.49575156 | 2.631455341 | 0.008508061 |
| Floxuridine:Ldose: -1 (uM) Interaction | 6.960383029 | 13.49575156 | 0.515746233 | 0.606036953 |
| Lomustine..CCNU.:Ldose: -1 (uM) Interaction | 43.66406855 | 13.49575156 | 3.235393623 | 0.001216584 |
| Melphalan:Ldose: -1 (uM) Interaction | 37.10844062 | 13.49575156 | 2.749638687 | 0.005971075 |
| BGJ398..NVPBGJ398.:Ldose: -1 (uM) Interaction | 37.72733567 | 13.49575156 | 2.795497197 | 0.005186581 |
| Navitoclax..ABT.263..5uM:Ldose: -1 (uM) Interaction | 40.71147666 | 13.49575156 | 3.016614263 | 0.002559131 |
| Azacitidine:Ldose: -1 (uM) Interaction | 44.56766902 | 13.49575156 | 3.302348062 | 0.000960351 |
| Capecitabine:Ldose: -1 (uM) Interaction | 37.82281311 | 13.49575156 | 2.802571827 | 0.00507422 |
| Megestrol.acetate:Ldose: -1 (uM) Interaction | 36.91264607 | 13.49575156 | 2.735130822 | 0.00624065 |
| Cytarabine.HCl...Ara.C:Ldose: -1 (uM) Interaction | 22.71970141 | 13.49575156 | 1.683470631 | 0.092298511 |
| Gemcitabine.HCl:Ldose: -1 (uM) Interaction | 43.71402652 | 13.49575156 | 3.239095378 | 0.001200912 |
| Vinblastine.Sulfate:Ldose: -1 (uM) Interaction | 35.46751207 | 13.49575156 | 2.628050161 | 0.008593674 |
| MLN9708..MLN2238.:Ldose: -1 (uM) Interaction | 35.74980471 | 13.49575156 | 2.648967309 | 0.008079703 |
| ABT.737:Ldose: -1 (uM) Interaction | 46.44075765 | 13.49575156 | 3.441139045 | 0.000580369 |
| Streptozocin:Ldose: -1 (uM) Interaction | 43.99000987 | 13.49575156 | 3.259545025 | 0.001117642 |
| Crizotinib:Ldose: -1 (uM) Interaction | 39.46597451 | 13.49575156 | 2.924325802 | 0.003455629 |
| Sunitinib:Ldose: -1 (uM) Interaction | 49.8132446 | 13.49575156 | 3.691031534 | 0.000223897 |
| Dexrazoxane:Ldose: -1 (uM) Interaction | 45.41379331 | 13.49575156 | 3.365043665 | 0.000766651 |
| Mitomycin.C:Ldose: -1 (uM) Interaction | 40.38736222 | 13.49575156 | 2.992598228 | 0.002769271 |
| Carfilzomib:Ldose: -1 (uM) Interaction | -10.22094797 | 13.49575156 | -0.757345593 | 0.448851127 |
| OSI.027:Ldose: -1 (uM) Interaction | 51.7363846 | 13.49575156 | 3.833531193 | 0.000126676 |
| Bioymifi:Ldose: -1 (uM) Interaction | 47.14716421 | 13.49575156 | 3.493481929 | 0.000477722 |
| Nelarabine:Ldose: -1 (uM) Interaction | 46.6972021 | 13.49575156 | 3.46014091 | 0.000540938 |
| Raloxifene:Ldose: -1 (uM) Interaction | 39.5374016 | 13.49575156 | 2.929618362 | 0.003397344 |
| Quinacrine.HCl:Ldose: -1 (uM) Interaction | 46.32025115 | 13.49575156 | 3.432209828 | 0.000599809 |
| Lenalidomide:Ldose: -1 (uM) Interaction | 48.53900389 | 13.49575156 | 3.59661362 | 0.000323108 |
| Fludarabine.Phosphate:Ldose: -1 (uM) Interaction | 48.73682167 | 13.49575156 | 3.611271402 | 0.000305392 |
| Nilotinib:Ldose: -1 (uM) Interaction | 47.6772069 | 13.49575156 | 3.532756711 | 0.000412111 |
| Linsitinib:Ldose: -1 (uM) Interaction | 41.02612927 | 13.49575156 | 3.039929201 | 0.002369178 |
| Aphrocallistin.analogue:Ldose: -1 (uM) Interaction | 23.99489866 | 13.49575156 | 1.777959423 | 0.075424623 |
| Mitotane..o.p..DDD..Lysodren.:Ldose: -1 (uM) Interaction | 37.48567465 | 13.49575156 | 2.777590746 | 0.00548109 |
| Etoposide:Ldose: -1 (uM) Interaction | 32.8137552 | 13.49575156 | 2.431413697 | 0.015048129 |
| Vandetanib:Ldose: -1 (uM) Interaction | 51.5481683 | 13.49575156 | 3.819584857 | 0.000134051 |
| Carboplatin:Ldose: -1 (uM) Interaction | 44.92987805 | 13.49575156 | 3.329186807 | 0.000872461 |
| Gefitinib:Ldose: -1 (uM) Interaction | 42.05977123 | 13.49575156 | 3.116519375 | 0.001832402 |
| Vincristine.Sulfate:Ldose: -1 (uM) Interaction | 9.270378084 | 13.49575156 | 0.686910843 | 0.492146309 |
| Trametinib..GSK1120212.:Ldose: -1 (uM) Interaction | 42.37283379 | 13.49575156 | 3.139716494 | 0.001693399 |
| MLN4924:Ldose: -1 (uM) Interaction | 37.79047513 | 13.49575156 | 2.800175667 | 0.005112028 |
| Bortezomib:Ldose: -1 (uM) Interaction | 23.42175918 | 13.49575156 | 1.735491282 | 0.082668126 |
| Fluorouracil...5.FU.:Ldose: -1 (uM) Interaction | 40.41165392 | 13.49575156 | 2.99439818 | 0.002752991 |
| Lapatinib:Ldose: -1 (uM) Interaction | 42.09454136 | 13.49575156 | 3.119095751 | 0.001816462 |
| Mitoxantrone:Ldose: -1 (uM) Interaction | 22.13148004 | 13.49575156 | 1.639884962 | 0.101043691 |
| Imatinib:Ldose: -1 (uM) Interaction | 46.63795493 | 13.49575156 | 3.45575085 | 0.00054982 |
| Imiquimod:Ldose: -1 (uM) Interaction | 47.11528262 | 13.49575156 | 3.491119586 | 0.000481964 |
| Dacomitinib..PF299804.:Ldose: -1 (uM) Interaction | 44.44678847 | 13.49575156 | 3.293391128 | 0.000991462 |
| PD325901:Ldose: -1 (uM) Interaction | 32.33170622 | 13.49575156 | 2.395695124 | 0.016597363 |
| Vismodegib:Ldose: -1 (uM) Interaction | 38.94938245 | 13.49575156 | 2.886047677 | 0.003905005 |
| Temozolomide:Ldose: -1 (uM) Interaction | 40.75043253 | 13.49575156 | 3.019500791 | 0.002534881 |
| Mercaptopurine:Ldose: -1 (uM) Interaction | 36.27608115 | 13.49575156 | 2.687963022 | 0.007194461 |
| Dasatinib:Ldose: -1 (uM) Interaction | 44.97119352 | 13.49575156 | 3.332248175 | 0.000862924 |
| Daunorubicin.HCl:Ldose: -1 (uM) Interaction | 7.058685757 | 13.49575156 | 0.523030209 | 0.600958611 |
| Sirolimus..Rapamycin.:Ldose: -1 (uM) Interaction | 42.76245371 | 13.49575156 | 3.168586314 | 0.001533963 |
| INK.128..MLN0128.:Ldose: -1 (uM) Interaction | 6.73699024 | 13.49575156 | 0.49919341 | 0.617648235 |
| Quizartinib:Ldose: -1 (uM) Interaction | 42.96827138 | 13.49575156 | 3.183836867 | 0.001455436 |
| Sorafenib:Ldose: -1 (uM) Interaction | 44.41127499 | 13.49575156 | 3.290759672 | 0.001000778 |
| Carmustine:Ldose: -1 (uM) Interaction | 39.2394577 | 13.49575156 | 2.907541497 | 0.003646537 |
| Uracil.mustard:Ldose: -1 (uM) Interaction | 37.7136682 | 13.49575156 | 2.794484473 | 0.005202848 |
| Ixabepilone:Ldose: -1 (uM) Interaction | 8.998514882 | 13.49575156 | 0.666766489 | 0.504928443 |
| Valrubicin:Ldose: -1 (uM) Interaction | 36.2077858 | 13.49575156 | 2.682902514 | 0.007304198 |
| Triethylenemelamine:Ldose: -1 (uM) Interaction | 42.48517825 | 13.49575156 | 3.148040926 | 0.001645929 |
| Palbociclib..PD.0332991..Isethionate:Ldose: -1 (uM) Interaction | 40.4722865 | 13.49575156 | 2.998890896 | 0.002712737 |
| Afatinib:Ldose: -1 (uM) Interaction | 33.75115241 | 13.49575156 | 2.500872386 | 0.012396148 |
| Doxorubicin.HCl:Ldose: -1 (uM) Interaction | 20.32041282 | 13.49575156 | 1.505689604 | 0.132161537 |
| Exemestane:Ldose: -1 (uM) Interaction | 36.72381688 | 13.49575156 | 2.721139071 | 0.006510964 |
| Tretinoin:Ldose: -1 (uM) Interaction | 37.08570574 | 13.49575156 | 2.747954092 | 0.00600183 |
| Fulvestrant:Ldose: -1 (uM) Interaction | 46.9647697 | 13.49575156 | 3.479966972 | 0.000502467 |
| Docetaxel:Ldose: -1 (uM) Interaction | 32.82075861 | 13.49575156 | 2.431932631 | 0.015026593 |
| Everolimus:Ldose: -1 (uM) Interaction | 39.75934893 | 13.49575156 | 2.946064081 | 0.003221902 |
| MLN.2480:Ldose: -1 (uM) Interaction | 40.09444684 | 13.49575156 | 2.970893962 | 0.00297262 |
| LY2157299:Ldose: -1 (uM) Interaction | 41.62582802 | 13.49575156 | 3.084365314 | 0.002042451 |
| Allopurinol:Ldose: -1 (uM) Interaction | 46.13413732 | 13.49575156 | 3.418419278 | 0.000631026 |
| Pipobroman:Ldose: -1 (uM) Interaction | 34.90179461 | 13.49575156 | 2.586131972 | 0.009712475 |
| Letrozole:Ldose: -1 (uM) Interaction | 33.9071469 | 13.49575156 | 2.51243117 | 0.011997496 |
| Thiotepa:Ldose: -1 (uM) Interaction | 47.64978626 | 13.49575156 | 3.530724913 | 0.000415288 |
| Plicamycin:Ldose: -1 (uM) Interaction | -11.26886669 | 13.49575156 | -0.834993637 | 0.403730552 |
| Erlotinib.HCl:Ldose: -1 (uM) Interaction | 45.61161033 | 13.49575156 | 3.37970139 | 0.000726933 |
| MEK.162..ARRY.438162.:Ldose: -1 (uM) Interaction | 3.11021527 | 13.49575156 | 0.230458841 | 0.817737431 |
| Baricitinib..LY3009104..INCB028050.:Ldose: -1 (uM) Interaction | 37.11921267 | 13.49575156 | 2.750436868 | 0.005956553 |
| Arsenic.Trioxide:Ldose: -1 (uM) Interaction | 34.57373182 | 13.49575156 | 2.561823375 | 0.010419168 |
| Celecoxib:Ldose: -1 (uM) Interaction | 33.18466437 | 13.49575156 | 2.458897099 | 0.013944262 |
| Bendamustine.HCl:Ldose: -1 (uM) Interaction | 35.55272707 | 13.49575156 | 2.63436437 | 0.008435526 |
| Chlorambucil:Ldose: -1 (uM) Interaction | 38.43903705 | 13.49575156 | 2.848232413 | 0.004400443 |
| Zoledronic.Acid:Ldose: -1 (uM) Interaction | 42.14743623 | 13.49575156 | 3.123015123 | 0.001792457 |
| Actinomycin.D:Ldose: -1 (uM) Interaction | 9.71927946 | 13.49575156 | 0.720173264 | 0.471426137 |
| Temsirolimus..CCI.779..Torisel.:Ldose: -1 (uM) Interaction | 36.52291773 | 13.49575156 | 2.706252969 | 0.006810078 |
| Foretinib..GSK1363089.:Ldose: -1 (uM) Interaction | 37.77055532 | 13.49575156 | 2.79869966 | 0.005135443 |
| Decitabine:Ldose: -1 (uM) Interaction | 43.84825683 | 13.49575156 | 3.249041494 | 0.001159721 |
| Methotrexate:Ldose: -1 (uM) Interaction | -3.946626068 | 13.49575156 | -0.292434701 | 0.769957097 |
| Axitinib:Ldose: -1 (uM) Interaction | 37.82411444 | 13.49575156 | 2.802668252 | 0.005072704 |
| Oxaliplatin:Ldose: -1 (uM) Interaction | 38.49320613 | 13.49575156 | 2.852246201 | 0.004345278 |
| Cabazitaxel:Ldose: -1 (uM) Interaction | 36.11775817 | 13.49575156 | 2.676231702 | 0.007451149 |
| Amifostine:Ldose: -1 (uM) Interaction | 42.8686669 | 13.49575156 | 3.176456435 | 0.001492964 |
| Flutamide..Eulexin.:Ldose: -1 (uM) Interaction | 40.08500727 | 13.49575156 | 2.970194515 | 0.002979394 |
| LDK378:Ldose: -1 (uM) Interaction | 33.61589135 | 13.49575156 | 2.490849894 | 0.012751266 |
| Pralatrexate:Ldose: -1 (uM) Interaction | 41.79978805 | 13.49575156 | 3.097255299 | 0.001955726 |
| Topotecan.HCl:Ldose: -1 (uM) Interaction | -4.334656613 | 13.49575156 | -0.321186752 | 0.748071987 |
| Pemetrexed:Ldose: -1 (uM) Interaction | 36.382944 | 13.49575156 | 2.69588128 | 0.007025721 |
| Bleomycin.Sulfate:Ldose: -1 (uM) Interaction | 26.572608 | 13.49575156 | 1.968960963 | 0.048970373 |
| Axitinib.1:Ldose: -1 (uM) Interaction | 34.81689763 | 13.49575156 | 2.579841328 | 0.009891134 |
| Ibrutinib..PCI.32765.:Ldose: -1 (uM) Interaction | 38.71170885 | 13.49575156 | 2.868436683 | 0.004129065 |
| Tamoxifen.Citrate:Ldose: -1 (uM) Interaction | 41.5251832 | 13.49575156 | 3.076907796 | 0.002094224 |
| Vemurafenib:Ldose: -1 (uM) Interaction | 3.306444272 | 13.49575156 | 0.244998899 | 0.806459618 |
| Pazopanib.HCl:Ldose: -1 (uM) Interaction | 43.47872543 | 13.49575156 | 3.221660181 | 0.001276395 |
| Abiraterone:Ldose: -1 (uM) Interaction | 39.56863543 | 13.49575156 | 2.931932708 | 0.00337214 |
| Bosutinib..SKI.606.:Ldose: -1 (uM) Interaction | 29.83632031 | 13.49575156 | 2.210793535 | 0.027060604 |
| Sabutoclax..BI.97C1.:Ldose: -1 (uM) Interaction | 37.9000849 | 13.49575156 | 2.808297465 | 0.004984901 |
| Thioguanine:Ldose: -0.698970004 (uM) Interaction | 62.73894857 | 13.49575156 | 4.648792494 | 3.36E-06 |
| Irinotecan.HCl:Ldose: -0.698970004 (uM) Interaction | 53.51425216 | 13.49575156 | 3.965266543 | 7.36E-05 |
| Romidepsin:Ldose: -0.698970004 (uM) Interaction | 101.3472302 | 13.49575156 | 7.509565493 | 6.16E-14 |
| Paclitaxel:Ldose: -0.698970004 (uM) Interaction | 50.36124342 | 13.49575156 | 3.731636818 | 0.000190728 |
| Alisertib..MLN8237.:Ldose: -0.698970004 (uM) Interaction | 45.74976887 | 13.49575156 | 3.389938578 | 0.000700337 |
| Vorinostat:Ldose: -0.698970004 (uM) Interaction | 59.71119661 | 13.49575156 | 4.424443969 | 9.72E-06 |
| Busulfan:Ldose: -0.698970004 (uM) Interaction | 65.21330787 | 13.49575156 | 4.832136066 | 1.36E-06 |
| Mechlorethamine.HCl:Ldose: -0.698970004 (uM) Interaction | 63.0506285 | 13.49575156 | 4.671887164 | 3.00E-06 |
| Teniposide:Ldose: -0.698970004 (uM) Interaction | 43.9072355 | 13.49575156 | 3.25341166 | 0.001142038 |
| Vinorelbine.Tartrate:Ldose: -0.698970004 (uM) Interaction | 61.03989798 | 13.49575156 | 4.522897276 | 6.13E-06 |
| Cabozantinib..XL.184.:Ldose: -0.698970004 (uM) Interaction | 52.13688747 | 13.49575156 | 3.863207411 | 0.000112235 |
| Dacarbazine:Ldose: -0.698970004 (uM) Interaction | 69.25596729 | 13.49575156 | 5.131686588 | 2.90E-07 |
| Clofarabine:Ldose: -0.698970004 (uM) Interaction | -13.90600905 | 13.49575156 | -1.03039901 | 0.302834307 |
| Cisplatin:Ldose: -0.698970004 (uM) Interaction | 65.57057674 | 13.49575156 | 4.858608758 | 1.19E-06 |
| Floxuridine:Ldose: -0.698970004 (uM) Interaction | 25.36009732 | 13.49575156 | 1.879117084 | 0.060241999 |
| Lomustine..CCNU.:Ldose: -0.698970004 (uM) Interaction | 65.42512024 | 13.49575156 | 4.84783081 | 1.26E-06 |
| Melphalan:Ldose: -0.698970004 (uM) Interaction | 66.01415694 | 13.49575156 | 4.891476894 | 1.01E-06 |
| BGJ398..NVPBGJ398.:Ldose: -0.698970004 (uM) Interaction | 59.96078501 | 13.49575156 | 4.442937818 | 8.92E-06 |
| Navitoclax..ABT.263..5uM:Ldose: -0.698970004 (uM) Interaction | 60.31839824 | 13.49575156 | 4.469436026 | 7.88E-06 |
| Azacitidine:Ldose: -0.698970004 (uM) Interaction | 80.38507684 | 13.49575156 | 5.956324585 | 2.62E-09 |
| Capecitabine:Ldose: -0.698970004 (uM) Interaction | 68.49076377 | 13.49575156 | 5.074987002 | 3.91E-07 |
| Megestrol.acetate:Ldose: -0.698970004 (uM) Interaction | 57.77633138 | 13.49575156 | 4.2810755 | 1.87E-05 |
| Cytarabine.HCl...Ara.C:Ldose: -0.698970004 (uM) Interaction | 17.40438032 | 13.49575156 | 1.289619199 | 0.197196753 |
| Gemcitabine.HCl:Ldose: -0.698970004 (uM) Interaction | 23.87652078 | 13.49575156 | 1.76918793 | 0.076876665 |
| Vinblastine.Sulfate:Ldose: -0.698970004 (uM) Interaction | 52.99925196 | 13.49575156 | 3.927106372 | 8.62E-05 |
| MLN9708..MLN2238.:Ldose: -0.698970004 (uM) Interaction | 42.86801971 | 13.49575156 | 3.17640848 | 0.001493211 |
| ABT.737:Ldose: -0.698970004 (uM) Interaction | 60.70693302 | 13.49575156 | 4.49822544 | 6.89E-06 |
| Streptozocin:Ldose: -0.698970004 (uM) Interaction | 69.83190902 | 13.49575156 | 5.174362368 | 2.31E-07 |
| Crizotinib:Ldose: -0.698970004 (uM) Interaction | 60.13562438 | 13.49575156 | 4.45589296 | 8.40E-06 |
| Sunitinib:Ldose: -0.698970004 (uM) Interaction | 65.60480424 | 13.49575156 | 4.861144926 | 1.18E-06 |
| Dexrazoxane:Ldose: -0.698970004 (uM) Interaction | 64.0459706 | 13.49575156 | 4.745639418 | 2.09E-06 |
| Mitomycin.C:Ldose: -0.698970004 (uM) Interaction | 62.37946252 | 13.49575156 | 4.622155515 | 3.82E-06 |
| Carfilzomib:Ldose: -0.698970004 (uM) Interaction | 8.676910214 | 13.49575156 | 0.642936421 | 0.520272199 |
| OSI.027:Ldose: -0.698970004 (uM) Interaction | 75.58473452 | 13.49575156 | 5.600631737 | 2.16E-08 |
| Bioymifi:Ldose: -0.698970004 (uM) Interaction | 64.60956126 | 13.49575156 | 4.787400017 | 1.70E-06 |
| Nelarabine:Ldose: -0.698970004 (uM) Interaction | 68.32205792 | 13.49575156 | 5.062486339 | 4.17E-07 |
| Raloxifene:Ldose: -0.698970004 (uM) Interaction | 51.11835982 | 13.49575156 | 3.787737169 | 0.000152439 |
| Quinacrine.HCl:Ldose: -0.698970004 (uM) Interaction | 70.74388808 | 13.49575156 | 5.241937638 | 1.60E-07 |
| Lenalidomide:Ldose: -0.698970004 (uM) Interaction | 65.49466994 | 13.49575156 | 4.852984261 | 1.22E-06 |
| Fludarabine.Phosphate:Ldose: -0.698970004 (uM) Interaction | 61.79138688 | 13.49575156 | 4.578580643 | 4.71E-06 |
| Nilotinib:Ldose: -0.698970004 (uM) Interaction | 62.21628743 | 13.49575156 | 4.610064666 | 4.05E-06 |
| Linsitinib:Ldose: -0.698970004 (uM) Interaction | 50.96463068 | 13.49575156 | 3.77634624 | 0.000159573 |
| Aphrocallistin.analogue:Ldose: -0.698970004 (uM) Interaction | 28.76519237 | 13.49575156 | 2.131425748 | 0.03306537 |
| Mitotane..o.p..DDD..Lysodren.:Ldose: -0.698970004 (uM) Interaction | 66.89895018 | 13.49575156 | 4.957037766 | 7.21E-07 |
| Etoposide:Ldose: -0.698970004 (uM) Interaction | 50.94066635 | 13.49575156 | 3.774570546 | 0.000160713 |
| Vandetanib:Ldose: -0.698970004 (uM) Interaction | 76.79163975 | 13.49575156 | 5.690060267 | 1.29E-08 |
| Carboplatin:Ldose: -0.698970004 (uM) Interaction | 62.90814463 | 13.49575156 | 4.661329481 | 3.16E-06 |
| Gefitinib:Ldose: -0.698970004 (uM) Interaction | 61.77323843 | 13.49575156 | 4.577235891 | 4.74E-06 |
| Vincristine.Sulfate:Ldose: -0.698970004 (uM) Interaction | 26.94805979 | 13.49575156 | 1.996780962 | 0.045861544 |
| Trametinib..GSK1120212.:Ldose: -0.698970004 (uM) Interaction | 63.67943498 | 13.49575156 | 4.718480085 | 2.39E-06 |
| MLN4924:Ldose: -0.698970004 (uM) Interaction | 46.04248334 | 13.49575156 | 3.411627957 | 0.000646948 |
| Bortezomib:Ldose: -0.698970004 (uM) Interaction | 45.80999163 | 13.49575156 | 3.394400928 | 0.000689029 |
| Fluorouracil...5.FU.:Ldose: -0.698970004 (uM) Interaction | 62.98187152 | 13.49575156 | 4.666792451 | 3.08E-06 |
| Lapatinib:Ldose: -0.698970004 (uM) Interaction | 71.56479478 | 13.49575156 | 5.302764684 | 1.15E-07 |
| Mitoxantrone:Ldose: -0.698970004 (uM) Interaction | 12.55481344 | 13.49575156 | 0.930278938 | 0.352237105 |
| Imatinib:Ldose: -0.698970004 (uM) Interaction | 61.77498682 | 13.49575156 | 4.577365442 | 4.73E-06 |
| Imiquimod:Ldose: -0.698970004 (uM) Interaction | 62.04441611 | 13.49575156 | 4.597329449 | 4.30E-06 |
| Dacomitinib..PF299804.:Ldose: -0.698970004 (uM) Interaction | 60.21778497 | 13.49575156 | 4.461980846 | 8.16E-06 |
| PD325901:Ldose: -0.698970004 (uM) Interaction | 55.30087191 | 13.49575156 | 4.097650408 | 4.19E-05 |
| Vismodegib:Ldose: -0.698970004 (uM) Interaction | 70.04948456 | 13.49575156 | 5.190484148 | 2.12E-07 |
| Temozolomide:Ldose: -0.698970004 (uM) Interaction | 67.16097588 | 13.49575156 | 4.976453187 | 6.53E-07 |
| Mercaptopurine:Ldose: -0.698970004 (uM) Interaction | 54.83581362 | 13.49575156 | 4.063190802 | 4.86E-05 |
| Dasatinib:Ldose: -0.698970004 (uM) Interaction | 67.83164605 | 13.49575156 | 5.026148098 | 5.04E-07 |
| Daunorubicin.HCl:Ldose: -0.698970004 (uM) Interaction | 7.938255191 | 13.49575156 | 0.58820401 | 0.556401533 |
| Sirolimus..Rapamycin.:Ldose: -0.698970004 (uM) Interaction | 69.8523168 | 13.49575156 | 5.175874531 | 2.29E-07 |
| INK.128..MLN0128.:Ldose: -0.698970004 (uM) Interaction | 19.09962727 | 13.49575156 | 1.415232578 | 0.157014773 |
| Quizartinib:Ldose: -0.698970004 (uM) Interaction | 61.32083911 | 13.49575156 | 4.54371428 | 5.56E-06 |
| Sorafenib:Ldose: -0.698970004 (uM) Interaction | 71.17112134 | 13.49575156 | 5.273594509 | 1.35E-07 |
| Carmustine:Ldose: -0.698970004 (uM) Interaction | 57.63108519 | 13.49575156 | 4.270313136 | 1.96E-05 |
| Uracil.mustard:Ldose: -0.698970004 (uM) Interaction | 56.93156635 | 13.49575156 | 4.218480614 | 2.47E-05 |
| Ixabepilone:Ldose: -0.698970004 (uM) Interaction | 23.11635877 | 13.49575156 | 1.712861908 | 0.086752339 |
| Valrubicin:Ldose: -0.698970004 (uM) Interaction | 45.81246409 | 13.49575156 | 3.39458413 | 0.000688568 |
| Triethylenemelamine:Ldose: -0.698970004 (uM) Interaction | 67.26200924 | 13.49575156 | 4.983939495 | 6.28E-07 |
| Palbociclib..PD.0332991..Isethionate:Ldose: -0.698970004 (uM) Interaction | 51.26647121 | 13.49575156 | 3.798711837 | 0.00014585 |
| Afatinib:Ldose: -0.698970004 (uM) Interaction | 59.75807474 | 13.49575156 | 4.427917516 | 9.56E-06 |
| Doxorubicin.HCl:Ldose: -0.698970004 (uM) Interaction | 29.71372422 | 13.49575156 | 2.201709484 | 0.027696399 |
| Exemestane:Ldose: -0.698970004 (uM) Interaction | 66.84613187 | 13.49575156 | 4.953124067 | 7.36E-07 |
| Tretinoin:Ldose: -0.698970004 (uM) Interaction | 55.31305278 | 13.49575156 | 4.098552979 | 4.17E-05 |
| Fulvestrant:Ldose: -0.698970004 (uM) Interaction | 71.66230971 | 13.49575156 | 5.309990286 | 1.11E-07 |
| Docetaxel:Ldose: -0.698970004 (uM) Interaction | 53.63837212 | 13.49575156 | 3.974463509 | 7.08E-05 |
| Everolimus:Ldose: -0.698970004 (uM) Interaction | 63.05688787 | 13.49575156 | 4.672350967 | 3.00E-06 |
| MLN.2480:Ldose: -0.698970004 (uM) Interaction | 54.42807558 | 13.49575156 | 4.032978476 | 5.53E-05 |
| LY2157299:Ldose: -0.698970004 (uM) Interaction | 67.47850451 | 13.49575156 | 4.99998123 | 5.78E-07 |
| Allopurinol:Ldose: -0.698970004 (uM) Interaction | 68.6290972 | 13.49575156 | 5.085237148 | 3.70E-07 |
| Pipobroman:Ldose: -0.698970004 (uM) Interaction | 55.80116205 | 13.49575156 | 4.134720603 | 3.57E-05 |
| Letrozole:Ldose: -0.698970004 (uM) Interaction | 61.49730072 | 13.49575156 | 4.556789626 | 5.22E-06 |
| Thiotepa:Ldose: -0.698970004 (uM) Interaction | 66.46903421 | 13.49575156 | 4.925182113 | 8.49E-07 |
| Plicamycin:Ldose: -0.698970004 (uM) Interaction | -37.67947904 | 13.49575156 | -2.791951146 | 0.005243742 |
| Erlotinib.HCl:Ldose: -0.698970004 (uM) Interaction | 72.59663751 | 13.49575156 | 5.37922154 | 7.56E-08 |
| MEK.162..ARRY.438162.:Ldose: -0.698970004 (uM) Interaction | 16.10468703 | 13.49575156 | 1.193315315 | 0.23275905 |
| Baricitinib..LY3009104..INCB028050.:Ldose: -0.698970004 (uM) Interaction | 53.17109941 | 13.49575156 | 3.939839819 | 8.18E-05 |
| Arsenic.Trioxide:Ldose: -0.698970004 (uM) Interaction | 61.69024727 | 13.49575156 | 4.571086462 | 4.88E-06 |
| Celecoxib:Ldose: -0.698970004 (uM) Interaction | 64.35919164 | 13.49575156 | 4.76884828 | 1.86E-06 |
| Bendamustine.HCl:Ldose: -0.698970004 (uM) Interaction | 66.09868763 | 13.49575156 | 4.897740397 | 9.76E-07 |
| Chlorambucil:Ldose: -0.698970004 (uM) Interaction | 60.184529 | 13.49575156 | 4.459516665 | 8.26E-06 |
| Zoledronic.Acid:Ldose: -0.698970004 (uM) Interaction | 65.19091196 | 13.49575156 | 4.830476587 | 1.37E-06 |
| Actinomycin.D:Ldose: -0.698970004 (uM) Interaction | 43.83652303 | 13.49575156 | 3.24817205 | 0.001163269 |
| Temsirolimus..CCI.779..Torisel.:Ldose: -0.698970004 (uM) Interaction | 54.78815885 | 13.49575156 | 4.059659708 | 4.93E-05 |
| Foretinib..GSK1363089.:Ldose: -0.698970004 (uM) Interaction | 55.78043208 | 13.49575156 | 4.133184566 | 3.59E-05 |
| Decitabine:Ldose: -0.698970004 (uM) Interaction | 71.39255202 | 13.49575156 | 5.290001944 | 1.24E-07 |
| Methotrexate:Ldose: -0.698970004 (uM) Interaction | 65.83120757 | 13.49575156 | 4.877920823 | 1.08E-06 |
| Axitinib:Ldose: -0.698970004 (uM) Interaction | 68.72762808 | 13.49575156 | 5.09253803 | 3.56E-07 |
| Oxaliplatin:Ldose: -0.698970004 (uM) Interaction | 59.58271859 | 13.49575156 | 4.414924082 | 1.02E-05 |
| Cabazitaxel:Ldose: -0.698970004 (uM) Interaction | 57.37140051 | 13.49575156 | 4.251071179 | 2.14E-05 |
| Amifostine:Ldose: -0.698970004 (uM) Interaction | 68.90834035 | 13.49575156 | 5.105928338 | 3.32E-07 |
| Flutamide..Eulexin.:Ldose: -0.698970004 (uM) Interaction | 56.91525657 | 13.49575156 | 4.217272102 | 2.48E-05 |
| LDK378:Ldose: -0.698970004 (uM) Interaction | 53.86989009 | 13.49575156 | 3.991618387 | 6.58E-05 |
| Pralatrexate:Ldose: -0.698970004 (uM) Interaction | 63.24364821 | 13.49575156 | 4.686189421 | 2.80E-06 |
| Topotecan.HCl:Ldose: -0.698970004 (uM) Interaction | -4.772476074 | 13.49575156 | -0.353628033 | 0.723621124 |
| Pemetrexed:Ldose: -0.698970004 (uM) Interaction | 58.06108952 | 13.49575156 | 4.302175336 | 1.70E-05 |
| Bleomycin.Sulfate:Ldose: -0.698970004 (uM) Interaction | 31.16823876 | 13.49575156 | 2.309485219 | 0.020926082 |
| Axitinib.1:Ldose: -0.698970004 (uM) Interaction | 65.15334327 | 13.49575156 | 4.827692846 | 1.39E-06 |
| Ibrutinib..PCI.32765.:Ldose: -0.698970004 (uM) Interaction | 66.34962399 | 13.49575156 | 4.916334127 | 8.88E-07 |
| Tamoxifen.Citrate:Ldose: -0.698970004 (uM) Interaction | 55.96722366 | 13.49575156 | 4.147025335 | 3.38E-05 |
| Vemurafenib:Ldose: -0.698970004 (uM) Interaction | 24.81144712 | 13.49575156 | 1.838463534 | 0.06600789 |
| Pazopanib.HCl:Ldose: -0.698970004 (uM) Interaction | 63.16364872 | 13.49575156 | 4.680261668 | 2.88E-06 |
| Abiraterone:Ldose: -0.698970004 (uM) Interaction | 61.26383524 | 13.49575156 | 4.539490443 | 5.67E-06 |
| Bosutinib..SKI.606.:Ldose: -0.698970004 (uM) Interaction | 56.97433722 | 13.49575156 | 4.221649825 | 2.44E-05 |
| Sabutoclax..BI.97C1.:Ldose: -0.698970004 (uM) Interaction | 56.22137631 | 13.49575156 | 4.165857384 | 3.11E-05 |
| Thioguanine:Ldose: -0.397940009 (uM) Interaction | 69.28913932 | 13.49575156 | 5.134144549 | 2.86E-07 |
| Irinotecan.HCl:Ldose: -0.397940009 (uM) Interaction | 70.16790843 | 13.49575156 | 5.199259048 | 2.02E-07 |
| Romidepsin:Ldose: -0.397940009 (uM) Interaction | 116.3358057 | 13.49575156 | 8.62017985 | 7.14E-18 |
| Paclitaxel:Ldose: -0.397940009 (uM) Interaction | 53.11356391 | 13.49575156 | 3.935576589 | 8.33E-05 |
| Alisertib..MLN8237.:Ldose: -0.397940009 (uM) Interaction | 56.15684991 | 13.49575156 | 4.161076146 | 3.18E-05 |
| Vorinostat:Ldose: -0.397940009 (uM) Interaction | 81.06429603 | 13.49575156 | 6.006652956 | 1.92E-09 |
| Busulfan:Ldose: -0.397940009 (uM) Interaction | 76.95873119 | 13.49575156 | 5.702441307 | 1.20E-08 |
| Mechlorethamine.HCl:Ldose: -0.397940009 (uM) Interaction | 88.10527027 | 13.49575156 | 6.528370788 | 6.80E-11 |
| Teniposide:Ldose: -0.397940009 (uM) Interaction | 35.64340491 | 13.49575156 | 2.641083362 | 0.008270103 |
| Vinorelbine.Tartrate:Ldose: -0.397940009 (uM) Interaction | 53.49940635 | 13.49575156 | 3.964166508 | 7.39E-05 |
| Cabozantinib..XL.184.:Ldose: -0.397940009 (uM) Interaction | 70.95152829 | 13.49575156 | 5.257323236 | 1.48E-07 |
| Dacarbazine:Ldose: -0.397940009 (uM) Interaction | 85.86996842 | 13.49575156 | 6.36274075 | 2.02E-10 |
| Clofarabine:Ldose: -0.397940009 (uM) Interaction | -0.178006051 | 13.49575156 | -0.013189784 | 0.989476502 |
| Cisplatin:Ldose: -0.397940009 (uM) Interaction | 77.27911014 | 13.49575156 | 5.726180552 | 1.04E-08 |
| Floxuridine:Ldose: -0.397940009 (uM) Interaction | 32.38844135 | 13.49575156 | 2.399899049 | 0.01640803 |
| Lomustine..CCNU.:Ldose: -0.397940009 (uM) Interaction | 80.77581125 | 13.49575156 | 5.985276987 | 2.19E-09 |
| Melphalan:Ldose: -0.397940009 (uM) Interaction | 76.76733772 | 13.49575156 | 5.68825955 | 1.30E-08 |
| BGJ398..NVPBGJ398.:Ldose: -0.397940009 (uM) Interaction | 74.48338991 | 13.49575156 | 5.519024973 | 3.45E-08 |
| Navitoclax..ABT.263..5uM:Ldose: -0.397940009 (uM) Interaction | 76.82823429 | 13.49575156 | 5.692771827 | 1.27E-08 |
| Azacitidine:Ldose: -0.397940009 (uM) Interaction | 86.80359639 | 13.49575156 | 6.431920147 | 1.29E-10 |
| Capecitabine:Ldose: -0.397940009 (uM) Interaction | 79.91675972 | 13.49575156 | 5.921623508 | 3.24E-09 |
| Megestrol.acetate:Ldose: -0.397940009 (uM) Interaction | 80.43340425 | 13.49575156 | 5.959905521 | 2.56E-09 |
| Cytarabine.HCl...Ara.C:Ldose: -0.397940009 (uM) Interaction | 20.0073968 | 13.49575156 | 1.482495933 | 0.138223015 |
| Gemcitabine.HCl:Ldose: -0.397940009 (uM) Interaction | 38.6807607 | 13.49575156 | 2.866143506 | 0.004159083 |
| Vinblastine.Sulfate:Ldose: -0.397940009 (uM) Interaction | 66.41870525 | 13.49575156 | 4.921452869 | 8.65E-07 |
| MLN9708..MLN2238.:Ldose: -0.397940009 (uM) Interaction | 38.90662451 | 13.49575156 | 2.882879425 | 0.003944481 |
| ABT.737:Ldose: -0.397940009 (uM) Interaction | 84.70675084 | 13.49575156 | 6.27654936 | 3.53E-10 |
| Streptozocin:Ldose: -0.397940009 (uM) Interaction | 85.03030872 | 13.49575156 | 6.300524155 | 3.02E-10 |
| Crizotinib:Ldose: -0.397940009 (uM) Interaction | 80.60700692 | 13.49575156 | 5.972769025 | 2.37E-09 |
| Sunitinib:Ldose: -0.397940009 (uM) Interaction | 93.93978459 | 13.49575156 | 6.960693085 | 3.48E-12 |
| Dexrazoxane:Ldose: -0.397940009 (uM) Interaction | 88.94194723 | 13.49575156 | 6.590366369 | 4.49E-11 |
| Mitomycin.C:Ldose: -0.397940009 (uM) Interaction | 80.44989826 | 13.49575156 | 5.961127684 | 2.54E-09 |
| Carfilzomib:Ldose: -0.397940009 (uM) Interaction | 22.3551488 | 13.49575156 | 1.656458234 | 0.097643622 |
| OSI.027:Ldose: -0.397940009 (uM) Interaction | 88.68159142 | 13.49575156 | 6.571074682 | 5.11E-11 |
| Bioymifi:Ldose: -0.397940009 (uM) Interaction | 83.0563719 | 13.49575156 | 6.154260584 | 7.68E-10 |
| Nelarabine:Ldose: -0.397940009 (uM) Interaction | 88.03923907 | 13.49575156 | 6.523478048 | 7.02E-11 |
| Raloxifene:Ldose: -0.397940009 (uM) Interaction | 74.43073538 | 13.49575156 | 5.51512341 | 3.53E-08 |
| Quinacrine.HCl:Ldose: -0.397940009 (uM) Interaction | 88.37129089 | 13.49575156 | 6.548082222 | 5.96E-11 |
| Lenalidomide:Ldose: -0.397940009 (uM) Interaction | 89.59057846 | 13.49575156 | 6.638428252 | 3.25E-11 |
| Fludarabine.Phosphate:Ldose: -0.397940009 (uM) Interaction | 90.55301159 | 13.49575156 | 6.709742037 | 2.00E-11 |
| Nilotinib:Ldose: -0.397940009 (uM) Interaction | 86.14959337 | 13.49575156 | 6.383460229 | 1.77E-10 |
| Linsitinib:Ldose: -0.397940009 (uM) Interaction | 74.18572052 | 13.49575156 | 5.496968448 | 3.91E-08 |
| Aphrocallistin.analogue:Ldose: -0.397940009 (uM) Interaction | 17.20142597 | 13.49575156 | 1.274580811 | 0.202471402 |
| Mitotane..o.p..DDD..Lysodren.:Ldose: -0.397940009 (uM) Interaction | 80.56049738 | 13.49575156 | 5.96932279 | 2.42E-09 |
| Etoposide:Ldose: -0.397940009 (uM) Interaction | 53.52896949 | 13.49575156 | 3.966357059 | 7.32E-05 |
| Vandetanib:Ldose: -0.397940009 (uM) Interaction | 92.06640449 | 13.49575156 | 6.821880505 | 9.22E-12 |
| Carboplatin:Ldose: -0.397940009 (uM) Interaction | 87.65098911 | 13.49575156 | 6.494709738 | 8.50E-11 |
| Gefitinib:Ldose: -0.397940009 (uM) Interaction | 83.18113775 | 13.49575156 | 6.163505408 | 7.24E-10 |
| Vincristine.Sulfate:Ldose: -0.397940009 (uM) Interaction | 39.04225812 | 13.49575156 | 2.892929522 | 0.003820493 |
| Trametinib..GSK1120212.:Ldose: -0.397940009 (uM) Interaction | 77.41764774 | 13.49575156 | 5.736445827 | 9.80E-09 |
| MLN4924:Ldose: -0.397940009 (uM) Interaction | 25.61390654 | 13.49575156 | 1.897923685 | 0.057719492 |
| Bortezomib:Ldose: -0.397940009 (uM) Interaction | 59.89668102 | 13.49575156 | 4.43818788 | 9.12E-06 |
| Fluorouracil...5.FU.:Ldose: -0.397940009 (uM) Interaction | 86.36457914 | 13.49575156 | 6.399390114 | 1.59E-10 |
| Lapatinib:Ldose: -0.397940009 (uM) Interaction | 88.31086048 | 13.49575156 | 6.543604486 | 6.14E-11 |
| Mitoxantrone:Ldose: -0.397940009 (uM) Interaction | 15.04756725 | 13.49575156 | 1.114985496 | 0.264869077 |
| Imatinib:Ldose: -0.397940009 (uM) Interaction | 85.06450259 | 13.49575156 | 6.303057832 | 2.97E-10 |
| Imiquimod:Ldose: -0.397940009 (uM) Interaction | 85.01258576 | 13.49575156 | 6.29921093 | 3.05E-10 |
| Dacomitinib..PF299804.:Ldose: -0.397940009 (uM) Interaction | 81.23981847 | 13.49575156 | 6.019658712 | 1.78E-09 |
| PD325901:Ldose: -0.397940009 (uM) Interaction | 68.47181099 | 13.49575156 | 5.07358265 | 3.94E-07 |
| Vismodegib:Ldose: -0.397940009 (uM) Interaction | 82.6836514 | 13.49575156 | 6.126642967 | 9.13E-10 |
| Temozolomide:Ldose: -0.397940009 (uM) Interaction | 84.04174803 | 13.49575156 | 6.227274386 | 4.83E-10 |
| Mercaptopurine:Ldose: -0.397940009 (uM) Interaction | 76.08266047 | 13.49575156 | 5.637526751 | 1.75E-08 |
| Dasatinib:Ldose: -0.397940009 (uM) Interaction | 84.14465022 | 13.49575156 | 6.23489917 | 4.60E-10 |
| Daunorubicin.HCl:Ldose: -0.397940009 (uM) Interaction | 6.472700869 | 13.49575156 | 0.479610256 | 0.631509421 |
| Sirolimus..Rapamycin.:Ldose: -0.397940009 (uM) Interaction | 84.37914767 | 13.49575156 | 6.252274819 | 4.12E-10 |
| INK.128..MLN0128.:Ldose: -0.397940009 (uM) Interaction | 26.05576816 | 13.49575156 | 1.930664479 | 0.053537667 |
| Quizartinib:Ldose: -0.397940009 (uM) Interaction | 78.3129227 | 13.49575156 | 5.802783367 | 6.61E-09 |
| Sorafenib:Ldose: -0.397940009 (uM) Interaction | 83.03415954 | 13.49575156 | 6.152614706 | 7.76E-10 |
| Carmustine:Ldose: -0.397940009 (uM) Interaction | 77.75809737 | 13.49575156 | 5.761672257 | 8.44E-09 |
| Uracil.mustard:Ldose: -0.397940009 (uM) Interaction | 77.45211417 | 13.49575156 | 5.738999699 | 9.65E-09 |
| Ixabepilone:Ldose: -0.397940009 (uM) Interaction | 13.59271223 | 13.49575156 | 1.007184533 | 0.313857418 |
| Valrubicin:Ldose: -0.397940009 (uM) Interaction | 52.91857849 | 13.49575156 | 3.921128678 | 8.84E-05 |
| Triethylenemelamine:Ldose: -0.397940009 (uM) Interaction | 83.05489795 | 13.49575156 | 6.154151368 | 7.68E-10 |
| Palbociclib..PD.0332991..Isethionate:Ldose: -0.397940009 (uM) Interaction | 66.8704266 | 13.49575156 | 4.954924243 | 7.29E-07 |
| Afatinib:Ldose: -0.397940009 (uM) Interaction | 66.39765352 | 13.49575156 | 4.91989299 | 8.72E-07 |
| Doxorubicin.HCl:Ldose: -0.397940009 (uM) Interaction | 27.19518891 | 13.49575156 | 2.015092585 | 0.043907348 |
| Exemestane:Ldose: -0.397940009 (uM) Interaction | 80.57114495 | 13.49575156 | 5.970111747 | 2.41E-09 |
| Tretinoin:Ldose: -0.397940009 (uM) Interaction | 77.18803033 | 13.49575156 | 5.719431775 | 1.08E-08 |
| Fulvestrant:Ldose: -0.397940009 (uM) Interaction | 85.55638769 | 13.49575156 | 6.339505235 | 2.35E-10 |
| Docetaxel:Ldose: -0.397940009 (uM) Interaction | 67.26384858 | 13.49575156 | 4.984075785 | 6.27E-07 |
| Everolimus:Ldose: -0.397940009 (uM) Interaction | 82.04688743 | 13.49575156 | 6.079460417 | 1.23E-09 |
| MLN.2480:Ldose: -0.397940009 (uM) Interaction | 71.48008557 | 13.49575156 | 5.296487953 | 1.19E-07 |
| LY2157299:Ldose: -0.397940009 (uM) Interaction | 77.00007159 | 13.49575156 | 5.705504523 | 1.18E-08 |
| Allopurinol:Ldose: -0.397940009 (uM) Interaction | 82.03183902 | 13.49575156 | 6.078345369 | 1.23E-09 |
| Pipobroman:Ldose: -0.397940009 (uM) Interaction | 65.06736208 | 13.49575156 | 4.821321863 | 1.44E-06 |
| Letrozole:Ldose: -0.397940009 (uM) Interaction | 77.46257413 | 13.49575156 | 5.739774755 | 9.61E-09 |
| Thiotepa:Ldose: -0.397940009 (uM) Interaction | 89.24166717 | 13.49575156 | 6.612574835 | 3.87E-11 |
| Plicamycin:Ldose: -0.397940009 (uM) Interaction | -25.315529 | 13.49575156 | -1.875814688 | 0.060694244 |
| Erlotinib.HCl:Ldose: -0.397940009 (uM) Interaction | 87.68497821 | 13.49575156 | 6.497228242 | 8.36E-11 |
| MEK.162..ARRY.438162.:Ldose: -0.397940009 (uM) Interaction | 25.51644644 | 13.49575156 | 1.890702146 | 0.058677524 |
| Baricitinib..LY3009104..INCB028050.:Ldose: -0.397940009 (uM) Interaction | 72.44859967 | 13.49575156 | 5.368252322 | 8.03E-08 |
| Arsenic.Trioxide:Ldose: -0.397940009 (uM) Interaction | 74.48291687 | 13.49575156 | 5.518989922 | 3.45E-08 |
| Celecoxib:Ldose: -0.397940009 (uM) Interaction | 74.59668549 | 13.49575156 | 5.52741988 | 3.29E-08 |
| Bendamustine.HCl:Ldose: -0.397940009 (uM) Interaction | 74.24035026 | 13.49575156 | 5.501016369 | 3.82E-08 |
| Chlorambucil:Ldose: -0.397940009 (uM) Interaction | 84.19205852 | 13.49575156 | 6.238412001 | 4.50E-10 |
| Zoledronic.Acid:Ldose: -0.397940009 (uM) Interaction | 82.1973866 | 13.49575156 | 6.090612013 | 1.14E-09 |
| Actinomycin.D:Ldose: -0.397940009 (uM) Interaction | 46.07452488 | 13.49575156 | 3.414002152 | 0.00064134 |
| Temsirolimus..CCI.779..Torisel.:Ldose: -0.397940009 (uM) Interaction | 73.09506264 | 13.49575156 | 5.416153543 | 6.15E-08 |
| Foretinib..GSK1363089.:Ldose: -0.397940009 (uM) Interaction | 65.90787416 | 13.49575156 | 4.883601617 | 1.05E-06 |
| Decitabine:Ldose: -0.397940009 (uM) Interaction | 77.20374863 | 13.49575156 | 5.720596461 | 1.08E-08 |
| Methotrexate:Ldose: -0.397940009 (uM) Interaction | 75.91649337 | 13.49575156 | 5.625214202 | 1.88E-08 |
| Axitinib:Ldose: -0.397940009 (uM) Interaction | 78.14396323 | 13.49575156 | 5.79026391 | 7.13E-09 |
| Oxaliplatin:Ldose: -0.397940009 (uM) Interaction | 80.83301835 | 13.49575156 | 5.989515884 | 2.14E-09 |
| Cabazitaxel:Ldose: -0.397940009 (uM) Interaction | 73.9172414 | 13.49575156 | 5.477074845 | 4.37E-08 |
| Amifostine:Ldose: -0.397940009 (uM) Interaction | 78.18402399 | 13.49575156 | 5.793232308 | 7.00E-09 |
| Flutamide..Eulexin.:Ldose: -0.397940009 (uM) Interaction | 75.76722482 | 13.49575156 | 5.614153792 | 2.00E-08 |
| LDK378:Ldose: -0.397940009 (uM) Interaction | 67.31881139 | 13.49575156 | 4.988148386 | 6.14E-07 |
| Pralatrexate:Ldose: -0.397940009 (uM) Interaction | 76.85892891 | 13.49575156 | 5.695046219 | 1.25E-08 |
| Topotecan.HCl:Ldose: -0.397940009 (uM) Interaction | -1.685569623 | 13.49575156 | -0.124896314 | 0.900606802 |
| Pemetrexed:Ldose: -0.397940009 (uM) Interaction | 76.64524964 | 13.49575156 | 5.679213141 | 1.37E-08 |
| Bleomycin.Sulfate:Ldose: -0.397940009 (uM) Interaction | 36.5059388 | 13.49575156 | 2.704994874 | 0.006835914 |
| Axitinib.1:Ldose: -0.397940009 (uM) Interaction | 74.7347774 | 13.49575156 | 5.537652131 | 3.10E-08 |
| Ibrutinib..PCI.32765.:Ldose: -0.397940009 (uM) Interaction | 76.77007109 | 13.49575156 | 5.688462085 | 1.30E-08 |
| Tamoxifen.Citrate:Ldose: -0.397940009 (uM) Interaction | 76.79554935 | 13.49575156 | 5.690349959 | 1.28E-08 |
| Vemurafenib:Ldose: -0.397940009 (uM) Interaction | 32.22424655 | 13.49575156 | 2.387732643 | 0.016961236 |
| Pazopanib.HCl:Ldose: -0.397940009 (uM) Interaction | 76.94486499 | 13.49575156 | 5.701413858 | 1.20E-08 |
| Abiraterone:Ldose: -0.397940009 (uM) Interaction | 77.33574672 | 13.49575156 | 5.730377175 | 1.02E-08 |
| Bosutinib..SKI.606.:Ldose: -0.397940009 (uM) Interaction | 60.26891493 | 13.49575156 | 4.465769442 | 8.02E-06 |
| Sabutoclax..BI.97C1.:Ldose: -0.397940009 (uM) Interaction | 47.12625701 | 13.49575156 | 3.49193276 | 0.0004805 |
| Thioguanine:Ldose: 0 (uM) Interaction | 47.8032694 | 13.49575156 | 3.542097613 | 0.000397797 |
| Irinotecan.HCl:Ldose: 0 (uM) Interaction | 62.68437141 | 13.49575156 | 4.644748469 | 3.43E-06 |
| Romidepsin:Ldose: 0 (uM) Interaction | 120.1899739 | 13.49575156 | 8.905763661 | 5.71E-19 |
| Paclitaxel:Ldose: 0 (uM) Interaction | 51.64274891 | 13.49575156 | 3.826593033 | 0.000130296 |
| Alisertib..MLN8237.:Ldose: 0 (uM) Interaction | 52.62340771 | 13.49575156 | 3.899257293 | 9.68E-05 |
| Vorinostat:Ldose: 0 (uM) Interaction | 71.71419992 | 13.49575156 | 5.313835216 | 1.08E-07 |
| Busulfan:Ldose: 0 (uM) Interaction | 77.42706432 | 13.49575156 | 5.737143571 | 9.76E-09 |
| Mechlorethamine.HCl:Ldose: 0 (uM) Interaction | 85.7977832 | 13.49575156 | 6.357392013 | 2.09E-10 |
| Teniposide:Ldose: 0 (uM) Interaction | 32.38088681 | 13.49575156 | 2.399339278 | 0.01643313 |
| Vinorelbine.Tartrate:Ldose: 0 (uM) Interaction | 23.53551723 | 13.49575156 | 1.743920457 | 0.081187226 |
| Cabozantinib..XL.184.:Ldose: 0 (uM) Interaction | 72.65259773 | 13.49575156 | 5.383368046 | 7.39E-08 |
| Dacarbazine:Ldose: 0 (uM) Interaction | 83.22599635 | 13.49575156 | 6.166829314 | 7.09E-10 |
| Clofarabine:Ldose: 0 (uM) Interaction | 2.194409869 | 13.49575156 | 0.162600049 | 0.870834873 |
| Cisplatin:Ldose: 0 (uM) Interaction | 74.80844057 | 13.49575156 | 5.543110379 | 3.01E-08 |
| Floxuridine:Ldose: 0 (uM) Interaction | 30.49845871 | 13.49575156 | 2.259856264 | 0.023840118 |
| Lomustine..CCNU.:Ldose: 0 (uM) Interaction | 83.05776135 | 13.49575156 | 6.154363539 | 7.67E-10 |
| Melphalan:Ldose: 0 (uM) Interaction | 79.07396905 | 13.49575156 | 5.859174917 | 4.72E-09 |
| BGJ398..NVPBGJ398.:Ldose: 0 (uM) Interaction | 77.02507237 | 13.49575156 | 5.707357016 | 1.16E-08 |
| Navitoclax..ABT.263..5uM:Ldose: 0 (uM) Interaction | 80.54641167 | 13.49575156 | 5.968279075 | 2.44E-09 |
| Azacitidine:Ldose: 0 (uM) Interaction | 87.42269789 | 13.49575156 | 6.477793954 | 9.51E-11 |
| Capecitabine:Ldose: 0 (uM) Interaction | 75.42820787 | 13.49575156 | 5.58903352 | 2.31E-08 |
| Megestrol.acetate:Ldose: 0 (uM) Interaction | 78.00954696 | 13.49575156 | 5.780304015 | 7.56E-09 |
| Cytarabine.HCl...Ara.C:Ldose: 0 (uM) Interaction | 3.867958797 | 13.49575156 | 0.286605661 | 0.774417036 |
| Gemcitabine.HCl:Ldose: 0 (uM) Interaction | 42.01425714 | 13.49575156 | 3.113146899 | 0.001853462 |
| Vinblastine.Sulfate:Ldose: 0 (uM) Interaction | 70.09903854 | 13.49575156 | 5.194155969 | 2.08E-07 |
| MLN9708..MLN2238.:Ldose: 0 (uM) Interaction | 41.51585543 | 13.49575156 | 3.076216633 | 0.002099082 |
| ABT.737:Ldose: 0 (uM) Interaction | 86.32674787 | 13.49575156 | 6.396586916 | 1.62E-10 |
| Streptozocin:Ldose: 0 (uM) Interaction | 80.95904634 | 13.49575156 | 5.998854229 | 2.02E-09 |
| Crizotinib:Ldose: 0 (uM) Interaction | 78.4975575 | 13.49575156 | 5.816464324 | 6.10E-09 |
| Sunitinib:Ldose: 0 (uM) Interaction | 86.58354452 | 13.49575156 | 6.415614878 | 1.43E-10 |
| Dexrazoxane:Ldose: 0 (uM) Interaction | 86.0041881 | 13.49575156 | 6.372686077 | 1.90E-10 |
| Mitomycin.C:Ldose: 0 (uM) Interaction | 51.80842393 | 13.49575156 | 3.83886912 | 0.000123956 |
| Carfilzomib:Ldose: 0 (uM) Interaction | 25.93351741 | 13.49575156 | 1.921606017 | 0.05466853 |
| OSI.027:Ldose: 0 (uM) Interaction | 88.3354628 | 13.49575156 | 6.545427454 | 6.07E-11 |
| Bioymifi:Ldose: 0 (uM) Interaction | 84.19213208 | 13.49575156 | 6.238417452 | 4.50E-10 |
| Nelarabine:Ldose: 0 (uM) Interaction | 89.20233017 | 13.49575156 | 6.609660066 | 3.94E-11 |
| Raloxifene:Ldose: 0 (uM) Interaction | 78.6039043 | 13.49575156 | 5.824344345 | 5.82E-09 |
| Quinacrine.HCl:Ldose: 0 (uM) Interaction | 83.55370491 | 13.49575156 | 6.191111663 | 6.08E-10 |
| Lenalidomide:Ldose: 0 (uM) Interaction | 87.06988709 | 13.49575156 | 6.451651594 | 1.13E-10 |
| Fludarabine.Phosphate:Ldose: 0 (uM) Interaction | 87.39310209 | 13.49575156 | 6.475600983 | 9.65E-11 |
| Nilotinib:Ldose: 0 (uM) Interaction | 84.19223164 | 13.49575156 | 6.238424829 | 4.50E-10 |
| Linsitinib:Ldose: 0 (uM) Interaction | 66.35074738 | 13.49575156 | 4.916417368 | 8.88E-07 |
| Aphrocallistin.analogue:Ldose: 0 (uM) Interaction | 2.806678392 | 13.49575156 | 0.20796755 | 0.835256271 |
| Mitotane..o.p..DDD..Lysodren.:Ldose: 0 (uM) Interaction | 80.77238479 | 13.49575156 | 5.985023095 | 2.20E-09 |
| Etoposide:Ldose: 0 (uM) Interaction | 41.13118743 | 13.49575156 | 3.047713736 | 0.002308688 |
| Vandetanib:Ldose: 0 (uM) Interaction | 89.65327399 | 13.49575156 | 6.643073827 | 3.15E-11 |
| Carboplatin:Ldose: 0 (uM) Interaction | 83.66073111 | 13.49575156 | 6.199042025 | 5.78E-10 |
| Gefitinib:Ldose: 0 (uM) Interaction | 84.03068446 | 13.49575156 | 6.226454604 | 4.86E-10 |
| Vincristine.Sulfate:Ldose: 0 (uM) Interaction | 39.01426661 | 13.49575156 | 2.890855424 | 0.003845787 |
| Trametinib..GSK1120212.:Ldose: 0 (uM) Interaction | 80.66891352 | 13.49575156 | 5.977356144 | 2.30E-09 |
| MLN4924:Ldose: 0 (uM) Interaction | 7.936089769 | 13.49575156 | 0.588043558 | 0.556509221 |
| Bortezomib:Ldose: 0 (uM) Interaction | 63.24349017 | 13.49575156 | 4.686177711 | 2.80E-06 |
| Fluorouracil...5.FU.:Ldose: 0 (uM) Interaction | 85.39559159 | 13.49575156 | 6.327590663 | 2.54E-10 |
| Lapatinib:Ldose: 0 (uM) Interaction | 83.26274946 | 13.49575156 | 6.169552623 | 6.97E-10 |
| Mitoxantrone:Ldose: 0 (uM) Interaction | 12.0086807 | 13.49575156 | 0.889811927 | 0.373576819 |
| Imatinib:Ldose: 0 (uM) Interaction | 86.03935551 | 13.49575156 | 6.375291891 | 1.86E-10 |
| Imiquimod:Ldose: 0 (uM) Interaction | 83.43840214 | 13.49575156 | 6.182568029 | 6.42E-10 |
| Dacomitinib..PF299804.:Ldose: 0 (uM) Interaction | 79.75047509 | 13.49575156 | 5.90930225 | 3.49E-09 |
| PD325901:Ldose: 0 (uM) Interaction | 71.50148263 | 13.49575156 | 5.298073419 | 1.18E-07 |
| Vismodegib:Ldose: 0 (uM) Interaction | 84.01413367 | 13.49575156 | 6.225228234 | 4.90E-10 |
| Temozolomide:Ldose: 0 (uM) Interaction | 84.24055435 | 13.49575156 | 6.242005416 | 4.40E-10 |
| Mercaptopurine:Ldose: 0 (uM) Interaction | 66.88168221 | 13.49575156 | 4.955758254 | 7.26E-07 |
| Dasatinib:Ldose: 0 (uM) Interaction | 81.98569235 | 13.49575156 | 6.074926021 | 1.26E-09 |
| Daunorubicin.HCl:Ldose: 0 (uM) Interaction | -8.971122736 | 13.49575156 | -0.664736802 | 0.506225967 |
| Sirolimus..Rapamycin.:Ldose: 0 (uM) Interaction | 84.36530819 | 13.49575156 | 6.25124935 | 4.15E-10 |
| INK.128..MLN0128.:Ldose: 0 (uM) Interaction | 26.89586874 | 13.49575156 | 1.992913741 | 0.046283488 |
| Quizartinib:Ldose: 0 (uM) Interaction | 77.6300155 | 13.49575156 | 5.752181724 | 8.93E-09 |
| Sorafenib:Ldose: 0 (uM) Interaction | 79.44841611 | 13.49575156 | 5.886920468 | 3.99E-09 |
| Carmustine:Ldose: 0 (uM) Interaction | 76.93351242 | 13.49575156 | 5.700572662 | 1.21E-08 |
| Uracil.mustard:Ldose: 0 (uM) Interaction | 78.19120059 | 13.49575156 | 5.793764076 | 6.98E-09 |
| Ixabepilone:Ldose: 0 (uM) Interaction | 3.835360432 | 13.49575156 | 0.284190207 | 0.776267352 |
| Valrubicin:Ldose: 0 (uM) Interaction | 43.74832221 | 13.49575156 | 3.2416366 | 0.001190261 |
| Triethylenemelamine:Ldose: 0 (uM) Interaction | 83.4409452 | 13.49575156 | 6.182756463 | 6.41E-10 |
| Palbociclib..PD.0332991..Isethionate:Ldose: 0 (uM) Interaction | 65.42309742 | 13.49575156 | 4.847680925 | 1.26E-06 |
| Afatinib:Ldose: 0 (uM) Interaction | 64.72615393 | 13.49575156 | 4.796039229 | 1.63E-06 |
| Doxorubicin.HCl:Ldose: 0 (uM) Interaction | 6.984151689 | 13.49575156 | 0.517507429 | 0.604807299 |
| Exemestane:Ldose: 0 (uM) Interaction | 78.65490372 | 13.49575156 | 5.828123269 | 5.69E-09 |
| Tretinoin:Ldose: 0 (uM) Interaction | 72.8834677 | 13.49575156 | 5.400474909 | 6.72E-08 |
| Fulvestrant:Ldose: 0 (uM) Interaction | 83.82321012 | 13.49575156 | 6.211081296 | 5.36E-10 |
| Docetaxel:Ldose: 0 (uM) Interaction | 65.60796492 | 13.49575156 | 4.861379124 | 1.17E-06 |
| Everolimus:Ldose: 0 (uM) Interaction | 79.34689208 | 13.49575156 | 5.879397802 | 4.18E-09 |
| MLN.2480:Ldose: 0 (uM) Interaction | 51.61291851 | 13.49575156 | 3.824382678 | 0.00013147 |
| LY2157299:Ldose: 0 (uM) Interaction | 79.5770075 | 13.49575156 | 5.896448755 | 3.77E-09 |
| Allopurinol:Ldose: 0 (uM) Interaction | 87.62954384 | 13.49575156 | 6.4931207 | 8.59E-11 |
| Pipobroman:Ldose: 0 (uM) Interaction | 76.9538918 | 13.49575156 | 5.702082721 | 1.20E-08 |
| Letrozole:Ldose: 0 (uM) Interaction | 70.43768577 | 13.49575156 | 5.219248845 | 1.81E-07 |
| Thiotepa:Ldose: 0 (uM) Interaction | 85.3460321 | 13.49575156 | 6.323918434 | 2.60E-10 |
| Plicamycin:Ldose: 0 (uM) Interaction | -22.27443151 | 13.49575156 | -1.650477293 | 0.098859933 |
| Erlotinib.HCl:Ldose: 0 (uM) Interaction | 86.5308888 | 13.49575156 | 6.411713226 | 1.47E-10 |
| MEK.162..ARRY.438162.:Ldose: 0 (uM) Interaction | 24.82427769 | 13.49575156 | 1.839414246 | 0.065868034 |
| Baricitinib..LY3009104..INCB028050.:Ldose: 0 (uM) Interaction | 73.62302505 | 13.49575156 | 5.455274181 | 4.94E-08 |
| Arsenic.Trioxide:Ldose: 0 (uM) Interaction | 72.60543755 | 13.49575156 | 5.3798736 | 7.53E-08 |
| Celecoxib:Ldose: 0 (uM) Interaction | 71.85608255 | 13.49575156 | 5.324348349 | 1.02E-07 |
| Bendamustine.HCl:Ldose: 0 (uM) Interaction | 76.36264801 | 13.49575156 | 5.658273098 | 1.55E-08 |
| Chlorambucil:Ldose: 0 (uM) Interaction | 81.56973177 | 13.49575156 | 6.044104427 | 1.53E-09 |
| Zoledronic.Acid:Ldose: 0 (uM) Interaction | 84.34857262 | 13.49575156 | 6.250009288 | 4.18E-10 |
| Actinomycin.D:Ldose: 0 (uM) Interaction | 42.34872545 | 13.49575156 | 3.13793013 | 0.001703748 |
| Temsirolimus..CCI.779..Torisel.:Ldose: 0 (uM) Interaction | 75.3937632 | 13.49575156 | 5.58648126 | 2.35E-08 |
| Foretinib..GSK1363089.:Ldose: 0 (uM) Interaction | 50.83548974 | 13.49575156 | 3.766777234 | 0.000165808 |
| Decitabine:Ldose: 0 (uM) Interaction | 74.8908712 | 13.49575156 | 5.549218274 | 2.90E-08 |
| Methotrexate:Ldose: 0 (uM) Interaction | 78.40773292 | 13.49575156 | 5.809808557 | 6.34E-09 |
| Axitinib:Ldose: 0 (uM) Interaction | 78.16011591 | 13.49575156 | 5.791460782 | 7.07E-09 |
| Oxaliplatin:Ldose: 0 (uM) Interaction | 81.2003743 | 13.49575156 | 6.016736002 | 1.81E-09 |
| Cabazitaxel:Ldose: 0 (uM) Interaction | 80.26180975 | 13.49575156 | 5.947190816 | 2.77E-09 |
| Amifostine:Ldose: 0 (uM) Interaction | 80.74797035 | 13.49575156 | 5.983214049 | 2.22E-09 |
| Flutamide..Eulexin.:Ldose: 0 (uM) Interaction | 77.52171574 | 13.49575156 | 5.744156994 | 9.36E-09 |
| LDK378:Ldose: 0 (uM) Interaction | 65.03218118 | 13.49575156 | 4.81871505 | 1.45E-06 |
| Pralatrexate:Ldose: 0 (uM) Interaction | 80.60340114 | 13.49575156 | 5.972501847 | 2.37E-09 |
| Topotecan.HCl:Ldose: 0 (uM) Interaction | -7.112635544 | 13.49575156 | -0.527027747 | 0.598179747 |
| Pemetrexed:Ldose: 0 (uM) Interaction | 78.91227845 | 13.49575156 | 5.847194065 | 5.07E-09 |
| Bleomycin.Sulfate:Ldose: 0 (uM) Interaction | 28.5775686 | 13.49575156 | 2.117523316 | 0.034226885 |
| Axitinib.1:Ldose: 0 (uM) Interaction | 76.96393479 | 13.49575156 | 5.70282688 | 1.19E-08 |
| Ibrutinib..PCI.32765.:Ldose: 0 (uM) Interaction | 81.66379152 | 13.49575156 | 6.051074009 | 1.46E-09 |
| Tamoxifen.Citrate:Ldose: 0 (uM) Interaction | 82.69305507 | 13.49575156 | 6.127339754 | 9.09E-10 |
| Vemurafenib:Ldose: 0 (uM) Interaction | 25.65488189 | 13.49575156 | 1.900959851 | 0.057320606 |
| Pazopanib.HCl:Ldose: 0 (uM) Interaction | 76.65865135 | 13.49575156 | 5.680206173 | 1.36E-08 |
| Abiraterone:Ldose: 0 (uM) Interaction | 80.7030421 | 13.49575156 | 5.979884982 | 2.27E-09 |
| Bosutinib..SKI.606.:Ldose: 0 (uM) Interaction | 58.26922158 | 13.49575156 | 4.317597379 | 1.58E-05 |
| Sabutoclax..BI.97C1.:Ldose: 0 (uM) Interaction | 33.8637332 | 13.49575156 | 2.509214329 | 0.012107284 |
| Thioguanine:Ldose: 0.301029996 (uM) Interaction | -2.390027468 | 13.49575156 | -0.177094803 | 0.859435589 |
| Irinotecan.HCl:Ldose: 0.301029996 (uM) Interaction | 35.52898355 | 13.49575156 | 2.632605038 | 0.008479327 |
| Romidepsin:Ldose: 0.301029996 (uM) Interaction | 117.096438 | 13.49575156 | 8.676540718 | 4.36E-18 |
| Paclitaxel:Ldose: 0.301029996 (uM) Interaction | 58.21142067 | 13.49575156 | 4.313314482 | 1.62E-05 |
| Alisertib..MLN8237.:Ldose: 0.301029996 (uM) Interaction | 58.10690949 | 13.49575156 | 4.305570476 | 1.67E-05 |
| Vorinostat:Ldose: 0.301029996 (uM) Interaction | 50.43145367 | 13.49575156 | 3.736839215 | 0.000186829 |
| Busulfan:Ldose: 0.301029996 (uM) Interaction | 79.72005964 | 13.49575156 | 5.907048545 | 3.54E-09 |
| Mechlorethamine.HCl:Ldose: 0.301029996 (uM) Interaction | 79.73357801 | 13.49575156 | 5.908050221 | 3.51E-09 |
| Teniposide:Ldose: 0.301029996 (uM) Interaction | 37.48610749 | 13.49575156 | 2.777622818 | 0.005480549 |
| Vinorelbine.Tartrate:Ldose: 0.301029996 (uM) Interaction | 19.3328142 | 13.49575156 | 1.432511121 | 0.15201211 |
| Cabozantinib..XL.184.:Ldose: 0.301029996 (uM) Interaction | 65.99358269 | 13.49575156 | 4.889952395 | 1.02E-06 |
| Dacarbazine:Ldose: 0.301029996 (uM) Interaction | 82.23092312 | 13.49575156 | 6.093096982 | 1.13E-09 |
| Clofarabine:Ldose: 0.301029996 (uM) Interaction | 2.201486052 | 13.49575156 | 0.163124376 | 0.870422038 |
| Cisplatin:Ldose: 0.301029996 (uM) Interaction | 70.04536693 | 13.49575156 | 5.190179042 | 2.12E-07 |
| Floxuridine:Ldose: 0.301029996 (uM) Interaction | 27.20421327 | 13.49575156 | 2.015761267 | 0.043837339 |
| Lomustine..CCNU.:Ldose: 0.301029996 (uM) Interaction | 86.81289458 | 13.49575156 | 6.432609119 | 1.28E-10 |
| Melphalan:Ldose: 0.301029996 (uM) Interaction | 79.94605505 | 13.49575156 | 5.923794215 | 3.19E-09 |
| BGJ398..NVPBGJ398.:Ldose: 0.301029996 (uM) Interaction | 67.55955101 | 13.49575156 | 5.005986565 | 5.60E-07 |
| Navitoclax..ABT.263..5uM:Ldose: 0.301029996 (uM) Interaction | 78.54718283 | 13.49575156 | 5.820141432 | 5.96E-09 |
| Azacitidine:Ldose: 0.301029996 (uM) Interaction | 83.92428942 | 13.49575156 | 6.218571009 | 5.11E-10 |
| Capecitabine:Ldose: 0.301029996 (uM) Interaction | 85.36237634 | 13.49575156 | 6.3251295 | 2.58E-10 |
| Megestrol.acetate:Ldose: 0.301029996 (uM) Interaction | 64.2239035 | 13.49575156 | 4.758823782 | 1.96E-06 |
| Cytarabine.HCl...Ara.C:Ldose: 0.301029996 (uM) Interaction | -5.314567286 | 13.49575156 | -0.393795578 | 0.693735867 |
| Gemcitabine.HCl:Ldose: 0.301029996 (uM) Interaction | 41.75532291 | 13.49575156 | 3.093960548 | 0.001977566 |
| Vinblastine.Sulfate:Ldose: 0.301029996 (uM) Interaction | 69.02688166 | 13.49575156 | 5.11471194 | 3.17E-07 |
| MLN9708..MLN2238.:Ldose: 0.301029996 (uM) Interaction | -10.36392404 | 13.49575156 | -0.767939747 | 0.4425314 |
| ABT.737:Ldose: 0.301029996 (uM) Interaction | 73.26184653 | 13.49575156 | 5.428511793 | 5.74E-08 |
| Streptozocin:Ldose: 0.301029996 (uM) Interaction | 87.85353615 | 13.49575156 | 6.509717946 | 7.70E-11 |
| Crizotinib:Ldose: 0.301029996 (uM) Interaction | 74.72850723 | 13.49575156 | 5.537187527 | 3.11E-08 |
| Sunitinib:Ldose: 0.301029996 (uM) Interaction | 87.10192838 | 13.49575156 | 6.45402577 | 1.11E-10 |
| Dexrazoxane:Ldose: 0.301029996 (uM) Interaction | 84.68649185 | 13.49575156 | 6.275048221 | 3.56E-10 |
| Mitomycin.C:Ldose: 0.301029996 (uM) Interaction | 44.46487969 | 13.49575156 | 3.294731641 | 0.000986747 |
| Carfilzomib:Ldose: 0.301029996 (uM) Interaction | 25.32998344 | 13.49575156 | 1.876885724 | 0.060547264 |
| OSI.027:Ldose: 0.301029996 (uM) Interaction | 67.11647263 | 13.49575156 | 4.973155612 | 6.64E-07 |
| Bioymifi:Ldose: 0.301029996 (uM) Interaction | 74.76170639 | 13.49575156 | 5.539647499 | 3.07E-08 |
| Nelarabine:Ldose: 0.301029996 (uM) Interaction | 90.50197404 | 13.49575156 | 6.705960288 | 2.05E-11 |
| Raloxifene:Ldose: 0.301029996 (uM) Interaction | 79.5528351 | 13.49575156 | 5.894657643 | 3.81E-09 |
| Quinacrine.HCl:Ldose: 0.301029996 (uM) Interaction | 92.18993145 | 13.49575156 | 6.83103353 | 8.66E-12 |
| Lenalidomide:Ldose: 0.301029996 (uM) Interaction | 78.27479411 | 13.49575156 | 5.799958138 | 6.73E-09 |
| Fludarabine.Phosphate:Ldose: 0.301029996 (uM) Interaction | 77.1888865 | 13.49575156 | 5.719495215 | 1.08E-08 |
| Nilotinib:Ldose: 0.301029996 (uM) Interaction | 85.7391286 | 13.49575156 | 6.35304586 | 2.15E-10 |
| Linsitinib:Ldose: 0.301029996 (uM) Interaction | 71.03680064 | 13.49575156 | 5.263641695 | 1.43E-07 |
| Aphrocallistin.analogue:Ldose: 0.301029996 (uM) Interaction | -12.19744557 | 13.49575156 | -0.903798911 | 0.366112147 |
| Mitotane..o.p..DDD..Lysodren.:Ldose: 0.301029996 (uM) Interaction | 76.34719793 | 13.49575156 | 5.657128287 | 1.56E-08 |
| Etoposide:Ldose: 0.301029996 (uM) Interaction | 25.10149633 | 13.49575156 | 1.859955424 | 0.062905454 |
| Vandetanib:Ldose: 0.301029996 (uM) Interaction | 89.56529845 | 13.49575156 | 6.636555069 | 3.29E-11 |
| Carboplatin:Ldose: 0.301029996 (uM) Interaction | 93.17618189 | 13.49575156 | 6.904112116 | 5.19E-12 |
| Gefitinib:Ldose: 0.301029996 (uM) Interaction | 90.97596028 | 13.49575156 | 6.741081432 | 1.61E-11 |
| Vincristine.Sulfate:Ldose: 0.301029996 (uM) Interaction | 39.40494066 | 13.49575156 | 2.919803352 | 0.003506152 |
| Trametinib..GSK1120212.:Ldose: 0.301029996 (uM) Interaction | 80.51925567 | 13.49575156 | 5.966266887 | 2.47E-09 |
| MLN4924:Ldose: 0.301029996 (uM) Interaction | -5.603133725 | 13.49575156 | -0.415177599 | 0.678016032 |
| Bortezomib:Ldose: 0.301029996 (uM) Interaction | 63.13276044 | 13.49575156 | 4.677972926 | 2.91E-06 |
| Fluorouracil...5.FU.:Ldose: 0.301029996 (uM) Interaction | 89.96551439 | 13.49575156 | 6.666210026 | 2.69E-11 |
| Lapatinib:Ldose: 0.301029996 (uM) Interaction | 77.56061024 | 13.49575156 | 5.747038975 | 9.20E-09 |
| Mitoxantrone:Ldose: 0.301029996 (uM) Interaction | 6.328134352 | 13.49575156 | 0.468898255 | 0.639147109 |
| Imatinib:Ldose: 0.301029996 (uM) Interaction | 78.28955247 | 13.49575156 | 5.801051694 | 6.68E-09 |
| Imiquimod:Ldose: 0.301029996 (uM) Interaction | 75.27481443 | 13.49575156 | 5.577667466 | 2.47E-08 |
| Dacomitinib..PF299804.:Ldose: 0.301029996 (uM) Interaction | 76.16359168 | 13.49575156 | 5.643523543 | 1.69E-08 |
| PD325901:Ldose: 0.301029996 (uM) Interaction | 70.29259389 | 13.49575156 | 5.208497915 | 1.92E-07 |
| Vismodegib:Ldose: 0.301029996 (uM) Interaction | 76.99988543 | 13.49575156 | 5.705490729 | 1.18E-08 |
| Temozolomide:Ldose: 0.301029996 (uM) Interaction | 89.96197545 | 13.49575156 | 6.6659478 | 2.69E-11 |
| Mercaptopurine:Ldose: 0.301029996 (uM) Interaction | 47.46451103 | 13.49575156 | 3.516996501 | 0.000437357 |
| Dasatinib:Ldose: 0.301029996 (uM) Interaction | 84.94380481 | 13.49575156 | 6.294114441 | 3.15E-10 |
| Daunorubicin.HCl:Ldose: 0.301029996 (uM) Interaction | -10.69235417 | 13.49575156 | -0.792275563 | 0.428208742 |
| Sirolimus..Rapamycin.:Ldose: 0.301029996 (uM) Interaction | 85.85016522 | 13.49575156 | 6.361273384 | 2.04E-10 |
| INK.128..MLN0128.:Ldose: 0.301029996 (uM) Interaction | 24.69614585 | 13.49575156 | 1.829920011 | 0.067275713 |
| Quizartinib:Ldose: 0.301029996 (uM) Interaction | 79.42709335 | 13.49575156 | 5.885340507 | 4.03E-09 |
| Sorafenib:Ldose: 0.301029996 (uM) Interaction | 84.5037277 | 13.49575156 | 6.261505874 | 3.88E-10 |
| Carmustine:Ldose: 0.301029996 (uM) Interaction | 83.64059806 | 13.49575156 | 6.197550219 | 5.84E-10 |
| Uracil.mustard:Ldose: 0.301029996 (uM) Interaction | 82.93323413 | 13.49575156 | 6.145136396 | 8.13E-10 |
| Ixabepilone:Ldose: 0.301029996 (uM) Interaction | 0.764837537 | 13.49575156 | 0.056672467 | 0.954806632 |
| Valrubicin:Ldose: 0.301029996 (uM) Interaction | 24.88328383 | 13.49575156 | 1.843786447 | 0.065227995 |
| Triethylenemelamine:Ldose: 0.301029996 (uM) Interaction | 85.45966907 | 13.49575156 | 6.332338638 | 2.46E-10 |
| Palbociclib..PD.0332991..Isethionate:Ldose: 0.301029996 (uM) Interaction | 59.31015313 | 13.49575156 | 4.394727692 | 1.11E-05 |
| Afatinib:Ldose: 0.301029996 (uM) Interaction | 52.6402898 | 13.49575156 | 3.900508212 | 9.63E-05 |
| Doxorubicin.HCl:Ldose: 0.301029996 (uM) Interaction | 5.05001798 | 13.49575156 | 0.374193164 | 0.708264293 |
| Exemestane:Ldose: 0.301029996 (uM) Interaction | 86.53678065 | 13.49575156 | 6.412149797 | 1.46E-10 |
| Tretinoin:Ldose: 0.301029996 (uM) Interaction | 81.96055928 | 13.49575156 | 6.073063726 | 1.28E-09 |
| Fulvestrant:Ldose: 0.301029996 (uM) Interaction | 77.71992148 | 13.49575156 | 5.758843523 | 8.58E-09 |
| Docetaxel:Ldose: 0.301029996 (uM) Interaction | 72.02646529 | 13.49575156 | 5.336973265 | 9.55E-08 |
| Everolimus:Ldose: 0.301029996 (uM) Interaction | 74.68291091 | 13.49575156 | 5.533808959 | 3.17E-08 |
| MLN.2480:Ldose: 0.301029996 (uM) Interaction | 46.55513783 | 13.49575156 | 3.449614318 | 0.000562463 |
| LY2157299:Ldose: 0.301029996 (uM) Interaction | 79.27105196 | 13.49575156 | 5.873778247 | 4.32E-09 |
| Allopurinol:Ldose: 0.301029996 (uM) Interaction | 86.97926407 | 13.49575156 | 6.444936664 | 1.18E-10 |
| Pipobroman:Ldose: 0.301029996 (uM) Interaction | 78.75708333 | 13.49575156 | 5.835694512 | 5.43E-09 |
| Letrozole:Ldose: 0.301029996 (uM) Interaction | 71.01694148 | 13.49575156 | 5.262170183 | 1.44E-07 |
| Thiotepa:Ldose: 0.301029996 (uM) Interaction | 80.9556468 | 13.49575156 | 5.998602332 | 2.02E-09 |
| Plicamycin:Ldose: 0.301029996 (uM) Interaction | 28.70026926 | 13.49575156 | 2.126615114 | 0.033463411 |
| Erlotinib.HCl:Ldose: 0.301029996 (uM) Interaction | 79.40756418 | 13.49575156 | 5.883893446 | 4.07E-09 |
| MEK.162..ARRY.438162.:Ldose: 0.301029996 (uM) Interaction | 25.72623748 | 13.49575156 | 1.906247114 | 0.056631447 |
| Baricitinib..LY3009104..INCB028050.:Ldose: 0.301029996 (uM) Interaction | 66.32120772 | 13.49575156 | 4.914228556 | 8.98E-07 |
| Arsenic.Trioxide:Ldose: 0.301029996 (uM) Interaction | 77.15122708 | 13.49575156 | 5.716704751 | 1.10E-08 |
| Celecoxib:Ldose: 0.301029996 (uM) Interaction | 79.21714764 | 13.49575156 | 5.869784077 | 4.43E-09 |
| Bendamustine.HCl:Ldose: 0.301029996 (uM) Interaction | 81.9826475 | 13.49575156 | 6.074700406 | 1.26E-09 |
| Chlorambucil:Ldose: 0.301029996 (uM) Interaction | 77.4996285 | 13.49575156 | 5.742520387 | 9.45E-09 |
| Zoledronic.Acid:Ldose: 0.301029996 (uM) Interaction | 76.24186735 | 13.49575156 | 5.649323566 | 1.63E-08 |
| Actinomycin.D:Ldose: 0.301029996 (uM) Interaction | 47.04890247 | 13.49575156 | 3.48620099 | 0.000490908 |
| Temsirolimus..CCI.779..Torisel.:Ldose: 0.301029996 (uM) Interaction | 82.28852937 | 13.49575156 | 6.097365455 | 1.10E-09 |
| Foretinib..GSK1363089.:Ldose: 0.301029996 (uM) Interaction | 29.93418656 | 13.49575156 | 2.218045169 | 0.026562143 |
| Decitabine:Ldose: 0.301029996 (uM) Interaction | 74.83338114 | 13.49575156 | 5.54495841 | 2.97E-08 |
| Methotrexate:Ldose: 0.301029996 (uM) Interaction | 84.1646402 | 13.49575156 | 6.236380375 | 4.56E-10 |
| Axitinib:Ldose: 0.301029996 (uM) Interaction | 82.03086709 | 13.49575156 | 6.078273351 | 1.24E-09 |
| Oxaliplatin:Ldose: 0.301029996 (uM) Interaction | 79.69709445 | 13.49575156 | 5.905346884 | 3.57E-09 |
| Cabazitaxel:Ldose: 0.301029996 (uM) Interaction | 85.40190048 | 13.49575156 | 6.328058136 | 2.53E-10 |
| Amifostine:Ldose: 0.301029996 (uM) Interaction | 84.58172586 | 13.49575156 | 6.267285335 | 3.74E-10 |
| Flutamide..Eulexin.:Ldose: 0.301029996 (uM) Interaction | 73.72089705 | 13.49575156 | 5.46252624 | 4.75E-08 |
| LDK378:Ldose: 0.301029996 (uM) Interaction | 64.33603333 | 13.49575156 | 4.76713231 | 1.88E-06 |
| Pralatrexate:Ldose: 0.301029996 (uM) Interaction | 80.48594739 | 13.49575156 | 5.96379883 | 2.50E-09 |
| Topotecan.HCl:Ldose: 0.301029996 (uM) Interaction | -20.08143458 | 13.49575156 | -1.487981939 | 0.136770296 |
| Pemetrexed:Ldose: 0.301029996 (uM) Interaction | 72.4435416 | 13.49575156 | 5.367877532 | 8.05E-08 |
| Bleomycin.Sulfate:Ldose: 0.301029996 (uM) Interaction | 19.29600453 | 13.49575156 | 1.42978362 | 0.152793635 |
| Axitinib.1:Ldose: 0.301029996 (uM) Interaction | 81.50344385 | 13.49575156 | 6.039192664 | 1.57E-09 |
| Ibrutinib..PCI.32765.:Ldose: 0.301029996 (uM) Interaction | 83.60911126 | 13.49575156 | 6.195217129 | 5.93E-10 |
| Tamoxifen.Citrate:Ldose: 0.301029996 (uM) Interaction | 79.05774993 | 13.49575156 | 5.857973123 | 4.75E-09 |
| Vemurafenib:Ldose: 0.301029996 (uM) Interaction | 11.95992835 | 13.49575156 | 0.886199505 | 0.375519924 |
| Pazopanib.HCl:Ldose: 0.301029996 (uM) Interaction | 73.23361293 | 13.49575156 | 5.426419757 | 5.81E-08 |
| Abiraterone:Ldose: 0.301029996 (uM) Interaction | 83.1129006 | 13.49575156 | 6.158449213 | 7.48E-10 |
| Bosutinib..SKI.606.:Ldose: 0.301029996 (uM) Interaction | 45.32838526 | 13.49575156 | 3.358715152 | 0.000784415 |
| Sabutoclax..BI.97C1.:Ldose: 0.301029996 (uM) Interaction | 63.81832091 | 13.49575156 | 4.72877117 | 2.27E-06 |
| Thioguanine:Ldose: 0.602059991 (uM) Interaction | -4.579657791 | 13.49575156 | -0.339340701 | 0.734356393 |
| Irinotecan.HCl:Ldose: 0.602059991 (uM) Interaction | 23.68663394 | 13.49575156 | 1.75511781 | 0.079253373 |
| Romidepsin:Ldose: 0.602059991 (uM) Interaction | 119.5597343 | 13.49575156 | 8.859064555 | 8.68E-19 |
| Paclitaxel:Ldose: 0.602059991 (uM) Interaction | 58.32276125 | 13.49575156 | 4.321564529 | 1.56E-05 |
| Alisertib..MLN8237.:Ldose: 0.602059991 (uM) Interaction | 58.29432736 | 13.49575156 | 4.319457652 | 1.57E-05 |
| Vorinostat:Ldose: 0.602059991 (uM) Interaction | 14.68609748 | 13.49575156 | 1.088201529 | 0.276518326 |
| Busulfan:Ldose: 0.602059991 (uM) Interaction | 76.84783844 | 13.49575156 | 5.694224444 | 1.26E-08 |
| Mechlorethamine.HCl:Ldose: 0.602059991 (uM) Interaction | 84.25452776 | 13.49575156 | 6.243040809 | 4.37E-10 |
| Teniposide:Ldose: 0.602059991 (uM) Interaction | 38.5981397 | 13.49575156 | 2.860021505 | 0.004240194 |
| Vinorelbine.Tartrate:Ldose: 0.602059991 (uM) Interaction | 16.509383 | 13.49575156 | 1.223302231 | 0.221228947 |
| Cabozantinib..XL.184.:Ldose: 0.602059991 (uM) Interaction | 64.5130725 | 13.49575156 | 4.780250451 | 1.76E-06 |
| Dacarbazine:Ldose: 0.602059991 (uM) Interaction | 76.70916162 | 13.49575156 | 5.683948853 | 1.33E-08 |
| Clofarabine:Ldose: 0.602059991 (uM) Interaction | 2.338951557 | 13.49575156 | 0.173310211 | 0.862409226 |
| Cisplatin:Ldose: 0.602059991 (uM) Interaction | 78.64242839 | 13.49575156 | 5.82719888 | 5.72E-09 |
| Floxuridine:Ldose: 0.602059991 (uM) Interaction | 23.66921451 | 13.49575156 | 1.753827076 | 0.079474363 |
| Lomustine..CCNU.:Ldose: 0.602059991 (uM) Interaction | 86.64919798 | 13.49575156 | 6.420479628 | 1.39E-10 |
| Melphalan:Ldose: 0.602059991 (uM) Interaction | 72.64840045 | 13.49575156 | 5.383057039 | 7.40E-08 |
| BGJ398..NVPBGJ398.:Ldose: 0.602059991 (uM) Interaction | 77.9774698 | 13.49575156 | 5.777927182 | 7.67E-09 |
| Navitoclax..ABT.263..5uM:Ldose: 0.602059991 (uM) Interaction | 75.52838382 | 13.49575156 | 5.596456297 | 2.21E-08 |
| Azacitidine:Ldose: 0.602059991 (uM) Interaction | 71.58052312 | 13.49575156 | 5.303930113 | 1.14E-07 |
| Capecitabine:Ldose: 0.602059991 (uM) Interaction | 86.06072996 | 13.49575156 | 6.376875682 | 1.84E-10 |
| Megestrol.acetate:Ldose: 0.602059991 (uM) Interaction | 81.59080089 | 13.49575156 | 6.045665594 | 1.51E-09 |
| Cytarabine.HCl...Ara.C:Ldose: 0.602059991 (uM) Interaction | -7.796797028 | 13.49575156 | -0.577722329 | 0.56345764 |
| Gemcitabine.HCl:Ldose: 0.602059991 (uM) Interaction | 41.03260915 | 13.49575156 | 3.040409343 | 0.002365405 |
| Vinblastine.Sulfate:Ldose: 0.602059991 (uM) Interaction | 69.29121613 | 13.49575156 | 5.134298434 | 2.86E-07 |
| MLN9708..MLN2238.:Ldose: 0.602059991 (uM) Interaction | -10.05793407 | 13.49575156 | -0.745266688 | 0.456118647 |
| ABT.737:Ldose: 0.602059991 (uM) Interaction | 80.8763505 | 13.49575156 | 5.992726682 | 2.10E-09 |
| Streptozocin:Ldose: 0.602059991 (uM) Interaction | 90.07932656 | 13.49575156 | 6.674643211 | 2.54E-11 |
| Crizotinib:Ldose: 0.602059991 (uM) Interaction | 64.55493687 | 13.49575156 | 4.783352492 | 1.74E-06 |
| Sunitinib:Ldose: 0.602059991 (uM) Interaction | 84.29428389 | 13.49575156 | 6.245986635 | 4.29E-10 |
| Dexrazoxane:Ldose: 0.602059991 (uM) Interaction | 87.33478752 | 13.49575156 | 6.471280025 | 9.93E-11 |
| Mitomycin.C:Ldose: 0.602059991 (uM) Interaction | 17.77317726 | 13.49575156 | 1.316946091 | 0.187870706 |
| Carfilzomib:Ldose: 0.602059991 (uM) Interaction | 26.05673049 | 13.49575156 | 1.930735785 | 0.053528843 |
| OSI.027:Ldose: 0.602059991 (uM) Interaction | 49.34333993 | 13.49575156 | 3.656212823 | 0.000256578 |
| Bioymifi:Ldose: 0.602059991 (uM) Interaction | 83.52379545 | 13.49575156 | 6.18889545 | 6.17E-10 |
| Nelarabine:Ldose: 0.602059991 (uM) Interaction | 93.54149164 | 13.49575156 | 6.931180616 | 4.29E-12 |
| Raloxifene:Ldose: 0.602059991 (uM) Interaction | 77.40540527 | 13.49575156 | 5.735538692 | 9.85E-09 |
| Quinacrine.HCl:Ldose: 0.602059991 (uM) Interaction | 92.33363955 | 13.49575156 | 6.841681925 | 8.04E-12 |
| Lenalidomide:Ldose: 0.602059991 (uM) Interaction | 87.29697995 | 13.49575156 | 6.468478583 | 1.01E-10 |
| Fludarabine.Phosphate:Ldose: 0.602059991 (uM) Interaction | 74.49345209 | 13.49575156 | 5.519770554 | 3.43E-08 |
| Nilotinib:Ldose: 0.602059991 (uM) Interaction | 88.98594703 | 13.49575156 | 6.593626639 | 4.39E-11 |
| Linsitinib:Ldose: 0.602059991 (uM) Interaction | 71.25260085 | 13.49575156 | 5.279631928 | 1.31E-07 |
| Aphrocallistin.analogue:Ldose: 0.602059991 (uM) Interaction | -2.028752783 | 13.49575156 | -0.150325291 | 0.880509388 |
| Mitotane..o.p..DDD..Lysodren.:Ldose: 0.602059991 (uM) Interaction | 84.41180257 | 13.49575156 | 6.254694462 | 4.06E-10 |
| Etoposide:Ldose: 0.602059991 (uM) Interaction | 10.61037732 | 13.49575156 | 0.786201292 | 0.431758218 |
| Vandetanib:Ldose: 0.602059991 (uM) Interaction | 94.483417 | 13.49575156 | 7.000974829 | 2.62E-12 |
| Carboplatin:Ldose: 0.602059991 (uM) Interaction | 90.14979945 | 13.49575156 | 6.679865069 | 2.45E-11 |
| Gefitinib:Ldose: 0.602059991 (uM) Interaction | 88.5341941 | 13.49575156 | 6.560152925 | 5.50E-11 |
| Vincristine.Sulfate:Ldose: 0.602059991 (uM) Interaction | 38.96992072 | 13.49575156 | 2.887569509 | 0.003886171 |
| Trametinib..GSK1120212.:Ldose: 0.602059991 (uM) Interaction | 81.3506724 | 13.49575156 | 6.027872699 | 1.69E-09 |
| MLN4924:Ldose: 0.602059991 (uM) Interaction | -8.579104833 | 13.49575156 | -0.635689298 | 0.524985707 |
| Bortezomib:Ldose: 0.602059991 (uM) Interaction | 63.74765571 | 13.49575156 | 4.723535063 | 2.33E-06 |
| Fluorouracil...5.FU.:Ldose: 0.602059991 (uM) Interaction | 87.40712231 | 13.49575156 | 6.476639845 | 9.58E-11 |
| Lapatinib:Ldose: 0.602059991 (uM) Interaction | 92.29221884 | 13.49575156 | 6.838612759 | 8.21E-12 |
| Mitoxantrone:Ldose: 0.602059991 (uM) Interaction | -3.110907191 | 13.49575156 | -0.230510111 | 0.817697597 |
| Imatinib:Ldose: 0.602059991 (uM) Interaction | 91.49645169 | 13.49575156 | 6.779648489 | 1.24E-11 |
| Imiquimod:Ldose: 0.602059991 (uM) Interaction | 85.8426242 | 13.49575156 | 6.360714614 | 2.05E-10 |
| Dacomitinib..PF299804.:Ldose: 0.602059991 (uM) Interaction | 83.00208886 | 13.49575156 | 6.150238352 | 7.87E-10 |
| PD325901:Ldose: 0.602059991 (uM) Interaction | 70.05687407 | 13.49575156 | 5.191031692 | 2.11E-07 |
| Vismodegib:Ldose: 0.602059991 (uM) Interaction | 86.77124103 | 13.49575156 | 6.429522699 | 1.31E-10 |
| Temozolomide:Ldose: 0.602059991 (uM) Interaction | 86.69665375 | 13.49575156 | 6.423995977 | 1.36E-10 |
| Mercaptopurine:Ldose: 0.602059991 (uM) Interaction | -2.724930006 | 13.49575156 | -0.201910208 | 0.839988828 |
| Dasatinib:Ldose: 0.602059991 (uM) Interaction | 85.9361867 | 13.49575156 | 6.367647351 | 1.96E-10 |
| Daunorubicin.HCl:Ldose: 0.602059991 (uM) Interaction | -9.949652647 | 13.49575156 | -0.737243317 | 0.460982426 |
| Sirolimus..Rapamycin.:Ldose: 0.602059991 (uM) Interaction | 89.18768139 | 13.49575156 | 6.608574629 | 3.97E-11 |
| INK.128..MLN0128.:Ldose: 0.602059991 (uM) Interaction | 20.74062855 | 13.49575156 | 1.536826493 | 0.124350492 |
| Quizartinib:Ldose: 0.602059991 (uM) Interaction | 77.65642699 | 13.49575156 | 5.754138747 | 8.83E-09 |
| Sorafenib:Ldose: 0.602059991 (uM) Interaction | 76.96091908 | 13.49575156 | 5.702603424 | 1.20E-08 |
| Carmustine:Ldose: 0.602059991 (uM) Interaction | 82.15453611 | 13.49575156 | 6.087436903 | 1.17E-09 |
| Uracil.mustard:Ldose: 0.602059991 (uM) Interaction | 77.78461121 | 13.49575156 | 5.763636863 | 8.34E-09 |
| Ixabepilone:Ldose: 0.602059991 (uM) Interaction | -1.062519326 | 13.49575156 | -0.078729912 | 0.937248185 |
| Valrubicin:Ldose: 0.602059991 (uM) Interaction | 21.3385071 | 13.49575156 | 1.581127735 | 0.113863517 |
| Triethylenemelamine:Ldose: 0.602059991 (uM) Interaction | 81.75034573 | 13.49575156 | 6.05748745 | 1.41E-09 |
| Palbociclib..PD.0332991..Isethionate:Ldose: 0.602059991 (uM) Interaction | 56.44669002 | 13.49575156 | 4.182552543 | 2.89E-05 |
| Afatinib:Ldose: 0.602059991 (uM) Interaction | 46.68544545 | 13.49575156 | 3.459269773 | 0.00054269 |
| Doxorubicin.HCl:Ldose: 0.602059991 (uM) Interaction | 40.56117721 | 13.49575156 | 3.005477466 | 0.002654695 |
| Exemestane:Ldose: 0.602059991 (uM) Interaction | 80.73964052 | 13.49575156 | 5.98259683 | 2.23E-09 |
| Tretinoin:Ldose: 0.602059991 (uM) Interaction | 80.1058031 | 13.49575156 | 5.93563113 | 2.97E-09 |
| Fulvestrant:Ldose: 0.602059991 (uM) Interaction | 84.85708583 | 13.49575156 | 6.28768879 | 3.28E-10 |
| Docetaxel:Ldose: 0.602059991 (uM) Interaction | 76.17041977 | 13.49575156 | 5.644029487 | 1.68E-08 |
| Everolimus:Ldose: 0.602059991 (uM) Interaction | 83.68296613 | 13.49575156 | 6.200689582 | 5.72E-10 |
| MLN.2480:Ldose: 0.602059991 (uM) Interaction | 49.80736236 | 13.49575156 | 3.690595676 | 0.000224281 |
| LY2157299:Ldose: 0.602059991 (uM) Interaction | 74.05757417 | 13.49575156 | 5.487473137 | 4.12E-08 |
| Allopurinol:Ldose: 0.602059991 (uM) Interaction | 88.99222298 | 13.49575156 | 6.594091671 | 4.38E-11 |
| Pipobroman:Ldose: 0.602059991 (uM) Interaction | 81.84164469 | 13.49575156 | 6.064252465 | 1.35E-09 |
| Letrozole:Ldose: 0.602059991 (uM) Interaction | 78.47670717 | 13.49575156 | 5.814919369 | 6.15E-09 |
| Thiotepa:Ldose: 0.602059991 (uM) Interaction | 88.39441268 | 13.49575156 | 6.549795486 | 5.89E-11 |
| Plicamycin:Ldose: 0.602059991 (uM) Interaction | 30.6959679 | 13.49575156 | 2.27449118 | 0.022946259 |
| Erlotinib.HCl:Ldose: 0.602059991 (uM) Interaction | 74.24208395 | 13.49575156 | 5.501144831 | 3.82E-08 |
| MEK.162..ARRY.438162.:Ldose: 0.602059991 (uM) Interaction | 26.13124382 | 13.49575156 | 1.936257029 | 0.052849294 |
| Baricitinib..LY3009104..INCB028050.:Ldose: 0.602059991 (uM) Interaction | 63.63011934 | 13.49575156 | 4.714825924 | 2.43E-06 |
| Arsenic.Trioxide:Ldose: 0.602059991 (uM) Interaction | 78.33909128 | 13.49575156 | 5.80472239 | 6.54E-09 |
| Celecoxib:Ldose: 0.602059991 (uM) Interaction | 76.59113246 | 13.49575156 | 5.6752032 | 1.40E-08 |
| Bendamustine.HCl:Ldose: 0.602059991 (uM) Interaction | 79.5613898 | 13.49575156 | 5.895291524 | 3.80E-09 |
| Chlorambucil:Ldose: 0.602059991 (uM) Interaction | 78.83124324 | 13.49575156 | 5.841189568 | 5.26E-09 |
| Zoledronic.Acid:Ldose: 0.602059991 (uM) Interaction | 86.62765221 | 13.49575156 | 6.418883142 | 1.40E-10 |
| Actinomycin.D:Ldose: 0.602059991 (uM) Interaction | 42.48821895 | 13.49575156 | 3.148266234 | 0.001644662 |
| Temsirolimus..CCI.779..Torisel.:Ldose: 0.602059991 (uM) Interaction | 77.52232726 | 13.49575156 | 5.744202307 | 9.36E-09 |
| Foretinib..GSK1363089.:Ldose: 0.602059991 (uM) Interaction | 13.77832847 | 13.49575156 | 1.020938212 | 0.30729518 |
| Decitabine:Ldose: 0.602059991 (uM) Interaction | 71.59281084 | 13.49575156 | 5.304840601 | 1.14E-07 |
| Methotrexate:Ldose: 0.602059991 (uM) Interaction | 80.40072133 | 13.49575156 | 5.957483801 | 2.60E-09 |
| Axitinib:Ldose: 0.602059991 (uM) Interaction | 79.56433953 | 13.49575156 | 5.895510091 | 3.79E-09 |
| Oxaliplatin:Ldose: 0.602059991 (uM) Interaction | 82.39523778 | 13.49575156 | 6.105272269 | 1.04E-09 |
| Cabazitaxel:Ldose: 0.602059991 (uM) Interaction | 86.26887303 | 13.49575156 | 6.392298541 | 1.67E-10 |
| Amifostine:Ldose: 0.602059991 (uM) Interaction | 82.06584514 | 13.49575156 | 6.080865134 | 1.22E-09 |
| Flutamide..Eulexin.:Ldose: 0.602059991 (uM) Interaction | 73.77967444 | 13.49575156 | 5.466881492 | 4.63E-08 |
| LDK378:Ldose: 0.602059991 (uM) Interaction | 32.41945958 | 13.49575156 | 2.402197419 | 0.016305322 |
| Pralatrexate:Ldose: 0.602059991 (uM) Interaction | 80.69734225 | 13.49575156 | 5.979462638 | 2.27E-09 |
| Topotecan.HCl:Ldose: 0.602059991 (uM) Interaction | -21.49932894 | 13.49575156 | -1.593044214 | 0.111164946 |
| Pemetrexed:Ldose: 0.602059991 (uM) Interaction | 80.55680544 | 13.49575156 | 5.969049227 | 2.42E-09 |
| Bleomycin.Sulfate:Ldose: 0.602059991 (uM) Interaction | 10.45693468 | 13.49575156 | 0.774831593 | 0.438447684 |
| Axitinib.1:Ldose: 0.602059991 (uM) Interaction | 62.22712219 | 13.49575156 | 4.610867494 | 4.03E-06 |
| Ibrutinib..PCI.32765.:Ldose: 0.602059991 (uM) Interaction | 83.54668858 | 13.49575156 | 6.190591771 | 6.10E-10 |
| Tamoxifen.Citrate:Ldose: 0.602059991 (uM) Interaction | 78.69731244 | 13.49575156 | 5.831265645 | 5.58E-09 |
| Vemurafenib:Ldose: 0.602059991 (uM) Interaction | 11.12679159 | 13.49575156 | 0.824466243 | 0.409683818 |
| Pazopanib.HCl:Ldose: 0.602059991 (uM) Interaction | 65.60324955 | 13.49575156 | 4.861029727 | 1.18E-06 |
| Abiraterone:Ldose: 0.602059991 (uM) Interaction | 77.75032156 | 13.49575156 | 5.761096089 | 8.47E-09 |
| Bosutinib..SKI.606.:Ldose: 0.602059991 (uM) Interaction | 44.67682225 | 13.49575156 | 3.310436032 | 0.000933037 |
| Sabutoclax..BI.97C1.:Ldose: 0.602059991 (uM) Interaction | -0.266263048 | 13.49575156 | -0.019729398 | 0.984259422 |
| Thioguanine:Ldose: 1 (uM) Interaction | -6.339679805 | 13.49575156 | -0.469753742 | 0.638535722 |
| Irinotecan.HCl:Ldose: 1 (uM) Interaction | 5.995787142 | 13.49575156 | 0.444272193 | 0.656850266 |
| Romidepsin:Ldose: 1 (uM) Interaction | 102.6434192 | 13.49575156 | 7.605609713 | 2.95E-14 |
| Paclitaxel:Ldose: 1 (uM) Interaction | 52.46829899 | 13.49575156 | 3.887764141 | 0.000101475 |
| Alisertib..MLN8237.:Ldose: 1 (uM) Interaction | 53.01583386 | 13.49575156 | 3.928335047 | 8.58E-05 |
| Vorinostat:Ldose: 1 (uM) Interaction | -5.423838951 | 13.49575156 | -0.401892323 | 0.68776726 |
| Busulfan:Ldose: 1 (uM) Interaction | 75.58356387 | 13.49575156 | 5.600544995 | 2.16E-08 |
| Mechlorethamine.HCl:Ldose: 1 (uM) Interaction | 60.79177725 | 13.49575156 | 4.504512177 | 6.69E-06 |
| Teniposide:Ldose: 1 (uM) Interaction | 25.82242657 | 13.49575156 | 1.913374475 | 0.055713371 |
| Vinorelbine.Tartrate:Ldose: 1 (uM) Interaction | 10.48216226 | 13.49575156 | 0.776700891 | 0.43734379 |
| Cabozantinib..XL.184.:Ldose: 1 (uM) Interaction | 42.81054675 | 13.49575156 | 3.172149883 | 0.001515272 |
| Dacarbazine:Ldose: 1 (uM) Interaction | 54.19281474 | 13.49575156 | 4.015546262 | 5.95E-05 |
| Clofarabine:Ldose: 1 (uM) Interaction | 1.451640594 | 13.49575156 | 0.107562783 | 0.914343522 |
| Cisplatin:Ldose: 1 (uM) Interaction | 74.78670255 | 13.49575156 | 5.541499649 | 3.03E-08 |
| Floxuridine:Ldose: 1 (uM) Interaction | 21.16523728 | 13.49575156 | 1.568288893 | 0.116828416 |
| Lomustine..CCNU.:Ldose: 1 (uM) Interaction | 81.43694447 | 13.49575156 | 6.034265234 | 1.62E-09 |
| Melphalan:Ldose: 1 (uM) Interaction | 49.02110169 | 13.49575156 | 3.63233581 | 0.000281519 |
| BGJ398..NVPBGJ398.:Ldose: 1 (uM) Interaction | 70.88572646 | 13.49575156 | 5.252447492 | 1.52E-07 |
| Navitoclax..ABT.263..5uM:Ldose: 1 (uM) Interaction | 76.29358495 | 13.49575156 | 5.653155706 | 1.60E-08 |
| Azacitidine:Ldose: 1 (uM) Interaction | 39.40336761 | 13.49575156 | 2.919686794 | 0.003507463 |
| Capecitabine:Ldose: 1 (uM) Interaction | 82.60612122 | 13.49575156 | 6.120898182 | 9.47E-10 |
| Megestrol.acetate:Ldose: 1 (uM) Interaction | 75.58266009 | 13.49575156 | 5.600478027 | 2.16E-08 |
| Cytarabine.HCl...Ara.C:Ldose: 1 (uM) Interaction | -6.80617053 | 13.49575156 | -0.504319489 | 0.614042043 |
| Gemcitabine.HCl:Ldose: 1 (uM) Interaction | 37.47912471 | 13.49575156 | 2.777105412 | 0.005489278 |
| Vinblastine.Sulfate:Ldose: 1 (uM) Interaction | 70.17240747 | 13.49575156 | 5.199592415 | 2.02E-07 |
| MLN9708..MLN2238.:Ldose: 1 (uM) Interaction | -10.61916017 | 13.49575156 | -0.786852079 | 0.43137712 |
| ABT.737:Ldose: 1 (uM) Interaction | 81.00148832 | 13.49575156 | 6.001999069 | 1.98E-09 |
| Streptozocin:Ldose: 1 (uM) Interaction | 86.30380385 | 13.49575156 | 6.394886824 | 1.64E-10 |
| Crizotinib:Ldose: 1 (uM) Interaction | 13.68356404 | 13.49575156 | 1.013916415 | 0.310634027 |
| Sunitinib:Ldose: 1 (uM) Interaction | 65.74897065 | 13.49575156 | 4.871827281 | 1.11E-06 |
| Dexrazoxane:Ldose: 1 (uM) Interaction | 75.35873921 | 13.49575156 | 5.583886074 | 2.38E-08 |
| Mitomycin.C:Ldose: 1 (uM) Interaction | -1.482668698 | 13.49575156 | -0.109861884 | 0.912519935 |
| Carfilzomib:Ldose: 1 (uM) Interaction | 58.85095506 | 13.49575156 | 4.360702313 | 1.30E-05 |
| OSI.027:Ldose: 1 (uM) Interaction | 25.16575325 | 13.49575156 | 1.864716695 | 0.062234715 |
| Bioymifi:Ldose: 1 (uM) Interaction | 80.84717622 | 13.49575156 | 5.990564944 | 2.12E-09 |
| Nelarabine:Ldose: 1 (uM) Interaction | 88.227858 | 13.49575156 | 6.537454219 | 6.40E-11 |
| Raloxifene:Ldose: 1 (uM) Interaction | 74.35465624 | 13.49575156 | 5.509486144 | 3.64E-08 |
| Quinacrine.HCl:Ldose: 1 (uM) Interaction | 67.26578185 | 13.49575156 | 4.984219036 | 6.27E-07 |
| Lenalidomide:Ldose: 1 (uM) Interaction | 79.96131989 | 13.49575156 | 5.924925301 | 3.17E-09 |
| Fludarabine.Phosphate:Ldose: 1 (uM) Interaction | 26.96437263 | 13.49575156 | 1.997989701 | 0.045730327 |
| Nilotinib:Ldose: 1 (uM) Interaction | 82.05262776 | 13.49575156 | 6.07988576 | 1.22E-09 |
| Linsitinib:Ldose: 1 (uM) Interaction | 62.96768098 | 13.49575156 | 4.665740969 | 3.09E-06 |
| Aphrocallistin.analogue:Ldose: 1 (uM) Interaction | 26.33683624 | 13.49575156 | 1.951490891 | 0.051011622 |
| Mitotane..o.p..DDD..Lysodren.:Ldose: 1 (uM) Interaction | 78.77780259 | 13.49575156 | 5.837229755 | 5.38E-09 |
| Etoposide:Ldose: 1 (uM) Interaction | 2.859931584 | 13.49575156 | 0.211913473 | 0.832176544 |
| Vandetanib:Ldose: 1 (uM) Interaction | 73.76869181 | 13.49575156 | 5.466067707 | 4.65E-08 |
| Carboplatin:Ldose: 1 (uM) Interaction | 81.28473013 | 13.49575156 | 6.022986548 | 1.74E-09 |
| Gefitinib:Ldose: 1 (uM) Interaction | 82.10288158 | 13.49575156 | 6.083609437 | 1.19E-09 |
| Vincristine.Sulfate:Ldose: 1 (uM) Interaction | 37.51444997 | 13.49575156 | 2.779722922 | 0.005445247 |
| Trametinib..GSK1120212.:Ldose: 1 (uM) Interaction | 80.36031066 | 13.49575156 | 5.954489476 | 2.65E-09 |
| MLN4924:Ldose: 1 (uM) Interaction | -8.704927438 | 13.49575156 | -0.645012425 | 0.518926004 |
| Bortezomib:Ldose: 1 (uM) Interaction | 63.67341898 | 13.49575156 | 4.718034314 | 2.40E-06 |
| Fluorouracil...5.FU.:Ldose: 1 (uM) Interaction | 66.10342843 | 13.49575156 | 4.898091678 | 9.75E-07 |
| Lapatinib:Ldose: 1 (uM) Interaction | 82.94294686 | 13.49575156 | 6.145856084 | 8.09E-10 |
| Mitoxantrone:Ldose: 1 (uM) Interaction | -18.03147878 | 13.49575156 | -1.336085559 | 0.181535424 |
| Imatinib:Ldose: 1 (uM) Interaction | 80.58100599 | 13.49575156 | 5.970842425 | 2.40E-09 |
| Imiquimod:Ldose: 1 (uM) Interaction | 77.78104977 | 13.49575156 | 5.76337297 | 8.36E-09 |
| Dacomitinib..PF299804.:Ldose: 1 (uM) Interaction | 73.52898157 | 13.49575156 | 5.448305804 | 5.14E-08 |
| PD325901:Ldose: 1 (uM) Interaction | 69.34036776 | 13.49575156 | 5.137940442 | 2.80E-07 |
| Vismodegib:Ldose: 1 (uM) Interaction | 81.50942574 | 13.49575156 | 6.039635907 | 1.57E-09 |
| Temozolomide:Ldose: 1 (uM) Interaction | 71.47219443 | 13.49575156 | 5.29590324 | 1.20E-07 |
| Mercaptopurine:Ldose: 1 (uM) Interaction | -17.01694939 | 13.49575156 | -1.260911578 | 0.207354363 |
| Dasatinib:Ldose: 1 (uM) Interaction | 49.2358833 | 13.49575156 | 3.648250568 | 0.000264655 |
| Daunorubicin.HCl:Ldose: 1 (uM) Interaction | -9.866622029 | 13.49575156 | -0.731090965 | 0.464731545 |
| Sirolimus..Rapamycin.:Ldose: 1 (uM) Interaction | 87.15045229 | 13.49575156 | 6.457621266 | 1.09E-10 |
| INK.128..MLN0128.:Ldose: 1 (uM) Interaction | 15.00973248 | 13.49575156 | 1.112182038 | 0.266072302 |
| Quizartinib:Ldose: 1 (uM) Interaction | 75.50387925 | 13.49575156 | 5.594640572 | 2.24E-08 |
| Sorafenib:Ldose: 1 (uM) Interaction | 34.88752351 | 13.49575156 | 2.585074521 | 0.009742305 |
| Carmustine:Ldose: 1 (uM) Interaction | 75.4246409 | 13.49575156 | 5.588769217 | 2.31E-08 |
| Uracil.mustard:Ldose: 1 (uM) Interaction | 63.7625185 | 13.49575156 | 4.724636357 | 2.32E-06 |
| Ixabepilone:Ldose: 1 (uM) Interaction | -4.860739635 | 13.49575156 | -0.360168132 | 0.718724936 |
| Valrubicin:Ldose: 1 (uM) Interaction | 60.28246051 | 13.49575156 | 4.466773134 | 7.98E-06 |
| Triethylenemelamine:Ldose: 1 (uM) Interaction | 79.65391012 | 13.49575156 | 5.902147038 | 3.64E-09 |
| Palbociclib..PD.0332991..Isethionate:Ldose: 1 (uM) Interaction | 43.26116183 | 13.49575156 | 3.205539286 | 0.001350062 |
| Afatinib:Ldose: 1 (uM) Interaction | 9.337930392 | 13.49575156 | 0.691916293 | 0.488997356 |
| Doxorubicin.HCl:Ldose: 1 (uM) Interaction | -12.65247852 | 13.49575156 | -0.937515666 | 0.348503881 |
| Exemestane:Ldose: 1 (uM) Interaction | 78.73690152 | 13.49575156 | 5.834199092 | 5.48E-09 |
| Tretinoin:Ldose: 1 (uM) Interaction | 74.64962093 | 13.49575156 | 5.531342258 | 3.21E-08 |
| Fulvestrant:Ldose: 1 (uM) Interaction | 81.2516974 | 13.49575156 | 6.02053891 | 1.77E-09 |
| Docetaxel:Ldose: 1 (uM) Interaction | 81.93531479 | 13.49575156 | 6.071193175 | 1.29E-09 |
| Everolimus:Ldose: 1 (uM) Interaction | 79.11255425 | 13.49575156 | 5.86203398 | 4.64E-09 |
| MLN.2480:Ldose: 1 (uM) Interaction | 33.48626421 | 13.49575156 | 2.481244861 | 0.013100011 |
| LY2157299:Ldose: 1 (uM) Interaction | 74.79197786 | 13.49575156 | 5.541890536 | 3.03E-08 |
| Allopurinol:Ldose: 1 (uM) Interaction | 81.8525913 | 13.49575156 | 6.065063581 | 1.34E-09 |
| Pipobroman:Ldose: 1 (uM) Interaction | 78.44539878 | 13.49575156 | 5.812599499 | 6.24E-09 |
| Letrozole:Ldose: 1 (uM) Interaction | 74.52852897 | 13.49575156 | 5.522369659 | 3.38E-08 |
| Thiotepa:Ldose: 1 (uM) Interaction | 71.34213397 | 13.49575156 | 5.286266099 | 1.26E-07 |
| Plicamycin:Ldose: 1 (uM) Interaction | -22.17954058 | 13.49575156 | -1.643446123 | 0.100305259 |
| Erlotinib.HCl:Ldose: 1 (uM) Interaction | 56.80618525 | 13.49575156 | 4.209190202 | 2.57E-05 |
| MEK.162..ARRY.438162.:Ldose: 1 (uM) Interaction | 25.35541721 | 13.49575156 | 1.878770299 | 0.060289357 |
| Baricitinib..LY3009104..INCB028050.:Ldose: 1 (uM) Interaction | 59.72128119 | 13.49575156 | 4.425191209 | 9.68E-06 |
| Arsenic.Trioxide:Ldose: 1 (uM) Interaction | 70.99366727 | 13.49575156 | 5.260445625 | 1.45E-07 |
| Celecoxib:Ldose: 1 (uM) Interaction | 72.105942 | 13.49575156 | 5.342862282 | 9.24E-08 |
| Bendamustine.HCl:Ldose: 1 (uM) Interaction | 73.51091589 | 13.49575156 | 5.446967184 | 5.18E-08 |
| Chlorambucil:Ldose: 1 (uM) Interaction | 73.87678965 | 13.49575156 | 5.474077476 | 4.45E-08 |
| Zoledronic.Acid:Ldose: 1 (uM) Interaction | 83.73907974 | 13.49575156 | 6.204847454 | 5.57E-10 |
| Actinomycin.D:Ldose: 1 (uM) Interaction | 42.11502914 | 13.49575156 | 3.120613842 | 0.001807129 |
| Temsirolimus..CCI.779..Torisel.:Ldose: 1 (uM) Interaction | 71.97486175 | 13.49575156 | 5.333149577 | 9.75E-08 |
| Foretinib..GSK1363089.:Ldose: 1 (uM) Interaction | -16.14076372 | 13.49575156 | -1.195988504 | 0.23171421 |
| Decitabine:Ldose: 1 (uM) Interaction | 68.63111737 | 13.49575156 | 5.085386838 | 3.70E-07 |
| Methotrexate:Ldose: 1 (uM) Interaction | 77.80228037 | 13.49575156 | 5.764946102 | 8.28E-09 |
| Axitinib:Ldose: 1 (uM) Interaction | 48.50522361 | 13.49575156 | 3.59411059 | 0.000326228 |
| Oxaliplatin:Ldose: 1 (uM) Interaction | 78.39633794 | 13.49575156 | 5.808964219 | 6.37E-09 |
| Cabazitaxel:Ldose: 1 (uM) Interaction | 87.06175052 | 13.49575156 | 6.451048696 | 1.13E-10 |
| Amifostine:Ldose: 1 (uM) Interaction | 79.12862749 | 13.49575156 | 5.863224965 | 4.61E-09 |
| Flutamide..Eulexin.:Ldose: 1 (uM) Interaction | 65.89192597 | 13.49575156 | 4.882419898 | 1.06E-06 |
| LDK378:Ldose: 1 (uM) Interaction | -24.06506743 | 13.49575156 | -1.783158745 | 0.074574543 |
| Pralatrexate:Ldose: 1 (uM) Interaction | 80.54686089 | 13.49575156 | 5.968312361 | 2.43E-09 |
| Topotecan.HCl:Ldose: 1 (uM) Interaction | -21.30536187 | 13.49575156 | -1.578671759 | 0.11442605 |
| Pemetrexed:Ldose: 1 (uM) Interaction | 65.31286094 | 13.49575156 | 4.839512689 | 1.31E-06 |
| Bleomycin.Sulfate:Ldose: 1 (uM) Interaction | 2.026350772 | 13.49575156 | 0.150147308 | 0.880649802 |
| Axitinib.1:Ldose: 1 (uM) Interaction | 49.8170055 | 13.49575156 | 3.691310207 | 0.000223652 |
| Ibrutinib..PCI.32765.:Ldose: 1 (uM) Interaction | 77.90282127 | 13.49575156 | 5.77239592 | 7.92E-09 |
| Tamoxifen.Citrate:Ldose: 1 (uM) Interaction | 66.9012433 | 13.49575156 | 4.95720768 | 7.21E-07 |
| Vemurafenib:Ldose: 1 (uM) Interaction | 50.02684373 | 13.49575156 | 3.706858673 | 0.000210372 |
| Pazopanib.HCl:Ldose: 1 (uM) Interaction | 48.09310148 | 13.49575156 | 3.563573414 | 0.000366631 |
| Abiraterone:Ldose: 1 (uM) Interaction | 78.95965595 | 13.49575156 | 5.850704615 | 4.97E-09 |
| Bosutinib..SKI.606.:Ldose: 1 (uM) Interaction | 28.34212364 | 13.49575156 | 2.100077458 | 0.035733627 |
| Sabutoclax..BI.97C1.:Ldose: 1 (uM) Interaction | -19.96539565 | 13.49575156 | -1.479383757 | 0.139052403 |
| MeWo:Thioguanine:Ldose: -1.397940009 (uM) Interaction | -5.560929414 | 19.0858749 | -0.29136361 | 0.770776048 |
| SKMEL2:Thioguanine:Ldose: -1.397940009 (uM) Interaction | -9.396399611 | 19.0858749 | -0.492322184 | 0.622496618 |
| UACC0257:Thioguanine:Ldose: -1.397940009 (uM) Interaction | -16.45233837 | 19.0858749 | -0.862016463 | 0.388688055 |
| MeWo:Irinotecan.HCl:Ldose: -1.397940009 (uM) Interaction | -1.054579855 | 19.0858749 | -0.055254468 | 0.955936249 |
| SKMEL2:Irinotecan.HCl:Ldose: -1.397940009 (uM) Interaction | -7.984183042 | 19.0858749 | -0.418329424 | 0.67571045 |
| UACC0257:Irinotecan.HCl:Ldose: -1.397940009 (uM) Interaction | -8.192191859 | 19.0858749 | -0.429227997 | 0.667761603 |
| MeWo:Romidepsin:Ldose: -1.397940009 (uM) Interaction | -2.325415751 | 19.0858749 | -0.12183962 | 0.903027172 |
| SKMEL2:Romidepsin:Ldose: -1.397940009 (uM) Interaction | -1.617790179 | 19.0858749 | -0.084763742 | 0.932450007 |
| UACC0257:Romidepsin:Ldose: -1.397940009 (uM) Interaction | 3.510163043 | 19.0858749 | 0.183914181 | 0.854082511 |
| MeWo:Paclitaxel:Ldose: -1.397940009 (uM) Interaction | 0.911396498 | 19.0858749 | 0.047752409 | 0.961914008 |
| SKMEL2:Paclitaxel:Ldose: -1.397940009 (uM) Interaction | 0.581555749 | 19.0858749 | 0.030470479 | 0.975692119 |
| UACC0257:Paclitaxel:Ldose: -1.397940009 (uM) Interaction | 3.569485421 | 19.0858749 | 0.187022363 | 0.851644861 |
| MeWo:Alisertib..MLN8237.:Ldose: -1.397940009 (uM) Interaction | -8.89087586 | 19.0858749 | -0.465835384 | 0.641338046 |
| SKMEL2:Alisertib..MLN8237.:Ldose: -1.397940009 (uM) Interaction | -17.32245582 | 19.0858749 | -0.907606065 | 0.364096542 |
| UACC0257:Alisertib..MLN8237.:Ldose: -1.397940009 (uM) Interaction | -18.38206596 | 19.0858749 | -0.963124094 | 0.335496064 |
| MeWo:Vorinostat:Ldose: -1.397940009 (uM) Interaction | -12.12335582 | 19.0858749 | -0.635200424 | 0.525304454 |
| SKMEL2:Vorinostat:Ldose: -1.397940009 (uM) Interaction | -14.48019149 | 19.0858749 | -0.758686283 | 0.448048545 |
| UACC0257:Vorinostat:Ldose: -1.397940009 (uM) Interaction | 4.591098706 | 19.0858749 | 0.240549555 | 0.809906541 |
| MeWo:Busulfan:Ldose: -1.397940009 (uM) Interaction | -3.667909319 | 19.0858749 | -0.19217926 | 0.847603603 |
| SKMEL2:Busulfan:Ldose: -1.397940009 (uM) Interaction | -5.605283289 | 19.0858749 | -0.293687521 | 0.76899952 |
| UACC0257:Busulfan:Ldose: -1.397940009 (uM) Interaction | 7.484371738 | 19.0858749 | 0.392141926 | 0.694957231 |
| MeWo:Mechlorethamine.HCl:Ldose: -1.397940009 (uM) Interaction | -11.01000373 | 19.0858749 | -0.576866599 | 0.564035604 |
| SKMEL2:Mechlorethamine.HCl:Ldose: -1.397940009 (uM) Interaction | -13.27331315 | 19.0858749 | -0.695452172 | 0.486779477 |
| UACC0257:Mechlorethamine.HCl:Ldose: -1.397940009 (uM) Interaction | -11.17396066 | 19.0858749 | -0.585457084 | 0.558246549 |
| MeWo:Teniposide:Ldose: -1.397940009 (uM) Interaction | -2.579764969 | 19.0858749 | -0.135166189 | 0.892481742 |
| SKMEL2:Teniposide:Ldose: -1.397940009 (uM) Interaction | -1.335766161 | 19.0858749 | -0.069987159 | 0.94420453 |
| UACC0257:Teniposide:Ldose: -1.397940009 (uM) Interaction | 1.122533225 | 19.0858749 | 0.058814869 | 0.953100111 |
| MeWo:Vinorelbine.Tartrate:Ldose: -1.397940009 (uM) Interaction | -9.201057209 | 19.0858749 | -0.482087264 | 0.629748855 |
| SKMEL2:Vinorelbine.Tartrate:Ldose: -1.397940009 (uM) Interaction | -9.666114722 | 19.0858749 | -0.506453845 | 0.612543267 |
| UACC0257:Vinorelbine.Tartrate:Ldose: -1.397940009 (uM) Interaction | -12.99640476 | 19.0858749 | -0.680943621 | 0.495914485 |
| MeWo:Cabozantinib..XL.184.:Ldose: -1.397940009 (uM) Interaction | -7.62103624 | 19.0858749 | -0.399302431 | 0.689674335 |
| SKMEL2:Cabozantinib..XL.184.:Ldose: -1.397940009 (uM) Interaction | -7.908337801 | 19.0858749 | -0.41435553 | 0.678617878 |
| UACC0257:Cabozantinib..XL.184.:Ldose: -1.397940009 (uM) Interaction | -19.23685708 | 19.0858749 | -1.007910676 | 0.313508668 |
| MeWo:Dacarbazine:Ldose: -1.397940009 (uM) Interaction | -0.349589666 | 19.0858749 | -0.01831667 | 0.985386399 |
| SKMEL2:Dacarbazine:Ldose: -1.397940009 (uM) Interaction | 3.016076312 | 19.0858749 | 0.158026621 | 0.874437303 |
| UACC0257:Dacarbazine:Ldose: -1.397940009 (uM) Interaction | -2.384475139 | 19.0858749 | -0.124934023 | 0.900576949 |
| MeWo:Clofarabine:Ldose: -1.397940009 (uM) Interaction | 14.48332793 | 19.0858749 | 0.758850616 | 0.447950226 |
| SKMEL2:Clofarabine:Ldose: -1.397940009 (uM) Interaction | 15.94640357 | 19.0858749 | 0.835508126 | 0.403440941 |
| UACC0257:Clofarabine:Ldose: -1.397940009 (uM) Interaction | 7.315405653 | 19.0858749 | 0.383288987 | 0.701509287 |
| MeWo:Cisplatin:Ldose: -1.397940009 (uM) Interaction | -3.180715602 | 19.0858749 | -0.166652858 | 0.867644765 |
| SKMEL2:Cisplatin:Ldose: -1.397940009 (uM) Interaction | -3.359331281 | 19.0858749 | -0.176011385 | 0.860286652 |
| UACC0257:Cisplatin:Ldose: -1.397940009 (uM) Interaction | -14.47959826 | 19.0858749 | -0.758655201 | 0.448067143 |
| MeWo:Floxuridine:Ldose: -1.397940009 (uM) Interaction | 9.347231843 | 19.0858749 | 0.48974605 | 0.624318599 |
| SKMEL2:Floxuridine:Ldose: -1.397940009 (uM) Interaction | 10.38997031 | 19.0858749 | 0.544380091 | 0.586185578 |
| UACC0257:Floxuridine:Ldose: -1.397940009 (uM) Interaction | 3.005440268 | 19.0858749 | 0.157469348 | 0.87487644 |
| MeWo:Lomustine..CCNU.:Ldose: -1.397940009 (uM) Interaction | -12.89640605 | 19.0858749 | -0.675704212 | 0.499235715 |
| SKMEL2:Lomustine..CCNU.:Ldose: -1.397940009 (uM) Interaction | -14.94028232 | 19.0858749 | -0.782792636 | 0.433757498 |
| UACC0257:Lomustine..CCNU.:Ldose: -1.397940009 (uM) Interaction | -9.140925827 | 19.0858749 | -0.478936694 | 0.631988527 |
| MeWo:Melphalan:Ldose: -1.397940009 (uM) Interaction | -3.699308919 | 19.0858749 | -0.193824435 | 0.846315181 |
| SKMEL2:Melphalan:Ldose: -1.397940009 (uM) Interaction | -8.831892659 | 19.0858749 | -0.462744973 | 0.643551853 |
| UACC0257:Melphalan:Ldose: -1.397940009 (uM) Interaction | -10.57870572 | 19.0858749 | -0.554268839 | 0.579400627 |
| MeWo:BGJ398..NVPBGJ398.:Ldose: -1.397940009 (uM) Interaction | -1.174036675 | 19.0858749 | -0.06151338 | 0.95095093 |
| SKMEL2:BGJ398..NVPBGJ398.:Ldose: -1.397940009 (uM) Interaction | -8.056438389 | 19.0858749 | -0.422115226 | 0.672945128 |
| UACC0257:BGJ398..NVPBGJ398.:Ldose: -1.397940009 (uM) Interaction | -20.27665196 | 19.0858749 | -1.062390489 | 0.28807036 |
| MeWo:Navitoclax..ABT.263..5uM:Ldose: -1.397940009 (uM) Interaction | -9.883742485 | 19.0858749 | -0.517856401 | 0.604563782 |
| SKMEL2:Navitoclax..ABT.263..5uM:Ldose: -1.397940009 (uM) Interaction | -15.06520521 | 19.0858749 | -0.789337942 | 0.429923205 |
| UACC0257:Navitoclax..ABT.263..5uM:Ldose: -1.397940009 (uM) Interaction | -4.599721095 | 19.0858749 | -0.241001323 | 0.809556385 |
| MeWo:Azacitidine:Ldose: -1.397940009 (uM) Interaction | -15.4664137 | 19.0858749 | -0.810359168 | 0.417742731 |
| SKMEL2:Azacitidine:Ldose: -1.397940009 (uM) Interaction | -8.338797834 | 19.0858749 | -0.436909383 | 0.662181446 |
| UACC0257:Azacitidine:Ldose: -1.397940009 (uM) Interaction | -12.16127909 | 19.0858749 | -0.637187405 | 0.524009553 |
| MeWo:Capecitabine:Ldose: -1.397940009 (uM) Interaction | -12.5549275 | 19.0858749 | -0.657812522 | 0.510665637 |
| SKMEL2:Capecitabine:Ldose: -1.397940009 (uM) Interaction | -12.67671215 | 19.0858749 | -0.6641934 | 0.506573646 |
| UACC0257:Capecitabine:Ldose: -1.397940009 (uM) Interaction | -15.91663901 | 19.0858749 | -0.833948619 | 0.404319186 |
| MeWo:Megestrol.acetate:Ldose: -1.397940009 (uM) Interaction | -8.554079221 | 19.0858749 | -0.448189002 | 0.654021307 |
| SKMEL2:Megestrol.acetate:Ldose: -1.397940009 (uM) Interaction | -6.137716611 | 19.0858749 | -0.321584242 | 0.747770804 |
| UACC0257:Megestrol.acetate:Ldose: -1.397940009 (uM) Interaction | -22.82651428 | 19.0858749 | -1.195989935 | 0.231713652 |
| MeWo:Cytarabine.HCl...Ara.C:Ldose: -1.397940009 (uM) Interaction | -5.473279427 | 19.0858749 | -0.286771209 | 0.774290267 |
| SKMEL2:Cytarabine.HCl...Ara.C:Ldose: -1.397940009 (uM) Interaction | -13.20338769 | 19.0858749 | -0.691788444 | 0.489077651 |
| UACC0257:Cytarabine.HCl...Ara.C:Ldose: -1.397940009 (uM) Interaction | -17.39219437 | 19.0858749 | -0.911260001 | 0.362168594 |
| MeWo:Gemcitabine.HCl:Ldose: -1.397940009 (uM) Interaction | -8.311295804 | 19.0858749 | -0.435468421 | 0.663226819 |
| SKMEL2:Gemcitabine.HCl:Ldose: -1.397940009 (uM) Interaction | -10.24561587 | 19.0858749 | -0.536816673 | 0.591399798 |
| UACC0257:Gemcitabine.HCl:Ldose: -1.397940009 (uM) Interaction | -6.647311779 | 19.0858749 | -0.348284363 | 0.72763004 |
| MeWo:Vinblastine.Sulfate:Ldose: -1.397940009 (uM) Interaction | -11.34808564 | 19.0858749 | -0.594580322 | 0.552130282 |
| SKMEL2:Vinblastine.Sulfate:Ldose: -1.397940009 (uM) Interaction | -7.087773267 | 19.0858749 | -0.371362241 | 0.710371398 |
| UACC0257:Vinblastine.Sulfate:Ldose: -1.397940009 (uM) Interaction | -1.201958877 | 19.0858749 | -0.062976357 | 0.949785915 |
| MeWo:MLN9708..MLN2238.:Ldose: -1.397940009 (uM) Interaction | -34.62439686 | 19.0858749 | -1.814137264 | 0.069670502 |
| SKMEL2:MLN9708..MLN2238.:Ldose: -1.397940009 (uM) Interaction | -24.91182984 | 19.0858749 | -1.305249561 | 0.191821825 |
| UACC0257:MLN9708..MLN2238.:Ldose: -1.397940009 (uM) Interaction | -32.95122383 | 19.0858749 | -1.72647175 | 0.084276916 |
| MeWo:ABT.737:Ldose: -1.397940009 (uM) Interaction | -17.04467367 | 19.0858749 | -0.893051734 | 0.371839446 |
| SKMEL2:ABT.737:Ldose: -1.397940009 (uM) Interaction | -17.75075538 | 19.0858749 | -0.930046722 | 0.352357317 |
| UACC0257:ABT.737:Ldose: -1.397940009 (uM) Interaction | -29.74108334 | 19.0858749 | -1.558277182 | 0.119182247 |
| MeWo:Streptozocin:Ldose: -1.397940009 (uM) Interaction | -11.14639702 | 19.0858749 | -0.584012893 | 0.559217753 |
| SKMEL2:Streptozocin:Ldose: -1.397940009 (uM) Interaction | -6.439005689 | 19.0858749 | -0.337370214 | 0.735841123 |
| UACC0257:Streptozocin:Ldose: -1.397940009 (uM) Interaction | -4.929010235 | 19.0858749 | -0.258254351 | 0.796213081 |
| MeWo:Crizotinib:Ldose: -1.397940009 (uM) Interaction | -10.66076943 | 19.0858749 | -0.558568548 | 0.576462016 |
| SKMEL2:Crizotinib:Ldose: -1.397940009 (uM) Interaction | -11.53043799 | 19.0858749 | -0.604134631 | 0.545760499 |
| UACC0257:Crizotinib:Ldose: -1.397940009 (uM) Interaction | -22.68369972 | 19.0858749 | -1.1885072 | 0.234646748 |
| MeWo:Sunitinib:Ldose: -1.397940009 (uM) Interaction | -15.00108037 | 19.0858749 | -0.785978136 | 0.431888942 |
| SKMEL2:Sunitinib:Ldose: -1.397940009 (uM) Interaction | -20.7511971 | 19.0858749 | -1.087254172 | 0.276936662 |
| UACC0257:Sunitinib:Ldose: -1.397940009 (uM) Interaction | -9.771306727 | 19.0858749 | -0.511965356 | 0.608680505 |
| MeWo:Dexrazoxane:Ldose: -1.397940009 (uM) Interaction | -15.45603331 | 19.0858749 | -0.80981529 | 0.418055289 |
| SKMEL2:Dexrazoxane:Ldose: -1.397940009 (uM) Interaction | -18.92008238 | 19.0858749 | -0.991313339 | 0.321543737 |
| UACC0257:Dexrazoxane:Ldose: -1.397940009 (uM) Interaction | -17.22984172 | 19.0858749 | -0.902753571 | 0.366666792 |
| MeWo:Mitomycin.C:Ldose: -1.397940009 (uM) Interaction | -17.78439339 | 19.0858749 | -0.931809178 | 0.351445594 |
| SKMEL2:Mitomycin.C:Ldose: -1.397940009 (uM) Interaction | -24.51757225 | 19.0858749 | -1.284592526 | 0.198948526 |
| UACC0257:Mitomycin.C:Ldose: -1.397940009 (uM) Interaction | -28.43486952 | 19.0858749 | -1.48983841 | 0.136281371 |
| MeWo:Carfilzomib:Ldose: -1.397940009 (uM) Interaction | -18.23828056 | 19.0858749 | -0.955590491 | 0.339289892 |
| SKMEL2:Carfilzomib:Ldose: -1.397940009 (uM) Interaction | 23.26113463 | 19.0858749 | 1.218761768 | 0.222947974 |
| UACC0257:Carfilzomib:Ldose: -1.397940009 (uM) Interaction | 11.92299912 | 19.0858749 | 0.624702781 | 0.532172765 |
| MeWo:OSI.027:Ldose: -1.397940009 (uM) Interaction | -25.6361582 | 19.0858749 | -1.343200579 | 0.179221186 |
| SKMEL2:OSI.027:Ldose: -1.397940009 (uM) Interaction | -26.10055807 | 19.0858749 | -1.367532702 | 0.171472663 |
| UACC0257:OSI.027:Ldose: -1.397940009 (uM) Interaction | -28.30011066 | 19.0858749 | -1.482777751 | 0.138148099 |
| MeWo:Bioymifi:Ldose: -1.397940009 (uM) Interaction | -14.04734638 | 19.0858749 | -0.736007464 | 0.46173417 |
| SKMEL2:Bioymifi:Ldose: -1.397940009 (uM) Interaction | -17.11042993 | 19.0858749 | -0.896497018 | 0.369997391 |
| UACC0257:Bioymifi:Ldose: -1.397940009 (uM) Interaction | -23.64229485 | 19.0858749 | -1.23873257 | 0.215458051 |
| MeWo:Nelarabine:Ldose: -1.397940009 (uM) Interaction | -11.86337185 | 19.0858749 | -0.621578624 | 0.534225559 |
| SKMEL2:Nelarabine:Ldose: -1.397940009 (uM) Interaction | -7.000068897 | 19.0858749 | -0.366766991 | 0.713796435 |
| UACC0257:Nelarabine:Ldose: -1.397940009 (uM) Interaction | -11.2775051 | 19.0858749 | -0.590882271 | 0.554605505 |
| MeWo:Raloxifene:Ldose: -1.397940009 (uM) Interaction | -6.976484982 | 19.0858749 | -0.365531317 | 0.714718424 |
| SKMEL2:Raloxifene:Ldose: -1.397940009 (uM) Interaction | -8.014797647 | 19.0858749 | -0.419933469 | 0.674538246 |
| UACC0257:Raloxifene:Ldose: -1.397940009 (uM) Interaction | -20.94789629 | 19.0858749 | -1.09756018 | 0.272408858 |
| MeWo:Quinacrine.HCl:Ldose: -1.397940009 (uM) Interaction | -13.12561776 | 19.0858749 | -0.687713706 | 0.491640492 |
| SKMEL2:Quinacrine.HCl:Ldose: -1.397940009 (uM) Interaction | -18.24448703 | 19.0858749 | -0.955915677 | 0.339125566 |
| UACC0257:Quinacrine.HCl:Ldose: -1.397940009 (uM) Interaction | -8.79773573 | 19.0858749 | -0.460955328 | 0.644835309 |
| MeWo:Lenalidomide:Ldose: -1.397940009 (uM) Interaction | -12.85718871 | 19.0858749 | -0.673649428 | 0.500541449 |
| SKMEL2:Lenalidomide:Ldose: -1.397940009 (uM) Interaction | -21.0876395 | 19.0858749 | -1.104881993 | 0.269223067 |
| UACC0257:Lenalidomide:Ldose: -1.397940009 (uM) Interaction | -15.6947822 | 19.0858749 | -0.822324483 | 0.410901349 |
| MeWo:Fludarabine.Phosphate:Ldose: -1.397940009 (uM) Interaction | -16.86365534 | 19.0858749 | -0.88356732 | 0.376939688 |
| SKMEL2:Fludarabine.Phosphate:Ldose: -1.397940009 (uM) Interaction | -21.19914381 | 19.0858749 | -1.110724236 | 0.266699465 |
| UACC0257:Fludarabine.Phosphate:Ldose: -1.397940009 (uM) Interaction | -15.82833752 | 19.0858749 | -0.829322083 | 0.406931369 |
| MeWo:Nilotinib:Ldose: -1.397940009 (uM) Interaction | -17.22880086 | 19.0858749 | -0.902699035 | 0.366695743 |
| SKMEL2:Nilotinib:Ldose: -1.397940009 (uM) Interaction | -19.57824395 | 19.0858749 | -1.025797563 | 0.304998534 |
| UACC0257:Nilotinib:Ldose: -1.397940009 (uM) Interaction | -23.19104616 | 19.0858749 | -1.215089499 | 0.224345277 |
| MeWo:Linsitinib:Ldose: -1.397940009 (uM) Interaction | -10.77944832 | 19.0858749 | -0.564786701 | 0.572224735 |
| SKMEL2:Linsitinib:Ldose: -1.397940009 (uM) Interaction | -15.08713016 | 19.0858749 | -0.790486695 | 0.429252292 |
| UACC0257:Linsitinib:Ldose: -1.397940009 (uM) Interaction | -26.2345973 | 19.0858749 | -1.374555656 | 0.169283544 |
| MeWo:Aphrocallistin.analogue:Ldose: -1.397940009 (uM) Interaction | -5.239008402 | 19.0858749 | -0.274496633 | 0.783705623 |
| SKMEL2:Aphrocallistin.analogue:Ldose: -1.397940009 (uM) Interaction | -8.057939559 | 19.0858749 | -0.422193879 | 0.672887723 |
| UACC0257:Aphrocallistin.analogue:Ldose: -1.397940009 (uM) Interaction | -14.34278013 | 19.0858749 | -0.751486647 | 0.452368044 |
| MeWo:Mitotane..o.p..DDD..Lysodren.:Ldose: -1.397940009 (uM) Interaction | -6.9321009 | 19.0858749 | -0.363205823 | 0.716454702 |
| SKMEL2:Mitotane..o.p..DDD..Lysodren.:Ldose: -1.397940009 (uM) Interaction | -7.492394892 | 19.0858749 | -0.392562297 | 0.694646675 |
| UACC0257:Mitotane..o.p..DDD..Lysodren.:Ldose: -1.397940009 (uM) Interaction | -7.35921596 | 19.0858749 | -0.385584418 | 0.699808283 |
| MeWo:Etoposide:Ldose: -1.397940009 (uM) Interaction | -9.484374254 | 19.0858749 | -0.496931595 | 0.619242362 |
| SKMEL2:Etoposide:Ldose: -1.397940009 (uM) Interaction | -4.198374144 | 19.0858749 | -0.219972842 | 0.82589439 |
| UACC0257:Etoposide:Ldose: -1.397940009 (uM) Interaction | -13.50947373 | 19.0858749 | -0.707825751 | 0.479061132 |
| MeWo:Vandetanib:Ldose: -1.397940009 (uM) Interaction | -19.20708569 | 19.0858749 | -1.006350812 | 0.314258151 |
| SKMEL2:Vandetanib:Ldose: -1.397940009 (uM) Interaction | -19.86940516 | 19.0858749 | -1.041052887 | 0.297862688 |
| UACC0257:Vandetanib:Ldose: -1.397940009 (uM) Interaction | -14.84711388 | 19.0858749 | -0.777911097 | 0.43662997 |
| MeWo:Carboplatin:Ldose: -1.397940009 (uM) Interaction | -13.70029672 | 19.0858749 | -0.717823878 | 0.472873674 |
| SKMEL2:Carboplatin:Ldose: -1.397940009 (uM) Interaction | -15.03744248 | 19.0858749 | -0.78788332 | 0.430773628 |
| UACC0257:Carboplatin:Ldose: -1.397940009 (uM) Interaction | -13.65874217 | 19.0858749 | -0.715646636 | 0.474217327 |
| MeWo:Gefitinib:Ldose: -1.397940009 (uM) Interaction | -13.44715033 | 19.0858749 | -0.704560331 | 0.48109151 |
| SKMEL2:Gefitinib:Ldose: -1.397940009 (uM) Interaction | -22.13401166 | 19.0858749 | -1.159706421 | 0.24618121 |
| UACC0257:Gefitinib:Ldose: -1.397940009 (uM) Interaction | -5.226133404 | 19.0858749 | -0.273822051 | 0.784224003 |
| MeWo:Vincristine.Sulfate:Ldose: -1.397940009 (uM) Interaction | 1.348606581 | 19.0858749 | 0.07065993 | 0.943669068 |
| SKMEL2:Vincristine.Sulfate:Ldose: -1.397940009 (uM) Interaction | 19.73124347 | 19.0858749 | 1.033813937 | 0.301234761 |
| UACC0257:Vincristine.Sulfate:Ldose: -1.397940009 (uM) Interaction | 25.83106944 | 19.0858749 | 1.353412908 | 0.175937958 |
| MeWo:Trametinib..GSK1120212.:Ldose: -1.397940009 (uM) Interaction | -18.36073522 | 19.0858749 | -0.962006474 | 0.336057151 |
| SKMEL2:Trametinib..GSK1120212.:Ldose: -1.397940009 (uM) Interaction | -13.36804531 | 19.0858749 | -0.700415641 | 0.483675336 |
| UACC0257:Trametinib..GSK1120212.:Ldose: -1.397940009 (uM) Interaction | -5.531482874 | 19.0858749 | -0.289820766 | 0.77195615 |
| MeWo:MLN4924:Ldose: -1.397940009 (uM) Interaction | -11.15016357 | 19.0858749 | -0.584210241 | 0.559084991 |
| SKMEL2:MLN4924:Ldose: -1.397940009 (uM) Interaction | -4.588959624 | 19.0858749 | -0.240437478 | 0.809993415 |
| UACC0257:MLN4924:Ldose: -1.397940009 (uM) Interaction | -19.95806732 | 19.0858749 | -1.04569832 | 0.295712073 |
| MeWo:Bortezomib:Ldose: -1.397940009 (uM) Interaction | 0.07344764 | 19.0858749 | 0.003848272 | 0.996929566 |
| SKMEL2:Bortezomib:Ldose: -1.397940009 (uM) Interaction | 7.846642943 | 19.0858749 | 0.411123042 | 0.680986405 |
| UACC0257:Bortezomib:Ldose: -1.397940009 (uM) Interaction | 6.573959784 | 19.0858749 | 0.344441102 | 0.730517945 |
| MeWo:Fluorouracil...5.FU.:Ldose: -1.397940009 (uM) Interaction | -7.259652587 | 19.0858749 | -0.380367818 | 0.703676151 |
| SKMEL2:Fluorouracil...5.FU.:Ldose: -1.397940009 (uM) Interaction | -9.286109632 | 19.0858749 | -0.486543566 | 0.626586775 |
| UACC0257:Fluorouracil...5.FU.:Ldose: -1.397940009 (uM) Interaction | -10.9062182 | 19.0858749 | -0.571428779 | 0.567714987 |
| MeWo:Lapatinib:Ldose: -1.397940009 (uM) Interaction | -18.96256355 | 19.0858749 | -0.99353913 | 0.320458452 |
| SKMEL2:Lapatinib:Ldose: -1.397940009 (uM) Interaction | -21.91870518 | 19.0858749 | -1.148425488 | 0.25080571 |
| UACC0257:Lapatinib:Ldose: -1.397940009 (uM) Interaction | -10.7547919 | 19.0858749 | -0.563494834 | 0.573103843 |
| MeWo:Mitoxantrone:Ldose: -1.397940009 (uM) Interaction | 12.82977083 | 19.0858749 | 0.672212874 | 0.501455396 |
| SKMEL2:Mitoxantrone:Ldose: -1.397940009 (uM) Interaction | 17.92012896 | 19.0858749 | 0.938921012 | 0.347781829 |
| UACC0257:Mitoxantrone:Ldose: -1.397940009 (uM) Interaction | 7.105264001 | 19.0858749 | 0.372278664 | 0.709689045 |
| MeWo:Imatinib:Ldose: -1.397940009 (uM) Interaction | -11.39180924 | 19.0858749 | -0.59687121 | 0.550599644 |
| SKMEL2:Imatinib:Ldose: -1.397940009 (uM) Interaction | -18.67918783 | 19.0858749 | -0.978691725 | 0.327743314 |
| UACC0257:Imatinib:Ldose: -1.397940009 (uM) Interaction | -7.493911712 | 19.0858749 | -0.392641771 | 0.694587969 |
| MeWo:Imiquimod:Ldose: -1.397940009 (uM) Interaction | -15.13195699 | 19.0858749 | -0.792835386 | 0.427882469 |
| SKMEL2:Imiquimod:Ldose: -1.397940009 (uM) Interaction | -15.58369737 | 19.0858749 | -0.816504219 | 0.414220841 |
| UACC0257:Imiquimod:Ldose: -1.397940009 (uM) Interaction | -8.14274518 | 19.0858749 | -0.42663725 | 0.669647825 |
| MeWo:Dacomitinib..PF299804.:Ldose: -1.397940009 (uM) Interaction | -16.81028014 | 19.0858749 | -0.880770739 | 0.378451747 |
| SKMEL2:Dacomitinib..PF299804.:Ldose: -1.397940009 (uM) Interaction | -16.19107465 | 19.0858749 | -0.848327611 | 0.396264972 |
| UACC0257:Dacomitinib..PF299804.:Ldose: -1.397940009 (uM) Interaction | -13.48570823 | 19.0858749 | -0.706580563 | 0.479834814 |
| MeWo:PD325901:Ldose: -1.397940009 (uM) Interaction | -8.838756517 | 19.0858749 | -0.463104603 | 0.643294069 |
| SKMEL2:PD325901:Ldose: -1.397940009 (uM) Interaction | -13.07406599 | 19.0858749 | -0.685012663 | 0.493343301 |
| UACC0257:PD325901:Ldose: -1.397940009 (uM) Interaction | -5.857691364 | 19.0858749 | -0.306912384 | 0.758913029 |
| MeWo:Vismodegib:Ldose: -1.397940009 (uM) Interaction | -9.748336919 | 19.0858749 | -0.510761858 | 0.609523055 |
| SKMEL2:Vismodegib:Ldose: -1.397940009 (uM) Interaction | -4.732475606 | 19.0858749 | -0.247956965 | 0.804170068 |
| UACC0257:Vismodegib:Ldose: -1.397940009 (uM) Interaction | -8.467227659 | 19.0858749 | -0.443638434 | 0.657308469 |
| MeWo:Temozolomide:Ldose: -1.397940009 (uM) Interaction | -11.29621386 | 19.0858749 | -0.591862512 | 0.55394887 |
| SKMEL2:Temozolomide:Ldose: -1.397940009 (uM) Interaction | -16.41099195 | 19.0858749 | -0.859850127 | 0.389881233 |
| UACC0257:Temozolomide:Ldose: -1.397940009 (uM) Interaction | -26.52691826 | 19.0858749 | -1.389871745 | 0.16458219 |
| MeWo:Mercaptopurine:Ldose: -1.397940009 (uM) Interaction | -7.909840455 | 19.0858749 | -0.414434261 | 0.678560229 |
| SKMEL2:Mercaptopurine:Ldose: -1.397940009 (uM) Interaction | -9.188167912 | 19.0858749 | -0.481411932 | 0.630228648 |
| UACC0257:Mercaptopurine:Ldose: -1.397940009 (uM) Interaction | -1.138076497 | 19.0858749 | -0.059629255 | 0.952451471 |
| MeWo:Dasatinib:Ldose: -1.397940009 (uM) Interaction | -16.10628612 | 19.0858749 | -0.843885135 | 0.398742967 |
| SKMEL2:Dasatinib:Ldose: -1.397940009 (uM) Interaction | -20.02527986 | 19.0858749 | -1.049219906 | 0.294088691 |
| UACC0257:Dasatinib:Ldose: -1.397940009 (uM) Interaction | -14.39386791 | 19.0858749 | -0.754163379 | 0.450759368 |
| MeWo:Daunorubicin.HCl:Ldose: -1.397940009 (uM) Interaction | 2.346229413 | 19.0858749 | 0.122930147 | 0.902163559 |
| SKMEL2:Daunorubicin.HCl:Ldose: -1.397940009 (uM) Interaction | -7.183450732 | 19.0858749 | -0.376375239 | 0.706641658 |
| UACC0257:Daunorubicin.HCl:Ldose: -1.397940009 (uM) Interaction | -6.108447229 | 19.0858749 | -0.320050679 | 0.748933017 |
| MeWo:Sirolimus..Rapamycin.:Ldose: -1.397940009 (uM) Interaction | -16.42312456 | 19.0858749 | -0.860485812 | 0.389530878 |
| SKMEL2:Sirolimus..Rapamycin.:Ldose: -1.397940009 (uM) Interaction | -13.28192937 | 19.0858749 | -0.695903617 | 0.4864967 |
| UACC0257:Sirolimus..Rapamycin.:Ldose: -1.397940009 (uM) Interaction | -18.64508555 | 19.0858749 | -0.976904944 | 0.328627185 |
| MeWo:INK.128..MLN0128.:Ldose: -1.397940009 (uM) Interaction | -7.752095555 | 19.0858749 | -0.406169253 | 0.684622279 |
| SKMEL2:INK.128..MLN0128.:Ldose: -1.397940009 (uM) Interaction | -4.623918947 | 19.0858749 | -0.242269164 | 0.808573913 |
| UACC0257:INK.128..MLN0128.:Ldose: -1.397940009 (uM) Interaction | -9.171468205 | 19.0858749 | -0.480536955 | 0.630850513 |
| MeWo:Quizartinib:Ldose: -1.397940009 (uM) Interaction | -8.422935252 | 19.0858749 | -0.441317744 | 0.658987412 |
| SKMEL2:Quizartinib:Ldose: -1.397940009 (uM) Interaction | -15.5423816 | 19.0858749 | -0.814339489 | 0.415459496 |
| UACC0257:Quizartinib:Ldose: -1.397940009 (uM) Interaction | -28.39307103 | 19.0858749 | -1.487648388 | 0.136858283 |
| MeWo:Sorafenib:Ldose: -1.397940009 (uM) Interaction | -4.086890609 | 19.0858749 | -0.214131688 | 0.830446392 |
| SKMEL2:Sorafenib:Ldose: -1.397940009 (uM) Interaction | -3.920054487 | 19.0858749 | -0.205390348 | 0.837269102 |
| UACC0257:Sorafenib:Ldose: -1.397940009 (uM) Interaction | -8.091970584 | 19.0858749 | -0.423976927 | 0.671586874 |
| MeWo:Carmustine:Ldose: -1.397940009 (uM) Interaction | -8.735996985 | 19.0858749 | -0.457720541 | 0.647157845 |
| SKMEL2:Carmustine:Ldose: -1.397940009 (uM) Interaction | -6.339126397 | 19.0858749 | -0.332137061 | 0.739788998 |
| UACC0257:Carmustine:Ldose: -1.397940009 (uM) Interaction | -12.41874881 | 19.0858749 | -0.650677471 | 0.515261649 |
| MeWo:Uracil.mustard:Ldose: -1.397940009 (uM) Interaction | -3.978516952 | 19.0858749 | -0.208453475 | 0.834876877 |
| SKMEL2:Uracil.mustard:Ldose: -1.397940009 (uM) Interaction | -6.324191976 | 19.0858749 | -0.331354576 | 0.740379896 |
| UACC0257:Uracil.mustard:Ldose: -1.397940009 (uM) Interaction | -17.07207021 | 19.0858749 | -0.894487169 | 0.371071286 |
| MeWo:Ixabepilone:Ldose: -1.397940009 (uM) Interaction | 7.507816665 | 19.0858749 | 0.393370317 | 0.694049883 |
| SKMEL2:Ixabepilone:Ldose: -1.397940009 (uM) Interaction | -4.788998492 | 19.0858749 | -0.250918468 | 0.801879539 |
| UACC0257:Ixabepilone:Ldose: -1.397940009 (uM) Interaction | -0.058628694 | 19.0858749 | -0.003071837 | 0.997549061 |
| MeWo:Valrubicin:Ldose: -1.397940009 (uM) Interaction | -12.36461715 | 19.0858749 | -0.647841255 | 0.517094534 |
| SKMEL2:Valrubicin:Ldose: -1.397940009 (uM) Interaction | -23.07496017 | 19.0858749 | -1.2090072 | 0.22667335 |
| UACC0257:Valrubicin:Ldose: -1.397940009 (uM) Interaction | -13.04037645 | 19.0858749 | -0.683247507 | 0.494457807 |
| MeWo:Triethylenemelamine:Ldose: -1.397940009 (uM) Interaction | -10.0342814 | 19.0858749 | -0.525743853 | 0.599071603 |
| SKMEL2:Triethylenemelamine:Ldose: -1.397940009 (uM) Interaction | -13.46770783 | 19.0858749 | -0.705637436 | 0.480421268 |
| UACC0257:Triethylenemelamine:Ldose: -1.397940009 (uM) Interaction | -17.22532393 | 19.0858749 | -0.902516862 | 0.36679246 |
| MeWo:Palbociclib..PD.0332991..Isethionate:Ldose: -1.397940009 (uM) Interaction | -8.892684242 | 19.0858749 | -0.465930134 | 0.641270222 |
| SKMEL2:Palbociclib..PD.0332991..Isethionate:Ldose: -1.397940009 (uM) Interaction | -13.06839592 | 19.0858749 | -0.684715581 | 0.493530782 |
| UACC0257:Palbociclib..PD.0332991..Isethionate:Ldose: -1.397940009 (uM) Interaction | -10.67492345 | 19.0858749 | -0.559310145 | 0.575955888 |
| MeWo:Afatinib:Ldose: -1.397940009 (uM) Interaction | -2.708748485 | 19.0858749 | -0.14192425 | 0.887141167 |
| SKMEL2:Afatinib:Ldose: -1.397940009 (uM) Interaction | -2.813353832 | 19.0858749 | -0.147405023 | 0.88281372 |
| UACC0257:Afatinib:Ldose: -1.397940009 (uM) Interaction | -15.02327064 | 19.0858749 | -0.78714079 | 0.431208114 |
| MeWo:Doxorubicin.HCl:Ldose: -1.397940009 (uM) Interaction | -5.825002905 | 19.0858749 | -0.30519968 | 0.760217025 |
| SKMEL2:Doxorubicin.HCl:Ldose: -1.397940009 (uM) Interaction | 0.385835863 | 19.0858749 | 0.020215781 | 0.983871426 |
| UACC0257:Doxorubicin.HCl:Ldose: -1.397940009 (uM) Interaction | -2.388256774 | 19.0858749 | -0.125132161 | 0.90042009 |
| MeWo:Exemestane:Ldose: -1.397940009 (uM) Interaction | -5.479948415 | 19.0858749 | -0.28712063 | 0.774022719 |
| SKMEL2:Exemestane:Ldose: -1.397940009 (uM) Interaction | -1.400376299 | 19.0858749 | -0.073372392 | 0.941510467 |
| UACC0257:Exemestane:Ldose: -1.397940009 (uM) Interaction | -16.33370164 | 19.0858749 | -0.855800519 | 0.392117648 |
| MeWo:Tretinoin:Ldose: -1.397940009 (uM) Interaction | -0.468341044 | 19.0858749 | -0.024538621 | 0.980423205 |
| SKMEL2:Tretinoin:Ldose: -1.397940009 (uM) Interaction | -7.570250731 | 19.0858749 | -0.396641536 | 0.691635748 |
| UACC0257:Tretinoin:Ldose: -1.397940009 (uM) Interaction | -5.412884719 | 19.0858749 | -0.283606843 | 0.776714418 |
| MeWo:Fulvestrant:Ldose: -1.397940009 (uM) Interaction | -15.71904517 | 19.0858749 | -0.823595735 | 0.410178418 |
| SKMEL2:Fulvestrant:Ldose: -1.397940009 (uM) Interaction | -19.21752549 | 19.0858749 | -1.006897802 | 0.313995199 |
| UACC0257:Fulvestrant:Ldose: -1.397940009 (uM) Interaction | -7.415716744 | 19.0858749 | -0.388544763 | 0.697616775 |
| MeWo:Docetaxel:Ldose: -1.397940009 (uM) Interaction | -8.064476804 | 19.0858749 | -0.422536397 | 0.672637758 |
| SKMEL2:Docetaxel:Ldose: -1.397940009 (uM) Interaction | -11.49798533 | 19.0858749 | -0.602434282 | 0.546891438 |
| UACC0257:Docetaxel:Ldose: -1.397940009 (uM) Interaction | -0.540995144 | 19.0858749 | -0.028345315 | 0.977387001 |
| MeWo:Everolimus:Ldose: -1.397940009 (uM) Interaction | -13.2597124 | 19.0858749 | -0.694739564 | 0.487226022 |
| SKMEL2:Everolimus:Ldose: -1.397940009 (uM) Interaction | -11.39746685 | 19.0858749 | -0.597167639 | 0.55040174 |
| UACC0257:Everolimus:Ldose: -1.397940009 (uM) Interaction | -19.01651667 | 19.0858749 | -0.996365992 | 0.319083543 |
| MeWo:MLN.2480:Ldose: -1.397940009 (uM) Interaction | -4.51115224 | 19.0858749 | -0.236360778 | 0.813154976 |
| SKMEL2:MLN.2480:Ldose: -1.397940009 (uM) Interaction | -4.897002954 | 19.0858749 | -0.256577337 | 0.797507522 |
| UACC0257:MLN.2480:Ldose: -1.397940009 (uM) Interaction | -18.72247278 | 19.0858749 | -0.98095963 | 0.326623667 |
| MeWo:LY2157299:Ldose: -1.397940009 (uM) Interaction | -9.448530419 | 19.0858749 | -0.495053566 | 0.62056736 |
| SKMEL2:LY2157299:Ldose: -1.397940009 (uM) Interaction | -5.425414891 | 19.0858749 | -0.284263358 | 0.776211297 |
| UACC0257:LY2157299:Ldose: -1.397940009 (uM) Interaction | -13.99454251 | 19.0858749 | -0.733240817 | 0.463419544 |
| MeWo:Allopurinol:Ldose: -1.397940009 (uM) Interaction | -13.23473571 | 19.0858749 | -0.693430916 | 0.488046643 |
| SKMEL2:Allopurinol:Ldose: -1.397940009 (uM) Interaction | -4.187532084 | 19.0858749 | -0.219404775 | 0.82633683 |
| UACC0257:Allopurinol:Ldose: -1.397940009 (uM) Interaction | -11.3602341 | 19.0858749 | -0.595216838 | 0.55170479 |
| MeWo:Pipobroman:Ldose: -1.397940009 (uM) Interaction | -6.712140879 | 19.0858749 | -0.351681069 | 0.7250809 |
| SKMEL2:Pipobroman:Ldose: -1.397940009 (uM) Interaction | -12.10853086 | 19.0858749 | -0.634423673 | 0.525811102 |
| UACC0257:Pipobroman:Ldose: -1.397940009 (uM) Interaction | -13.66141344 | 19.0858749 | -0.715786597 | 0.474130889 |
| MeWo:Letrozole:Ldose: -1.397940009 (uM) Interaction | -3.158924749 | 19.0858749 | -0.165511131 | 0.868543242 |
| SKMEL2:Letrozole:Ldose: -1.397940009 (uM) Interaction | -5.002401871 | 19.0858749 | -0.262099689 | 0.793247093 |
| UACC0257:Letrozole:Ldose: -1.397940009 (uM) Interaction | -15.50909179 | 19.0858749 | -0.812595277 | 0.416459121 |
| MeWo:Thiotepa:Ldose: -1.397940009 (uM) Interaction | -15.95610738 | 19.0858749 | -0.836016555 | 0.403154863 |
| SKMEL2:Thiotepa:Ldose: -1.397940009 (uM) Interaction | -19.01777717 | 19.0858749 | -0.996432035 | 0.319051467 |
| UACC0257:Thiotepa:Ldose: -1.397940009 (uM) Interaction | -8.408065909 | 19.0858749 | -0.440538668 | 0.659551433 |
| MeWo:Plicamycin:Ldose: -1.397940009 (uM) Interaction | -4.026010177 | 19.0858749 | -0.210941872 | 0.832934626 |
| SKMEL2:Plicamycin:Ldose: -1.397940009 (uM) Interaction | -18.33572365 | 19.0858749 | -0.960695999 | 0.336715827 |
| UACC0257:Plicamycin:Ldose: -1.397940009 (uM) Interaction | 7.280560215 | 19.0858749 | 0.381463268 | 0.702863285 |
| MeWo:Erlotinib.HCl:Ldose: -1.397940009 (uM) Interaction | -19.12263338 | 19.0858749 | -1.001925952 | 0.316390612 |
| SKMEL2:Erlotinib.HCl:Ldose: -1.397940009 (uM) Interaction | -21.58652244 | 19.0858749 | -1.13102085 | 0.258058908 |
| UACC0257:Erlotinib.HCl:Ldose: -1.397940009 (uM) Interaction | -22.0129143 | 19.0858749 | -1.153361553 | 0.248774813 |
| MeWo:MEK.162..ARRY.438162.:Ldose: -1.397940009 (uM) Interaction | 6.233831498 | 19.0858749 | 0.326620159 | 0.743958368 |
| SKMEL2:MEK.162..ARRY.438162.:Ldose: -1.397940009 (uM) Interaction | 2.820521551 | 19.0858749 | 0.147780574 | 0.882517324 |
| UACC0257:MEK.162..ARRY.438162.:Ldose: -1.397940009 (uM) Interaction | -0.033894557 | 19.0858749 | -0.001775897 | 0.998583056 |
| MeWo:Baricitinib..LY3009104..INCB028050.:Ldose: -1.397940009 (uM) Interaction | -1.393268569 | 19.0858749 | -0.072999984 | 0.941806808 |
| SKMEL2:Baricitinib..LY3009104..INCB028050.:Ldose: -1.397940009 (uM) Interaction | -3.263755342 | 19.0858749 | -0.171003706 | 0.864222458 |
| UACC0257:Baricitinib..LY3009104..INCB028050.:Ldose: -1.397940009 (uM) Interaction | -18.67615229 | 19.0858749 | -0.978532679 | 0.327821927 |
| MeWo:Arsenic.Trioxide:Ldose: -1.397940009 (uM) Interaction | -6.357423469 | 19.0858749 | -0.333095732 | 0.739065263 |
| SKMEL2:Arsenic.Trioxide:Ldose: -1.397940009 (uM) Interaction | 5.175291921 | 19.0858749 | 0.271158223 | 0.786271941 |
| UACC0257:Arsenic.Trioxide:Ldose: -1.397940009 (uM) Interaction | -2.657430157 | 19.0858749 | -0.139235438 | 0.889265407 |
| MeWo:Celecoxib:Ldose: -1.397940009 (uM) Interaction | 0.097639115 | 19.0858749 | 0.005115779 | 0.995918264 |
| SKMEL2:Celecoxib:Ldose: -1.397940009 (uM) Interaction | -1.393990249 | 19.0858749 | -0.073037797 | 0.941776719 |
| UACC0257:Celecoxib:Ldose: -1.397940009 (uM) Interaction | -16.87529391 | 19.0858749 | -0.884177121 | 0.376610476 |
| MeWo:Bendamustine.HCl:Ldose: -1.397940009 (uM) Interaction | -8.188914025 | 19.0858749 | -0.429056256 | 0.667886576 |
| SKMEL2:Bendamustine.HCl:Ldose: -1.397940009 (uM) Interaction | -4.292525429 | 19.0858749 | -0.224905877 | 0.822054631 |
| UACC0257:Bendamustine.HCl:Ldose: -1.397940009 (uM) Interaction | -18.07484768 | 19.0858749 | -0.947027463 | 0.343635409 |
| MeWo:Chlorambucil:Ldose: -1.397940009 (uM) Interaction | -16.84738722 | 19.0858749 | -0.882714956 | 0.37740015 |
| SKMEL2:Chlorambucil:Ldose: -1.397940009 (uM) Interaction | -10.94360789 | 19.0858749 | -0.573387804 | 0.566388133 |
| UACC0257:Chlorambucil:Ldose: -1.397940009 (uM) Interaction | -26.96987153 | 19.0858749 | -1.41308018 | 0.1576466 |
| MeWo:Zoledronic.Acid:Ldose: -1.397940009 (uM) Interaction | -10.46740937 | 19.0858749 | -0.548437492 | 0.583397222 |
| SKMEL2:Zoledronic.Acid:Ldose: -1.397940009 (uM) Interaction | -13.02496319 | 19.0858749 | -0.682439933 | 0.494968151 |
| UACC0257:Zoledronic.Acid:Ldose: -1.397940009 (uM) Interaction | -13.48034183 | 19.0858749 | -0.706299392 | 0.480009611 |
| MeWo:Actinomycin.D:Ldose: -1.397940009 (uM) Interaction | -3.962744364 | 19.0858749 | -0.207627074 | 0.835522126 |
| SKMEL2:Actinomycin.D:Ldose: -1.397940009 (uM) Interaction | -42.0744664 | 19.0858749 | -2.204481934 | 0.027501002 |
| UACC0257:Actinomycin.D:Ldose: -1.397940009 (uM) Interaction | -8.29440216 | 19.0858749 | -0.434583282 | 0.663869285 |
| MeWo:Temsirolimus..CCI.779..Torisel.:Ldose: -1.397940009 (uM) Interaction | -9.979448245 | 19.0858749 | -0.522870882 | 0.601069487 |
| SKMEL2:Temsirolimus..CCI.779..Torisel.:Ldose: -1.397940009 (uM) Interaction | -6.549630761 | 19.0858749 | -0.343166389 | 0.731476638 |
| UACC0257:Temsirolimus..CCI.779..Torisel.:Ldose: -1.397940009 (uM) Interaction | -6.172210959 | 19.0858749 | -0.323391565 | 0.746401856 |
| MeWo:Foretinib..GSK1363089.:Ldose: -1.397940009 (uM) Interaction | -15.37170316 | 19.0858749 | -0.805396831 | 0.42059961 |
| SKMEL2:Foretinib..GSK1363089.:Ldose: -1.397940009 (uM) Interaction | -2.967565507 | 19.0858749 | -0.155484908 | 0.876440508 |
| UACC0257:Foretinib..GSK1363089.:Ldose: -1.397940009 (uM) Interaction | -12.11358924 | 19.0858749 | -0.634688706 | 0.525638202 |
| MeWo:Decitabine:Ldose: -1.397940009 (uM) Interaction | -6.816593974 | 19.0858749 | -0.357153864 | 0.720980121 |
| SKMEL2:Decitabine:Ldose: -1.397940009 (uM) Interaction | -7.540271599 | 19.0858749 | -0.395070786 | 0.69279456 |
| UACC0257:Decitabine:Ldose: -1.397940009 (uM) Interaction | -12.61991243 | 19.0858749 | -0.661217392 | 0.508479981 |
| MeWo:Methotrexate:Ldose: -1.397940009 (uM) Interaction | 25.8252295 | 19.0858749 | 1.353106925 | 0.176035674 |
| SKMEL2:Methotrexate:Ldose: -1.397940009 (uM) Interaction | 18.00067866 | 19.0858749 | 0.943141394 | 0.345619163 |
| UACC0257:Methotrexate:Ldose: -1.397940009 (uM) Interaction | 9.299223956 | 19.0858749 | 0.487230688 | 0.626099818 |
| MeWo:Axitinib:Ldose: -1.397940009 (uM) Interaction | -0.645868242 | 19.0858749 | -0.033840117 | 0.973004959 |
| SKMEL2:Axitinib:Ldose: -1.397940009 (uM) Interaction | -7.301072397 | 19.0858749 | -0.382537999 | 0.702066123 |
| UACC0257:Axitinib:Ldose: -1.397940009 (uM) Interaction | -17.80501472 | 19.0858749 | -0.932889627 | 0.350887415 |
| MeWo:Oxaliplatin:Ldose: -1.397940009 (uM) Interaction | -7.787571119 | 19.0858749 | -0.408027987 | 0.683257183 |
| SKMEL2:Oxaliplatin:Ldose: -1.397940009 (uM) Interaction | -5.486317667 | 19.0858749 | -0.287454345 | 0.77376722 |
| UACC0257:Oxaliplatin:Ldose: -1.397940009 (uM) Interaction | -16.75112503 | 19.0858749 | -0.877671321 | 0.380131902 |
| MeWo:Cabazitaxel:Ldose: -1.397940009 (uM) Interaction | -10.24882089 | 19.0858749 | -0.5369846 | 0.591283798 |
| SKMEL2:Cabazitaxel:Ldose: -1.397940009 (uM) Interaction | -12.28816119 | 19.0858749 | -0.643835363 | 0.519689055 |
| UACC0257:Cabazitaxel:Ldose: -1.397940009 (uM) Interaction | 6.02933681 | 19.0858749 | 0.315905707 | 0.752077141 |
| MeWo:Amifostine:Ldose: -1.397940009 (uM) Interaction | -10.30573932 | 19.0858749 | -0.539966828 | 0.589225492 |
| SKMEL2:Amifostine:Ldose: -1.397940009 (uM) Interaction | -12.65017092 | 19.0858749 | -0.662802779 | 0.507463966 |
| UACC0257:Amifostine:Ldose: -1.397940009 (uM) Interaction | -22.80356136 | 19.0858749 | -1.194787322 | 0.23218329 |
| MeWo:Flutamide..Eulexin.:Ldose: -1.397940009 (uM) Interaction | -7.598947685 | 19.0858749 | -0.398145106 | 0.690527173 |
| SKMEL2:Flutamide..Eulexin.:Ldose: -1.397940009 (uM) Interaction | -9.824899021 | 19.0858749 | -0.514773311 | 0.606716719 |
| UACC0257:Flutamide..Eulexin.:Ldose: -1.397940009 (uM) Interaction | -25.52143429 | 19.0858749 | -1.337189646 | 0.181174861 |
| MeWo:LDK378:Ldose: -1.397940009 (uM) Interaction | 0.268427181 | 19.0858749 | 0.01406418 | 0.988778908 |
| SKMEL2:LDK378:Ldose: -1.397940009 (uM) Interaction | 0.195601193 | 19.0858749 | 0.010248479 | 0.991823135 |
| UACC0257:LDK378:Ldose: -1.397940009 (uM) Interaction | -16.19390259 | 19.0858749 | -0.84847578 | 0.396182484 |
| MeWo:Pralatrexate:Ldose: -1.397940009 (uM) Interaction | -5.524628056 | 19.0858749 | -0.289461609 | 0.772230941 |
| SKMEL2:Pralatrexate:Ldose: -1.397940009 (uM) Interaction | -4.882680534 | 19.0858749 | -0.255826917 | 0.798086931 |
| UACC0257:Pralatrexate:Ldose: -1.397940009 (uM) Interaction | -11.97981774 | 19.0858749 | -0.627679779 | 0.530220388 |
| MeWo:Topotecan.HCl:Ldose: -1.397940009 (uM) Interaction | 8.43914692 | 19.0858749 | 0.442167151 | 0.658372694 |
| SKMEL2:Topotecan.HCl:Ldose: -1.397940009 (uM) Interaction | 3.207310344 | 19.0858749 | 0.168046284 | 0.866548445 |
| UACC0257:Topotecan.HCl:Ldose: -1.397940009 (uM) Interaction | -0.00379972 | 19.0858749 | -0.000199085 | 0.999841155 |
| MeWo:Pemetrexed:Ldose: -1.397940009 (uM) Interaction | -7.892329785 | 19.0858749 | -0.413516793 | 0.679232138 |
| SKMEL2:Pemetrexed:Ldose: -1.397940009 (uM) Interaction | -6.246525432 | 19.0858749 | -0.327285255 | 0.743455324 |
| UACC0257:Pemetrexed:Ldose: -1.397940009 (uM) Interaction | -6.61540776 | 19.0858749 | -0.346612759 | 0.728885645 |
| MeWo:Bleomycin.Sulfate:Ldose: -1.397940009 (uM) Interaction | -12.98442185 | 19.0858749 | -0.680315779 | 0.496311847 |
| SKMEL2:Bleomycin.Sulfate:Ldose: -1.397940009 (uM) Interaction | -15.8598718 | 19.0858749 | -0.830974314 | 0.405997351 |
| UACC0257:Bleomycin.Sulfate:Ldose: -1.397940009 (uM) Interaction | -11.81417086 | 19.0858749 | -0.619000749 | 0.535922412 |
| MeWo:Axitinib.1:Ldose: -1.397940009 (uM) Interaction | -3.271256585 | 19.0858749 | -0.171396732 | 0.863913434 |
| SKMEL2:Axitinib.1:Ldose: -1.397940009 (uM) Interaction | -2.849705529 | 19.0858749 | -0.149309662 | 0.881310689 |
| UACC0257:Axitinib.1:Ldose: -1.397940009 (uM) Interaction | -19.11892394 | 19.0858749 | -1.001731597 | 0.316484495 |
| MeWo:Ibrutinib..PCI.32765.:Ldose: -1.397940009 (uM) Interaction | -6.622990435 | 19.0858749 | -0.347010052 | 0.728587157 |
| SKMEL2:Ibrutinib..PCI.32765.:Ldose: -1.397940009 (uM) Interaction | -6.066842167 | 19.0858749 | -0.317870792 | 0.75058603 |
| UACC0257:Ibrutinib..PCI.32765.:Ldose: -1.397940009 (uM) Interaction | -9.40346416 | 19.0858749 | -0.492692329 | 0.62223502 |
| MeWo:Tamoxifen.Citrate:Ldose: -1.397940009 (uM) Interaction | -4.027997435 | 19.0858749 | -0.211045994 | 0.832853378 |
| SKMEL2:Tamoxifen.Citrate:Ldose: -1.397940009 (uM) Interaction | -0.293124862 | 19.0858749 | -0.015358209 | 0.987746546 |
| UACC0257:Tamoxifen.Citrate:Ldose: -1.397940009 (uM) Interaction | -13.67881298 | 19.0858749 | -0.716698242 | 0.473568082 |
| MeWo:Vemurafenib:Ldose: -1.397940009 (uM) Interaction | 4.797398876 | 19.0858749 | 0.251358604 | 0.801539267 |
| SKMEL2:Vemurafenib:Ldose: -1.397940009 (uM) Interaction | 4.681352878 | 19.0858749 | 0.245278401 | 0.806243211 |
| UACC0257:Vemurafenib:Ldose: -1.397940009 (uM) Interaction | -0.403268409 | 19.0858749 | -0.021129155 | 0.983142823 |
| MeWo:Pazopanib.HCl:Ldose: -1.397940009 (uM) Interaction | -7.177939969 | 19.0858749 | -0.376086504 | 0.706856291 |
| SKMEL2:Pazopanib.HCl:Ldose: -1.397940009 (uM) Interaction | -4.729651452 | 19.0858749 | -0.247808994 | 0.804284558 |
| UACC0257:Pazopanib.HCl:Ldose: -1.397940009 (uM) Interaction | -1.685770027 | 19.0858749 | -0.088325531 | 0.929618769 |
| MeWo:Abiraterone:Ldose: -1.397940009 (uM) Interaction | -9.858220835 | 19.0858749 | -0.5165192 | 0.605497135 |
| SKMEL2:Abiraterone:Ldose: -1.397940009 (uM) Interaction | -8.244917991 | 19.0858749 | -0.431990571 | 0.66575259 |
| UACC0257:Abiraterone:Ldose: -1.397940009 (uM) Interaction | -15.70481039 | 19.0858749 | -0.822849908 | 0.410602461 |
| MeWo:Bosutinib..SKI.606.:Ldose: -1.397940009 (uM) Interaction | -0.932141252 | 19.0858749 | -0.048839325 | 0.961047795 |
| SKMEL2:Bosutinib..SKI.606.:Ldose: -1.397940009 (uM) Interaction | -3.724495059 | 19.0858749 | -0.195144057 | 0.845282014 |
| UACC0257:Bosutinib..SKI.606.:Ldose: -1.397940009 (uM) Interaction | -10.31276814 | 19.0858749 | -0.540335101 | 0.588971542 |
| MeWo:Sabutoclax..BI.97C1.:Ldose: -1.397940009 (uM) Interaction | -3.798675299 | 19.0858749 | -0.199030714 | 0.842240598 |
| SKMEL2:Sabutoclax..BI.97C1.:Ldose: -1.397940009 (uM) Interaction | -5.935289484 | 19.0858749 | -0.310978119 | 0.755820265 |
| UACC0257:Sabutoclax..BI.97C1.:Ldose: -1.397940009 (uM) Interaction | -3.13655504 | 19.0858749 | -0.164339076 | 0.869465764 |
| MeWo:Thioguanine:Ldose: -1 (uM) Interaction | -30.20049874 | 19.0858749 | -1.582348145 | 0.113584797 |
| SKMEL2:Thioguanine:Ldose: -1 (uM) Interaction | -51.35747338 | 19.0858749 | -2.690862937 | 0.007132246 |
| UACC0257:Thioguanine:Ldose: -1 (uM) Interaction | -48.85603233 | 19.0858749 | -2.559800512 | 0.010479989 |
| MeWo:Irinotecan.HCl:Ldose: -1 (uM) Interaction | -25.98888035 | 19.0858749 | -1.361681374 | 0.173312703 |
| SKMEL2:Irinotecan.HCl:Ldose: -1 (uM) Interaction | -44.01299612 | 19.0858749 | -2.30605075 | 0.021117245 |
| UACC0257:Irinotecan.HCl:Ldose: -1 (uM) Interaction | -40.81222775 | 19.0858749 | -2.138347232 | 0.032499789 |
| MeWo:Romidepsin:Ldose: -1 (uM) Interaction | -27.6388964 | 19.0858749 | -1.448133583 | 0.14759425 |
| SKMEL2:Romidepsin:Ldose: -1 (uM) Interaction | -37.99300374 | 19.0858749 | -1.990634642 | 0.046533683 |
| UACC0257:Romidepsin:Ldose: -1 (uM) Interaction | -43.97421183 | 19.0858749 | -2.304018656 | 0.021231066 |
| MeWo:Paclitaxel:Ldose: -1 (uM) Interaction | -25.22547961 | 19.0858749 | -1.321683169 | 0.186287712 |
| SKMEL2:Paclitaxel:Ldose: -1 (uM) Interaction | -45.90016547 | 19.0858749 | -2.404928551 | 0.01618401 |
| UACC0257:Paclitaxel:Ldose: -1 (uM) Interaction | -36.2815071 | 19.0858749 | -1.900961171 | 0.057320433 |
| MeWo:Alisertib..MLN8237.:Ldose: -1 (uM) Interaction | -31.24954152 | 19.0858749 | -1.637312499 | 0.101579798 |
| SKMEL2:Alisertib..MLN8237.:Ldose: -1 (uM) Interaction | -58.73098688 | 19.0858749 | -3.077196471 | 0.002092197 |
| UACC0257:Alisertib..MLN8237.:Ldose: -1 (uM) Interaction | -53.18351065 | 19.0858749 | -2.786537738 | 0.005332102 |
| MeWo:Vorinostat:Ldose: -1 (uM) Interaction | -35.52809596 | 19.0858749 | -1.861486369 | 0.062689135 |
| SKMEL2:Vorinostat:Ldose: -1 (uM) Interaction | -47.49384322 | 19.0858749 | -2.488428929 | 0.012838384 |
| UACC0257:Vorinostat:Ldose: -1 (uM) Interaction | -30.88285986 | 19.0858749 | -1.618100298 | 0.105655626 |
| MeWo:Busulfan:Ldose: -1 (uM) Interaction | -31.03954633 | 19.0858749 | -1.626309849 | 0.103898386 |
| SKMEL2:Busulfan:Ldose: -1 (uM) Interaction | -45.03164997 | 19.0858749 | -2.359422883 | 0.018312256 |
| UACC0257:Busulfan:Ldose: -1 (uM) Interaction | -24.45043503 | 19.0858749 | -1.281074887 | 0.200181156 |
| MeWo:Mechlorethamine.HCl:Ldose: -1 (uM) Interaction | -37.51139336 | 19.0858749 | -1.965400777 | 0.049380698 |
| SKMEL2:Mechlorethamine.HCl:Ldose: -1 (uM) Interaction | -59.03897106 | 19.0858749 | -3.093333231 | 0.001981749 |
| UACC0257:Mechlorethamine.HCl:Ldose: -1 (uM) Interaction | -47.53443937 | 19.0858749 | -2.490555954 | 0.012761815 |
| MeWo:Teniposide:Ldose: -1 (uM) Interaction | -33.94398461 | 19.0858749 | -1.778487221 | 0.07533797 |
| SKMEL2:Teniposide:Ldose: -1 (uM) Interaction | -39.49551522 | 19.0858749 | -2.069358383 | 0.038524365 |
| UACC0257:Teniposide:Ldose: -1 (uM) Interaction | -37.16310066 | 19.0858749 | -1.947152062 | 0.051529485 |
| MeWo:Vinorelbine.Tartrate:Ldose: -1 (uM) Interaction | -41.49601395 | 19.0858749 | -2.174174051 | 0.029702916 |
| SKMEL2:Vinorelbine.Tartrate:Ldose: -1 (uM) Interaction | -62.74763793 | 19.0858749 | -3.287647974 | 0.001011898 |
| UACC0257:Vinorelbine.Tartrate:Ldose: -1 (uM) Interaction | -56.48521786 | 19.0858749 | -2.959529923 | 0.003084441 |
| MeWo:Cabozantinib..XL.184.:Ldose: -1 (uM) Interaction | -35.89635566 | 19.0858749 | -1.880781251 | 0.060015161 |
| SKMEL2:Cabozantinib..XL.184.:Ldose: -1 (uM) Interaction | -44.67835987 | 19.0858749 | -2.34091233 | 0.019245755 |
| UACC0257:Cabozantinib..XL.184.:Ldose: -1 (uM) Interaction | -51.93266168 | 19.0858749 | -2.720999795 | 0.006513707 |
| MeWo:Dacarbazine:Ldose: -1 (uM) Interaction | -33.98147691 | 19.0858749 | -1.780451622 | 0.075016171 |
| SKMEL2:Dacarbazine:Ldose: -1 (uM) Interaction | -42.06431946 | 19.0858749 | -2.203950287 | 0.027538379 |
| UACC0257:Dacarbazine:Ldose: -1 (uM) Interaction | -53.30679218 | 19.0858749 | -2.792997045 | 0.005226824 |
| MeWo:Clofarabine:Ldose: -1 (uM) Interaction | 17.81610216 | 19.0858749 | 0.933470551 | 0.350587532 |
| SKMEL2:Clofarabine:Ldose: -1 (uM) Interaction | -1.280720497 | 19.0858749 | -0.067103054 | 0.946500285 |
| UACC0257:Clofarabine:Ldose: -1 (uM) Interaction | -9.176253744 | 19.0858749 | -0.480787692 | 0.630672282 |
| MeWo:Cisplatin:Ldose: -1 (uM) Interaction | -29.29010686 | 19.0858749 | -1.534648373 | 0.124884911 |
| SKMEL2:Cisplatin:Ldose: -1 (uM) Interaction | -48.97247162 | 19.0858749 | -2.565901321 | 0.010297511 |
| UACC0257:Cisplatin:Ldose: -1 (uM) Interaction | -63.6466535 | 19.0858749 | -3.334751686 | 0.000855197 |
| MeWo:Floxuridine:Ldose: -1 (uM) Interaction | -6.032910455 | 19.0858749 | -0.316092948 | 0.751935022 |
| SKMEL2:Floxuridine:Ldose: -1 (uM) Interaction | -13.60304102 | 19.0858749 | -0.712728187 | 0.476021691 |
| UACC0257:Floxuridine:Ldose: -1 (uM) Interaction | -42.95692425 | 19.0858749 | -2.250718109 | 0.02441344 |
| MeWo:Lomustine..CCNU.:Ldose: -1 (uM) Interaction | -33.75976513 | 19.0858749 | -1.768835084 | 0.076935549 |
| SKMEL2:Lomustine..CCNU.:Ldose: -1 (uM) Interaction | -55.31052116 | 19.0858749 | -2.897981961 | 0.003759508 |
| UACC0257:Lomustine..CCNU.:Ldose: -1 (uM) Interaction | -46.11893215 | 19.0858749 | -2.416390781 | 0.015683487 |
| MeWo:Melphalan:Ldose: -1 (uM) Interaction | -27.57663088 | 19.0858749 | -1.444871195 | 0.148508615 |
| SKMEL2:Melphalan:Ldose: -1 (uM) Interaction | -52.71886548 | 19.0858749 | -2.762192761 | 0.005746323 |
| UACC0257:Melphalan:Ldose: -1 (uM) Interaction | -49.95041971 | 19.0858749 | -2.617140686 | 0.008873174 |
| MeWo:BGJ398..NVPBGJ398.:Ldose: -1 (uM) Interaction | -28.57095819 | 19.0858749 | -1.496968745 | 0.134416043 |
| SKMEL2:BGJ398..NVPBGJ398.:Ldose: -1 (uM) Interaction | -52.74505036 | 19.0858749 | -2.763564712 | 0.00572223 |
| UACC0257:BGJ398..NVPBGJ398.:Ldose: -1 (uM) Interaction | -70.07780338 | 19.0858749 | -3.671710297 | 0.000241515 |
| MeWo:Navitoclax..ABT.263..5uM:Ldose: -1 (uM) Interaction | -40.98178715 | 19.0858749 | -2.147231258 | 0.031786002 |
| SKMEL2:Navitoclax..ABT.263..5uM:Ldose: -1 (uM) Interaction | -59.03218421 | 19.0858749 | -3.092977636 | 0.001984124 |
| UACC0257:Navitoclax..ABT.263..5uM:Ldose: -1 (uM) Interaction | -46.59139725 | 19.0858749 | -2.441145481 | 0.01464875 |
| MeWo:Azacitidine:Ldose: -1 (uM) Interaction | -40.06417728 | 19.0858749 | -2.0991533 | 0.035814997 |
| SKMEL2:Azacitidine:Ldose: -1 (uM) Interaction | -52.17995379 | 19.0858749 | -2.733956608 | 0.006262941 |
| UACC0257:Azacitidine:Ldose: -1 (uM) Interaction | -50.31982633 | 19.0858749 | -2.63649566 | 0.008382735 |
| MeWo:Capecitabine:Ldose: -1 (uM) Interaction | -32.96026854 | 19.0858749 | -1.726945645 | 0.084191762 |
| SKMEL2:Capecitabine:Ldose: -1 (uM) Interaction | -48.03852793 | 19.0858749 | -2.516967558 | 0.011844173 |
| UACC0257:Capecitabine:Ldose: -1 (uM) Interaction | -56.93759188 | 19.0858749 | -2.983231955 | 0.002855413 |
| MeWo:Megestrol.acetate:Ldose: -1 (uM) Interaction | -28.59494778 | 19.0858749 | -1.498225674 | 0.134089281 |
| SKMEL2:Megestrol.acetate:Ldose: -1 (uM) Interaction | -49.65273782 | 19.0858749 | -2.601543712 | 0.009286871 |
| UACC0257:Megestrol.acetate:Ldose: -1 (uM) Interaction | -69.95001526 | 19.0858749 | -3.665014868 | 0.000247918 |
| MeWo:Cytarabine.HCl...Ara.C:Ldose: -1 (uM) Interaction | -12.33680452 | 19.0858749 | -0.646384019 | 0.518037574 |
| SKMEL2:Cytarabine.HCl...Ara.C:Ldose: -1 (uM) Interaction | -39.72518525 | 19.0858749 | -2.081391892 | 0.037409831 |
| UACC0257:Cytarabine.HCl...Ara.C:Ldose: -1 (uM) Interaction | -57.42061697 | 19.0858749 | -3.008539943 | 0.002628096 |
| MeWo:Gemcitabine.HCl:Ldose: -1 (uM) Interaction | -8.788688129 | 19.0858749 | -0.460481281 | 0.645175453 |
| SKMEL2:Gemcitabine.HCl:Ldose: -1 (uM) Interaction | -5.338423474 | 19.0858749 | -0.279705463 | 0.779706172 |
| UACC0257:Gemcitabine.HCl:Ldose: -1 (uM) Interaction | -67.5747045 | 19.0858749 | -3.54056101 | 0.00040012 |
| MeWo:Vinblastine.Sulfate:Ldose: -1 (uM) Interaction | -43.93622838 | 19.0858749 | -2.302028522 | 0.021343055 |
| SKMEL2:Vinblastine.Sulfate:Ldose: -1 (uM) Interaction | -51.43619796 | 19.0858749 | -2.694987693 | 0.007044584 |
| UACC0257:Vinblastine.Sulfate:Ldose: -1 (uM) Interaction | -40.5022996 | 19.0858749 | -2.122108618 | 0.033839998 |
| MeWo:MLN9708..MLN2238.:Ldose: -1 (uM) Interaction | -84.63869656 | 19.0858749 | -4.434624926 | 9.27E-06 |
| SKMEL2:MLN9708..MLN2238.:Ldose: -1 (uM) Interaction | -98.4650796 | 19.0858749 | -5.159055067 | 2.50E-07 |
| UACC0257:MLN9708..MLN2238.:Ldose: -1 (uM) Interaction | -89.91471517 | 19.0858749 | -4.711060701 | 2.48E-06 |
| MeWo:ABT.737:Ldose: -1 (uM) Interaction | -45.71070521 | 19.0858749 | -2.395001825 | 0.016628771 |
| SKMEL2:ABT.737:Ldose: -1 (uM) Interaction | -63.27806334 | 19.0858749 | -3.31543949 | 0.000916503 |
| UACC0257:ABT.737:Ldose: -1 (uM) Interaction | -63.87991943 | 19.0858749 | -3.346973601 | 0.000818388 |
| MeWo:Streptozocin:Ldose: -1 (uM) Interaction | -36.18776343 | 19.0858749 | -1.896049493 | 0.05796687 |
| SKMEL2:Streptozocin:Ldose: -1 (uM) Interaction | -47.90344897 | 19.0858749 | -2.509890127 | 0.012084146 |
| UACC0257:Streptozocin:Ldose: -1 (uM) Interaction | -50.67220171 | 19.0858749 | -2.654958287 | 0.007937654 |
| MeWo:Crizotinib:Ldose: -1 (uM) Interaction | -35.58048506 | 19.0858749 | -1.864231284 | 0.062302825 |
| SKMEL2:Crizotinib:Ldose: -1 (uM) Interaction | -48.59549733 | 19.0858749 | -2.546149841 | 0.010898743 |
| UACC0257:Crizotinib:Ldose: -1 (uM) Interaction | -71.77445533 | 19.0858749 | -3.760605983 | 0.000169951 |
| MeWo:Sunitinib:Ldose: -1 (uM) Interaction | -39.79980091 | 19.0858749 | -2.085301362 | 0.037053697 |
| SKMEL2:Sunitinib:Ldose: -1 (uM) Interaction | -62.96262562 | 19.0858749 | -3.298912204 | 0.000972176 |
| UACC0257:Sunitinib:Ldose: -1 (uM) Interaction | -66.0354207 | 19.0858749 | -3.459910591 | 0.000541401 |
| MeWo:Dexrazoxane:Ldose: -1 (uM) Interaction | -34.54444677 | 19.0858749 | -1.809948297 | 0.070317736 |
| SKMEL2:Dexrazoxane:Ldose: -1 (uM) Interaction | -60.4934698 | 19.0858749 | -3.169541356 | 0.001528933 |
| UACC0257:Dexrazoxane:Ldose: -1 (uM) Interaction | -64.35463099 | 19.0858749 | -3.371846003 | 0.000747975 |
| MeWo:Mitomycin.C:Ldose: -1 (uM) Interaction | -26.81240672 | 19.0858749 | -1.404829847 | 0.160086302 |
| SKMEL2:Mitomycin.C:Ldose: -1 (uM) Interaction | -56.2394516 | 19.0858749 | -2.946653057 | 0.003215775 |
| UACC0257:Mitomycin.C:Ldose: -1 (uM) Interaction | -69.24393745 | 19.0858749 | -3.628020084 | 0.000286263 |
| MeWo:Carfilzomib:Ldose: -1 (uM) Interaction | -69.96339768 | 19.0858749 | -3.665716037 | 0.00024724 |
| SKMEL2:Carfilzomib:Ldose: -1 (uM) Interaction | 4.782720364 | 19.0858749 | 0.250589527 | 0.80213387 |
| UACC0257:Carfilzomib:Ldose: -1 (uM) Interaction | -50.33448787 | 19.0858749 | -2.637263848 | 0.00836378 |
| MeWo:OSI.027:Ldose: -1 (uM) Interaction | -48.02102208 | 19.0858749 | -2.516050343 | 0.011875032 |
| SKMEL2:OSI.027:Ldose: -1 (uM) Interaction | -64.24131708 | 19.0858749 | -3.365908947 | 0.000764252 |
| UACC0257:OSI.027:Ldose: -1 (uM) Interaction | -67.43004131 | 19.0858749 | -3.532981416 | 0.000411761 |
| MeWo:Bioymifi:Ldose: -1 (uM) Interaction | -41.11831917 | 19.0858749 | -2.154384821 | 0.031221059 |
| SKMEL2:Bioymifi:Ldose: -1 (uM) Interaction | -60.78105655 | 19.0858749 | -3.184609397 | 0.001451558 |
| UACC0257:Bioymifi:Ldose: -1 (uM) Interaction | -77.25624404 | 19.0858749 | -4.047823035 | 5.19E-05 |
| MeWo:Nelarabine:Ldose: -1 (uM) Interaction | -44.4778305 | 19.0858749 | -2.33040564 | 0.019793907 |
| SKMEL2:Nelarabine:Ldose: -1 (uM) Interaction | -52.10478678 | 19.0858749 | -2.73001825 | 0.006338228 |
| UACC0257:Nelarabine:Ldose: -1 (uM) Interaction | -55.46765704 | 19.0858749 | -2.906215059 | 0.003662026 |
| MeWo:Raloxifene:Ldose: -1 (uM) Interaction | -35.64898209 | 19.0858749 | -1.86782017 | 0.061800709 |
| SKMEL2:Raloxifene:Ldose: -1 (uM) Interaction | -45.35444953 | 19.0858749 | -2.376335891 | 0.017494227 |
| UACC0257:Raloxifene:Ldose: -1 (uM) Interaction | -72.21512446 | 19.0858749 | -3.78369474 | 0.000154935 |
| MeWo:Quinacrine.HCl:Ldose: -1 (uM) Interaction | -41.25283886 | 19.0858749 | -2.161432949 | 0.030672892 |
| SKMEL2:Quinacrine.HCl:Ldose: -1 (uM) Interaction | -59.40252899 | 19.0858749 | -3.112381765 | 0.001858271 |
| UACC0257:Quinacrine.HCl:Ldose: -1 (uM) Interaction | -57.86528163 | 19.0858749 | -3.031838045 | 0.002433585 |
| MeWo:Lenalidomide:Ldose: -1 (uM) Interaction | -39.28293105 | 19.0858749 | -2.058220085 | 0.039581014 |
| SKMEL2:Lenalidomide:Ldose: -1 (uM) Interaction | -62.5379732 | 19.0858749 | -3.276662639 | 0.001052079 |
| UACC0257:Lenalidomide:Ldose: -1 (uM) Interaction | -59.43094367 | 19.0858749 | -3.113870545 | 0.001848925 |
| MeWo:Fludarabine.Phosphate:Ldose: -1 (uM) Interaction | -37.166568 | 19.0858749 | -1.947333732 | 0.051507714 |
| SKMEL2:Fludarabine.Phosphate:Ldose: -1 (uM) Interaction | -56.52540096 | 19.0858749 | -2.961635307 | 0.003063439 |
| UACC0257:Fludarabine.Phosphate:Ldose: -1 (uM) Interaction | -61.57358762 | 19.0858749 | -3.226133879 | 0.00125662 |
| MeWo:Nilotinib:Ldose: -1 (uM) Interaction | -38.5552792 | 19.0858749 | -2.020094934 | 0.043385895 |
| SKMEL2:Nilotinib:Ldose: -1 (uM) Interaction | -57.52728079 | 19.0858749 | -3.014128569 | 0.002580184 |
| UACC0257:Nilotinib:Ldose: -1 (uM) Interaction | -52.8560803 | 19.0858749 | -2.7693821 | 0.005621078 |
| MeWo:Linsitinib:Ldose: -1 (uM) Interaction | -34.85220049 | 19.0858749 | -1.826072982 | 0.067853105 |
| SKMEL2:Linsitinib:Ldose: -1 (uM) Interaction | -54.60033333 | 19.0858749 | -2.860771834 | 0.004230176 |
| UACC0257:Linsitinib:Ldose: -1 (uM) Interaction | -61.65139387 | 19.0858749 | -3.23021052 | 0.001238847 |
| MeWo:Aphrocallistin.analogue:Ldose: -1 (uM) Interaction | -37.95401693 | 19.0858749 | -1.988591937 | 0.046758894 |
| SKMEL2:Aphrocallistin.analogue:Ldose: -1 (uM) Interaction | -44.967301 | 19.0858749 | -2.356051333 | 0.018479273 |
| UACC0257:Aphrocallistin.analogue:Ldose: -1 (uM) Interaction | -63.09388909 | 19.0858749 | -3.305789723 | 0.000948639 |
| MeWo:Mitotane..o.p..DDD..Lysodren.:Ldose: -1 (uM) Interaction | -33.45018433 | 19.0858749 | -1.75261467 | 0.079682398 |
| SKMEL2:Mitotane..o.p..DDD..Lysodren.:Ldose: -1 (uM) Interaction | -47.45188008 | 19.0858749 | -2.48623028 | 0.012917959 |
| UACC0257:Mitotane..o.p..DDD..Lysodren.:Ldose: -1 (uM) Interaction | -43.47426411 | 19.0858749 | -2.277824011 | 0.022746817 |
| MeWo:Etoposide:Ldose: -1 (uM) Interaction | -34.61924388 | 19.0858749 | -1.813867274 | 0.06971207 |
| SKMEL2:Etoposide:Ldose: -1 (uM) Interaction | -49.20423413 | 19.0858749 | -2.578044465 | 0.009942701 |
| UACC0257:Etoposide:Ldose: -1 (uM) Interaction | -63.20848205 | 19.0858749 | -3.311793795 | 0.000928523 |
| MeWo:Vandetanib:Ldose: -1 (uM) Interaction | -47.95997913 | 19.0858749 | -2.512852012 | 0.011983198 |
| SKMEL2:Vandetanib:Ldose: -1 (uM) Interaction | -62.96637819 | 19.0858749 | -3.299108819 | 0.000971496 |
| UACC0257:Vandetanib:Ldose: -1 (uM) Interaction | -61.38072225 | 19.0858749 | -3.216028743 | 0.001301697 |
| MeWo:Carboplatin:Ldose: -1 (uM) Interaction | -40.84881447 | 19.0858749 | -2.140264184 | 0.03234462 |
| SKMEL2:Carboplatin:Ldose: -1 (uM) Interaction | -55.30820094 | 19.0858749 | -2.897860394 | 0.003760965 |
| UACC0257:Carboplatin:Ldose: -1 (uM) Interaction | -62.87127973 | 19.0858749 | -3.294126157 | 0.000988874 |
| MeWo:Gefitinib:Ldose: -1 (uM) Interaction | -36.21268815 | 19.0858749 | -1.897355419 | 0.057794406 |
| SKMEL2:Gefitinib:Ldose: -1 (uM) Interaction | -56.40190631 | 19.0858749 | -2.955164834 | 0.003128403 |
| UACC0257:Gefitinib:Ldose: -1 (uM) Interaction | -45.97555981 | 19.0858749 | -2.40887882 | 0.016009949 |
| MeWo:Vincristine.Sulfate:Ldose: -1 (uM) Interaction | -8.469965137 | 19.0858749 | -0.443781864 | 0.657204759 |
| SKMEL2:Vincristine.Sulfate:Ldose: -1 (uM) Interaction | -14.99289054 | 19.0858749 | -0.785549031 | 0.432140375 |
| UACC0257:Vincristine.Sulfate:Ldose: -1 (uM) Interaction | -4.124173219 | 19.0858749 | -0.216085102 | 0.828923459 |
| MeWo:Trametinib..GSK1120212.:Ldose: -1 (uM) Interaction | -57.47951198 | 19.0858749 | -3.011625733 | 0.002601541 |
| SKMEL2:Trametinib..GSK1120212.:Ldose: -1 (uM) Interaction | -61.65913703 | 19.0858749 | -3.230616221 | 0.001237091 |
| UACC0257:Trametinib..GSK1120212.:Ldose: -1 (uM) Interaction | -47.81204022 | 19.0858749 | -2.505100787 | 0.012248973 |
| MeWo:MLN4924:Ldose: -1 (uM) Interaction | -57.36538973 | 19.0858749 | -3.005646324 | 0.002653222 |
| SKMEL2:MLN4924:Ldose: -1 (uM) Interaction | -54.84234018 | 19.0858749 | -2.873451727 | 0.0040641 |
| UACC0257:MLN4924:Ldose: -1 (uM) Interaction | -80.22852409 | 19.0858749 | -4.20355496 | 2.64E-05 |
| MeWo:Bortezomib:Ldose: -1 (uM) Interaction | -29.3208023 | 19.0858749 | -1.536256654 | 0.124490134 |
| SKMEL2:Bortezomib:Ldose: -1 (uM) Interaction | -32.81238949 | 19.0858749 | -1.719197557 | 0.085592773 |
| UACC0257:Bortezomib:Ldose: -1 (uM) Interaction | -32.70541421 | 19.0858749 | -1.713592612 | 0.08661796 |
| MeWo:Fluorouracil...5.FU.:Ldose: -1 (uM) Interaction | -36.6137235 | 19.0858749 | -1.918367573 | 0.055077624 |
| SKMEL2:Fluorouracil...5.FU.:Ldose: -1 (uM) Interaction | -50.49607839 | 19.0858749 | -2.645730346 | 0.008157397 |
| UACC0257:Fluorouracil...5.FU.:Ldose: -1 (uM) Interaction | -64.4210098 | 19.0858749 | -3.375323906 | 0.00073859 |
| MeWo:Lapatinib:Ldose: -1 (uM) Interaction | -41.55279464 | 19.0858749 | -2.177149063 | 0.029480266 |
| SKMEL2:Lapatinib:Ldose: -1 (uM) Interaction | -57.50100346 | 19.0858749 | -3.012751774 | 0.002591912 |
| UACC0257:Lapatinib:Ldose: -1 (uM) Interaction | -42.62266662 | 19.0858749 | -2.233204758 | 0.025545674 |
| MeWo:Mitoxantrone:Ldose: -1 (uM) Interaction | -30.175503 | 19.0858749 | -1.581038499 | 0.113883918 |
| SKMEL2:Mitoxantrone:Ldose: -1 (uM) Interaction | -36.4374634 | 19.0858749 | -1.909132466 | 0.056258279 |
| UACC0257:Mitoxantrone:Ldose: -1 (uM) Interaction | -52.83994758 | 19.0858749 | -2.76853683 | 0.005635674 |
| MeWo:Imatinib:Ldose: -1 (uM) Interaction | -39.92993816 | 19.0858749 | -2.092119873 | 0.036439471 |
| SKMEL2:Imatinib:Ldose: -1 (uM) Interaction | -59.40544965 | 19.0858749 | -3.112534792 | 0.001857309 |
| UACC0257:Imatinib:Ldose: -1 (uM) Interaction | -41.13664148 | 19.0858749 | -2.155344814 | 0.031145905 |
| MeWo:Imiquimod:Ldose: -1 (uM) Interaction | -44.34090228 | 19.0858749 | -2.323231317 | 0.020175992 |
| SKMEL2:Imiquimod:Ldose: -1 (uM) Interaction | -56.98680015 | 19.0858749 | -2.985810211 | 0.00283146 |
| UACC0257:Imiquimod:Ldose: -1 (uM) Interaction | -39.24648017 | 19.0858749 | -2.056310249 | 0.039764641 |
| MeWo:Dacomitinib..PF299804.:Ldose: -1 (uM) Interaction | -42.33152241 | 19.0858749 | -2.217950324 | 0.026568611 |
| SKMEL2:Dacomitinib..PF299804.:Ldose: -1 (uM) Interaction | -58.95161494 | 19.0858749 | -3.088756227 | 0.00201252 |
| UACC0257:Dacomitinib..PF299804.:Ldose: -1 (uM) Interaction | -46.43193866 | 19.0858749 | -2.432790685 | 0.014991043 |
| MeWo:PD325901:Ldose: -1 (uM) Interaction | -46.84835437 | 19.0858749 | -2.45460869 | 0.014111654 |
| SKMEL2:PD325901:Ldose: -1 (uM) Interaction | -68.87876135 | 19.0858749 | -3.608886767 | 0.000308211 |
| UACC0257:PD325901:Ldose: -1 (uM) Interaction | -54.66980178 | 19.0858749 | -2.864411617 | 0.004181885 |
| MeWo:Vismodegib:Ldose: -1 (uM) Interaction | -32.90611331 | 19.0858749 | -1.724108195 | 0.084702661 |
| SKMEL2:Vismodegib:Ldose: -1 (uM) Interaction | -45.27328543 | 19.0858749 | -2.372083317 | 0.017696837 |
| UACC0257:Vismodegib:Ldose: -1 (uM) Interaction | -48.49223044 | 19.0858749 | -2.540739196 | 0.011068796 |
| MeWo:Temozolomide:Ldose: -1 (uM) Interaction | -29.29717032 | 19.0858749 | -1.535018462 | 0.124793981 |
| SKMEL2:Temozolomide:Ldose: -1 (uM) Interaction | -54.42771128 | 19.0858749 | -2.851727342 | 0.004352373 |
| UACC0257:Temozolomide:Ldose: -1 (uM) Interaction | -56.70542072 | 19.0858749 | -2.971067401 | 0.002970942 |
| MeWo:Mercaptopurine:Ldose: -1 (uM) Interaction | -31.6775522 | 19.0858749 | -1.659738019 | 0.096981724 |
| SKMEL2:Mercaptopurine:Ldose: -1 (uM) Interaction | -49.78701421 | 19.0858749 | -2.608579092 | 0.009098178 |
| UACC0257:Mercaptopurine:Ldose: -1 (uM) Interaction | -35.43629545 | 19.0858749 | -1.856676502 | 0.063370835 |
| MeWo:Dasatinib:Ldose: -1 (uM) Interaction | -41.16014308 | 19.0858749 | -2.156576175 | 0.031049734 |
| SKMEL2:Dasatinib:Ldose: -1 (uM) Interaction | -65.0107071 | 19.0858749 | -3.406220959 | 0.000659891 |
| UACC0257:Dasatinib:Ldose: -1 (uM) Interaction | -54.09643928 | 19.0858749 | -2.834370422 | 0.004595879 |
| MeWo:Daunorubicin.HCl:Ldose: -1 (uM) Interaction | -14.52633012 | 19.0858749 | -0.761103706 | 0.446603459 |
| SKMEL2:Daunorubicin.HCl:Ldose: -1 (uM) Interaction | -34.35570462 | 19.0858749 | -1.800059196 | 0.071865278 |
| UACC0257:Daunorubicin.HCl:Ldose: -1 (uM) Interaction | -37.78392505 | 19.0858749 | -1.979680012 | 0.047752204 |
| MeWo:Sirolimus..Rapamycin.:Ldose: -1 (uM) Interaction | -40.45069218 | 19.0858749 | -2.119404659 | 0.034067691 |
| SKMEL2:Sirolimus..Rapamycin.:Ldose: -1 (uM) Interaction | -53.53279443 | 19.0858749 | -2.80483838 | 0.005038691 |
| UACC0257:Sirolimus..Rapamycin.:Ldose: -1 (uM) Interaction | -57.09337656 | 19.0858749 | -2.991394257 | 0.002780209 |
| MeWo:INK.128..MLN0128.:Ldose: -1 (uM) Interaction | -22.93226857 | 19.0858749 | -1.201530907 | 0.229558535 |
| SKMEL2:INK.128..MLN0128.:Ldose: -1 (uM) Interaction | -47.40614765 | 19.0858749 | -2.483834139 | 0.013005177 |
| UACC0257:INK.128..MLN0128.:Ldose: -1 (uM) Interaction | -49.71530649 | 19.0858749 | -2.604821983 | 0.009198516 |
| MeWo:Quizartinib:Ldose: -1 (uM) Interaction | -36.47756013 | 19.0858749 | -1.911233324 | 0.055987861 |
| SKMEL2:Quizartinib:Ldose: -1 (uM) Interaction | -59.99544123 | 19.0858749 | -3.143447265 | 0.001671971 |
| UACC0257:Quizartinib:Ldose: -1 (uM) Interaction | -76.8621476 | 19.0858749 | -4.027174443 | 5.66E-05 |
| MeWo:Sorafenib:Ldose: -1 (uM) Interaction | -33.01140956 | 19.0858749 | -1.729625167 | 0.083711592 |
| SKMEL2:Sorafenib:Ldose: -1 (uM) Interaction | -49.66894184 | 19.0858749 | -2.602392718 | 0.009263916 |
| UACC0257:Sorafenib:Ldose: -1 (uM) Interaction | -46.12495095 | 19.0858749 | -2.416706135 | 0.015669911 |
| MeWo:Carmustine:Ldose: -1 (uM) Interaction | -34.03939763 | 19.0858749 | -1.783486364 | 0.074521241 |
| SKMEL2:Carmustine:Ldose: -1 (uM) Interaction | -48.9579822 | 19.0858749 | -2.565142152 | 0.010320063 |
| UACC0257:Carmustine:Ldose: -1 (uM) Interaction | -55.94083208 | 19.0858749 | -2.931006956 | 0.003382202 |
| MeWo:Uracil.mustard:Ldose: -1 (uM) Interaction | -28.20749449 | 19.0858749 | -1.477925149 | 0.139442435 |
| SKMEL2:Uracil.mustard:Ldose: -1 (uM) Interaction | -48.82070861 | 19.0858749 | -2.557949734 | 0.010535912 |
| UACC0257:Uracil.mustard:Ldose: -1 (uM) Interaction | -49.63936913 | 19.0858749 | -2.600843262 | 0.009305847 |
| MeWo:Ixabepilone:Ldose: -1 (uM) Interaction | -18.48387493 | 19.0858749 | -0.968458351 | 0.332826384 |
| SKMEL2:Ixabepilone:Ldose: -1 (uM) Interaction | -50.63308836 | 19.0858749 | -2.652908951 | 0.007985991 |
| UACC0257:Ixabepilone:Ldose: -1 (uM) Interaction | -44.31634883 | 19.0858749 | -2.321944845 | 0.020245182 |
| MeWo:Valrubicin:Ldose: -1 (uM) Interaction | -30.96503946 | 19.0858749 | -1.622406079 | 0.104731063 |
| SKMEL2:Valrubicin:Ldose: -1 (uM) Interaction | -54.90254374 | 19.0858749 | -2.876606079 | 0.004023716 |
| UACC0257:Valrubicin:Ldose: -1 (uM) Interaction | -47.94010652 | 19.0858749 | -2.511810791 | 0.0120186 |
| MeWo:Triethylenemelamine:Ldose: -1 (uM) Interaction | -33.62476021 | 19.0858749 | -1.761761533 | 0.07812378 |
| SKMEL2:Triethylenemelamine:Ldose: -1 (uM) Interaction | -51.5949039 | 19.0858749 | -2.703303054 | 0.006870797 |
| UACC0257:Triethylenemelamine:Ldose: -1 (uM) Interaction | -47.22057315 | 19.0858749 | -2.474111006 | 0.013364465 |
| MeWo:Palbociclib..PD.0332991..Isethionate:Ldose: -1 (uM) Interaction | -36.07902958 | 19.0858749 | -1.890352408 | 0.058724255 |
| SKMEL2:Palbociclib..PD.0332991..Isethionate:Ldose: -1 (uM) Interaction | -51.8987135 | 19.0858749 | -2.719221088 | 0.006548828 |
| UACC0257:Palbociclib..PD.0332991..Isethionate:Ldose: -1 (uM) Interaction | -55.10032802 | 19.0858749 | -2.88696894 | 0.003893594 |
| MeWo:Afatinib:Ldose: -1 (uM) Interaction | -31.68706265 | 19.0858749 | -1.660236317 | 0.096881477 |
| SKMEL2:Afatinib:Ldose: -1 (uM) Interaction | -48.62382251 | 19.0858749 | -2.547633932 | 0.010852507 |
| UACC0257:Afatinib:Ldose: -1 (uM) Interaction | -62.32238272 | 19.0858749 | -3.265366826 | 0.001094931 |
| MeWo:Doxorubicin.HCl:Ldose: -1 (uM) Interaction | -26.5315974 | 19.0858749 | -1.390116908 | 0.164507744 |
| SKMEL2:Doxorubicin.HCl:Ldose: -1 (uM) Interaction | -41.9069922 | 19.0858749 | -2.195707162 | 0.028123536 |
| UACC0257:Doxorubicin.HCl:Ldose: -1 (uM) Interaction | -36.83508103 | 19.0858749 | -1.92996555 | 0.053624221 |
| MeWo:Exemestane:Ldose: -1 (uM) Interaction | -36.66469014 | 19.0858749 | -1.921037958 | 0.054740106 |
| SKMEL2:Exemestane:Ldose: -1 (uM) Interaction | -45.28821424 | 19.0858749 | -2.372865509 | 0.017659416 |
| UACC0257:Exemestane:Ldose: -1 (uM) Interaction | -65.57945919 | 19.0858749 | -3.436020594 | 0.00059144 |
| MeWo:Tretinoin:Ldose: -1 (uM) Interaction | -33.2253489 | 19.0858749 | -1.74083447 | 0.081726877 |
| SKMEL2:Tretinoin:Ldose: -1 (uM) Interaction | -50.62893757 | 19.0858749 | -2.652691472 | 0.007991136 |
| UACC0257:Tretinoin:Ldose: -1 (uM) Interaction | -56.04860729 | 19.0858749 | -2.936653813 | 0.003321252 |
| MeWo:Fulvestrant:Ldose: -1 (uM) Interaction | -41.20836324 | 19.0858749 | -2.159102659 | 0.030853209 |
| SKMEL2:Fulvestrant:Ldose: -1 (uM) Interaction | -58.87951708 | 19.0858749 | -3.084978677 | 0.002038246 |
| UACC0257:Fulvestrant:Ldose: -1 (uM) Interaction | -55.5533205 | 19.0858749 | -2.910703376 | 0.003609856 |
| MeWo:Docetaxel:Ldose: -1 (uM) Interaction | -29.29237016 | 19.0858749 | -1.534766958 | 0.124855769 |
| SKMEL2:Docetaxel:Ldose: -1 (uM) Interaction | -54.43441414 | 19.0858749 | -2.852078537 | 0.004347569 |
| UACC0257:Docetaxel:Ldose: -1 (uM) Interaction | -45.59831588 | 19.0858749 | -2.389113212 | 0.016897649 |
| MeWo:Everolimus:Ldose: -1 (uM) Interaction | -42.25882804 | 19.0858749 | -2.21414152 | 0.026829477 |
| SKMEL2:Everolimus:Ldose: -1 (uM) Interaction | -53.52806481 | 19.0858749 | -2.804590573 | 0.005042564 |
| UACC0257:Everolimus:Ldose: -1 (uM) Interaction | -67.50803477 | 19.0858749 | -3.537067865 | 0.000405446 |
| MeWo:MLN.2480:Ldose: -1 (uM) Interaction | -32.39587185 | 19.0858749 | -1.697374211 | 0.089640381 |
| SKMEL2:MLN.2480:Ldose: -1 (uM) Interaction | -47.15404736 | 19.0858749 | -2.470625403 | 0.013495385 |
| UACC0257:MLN.2480:Ldose: -1 (uM) Interaction | -75.90760945 | 19.0858749 | -3.977161637 | 7.00E-05 |
| MeWo:LY2157299:Ldose: -1 (uM) Interaction | -40.28254091 | 19.0858749 | -2.110594412 | 0.034818683 |
| SKMEL2:LY2157299:Ldose: -1 (uM) Interaction | -53.30888532 | 19.0858749 | -2.793106714 | 0.005225052 |
| UACC0257:LY2157299:Ldose: -1 (uM) Interaction | -60.84011683 | 19.0858749 | -3.187703847 | 0.001436122 |
| MeWo:Allopurinol:Ldose: -1 (uM) Interaction | -51.74720324 | 19.0858749 | -2.711282743 | 0.006707658 |
| SKMEL2:Allopurinol:Ldose: -1 (uM) Interaction | -50.38821418 | 19.0858749 | -2.640078826 | 0.008294649 |
| UACC0257:Allopurinol:Ldose: -1 (uM) Interaction | -53.63276384 | 19.0858749 | -2.810076254 | 0.004957443 |
| MeWo:Pipobroman:Ldose: -1 (uM) Interaction | -36.7427299 | 19.0858749 | -1.925126834 | 0.054226645 |
| SKMEL2:Pipobroman:Ldose: -1 (uM) Interaction | -48.41603981 | 19.0858749 | -2.536747205 | 0.011195768 |
| UACC0257:Pipobroman:Ldose: -1 (uM) Interaction | -57.54067806 | 19.0858749 | -3.014830516 | 0.002574223 |
| MeWo:Letrozole:Ldose: -1 (uM) Interaction | -31.26686837 | 19.0858749 | -1.638220335 | 0.101390345 |
| SKMEL2:Letrozole:Ldose: -1 (uM) Interaction | -42.73497653 | 19.0858749 | -2.239089209 | 0.025160279 |
| UACC0257:Letrozole:Ldose: -1 (uM) Interaction | -63.12475366 | 19.0858749 | -3.307406865 | 0.000943182 |
| MeWo:Thiotepa:Ldose: -1 (uM) Interaction | -45.73287548 | 19.0858749 | -2.396163431 | 0.016576177 |
| SKMEL2:Thiotepa:Ldose: -1 (uM) Interaction | -58.09745156 | 19.0858749 | -3.044002535 | 0.002337347 |
| UACC0257:Thiotepa:Ldose: -1 (uM) Interaction | -51.75788859 | 19.0858749 | -2.7118426 | 0.006696344 |
| MeWo:Plicamycin:Ldose: -1 (uM) Interaction | -8.508303307 | 19.0858749 | -0.445790584 | 0.655753005 |
| SKMEL2:Plicamycin:Ldose: -1 (uM) Interaction | -36.08092421 | 19.0858749 | -1.890451677 | 0.058710988 |
| UACC0257:Plicamycin:Ldose: -1 (uM) Interaction | -18.25963598 | 19.0858749 | -0.956709403 | 0.338724688 |
| MeWo:Erlotinib.HCl:Ldose: -1 (uM) Interaction | -47.84765697 | 19.0858749 | -2.506966918 | 0.012184514 |
| SKMEL2:Erlotinib.HCl:Ldose: -1 (uM) Interaction | -54.61264271 | 19.0858749 | -2.861416781 | 0.004221583 |
| UACC0257:Erlotinib.HCl:Ldose: -1 (uM) Interaction | -70.05634464 | 19.0858749 | -3.670585971 | 0.000242579 |
| MeWo:MEK.162..ARRY.438162.:Ldose: -1 (uM) Interaction | -11.24572522 | 19.0858749 | -0.589217171 | 0.555721779 |
| SKMEL2:MEK.162..ARRY.438162.:Ldose: -1 (uM) Interaction | -15.14245037 | 19.0858749 | -0.793385184 | 0.427562179 |
| UACC0257:MEK.162..ARRY.438162.:Ldose: -1 (uM) Interaction | -36.31438271 | 19.0858749 | -1.902683682 | 0.057095154 |
| MeWo:Baricitinib..LY3009104..INCB028050.:Ldose: -1 (uM) Interaction | -33.72161446 | 19.0858749 | -1.766836189 | 0.077269825 |
| SKMEL2:Baricitinib..LY3009104..INCB028050.:Ldose: -1 (uM) Interaction | -46.03237053 | 19.0858749 | -2.411855405 | 0.015879882 |
| UACC0257:Baricitinib..LY3009104..INCB028050.:Ldose: -1 (uM) Interaction | -71.75153568 | 19.0858749 | -3.759405113 | 0.000170768 |
| MeWo:Arsenic.Trioxide:Ldose: -1 (uM) Interaction | -35.43869831 | 19.0858749 | -1.8568024 | 0.063352914 |
| SKMEL2:Arsenic.Trioxide:Ldose: -1 (uM) Interaction | -33.05168889 | 19.0858749 | -1.731735594 | 0.083334967 |
| UACC0257:Arsenic.Trioxide:Ldose: -1 (uM) Interaction | -44.34475762 | 19.0858749 | -2.323433317 | 0.020165146 |
| MeWo:Celecoxib:Ldose: -1 (uM) Interaction | -28.30352461 | 19.0858749 | -1.482956624 | 0.138100566 |
| SKMEL2:Celecoxib:Ldose: -1 (uM) Interaction | -40.86012822 | 19.0858749 | -2.140856966 | 0.032296766 |
| UACC0257:Celecoxib:Ldose: -1 (uM) Interaction | -76.52741178 | 19.0858749 | -4.009636037 | 6.10E-05 |
| MeWo:Bendamustine.HCl:Ldose: -1 (uM) Interaction | -35.69820665 | 19.0858749 | -1.870399279 | 0.061441943 |
| SKMEL2:Bendamustine.HCl:Ldose: -1 (uM) Interaction | -45.57715883 | 19.0858749 | -2.388004693 | 0.016948689 |
| UACC0257:Bendamustine.HCl:Ldose: -1 (uM) Interaction | -66.13677475 | 19.0858749 | -3.465221014 | 0.000530827 |
| MeWo:Chlorambucil:Ldose: -1 (uM) Interaction | -36.27965518 | 19.0858749 | -1.90086414 | 0.057333145 |
| SKMEL2:Chlorambucil:Ldose: -1 (uM) Interaction | -47.69980629 | 19.0858749 | -2.499220316 | 0.012454074 |
| UACC0257:Chlorambucil:Ldose: -1 (uM) Interaction | -74.17480335 | 19.0858749 | -3.886371662 | 0.000102058 |
| MeWo:Zoledronic.Acid:Ldose: -1 (uM) Interaction | -34.71019809 | 19.0858749 | -1.8186328 | 0.068981351 |
| SKMEL2:Zoledronic.Acid:Ldose: -1 (uM) Interaction | -52.99428542 | 19.0858749 | -2.776623325 | 0.005497423 |
| UACC0257:Zoledronic.Acid:Ldose: -1 (uM) Interaction | -60.11946068 | 19.0858749 | -3.149945235 | 0.001635243 |
| MeWo:Actinomycin.D:Ldose: -1 (uM) Interaction | -46.32855764 | 19.0858749 | -2.42737406 | 0.015216708 |
| SKMEL2:Actinomycin.D:Ldose: -1 (uM) Interaction | -89.90565749 | 19.0858749 | -4.710586126 | 2.49E-06 |
| UACC0257:Actinomycin.D:Ldose: -1 (uM) Interaction | -80.98324842 | 19.0858749 | -4.243098567 | 2.21E-05 |
| MeWo:Temsirolimus..CCI.779..Torisel.:Ldose: -1 (uM) Interaction | -39.66238058 | 19.0858749 | -2.078101255 | 0.037711847 |
| SKMEL2:Temsirolimus..CCI.779..Torisel.:Ldose: -1 (uM) Interaction | -50.56397637 | 19.0858749 | -2.649287845 | 0.008072046 |
| UACC0257:Temsirolimus..CCI.779..Torisel.:Ldose: -1 (uM) Interaction | -60.48533028 | 19.0858749 | -3.169114888 | 0.001531177 |
| MeWo:Foretinib..GSK1363089.:Ldose: -1 (uM) Interaction | -52.78444906 | 19.0858749 | -2.765628998 | 0.00568615 |
| SKMEL2:Foretinib..GSK1363089.:Ldose: -1 (uM) Interaction | -45.19742353 | 19.0858749 | -2.36810855 | 0.017888067 |
| UACC0257:Foretinib..GSK1363089.:Ldose: -1 (uM) Interaction | -59.83459566 | 19.0858749 | -3.135019798 | 0.001720734 |
| MeWo:Decitabine:Ldose: -1 (uM) Interaction | -44.53764672 | 19.0858749 | -2.333539697 | 0.019628989 |
| SKMEL2:Decitabine:Ldose: -1 (uM) Interaction | -49.10035086 | 19.0858749 | -2.572601525 | 0.010100369 |
| UACC0257:Decitabine:Ldose: -1 (uM) Interaction | -51.78313768 | 19.0858749 | -2.71316552 | 0.006669677 |
| MeWo:Methotrexate:Ldose: -1 (uM) Interaction | 9.057663156 | 19.0858749 | 0.474574166 | 0.635095335 |
| SKMEL2:Methotrexate:Ldose: -1 (uM) Interaction | -5.288368354 | 19.0858749 | -0.277082837 | 0.781719159 |
| UACC0257:Methotrexate:Ldose: -1 (uM) Interaction | -21.08536305 | 19.0858749 | -1.104762719 | 0.269274759 |
| MeWo:Axitinib:Ldose: -1 (uM) Interaction | -28.74889992 | 19.0858749 | -1.506291961 | 0.132006905 |
| SKMEL2:Axitinib:Ldose: -1 (uM) Interaction | -47.98753685 | 19.0858749 | -2.514295892 | 0.011934259 |
| UACC0257:Axitinib:Ldose: -1 (uM) Interaction | -68.62250305 | 19.0858749 | -3.595460173 | 0.000324542 |
| MeWo:Oxaliplatin:Ldose: -1 (uM) Interaction | -38.43199011 | 19.0858749 | -2.013635231 | 0.044060257 |
| SKMEL2:Oxaliplatin:Ldose: -1 (uM) Interaction | -45.37752846 | 19.0858749 | -2.377545106 | 0.017436987 |
| UACC0257:Oxaliplatin:Ldose: -1 (uM) Interaction | -65.37059068 | 19.0858749 | -3.425076976 | 0.000615771 |
| MeWo:Cabazitaxel:Ldose: -1 (uM) Interaction | -35.79063916 | 19.0858749 | -1.875242259 | 0.06077292 |
| SKMEL2:Cabazitaxel:Ldose: -1 (uM) Interaction | -49.04559653 | 19.0858749 | -2.569732684 | 0.010184364 |
| UACC0257:Cabazitaxel:Ldose: -1 (uM) Interaction | -39.13080057 | 19.0858749 | -2.050249244 | 0.040352192 |
| MeWo:Amifostine:Ldose: -1 (uM) Interaction | -42.64020606 | 19.0858749 | -2.234123732 | 0.025485153 |
| SKMEL2:Amifostine:Ldose: -1 (uM) Interaction | -53.12623207 | 19.0858749 | -2.783536639 | 0.005381664 |
| UACC0257:Amifostine:Ldose: -1 (uM) Interaction | -69.5869812 | 19.0858749 | -3.645993782 | 0.000266987 |
| MeWo:Flutamide..Eulexin.:Ldose: -1 (uM) Interaction | -37.91275966 | 19.0858749 | -1.986430272 | 0.046998219 |
| SKMEL2:Flutamide..Eulexin.:Ldose: -1 (uM) Interaction | -49.9910452 | 19.0858749 | -2.619269249 | 0.008818011 |
| UACC0257:Flutamide..Eulexin.:Ldose: -1 (uM) Interaction | -75.65406523 | 19.0858749 | -3.963877246 | 7.40E-05 |
| MeWo:LDK378:Ldose: -1 (uM) Interaction | -31.02263801 | 19.0858749 | -1.625423942 | 0.104086888 |
| SKMEL2:LDK378:Ldose: -1 (uM) Interaction | -40.58362006 | 19.0858749 | -2.126369385 | 0.033483853 |
| UACC0257:LDK378:Ldose: -1 (uM) Interaction | -55.91542298 | 19.0858749 | -2.929675652 | 0.003396719 |
| MeWo:Pralatrexate:Ldose: -1 (uM) Interaction | -30.2786009 | 19.0858749 | -1.58644029 | 0.112654146 |
| SKMEL2:Pralatrexate:Ldose: -1 (uM) Interaction | -46.17173547 | 19.0858749 | -2.419157399 | 0.015564736 |
| UACC0257:Pralatrexate:Ldose: -1 (uM) Interaction | -55.69286104 | 19.0858749 | -2.918014571 | 0.00352632 |
| MeWo:Topotecan.HCl:Ldose: -1 (uM) Interaction | -0.490637985 | 19.0858749 | -0.025706864 | 0.979491387 |
| SKMEL2:Topotecan.HCl:Ldose: -1 (uM) Interaction | -33.17612378 | 19.0858749 | -1.738255331 | 0.082180123 |
| UACC0257:Topotecan.HCl:Ldose: -1 (uM) Interaction | -38.93835345 | 19.0858749 | -2.040166021 | 0.041345964 |
| MeWo:Pemetrexed:Ldose: -1 (uM) Interaction | -33.92619466 | 19.0858749 | -1.777555121 | 0.075491056 |
| SKMEL2:Pemetrexed:Ldose: -1 (uM) Interaction | -42.90768344 | 19.0858749 | -2.248138148 | 0.024577453 |
| UACC0257:Pemetrexed:Ldose: -1 (uM) Interaction | -42.74854866 | 19.0858749 | -2.239800318 | 0.025114049 |
| MeWo:Bleomycin.Sulfate:Ldose: -1 (uM) Interaction | -30.94918516 | 19.0858749 | -1.621575397 | 0.10490893 |
| SKMEL2:Bleomycin.Sulfate:Ldose: -1 (uM) Interaction | -48.22790105 | 19.0858749 | -2.526889719 | 0.011514861 |
| UACC0257:Bleomycin.Sulfate:Ldose: -1 (uM) Interaction | -37.44872691 | 19.0858749 | -1.962117383 | 0.049761675 |
| MeWo:Axitinib.1:Ldose: -1 (uM) Interaction | -29.36592513 | 19.0858749 | -1.538620854 | 0.123911573 |
| SKMEL2:Axitinib.1:Ldose: -1 (uM) Interaction | -40.51886871 | 19.0858749 | -2.122976753 | 0.033767172 |
| UACC0257:Axitinib.1:Ldose: -1 (uM) Interaction | -55.89721895 | 19.0858749 | -2.928721856 | 0.003407154 |
| MeWo:Ibrutinib..PCI.32765.:Ldose: -1 (uM) Interaction | -35.67084199 | 19.0858749 | -1.868965514 | 0.061641173 |
| SKMEL2:Ibrutinib..PCI.32765.:Ldose: -1 (uM) Interaction | -48.13298963 | 19.0858749 | -2.521916857 | 0.011678878 |
| UACC0257:Ibrutinib..PCI.32765.:Ldose: -1 (uM) Interaction | -50.12224855 | 19.0858749 | -2.626143618 | 0.008641945 |
| MeWo:Tamoxifen.Citrate:Ldose: -1 (uM) Interaction | -36.34885539 | 19.0858749 | -1.90448987 | 0.056859723 |
| SKMEL2:Tamoxifen.Citrate:Ldose: -1 (uM) Interaction | -44.12502683 | 19.0858749 | -2.311920573 | 0.020791445 |
| UACC0257:Tamoxifen.Citrate:Ldose: -1 (uM) Interaction | -53.98943796 | 19.0858749 | -2.828764113 | 0.004677129 |
| MeWo:Vemurafenib:Ldose: -1 (uM) Interaction | 5.789764156 | 19.0858749 | 0.303353354 | 0.76162352 |
| SKMEL2:Vemurafenib:Ldose: -1 (uM) Interaction | -6.631152462 | 19.0858749 | -0.347437699 | 0.72826591 |
| UACC0257:Vemurafenib:Ldose: -1 (uM) Interaction | -28.03191957 | 19.0858749 | -1.468725941 | 0.141921742 |
| MeWo:Pazopanib.HCl:Ldose: -1 (uM) Interaction | -37.05897286 | 19.0858749 | -1.94169631 | 0.052186898 |
| SKMEL2:Pazopanib.HCl:Ldose: -1 (uM) Interaction | -50.47982169 | 19.0858749 | -2.64487858 | 0.008177952 |
| UACC0257:Pazopanib.HCl:Ldose: -1 (uM) Interaction | -45.82335955 | 19.0858749 | -2.400904323 | 0.016363037 |
| MeWo:Abiraterone:Ldose: -1 (uM) Interaction | -35.98478614 | 19.0858749 | -1.885414546 | 0.059387338 |
| SKMEL2:Abiraterone:Ldose: -1 (uM) Interaction | -49.92676111 | 19.0858749 | -2.615901099 | 0.00890544 |
| UACC0257:Abiraterone:Ldose: -1 (uM) Interaction | -58.89586906 | 19.0858749 | -3.085835435 | 0.002032385 |
| MeWo:Bosutinib..SKI.606.:Ldose: -1 (uM) Interaction | -27.55099104 | 19.0858749 | -1.443527802 | 0.148886389 |
| SKMEL2:Bosutinib..SKI.606.:Ldose: -1 (uM) Interaction | -38.58260711 | 19.0858749 | -2.021526774 | 0.043237605 |
| UACC0257:Bosutinib..SKI.606.:Ldose: -1 (uM) Interaction | -46.56980698 | 19.0858749 | -2.440014263 | 0.014694689 |
| MeWo:Sabutoclax..BI.97C1.:Ldose: -1 (uM) Interaction | -33.76569473 | 19.0858749 | -1.769145764 | 0.0768837 |
| SKMEL2:Sabutoclax..BI.97C1.:Ldose: -1 (uM) Interaction | -50.0580464 | 19.0858749 | -2.622779761 | 0.008727703 |
| UACC0257:Sabutoclax..BI.97C1.:Ldose: -1 (uM) Interaction | -42.0243398 | 19.0858749 | -2.201855562 | 0.027686074 |
| MeWo:Thioguanine:Ldose: -0.698970004 (uM) Interaction | -58.71205177 | 19.0858749 | -3.076204371 | 0.002099169 |
| SKMEL2:Thioguanine:Ldose: -0.698970004 (uM) Interaction | -65.32046296 | 19.0858749 | -3.422450546 | 0.000621747 |
| UACC0257:Thioguanine:Ldose: -0.698970004 (uM) Interaction | -59.50973332 | 19.0858749 | -3.117998711 | 0.001823234 |
| MeWo:Irinotecan.HCl:Ldose: -0.698970004 (uM) Interaction | -46.46132891 | 19.0858749 | -2.43433058 | 0.01492743 |
| SKMEL2:Irinotecan.HCl:Ldose: -0.698970004 (uM) Interaction | -54.03346353 | 19.0858749 | -2.831070822 | 0.004643542 |
| UACC0257:Irinotecan.HCl:Ldose: -0.698970004 (uM) Interaction | -53.13126987 | 19.0858749 | -2.783800594 | 0.005377289 |
| MeWo:Romidepsin:Ldose: -0.698970004 (uM) Interaction | -52.85108309 | 19.0858749 | -2.769120272 | 0.005625595 |
| SKMEL2:Romidepsin:Ldose: -0.698970004 (uM) Interaction | -59.58576088 | 19.0858749 | -3.121982157 | 0.001798755 |
| UACC0257:Romidepsin:Ldose: -0.698970004 (uM) Interaction | -65.72236298 | 19.0858749 | -3.443508005 | 0.000575311 |
| MeWo:Paclitaxel:Ldose: -0.698970004 (uM) Interaction | -51.0098105 | 19.0858749 | -2.672647221 | 0.007531201 |
| SKMEL2:Paclitaxel:Ldose: -0.698970004 (uM) Interaction | -66.55720956 | 19.0858749 | -3.487249598 | 0.000488988 |
| UACC0257:Paclitaxel:Ldose: -0.698970004 (uM) Interaction | -52.89505121 | 19.0858749 | -2.771423972 | 0.005585958 |
| MeWo:Alisertib..MLN8237.:Ldose: -0.698970004 (uM) Interaction | -49.25731312 | 19.0858749 | -2.580825526 | 0.00986299 |
| SKMEL2:Alisertib..MLN8237.:Ldose: -0.698970004 (uM) Interaction | -64.67315458 | 19.0858749 | -3.388534973 | 0.000703929 |
| UACC0257:Alisertib..MLN8237.:Ldose: -0.698970004 (uM) Interaction | -60.71826165 | 19.0858749 | -3.181319273 | 0.001468139 |
| MeWo:Vorinostat:Ldose: -0.698970004 (uM) Interaction | -53.49935286 | 19.0858749 | -2.803086217 | 0.005066137 |
| SKMEL2:Vorinostat:Ldose: -0.698970004 (uM) Interaction | -64.50207261 | 19.0858749 | -3.379571173 | 0.000727278 |
| UACC0257:Vorinostat:Ldose: -0.698970004 (uM) Interaction | -52.88428915 | 19.0858749 | -2.770860096 | 0.005595637 |
| MeWo:Busulfan:Ldose: -0.698970004 (uM) Interaction | -57.2826039 | 19.0858749 | -3.00130878 | 0.002691297 |
| SKMEL2:Busulfan:Ldose: -0.698970004 (uM) Interaction | -62.08359061 | 19.0858749 | -3.252855368 | 0.001144275 |
| UACC0257:Busulfan:Ldose: -0.698970004 (uM) Interaction | -58.55808212 | 19.0858749 | -3.068137166 | 0.002156651 |
| MeWo:Mechlorethamine.HCl:Ldose: -0.698970004 (uM) Interaction | -60.8360148 | 19.0858749 | -3.187488922 | 0.001437189 |
| SKMEL2:Mechlorethamine.HCl:Ldose: -0.698970004 (uM) Interaction | -67.18094394 | 19.0858749 | -3.519930017 | 0.000432552 |
| UACC0257:Mechlorethamine.HCl:Ldose: -0.698970004 (uM) Interaction | -54.83388922 | 19.0858749 | -2.873008941 | 0.004069798 |
| MeWo:Teniposide:Ldose: -0.698970004 (uM) Interaction | -52.9182159 | 19.0858749 | -2.77263768 | 0.005565177 |
| SKMEL2:Teniposide:Ldose: -0.698970004 (uM) Interaction | -49.35675206 | 19.0858749 | -2.586035606 | 0.00971519 |
| UACC0257:Teniposide:Ldose: -0.698970004 (uM) Interaction | -52.24274581 | 19.0858749 | -2.737246581 | 0.006200666 |
| MeWo:Vinorelbine.Tartrate:Ldose: -0.698970004 (uM) Interaction | -91.57623471 | 19.0858749 | -4.798115633 | 1.61E-06 |
| SKMEL2:Vinorelbine.Tartrate:Ldose: -0.698970004 (uM) Interaction | -89.39880204 | 19.0858749 | -4.684029552 | 2.83E-06 |
| UACC0257:Vinorelbine.Tartrate:Ldose: -0.698970004 (uM) Interaction | -98.64742125 | 19.0858749 | -5.168608816 | 2.38E-07 |
| MeWo:Cabozantinib..XL.184.:Ldose: -0.698970004 (uM) Interaction | -58.65696426 | 19.0858749 | -3.073318073 | 0.002119571 |
| SKMEL2:Cabozantinib..XL.184.:Ldose: -0.698970004 (uM) Interaction | -51.85150931 | 19.0858749 | -2.716747835 | 0.006597946 |
| UACC0257:Cabozantinib..XL.184.:Ldose: -0.698970004 (uM) Interaction | -70.31502643 | 19.0858749 | -3.684139544 | 0.000230038 |
| MeWo:Dacarbazine:Ldose: -0.698970004 (uM) Interaction | -54.11551421 | 19.0858749 | -2.835369848 | 0.00458153 |
| SKMEL2:Dacarbazine:Ldose: -0.698970004 (uM) Interaction | -76.89473057 | 19.0858749 | -4.02888162 | 5.62E-05 |
| UACC0257:Dacarbazine:Ldose: -0.698970004 (uM) Interaction | -56.47411464 | 19.0858749 | -2.958948172 | 0.003090267 |
| MeWo:Clofarabine:Ldose: -0.698970004 (uM) Interaction | -2.133953437 | 19.0858749 | -0.111807997 | 0.910976689 |
| SKMEL2:Clofarabine:Ldose: -0.698970004 (uM) Interaction | -17.91849108 | 19.0858749 | -0.938835195 | 0.347825893 |
| UACC0257:Clofarabine:Ldose: -0.698970004 (uM) Interaction | -25.41703527 | 19.0858749 | -1.331719683 | 0.18296641 |
| MeWo:Cisplatin:Ldose: -0.698970004 (uM) Interaction | -60.15971462 | 19.0858749 | -3.152054331 | 0.001623483 |
| SKMEL2:Cisplatin:Ldose: -0.698970004 (uM) Interaction | -65.87447747 | 19.0858749 | -3.451478008 | 0.000558595 |
| UACC0257:Cisplatin:Ldose: -0.698970004 (uM) Interaction | -62.39937606 | 19.0858749 | -3.269400874 | 0.001079445 |
| MeWo:Floxuridine:Ldose: -0.698970004 (uM) Interaction | -29.33814996 | 19.0858749 | -1.53716558 | 0.124267455 |
| SKMEL2:Floxuridine:Ldose: -0.698970004 (uM) Interaction | -24.74575379 | 19.0858749 | -1.296548045 | 0.194800628 |
| UACC0257:Floxuridine:Ldose: -0.698970004 (uM) Interaction | -46.93677173 | 19.0858749 | -2.459241297 | 0.013930903 |
| MeWo:Lomustine..CCNU.:Ldose: -0.698970004 (uM) Interaction | -62.02031387 | 19.0858749 | -3.249539998 | 0.001157691 |
| SKMEL2:Lomustine..CCNU.:Ldose: -0.698970004 (uM) Interaction | -67.61917979 | 19.0858749 | -3.542891283 | 0.000396603 |
| UACC0257:Lomustine..CCNU.:Ldose: -0.698970004 (uM) Interaction | -68.3375816 | 19.0858749 | -3.580531779 | 0.00034365 |
| MeWo:Melphalan:Ldose: -0.698970004 (uM) Interaction | -58.95589459 | 19.0858749 | -3.088980458 | 0.002011002 |
| SKMEL2:Melphalan:Ldose: -0.698970004 (uM) Interaction | -66.50696801 | 19.0858749 | -3.484617204 | 0.000493821 |
| UACC0257:Melphalan:Ldose: -0.698970004 (uM) Interaction | -51.40036719 | 19.0858749 | -2.693110348 | 0.007084362 |
| MeWo:BGJ398..NVPBGJ398.:Ldose: -0.698970004 (uM) Interaction | -57.85821027 | 19.0858749 | -3.031467543 | 0.002436572 |
| SKMEL2:BGJ398..NVPBGJ398.:Ldose: -0.698970004 (uM) Interaction | -56.41736044 | 19.0858749 | -2.955974549 | 0.003120205 |
| UACC0257:BGJ398..NVPBGJ398.:Ldose: -0.698970004 (uM) Interaction | -61.96175544 | 19.0858749 | -3.246471843 | 0.001170236 |
| MeWo:Navitoclax..ABT.263..5uM:Ldose: -0.698970004 (uM) Interaction | -60.19269536 | 19.0858749 | -3.153782349 | 0.001613906 |
| SKMEL2:Navitoclax..ABT.263..5uM:Ldose: -0.698970004 (uM) Interaction | -61.50408935 | 19.0858749 | -3.222492534 | 0.001272694 |
| UACC0257:Navitoclax..ABT.263..5uM:Ldose: -0.698970004 (uM) Interaction | -49.17455796 | 19.0858749 | -2.576489589 | 0.009987516 |
| MeWo:Azacitidine:Ldose: -0.698970004 (uM) Interaction | -66.68805549 | 19.0858749 | -3.49410524 | 0.000476608 |
| SKMEL2:Azacitidine:Ldose: -0.698970004 (uM) Interaction | -86.87265636 | 19.0858749 | -4.551672734 | 5.35E-06 |
| UACC0257:Azacitidine:Ldose: -0.698970004 (uM) Interaction | -74.19340936 | 19.0858749 | -3.88734652 | 0.00010165 |
| MeWo:Capecitabine:Ldose: -0.698970004 (uM) Interaction | -64.58157228 | 19.0858749 | -3.38373654 | 0.00071634 |
| SKMEL2:Capecitabine:Ldose: -0.698970004 (uM) Interaction | -70.55696379 | 19.0858749 | -3.696815796 | 0.000218862 |
| UACC0257:Capecitabine:Ldose: -0.698970004 (uM) Interaction | -77.83244349 | 19.0858749 | -4.078012871 | 4.56E-05 |
| MeWo:Megestrol.acetate:Ldose: -0.698970004 (uM) Interaction | -51.49306303 | 19.0858749 | -2.697967125 | 0.006981867 |
| SKMEL2:Megestrol.acetate:Ldose: -0.698970004 (uM) Interaction | -59.54466424 | 19.0858749 | -3.119828908 | 0.001811949 |
| UACC0257:Megestrol.acetate:Ldose: -0.698970004 (uM) Interaction | -61.6077453 | 19.0858749 | -3.227923563 | 0.001248789 |
| MeWo:Cytarabine.HCl...Ara.C:Ldose: -0.698970004 (uM) Interaction | -17.04492795 | 19.0858749 | -0.893065057 | 0.371832312 |
| SKMEL2:Cytarabine.HCl...Ara.C:Ldose: -0.698970004 (uM) Interaction | -2.244309116 | 19.0858749 | -0.117590057 | 0.90639358 |
| UACC0257:Cytarabine.HCl...Ara.C:Ldose: -0.698970004 (uM) Interaction | -33.73372804 | 19.0858749 | -1.767470877 | 0.077163558 |
| MeWo:Gemcitabine.HCl:Ldose: -0.698970004 (uM) Interaction | -28.42057258 | 19.0858749 | -1.489089326 | 0.136478489 |
| SKMEL2:Gemcitabine.HCl:Ldose: -0.698970004 (uM) Interaction | 2.903680668 | 19.0858749 | 0.152137677 | 0.879079773 |
| UACC0257:Gemcitabine.HCl:Ldose: -0.698970004 (uM) Interaction | -83.89137847 | 19.0858749 | -4.395469368 | 1.11E-05 |
| MeWo:Vinblastine.Sulfate:Ldose: -0.698970004 (uM) Interaction | -52.85480643 | 19.0858749 | -2.769315356 | 0.005622229 |
| SKMEL2:Vinblastine.Sulfate:Ldose: -0.698970004 (uM) Interaction | -58.07385464 | 19.0858749 | -3.042766179 | 0.002346967 |
| UACC0257:Vinblastine.Sulfate:Ldose: -0.698970004 (uM) Interaction | -46.25664586 | 19.0858749 | -2.423606259 | 0.015375439 |
| MeWo:MLN9708..MLN2238.:Ldose: -0.698970004 (uM) Interaction | -57.98370224 | 19.0858749 | -3.038042665 | 0.002384054 |
| SKMEL2:MLN9708..MLN2238.:Ldose: -0.698970004 (uM) Interaction | -90.26645044 | 19.0858749 | -4.729489789 | 2.27E-06 |
| UACC0257:MLN9708..MLN2238.:Ldose: -0.698970004 (uM) Interaction | -70.58925805 | 19.0858749 | -3.698507846 | 0.000217409 |
| MeWo:ABT.737:Ldose: -0.698970004 (uM) Interaction | -62.86632105 | 19.0858749 | -3.293866349 | 0.000989788 |
| SKMEL2:ABT.737:Ldose: -0.698970004 (uM) Interaction | -66.13943049 | 19.0858749 | -3.46536016 | 0.000530553 |
| UACC0257:ABT.737:Ldose: -0.698970004 (uM) Interaction | -58.40837156 | 19.0858749 | -3.060293116 | 0.002213924 |
| MeWo:Streptozocin:Ldose: -0.698970004 (uM) Interaction | -55.01566148 | 19.0858749 | -2.882532856 | 0.003948821 |
| SKMEL2:Streptozocin:Ldose: -0.698970004 (uM) Interaction | -77.08282935 | 19.0858749 | -4.038737012 | 5.39E-05 |
| UACC0257:Streptozocin:Ldose: -0.698970004 (uM) Interaction | -56.69674532 | 19.0858749 | -2.970612855 | 0.00297534 |
| MeWo:Crizotinib:Ldose: -0.698970004 (uM) Interaction | -58.82950239 | 19.0858749 | -3.082358169 | 0.002056269 |
| SKMEL2:Crizotinib:Ldose: -0.698970004 (uM) Interaction | -59.53610927 | 19.0858749 | -3.119380673 | 0.001814707 |
| UACC0257:Crizotinib:Ldose: -0.698970004 (uM) Interaction | -80.95412227 | 19.0858749 | -4.241572509 | 2.23E-05 |
| MeWo:Sunitinib:Ldose: -0.698970004 (uM) Interaction | -56.97954613 | 19.0858749 | -2.985430138 | 0.002834979 |
| SKMEL2:Sunitinib:Ldose: -0.698970004 (uM) Interaction | -64.17933615 | 19.0858749 | -3.362661471 | 0.000773293 |
| UACC0257:Sunitinib:Ldose: -0.698970004 (uM) Interaction | -55.39382582 | 19.0858749 | -2.90234669 | 0.003707538 |
| MeWo:Dexrazoxane:Ldose: -0.698970004 (uM) Interaction | -59.39865533 | 19.0858749 | -3.112178805 | 0.001859549 |
| SKMEL2:Dexrazoxane:Ldose: -0.698970004 (uM) Interaction | -66.30655471 | 19.0858749 | -3.474116595 | 0.000513546 |
| UACC0257:Dexrazoxane:Ldose: -0.698970004 (uM) Interaction | -76.17119182 | 19.0858749 | -3.990971974 | 6.60E-05 |
| MeWo:Mitomycin.C:Ldose: -0.698970004 (uM) Interaction | -58.77930555 | 19.0858749 | -3.079728117 | 0.002074504 |
| SKMEL2:Mitomycin.C:Ldose: -0.698970004 (uM) Interaction | -66.74895831 | 19.0858749 | -3.497296229 | 0.000470946 |
| UACC0257:Mitomycin.C:Ldose: -0.698970004 (uM) Interaction | -77.65768577 | 19.0858749 | -4.068856481 | 4.74E-05 |
| MeWo:Carfilzomib:Ldose: -0.698970004 (uM) Interaction | -93.02327758 | 19.0858749 | -4.873933109 | 1.10E-06 |
| SKMEL2:Carfilzomib:Ldose: -0.698970004 (uM) Interaction | -20.95318677 | 19.0858749 | -1.097837373 | 0.27228778 |
| UACC0257:Carfilzomib:Ldose: -0.698970004 (uM) Interaction | -104.9726568 | 19.0858749 | -5.500018071 | 3.84E-08 |
| MeWo:OSI.027:Ldose: -0.698970004 (uM) Interaction | -78.76341945 | 19.0858749 | -4.126791142 | 3.69E-05 |
| SKMEL2:OSI.027:Ldose: -0.698970004 (uM) Interaction | -76.87988859 | 19.0858749 | -4.028103978 | 5.64E-05 |
| UACC0257:OSI.027:Ldose: -0.698970004 (uM) Interaction | -80.56885003 | 19.0858749 | -4.221386259 | 2.44E-05 |
| MeWo:Bioymifi:Ldose: -0.698970004 (uM) Interaction | -62.02820556 | 19.0858749 | -3.249953481 | 0.00115601 |
| SKMEL2:Bioymifi:Ldose: -0.698970004 (uM) Interaction | -65.43489649 | 19.0858749 | -3.428446264 | 0.000608182 |
| UACC0257:Bioymifi:Ldose: -0.698970004 (uM) Interaction | -59.37873083 | 19.0858749 | -3.111134866 | 0.001866133 |
| MeWo:Nelarabine:Ldose: -0.698970004 (uM) Interaction | -55.09302537 | 19.0858749 | -2.886586319 | 0.00389833 |
| SKMEL2:Nelarabine:Ldose: -0.698970004 (uM) Interaction | -75.73586277 | 19.0858749 | -3.968163009 | 7.27E-05 |
| UACC0257:Nelarabine:Ldose: -0.698970004 (uM) Interaction | -60.97087822 | 19.0858749 | -3.19455506 | 0.001402482 |
| MeWo:Raloxifene:Ldose: -0.698970004 (uM) Interaction | -43.8059081 | 19.0858749 | -2.295200421 | 0.021731206 |
| SKMEL2:Raloxifene:Ldose: -0.698970004 (uM) Interaction | -51.96537036 | 19.0858749 | -2.722713559 | 0.006480028 |
| UACC0257:Raloxifene:Ldose: -0.698970004 (uM) Interaction | -56.57247386 | 19.0858749 | -2.964101681 | 0.003039002 |
| MeWo:Quinacrine.HCl:Ldose: -0.698970004 (uM) Interaction | -67.45561845 | 19.0858749 | -3.534321524 | 0.00040968 |
| SKMEL2:Quinacrine.HCl:Ldose: -0.698970004 (uM) Interaction | -69.45297838 | 19.0858749 | -3.638972736 | 0.000274368 |
| UACC0257:Quinacrine.HCl:Ldose: -0.698970004 (uM) Interaction | -69.01387244 | 19.0858749 | -3.615965881 | 0.000299913 |
| MeWo:Lenalidomide:Ldose: -0.698970004 (uM) Interaction | -58.07265547 | 19.0858749 | -3.042703349 | 0.002347457 |
| SKMEL2:Lenalidomide:Ldose: -0.698970004 (uM) Interaction | -68.7310573 | 19.0858749 | -3.601147847 | 0.000317527 |
| UACC0257:Lenalidomide:Ldose: -0.698970004 (uM) Interaction | -69.67366822 | 19.0858749 | -3.650535729 | 0.000262313 |
| MeWo:Fludarabine.Phosphate:Ldose: -0.698970004 (uM) Interaction | -53.73416143 | 19.0858749 | -2.815388957 | 0.004876246 |
| SKMEL2:Fludarabine.Phosphate:Ldose: -0.698970004 (uM) Interaction | -60.43057276 | 19.0858749 | -3.16624588 | 0.001546354 |
| UACC0257:Fludarabine.Phosphate:Ldose: -0.698970004 (uM) Interaction | -53.10093445 | 19.0858749 | -2.782211176 | 0.005403686 |
| MeWo:Nilotinib:Ldose: -0.698970004 (uM) Interaction | -61.61482241 | 19.0858749 | -3.228294367 | 0.001247172 |
| SKMEL2:Nilotinib:Ldose: -0.698970004 (uM) Interaction | -63.69533856 | 19.0858749 | -3.337302529 | 0.00084739 |
| UACC0257:Nilotinib:Ldose: -0.698970004 (uM) Interaction | -70.48093747 | 19.0858749 | -3.692832414 | 0.000222318 |
| MeWo:Linsitinib:Ldose: -0.698970004 (uM) Interaction | -48.12156769 | 19.0858749 | -2.521318407 | 0.011698755 |
| SKMEL2:Linsitinib:Ldose: -0.698970004 (uM) Interaction | -54.93863764 | 19.0858749 | -2.87849721 | 0.003999679 |
| UACC0257:Linsitinib:Ldose: -0.698970004 (uM) Interaction | -71.04814754 | 19.0858749 | -3.722551255 | 0.000197722 |
| MeWo:Aphrocallistin.analogue:Ldose: -0.698970004 (uM) Interaction | -78.85114078 | 19.0858749 | -4.13138728 | 3.62E-05 |
| SKMEL2:Aphrocallistin.analogue:Ldose: -0.698970004 (uM) Interaction | -69.53932525 | 19.0858749 | -3.64349686 | 0.000269591 |
| UACC0257:Aphrocallistin.analogue:Ldose: -0.698970004 (uM) Interaction | -46.45258137 | 19.0858749 | -2.433872255 | 0.014946338 |
| MeWo:Mitotane..o.p..DDD..Lysodren.:Ldose: -0.698970004 (uM) Interaction | -52.43057359 | 19.0858749 | -2.747087774 | 0.006017701 |
| SKMEL2:Mitotane..o.p..DDD..Lysodren.:Ldose: -0.698970004 (uM) Interaction | -76.26965858 | 19.0858749 | -3.996131118 | 6.46E-05 |
| UACC0257:Mitotane..o.p..DDD..Lysodren.:Ldose: -0.698970004 (uM) Interaction | -59.62025134 | 19.0858749 | -3.123789277 | 0.00178775 |
| MeWo:Etoposide:Ldose: -0.698970004 (uM) Interaction | -48.1805948 | 19.0858749 | -2.524411119 | 0.011596354 |
| SKMEL2:Etoposide:Ldose: -0.698970004 (uM) Interaction | -60.59696038 | 19.0858749 | -3.174963721 | 0.001500662 |
| UACC0257:Etoposide:Ldose: -0.698970004 (uM) Interaction | -45.06798675 | 19.0858749 | -2.36132674 | 0.01821853 |
| MeWo:Vandetanib:Ldose: -0.698970004 (uM) Interaction | -75.39776811 | 19.0858749 | -3.950448618 | 7.83E-05 |
| SKMEL2:Vandetanib:Ldose: -0.698970004 (uM) Interaction | -76.12786413 | 19.0858749 | -3.98870183 | 6.67E-05 |
| UACC0257:Vandetanib:Ldose: -0.698970004 (uM) Interaction | -63.97816437 | 19.0858749 | -3.352121122 | 0.000803329 |
| MeWo:Carboplatin:Ldose: -0.698970004 (uM) Interaction | -57.36317925 | 19.0858749 | -3.005530507 | 0.002654232 |
| SKMEL2:Carboplatin:Ldose: -0.698970004 (uM) Interaction | -65.56498358 | 19.0858749 | -3.435262148 | 0.000593097 |
| UACC0257:Carboplatin:Ldose: -0.698970004 (uM) Interaction | -70.1796411 | 19.0858749 | -3.677046061 | 0.000236524 |
| MeWo:Gefitinib:Ldose: -0.698970004 (uM) Interaction | -55.1561247 | 19.0858749 | -2.889892394 | 0.003857583 |
| SKMEL2:Gefitinib:Ldose: -0.698970004 (uM) Interaction | -67.35781779 | 19.0858749 | -3.529197281 | 0.000417691 |
| UACC0257:Gefitinib:Ldose: -0.698970004 (uM) Interaction | -60.47092112 | 19.0858749 | -3.168359923 | 0.001535158 |
| MeWo:Vincristine.Sulfate:Ldose: -0.698970004 (uM) Interaction | -24.6735048 | 19.0858749 | -1.292762576 | 0.196107054 |
| SKMEL2:Vincristine.Sulfate:Ldose: -0.698970004 (uM) Interaction | -17.03428698 | 19.0858749 | -0.892507526 | 0.372130931 |
| UACC0257:Vincristine.Sulfate:Ldose: -0.698970004 (uM) Interaction | -19.90642255 | 19.0858749 | -1.042992405 | 0.296963516 |
| MeWo:Trametinib..GSK1120212.:Ldose: -0.698970004 (uM) Interaction | -63.55653156 | 19.0858749 | -3.330029768 | 0.000869825 |
| SKMEL2:Trametinib..GSK1120212.:Ldose: -0.698970004 (uM) Interaction | -67.12756558 | 19.0858749 | -3.51713327 | 0.000437132 |
| UACC0257:Trametinib..GSK1120212.:Ldose: -0.698970004 (uM) Interaction | -57.34851815 | 19.0858749 | -3.004762342 | 0.002660941 |
| MeWo:MLN4924:Ldose: -0.698970004 (uM) Interaction | -79.32747815 | 19.0858749 | -4.156344866 | 3.25E-05 |
| SKMEL2:MLN4924:Ldose: -0.698970004 (uM) Interaction | -54.07252559 | 19.0858749 | -2.83311747 | 0.004613926 |
| UACC0257:MLN4924:Ldose: -0.698970004 (uM) Interaction | -56.42713852 | 19.0858749 | -2.956486869 | 0.003115028 |
| MeWo:Bortezomib:Ldose: -0.698970004 (uM) Interaction | -50.78927234 | 19.0858749 | -2.661092175 | 0.007794538 |
| SKMEL2:Bortezomib:Ldose: -0.698970004 (uM) Interaction | -48.6101111 | 19.0858749 | -2.546915526 | 0.010874867 |
| UACC0257:Bortezomib:Ldose: -0.698970004 (uM) Interaction | -47.36267652 | 19.0858749 | -2.481556479 | 0.013088566 |
| MeWo:Fluorouracil...5.FU.:Ldose: -0.698970004 (uM) Interaction | -55.50588198 | 19.0858749 | -2.908217846 | 0.003638662 |
| SKMEL2:Fluorouracil...5.FU.:Ldose: -0.698970004 (uM) Interaction | -65.25866128 | 19.0858749 | -3.419212462 | 0.00062919 |
| UACC0257:Fluorouracil...5.FU.:Ldose: -0.698970004 (uM) Interaction | -60.91874725 | 19.0858749 | -3.19182367 | 0.001415805 |
| MeWo:Lapatinib:Ldose: -0.698970004 (uM) Interaction | -70.29689091 | 19.0858749 | -3.683189337 | 0.000230897 |
| SKMEL2:Lapatinib:Ldose: -0.698970004 (uM) Interaction | -76.82781528 | 19.0858749 | -4.025375609 | 5.71E-05 |
| UACC0257:Lapatinib:Ldose: -0.698970004 (uM) Interaction | -70.56858912 | 19.0858749 | -3.697424902 | 0.000218338 |
| MeWo:Mitoxantrone:Ldose: -0.698970004 (uM) Interaction | -21.16708104 | 19.0858749 | -1.109044314 | 0.267423448 |
| SKMEL2:Mitoxantrone:Ldose: -0.698970004 (uM) Interaction | -17.86609236 | 19.0858749 | -0.936089776 | 0.349237461 |
| UACC0257:Mitoxantrone:Ldose: -0.698970004 (uM) Interaction | -33.47265159 | 19.0858749 | -1.753791837 | 0.079480403 |
| MeWo:Imatinib:Ldose: -0.698970004 (uM) Interaction | -58.33865402 | 19.0858749 | -3.056640282 | 0.002241068 |
| SKMEL2:Imatinib:Ldose: -0.698970004 (uM) Interaction | -64.04363527 | 19.0858749 | -3.355551455 | 0.000793437 |
| UACC0257:Imatinib:Ldose: -0.698970004 (uM) Interaction | -59.15645303 | 19.0858749 | -3.099488672 | 0.001941048 |
| MeWo:Imiquimod:Ldose: -0.698970004 (uM) Interaction | -57.44093389 | 19.0858749 | -3.009604443 | 0.002618908 |
| SKMEL2:Imiquimod:Ldose: -0.698970004 (uM) Interaction | -60.94224359 | 19.0858749 | -3.193054755 | 0.001409786 |
| UACC0257:Imiquimod:Ldose: -0.698970004 (uM) Interaction | -59.25348134 | 19.0858749 | -3.104572448 | 0.001908013 |
| MeWo:Dacomitinib..PF299804.:Ldose: -0.698970004 (uM) Interaction | -61.65122172 | 19.0858749 | -3.2302015 | 0.001238886 |
| SKMEL2:Dacomitinib..PF299804.:Ldose: -0.698970004 (uM) Interaction | -61.36588672 | 19.0858749 | -3.215251439 | 0.001305225 |
| UACC0257:Dacomitinib..PF299804.:Ldose: -0.698970004 (uM) Interaction | -58.02152449 | 19.0858749 | -3.040024354 | 0.002368429 |
| MeWo:PD325901:Ldose: -0.698970004 (uM) Interaction | -75.13793982 | 19.0858749 | -3.936834975 | 8.28E-05 |
| SKMEL2:PD325901:Ldose: -0.698970004 (uM) Interaction | -88.68885202 | 19.0858749 | -4.646831885 | 3.39E-06 |
| UACC0257:PD325901:Ldose: -0.698970004 (uM) Interaction | -67.60940964 | 19.0858749 | -3.542379378 | 0.000397373 |
| MeWo:Vismodegib:Ldose: -0.698970004 (uM) Interaction | -57.53577834 | 19.0858749 | -3.014573796 | 0.002576401 |
| SKMEL2:Vismodegib:Ldose: -0.698970004 (uM) Interaction | -78.04562812 | 19.0858749 | -4.089182631 | 4.34E-05 |
| UACC0257:Vismodegib:Ldose: -0.698970004 (uM) Interaction | -48.21981297 | 19.0858749 | -2.526465946 | 0.011528758 |
| MeWo:Temozolomide:Ldose: -0.698970004 (uM) Interaction | -60.19051432 | 19.0858749 | -3.153668074 | 0.001614538 |
| SKMEL2:Temozolomide:Ldose: -0.698970004 (uM) Interaction | -66.4102463 | 19.0858749 | -3.479549492 | 0.00050325 |
| UACC0257:Temozolomide:Ldose: -0.698970004 (uM) Interaction | -70.86799616 | 19.0858749 | -3.713112265 | 0.000205242 |
| MeWo:Mercaptopurine:Ldose: -0.698970004 (uM) Interaction | -53.56123013 | 19.0858749 | -2.806328262 | 0.005015459 |
| SKMEL2:Mercaptopurine:Ldose: -0.698970004 (uM) Interaction | -55.89602643 | 19.0858749 | -2.928659374 | 0.003407839 |
| UACC0257:Mercaptopurine:Ldose: -0.698970004 (uM) Interaction | -34.96801299 | 19.0858749 | -1.832140951 | 0.066944223 |
| MeWo:Dasatinib:Ldose: -0.698970004 (uM) Interaction | -68.63726373 | 19.0858749 | -3.596233555 | 0.00032358 |
| SKMEL2:Dasatinib:Ldose: -0.698970004 (uM) Interaction | -81.36517543 | 19.0858749 | -4.263109544 | 2.02E-05 |
| UACC0257:Dasatinib:Ldose: -0.698970004 (uM) Interaction | -66.07252478 | 19.0858749 | -3.461854651 | 0.000537507 |
| MeWo:Daunorubicin.HCl:Ldose: -0.698970004 (uM) Interaction | -22.11610012 | 19.0858749 | -1.15876795 | 0.246563631 |
| SKMEL2:Daunorubicin.HCl:Ldose: -0.698970004 (uM) Interaction | -25.05198809 | 19.0858749 | -1.312593121 | 0.189334074 |
| UACC0257:Daunorubicin.HCl:Ldose: -0.698970004 (uM) Interaction | -44.94626967 | 19.0858749 | -2.354949402 | 0.018534148 |
| MeWo:Sirolimus..Rapamycin.:Ldose: -0.698970004 (uM) Interaction | -64.59625124 | 19.0858749 | -3.38450564 | 0.000714337 |
| SKMEL2:Sirolimus..Rapamycin.:Ldose: -0.698970004 (uM) Interaction | -71.84832446 | 19.0858749 | -3.764476339 | 0.000167342 |
| UACC0257:Sirolimus..Rapamycin.:Ldose: -0.698970004 (uM) Interaction | -75.0803546 | 19.0858749 | -3.933817811 | 8.39E-05 |
| MeWo:INK.128..MLN0128.:Ldose: -0.698970004 (uM) Interaction | -36.51331679 | 19.0858749 | -1.913106787 | 0.055747627 |
| SKMEL2:INK.128..MLN0128.:Ldose: -0.698970004 (uM) Interaction | -57.04552547 | 19.0858749 | -2.988887111 | 0.002803114 |
| UACC0257:INK.128..MLN0128.:Ldose: -0.698970004 (uM) Interaction | -61.24680625 | 19.0858749 | -3.209012245 | 0.001333868 |
| MeWo:Quizartinib:Ldose: -0.698970004 (uM) Interaction | -55.88828422 | 19.0858749 | -2.928253723 | 0.003412286 |
| SKMEL2:Quizartinib:Ldose: -0.698970004 (uM) Interaction | -60.51774315 | 19.0858749 | -3.170813153 | 0.001522258 |
| UACC0257:Quizartinib:Ldose: -0.698970004 (uM) Interaction | -61.29581958 | 19.0858749 | -3.211580287 | 0.001322009 |
| MeWo:Sorafenib:Ldose: -0.698970004 (uM) Interaction | -55.51145597 | 19.0858749 | -2.908509894 | 0.003635267 |
| SKMEL2:Sorafenib:Ldose: -0.698970004 (uM) Interaction | -77.89776567 | 19.0858749 | -4.081435412 | 4.49E-05 |
| UACC0257:Sorafenib:Ldose: -0.698970004 (uM) Interaction | -56.52633308 | 19.0858749 | -2.961684145 | 0.003062953 |
| MeWo:Carmustine:Ldose: -0.698970004 (uM) Interaction | -52.01362037 | 19.0858749 | -2.725241607 | 0.006430633 |
| SKMEL2:Carmustine:Ldose: -0.698970004 (uM) Interaction | -60.07181156 | 19.0858749 | -3.14744867 | 0.001649266 |
| UACC0257:Carmustine:Ldose: -0.698970004 (uM) Interaction | -75.17891458 | 19.0858749 | -3.938981838 | 8.21E-05 |
| MeWo:Uracil.mustard:Ldose: -0.698970004 (uM) Interaction | -52.79080908 | 19.0858749 | -2.765962229 | 0.005680345 |
| SKMEL2:Uracil.mustard:Ldose: -0.698970004 (uM) Interaction | -60.85541572 | 19.0858749 | -3.188505429 | 0.001432148 |
| UACC0257:Uracil.mustard:Ldose: -0.698970004 (uM) Interaction | -68.4775008 | 19.0858749 | -3.587862813 | 0.000334139 |
| MeWo:Ixabepilone:Ldose: -0.698970004 (uM) Interaction | -52.52922391 | 19.0858749 | -2.752256535 | 0.005923566 |
| SKMEL2:Ixabepilone:Ldose: -0.698970004 (uM) Interaction | -55.44383716 | 19.0858749 | -2.904967022 | 0.003676653 |
| UACC0257:Ixabepilone:Ldose: -0.698970004 (uM) Interaction | -77.38685394 | 19.0858749 | -4.05466631 | 5.04E-05 |
| MeWo:Valrubicin:Ldose: -0.698970004 (uM) Interaction | -42.78269082 | 19.0858749 | -2.241589188 | 0.024998076 |
| SKMEL2:Valrubicin:Ldose: -0.698970004 (uM) Interaction | -58.64018356 | 19.0858749 | -3.072438852 | 0.002125822 |
| UACC0257:Valrubicin:Ldose: -0.698970004 (uM) Interaction | -55.22023133 | 19.0858749 | -2.893251246 | 0.003816583 |
| MeWo:Triethylenemelamine:Ldose: -0.698970004 (uM) Interaction | -61.8089149 | 19.0858749 | -3.238463798 | 0.001203573 |
| SKMEL2:Triethylenemelamine:Ldose: -0.698970004 (uM) Interaction | -66.45550358 | 19.0858749 | -3.481920737 | 0.000498818 |
| UACC0257:Triethylenemelamine:Ldose: -0.698970004 (uM) Interaction | -66.70161704 | 19.0858749 | -3.494815795 | 0.000475342 |
| MeWo:Palbociclib..PD.0332991..Isethionate:Ldose: -0.698970004 (uM) Interaction | -50.5497338 | 19.0858749 | -2.648541608 | 0.008089883 |
| SKMEL2:Palbociclib..PD.0332991..Isethionate:Ldose: -0.698970004 (uM) Interaction | -51.11439363 | 19.0858749 | -2.67812683 | 0.007409134 |
| UACC0257:Palbociclib..PD.0332991..Isethionate:Ldose: -0.698970004 (uM) Interaction | -61.28715084 | 19.0858749 | -3.21112609 | 0.0013241 |
| MeWo:Afatinib:Ldose: -0.698970004 (uM) Interaction | -59.15063764 | 19.0858749 | -3.099183976 | 0.001943044 |
| SKMEL2:Afatinib:Ldose: -0.698970004 (uM) Interaction | -64.1204331 | 19.0858749 | -3.359575259 | 0.000781978 |
| UACC0257:Afatinib:Ldose: -0.698970004 (uM) Interaction | -64.76724188 | 19.0858749 | -3.393464656 | 0.000691387 |
| MeWo:Doxorubicin.HCl:Ldose: -0.698970004 (uM) Interaction | -28.88175368 | 19.0858749 | -1.513252803 | 0.130230128 |
| SKMEL2:Doxorubicin.HCl:Ldose: -0.698970004 (uM) Interaction | -55.40530073 | 19.0858749 | -2.902947914 | 0.003700431 |
| UACC0257:Doxorubicin.HCl:Ldose: -0.698970004 (uM) Interaction | -43.1022134 | 19.0858749 | -2.2583305 | 0.023935023 |
| MeWo:Exemestane:Ldose: -0.698970004 (uM) Interaction | -57.99074637 | 19.0858749 | -3.038411741 | 0.002381137 |
| SKMEL2:Exemestane:Ldose: -0.698970004 (uM) Interaction | -68.57781657 | 19.0858749 | -3.593118835 | 0.000327472 |
| UACC0257:Exemestane:Ldose: -0.698970004 (uM) Interaction | -62.38317972 | 19.0858749 | -3.26855227 | 0.001082686 |
| MeWo:Tretinoin:Ldose: -0.698970004 (uM) Interaction | -50.81439379 | 19.0858749 | -2.662408407 | 0.007764131 |
| SKMEL2:Tretinoin:Ldose: -0.698970004 (uM) Interaction | -56.12411929 | 19.0858749 | -2.940610247 | 0.003279147 |
| UACC0257:Tretinoin:Ldose: -0.698970004 (uM) Interaction | -52.14240045 | 19.0858749 | -2.731989009 | 0.006300453 |
| MeWo:Fulvestrant:Ldose: -0.698970004 (uM) Interaction | -62.95871397 | 19.0858749 | -3.298707254 | 0.000972886 |
| SKMEL2:Fulvestrant:Ldose: -0.698970004 (uM) Interaction | -70.79725289 | 19.0858749 | -3.709405688 | 0.000208268 |
| UACC0257:Fulvestrant:Ldose: -0.698970004 (uM) Interaction | -64.59244713 | 19.0858749 | -3.384306325 | 0.000714856 |
| MeWo:Docetaxel:Ldose: -0.698970004 (uM) Interaction | -45.87100204 | 19.0858749 | -2.403400541 | 0.016251783 |
| SKMEL2:Docetaxel:Ldose: -0.698970004 (uM) Interaction | -67.05197604 | 19.0858749 | -3.513172773 | 0.000443697 |
| UACC0257:Docetaxel:Ldose: -0.698970004 (uM) Interaction | -55.74153647 | 19.0858749 | -2.920564909 | 0.003497597 |
| MeWo:Everolimus:Ldose: -0.698970004 (uM) Interaction | -62.86015371 | 19.0858749 | -3.293543212 | 0.000990926 |
| SKMEL2:Everolimus:Ldose: -0.698970004 (uM) Interaction | -64.71997341 | 19.0858749 | -3.390988035 | 0.000697662 |
| UACC0257:Everolimus:Ldose: -0.698970004 (uM) Interaction | -63.93954871 | 19.0858749 | -3.350097864 | 0.000809217 |
| MeWo:MLN.2480:Ldose: -0.698970004 (uM) Interaction | -49.91446339 | 19.0858749 | -2.615256763 | 0.008922253 |
| SKMEL2:MLN.2480:Ldose: -0.698970004 (uM) Interaction | -53.64694817 | 19.0858749 | -2.810819439 | 0.004946011 |
| UACC0257:MLN.2480:Ldose: -0.698970004 (uM) Interaction | -62.39989722 | 19.0858749 | -3.26942818 | 0.001079341 |
| MeWo:LY2157299:Ldose: -0.698970004 (uM) Interaction | -63.45531669 | 19.0858749 | -3.324726639 | 0.00088653 |
| SKMEL2:LY2157299:Ldose: -0.698970004 (uM) Interaction | -69.03118001 | 19.0858749 | -3.616872708 | 0.000298865 |
| UACC0257:LY2157299:Ldose: -0.698970004 (uM) Interaction | -51.63442101 | 19.0858749 | -2.705373544 | 0.006828129 |
| MeWo:Allopurinol:Ldose: -0.698970004 (uM) Interaction | -62.04957569 | 19.0858749 | -3.251073164 | 0.001151469 |
| SKMEL2:Allopurinol:Ldose: -0.698970004 (uM) Interaction | -74.31077634 | 19.0858749 | -3.893495936 | 9.91E-05 |
| UACC0257:Allopurinol:Ldose: -0.698970004 (uM) Interaction | -49.75116177 | 19.0858749 | -2.606700612 | 0.009148222 |
| MeWo:Pipobroman:Ldose: -0.698970004 (uM) Interaction | -51.67090593 | 19.0858749 | -2.707285163 | 0.006788945 |
| SKMEL2:Pipobroman:Ldose: -0.698970004 (uM) Interaction | -62.80954777 | 19.0858749 | -3.290891725 | 0.001000308 |
| UACC0257:Pipobroman:Ldose: -0.698970004 (uM) Interaction | -48.006903 | 19.0858749 | -2.515310577 | 0.011899973 |
| MeWo:Letrozole:Ldose: -0.698970004 (uM) Interaction | -54.49520705 | 19.0858749 | -2.855263767 | 0.004304218 |
| SKMEL2:Letrozole:Ldose: -0.698970004 (uM) Interaction | -61.12382832 | 19.0858749 | -3.202568845 | 0.001364057 |
| UACC0257:Letrozole:Ldose: -0.698970004 (uM) Interaction | -57.23328923 | 19.0858749 | -2.998724949 | 0.002714215 |
| MeWo:Thiotepa:Ldose: -0.698970004 (uM) Interaction | -55.33343754 | 19.0858749 | -2.899182659 | 0.003745146 |
| SKMEL2:Thiotepa:Ldose: -0.698970004 (uM) Interaction | -67.31908198 | 19.0858749 | -3.527167727 | 0.000420904 |
| UACC0257:Thiotepa:Ldose: -0.698970004 (uM) Interaction | -50.88606538 | 19.0858749 | -2.666163624 | 0.007677962 |
| MeWo:Plicamycin:Ldose: -0.698970004 (uM) Interaction | -35.73255992 | 19.0858749 | -1.872199211 | 0.061192587 |
| SKMEL2:Plicamycin:Ldose: -0.698970004 (uM) Interaction | -49.60310346 | 19.0858749 | -2.598943131 | 0.009357499 |
| UACC0257:Plicamycin:Ldose: -0.698970004 (uM) Interaction | -59.16026779 | 19.0858749 | -3.099688545 | 0.001939739 |
| MeWo:Erlotinib.HCl:Ldose: -0.698970004 (uM) Interaction | -78.13394894 | 19.0858749 | -4.09381018 | 4.26E-05 |
| SKMEL2:Erlotinib.HCl:Ldose: -0.698970004 (uM) Interaction | -84.53164557 | 19.0858749 | -4.429016015 | 9.51E-06 |
| UACC0257:Erlotinib.HCl:Ldose: -0.698970004 (uM) Interaction | -79.14281121 | 19.0858749 | -4.146669285 | 3.39E-05 |
| MeWo:MEK.162..ARRY.438162.:Ldose: -0.698970004 (uM) Interaction | -27.53127841 | 19.0858749 | -1.442494963 | 0.149177332 |
| SKMEL2:MEK.162..ARRY.438162.:Ldose: -0.698970004 (uM) Interaction | -18.18223199 | 19.0858749 | -0.952653839 | 0.340776179 |
| UACC0257:MEK.162..ARRY.438162.:Ldose: -0.698970004 (uM) Interaction | -35.56899947 | 19.0858749 | -1.863629499 | 0.062387349 |
| MeWo:Baricitinib..LY3009104..INCB028050.:Ldose: -0.698970004 (uM) Interaction | -48.43305199 | 19.0858749 | -2.537638555 | 0.011167306 |
| SKMEL2:Baricitinib..LY3009104..INCB028050.:Ldose: -0.698970004 (uM) Interaction | -49.02653773 | 19.0858749 | -2.568734103 | 0.010213747 |
| UACC0257:Baricitinib..LY3009104..INCB028050.:Ldose: -0.698970004 (uM) Interaction | -56.50689153 | 19.0858749 | -2.96066551 | 0.003073097 |
| MeWo:Arsenic.Trioxide:Ldose: -0.698970004 (uM) Interaction | -54.67846022 | 19.0858749 | -2.864865274 | 0.004175901 |
| SKMEL2:Arsenic.Trioxide:Ldose: -0.698970004 (uM) Interaction | -65.78340902 | 19.0858749 | -3.446706498 | 0.000568547 |
| UACC0257:Arsenic.Trioxide:Ldose: -0.698970004 (uM) Interaction | -47.79191521 | 19.0858749 | -2.504046342 | 0.012285529 |
| MeWo:Celecoxib:Ldose: -0.698970004 (uM) Interaction | -54.43058052 | 19.0858749 | -2.851877675 | 0.004350316 |
| SKMEL2:Celecoxib:Ldose: -0.698970004 (uM) Interaction | -66.192576 | 19.0858749 | -3.468144707 | 0.000525088 |
| UACC0257:Celecoxib:Ldose: -0.698970004 (uM) Interaction | -58.75737826 | 19.0858749 | -3.078579241 | 0.002082516 |
| MeWo:Bendamustine.HCl:Ldose: -0.698970004 (uM) Interaction | -63.71624255 | 19.0858749 | -3.338397789 | 0.000844058 |
| SKMEL2:Bendamustine.HCl:Ldose: -0.698970004 (uM) Interaction | -63.96166868 | 19.0858749 | -3.351256834 | 0.000805839 |
| UACC0257:Bendamustine.HCl:Ldose: -0.698970004 (uM) Interaction | -66.14220227 | 19.0858749 | -3.465505387 | 0.000530266 |
| MeWo:Chlorambucil:Ldose: -0.698970004 (uM) Interaction | -56.11933532 | 19.0858749 | -2.940359592 | 0.0032818 |
| SKMEL2:Chlorambucil:Ldose: -0.698970004 (uM) Interaction | -58.90955546 | 19.0858749 | -3.086552531 | 0.002027491 |
| UACC0257:Chlorambucil:Ldose: -0.698970004 (uM) Interaction | -57.13749197 | 19.0858749 | -2.993705674 | 0.002759244 |
| MeWo:Zoledronic.Acid:Ldose: -0.698970004 (uM) Interaction | -56.13548462 | 19.0858749 | -2.941205731 | 0.003272851 |
| SKMEL2:Zoledronic.Acid:Ldose: -0.698970004 (uM) Interaction | -68.25247056 | 19.0858749 | -3.576072406 | 0.000349559 |
| UACC0257:Zoledronic.Acid:Ldose: -0.698970004 (uM) Interaction | -62.58335149 | 19.0858749 | -3.279040224 | 0.00104326 |
| MeWo:Actinomycin.D:Ldose: -0.698970004 (uM) Interaction | -51.65591942 | 19.0858749 | -2.706499948 | 0.006805016 |
| SKMEL2:Actinomycin.D:Ldose: -0.698970004 (uM) Interaction | -109.6622419 | 19.0858749 | -5.745727795 | 9.28E-09 |
| UACC0257:Actinomycin.D:Ldose: -0.698970004 (uM) Interaction | -80.9034646 | 19.0858749 | -4.238918312 | 2.26E-05 |
| MeWo:Temsirolimus..CCI.779..Torisel.:Ldose: -0.698970004 (uM) Interaction | -53.3394628 | 19.0858749 | -2.794708814 | 0.005199241 |
| SKMEL2:Temsirolimus..CCI.779..Torisel.:Ldose: -0.698970004 (uM) Interaction | -56.24978185 | 19.0858749 | -2.947194308 | 0.003210153 |
| UACC0257:Temsirolimus..CCI.779..Torisel.:Ldose: -0.698970004 (uM) Interaction | -43.58080987 | 19.0858749 | -2.283406452 | 0.02241613 |
| MeWo:Foretinib..GSK1363089.:Ldose: -0.698970004 (uM) Interaction | -70.62930528 | 19.0858749 | -3.700606111 | 0.000215621 |
| SKMEL2:Foretinib..GSK1363089.:Ldose: -0.698970004 (uM) Interaction | -53.10212987 | 19.0858749 | -2.782273811 | 0.005402644 |
| UACC0257:Foretinib..GSK1363089.:Ldose: -0.698970004 (uM) Interaction | -49.33813187 | 19.0858749 | -2.585060006 | 0.009742715 |
| MeWo:Decitabine:Ldose: -0.698970004 (uM) Interaction | -60.21790655 | 19.0858749 | -3.155103283 | 0.00160662 |
| SKMEL2:Decitabine:Ldose: -0.698970004 (uM) Interaction | -77.60064499 | 19.0858749 | -4.065867843 | 4.80E-05 |
| UACC0257:Decitabine:Ldose: -0.698970004 (uM) Interaction | -53.37115514 | 19.0858749 | -2.796369327 | 0.005172609 |
| MeWo:Methotrexate:Ldose: -0.698970004 (uM) Interaction | -56.72007172 | 19.0858749 | -2.971835037 | 0.002963527 |
| SKMEL2:Methotrexate:Ldose: -0.698970004 (uM) Interaction | -67.09342844 | 19.0858749 | -3.515344662 | 0.000440086 |
| UACC0257:Methotrexate:Ldose: -0.698970004 (uM) Interaction | -58.79918589 | 19.0858749 | -3.080769743 | 0.002067264 |
| MeWo:Axitinib:Ldose: -0.698970004 (uM) Interaction | -57.46794318 | 19.0858749 | -3.011019589 | 0.002606738 |
| SKMEL2:Axitinib:Ldose: -0.698970004 (uM) Interaction | -72.57672778 | 19.0858749 | -3.802640863 | 0.000143557 |
| UACC0257:Axitinib:Ldose: -0.698970004 (uM) Interaction | -64.17139747 | 19.0858749 | -3.362245525 | 0.000774459 |
| MeWo:Oxaliplatin:Ldose: -0.698970004 (uM) Interaction | -59.42798163 | 19.0858749 | -3.11371535 | 0.001849897 |
| SKMEL2:Oxaliplatin:Ldose: -0.698970004 (uM) Interaction | -60.5921472 | 19.0858749 | -3.174711536 | 0.001501966 |
| UACC0257:Oxaliplatin:Ldose: -0.698970004 (uM) Interaction | -73.45937461 | 19.0858749 | -3.848886939 | 0.000118998 |
| MeWo:Cabazitaxel:Ldose: -0.698970004 (uM) Interaction | -52.45165332 | 19.0858749 | -2.748192242 | 0.005997473 |
| SKMEL2:Cabazitaxel:Ldose: -0.698970004 (uM) Interaction | -62.21196811 | 19.0858749 | -3.259581677 | 0.001117497 |
| UACC0257:Cabazitaxel:Ldose: -0.698970004 (uM) Interaction | -69.50024432 | 19.0858749 | -3.641449224 | 0.000271743 |
| MeWo:Amifostine:Ldose: -0.698970004 (uM) Interaction | -66.8942715 | 19.0858749 | -3.504909881 | 0.00045769 |
| SKMEL2:Amifostine:Ldose: -0.698970004 (uM) Interaction | -73.03181541 | 19.0858749 | -3.826485074 | 0.000130353 |
| UACC0257:Amifostine:Ldose: -0.698970004 (uM) Interaction | -65.58663592 | 19.0858749 | -3.436396617 | 0.00059062 |
| MeWo:Flutamide..Eulexin.:Ldose: -0.698970004 (uM) Interaction | -50.15375597 | 19.0858749 | -2.627794442 | 0.008600135 |
| SKMEL2:Flutamide..Eulexin.:Ldose: -0.698970004 (uM) Interaction | -60.61712624 | 19.0858749 | -3.176020307 | 0.001495209 |
| UACC0257:Flutamide..Eulexin.:Ldose: -0.698970004 (uM) Interaction | -63.96231859 | 19.0858749 | -3.351290886 | 0.00080574 |
| MeWo:LDK378:Ldose: -0.698970004 (uM) Interaction | -46.85803237 | 19.0858749 | -2.455115766 | 0.014091769 |
| SKMEL2:LDK378:Ldose: -0.698970004 (uM) Interaction | -58.81503813 | 19.0858749 | -3.081600317 | 0.002061508 |
| UACC0257:LDK378:Ldose: -0.698970004 (uM) Interaction | -67.10372319 | 19.0858749 | -3.515884053 | 0.000439193 |
| MeWo:Pralatrexate:Ldose: -0.698970004 (uM) Interaction | -57.51759765 | 19.0858749 | -3.013621223 | 0.0025845 |
| SKMEL2:Pralatrexate:Ldose: -0.698970004 (uM) Interaction | -64.23646382 | 19.0858749 | -3.365654662 | 0.000764956 |
| UACC0257:Pralatrexate:Ldose: -0.698970004 (uM) Interaction | -68.32159618 | 19.0858749 | -3.579694227 | 0.000344753 |
| MeWo:Topotecan.HCl:Ldose: -0.698970004 (uM) Interaction | -4.858343024 | 19.0858749 | -0.254551759 | 0.799071753 |
| SKMEL2:Topotecan.HCl:Ldose: -0.698970004 (uM) Interaction | -26.50742029 | 19.0858749 | -1.388850154 | 0.16489268 |
| UACC0257:Topotecan.HCl:Ldose: -0.698970004 (uM) Interaction | -41.80172767 | 19.0858749 | -2.190191851 | 0.028521009 |
| MeWo:Pemetrexed:Ldose: -0.698970004 (uM) Interaction | -51.47577325 | 19.0858749 | -2.697061232 | 0.007000883 |
| SKMEL2:Pemetrexed:Ldose: -0.698970004 (uM) Interaction | -58.21357821 | 19.0858749 | -3.050086964 | 0.00229053 |
| UACC0257:Pemetrexed:Ldose: -0.698970004 (uM) Interaction | -62.26910236 | 19.0858749 | -3.262575213 | 0.001105767 |
| MeWo:Bleomycin.Sulfate:Ldose: -0.698970004 (uM) Interaction | -36.23569524 | 19.0858749 | -1.89856087 | 0.057635589 |
| SKMEL2:Bleomycin.Sulfate:Ldose: -0.698970004 (uM) Interaction | -53.15123229 | 19.0858749 | -2.78484652 | 0.005359981 |
| UACC0257:Bleomycin.Sulfate:Ldose: -0.698970004 (uM) Interaction | -36.6490858 | 19.0858749 | -1.920220372 | 0.054843259 |
| MeWo:Axitinib.1:Ldose: -0.698970004 (uM) Interaction | -56.03310944 | 19.0858749 | -2.935841807 | 0.003329955 |
| SKMEL2:Axitinib.1:Ldose: -0.698970004 (uM) Interaction | -59.91172825 | 19.0858749 | -3.139061142 | 0.001697189 |
| UACC0257:Axitinib.1:Ldose: -0.698970004 (uM) Interaction | -61.04491396 | 19.0858749 | -3.198434145 | 0.001383759 |
| MeWo:Ibrutinib..PCI.32765.:Ldose: -0.698970004 (uM) Interaction | -61.7213026 | 19.0858749 | -3.233873372 | 0.001223076 |
| SKMEL2:Ibrutinib..PCI.32765.:Ldose: -0.698970004 (uM) Interaction | -68.53659572 | 19.0858749 | -3.590959078 | 0.000330196 |
| UACC0257:Ibrutinib..PCI.32765.:Ldose: -0.698970004 (uM) Interaction | -59.74892873 | 19.0858749 | -3.130531299 | 0.001747236 |
| MeWo:Tamoxifen.Citrate:Ldose: -0.698970004 (uM) Interaction | -50.18016847 | 19.0858749 | -2.629178319 | 0.008565225 |
| SKMEL2:Tamoxifen.Citrate:Ldose: -0.698970004 (uM) Interaction | -58.45509611 | 19.0858749 | -3.062741238 | 0.002195901 |
| UACC0257:Tamoxifen.Citrate:Ldose: -0.698970004 (uM) Interaction | -62.38259389 | 19.0858749 | -3.268521576 | 0.001082803 |
| MeWo:Vemurafenib:Ldose: -0.698970004 (uM) Interaction | -13.36569324 | 19.0858749 | -0.700292405 | 0.483752277 |
| SKMEL2:Vemurafenib:Ldose: -0.698970004 (uM) Interaction | -24.22829169 | 19.0858749 | -1.269435738 | 0.204299412 |
| UACC0257:Vemurafenib:Ldose: -0.698970004 (uM) Interaction | -33.6436851 | 19.0858749 | -1.762753098 | 0.07795632 |
| MeWo:Pazopanib.HCl:Ldose: -0.698970004 (uM) Interaction | -55.34419519 | 19.0858749 | -2.899746304 | 0.003738421 |
| SKMEL2:Pazopanib.HCl:Ldose: -0.698970004 (uM) Interaction | -63.48998812 | 19.0858749 | -3.32654324 | 0.000880774 |
| UACC0257:Pazopanib.HCl:Ldose: -0.698970004 (uM) Interaction | -56.28288351 | 19.0858749 | -2.948928661 | 0.003192201 |
| MeWo:Abiraterone:Ldose: -0.698970004 (uM) Interaction | -57.35844495 | 19.0858749 | -3.005282454 | 0.002656397 |
| SKMEL2:Abiraterone:Ldose: -0.698970004 (uM) Interaction | -66.77418222 | 19.0858749 | -3.498617831 | 0.00046862 |
| UACC0257:Abiraterone:Ldose: -0.698970004 (uM) Interaction | -66.85654596 | 19.0858749 | -3.502933259 | 0.000461097 |
| MeWo:Bosutinib..SKI.606.:Ldose: -0.698970004 (uM) Interaction | -50.74297987 | 19.0858749 | -2.658666692 | 0.007850851 |
| SKMEL2:Bosutinib..SKI.606.:Ldose: -0.698970004 (uM) Interaction | -54.57281891 | 19.0858749 | -2.859330223 | 0.004249443 |
| UACC0257:Bosutinib..SKI.606.:Ldose: -0.698970004 (uM) Interaction | -60.97994253 | 19.0858749 | -3.195029982 | 0.001400178 |
| MeWo:Sabutoclax..BI.97C1.:Ldose: -0.698970004 (uM) Interaction | -58.08392223 | 19.0858749 | -3.043293668 | 0.002342858 |
| SKMEL2:Sabutoclax..BI.97C1.:Ldose: -0.698970004 (uM) Interaction | -60.14120996 | 19.0858749 | -3.151084783 | 0.00162888 |
| UACC0257:Sabutoclax..BI.97C1.:Ldose: -0.698970004 (uM) Interaction | -50.40836304 | 19.0858749 | -2.641134521 | 0.008268854 |
| MeWo:Thioguanine:Ldose: -0.397940009 (uM) Interaction | -61.15352109 | 19.0858749 | -3.20412459 | 0.001356711 |
| SKMEL2:Thioguanine:Ldose: -0.397940009 (uM) Interaction | -63.30155055 | 19.0858749 | -3.316670098 | 0.000912478 |
| UACC0257:Thioguanine:Ldose: -0.397940009 (uM) Interaction | -66.52843037 | 19.0858749 | -3.485741719 | 0.000491751 |
| MeWo:Irinotecan.HCl:Ldose: -0.397940009 (uM) Interaction | -60.22725161 | 19.0858749 | -3.155592916 | 0.001603927 |
| SKMEL2:Irinotecan.HCl:Ldose: -0.397940009 (uM) Interaction | -57.37052732 | 19.0858749 | -3.005915507 | 0.002650875 |
| UACC0257:Irinotecan.HCl:Ldose: -0.397940009 (uM) Interaction | -61.06855144 | 19.0858749 | -3.199672625 | 0.001377831 |
| MeWo:Romidepsin:Ldose: -0.397940009 (uM) Interaction | -62.84083131 | 19.0858749 | -3.29253082 | 0.000994499 |
| SKMEL2:Romidepsin:Ldose: -0.397940009 (uM) Interaction | -60.32838186 | 19.0858749 | -3.160891612 | 0.001575048 |
| UACC0257:Romidepsin:Ldose: -0.397940009 (uM) Interaction | -69.27005447 | 19.0858749 | -3.62938848 | 0.000284751 |
| MeWo:Paclitaxel:Ldose: -0.397940009 (uM) Interaction | -52.34630129 | 19.0858749 | -2.742672347 | 0.006099181 |
| SKMEL2:Paclitaxel:Ldose: -0.397940009 (uM) Interaction | -55.77948333 | 19.0858749 | -2.922553126 | 0.003475353 |
| UACC0257:Paclitaxel:Ldose: -0.397940009 (uM) Interaction | -45.67927545 | 19.0858749 | -2.39335507 | 0.016703582 |
| MeWo:Alisertib..MLN8237.:Ldose: -0.397940009 (uM) Interaction | -49.1189593 | 19.0858749 | -2.57357651 | 0.010071963 |
| SKMEL2:Alisertib..MLN8237.:Ldose: -0.397940009 (uM) Interaction | -47.45874249 | 19.0858749 | -2.486589834 | 0.012904916 |
| UACC0257:Alisertib..MLN8237.:Ldose: -0.397940009 (uM) Interaction | -45.04817558 | 19.0858749 | -2.360288738 | 0.018269578 |
| MeWo:Vorinostat:Ldose: -0.397940009 (uM) Interaction | -75.93075851 | 19.0858749 | -3.978374527 | 6.96E-05 |
| SKMEL2:Vorinostat:Ldose: -0.397940009 (uM) Interaction | -71.65227473 | 19.0858749 | -3.754204359 | 0.00017435 |
| UACC0257:Vorinostat:Ldose: -0.397940009 (uM) Interaction | -59.53949568 | 19.0858749 | -3.119558103 | 0.001813615 |
| MeWo:Busulfan:Ldose: -0.397940009 (uM) Interaction | -64.83190308 | 19.0858749 | -3.396852564 | 0.000682889 |
| SKMEL2:Busulfan:Ldose: -0.397940009 (uM) Interaction | -61.21287399 | 19.0858749 | -3.207234372 | 0.001342136 |
| UACC0257:Busulfan:Ldose: -0.397940009 (uM) Interaction | -48.62436527 | 19.0858749 | -2.54766237 | 0.010851623 |
| MeWo:Mechlorethamine.HCl:Ldose: -0.397940009 (uM) Interaction | -77.74082383 | 19.0858749 | -4.073212481 | 4.65E-05 |
| SKMEL2:Mechlorethamine.HCl:Ldose: -0.397940009 (uM) Interaction | -74.46991522 | 19.0858749 | -3.901833981 | 9.58E-05 |
| UACC0257:Mechlorethamine.HCl:Ldose: -0.397940009 (uM) Interaction | -73.76338891 | 19.0858749 | -3.864815698 | 0.000111499 |
| MeWo:Teniposide:Ldose: -0.397940009 (uM) Interaction | -49.03367621 | 19.0858749 | -2.569108122 | 0.010202733 |
| SKMEL2:Teniposide:Ldose: -0.397940009 (uM) Interaction | -31.59757678 | 19.0858749 | -1.655547726 | 0.097828011 |
| UACC0257:Teniposide:Ldose: -0.397940009 (uM) Interaction | -44.62733476 | 19.0858749 | -2.338238881 | 0.01938396 |
| MeWo:Vinorelbine.Tartrate:Ldose: -0.397940009 (uM) Interaction | -99.79529307 | 19.0858749 | -5.228751294 | 1.72E-07 |
| SKMEL2:Vinorelbine.Tartrate:Ldose: -0.397940009 (uM) Interaction | -86.95298307 | 19.0858749 | -4.555881433 | 5.25E-06 |
| UACC0257:Vinorelbine.Tartrate:Ldose: -0.397940009 (uM) Interaction | -91.2712112 | 19.0858749 | -4.782133997 | 1.75E-06 |
| MeWo:Cabozantinib..XL.184.:Ldose: -0.397940009 (uM) Interaction | -75.84823762 | 19.0858749 | -3.974050864 | 7.09E-05 |
| SKMEL2:Cabozantinib..XL.184.:Ldose: -0.397940009 (uM) Interaction | -56.17905779 | 19.0858749 | -2.943488737 | 0.003248819 |
| UACC0257:Cabozantinib..XL.184.:Ldose: -0.397940009 (uM) Interaction | -77.31226279 | 19.0858749 | -4.050758124 | 5.12E-05 |
| MeWo:Dacarbazine:Ldose: -0.397940009 (uM) Interaction | -73.39302531 | 19.0858749 | -3.845410583 | 0.000120697 |
| SKMEL2:Dacarbazine:Ldose: -0.397940009 (uM) Interaction | -75.95086687 | 19.0858749 | -3.9794281 | 6.93E-05 |
| UACC0257:Dacarbazine:Ldose: -0.397940009 (uM) Interaction | -70.32646494 | 19.0858749 | -3.684738862 | 0.000229498 |
| MeWo:Clofarabine:Ldose: -0.397940009 (uM) Interaction | -21.95693555 | 19.0858749 | -1.150428559 | 0.249980174 |
| SKMEL2:Clofarabine:Ldose: -0.397940009 (uM) Interaction | -33.63553507 | 19.0858749 | -1.762326079 | 0.078028401 |
| UACC0257:Clofarabine:Ldose: -0.397940009 (uM) Interaction | -46.4035476 | 19.0858749 | -2.431303142 | 0.015052721 |
| MeWo:Cisplatin:Ldose: -0.397940009 (uM) Interaction | -64.88663928 | 19.0858749 | -3.399720455 | 0.000675771 |
| SKMEL2:Cisplatin:Ldose: -0.397940009 (uM) Interaction | -62.61498495 | 19.0858749 | -3.280697652 | 0.001037152 |
| UACC0257:Cisplatin:Ldose: -0.397940009 (uM) Interaction | -76.56340507 | 19.0858749 | -4.011521897 | 6.05E-05 |
| MeWo:Floxuridine:Ldose: -0.397940009 (uM) Interaction | -45.08841821 | 19.0858749 | -2.362397242 | 0.018166014 |
| SKMEL2:Floxuridine:Ldose: -0.397940009 (uM) Interaction | -12.64382341 | 19.0858749 | -0.662470203 | 0.507677013 |
| UACC0257:Floxuridine:Ldose: -0.397940009 (uM) Interaction | -50.4623713 | 19.0858749 | -2.643964271 | 0.008200067 |
| MeWo:Lomustine..CCNU.:Ldose: -0.397940009 (uM) Interaction | -71.16090843 | 19.0858749 | -3.728459336 | 0.000193147 |
| SKMEL2:Lomustine..CCNU.:Ldose: -0.397940009 (uM) Interaction | -65.3361152 | 19.0858749 | -3.423270642 | 0.000619875 |
| UACC0257:Lomustine..CCNU.:Ldose: -0.397940009 (uM) Interaction | -70.22469882 | 19.0858749 | -3.67940685 | 0.000234347 |
| MeWo:Melphalan:Ldose: -0.397940009 (uM) Interaction | -64.97681808 | 19.0858749 | -3.404445352 | 0.000664194 |
| SKMEL2:Melphalan:Ldose: -0.397940009 (uM) Interaction | -61.14577033 | 19.0858749 | -3.203718491 | 0.001358625 |
| UACC0257:Melphalan:Ldose: -0.397940009 (uM) Interaction | -68.63711911 | 19.0858749 | -3.596225978 | 0.000323589 |
| MeWo:BGJ398..NVPBGJ398.:Ldose: -0.397940009 (uM) Interaction | -63.4523341 | 19.0858749 | -3.324570366 | 0.000887027 |
| SKMEL2:BGJ398..NVPBGJ398.:Ldose: -0.397940009 (uM) Interaction | -62.72957639 | 19.0858749 | -3.286701644 | 0.001015303 |
| UACC0257:BGJ398..NVPBGJ398.:Ldose: -0.397940009 (uM) Interaction | -77.14486894 | 19.0858749 | -4.041987562 | 5.32E-05 |
| MeWo:Navitoclax..ABT.263..5uM:Ldose: -0.397940009 (uM) Interaction | -74.68657386 | 19.0858749 | -3.91318576 | 9.14E-05 |
| SKMEL2:Navitoclax..ABT.263..5uM:Ldose: -0.397940009 (uM) Interaction | -63.4778559 | 19.0858749 | -3.325907575 | 0.000882784 |
| UACC0257:Navitoclax..ABT.263..5uM:Ldose: -0.397940009 (uM) Interaction | -60.97982375 | 19.0858749 | -3.195023759 | 0.001400208 |
| MeWo:Azacitidine:Ldose: -0.397940009 (uM) Interaction | -69.92493909 | 19.0858749 | -3.663701008 | 0.000249193 |
| SKMEL2:Azacitidine:Ldose: -0.397940009 (uM) Interaction | -78.01902778 | 19.0858749 | -4.087788912 | 4.37E-05 |
| UACC0257:Azacitidine:Ldose: -0.397940009 (uM) Interaction | -71.24750248 | 19.0858749 | -3.73299641 | 0.000189702 |
| MeWo:Capecitabine:Ldose: -0.397940009 (uM) Interaction | -70.53401248 | 19.0858749 | -3.695613267 | 0.0002199 |
| SKMEL2:Capecitabine:Ldose: -0.397940009 (uM) Interaction | -67.0780913 | 19.0858749 | -3.514541076 | 0.000441418 |
| UACC0257:Capecitabine:Ldose: -0.397940009 (uM) Interaction | -68.33409019 | 19.0858749 | -3.580348847 | 0.00034389 |
| MeWo:Megestrol.acetate:Ldose: -0.397940009 (uM) Interaction | -67.22207025 | 19.0858749 | -3.52208482 | 0.000429053 |
| SKMEL2:Megestrol.acetate:Ldose: -0.397940009 (uM) Interaction | -62.87393466 | 19.0858749 | -3.294265262 | 0.000988385 |
| UACC0257:Megestrol.acetate:Ldose: -0.397940009 (uM) Interaction | -85.55594731 | 19.0858749 | -4.482684067 | 7.41E-06 |
| MeWo:Cytarabine.HCl...Ara.C:Ldose: -0.397940009 (uM) Interaction | -17.98415488 | 19.0858749 | -0.942275635 | 0.346062107 |
| SKMEL2:Cytarabine.HCl...Ara.C:Ldose: -0.397940009 (uM) Interaction | -2.441368799 | 19.0858749 | -0.127914953 | 0.898217471 |
| UACC0257:Cytarabine.HCl...Ara.C:Ldose: -0.397940009 (uM) Interaction | -26.92643912 | 19.0858749 | -1.410804549 | 0.158316695 |
| MeWo:Gemcitabine.HCl:Ldose: -0.397940009 (uM) Interaction | -39.51924563 | 19.0858749 | -2.070601733 | 0.038407915 |
| SKMEL2:Gemcitabine.HCl:Ldose: -0.397940009 (uM) Interaction | 6.880353864 | 19.0858749 | 0.360494549 | 0.718480867 |
| UACC0257:Gemcitabine.HCl:Ldose: -0.397940009 (uM) Interaction | -87.30444657 | 19.0858749 | -4.574296282 | 4.80E-06 |
| MeWo:Vinblastine.Sulfate:Ldose: -0.397940009 (uM) Interaction | -67.85152162 | 19.0858749 | -3.555064779 | 0.000378695 |
| SKMEL2:Vinblastine.Sulfate:Ldose: -0.397940009 (uM) Interaction | -55.75397507 | 19.0858749 | -2.921216626 | 0.003490292 |
| UACC0257:Vinblastine.Sulfate:Ldose: -0.397940009 (uM) Interaction | -39.77350289 | 19.0858749 | -2.083923483 | 0.037178884 |
| MeWo:MLN9708..MLN2238.:Ldose: -0.397940009 (uM) Interaction | -71.67833438 | 19.0858749 | -3.755569748 | 0.000173403 |
| SKMEL2:MLN9708..MLN2238.:Ldose: -0.397940009 (uM) Interaction | -75.63009363 | 19.0858749 | -3.96262126 | 7.44E-05 |
| UACC0257:MLN9708..MLN2238.:Ldose: -0.397940009 (uM) Interaction | -70.76760773 | 19.0858749 | -3.707852436 | 0.000209549 |
| MeWo:ABT.737:Ldose: -0.397940009 (uM) Interaction | -86.32378252 | 19.0858749 | -4.522914615 | 6.13E-06 |
| SKMEL2:ABT.737:Ldose: -0.397940009 (uM) Interaction | -75.18031327 | 19.0858749 | -3.939055122 | 8.21E-05 |
| UACC0257:ABT.737:Ldose: -0.397940009 (uM) Interaction | -85.27105692 | 19.0858749 | -4.467757301 | 7.94E-06 |
| MeWo:Streptozocin:Ldose: -0.397940009 (uM) Interaction | -67.94689458 | 19.0858749 | -3.560061823 | 0.000371566 |
| SKMEL2:Streptozocin:Ldose: -0.397940009 (uM) Interaction | -74.90350995 | 19.0858749 | -3.924552076 | 8.72E-05 |
| UACC0257:Streptozocin:Ldose: -0.397940009 (uM) Interaction | -62.73132542 | 19.0858749 | -3.286793284 | 0.001014973 |
| MeWo:Crizotinib:Ldose: -0.397940009 (uM) Interaction | -73.23358478 | 19.0858749 | -3.837056733 | 0.000124873 |
| SKMEL2:Crizotinib:Ldose: -0.397940009 (uM) Interaction | -64.13570876 | 19.0858749 | -3.360375624 | 0.000779717 |
| UACC0257:Crizotinib:Ldose: -0.397940009 (uM) Interaction | -81.70794459 | 19.0858749 | -4.281068855 | 1.87E-05 |
| MeWo:Sunitinib:Ldose: -0.397940009 (uM) Interaction | -81.71080307 | 19.0858749 | -4.281218624 | 1.87E-05 |
| SKMEL2:Sunitinib:Ldose: -0.397940009 (uM) Interaction | -77.50978023 | 19.0858749 | -4.061107004 | 4.90E-05 |
| UACC0257:Sunitinib:Ldose: -0.397940009 (uM) Interaction | -58.61658174 | 19.0858749 | -3.07120224 | 0.002134643 |
| MeWo:Dexrazoxane:Ldose: -0.397940009 (uM) Interaction | -76.66210614 | 19.0858749 | -4.016693317 | 5.92E-05 |
| SKMEL2:Dexrazoxane:Ldose: -0.397940009 (uM) Interaction | -78.18656123 | 19.0858749 | -4.096566789 | 4.21E-05 |
| UACC0257:Dexrazoxane:Ldose: -0.397940009 (uM) Interaction | -80.04085168 | 19.0858749 | -4.193721908 | 2.76E-05 |
| MeWo:Mitomycin.C:Ldose: -0.397940009 (uM) Interaction | -73.79601773 | 19.0858749 | -3.866525277 | 0.000110721 |
| SKMEL2:Mitomycin.C:Ldose: -0.397940009 (uM) Interaction | -73.64272377 | 19.0858749 | -3.858493476 | 0.000114421 |
| UACC0257:Mitomycin.C:Ldose: -0.397940009 (uM) Interaction | -88.73131373 | 19.0858749 | -4.649056657 | 3.35E-06 |
| MeWo:Carfilzomib:Ldose: -0.397940009 (uM) Interaction | -104.4083847 | 19.0858749 | -5.470453162 | 4.54E-08 |
| SKMEL2:Carfilzomib:Ldose: -0.397940009 (uM) Interaction | -30.21235602 | 19.0858749 | -1.582969405 | 0.113443119 |
| UACC0257:Carfilzomib:Ldose: -0.397940009 (uM) Interaction | -111.4177872 | 19.0858749 | -5.837709187 | 5.37E-09 |
| MeWo:OSI.027:Ldose: -0.397940009 (uM) Interaction | -91.18029657 | 19.0858749 | -4.777370546 | 1.79E-06 |
| SKMEL2:OSI.027:Ldose: -0.397940009 (uM) Interaction | -80.84845498 | 19.0858749 | -4.236036096 | 2.28E-05 |
| UACC0257:OSI.027:Ldose: -0.397940009 (uM) Interaction | -88.11554414 | 19.0858749 | -4.616793551 | 3.92E-06 |
| MeWo:Bioymifi:Ldose: -0.397940009 (uM) Interaction | -74.85628508 | 19.0858749 | -3.92207774 | 8.81E-05 |
| SKMEL2:Bioymifi:Ldose: -0.397940009 (uM) Interaction | -72.24300674 | 19.0858749 | -3.785155626 | 0.000154029 |
| UACC0257:Bioymifi:Ldose: -0.397940009 (uM) Interaction | -83.73834202 | 19.0858749 | -4.387451059 | 1.15E-05 |
| MeWo:Nelarabine:Ldose: -0.397940009 (uM) Interaction | -74.06432951 | 19.0858749 | -3.880583411 | 0.000104516 |
| SKMEL2:Nelarabine:Ldose: -0.397940009 (uM) Interaction | -75.95988204 | 19.0858749 | -3.979900447 | 6.92E-05 |
| UACC0257:Nelarabine:Ldose: -0.397940009 (uM) Interaction | -71.738187 | 19.0858749 | -3.758705712 | 0.000171245 |
| MeWo:Raloxifene:Ldose: -0.397940009 (uM) Interaction | -64.7390725 | 19.0858749 | -3.391988727 | 0.00069512 |
| SKMEL2:Raloxifene:Ldose: -0.397940009 (uM) Interaction | -64.23056953 | 19.0858749 | -3.365345832 | 0.000765812 |
| UACC0257:Raloxifene:Ldose: -0.397940009 (uM) Interaction | -79.81774917 | 19.0858749 | -4.182032503 | 2.90E-05 |
| MeWo:Quinacrine.HCl:Ldose: -0.397940009 (uM) Interaction | -78.81183774 | 19.0858749 | -4.129328006 | 3.65E-05 |
| SKMEL2:Quinacrine.HCl:Ldose: -0.397940009 (uM) Interaction | -74.80819245 | 19.0858749 | -3.919557938 | 8.90E-05 |
| UACC0257:Quinacrine.HCl:Ldose: -0.397940009 (uM) Interaction | -71.64141903 | 19.0858749 | -3.753635577 | 0.000174746 |
| MeWo:Lenalidomide:Ldose: -0.397940009 (uM) Interaction | -76.87325179 | 19.0858749 | -4.027756244 | 5.65E-05 |
| SKMEL2:Lenalidomide:Ldose: -0.397940009 (uM) Interaction | -74.99494052 | 19.0858749 | -3.92934256 | 8.54E-05 |
| UACC0257:Lenalidomide:Ldose: -0.397940009 (uM) Interaction | -73.82248796 | 19.0858749 | -3.867912179 | 0.000110094 |
| MeWo:Fludarabine.Phosphate:Ldose: -0.397940009 (uM) Interaction | -79.53532982 | 19.0858749 | -4.167235207 | 3.10E-05 |
| SKMEL2:Fludarabine.Phosphate:Ldose: -0.397940009 (uM) Interaction | -74.38857457 | 19.0858749 | -3.897572156 | 9.75E-05 |
| UACC0257:Fludarabine.Phosphate:Ldose: -0.397940009 (uM) Interaction | -69.94765138 | 19.0858749 | -3.664891013 | 0.000248038 |
| MeWo:Nilotinib:Ldose: -0.397940009 (uM) Interaction | -75.51524139 | 19.0858749 | -3.956603603 | 7.63E-05 |
| SKMEL2:Nilotinib:Ldose: -0.397940009 (uM) Interaction | -72.75558317 | 19.0858749 | -3.812011949 | 0.000138224 |
| UACC0257:Nilotinib:Ldose: -0.397940009 (uM) Interaction | -78.71977161 | 19.0858749 | -4.124504223 | 3.73E-05 |
| MeWo:Linsitinib:Ldose: -0.397940009 (uM) Interaction | -70.38287914 | 19.0858749 | -3.687694671 | 0.000226851 |
| SKMEL2:Linsitinib:Ldose: -0.397940009 (uM) Interaction | -59.44841095 | 19.0858749 | -3.114785739 | 0.001843201 |
| UACC0257:Linsitinib:Ldose: -0.397940009 (uM) Interaction | -87.06647957 | 19.0858749 | -4.561828056 | 5.10E-06 |
| MeWo:Aphrocallistin.analogue:Ldose: -0.397940009 (uM) Interaction | -65.16468846 | 19.0858749 | -3.414288777 | 0.000640666 |
| SKMEL2:Aphrocallistin.analogue:Ldose: -0.397940009 (uM) Interaction | -25.95806218 | 19.0858749 | -1.360066663 | 0.173823063 |
| UACC0257:Aphrocallistin.analogue:Ldose: -0.397940009 (uM) Interaction | -27.6730707 | 19.0858749 | -1.449924138 | 0.147094235 |
| MeWo:Mitotane..o.p..DDD..Lysodren.:Ldose: -0.397940009 (uM) Interaction | -63.75882516 | 19.0858749 | -3.340628895 | 0.000837309 |
| SKMEL2:Mitotane..o.p..DDD..Lysodren.:Ldose: -0.397940009 (uM) Interaction | -75.27443946 | 19.0858749 | -3.943986842 | 8.04E-05 |
| UACC0257:Mitotane..o.p..DDD..Lysodren.:Ldose: -0.397940009 (uM) Interaction | -60.77099286 | 19.0858749 | -3.184082113 | 0.001454204 |
| MeWo:Etoposide:Ldose: -0.397940009 (uM) Interaction | -54.21315145 | 19.0858749 | -2.840485529 | 0.004508715 |
| SKMEL2:Etoposide:Ldose: -0.397940009 (uM) Interaction | -52.85870525 | 19.0858749 | -2.769519634 | 0.005618706 |
| UACC0257:Etoposide:Ldose: -0.397940009 (uM) Interaction | -56.21509849 | 19.0858749 | -2.945377081 | 0.003229062 |
| MeWo:Vandetanib:Ldose: -0.397940009 (uM) Interaction | -85.62795724 | 19.0858749 | -4.486457011 | 7.28E-06 |
| SKMEL2:Vandetanib:Ldose: -0.397940009 (uM) Interaction | -75.85908805 | 19.0858749 | -3.97461937 | 7.07E-05 |
| UACC0257:Vandetanib:Ldose: -0.397940009 (uM) Interaction | -80.04774081 | 19.0858749 | -4.194082862 | 2.75E-05 |
| MeWo:Carboplatin:Ldose: -0.397940009 (uM) Interaction | -75.40279672 | 19.0858749 | -3.950712091 | 7.82E-05 |
| SKMEL2:Carboplatin:Ldose: -0.397940009 (uM) Interaction | -77.04388273 | 19.0858749 | -4.036696413 | 5.44E-05 |
| UACC0257:Carboplatin:Ldose: -0.397940009 (uM) Interaction | -74.78038834 | 19.0858749 | -3.918101148 | 8.95E-05 |
| MeWo:Gefitinib:Ldose: -0.397940009 (uM) Interaction | -71.51007758 | 19.0858749 | -3.746753972 | 0.000179605 |
| SKMEL2:Gefitinib:Ldose: -0.397940009 (uM) Interaction | -74.26087279 | 19.0858749 | -3.890881251 | 0.000100181 |
| UACC0257:Gefitinib:Ldose: -0.397940009 (uM) Interaction | -62.6276262 | 19.0858749 | -3.281359987 | 0.00103472 |
| MeWo:Vincristine.Sulfate:Ldose: -0.397940009 (uM) Interaction | -40.65120474 | 19.0858749 | -2.129910468 | 0.033190308 |
| SKMEL2:Vincristine.Sulfate:Ldose: -0.397940009 (uM) Interaction | -15.67983678 | 19.0858749 | -0.821541421 | 0.411347033 |
| UACC0257:Vincristine.Sulfate:Ldose: -0.397940009 (uM) Interaction | -6.93770028 | 19.0858749 | -0.363499201 | 0.716235577 |
| MeWo:Trametinib..GSK1120212.:Ldose: -0.397940009 (uM) Interaction | -80.6467216 | 19.0858749 | -4.225466322 | 2.39E-05 |
| SKMEL2:Trametinib..GSK1120212.:Ldose: -0.397940009 (uM) Interaction | -66.70369509 | 19.0858749 | -3.494924673 | 0.000475148 |
| UACC0257:Trametinib..GSK1120212.:Ldose: -0.397940009 (uM) Interaction | -62.62943523 | 19.0858749 | -3.281454771 | 0.001034373 |
| MeWo:MLN4924:Ldose: -0.397940009 (uM) Interaction | -64.68817066 | 19.0858749 | -3.389321737 | 0.000701913 |
| SKMEL2:MLN4924:Ldose: -0.397940009 (uM) Interaction | -29.92663368 | 19.0858749 | -1.567999049 | 0.116896044 |
| UACC0257:MLN4924:Ldose: -0.397940009 (uM) Interaction | -33.39990804 | 19.0858749 | -1.749980455 | 0.080135924 |
| MeWo:Bortezomib:Ldose: -0.397940009 (uM) Interaction | -61.82546343 | 19.0858749 | -3.239330854 | 0.001199921 |
| SKMEL2:Bortezomib:Ldose: -0.397940009 (uM) Interaction | -47.90498553 | 19.0858749 | -2.509970635 | 0.012081392 |
| UACC0257:Bortezomib:Ldose: -0.397940009 (uM) Interaction | -52.90740476 | 19.0858749 | -2.772071233 | 0.005574867 |
| MeWo:Fluorouracil...5.FU.:Ldose: -0.397940009 (uM) Interaction | -77.59163505 | 19.0858749 | -4.065395769 | 4.81E-05 |
| SKMEL2:Fluorouracil...5.FU.:Ldose: -0.397940009 (uM) Interaction | -77.33204678 | 19.0858749 | -4.051794702 | 5.10E-05 |
| UACC0257:Fluorouracil...5.FU.:Ldose: -0.397940009 (uM) Interaction | -74.39594801 | 19.0858749 | -3.897958486 | 9.73E-05 |
| MeWo:Lapatinib:Ldose: -0.397940009 (uM) Interaction | -81.70343754 | 19.0858749 | -4.280832709 | 1.87E-05 |
| SKMEL2:Lapatinib:Ldose: -0.397940009 (uM) Interaction | -76.67201914 | 19.0858749 | -4.017212706 | 5.91E-05 |
| UACC0257:Lapatinib:Ldose: -0.397940009 (uM) Interaction | -69.15333942 | 19.0858749 | -3.623273221 | 0.000291567 |
| MeWo:Mitoxantrone:Ldose: -0.397940009 (uM) Interaction | -27.22509436 | 19.0858749 | -1.426452521 | 0.153752256 |
| SKMEL2:Mitoxantrone:Ldose: -0.397940009 (uM) Interaction | -2.143939191 | 19.0858749 | -0.112331198 | 0.910561853 |
| UACC0257:Mitoxantrone:Ldose: -0.397940009 (uM) Interaction | -29.41137792 | 19.0858749 | -1.541002342 | 0.123330906 |
| MeWo:Imatinib:Ldose: -0.397940009 (uM) Interaction | -76.9743256 | 19.0858749 | -4.033051983 | 5.52E-05 |
| SKMEL2:Imatinib:Ldose: -0.397940009 (uM) Interaction | -73.79342805 | 19.0858749 | -3.866389592 | 0.000110783 |
| UACC0257:Imatinib:Ldose: -0.397940009 (uM) Interaction | -66.77013577 | 19.0858749 | -3.498405818 | 0.000468992 |
| MeWo:Imiquimod:Ldose: -0.397940009 (uM) Interaction | -77.9274219 | 19.0858749 | -4.082989244 | 4.46E-05 |
| SKMEL2:Imiquimod:Ldose: -0.397940009 (uM) Interaction | -68.13891505 | 19.0858749 | -3.570122691 | 0.00035759 |
| UACC0257:Imiquimod:Ldose: -0.397940009 (uM) Interaction | -64.89299151 | 19.0858749 | -3.400053278 | 0.000674949 |
| MeWo:Dacomitinib..PF299804.:Ldose: -0.397940009 (uM) Interaction | -74.7679959 | 19.0858749 | -3.917451849 | 8.98E-05 |
| SKMEL2:Dacomitinib..PF299804.:Ldose: -0.397940009 (uM) Interaction | -70.69640245 | 19.0858749 | -3.704121652 | 0.000212654 |
| UACC0257:Dacomitinib..PF299804.:Ldose: -0.397940009 (uM) Interaction | -68.55049797 | 19.0858749 | -3.591687483 | 0.000329275 |
| MeWo:PD325901:Ldose: -0.397940009 (uM) Interaction | -92.20822776 | 19.0858749 | -4.831228763 | 1.37E-06 |
| SKMEL2:PD325901:Ldose: -0.397940009 (uM) Interaction | -91.6474145 | 19.0858749 | -4.801845082 | 1.58E-06 |
| UACC0257:PD325901:Ldose: -0.397940009 (uM) Interaction | -66.82215166 | 19.0858749 | -3.501131178 | 0.000464225 |
| MeWo:Vismodegib:Ldose: -0.397940009 (uM) Interaction | -66.23346755 | 19.0858749 | -3.47028721 | 0.00052092 |
| SKMEL2:Vismodegib:Ldose: -0.397940009 (uM) Interaction | -73.22572449 | 19.0858749 | -3.836644895 | 0.000125082 |
| UACC0257:Vismodegib:Ldose: -0.397940009 (uM) Interaction | -68.43077865 | 19.0858749 | -3.585414817 | 0.000337287 |
| MeWo:Temozolomide:Ldose: -0.397940009 (uM) Interaction | -73.22170681 | 19.0858749 | -3.83643439 | 0.000125189 |
| SKMEL2:Temozolomide:Ldose: -0.397940009 (uM) Interaction | -71.06256793 | 19.0858749 | -3.723306807 | 0.000197131 |
| UACC0257:Temozolomide:Ldose: -0.397940009 (uM) Interaction | -79.53777018 | 19.0858749 | -4.167363069 | 3.09E-05 |
| MeWo:Mercaptopurine:Ldose: -0.397940009 (uM) Interaction | -76.37230845 | 19.0858749 | -4.001509434 | 6.32E-05 |
| SKMEL2:Mercaptopurine:Ldose: -0.397940009 (uM) Interaction | -67.75704552 | 19.0858749 | -3.550114726 | 0.000385884 |
| UACC0257:Mercaptopurine:Ldose: -0.397940009 (uM) Interaction | -54.75753291 | 19.0858749 | -2.86900827 | 0.004121613 |
| MeWo:Dasatinib:Ldose: -0.397940009 (uM) Interaction | -80.46824856 | 19.0858749 | -4.216115268 | 2.50E-05 |
| SKMEL2:Dasatinib:Ldose: -0.397940009 (uM) Interaction | -79.97024408 | 19.0858749 | -4.190022439 | 2.80E-05 |
| UACC0257:Dasatinib:Ldose: -0.397940009 (uM) Interaction | -64.84923976 | 19.0858749 | -3.397760916 | 0.000680627 |
| MeWo:Daunorubicin.HCl:Ldose: -0.397940009 (uM) Interaction | -44.24773895 | 19.0858749 | -2.318350046 | 0.02043962 |
| SKMEL2:Daunorubicin.HCl:Ldose: -0.397940009 (uM) Interaction | -16.46186623 | 19.0858749 | -0.862515673 | 0.388413415 |
| UACC0257:Daunorubicin.HCl:Ldose: -0.397940009 (uM) Interaction | -40.85050198 | 19.0858749 | -2.140352601 | 0.032337479 |
| MeWo:Sirolimus..Rapamycin.:Ldose: -0.397940009 (uM) Interaction | -76.95018536 | 19.0858749 | -4.031787161 | 5.55E-05 |
| SKMEL2:Sirolimus..Rapamycin.:Ldose: -0.397940009 (uM) Interaction | -70.67486067 | 19.0858749 | -3.702992975 | 0.000213602 |
| UACC0257:Sirolimus..Rapamycin.:Ldose: -0.397940009 (uM) Interaction | -82.78366841 | 19.0858749 | -4.337431156 | 1.45E-05 |
| MeWo:INK.128..MLN0128.:Ldose: -0.397940009 (uM) Interaction | -43.0435265 | 19.0858749 | -2.255255614 | 0.024127283 |
| SKMEL2:INK.128..MLN0128.:Ldose: -0.397940009 (uM) Interaction | -56.35632183 | 19.0858749 | -2.952776445 | 0.003152698 |
| UACC0257:INK.128..MLN0128.:Ldose: -0.397940009 (uM) Interaction | -57.78662075 | 19.0858749 | -3.027716626 | 0.002467004 |
| MeWo:Quizartinib:Ldose: -0.397940009 (uM) Interaction | -69.36749377 | 19.0858749 | -3.634493789 | 0.000279175 |
| SKMEL2:Quizartinib:Ldose: -0.397940009 (uM) Interaction | -63.67557149 | 19.0858749 | -3.336266838 | 0.000850552 |
| UACC0257:Quizartinib:Ldose: -0.397940009 (uM) Interaction | -82.31856232 | 19.0858749 | -4.31306203 | 1.62E-05 |
| MeWo:Sorafenib:Ldose: -0.397940009 (uM) Interaction | -64.32841594 | 19.0858749 | -3.370472472 | 0.000751712 |
| SKMEL2:Sorafenib:Ldose: -0.397940009 (uM) Interaction | -70.10129957 | 19.0858749 | -3.672941374 | 0.000240355 |
| UACC0257:Sorafenib:Ldose: -0.397940009 (uM) Interaction | -67.60059862 | 19.0858749 | -3.541917727 | 0.000398068 |
| MeWo:Carmustine:Ldose: -0.397940009 (uM) Interaction | -70.32397612 | 19.0858749 | -3.68460846 | 0.000229615 |
| SKMEL2:Carmustine:Ldose: -0.397940009 (uM) Interaction | -62.29710534 | 19.0858749 | -3.264042423 | 0.001100059 |
| UACC0257:Carmustine:Ldose: -0.397940009 (uM) Interaction | -74.92446462 | 19.0858749 | -3.925649991 | 8.68E-05 |
| MeWo:Uracil.mustard:Ldose: -0.397940009 (uM) Interaction | -63.28954442 | 19.0858749 | -3.316041039 | 0.000914533 |
| SKMEL2:Uracil.mustard:Ldose: -0.397940009 (uM) Interaction | -61.75727528 | 19.0858749 | -3.235758152 | 0.001215033 |
| UACC0257:Uracil.mustard:Ldose: -0.397940009 (uM) Interaction | -76.47699719 | 19.0858749 | -4.006994576 | 6.17E-05 |
| MeWo:Ixabepilone:Ldose: -0.397940009 (uM) Interaction | -52.22944284 | 19.0858749 | -2.736549575 | 0.006213813 |
| SKMEL2:Ixabepilone:Ldose: -0.397940009 (uM) Interaction | -42.29103051 | 19.0858749 | -2.21582876 | 0.026713646 |
| UACC0257:Ixabepilone:Ldose: -0.397940009 (uM) Interaction | -59.7916268 | 19.0858749 | -3.132768455 | 0.00173398 |
| MeWo:Valrubicin:Ldose: -0.397940009 (uM) Interaction | -45.75069077 | 19.0858749 | -2.397096859 | 0.01653402 |
| SKMEL2:Valrubicin:Ldose: -0.397940009 (uM) Interaction | -60.25943557 | 19.0858749 | -3.157279187 | 0.001594684 |
| UACC0257:Valrubicin:Ldose: -0.397940009 (uM) Interaction | -49.00752112 | 19.0858749 | -2.567737732 | 0.01024314 |
| MeWo:Triethylenemelamine:Ldose: -0.397940009 (uM) Interaction | -74.04146742 | 19.0858749 | -3.879385557 | 0.000105032 |
| SKMEL2:Triethylenemelamine:Ldose: -0.397940009 (uM) Interaction | -68.00567654 | 19.0858749 | -3.563141691 | 0.000367234 |
| UACC0257:Triethylenemelamine:Ldose: -0.397940009 (uM) Interaction | -73.46149977 | 19.0858749 | -3.848998287 | 0.000118944 |
| MeWo:Palbociclib..PD.0332991..Isethionate:Ldose: -0.397940009 (uM) Interaction | -60.98714687 | 19.0858749 | -3.195407452 | 0.001398348 |
| SKMEL2:Palbociclib..PD.0332991..Isethionate:Ldose: -0.397940009 (uM) Interaction | -52.82402532 | 19.0858749 | -2.767702587 | 0.005650114 |
| UACC0257:Palbociclib..PD.0332991..Isethionate:Ldose: -0.397940009 (uM) Interaction | -66.09863204 | 19.0858749 | -3.463222535 | 0.000534784 |
| MeWo:Afatinib:Ldose: -0.397940009 (uM) Interaction | -64.48798892 | 19.0858749 | -3.378833261 | 0.000729231 |
| SKMEL2:Afatinib:Ldose: -0.397940009 (uM) Interaction | -60.24853921 | 19.0858749 | -3.156708274 | 0.001597808 |
| UACC0257:Afatinib:Ldose: -0.397940009 (uM) Interaction | -65.99880349 | 19.0858749 | -3.457992041 | 0.000545269 |
| MeWo:Doxorubicin.HCl:Ldose: -0.397940009 (uM) Interaction | -32.09284922 | 19.0858749 | -1.681497411 | 0.092680836 |
| SKMEL2:Doxorubicin.HCl:Ldose: -0.397940009 (uM) Interaction | -36.81691903 | 19.0858749 | -1.929013956 | 0.053742252 |
| UACC0257:Doxorubicin.HCl:Ldose: -0.397940009 (uM) Interaction | -44.68223447 | 19.0858749 | -2.341115339 | 0.019235296 |
| MeWo:Exemestane:Ldose: -0.397940009 (uM) Interaction | -70.5327026 | 19.0858749 | -3.695544636 | 0.000219959 |
| SKMEL2:Exemestane:Ldose: -0.397940009 (uM) Interaction | -66.20671264 | 19.0858749 | -3.468885393 | 0.000523644 |
| UACC0257:Exemestane:Ldose: -0.397940009 (uM) Interaction | -73.87966345 | 19.0858749 | -3.870907876 | 0.000108751 |
| MeWo:Tretinoin:Ldose: -0.397940009 (uM) Interaction | -68.13390841 | 19.0858749 | -3.569860369 | 0.000357949 |
| SKMEL2:Tretinoin:Ldose: -0.397940009 (uM) Interaction | -62.99440477 | 19.0858749 | -3.300577265 | 0.000966429 |
| UACC0257:Tretinoin:Ldose: -0.397940009 (uM) Interaction | -67.87163668 | 19.0858749 | -3.556118703 | 0.000377181 |
| MeWo:Fulvestrant:Ldose: -0.397940009 (uM) Interaction | -66.70663972 | 19.0858749 | -3.495078957 | 0.000474874 |
| SKMEL2:Fulvestrant:Ldose: -0.397940009 (uM) Interaction | -70.94828589 | 19.0858749 | -3.717319026 | 0.000201858 |
| UACC0257:Fulvestrant:Ldose: -0.397940009 (uM) Interaction | -61.72571562 | 19.0858749 | -3.234104591 | 0.001222086 |
| MeWo:Docetaxel:Ldose: -0.397940009 (uM) Interaction | -55.42370597 | 19.0858749 | -2.903912253 | 0.003689057 |
| SKMEL2:Docetaxel:Ldose: -0.397940009 (uM) Interaction | -62.6181584 | 19.0858749 | -3.280863924 | 0.001036541 |
| UACC0257:Docetaxel:Ldose: -0.397940009 (uM) Interaction | -56.80269347 | 19.0858749 | -2.976163984 | 0.002922029 |
| MeWo:Everolimus:Ldose: -0.397940009 (uM) Interaction | -82.78279263 | 19.0858749 | -4.337385269 | 1.45E-05 |
| SKMEL2:Everolimus:Ldose: -0.397940009 (uM) Interaction | -69.02968803 | 19.0858749 | -3.616794535 | 0.000298955 |
| UACC0257:Everolimus:Ldose: -0.397940009 (uM) Interaction | -73.79706014 | 19.0858749 | -3.866579895 | 0.000110696 |
| MeWo:MLN.2480:Ldose: -0.397940009 (uM) Interaction | -64.10010784 | 19.0858749 | -3.358510322 | 0.000784996 |
| SKMEL2:MLN.2480:Ldose: -0.397940009 (uM) Interaction | -53.77171169 | 19.0858749 | -2.817356395 | 0.004846484 |
| UACC0257:MLN.2480:Ldose: -0.397940009 (uM) Interaction | -82.81703021 | 19.0858749 | -4.33917914 | 1.44E-05 |
| MeWo:LY2157299:Ldose: -0.397940009 (uM) Interaction | -67.91112298 | 19.0858749 | -3.558187579 | 0.000374225 |
| SKMEL2:LY2157299:Ldose: -0.397940009 (uM) Interaction | -63.44735094 | 19.0858749 | -3.324309275 | 0.000887857 |
| UACC0257:LY2157299:Ldose: -0.397940009 (uM) Interaction | -62.40253849 | 19.0858749 | -3.269566568 | 0.001078814 |
| MeWo:Allopurinol:Ldose: -0.397940009 (uM) Interaction | -75.5056251 | 19.0858749 | -3.95609976 | 7.64E-05 |
| SKMEL2:Allopurinol:Ldose: -0.397940009 (uM) Interaction | -71.9455089 | 19.0858749 | -3.769568295 | 0.000163967 |
| UACC0257:Allopurinol:Ldose: -0.397940009 (uM) Interaction | -66.7824453 | 19.0858749 | -3.499050772 | 0.00046786 |
| MeWo:Pipobroman:Ldose: -0.397940009 (uM) Interaction | -58.04270184 | 19.0858749 | -3.041133936 | 0.002359722 |
| SKMEL2:Pipobroman:Ldose: -0.397940009 (uM) Interaction | -55.45945338 | 19.0858749 | -2.90578523 | 0.003667057 |
| UACC0257:Pipobroman:Ldose: -0.397940009 (uM) Interaction | -58.55839802 | 19.0858749 | -3.068153718 | 0.002156532 |
| MeWo:Letrozole:Ldose: -0.397940009 (uM) Interaction | -70.06363831 | 19.0858749 | -3.670968121 | 0.000242217 |
| SKMEL2:Letrozole:Ldose: -0.397940009 (uM) Interaction | -61.79935492 | 19.0858749 | -3.237962905 | 0.001205687 |
| UACC0257:Letrozole:Ldose: -0.397940009 (uM) Interaction | -72.49467205 | 19.0858749 | -3.798341572 | 0.000146067 |
| MeWo:Thiotepa:Ldose: -0.397940009 (uM) Interaction | -78.9621856 | 19.0858749 | -4.137205448 | 3.53E-05 |
| SKMEL2:Thiotepa:Ldose: -0.397940009 (uM) Interaction | -77.15103506 | 19.0858749 | -4.042310634 | 5.31E-05 |
| UACC0257:Thiotepa:Ldose: -0.397940009 (uM) Interaction | -68.6854436 | 19.0858749 | -3.598757928 | 0.000320457 |
| MeWo:Plicamycin:Ldose: -0.397940009 (uM) Interaction | -63.95725445 | 19.0858749 | -3.351025552 | 0.000806512 |
| SKMEL2:Plicamycin:Ldose: -0.397940009 (uM) Interaction | -45.16720382 | 19.0858749 | -2.366525195 | 0.017964747 |
| UACC0257:Plicamycin:Ldose: -0.397940009 (uM) Interaction | -59.31644162 | 19.0858749 | -3.107871237 | 0.001886854 |
| MeWo:Erlotinib.HCl:Ldose: -0.397940009 (uM) Interaction | -92.67201433 | 19.0858749 | -4.855528753 | 1.21E-06 |
| SKMEL2:Erlotinib.HCl:Ldose: -0.397940009 (uM) Interaction | -83.47238085 | 19.0858749 | -4.373516085 | 1.23E-05 |
| UACC0257:Erlotinib.HCl:Ldose: -0.397940009 (uM) Interaction | -82.25298685 | 19.0858749 | -4.309626218 | 1.64E-05 |
| MeWo:MEK.162..ARRY.438162.:Ldose: -0.397940009 (uM) Interaction | -42.90571731 | 19.0858749 | -2.248035133 | 0.024584021 |
| SKMEL2:MEK.162..ARRY.438162.:Ldose: -0.397940009 (uM) Interaction | -21.37918559 | 19.0858749 | -1.120157483 | 0.262659148 |
| UACC0257:MEK.162..ARRY.438162.:Ldose: -0.397940009 (uM) Interaction | -37.52206231 | 19.0858749 | -1.965959775 | 0.049316081 |
| MeWo:Baricitinib..LY3009104..INCB028050.:Ldose: -0.397940009 (uM) Interaction | -61.80308652 | 19.0858749 | -3.238158421 | 0.001204861 |
| SKMEL2:Baricitinib..LY3009104..INCB028050.:Ldose: -0.397940009 (uM) Interaction | -54.07657356 | 19.0858749 | -2.833329562 | 0.004610866 |
| UACC0257:Baricitinib..LY3009104..INCB028050.:Ldose: -0.397940009 (uM) Interaction | -75.09168811 | 19.0858749 | -3.934411627 | 8.37E-05 |
| MeWo:Arsenic.Trioxide:Ldose: -0.397940009 (uM) Interaction | -61.42746865 | 19.0858749 | -3.21847801 | 0.001290636 |
| SKMEL2:Arsenic.Trioxide:Ldose: -0.397940009 (uM) Interaction | -63.56738915 | 19.0858749 | -3.33059865 | 0.00086805 |
| UACC0257:Arsenic.Trioxide:Ldose: -0.397940009 (uM) Interaction | -59.68815549 | 19.0858749 | -3.127347099 | 0.001766264 |
| MeWo:Celecoxib:Ldose: -0.397940009 (uM) Interaction | -62.3504638 | 19.0858749 | -3.266838127 | 0.001089259 |
| SKMEL2:Celecoxib:Ldose: -0.397940009 (uM) Interaction | -60.39467163 | 19.0858749 | -3.164364849 | 0.001556379 |
| UACC0257:Celecoxib:Ldose: -0.397940009 (uM) Interaction | -72.26969737 | 19.0858749 | -3.786554075 | 0.000153165 |
| MeWo:Bendamustine.HCl:Ldose: -0.397940009 (uM) Interaction | -70.92355474 | 19.0858749 | -3.716023244 | 0.000202895 |
| SKMEL2:Bendamustine.HCl:Ldose: -0.397940009 (uM) Interaction | -58.26417068 | 19.0858749 | -3.052737744 | 0.002270404 |
| UACC0257:Bendamustine.HCl:Ldose: -0.397940009 (uM) Interaction | -75.7830633 | 19.0858749 | -3.97063607 | 7.19E-05 |
| MeWo:Chlorambucil:Ldose: -0.397940009 (uM) Interaction | -74.94272613 | 19.0858749 | -3.926606799 | 8.64E-05 |
| SKMEL2:Chlorambucil:Ldose: -0.397940009 (uM) Interaction | -67.34604909 | 19.0858749 | -3.528580663 | 0.000418665 |
| UACC0257:Chlorambucil:Ldose: -0.397940009 (uM) Interaction | -86.91047494 | 19.0858749 | -4.553654229 | 5.30E-06 |
| MeWo:Zoledronic.Acid:Ldose: -0.397940009 (uM) Interaction | -65.24051247 | 19.0858749 | -3.418261558 | 0.000631391 |
| SKMEL2:Zoledronic.Acid:Ldose: -0.397940009 (uM) Interaction | -73.33286628 | 19.0858749 | -3.842258564 | 0.000122257 |
| UACC0257:Zoledronic.Acid:Ldose: -0.397940009 (uM) Interaction | -67.56580592 | 19.0858749 | -3.540094771 | 0.000400827 |
| MeWo:Actinomycin.D:Ldose: -0.397940009 (uM) Interaction | -78.99023567 | 19.0858749 | -4.138675125 | 3.51E-05 |
| SKMEL2:Actinomycin.D:Ldose: -0.397940009 (uM) Interaction | -105.3109574 | 19.0858749 | -5.517743253 | 3.47E-08 |
| UACC0257:Actinomycin.D:Ldose: -0.397940009 (uM) Interaction | -99.26409695 | 19.0858749 | -5.200919397 | 2.00E-07 |
| MeWo:Temsirolimus..CCI.779..Torisel.:Ldose: -0.397940009 (uM) Interaction | -71.8954054 | 19.0858749 | -3.766943134 | 0.000165698 |
| SKMEL2:Temsirolimus..CCI.779..Torisel.:Ldose: -0.397940009 (uM) Interaction | -58.53625036 | 19.0858749 | -3.066993296 | 0.002164918 |
| UACC0257:Temsirolimus..CCI.779..Torisel.:Ldose: -0.397940009 (uM) Interaction | -62.7935163 | 19.0858749 | -3.290051761 | 0.001003298 |
| MeWo:Foretinib..GSK1363089.:Ldose: -0.397940009 (uM) Interaction | -81.06305395 | 19.0858749 | -4.247279959 | 2.17E-05 |
| SKMEL2:Foretinib..GSK1363089.:Ldose: -0.397940009 (uM) Interaction | -44.5108729 | 19.0858749 | -2.332136889 | 0.019702657 |
| UACC0257:Foretinib..GSK1363089.:Ldose: -0.397940009 (uM) Interaction | -71.91611059 | 19.0858749 | -3.768027978 | 0.000164981 |
| MeWo:Decitabine:Ldose: -0.397940009 (uM) Interaction | -73.28996575 | 19.0858749 | -3.840010801 | 0.000123381 |
| SKMEL2:Decitabine:Ldose: -0.397940009 (uM) Interaction | -65.74741595 | 19.0858749 | -3.444820649 | 0.000572527 |
| UACC0257:Decitabine:Ldose: -0.397940009 (uM) Interaction | -66.19371185 | 19.0858749 | -3.46820422 | 0.000524972 |
| MeWo:Methotrexate:Ldose: -0.397940009 (uM) Interaction | -65.50440307 | 19.0858749 | -3.432088045 | 0.000600078 |
| SKMEL2:Methotrexate:Ldose: -0.397940009 (uM) Interaction | -64.69292418 | 19.0858749 | -3.389570797 | 0.000701276 |
| UACC0257:Methotrexate:Ldose: -0.397940009 (uM) Interaction | -75.97546176 | 19.0858749 | -3.980716743 | 6.89E-05 |
| MeWo:Axitinib:Ldose: -0.397940009 (uM) Interaction | -64.11621499 | 19.0858749 | -3.359354252 | 0.000782603 |
| SKMEL2:Axitinib:Ldose: -0.397940009 (uM) Interaction | -66.81064709 | 19.0858749 | -3.500528399 | 0.000465275 |
| UACC0257:Axitinib:Ldose: -0.397940009 (uM) Interaction | -76.73318555 | 19.0858749 | -4.020417506 | 5.83E-05 |
| MeWo:Oxaliplatin:Ldose: -0.397940009 (uM) Interaction | -79.78455962 | 19.0858749 | -4.180293544 | 2.92E-05 |
| SKMEL2:Oxaliplatin:Ldose: -0.397940009 (uM) Interaction | -67.94500398 | 19.0858749 | -3.559962766 | 0.000371706 |
| UACC0257:Oxaliplatin:Ldose: -0.397940009 (uM) Interaction | -75.48060439 | 19.0858749 | -3.954788806 | 7.68E-05 |
| MeWo:Cabazitaxel:Ldose: -0.397940009 (uM) Interaction | -63.60886907 | 19.0858749 | -3.33277198 | 0.000861302 |
| SKMEL2:Cabazitaxel:Ldose: -0.397940009 (uM) Interaction | -59.19393552 | 19.0858749 | -3.101452558 | 0.001928224 |
| UACC0257:Cabazitaxel:Ldose: -0.397940009 (uM) Interaction | -70.33825267 | 19.0858749 | -3.685356477 | 0.000228943 |
| MeWo:Amifostine:Ldose: -0.397940009 (uM) Interaction | -73.3863498 | 19.0858749 | -3.845060821 | 0.000120869 |
| SKMEL2:Amifostine:Ldose: -0.397940009 (uM) Interaction | -67.34253834 | 19.0858749 | -3.528396718 | 0.000418956 |
| UACC0257:Amifostine:Ldose: -0.397940009 (uM) Interaction | -75.77949911 | 19.0858749 | -3.970449325 | 7.20E-05 |
| MeWo:Flutamide..Eulexin.:Ldose: -0.397940009 (uM) Interaction | -68.66496677 | 19.0858749 | -3.597685049 | 0.000321781 |
| SKMEL2:Flutamide..Eulexin.:Ldose: -0.397940009 (uM) Interaction | -63.39622118 | 19.0858749 | -3.321630343 | 0.00089642 |
| UACC0257:Flutamide..Eulexin.:Ldose: -0.397940009 (uM) Interaction | -81.31879109 | 19.0858749 | -4.260679247 | 2.05E-05 |
| MeWo:LDK378:Ldose: -0.397940009 (uM) Interaction | -59.03389263 | 19.0858749 | -3.093067148 | 0.001983526 |
| SKMEL2:LDK378:Ldose: -0.397940009 (uM) Interaction | -51.93016678 | 19.0858749 | -2.720869075 | 0.006516282 |
| UACC0257:LDK378:Ldose: -0.397940009 (uM) Interaction | -76.73874498 | 19.0858749 | -4.020708791 | 5.82E-05 |
| MeWo:Pralatrexate:Ldose: -0.397940009 (uM) Interaction | -63.76574207 | 19.0858749 | -3.340991305 | 0.000836217 |
| SKMEL2:Pralatrexate:Ldose: -0.397940009 (uM) Interaction | -60.81052607 | 19.0858749 | -3.186153446 | 0.001443837 |
| UACC0257:Pralatrexate:Ldose: -0.397940009 (uM) Interaction | -73.22997634 | 19.0858749 | -3.83686767 | 0.000124969 |
| MeWo:Topotecan.HCl:Ldose: -0.397940009 (uM) Interaction | -11.17300259 | 19.0858749 | -0.585406886 | 0.558280292 |
| SKMEL2:Topotecan.HCl:Ldose: -0.397940009 (uM) Interaction | -12.40819273 | 19.0858749 | -0.650124387 | 0.515618811 |
| UACC0257:Topotecan.HCl:Ldose: -0.397940009 (uM) Interaction | -42.2920264 | 19.0858749 | -2.21588094 | 0.02671007 |
| MeWo:Pemetrexed:Ldose: -0.397940009 (uM) Interaction | -68.09603267 | 19.0858749 | -3.567875879 | 0.000360668 |
| SKMEL2:Pemetrexed:Ldose: -0.397940009 (uM) Interaction | -60.57669412 | 19.0858749 | -3.173901875 | 0.00150616 |
| UACC0257:Pemetrexed:Ldose: -0.397940009 (uM) Interaction | -60.85925262 | 19.0858749 | -3.188706462 | 0.001431153 |
| MeWo:Bleomycin.Sulfate:Ldose: -0.397940009 (uM) Interaction | -47.31299423 | 19.0858749 | -2.478953387 | 0.013184448 |
| SKMEL2:Bleomycin.Sulfate:Ldose: -0.397940009 (uM) Interaction | -49.37724796 | 19.0858749 | -2.587109484 | 0.009684973 |
| UACC0257:Bleomycin.Sulfate:Ldose: -0.397940009 (uM) Interaction | -30.33342508 | 19.0858749 | -1.58931279 | 0.112004469 |
| MeWo:Axitinib.1:Ldose: -0.397940009 (uM) Interaction | -63.78453653 | 19.0858749 | -3.341976036 | 0.000833258 |
| SKMEL2:Axitinib.1:Ldose: -0.397940009 (uM) Interaction | -54.20371274 | 19.0858749 | -2.83999099 | 0.004515708 |
| UACC0257:Axitinib.1:Ldose: -0.397940009 (uM) Interaction | -73.59830664 | 19.0858749 | -3.856166251 | 0.000115514 |
| MeWo:Ibrutinib..PCI.32765.:Ldose: -0.397940009 (uM) Interaction | -69.1781755 | 19.0858749 | -3.624574502 | 0.000290104 |
| SKMEL2:Ibrutinib..PCI.32765.:Ldose: -0.397940009 (uM) Interaction | -61.75439927 | 19.0858749 | -3.235607464 | 0.001215674 |
| UACC0257:Ibrutinib..PCI.32765.:Ldose: -0.397940009 (uM) Interaction | -72.83949373 | 19.0858749 | -3.816408424 | 0.000135787 |
| MeWo:Tamoxifen.Citrate:Ldose: -0.397940009 (uM) Interaction | -67.92943332 | 19.0858749 | -3.559146944 | 0.000372862 |
| SKMEL2:Tamoxifen.Citrate:Ldose: -0.397940009 (uM) Interaction | -59.56366078 | 19.0858749 | -3.120824228 | 0.00180584 |
| UACC0257:Tamoxifen.Citrate:Ldose: -0.397940009 (uM) Interaction | -70.38426112 | 19.0858749 | -3.687767079 | 0.000226786 |
| MeWo:Vemurafenib:Ldose: -0.397940009 (uM) Interaction | -16.71029629 | 19.0858749 | -0.875532109 | 0.38129421 |
| SKMEL2:Vemurafenib:Ldose: -0.397940009 (uM) Interaction | -15.17709348 | 19.0858749 | -0.795200302 | 0.426505758 |
| UACC0257:Vemurafenib:Ldose: -0.397940009 (uM) Interaction | -40.07537443 | 19.0858749 | -2.099739972 | 0.035763324 |
| MeWo:Pazopanib.HCl:Ldose: -0.397940009 (uM) Interaction | -65.54335095 | 19.0858749 | -3.434128711 | 0.000595581 |
| SKMEL2:Pazopanib.HCl:Ldose: -0.397940009 (uM) Interaction | -62.96794895 | 19.0858749 | -3.299191119 | 0.000971211 |
| UACC0257:Pazopanib.HCl:Ldose: -0.397940009 (uM) Interaction | -57.19383421 | 19.0858749 | -2.996657713 | 0.002732679 |
| MeWo:Abiraterone:Ldose: -0.397940009 (uM) Interaction | -68.65028623 | 19.0858749 | -3.596915866 | 0.000322733 |
| SKMEL2:Abiraterone:Ldose: -0.397940009 (uM) Interaction | -66.69360782 | 19.0858749 | -3.494396153 | 0.00047609 |
| UACC0257:Abiraterone:Ldose: -0.397940009 (uM) Interaction | -71.89618169 | 19.0858749 | -3.766983808 | 0.000165671 |
| MeWo:Bosutinib..SKI.606.:Ldose: -0.397940009 (uM) Interaction | -48.29090087 | 19.0858749 | -2.53019058 | 0.011407122 |
| SKMEL2:Bosutinib..SKI.606.:Ldose: -0.397940009 (uM) Interaction | -42.52758312 | 19.0858749 | -2.22822288 | 0.025875938 |
| UACC0257:Bosutinib..SKI.606.:Ldose: -0.397940009 (uM) Interaction | -66.35443865 | 19.0858749 | -3.476625463 | 0.000508767 |
| MeWo:Sabutoclax..BI.97C1.:Ldose: -0.397940009 (uM) Interaction | -59.36147536 | 19.0858749 | -3.110230769 | 0.001871852 |
| SKMEL2:Sabutoclax..BI.97C1.:Ldose: -0.397940009 (uM) Interaction | -40.41386512 | 19.0858749 | -2.117475114 | 0.034230972 |
| UACC0257:Sabutoclax..BI.97C1.:Ldose: -0.397940009 (uM) Interaction | -37.76815243 | 19.0858749 | -1.97885361 | 0.047845206 |
| MeWo:Thioguanine:Ldose: 0 (uM) Interaction | -39.35984083 | 19.0858749 | -2.062249755 | 0.039195928 |
| SKMEL2:Thioguanine:Ldose: 0 (uM) Interaction | -31.8709066 | 19.0858749 | -1.669868779 | 0.094959859 |
| UACC0257:Thioguanine:Ldose: 0 (uM) Interaction | -7.281937847 | 19.0858749 | -0.381535449 | 0.702809736 |
| MeWo:Irinotecan.HCl:Ldose: 0 (uM) Interaction | -35.47803856 | 19.0858749 | -1.858863623 | 0.0630601 |
| SKMEL2:Irinotecan.HCl:Ldose: 0 (uM) Interaction | -37.49896284 | 19.0858749 | -1.964749483 | 0.049456074 |
| UACC0257:Irinotecan.HCl:Ldose: 0 (uM) Interaction | -17.82923242 | 19.0858749 | -0.934158508 | 0.350232607 |
| MeWo:Romidepsin:Ldose: 0 (uM) Interaction | -49.69610824 | 19.0858749 | -2.603816095 | 0.009225546 |
| SKMEL2:Romidepsin:Ldose: 0 (uM) Interaction | -48.9672599 | 19.0858749 | -2.565628255 | 0.010305618 |
| UACC0257:Romidepsin:Ldose: 0 (uM) Interaction | -40.64554597 | 19.0858749 | -2.129613978 | 0.033214801 |
| MeWo:Paclitaxel:Ldose: 0 (uM) Interaction | -33.19352663 | 19.0858749 | -1.739167149 | 0.082019652 |
| SKMEL2:Paclitaxel:Ldose: 0 (uM) Interaction | -38.84349133 | 19.0858749 | -2.035195742 | 0.041843398 |
| UACC0257:Paclitaxel:Ldose: 0 (uM) Interaction | -5.846139022 | 19.0858749 | -0.306307102 | 0.759373793 |
| MeWo:Alisertib..MLN8237.:Ldose: 0 (uM) Interaction | -29.56813115 | 19.0858749 | -1.549215391 | 0.121344642 |
| SKMEL2:Alisertib..MLN8237.:Ldose: 0 (uM) Interaction | -15.44184463 | 19.0858749 | -0.809071877 | 0.41848274 |
| UACC0257:Alisertib..MLN8237.:Ldose: 0 (uM) Interaction | -2.805627896 | 19.0858749 | -0.147000225 | 0.883133219 |
| MeWo:Vorinostat:Ldose: 0 (uM) Interaction | -72.28572214 | 19.0858749 | -3.78739369 | 0.000152649 |
| SKMEL2:Vorinostat:Ldose: 0 (uM) Interaction | -50.13648069 | 19.0858749 | -2.626889307 | 0.008623036 |
| UACC0257:Vorinostat:Ldose: 0 (uM) Interaction | -27.84669146 | 19.0858749 | -1.459020957 | 0.144573921 |
| MeWo:Busulfan:Ldose: 0 (uM) Interaction | -49.80237893 | 19.0858749 | -2.609384123 | 0.009076806 |
| SKMEL2:Busulfan:Ldose: 0 (uM) Interaction | -42.18544455 | 19.0858749 | -2.210296608 | 0.027095056 |
| UACC0257:Busulfan:Ldose: 0 (uM) Interaction | -6.342475612 | 19.0858749 | -0.332312542 | 0.739656504 |
| MeWo:Mechlorethamine.HCl:Ldose: 0 (uM) Interaction | -61.24299797 | 19.0858749 | -3.208812711 | 0.001334794 |
| SKMEL2:Mechlorethamine.HCl:Ldose: 0 (uM) Interaction | -61.41038831 | 19.0858749 | -3.217583089 | 0.001294667 |
| UACC0257:Mechlorethamine.HCl:Ldose: 0 (uM) Interaction | -31.87173207 | 19.0858749 | -1.669912029 | 0.0949513 |
| MeWo:Teniposide:Ldose: 0 (uM) Interaction | -43.5734992 | 19.0858749 | -2.283023411 | 0.022438686 |
| SKMEL2:Teniposide:Ldose: 0 (uM) Interaction | -47.08628897 | 19.0858749 | -2.467075218 | 0.013629894 |
| UACC0257:Teniposide:Ldose: 0 (uM) Interaction | -15.22832444 | 19.0858749 | -0.797884536 | 0.424946294 |
| MeWo:Vinorelbine.Tartrate:Ldose: 0 (uM) Interaction | -48.79003834 | 19.0858749 | -2.556342772 | 0.010584684 |
| SKMEL2:Vinorelbine.Tartrate:Ldose: 0 (uM) Interaction | -38.26712861 | 19.0858749 | -2.004997351 | 0.044975819 |
| UACC0257:Vinorelbine.Tartrate:Ldose: 0 (uM) Interaction | -14.06913014 | 19.0858749 | -0.737148819 | 0.461039883 |
| MeWo:Cabozantinib..XL.184.:Ldose: 0 (uM) Interaction | -65.60383337 | 19.0858749 | -3.437297673 | 0.000588659 |
| SKMEL2:Cabozantinib..XL.184.:Ldose: 0 (uM) Interaction | -42.94180521 | 19.0858749 | -2.24992595 | 0.024463698 |
| UACC0257:Cabozantinib..XL.184.:Ldose: 0 (uM) Interaction | -42.06072671 | 19.0858749 | -2.203762046 | 0.027551624 |
| MeWo:Dacarbazine:Ldose: 0 (uM) Interaction | -53.33137478 | 19.0858749 | -2.794285044 | 0.005206057 |
| SKMEL2:Dacarbazine:Ldose: 0 (uM) Interaction | -58.39358428 | 19.0858749 | -3.05951834 | 0.002219656 |
| UACC0257:Dacarbazine:Ldose: 0 (uM) Interaction | -26.84811248 | 19.0858749 | -1.406700643 | 0.159530602 |
| MeWo:Clofarabine:Ldose: 0 (uM) Interaction | -8.768669049 | 19.0858749 | -0.459432386 | 0.645928332 |
| SKMEL2:Clofarabine:Ldose: 0 (uM) Interaction | -21.15665812 | 19.0858749 | -1.108498208 | 0.267659089 |
| UACC0257:Clofarabine:Ldose: 0 (uM) Interaction | -38.02743687 | 19.0858749 | -1.992438758 | 0.046335537 |
| MeWo:Cisplatin:Ldose: 0 (uM) Interaction | -48.66727744 | 19.0858749 | -2.549910743 | 0.010781913 |
| SKMEL2:Cisplatin:Ldose: 0 (uM) Interaction | -46.61437371 | 19.0858749 | -2.442349327 | 0.014600001 |
| UACC0257:Cisplatin:Ldose: 0 (uM) Interaction | -46.71679963 | 19.0858749 | -2.447715909 | 0.014384421 |
| MeWo:Floxuridine:Ldose: 0 (uM) Interaction | -42.33245129 | 19.0858749 | -2.217998992 | 0.026565292 |
| SKMEL2:Floxuridine:Ldose: 0 (uM) Interaction | 3.875381618 | 19.0858749 | 0.203049723 | 0.839098086 |
| UACC0257:Floxuridine:Ldose: 0 (uM) Interaction | -34.76231528 | 19.0858749 | -1.821363468 | 0.068565489 |
| MeWo:Lomustine..CCNU.:Ldose: 0 (uM) Interaction | -58.56282776 | 19.0858749 | -3.068385813 | 0.002154858 |
| SKMEL2:Lomustine..CCNU.:Ldose: 0 (uM) Interaction | -56.96969111 | 19.0858749 | -2.984913787 | 0.002839767 |
| UACC0257:Lomustine..CCNU.:Ldose: 0 (uM) Interaction | -28.73035807 | 19.0858749 | -1.505320465 | 0.132256369 |
| MeWo:Melphalan:Ldose: 0 (uM) Interaction | -48.82536419 | 19.0858749 | -2.558193662 | 0.010528527 |
| SKMEL2:Melphalan:Ldose: 0 (uM) Interaction | -54.24649996 | 19.0858749 | -2.842232817 | 0.004484086 |
| UACC0257:Melphalan:Ldose: 0 (uM) Interaction | -27.2615699 | 19.0858749 | -1.428363649 | 0.153201716 |
| MeWo:BGJ398..NVPBGJ398.:Ldose: 0 (uM) Interaction | -49.92567821 | 19.0858749 | -2.61584436 | 0.008906919 |
| SKMEL2:BGJ398..NVPBGJ398.:Ldose: 0 (uM) Interaction | -49.41712005 | 19.0858749 | -2.589198573 | 0.009626429 |
| UACC0257:BGJ398..NVPBGJ398.:Ldose: 0 (uM) Interaction | -48.40939915 | 19.0858749 | -2.53639927 | 0.011206896 |
| MeWo:Navitoclax..ABT.263..5uM:Ldose: 0 (uM) Interaction | -67.29896195 | 19.0858749 | -3.526113543 | 0.000422582 |
| SKMEL2:Navitoclax..ABT.263..5uM:Ldose: 0 (uM) Interaction | -59.42443332 | 19.0858749 | -3.113529437 | 0.001851063 |
| UACC0257:Navitoclax..ABT.263..5uM:Ldose: 0 (uM) Interaction | -26.72618315 | 19.0858749 | -1.400312183 | 0.161434258 |
| MeWo:Azacitidine:Ldose: 0 (uM) Interaction | -53.21556305 | 19.0858749 | -2.788217115 | 0.005304548 |
| SKMEL2:Azacitidine:Ldose: 0 (uM) Interaction | -63.10669516 | 19.0858749 | -3.306460694 | 0.000946371 |
| UACC0257:Azacitidine:Ldose: 0 (uM) Interaction | -31.08280582 | 19.0858749 | -1.62857642 | 0.103417343 |
| MeWo:Capecitabine:Ldose: 0 (uM) Interaction | -51.15952987 | 19.0858749 | -2.680491733 | 0.007357002 |
| SKMEL2:Capecitabine:Ldose: 0 (uM) Interaction | -47.4240891 | 19.0858749 | -2.484774178 | 0.012970898 |
| UACC0257:Capecitabine:Ldose: 0 (uM) Interaction | -41.28167052 | 19.0858749 | -2.162943577 | 0.030556485 |
| MeWo:Megestrol.acetate:Ldose: 0 (uM) Interaction | -50.12867879 | 19.0858749 | -2.626480529 | 0.008633397 |
| SKMEL2:Megestrol.acetate:Ldose: 0 (uM) Interaction | -54.32658945 | 19.0858749 | -2.846429087 | 0.004425434 |
| UACC0257:Megestrol.acetate:Ldose: 0 (uM) Interaction | -49.39228696 | 19.0858749 | -2.587897449 | 0.009662854 |
| MeWo:Cytarabine.HCl...Ara.C:Ldose: 0 (uM) Interaction | 2.825583398 | 19.0858749 | 0.148045788 | 0.882308018 |
| SKMEL2:Cytarabine.HCl...Ara.C:Ldose: 0 (uM) Interaction | 3.631293588 | 19.0858749 | 0.190260787 | 0.849106572 |
| UACC0257:Cytarabine.HCl...Ara.C:Ldose: 0 (uM) Interaction | -1.034061691 | 19.0858749 | -0.054179423 | 0.956792717 |
| MeWo:Gemcitabine.HCl:Ldose: 0 (uM) Interaction | -28.20201251 | 19.0858749 | -1.477637922 | 0.139519339 |
| SKMEL2:Gemcitabine.HCl:Ldose: 0 (uM) Interaction | 14.84310658 | 19.0858749 | 0.777701135 | 0.436753764 |
| UACC0257:Gemcitabine.HCl:Ldose: 0 (uM) Interaction | -52.27260024 | 19.0858749 | -2.738810797 | 0.006171254 |
| MeWo:Vinblastine.Sulfate:Ldose: 0 (uM) Interaction | -59.80851364 | 19.0858749 | -3.133653237 | 0.001728763 |
| SKMEL2:Vinblastine.Sulfate:Ldose: 0 (uM) Interaction | -47.64649741 | 19.0858749 | -2.49642721 | 0.012552554 |
| UACC0257:Vinblastine.Sulfate:Ldose: 0 (uM) Interaction | -16.47481901 | 19.0858749 | -0.863194331 | 0.388040241 |
| MeWo:MLN9708..MLN2238.:Ldose: 0 (uM) Interaction | -70.97478477 | 19.0858749 | -3.718707429 | 0.000200753 |
| SKMEL2:MLN9708..MLN2238.:Ldose: 0 (uM) Interaction | -64.98026272 | 19.0858749 | -3.404625833 | 0.000663755 |
| UACC0257:MLN9708..MLN2238.:Ldose: 0 (uM) Interaction | -36.62649224 | 19.0858749 | -1.919036588 | 0.054992902 |
| MeWo:ABT.737:Ldose: 0 (uM) Interaction | -79.04422266 | 19.0858749 | -4.141503761 | 3.46E-05 |
| SKMEL2:ABT.737:Ldose: 0 (uM) Interaction | -69.86965525 | 19.0858749 | -3.660804424 | 0.000252026 |
| UACC0257:ABT.737:Ldose: 0 (uM) Interaction | -48.33497918 | 19.0858749 | -2.532500053 | 0.011332275 |
| MeWo:Streptozocin:Ldose: 0 (uM) Interaction | -47.89750609 | 19.0858749 | -2.509578751 | 0.012094802 |
| SKMEL2:Streptozocin:Ldose: 0 (uM) Interaction | -53.07423965 | 19.0858749 | -2.780812509 | 0.005427013 |
| UACC0257:Streptozocin:Ldose: 0 (uM) Interaction | -28.00780399 | 19.0858749 | -1.467462411 | 0.142264908 |
| MeWo:Crizotinib:Ldose: 0 (uM) Interaction | -54.23620556 | 19.0858749 | -2.841693443 | 0.004491675 |
| SKMEL2:Crizotinib:Ldose: 0 (uM) Interaction | -49.64819679 | 19.0858749 | -2.601305786 | 0.009293313 |
| UACC0257:Crizotinib:Ldose: 0 (uM) Interaction | -55.76111966 | 19.0858749 | -2.921590965 | 0.003486102 |
| MeWo:Sunitinib:Ldose: 0 (uM) Interaction | -55.96123527 | 19.0858749 | -2.932075976 | 0.003370586 |
| SKMEL2:Sunitinib:Ldose: 0 (uM) Interaction | -56.78899853 | 19.0858749 | -2.975446441 | 0.002928871 |
| UACC0257:Sunitinib:Ldose: 0 (uM) Interaction | -39.88172572 | 19.0858749 | -2.089593793 | 0.036666006 |
| MeWo:Dexrazoxane:Ldose: 0 (uM) Interaction | -57.6259774 | 19.0858749 | -3.019299755 | 0.002536563 |
| SKMEL2:Dexrazoxane:Ldose: 0 (uM) Interaction | -62.73070834 | 19.0858749 | -3.286760952 | 0.001015089 |
| UACC0257:Dexrazoxane:Ldose: 0 (uM) Interaction | -42.39730647 | 19.0858749 | -2.221397065 | 0.026334434 |
| MeWo:Mitomycin.C:Ldose: 0 (uM) Interaction | -32.5533207 | 19.0858749 | -1.705623707 | 0.08809258 |
| SKMEL2:Mitomycin.C:Ldose: 0 (uM) Interaction | -42.93263902 | 19.0858749 | -2.24944569 | 0.024494211 |
| UACC0257:Mitomycin.C:Ldose: 0 (uM) Interaction | -51.85048805 | 19.0858749 | -2.716694327 | 0.006599012 |
| MeWo:Carfilzomib:Ldose: 0 (uM) Interaction | -92.95362083 | 19.0858749 | -4.87028346 | 1.12E-06 |
| SKMEL2:Carfilzomib:Ldose: 0 (uM) Interaction | -22.03530713 | 19.0858749 | -1.15453482 | 0.24829378 |
| UACC0257:Carfilzomib:Ldose: 0 (uM) Interaction | -73.69020365 | 19.0858749 | -3.860981173 | 0.000113262 |
| MeWo:OSI.027:Ldose: 0 (uM) Interaction | -83.76655901 | 19.0858749 | -4.388929481 | 1.14E-05 |
| SKMEL2:OSI.027:Ldose: 0 (uM) Interaction | -77.08122712 | 19.0858749 | -4.038653064 | 5.39E-05 |
| UACC0257:OSI.027:Ldose: 0 (uM) Interaction | -60.48002023 | 19.0858749 | -3.168836669 | 0.001532643 |
| MeWo:Bioymifi:Ldose: 0 (uM) Interaction | -59.0790188 | 19.0858749 | -3.095431523 | 0.001967788 |
| SKMEL2:Bioymifi:Ldose: 0 (uM) Interaction | -57.61534097 | 19.0858749 | -3.018742462 | 0.002541231 |
| UACC0257:Bioymifi:Ldose: 0 (uM) Interaction | -48.15840434 | 19.0858749 | -2.523248455 | 0.011634757 |
| MeWo:Nelarabine:Ldose: 0 (uM) Interaction | -61.16195794 | 19.0858749 | -3.204566637 | 0.00135463 |
| SKMEL2:Nelarabine:Ldose: 0 (uM) Interaction | -60.21569437 | 19.0858749 | -3.154987377 | 0.001607258 |
| UACC0257:Nelarabine:Ldose: 0 (uM) Interaction | -32.15577814 | 19.0858749 | -1.684794557 | 0.092042702 |
| MeWo:Raloxifene:Ldose: 0 (uM) Interaction | -55.86556641 | 19.0858749 | -2.927063428 | 0.003425368 |
| SKMEL2:Raloxifene:Ldose: 0 (uM) Interaction | -50.86679769 | 19.0858749 | -2.665154098 | 0.007701042 |
| UACC0257:Raloxifene:Ldose: 0 (uM) Interaction | -54.14252481 | 19.0858749 | -2.836785062 | 0.00456128 |
| MeWo:Quinacrine.HCl:Ldose: 0 (uM) Interaction | -60.77276155 | 19.0858749 | -3.184174782 | 0.001453739 |
| SKMEL2:Quinacrine.HCl:Ldose: 0 (uM) Interaction | -56.45205331 | 19.0858749 | -2.957792274 | 0.003101873 |
| UACC0257:Quinacrine.HCl:Ldose: 0 (uM) Interaction | -41.42883188 | 19.0858749 | -2.170654063 | 0.029968218 |
| MeWo:Lenalidomide:Ldose: 0 (uM) Interaction | -58.93829998 | 19.0858749 | -3.088058593 | 0.002017248 |
| SKMEL2:Lenalidomide:Ldose: 0 (uM) Interaction | -60.44146 | 19.0858749 | -3.166816315 | 0.001543325 |
| UACC0257:Lenalidomide:Ldose: 0 (uM) Interaction | -40.19134928 | 19.0858749 | -2.105816448 | 0.035231842 |
| MeWo:Fludarabine.Phosphate:Ldose: 0 (uM) Interaction | -63.50660496 | 19.0858749 | -3.327413876 | 0.000878028 |
| SKMEL2:Fludarabine.Phosphate:Ldose: 0 (uM) Interaction | -53.17823887 | 19.0858749 | -2.786261524 | 0.005336646 |
| UACC0257:Fludarabine.Phosphate:Ldose: 0 (uM) Interaction | -37.18669558 | 19.0858749 | -1.948388313 | 0.051381485 |
| MeWo:Nilotinib:Ldose: 0 (uM) Interaction | -57.9398143 | 19.0858749 | -3.035743167 | 0.002402302 |
| SKMEL2:Nilotinib:Ldose: 0 (uM) Interaction | -57.51376908 | 19.0858749 | -3.013420626 | 0.002586208 |
| UACC0257:Nilotinib:Ldose: 0 (uM) Interaction | -36.58139583 | 19.0858749 | -1.916673772 | 0.055292606 |
| MeWo:Linsitinib:Ldose: 0 (uM) Interaction | -43.69055371 | 19.0858749 | -2.289156454 | 0.022079894 |
| SKMEL2:Linsitinib:Ldose: 0 (uM) Interaction | -47.32686873 | 19.0858749 | -2.479680339 | 0.013157609 |
| UACC0257:Linsitinib:Ldose: 0 (uM) Interaction | -49.44585213 | 19.0858749 | -2.590703984 | 0.009584438 |
| MeWo:Aphrocallistin.analogue:Ldose: 0 (uM) Interaction | -35.82784682 | 19.0858749 | -1.877191746 | 0.060505322 |
| SKMEL2:Aphrocallistin.analogue:Ldose: 0 (uM) Interaction | 10.92581384 | 19.0858749 | 0.572455489 | 0.567019408 |
| UACC0257:Aphrocallistin.analogue:Ldose: 0 (uM) Interaction | -1.307936454 | 19.0858749 | -0.068529028 | 0.94536515 |
| MeWo:Mitotane..o.p..DDD..Lysodren.:Ldose: 0 (uM) Interaction | -50.84273487 | 19.0858749 | -2.663893332 | 0.007729954 |
| SKMEL2:Mitotane..o.p..DDD..Lysodren.:Ldose: 0 (uM) Interaction | -59.94456516 | 19.0858749 | -3.140781624 | 0.001687255 |
| UACC0257:Mitotane..o.p..DDD..Lysodren.:Ldose: 0 (uM) Interaction | -22.63027935 | 19.0858749 | -1.185708252 | 0.235750614 |
| MeWo:Etoposide:Ldose: 0 (uM) Interaction | -36.83330556 | 19.0858749 | -1.929872524 | 0.05363575 |
| SKMEL2:Etoposide:Ldose: 0 (uM) Interaction | -26.61465259 | 19.0858749 | -1.394468565 | 0.163190537 |
| UACC0257:Etoposide:Ldose: 0 (uM) Interaction | -19.42168959 | 19.0858749 | -1.017594933 | 0.308881926 |
| MeWo:Vandetanib:Ldose: 0 (uM) Interaction | -73.64868063 | 19.0858749 | -3.858805584 | 0.000114275 |
| SKMEL2:Vandetanib:Ldose: 0 (uM) Interaction | -66.63484381 | 19.0858749 | -3.491317227 | 0.000481607 |
| UACC0257:Vandetanib:Ldose: 0 (uM) Interaction | -44.53956748 | 19.0858749 | -2.333640335 | 0.019623713 |
| MeWo:Carboplatin:Ldose: 0 (uM) Interaction | -60.6775876 | 19.0858749 | -3.179188165 | 0.001478971 |
| SKMEL2:Carboplatin:Ldose: 0 (uM) Interaction | -56.61087446 | 19.0858749 | -2.966113671 | 0.003019199 |
| UACC0257:Carboplatin:Ldose: 0 (uM) Interaction | -41.94695477 | 19.0858749 | -2.197800991 | 0.027973894 |
| MeWo:Gefitinib:Ldose: 0 (uM) Interaction | -60.57163719 | 19.0858749 | -3.173636918 | 0.001507535 |
| SKMEL2:Gefitinib:Ldose: 0 (uM) Interaction | -64.29637737 | 19.0858749 | -3.368793818 | 0.000756302 |
| UACC0257:Gefitinib:Ldose: 0 (uM) Interaction | -30.98225157 | 19.0858749 | -1.623307904 | 0.104538234 |
| MeWo:Vincristine.Sulfate:Ldose: 0 (uM) Interaction | -30.07587537 | 19.0858749 | -1.575818532 | 0.115082316 |
| SKMEL2:Vincristine.Sulfate:Ldose: 0 (uM) Interaction | -4.447087084 | 19.0858749 | -0.233004099 | 0.815760437 |
| UACC0257:Vincristine.Sulfate:Ldose: 0 (uM) Interaction | 21.78676459 | 19.0858749 | 1.141512491 | 0.253669407 |
| MeWo:Trametinib..GSK1120212.:Ldose: 0 (uM) Interaction | -76.04154205 | 19.0858749 | -3.984179005 | 6.79E-05 |
| SKMEL2:Trametinib..GSK1120212.:Ldose: 0 (uM) Interaction | -61.38764362 | 19.0858749 | -3.216391387 | 0.001300053 |
| UACC0257:Trametinib..GSK1120212.:Ldose: 0 (uM) Interaction | -29.3839702 | 19.0858749 | -1.539566321 | 0.123680789 |
| MeWo:MLN4924:Ldose: 0 (uM) Interaction | -30.47023166 | 19.0858749 | -1.596480739 | 0.110396172 |
| SKMEL2:MLN4924:Ldose: 0 (uM) Interaction | -9.913450584 | 19.0858749 | -0.51941295 | 0.603478139 |
| UACC0257:MLN4924:Ldose: 0 (uM) Interaction | 2.131776581 | 19.0858749 | 0.111693941 | 0.911067125 |
| MeWo:Bortezomib:Ldose: 0 (uM) Interaction | -50.25845971 | 19.0858749 | -2.63328037 | 0.00846249 |
| SKMEL2:Bortezomib:Ldose: 0 (uM) Interaction | -36.0137602 | 19.0858749 | -1.886932635 | 0.059182823 |
| UACC0257:Bortezomib:Ldose: 0 (uM) Interaction | -14.46595595 | 19.0858749 | -0.757940415 | 0.448494946 |
| MeWo:Fluorouracil...5.FU.:Ldose: 0 (uM) Interaction | -61.79572928 | 19.0858749 | -3.23777294 | 0.001206489 |
| SKMEL2:Fluorouracil...5.FU.:Ldose: 0 (uM) Interaction | -58.41820137 | 19.0858749 | -3.060808147 | 0.002210121 |
| UACC0257:Fluorouracil...5.FU.:Ldose: 0 (uM) Interaction | -43.26172358 | 19.0858749 | -2.266687999 | 0.023419164 |
| MeWo:Lapatinib:Ldose: 0 (uM) Interaction | -61.0711638 | 19.0858749 | -3.1998095 | 0.001377177 |
| SKMEL2:Lapatinib:Ldose: 0 (uM) Interaction | -61.88639423 | 19.0858749 | -3.242523309 | 0.001186565 |
| UACC0257:Lapatinib:Ldose: 0 (uM) Interaction | -31.30034922 | 19.0858749 | -1.639974557 | 0.101025059 |
| MeWo:Mitoxantrone:Ldose: 0 (uM) Interaction | -21.29207618 | 19.0858749 | -1.115593406 | 0.264608662 |
| SKMEL2:Mitoxantrone:Ldose: 0 (uM) Interaction | -4.413899722 | 19.0858749 | -0.231265255 | 0.817110934 |
| UACC0257:Mitoxantrone:Ldose: 0 (uM) Interaction | -12.35315843 | 19.0858749 | -0.647240878 | 0.517482956 |
| MeWo:Imatinib:Ldose: 0 (uM) Interaction | -64.80019743 | 19.0858749 | -3.395191354 | 0.000687044 |
| SKMEL2:Imatinib:Ldose: 0 (uM) Interaction | -60.81120269 | 19.0858749 | -3.186188897 | 0.00144366 |
| UACC0257:Imatinib:Ldose: 0 (uM) Interaction | -32.81675343 | 19.0858749 | -1.719426204 | 0.08555116 |
| MeWo:Imiquimod:Ldose: 0 (uM) Interaction | -61.72652801 | 19.0858749 | -3.234147156 | 0.001221904 |
| SKMEL2:Imiquimod:Ldose: 0 (uM) Interaction | -57.80353795 | 19.0858749 | -3.028602999 | 0.002459782 |
| UACC0257:Imiquimod:Ldose: 0 (uM) Interaction | -20.71718725 | 19.0858749 | -1.085472233 | 0.277724703 |
| MeWo:Dacomitinib..PF299804.:Ldose: 0 (uM) Interaction | -64.24177561 | 19.0858749 | -3.365932972 | 0.000764185 |
| SKMEL2:Dacomitinib..PF299804.:Ldose: 0 (uM) Interaction | -55.71806484 | 19.0858749 | -2.919335118 | 0.003511421 |
| UACC0257:Dacomitinib..PF299804.:Ldose: 0 (uM) Interaction | -26.45335948 | 19.0858749 | -1.38601765 | 0.165755862 |
| MeWo:PD325901:Ldose: 0 (uM) Interaction | -85.96078665 | 19.0858749 | -4.503895531 | 6.71E-06 |
| SKMEL2:PD325901:Ldose: 0 (uM) Interaction | -85.17908764 | 19.0858749 | -4.462938592 | 8.13E-06 |
| UACC0257:PD325901:Ldose: 0 (uM) Interaction | -35.95974236 | 19.0858749 | -1.884102383 | 0.059564584 |
| MeWo:Vismodegib:Ldose: 0 (uM) Interaction | -51.63049448 | 19.0858749 | -2.705167814 | 0.006832358 |
| SKMEL2:Vismodegib:Ldose: 0 (uM) Interaction | -57.9665698 | 19.0858749 | -3.037145015 | 0.002391162 |
| UACC0257:Vismodegib:Ldose: 0 (uM) Interaction | -28.00066852 | 19.0858749 | -1.46708855 | 0.142366568 |
| MeWo:Temozolomide:Ldose: 0 (uM) Interaction | -59.13660945 | 19.0858749 | -3.098448972 | 0.001947868 |
| SKMEL2:Temozolomide:Ldose: 0 (uM) Interaction | -60.00339302 | 19.0858749 | -3.143863897 | 0.001669593 |
| UACC0257:Temozolomide:Ldose: 0 (uM) Interaction | -36.49711589 | 19.0858749 | -1.912257944 | 0.055856367 |
| MeWo:Mercaptopurine:Ldose: 0 (uM) Interaction | -63.85461672 | 19.0858749 | -3.345647871 | 0.000822308 |
| SKMEL2:Mercaptopurine:Ldose: 0 (uM) Interaction | -56.54919367 | 19.0858749 | -2.962881921 | 0.003051065 |
| UACC0257:Mercaptopurine:Ldose: 0 (uM) Interaction | -22.32036434 | 19.0858749 | -1.169470326 | 0.242227131 |
| MeWo:Dasatinib:Ldose: 0 (uM) Interaction | -61.98760205 | 19.0858749 | -3.24782607 | 0.001164683 |
| SKMEL2:Dasatinib:Ldose: 0 (uM) Interaction | -67.01455089 | 19.0858749 | -3.511211891 | 0.000446981 |
| UACC0257:Dasatinib:Ldose: 0 (uM) Interaction | -23.39208595 | 19.0858749 | -1.225622932 | 0.220354004 |
| MeWo:Daunorubicin.HCl:Ldose: 0 (uM) Interaction | -43.07868671 | 19.0858749 | -2.257097825 | 0.024011937 |
| SKMEL2:Daunorubicin.HCl:Ldose: 0 (uM) Interaction | -13.32470971 | 19.0858749 | -0.698145083 | 0.485094003 |
| UACC0257:Daunorubicin.HCl:Ldose: 0 (uM) Interaction | -42.96585601 | 19.0858749 | -2.251186086 | 0.024383792 |
| MeWo:Sirolimus..Rapamycin.:Ldose: 0 (uM) Interaction | -61.95801003 | 19.0858749 | -3.246275603 | 0.001171042 |
| SKMEL2:Sirolimus..Rapamycin.:Ldose: 0 (uM) Interaction | -59.25105969 | 19.0858749 | -3.104445566 | 0.001908831 |
| UACC0257:Sirolimus..Rapamycin.:Ldose: 0 (uM) Interaction | -43.91326011 | 19.0858749 | -2.300825105 | 0.021411023 |
| MeWo:INK.128..MLN0128.:Ldose: 0 (uM) Interaction | -32.23867505 | 19.0858749 | -1.689137922 | 0.091207475 |
| SKMEL2:INK.128..MLN0128.:Ldose: 0 (uM) Interaction | -50.59090538 | 19.0858749 | -2.650698784 | 0.008038417 |
| UACC0257:INK.128..MLN0128.:Ldose: 0 (uM) Interaction | -27.62209241 | 19.0858749 | -1.447253142 | 0.14784059 |
| MeWo:Quizartinib:Ldose: 0 (uM) Interaction | -53.18269198 | 19.0858749 | -2.786494843 | 0.005332807 |
| SKMEL2:Quizartinib:Ldose: 0 (uM) Interaction | -51.99077123 | 19.0858749 | -2.724044431 | 0.006453982 |
| UACC0257:Quizartinib:Ldose: 0 (uM) Interaction | -38.38452129 | 19.0858749 | -2.011148114 | 0.044322249 |
| MeWo:Sorafenib:Ldose: 0 (uM) Interaction | -46.67188599 | 19.0858749 | -2.44536267 | 0.014478605 |
| SKMEL2:Sorafenib:Ldose: 0 (uM) Interaction | -45.42470324 | 19.0858749 | -2.380016818 | 0.017320497 |
| UACC0257:Sorafenib:Ldose: 0 (uM) Interaction | -29.8777389 | 19.0858749 | -1.565437218 | 0.117495117 |
| MeWo:Carmustine:Ldose: 0 (uM) Interaction | -54.78820438 | 19.0858749 | -2.870615294 | 0.004100728 |
| SKMEL2:Carmustine:Ldose: 0 (uM) Interaction | -48.80813071 | 19.0858749 | -2.557290718 | 0.010555889 |
| UACC0257:Carmustine:Ldose: 0 (uM) Interaction | -30.31966584 | 19.0858749 | -1.588591878 | 0.11216724 |
| MeWo:Uracil.mustard:Ldose: 0 (uM) Interaction | -50.46231793 | 19.0858749 | -2.643961474 | 0.008200135 |
| SKMEL2:Uracil.mustard:Ldose: 0 (uM) Interaction | -50.35615319 | 19.0858749 | -2.638398997 | 0.008335841 |
| UACC0257:Uracil.mustard:Ldose: 0 (uM) Interaction | -47.55417329 | 19.0858749 | -2.491589909 | 0.012724741 |
| MeWo:Ixabepilone:Ldose: 0 (uM) Interaction | -33.49133272 | 19.0858749 | -1.75477063 | 0.079312766 |
| SKMEL2:Ixabepilone:Ldose: 0 (uM) Interaction | -30.82391506 | 19.0858749 | -1.615011899 | 0.106322764 |
| UACC0257:Ixabepilone:Ldose: 0 (uM) Interaction | -17.7221408 | 19.0858749 | -0.928547468 | 0.353134062 |
| MeWo:Valrubicin:Ldose: 0 (uM) Interaction | -29.06604103 | 19.0858749 | -1.522908496 | 0.12779628 |
| SKMEL2:Valrubicin:Ldose: 0 (uM) Interaction | -41.35662175 | 19.0858749 | -2.166870629 | 0.030255645 |
| UACC0257:Valrubicin:Ldose: 0 (uM) Interaction | -22.70219719 | 19.0858749 | -1.18947637 | 0.234265375 |
| MeWo:Triethylenemelamine:Ldose: 0 (uM) Interaction | -58.41672166 | 19.0858749 | -3.060730618 | 0.002210693 |
| SKMEL2:Triethylenemelamine:Ldose: 0 (uM) Interaction | -56.80328953 | 19.0858749 | -2.976195215 | 0.002921732 |
| UACC0257:Triethylenemelamine:Ldose: 0 (uM) Interaction | -33.98008131 | 19.0858749 | -1.7803785 | 0.075028129 |
| MeWo:Palbociclib..PD.0332991..Isethionate:Ldose: 0 (uM) Interaction | -42.69410604 | 19.0858749 | -2.236947809 | 0.025299941 |
| SKMEL2:Palbociclib..PD.0332991..Isethionate:Ldose: 0 (uM) Interaction | -40.8595997 | 19.0858749 | -2.140829274 | 0.032299 |
| UACC0257:Palbociclib..PD.0332991..Isethionate:Ldose: 0 (uM) Interaction | -33.14159104 | 19.0858749 | -1.736445996 | 0.082499303 |
| MeWo:Afatinib:Ldose: 0 (uM) Interaction | -51.62712102 | 19.0858749 | -2.704991063 | 0.006835993 |
| SKMEL2:Afatinib:Ldose: 0 (uM) Interaction | -55.558849 | 19.0858749 | -2.910993041 | 0.003606512 |
| UACC0257:Afatinib:Ldose: 0 (uM) Interaction | -31.51285229 | 19.0858749 | -1.651108606 | 0.098730978 |
| MeWo:Doxorubicin.HCl:Ldose: 0 (uM) Interaction | -15.86370121 | 19.0858749 | -0.831174955 | 0.405884014 |
| SKMEL2:Doxorubicin.HCl:Ldose: 0 (uM) Interaction | -17.94156036 | 19.0858749 | -0.940043905 | 0.347205582 |
| UACC0257:Doxorubicin.HCl:Ldose: 0 (uM) Interaction | -11.73315633 | 19.0858749 | -0.614756012 | 0.538722357 |
| MeWo:Exemestane:Ldose: 0 (uM) Interaction | -58.55071181 | 19.0858749 | -3.067751 | 0.002159439 |
| SKMEL2:Exemestane:Ldose: 0 (uM) Interaction | -51.53291185 | 19.0858749 | -2.700054995 | 0.006938216 |
| UACC0257:Exemestane:Ldose: 0 (uM) Interaction | -49.50863281 | 19.0858749 | -2.593993363 | 0.009493253 |
| MeWo:Tretinoin:Ldose: 0 (uM) Interaction | -51.79605897 | 19.0858749 | -2.713842527 | 0.006656068 |
| SKMEL2:Tretinoin:Ldose: 0 (uM) Interaction | -48.70945819 | 19.0858749 | -2.552120794 | 0.010713779 |
| UACC0257:Tretinoin:Ldose: 0 (uM) Interaction | -39.4002585 | 19.0858749 | -2.06436743 | 0.038994836 |
| MeWo:Fulvestrant:Ldose: 0 (uM) Interaction | -61.00373313 | 19.0858749 | -3.196276485 | 0.001394145 |
| SKMEL2:Fulvestrant:Ldose: 0 (uM) Interaction | -59.79683525 | 19.0858749 | -3.13304135 | 0.00173237 |
| UACC0257:Fulvestrant:Ldose: 0 (uM) Interaction | -22.45732304 | 19.0858749 | -1.176646246 | 0.239349734 |
| MeWo:Docetaxel:Ldose: 0 (uM) Interaction | -39.04043624 | 19.0858749 | -2.045514626 | 0.04081627 |
| SKMEL2:Docetaxel:Ldose: 0 (uM) Interaction | -45.56310028 | 19.0858749 | -2.387268099 | 0.016982679 |
| UACC0257:Docetaxel:Ldose: 0 (uM) Interaction | -21.7030084 | 19.0858749 | -1.137124104 | 0.255499058 |
| MeWo:Everolimus:Ldose: 0 (uM) Interaction | -64.36763637 | 19.0858749 | -3.372527417 | 0.000746127 |
| SKMEL2:Everolimus:Ldose: 0 (uM) Interaction | -52.92233363 | 19.0858749 | -2.772853428 | 0.00556149 |
| UACC0257:Everolimus:Ldose: 0 (uM) Interaction | -50.14435522 | 19.0858749 | -2.627301892 | 0.008612591 |
| MeWo:MLN.2480:Ldose: 0 (uM) Interaction | -31.19876585 | 19.0858749 | -1.634652119 | 0.102136608 |
| SKMEL2:MLN.2480:Ldose: 0 (uM) Interaction | -18.63149105 | 19.0858749 | -0.976192663 | 0.328979961 |
| UACC0257:MLN.2480:Ldose: 0 (uM) Interaction | -56.96021238 | 19.0858749 | -2.984417151 | 0.002844379 |
| MeWo:LY2157299:Ldose: 0 (uM) Interaction | -59.74237676 | 19.0858749 | -3.13018801 | 0.001749279 |
| SKMEL2:LY2157299:Ldose: 0 (uM) Interaction | -51.59500844 | 19.0858749 | -2.703308532 | 0.006870684 |
| UACC0257:LY2157299:Ldose: 0 (uM) Interaction | -22.10591193 | 19.0858749 | -1.158234142 | 0.246781341 |
| MeWo:Allopurinol:Ldose: 0 (uM) Interaction | -59.70326329 | 19.0858749 | -3.128138669 | 0.001761516 |
| SKMEL2:Allopurinol:Ldose: 0 (uM) Interaction | -60.99387486 | 19.0858749 | -3.195759964 | 0.001396642 |
| UACC0257:Allopurinol:Ldose: 0 (uM) Interaction | -36.46591974 | 19.0858749 | -1.910623429 | 0.056066254 |
| MeWo:Pipobroman:Ldose: 0 (uM) Interaction | -54.66673649 | 19.0858749 | -2.864251012 | 0.004184005 |
| SKMEL2:Pipobroman:Ldose: 0 (uM) Interaction | -52.11249571 | 19.0858749 | -2.730422157 | 0.00633047 |
| UACC0257:Pipobroman:Ldose: 0 (uM) Interaction | -41.68897564 | 19.0858749 | -2.184284235 | 0.028952113 |
| MeWo:Letrozole:Ldose: 0 (uM) Interaction | -46.61322654 | 19.0858749 | -2.442289221 | 0.014602432 |
| SKMEL2:Letrozole:Ldose: 0 (uM) Interaction | -39.5788666 | 19.0858749 | -2.073725559 | 0.038116661 |
| UACC0257:Letrozole:Ldose: 0 (uM) Interaction | -43.34232657 | 19.0858749 | -2.270911174 | 0.023162182 |
| MeWo:Thiotepa:Ldose: 0 (uM) Interaction | -65.36014362 | 19.0858749 | -3.424529605 | 0.000617012 |
| SKMEL2:Thiotepa:Ldose: 0 (uM) Interaction | -53.94400458 | 19.0858749 | -2.826383641 | 0.004712019 |
| UACC0257:Thiotepa:Ldose: 0 (uM) Interaction | -25.97247174 | 19.0858749 | -1.360821649 | 0.173584296 |
| MeWo:Plicamycin:Ldose: 0 (uM) Interaction | -57.66166695 | 19.0858749 | -3.021169701 | 0.002520956 |
| SKMEL2:Plicamycin:Ldose: 0 (uM) Interaction | -29.57357151 | 19.0858749 | -1.549500438 | 0.121276158 |
| UACC0257:Plicamycin:Ldose: 0 (uM) Interaction | -20.10351686 | 19.0858749 | -1.053319116 | 0.292206576 |
| MeWo:Erlotinib.HCl:Ldose: 0 (uM) Interaction | -82.33258759 | 19.0858749 | -4.31379688 | 1.61E-05 |
| SKMEL2:Erlotinib.HCl:Ldose: 0 (uM) Interaction | -69.91844472 | 19.0858749 | -3.663360736 | 0.000249524 |
| UACC0257:Erlotinib.HCl:Ldose: 0 (uM) Interaction | -54.97214947 | 19.0858749 | -2.880253055 | 0.003977479 |
| MeWo:MEK.162..ARRY.438162.:Ldose: 0 (uM) Interaction | -34.58377888 | 19.0858749 | -1.812009094 | 0.06999871 |
| SKMEL2:MEK.162..ARRY.438162.:Ldose: 0 (uM) Interaction | -18.17159655 | 19.0858749 | -0.952096598 | 0.341058678 |
| UACC0257:MEK.162..ARRY.438162.:Ldose: 0 (uM) Interaction | -12.74808497 | 19.0858749 | -0.667932963 | 0.504183542 |
| MeWo:Baricitinib..LY3009104..INCB028050.:Ldose: 0 (uM) Interaction | -50.78641678 | 19.0858749 | -2.660942558 | 0.007798001 |
| SKMEL2:Baricitinib..LY3009104..INCB028050.:Ldose: 0 (uM) Interaction | -39.65815461 | 19.0858749 | -2.077879837 | 0.037732243 |
| UACC0257:Baricitinib..LY3009104..INCB028050.:Ldose: 0 (uM) Interaction | -30.62854633 | 19.0858749 | -1.604775599 | 0.108557858 |
| MeWo:Arsenic.Trioxide:Ldose: 0 (uM) Interaction | -48.33568416 | 19.0858749 | -2.53253699 | 0.011331082 |
| SKMEL2:Arsenic.Trioxide:Ldose: 0 (uM) Interaction | -48.88279275 | 19.0858749 | -2.561202618 | 0.010437799 |
| UACC0257:Arsenic.Trioxide:Ldose: 0 (uM) Interaction | -22.73407103 | 19.0858749 | -1.191146392 | 0.233609245 |
| MeWo:Celecoxib:Ldose: 0 (uM) Interaction | -49.71546201 | 19.0858749 | -2.604830131 | 0.009198297 |
| SKMEL2:Celecoxib:Ldose: 0 (uM) Interaction | -44.87132007 | 19.0858749 | -2.351022435 | 0.018730867 |
| UACC0257:Celecoxib:Ldose: 0 (uM) Interaction | -41.51153441 | 19.0858749 | -2.174987242 | 0.029641914 |
| MeWo:Bendamustine.HCl:Ldose: 0 (uM) Interaction | -56.20878008 | 19.0858749 | -2.945046029 | 0.003232518 |
| SKMEL2:Bendamustine.HCl:Ldose: 0 (uM) Interaction | -48.06911964 | 19.0858749 | -2.518570403 | 0.011790416 |
| UACC0257:Bendamustine.HCl:Ldose: 0 (uM) Interaction | -49.04945462 | 19.0858749 | -2.569934828 | 0.010178426 |
| MeWo:Chlorambucil:Ldose: 0 (uM) Interaction | -60.15405339 | 19.0858749 | -3.151757712 | 0.001625132 |
| SKMEL2:Chlorambucil:Ldose: 0 (uM) Interaction | -50.87533949 | 19.0858749 | -2.665601643 | 0.007690802 |
| UACC0257:Chlorambucil:Ldose: 0 (uM) Interaction | -47.49894226 | 19.0858749 | -2.488696092 | 0.012828744 |
| MeWo:Zoledronic.Acid:Ldose: 0 (uM) Interaction | -60.54278156 | 19.0858749 | -3.172125034 | 0.001515402 |
| SKMEL2:Zoledronic.Acid:Ldose: 0 (uM) Interaction | -53.29628437 | 19.0858749 | -2.79244649 | 0.005235723 |
| UACC0257:Zoledronic.Acid:Ldose: 0 (uM) Interaction | -45.00262431 | 19.0858749 | -2.35790209 | 0.018387428 |
| MeWo:Actinomycin.D:Ldose: 0 (uM) Interaction | -88.00750123 | 19.0858749 | -4.611132668 | 4.03E-06 |
| SKMEL2:Actinomycin.D:Ldose: 0 (uM) Interaction | -84.47814708 | 19.0858749 | -4.426212974 | 9.64E-06 |
| UACC0257:Actinomycin.D:Ldose: 0 (uM) Interaction | -54.14295707 | 19.0858749 | -2.836807711 | 0.004560957 |
| MeWo:Temsirolimus..CCI.779..Torisel.:Ldose: 0 (uM) Interaction | -61.76161101 | 19.0858749 | -3.235985322 | 0.001214067 |
| SKMEL2:Temsirolimus..CCI.779..Torisel.:Ldose: 0 (uM) Interaction | -48.85356391 | 19.0858749 | -2.559671179 | 0.010483888 |
| UACC0257:Temsirolimus..CCI.779..Torisel.:Ldose: 0 (uM) Interaction | -42.71468294 | 19.0858749 | -2.238025931 | 0.025229542 |
| MeWo:Foretinib..GSK1363089.:Ldose: 0 (uM) Interaction | -58.5458145 | 19.0858749 | -3.067494407 | 0.002161293 |
| SKMEL2:Foretinib..GSK1363089.:Ldose: 0 (uM) Interaction | -18.72093364 | 19.0858749 | -0.980878987 | 0.326663437 |
| UACC0257:Foretinib..GSK1363089.:Ldose: 0 (uM) Interaction | -37.40235825 | 19.0858749 | -1.959687908 | 0.050045155 |
| MeWo:Decitabine:Ldose: 0 (uM) Interaction | -56.97179015 | 19.0858749 | -2.985023766 | 0.002838747 |
| SKMEL2:Decitabine:Ldose: 0 (uM) Interaction | -50.43815543 | 19.0858749 | -2.642695486 | 0.008230846 |
| UACC0257:Decitabine:Ldose: 0 (uM) Interaction | -21.75141464 | 19.0858749 | -1.139660338 | 0.25444051 |
| MeWo:Methotrexate:Ldose: 0 (uM) Interaction | -55.69101509 | 19.0858749 | -2.917917853 | 0.003527414 |
| SKMEL2:Methotrexate:Ldose: 0 (uM) Interaction | -50.37507345 | 19.0858749 | -2.63939032 | 0.00831151 |
| UACC0257:Methotrexate:Ldose: 0 (uM) Interaction | -45.49007171 | 19.0858749 | -2.383441784 | 0.01716021 |
| MeWo:Axitinib:Ldose: 0 (uM) Interaction | -53.31212913 | 19.0858749 | -2.793276673 | 0.005222309 |
| SKMEL2:Axitinib:Ldose: 0 (uM) Interaction | -47.12967944 | 19.0858749 | -2.469348652 | 0.013543623 |
| UACC0257:Axitinib:Ldose: 0 (uM) Interaction | -54.66964327 | 19.0858749 | -2.864403312 | 0.004181995 |
| MeWo:Oxaliplatin:Ldose: 0 (uM) Interaction | -64.20593413 | 19.0858749 | -3.364055066 | 0.000769401 |
| SKMEL2:Oxaliplatin:Ldose: 0 (uM) Interaction | -50.38066465 | 19.0858749 | -2.63968327 | 0.008304332 |
| UACC0257:Oxaliplatin:Ldose: 0 (uM) Interaction | -44.74369871 | 19.0858749 | -2.344335743 | 0.019070039 |
| MeWo:Cabazitaxel:Ldose: 0 (uM) Interaction | -51.98663952 | 19.0858749 | -2.723827951 | 0.006458212 |
| SKMEL2:Cabazitaxel:Ldose: 0 (uM) Interaction | -48.159087 | 19.0858749 | -2.523284223 | 0.011633573 |
| UACC0257:Cabazitaxel:Ldose: 0 (uM) Interaction | -46.0583302 | 19.0858749 | -2.413215556 | 0.015820758 |
| MeWo:Amifostine:Ldose: 0 (uM) Interaction | -64.28625146 | 19.0858749 | -3.368263274 | 0.000757758 |
| SKMEL2:Amifostine:Ldose: 0 (uM) Interaction | -51.97088143 | 19.0858749 | -2.72300231 | 0.006474369 |
| UACC0257:Amifostine:Ldose: 0 (uM) Interaction | -55.41375338 | 19.0858749 | -2.903390789 | 0.003695204 |
| MeWo:Flutamide..Eulexin.:Ldose: 0 (uM) Interaction | -57.89413919 | 19.0858749 | -3.03335003 | 0.002421428 |
| SKMEL2:Flutamide..Eulexin.:Ldose: 0 (uM) Interaction | -49.16421289 | 19.0858749 | -2.575947561 | 0.010003181 |
| UACC0257:Flutamide..Eulexin.:Ldose: 0 (uM) Interaction | -58.25082183 | 19.0858749 | -3.052038334 | 0.002275698 |
| MeWo:LDK378:Ldose: 0 (uM) Interaction | -51.1698986 | 19.0858749 | -2.681035 | 0.007345073 |
| SKMEL2:LDK378:Ldose: 0 (uM) Interaction | -39.54825204 | 19.0858749 | -2.072121517 | 0.03826598 |
| UACC0257:LDK378:Ldose: 0 (uM) Interaction | -41.58017302 | 19.0858749 | -2.178583547 | 0.029373423 |
| MeWo:Pralatrexate:Ldose: 0 (uM) Interaction | -52.40432028 | 19.0858749 | -2.745712238 | 0.006042978 |
| SKMEL2:Pralatrexate:Ldose: 0 (uM) Interaction | -47.82377396 | 19.0858749 | -2.505715573 | 0.012227704 |
| UACC0257:Pralatrexate:Ldose: 0 (uM) Interaction | -37.52844121 | 19.0858749 | -1.966293996 | 0.049277481 |
| MeWo:Topotecan.HCl:Ldose: 0 (uM) Interaction | 1.444941239 | 19.0858749 | 0.075707362 | 0.939652622 |
| SKMEL2:Topotecan.HCl:Ldose: 0 (uM) Interaction | 7.458392124 | 19.0858749 | 0.39078073 | 0.695963185 |
| UACC0257:Topotecan.HCl:Ldose: 0 (uM) Interaction | -16.70488924 | 19.0858749 | -0.875248807 | 0.381448301 |
| MeWo:Pemetrexed:Ldose: 0 (uM) Interaction | -55.58979994 | 19.0858749 | -2.912614708 | 0.003587845 |
| SKMEL2:Pemetrexed:Ldose: 0 (uM) Interaction | -47.18018532 | 19.0858749 | -2.471994895 | 0.013443812 |
| UACC0257:Pemetrexed:Ldose: 0 (uM) Interaction | -32.74874343 | 19.0858749 | -1.715862836 | 0.08620153 |
| MeWo:Bleomycin.Sulfate:Ldose: 0 (uM) Interaction | -35.37692123 | 19.0858749 | -1.853565604 | 0.063814996 |
| SKMEL2:Bleomycin.Sulfate:Ldose: 0 (uM) Interaction | -29.87606034 | 19.0858749 | -1.56534927 | 0.117515726 |
| UACC0257:Bleomycin.Sulfate:Ldose: 0 (uM) Interaction | 1.798273691 | 19.0858749 | 0.094220134 | 0.924935168 |
| MeWo:Axitinib.1:Ldose: 0 (uM) Interaction | -50.64130898 | 19.0858749 | -2.653339669 | 0.00797581 |
| SKMEL2:Axitinib.1:Ldose: 0 (uM) Interaction | -40.30485999 | 19.0858749 | -2.111763815 | 0.034718196 |
| UACC0257:Axitinib.1:Ldose: 0 (uM) Interaction | -36.32254354 | 19.0858749 | -1.903111266 | 0.057039346 |
| MeWo:Ibrutinib..PCI.32765.:Ldose: 0 (uM) Interaction | -59.99960788 | 19.0858749 | -3.143665575 | 0.001670725 |
| SKMEL2:Ibrutinib..PCI.32765.:Ldose: 0 (uM) Interaction | -50.4161547 | 19.0858749 | -2.641542763 | 0.008258899 |
| UACC0257:Ibrutinib..PCI.32765.:Ldose: 0 (uM) Interaction | -37.84469524 | 19.0858749 | -1.982864053 | 0.047395298 |
| MeWo:Tamoxifen.Citrate:Ldose: 0 (uM) Interaction | -56.42317026 | 19.0858749 | -2.956278953 | 0.003117128 |
| SKMEL2:Tamoxifen.Citrate:Ldose: 0 (uM) Interaction | -46.68785653 | 19.0858749 | -2.446199442 | 0.014445052 |
| UACC0257:Tamoxifen.Citrate:Ldose: 0 (uM) Interaction | -36.65416711 | 19.0858749 | -1.920486606 | 0.054809651 |
| MeWo:Vemurafenib:Ldose: 0 (uM) Interaction | 6.982647648 | 19.0858749 | 0.365854208 | 0.71447746 |
| SKMEL2:Vemurafenib:Ldose: 0 (uM) Interaction | 6.934818576 | 19.0858749 | 0.363348215 | 0.716348346 |
| UACC0257:Vemurafenib:Ldose: 0 (uM) Interaction | -10.3296649 | 19.0858749 | -0.541220403 | 0.588361273 |
| MeWo:Pazopanib.HCl:Ldose: 0 (uM) Interaction | -48.82283105 | 19.0858749 | -2.558060939 | 0.010532545 |
| SKMEL2:Pazopanib.HCl:Ldose: 0 (uM) Interaction | -47.00303394 | 19.0858749 | -2.46271309 | 0.013796787 |
| UACC0257:Pazopanib.HCl:Ldose: 0 (uM) Interaction | -28.52947609 | 19.0858749 | -1.4947953 | 0.134982522 |
| MeWo:Abiraterone:Ldose: 0 (uM) Interaction | -59.63404824 | 19.0858749 | -3.124512162 | 0.001783365 |
| SKMEL2:Abiraterone:Ldose: 0 (uM) Interaction | -56.67662514 | 19.0858749 | -2.969558663 | 0.002985564 |
| UACC0257:Abiraterone:Ldose: 0 (uM) Interaction | -44.06816683 | 19.0858749 | -2.308941407 | 0.02095625 |
| MeWo:Bosutinib..SKI.606.:Ldose: 0 (uM) Interaction | -38.52416248 | 19.0858749 | -2.018464581 | 0.043555268 |
| SKMEL2:Bosutinib..SKI.606.:Ldose: 0 (uM) Interaction | -27.72142564 | 19.0858749 | -1.452457684 | 0.146388953 |
| UACC0257:Bosutinib..SKI.606.:Ldose: 0 (uM) Interaction | -26.21887114 | 19.0858749 | -1.373731688 | 0.169539291 |
| MeWo:Sabutoclax..BI.97C1.:Ldose: 0 (uM) Interaction | -52.66413288 | 19.0858749 | -2.759325059 | 0.00579698 |
| SKMEL2:Sabutoclax..BI.97C1.:Ldose: 0 (uM) Interaction | -31.27965563 | 19.0858749 | -1.638890321 | 0.101250708 |
| UACC0257:Sabutoclax..BI.97C1.:Ldose: 0 (uM) Interaction | -1.129970601 | 19.0858749 | -0.059204548 | 0.952789736 |
| MeWo:Thioguanine:Ldose: 0.301029996 (uM) Interaction | -20.82063394 | 19.0858749 | -1.090892299 | 0.275332479 |
| SKMEL2:Thioguanine:Ldose: 0.301029996 (uM) Interaction | 25.33937609 | 19.0858749 | 1.327650749 | 0.184307581 |
| UACC0257:Thioguanine:Ldose: 0.301029996 (uM) Interaction | 20.64551336 | 19.0858749 | 1.081716896 | 0.279390453 |
| MeWo:Irinotecan.HCl:Ldose: 0.301029996 (uM) Interaction | -10.19264798 | 19.0858749 | -0.534041433 | 0.59331838 |
| SKMEL2:Irinotecan.HCl:Ldose: 0.301029996 (uM) Interaction | -8.184269327 | 19.0858749 | -0.428812898 | 0.66806368 |
| UACC0257:Irinotecan.HCl:Ldose: 0.301029996 (uM) Interaction | -1.676443275 | 19.0858749 | -0.087836858 | 0.930007159 |
| MeWo:Romidepsin:Ldose: 0.301029996 (uM) Interaction | -45.02575063 | 19.0858749 | -2.359113788 | 0.018327513 |
| SKMEL2:Romidepsin:Ldose: 0.301029996 (uM) Interaction | -30.87375802 | 19.0858749 | -1.617623409 | 0.105758423 |
| UACC0257:Romidepsin:Ldose: 0.301029996 (uM) Interaction | -27.04232075 | 19.0858749 | -1.41687614 | 0.156533606 |
| MeWo:Paclitaxel:Ldose: 0.301029996 (uM) Interaction | -10.17212739 | 19.0858749 | -0.532966261 | 0.594062434 |
| SKMEL2:Paclitaxel:Ldose: 0.301029996 (uM) Interaction | 20.97861444 | 19.0858749 | 1.099169651 | 0.271706359 |
| UACC0257:Paclitaxel:Ldose: 0.301029996 (uM) Interaction | -1.184141374 | 19.0858749 | -0.062042813 | 0.950529313 |
| MeWo:Alisertib..MLN8237.:Ldose: 0.301029996 (uM) Interaction | -36.19637166 | 19.0858749 | -1.896500519 | 0.057907258 |
| SKMEL2:Alisertib..MLN8237.:Ldose: 0.301029996 (uM) Interaction | -29.70680569 | 19.0858749 | -1.556481212 | 0.119608398 |
| UACC0257:Alisertib..MLN8237.:Ldose: 0.301029996 (uM) Interaction | -3.993863912 | 19.0858749 | -0.209257576 | 0.834249147 |
| MeWo:Vorinostat:Ldose: 0.301029996 (uM) Interaction | -59.37076342 | 19.0858749 | -3.110717415 | 0.001868771 |
| SKMEL2:Vorinostat:Ldose: 0.301029996 (uM) Interaction | -46.52389049 | 19.0858749 | -2.43760848 | 0.01479281 |
| UACC0257:Vorinostat:Ldose: 0.301029996 (uM) Interaction | -13.82327995 | 19.0858749 | -0.724267555 | 0.468909358 |
| MeWo:Busulfan:Ldose: 0.301029996 (uM) Interaction | -53.08142504 | 19.0858749 | -2.781188986 | 0.005420725 |
| SKMEL2:Busulfan:Ldose: 0.301029996 (uM) Interaction | -34.49123841 | 19.0858749 | -1.807160458 | 0.070751211 |
| UACC0257:Busulfan:Ldose: 0.301029996 (uM) Interaction | -8.452561344 | 19.0858749 | -0.442869996 | 0.657864218 |
| MeWo:Mechlorethamine.HCl:Ldose: 0.301029996 (uM) Interaction | -56.08194498 | 19.0858749 | -2.938400533 | 0.003302603 |
| SKMEL2:Mechlorethamine.HCl:Ldose: 0.301029996 (uM) Interaction | -37.04318033 | 19.0858749 | -1.940868864 | 0.052287214 |
| UACC0257:Mechlorethamine.HCl:Ldose: 0.301029996 (uM) Interaction | -20.47362373 | 19.0858749 | -1.072710779 | 0.28341292 |
| MeWo:Teniposide:Ldose: 0.301029996 (uM) Interaction | -54.50418582 | 19.0858749 | -2.855734207 | 0.004297848 |
| SKMEL2:Teniposide:Ldose: 0.301029996 (uM) Interaction | -45.79819264 | 19.0858749 | -2.399585709 | 0.016422076 |
| UACC0257:Teniposide:Ldose: 0.301029996 (uM) Interaction | -12.16947772 | 19.0858749 | -0.63761697 | 0.523729824 |
| MeWo:Vinorelbine.Tartrate:Ldose: 0.301029996 (uM) Interaction | -26.44571296 | 19.0858749 | -1.385617013 | 0.165878227 |
| SKMEL2:Vinorelbine.Tartrate:Ldose: 0.301029996 (uM) Interaction | -12.09117479 | 19.0858749 | -0.633514306 | 0.526404568 |
| UACC0257:Vinorelbine.Tartrate:Ldose: 0.301029996 (uM) Interaction | 3.522825103 | 19.0858749 | 0.184577606 | 0.853562089 |
| MeWo:Cabozantinib..XL.184.:Ldose: 0.301029996 (uM) Interaction | -45.84346061 | 19.0858749 | -2.401957514 | 0.016316016 |
| SKMEL2:Cabozantinib..XL.184.:Ldose: 0.301029996 (uM) Interaction | -16.74770412 | 19.0858749 | -0.877492083 | 0.380229204 |
| UACC0257:Cabozantinib..XL.184.:Ldose: 0.301029996 (uM) Interaction | -16.96673369 | 19.0858749 | -0.888968087 | 0.374030159 |
| MeWo:Dacarbazine:Ldose: 0.301029996 (uM) Interaction | -39.17365288 | 19.0858749 | -2.052494481 | 0.040133687 |
| SKMEL2:Dacarbazine:Ldose: 0.301029996 (uM) Interaction | -20.41164659 | 19.0858749 | -1.069463501 | 0.284872851 |
| UACC0257:Dacarbazine:Ldose: 0.301029996 (uM) Interaction | -17.49348984 | 19.0858749 | -0.916567353 | 0.35937966 |
| MeWo:Clofarabine:Ldose: 0.301029996 (uM) Interaction | -5.588833067 | 19.0858749 | -0.292825616 | 0.769658268 |
| SKMEL2:Clofarabine:Ldose: 0.301029996 (uM) Interaction | -4.416459119 | 19.0858749 | -0.231399354 | 0.817006765 |
| UACC0257:Clofarabine:Ldose: 0.301029996 (uM) Interaction | -23.69172771 | 19.0858749 | -1.241322593 | 0.214500119 |
| MeWo:Cisplatin:Ldose: 0.301029996 (uM) Interaction | -44.86214673 | 19.0858749 | -2.3505418 | 0.018755069 |
| SKMEL2:Cisplatin:Ldose: 0.301029996 (uM) Interaction | -25.26499036 | 19.0858749 | -1.323753325 | 0.18559903 |
| UACC0257:Cisplatin:Ldose: 0.301029996 (uM) Interaction | -26.8222035 | 19.0858749 | -1.405343148 | 0.159933686 |
| MeWo:Floxuridine:Ldose: 0.301029996 (uM) Interaction | -34.1347302 | 19.0858749 | -1.788481292 | 0.07371244 |
| SKMEL2:Floxuridine:Ldose: 0.301029996 (uM) Interaction | 26.46544956 | 19.0858749 | 1.386651107 | 0.165562527 |
| UACC0257:Floxuridine:Ldose: 0.301029996 (uM) Interaction | -22.96783893 | 19.0858749 | -1.203394607 | 0.228836879 |
| MeWo:Lomustine..CCNU.:Ldose: 0.301029996 (uM) Interaction | -59.72846907 | 19.0858749 | -3.12945932 | 0.001753621 |
| SKMEL2:Lomustine..CCNU.:Ldose: 0.301029996 (uM) Interaction | -41.63279201 | 19.0858749 | -2.181340506 | 0.029169016 |
| UACC0257:Lomustine..CCNU.:Ldose: 0.301029996 (uM) Interaction | -16.68176407 | 19.0858749 | -0.87403717 | 0.382107756 |
| MeWo:Melphalan:Ldose: 0.301029996 (uM) Interaction | -53.06018492 | 19.0858749 | -2.780076114 | 0.00543933 |
| SKMEL2:Melphalan:Ldose: 0.301029996 (uM) Interaction | -39.91241996 | 19.0858749 | -2.091202011 | 0.036521645 |
| UACC0257:Melphalan:Ldose: 0.301029996 (uM) Interaction | -9.2796635 | 19.0858749 | -0.486205822 | 0.62682619 |
| MeWo:BGJ398..NVPBGJ398.:Ldose: 0.301029996 (uM) Interaction | -44.03893848 | 19.0858749 | -2.307409994 | 0.021041408 |
| SKMEL2:BGJ398..NVPBGJ398.:Ldose: 0.301029996 (uM) Interaction | -26.72531256 | 19.0858749 | -1.400266569 | 0.161447912 |
| UACC0257:BGJ398..NVPBGJ398.:Ldose: 0.301029996 (uM) Interaction | -24.18988369 | 19.0858749 | -1.26742336 | 0.205017653 |
| MeWo:Navitoclax..ABT.263..5uM:Ldose: 0.301029996 (uM) Interaction | -57.35155372 | 19.0858749 | -3.00492139 | 0.002659551 |
| SKMEL2:Navitoclax..ABT.263..5uM:Ldose: 0.301029996 (uM) Interaction | -26.73622157 | 19.0858749 | -1.400838144 | 0.161276885 |
| UACC0257:Navitoclax..ABT.263..5uM:Ldose: 0.301029996 (uM) Interaction | -11.85748297 | 19.0858749 | -0.621270077 | 0.534428513 |
| MeWo:Azacitidine:Ldose: 0.301029996 (uM) Interaction | -48.03929746 | 19.0858749 | -2.517007877 | 0.011842818 |
| SKMEL2:Azacitidine:Ldose: 0.301029996 (uM) Interaction | -50.16364962 | 19.0858749 | -2.628312817 | 0.008587043 |
| UACC0257:Azacitidine:Ldose: 0.301029996 (uM) Interaction | -8.850014329 | 19.0858749 | -0.463694453 | 0.642871357 |
| MeWo:Capecitabine:Ldose: 0.301029996 (uM) Interaction | -56.14853079 | 19.0858749 | -2.941889282 | 0.003265639 |
| SKMEL2:Capecitabine:Ldose: 0.301029996 (uM) Interaction | -42.60875076 | 19.0858749 | -2.232475639 | 0.025593781 |
| UACC0257:Capecitabine:Ldose: 0.301029996 (uM) Interaction | -24.00087188 | 19.0858749 | -1.25752013 | 0.208578988 |
| MeWo:Megestrol.acetate:Ldose: 0.301029996 (uM) Interaction | -35.44378792 | 19.0858749 | -1.857069069 | 0.063314968 |
| SKMEL2:Megestrol.acetate:Ldose: 0.301029996 (uM) Interaction | -22.19875129 | 19.0858749 | -1.163098438 | 0.24480245 |
| UACC0257:Megestrol.acetate:Ldose: 0.301029996 (uM) Interaction | -25.24393028 | 19.0858749 | -1.322649887 | 0.185965878 |
| MeWo:Cytarabine.HCl...Ara.C:Ldose: 0.301029996 (uM) Interaction | 11.65701195 | 19.0858749 | 0.610766444 | 0.541360655 |
| SKMEL2:Cytarabine.HCl...Ara.C:Ldose: 0.301029996 (uM) Interaction | 25.74260477 | 19.0858749 | 1.348777822 | 0.17742252 |
| UACC0257:Cytarabine.HCl...Ara.C:Ldose: 0.301029996 (uM) Interaction | 15.95756006 | 19.0858749 | 0.836092668 | 0.403112048 |
| MeWo:Gemcitabine.HCl:Ldose: 0.301029996 (uM) Interaction | -19.61551972 | 19.0858749 | -1.027750618 | 0.304078691 |
| SKMEL2:Gemcitabine.HCl:Ldose: 0.301029996 (uM) Interaction | 31.76365102 | 19.0858749 | 1.664249147 | 0.096077195 |
| UACC0257:Gemcitabine.HCl:Ldose: 0.301029996 (uM) Interaction | -35.9599863 | 19.0858749 | -1.884115164 | 0.059562855 |
| MeWo:Vinblastine.Sulfate:Ldose: 0.301029996 (uM) Interaction | -55.37897987 | 19.0858749 | -2.901568839 | 0.003716752 |
| SKMEL2:Vinblastine.Sulfate:Ldose: 0.301029996 (uM) Interaction | -30.48855746 | 19.0858749 | -1.597440915 | 0.110182127 |
| UACC0257:Vinblastine.Sulfate:Ldose: 0.301029996 (uM) Interaction | 7.136458316 | 19.0858749 | 0.373913083 | 0.708472663 |
| MeWo:MLN9708..MLN2238.:Ldose: 0.301029996 (uM) Interaction | -68.28427953 | 19.0858749 | -3.577739029 | 0.000347339 |
| SKMEL2:MLN9708..MLN2238.:Ldose: 0.301029996 (uM) Interaction | -16.66351241 | 19.0858749 | -0.873080878 | 0.382628728 |
| UACC0257:MLN9708..MLN2238.:Ldose: 0.301029996 (uM) Interaction | -27.43522394 | 19.0858749 | -1.437462212 | 0.150601229 |
| MeWo:ABT.737:Ldose: 0.301029996 (uM) Interaction | -57.72550628 | 19.0858749 | -3.024514548 | 0.002493259 |
| SKMEL2:ABT.737:Ldose: 0.301029996 (uM) Interaction | -32.80512758 | 19.0858749 | -1.718817071 | 0.085662055 |
| UACC0257:ABT.737:Ldose: 0.301029996 (uM) Interaction | -24.00897393 | 19.0858749 | -1.257944635 | 0.208425416 |
| MeWo:Streptozocin:Ldose: 0.301029996 (uM) Interaction | -46.89960407 | 19.0858749 | -2.457293906 | 0.014006634 |
| SKMEL2:Streptozocin:Ldose: 0.301029996 (uM) Interaction | -51.01823516 | 19.0858749 | -2.673088629 | 0.007521302 |
| UACC0257:Streptozocin:Ldose: 0.301029996 (uM) Interaction | -9.511780607 | 19.0858749 | -0.498367545 | 0.618230097 |
| MeWo:Crizotinib:Ldose: 0.301029996 (uM) Interaction | -52.19067666 | 19.0858749 | -2.73451843 | 0.006252266 |
| SKMEL2:Crizotinib:Ldose: 0.301029996 (uM) Interaction | -38.08878981 | 19.0858749 | -1.995653331 | 0.045984241 |
| UACC0257:Crizotinib:Ldose: 0.301029996 (uM) Interaction | -34.89665797 | 19.0858749 | -1.828402321 | 0.067503015 |
| MeWo:Sunitinib:Ldose: 0.301029996 (uM) Interaction | -59.48637457 | 19.0858749 | -3.116774834 | 0.001830816 |
| SKMEL2:Sunitinib:Ldose: 0.301029996 (uM) Interaction | -40.8662537 | 19.0858749 | -2.141177909 | 0.032270882 |
| UACC0257:Sunitinib:Ldose: 0.301029996 (uM) Interaction | -39.41734309 | 19.0858749 | -2.065262573 | 0.038910098 |
| MeWo:Dexrazoxane:Ldose: 0.301029996 (uM) Interaction | -56.56577472 | 19.0858749 | -2.963750681 | 0.003042469 |
| SKMEL2:Dexrazoxane:Ldose: 0.301029996 (uM) Interaction | -40.00649739 | 19.0858749 | -2.096131176 | 0.036082194 |
| UACC0257:Dexrazoxane:Ldose: 0.301029996 (uM) Interaction | -18.98986782 | 19.0858749 | -0.994969731 | 0.319762163 |
| MeWo:Mitomycin.C:Ldose: 0.301029996 (uM) Interaction | -32.19333725 | 19.0858749 | -1.686762458 | 0.091663517 |
| SKMEL2:Mitomycin.C:Ldose: 0.301029996 (uM) Interaction | -23.31514077 | 19.0858749 | -1.221591407 | 0.221875549 |
| UACC0257:Mitomycin.C:Ldose: 0.301029996 (uM) Interaction | -38.96410538 | 19.0858749 | -2.041515288 | 0.041211794 |
| MeWo:Carfilzomib:Ldose: 0.301029996 (uM) Interaction | -86.75517626 | 19.0858749 | -4.545517391 | 5.51E-06 |
| SKMEL2:Carfilzomib:Ldose: 0.301029996 (uM) Interaction | -1.473726603 | 19.0858749 | -0.077215564 | 0.938452777 |
| UACC0257:Carfilzomib:Ldose: 0.301029996 (uM) Interaction | -63.11876849 | 19.0858749 | -3.307093274 | 0.000944238 |
| MeWo:OSI.027:Ldose: 0.301029996 (uM) Interaction | -91.12745574 | 19.0858749 | -4.774601963 | 1.81E-06 |
| SKMEL2:OSI.027:Ldose: 0.301029996 (uM) Interaction | -24.53627853 | 19.0858749 | -1.285572637 | 0.198606072 |
| UACC0257:OSI.027:Ldose: 0.301029996 (uM) Interaction | -54.61886207 | 19.0858749 | -2.861742643 | 0.004217247 |
| MeWo:Bioymifi:Ldose: 0.301029996 (uM) Interaction | -55.40325603 | 19.0858749 | -2.902840783 | 0.003701697 |
| SKMEL2:Bioymifi:Ldose: 0.301029996 (uM) Interaction | -25.12237673 | 19.0858749 | -1.316281117 | 0.188093713 |
| UACC0257:Bioymifi:Ldose: 0.301029996 (uM) Interaction | -16.7954038 | 19.0858749 | -0.879991297 | 0.378873842 |
| MeWo:Nelarabine:Ldose: 0.301029996 (uM) Interaction | -48.49674808 | 19.0858749 | -2.540975897 | 0.011061307 |
| SKMEL2:Nelarabine:Ldose: 0.301029996 (uM) Interaction | -51.9076283 | 19.0858749 | -2.719688177 | 0.006539588 |
| UACC0257:Nelarabine:Ldose: 0.301029996 (uM) Interaction | -6.860722475 | 19.0858749 | -0.359465967 | 0.719250057 |
| MeWo:Raloxifene:Ldose: 0.301029996 (uM) Interaction | -51.29178982 | 19.0858749 | -2.687421462 | 0.007206134 |
| SKMEL2:Raloxifene:Ldose: 0.301029996 (uM) Interaction | -37.48308246 | 19.0858749 | -1.963917434 | 0.049552509 |
| UACC0257:Raloxifene:Ldose: 0.301029996 (uM) Interaction | -32.91884977 | 19.0858749 | -1.724775519 | 0.08458228 |
| MeWo:Quinacrine.HCl:Ldose: 0.301029996 (uM) Interaction | -68.67316202 | 19.0858749 | -3.598114438 | 0.000321251 |
| SKMEL2:Quinacrine.HCl:Ldose: 0.301029996 (uM) Interaction | -52.40225757 | 19.0858749 | -2.745604163 | 0.006044969 |
| UACC0257:Quinacrine.HCl:Ldose: 0.301029996 (uM) Interaction | -40.33543117 | 19.0858749 | -2.113365585 | 0.034580957 |
| MeWo:Lenalidomide:Ldose: 0.301029996 (uM) Interaction | -46.00224323 | 19.0858749 | -2.410276892 | 0.015948742 |
| SKMEL2:Lenalidomide:Ldose: 0.301029996 (uM) Interaction | -37.06330007 | 19.0858749 | -1.941923033 | 0.052159439 |
| UACC0257:Lenalidomide:Ldose: 0.301029996 (uM) Interaction | -22.1124725 | 19.0858749 | -1.158577881 | 0.246641134 |
| MeWo:Fludarabine.Phosphate:Ldose: 0.301029996 (uM) Interaction | -48.31719632 | 19.0858749 | -2.531568324 | 0.011362419 |
| SKMEL2:Fludarabine.Phosphate:Ldose: 0.301029996 (uM) Interaction | -22.2680861 | 19.0858749 | -1.16673122 | 0.243331847 |
| UACC0257:Fludarabine.Phosphate:Ldose: 0.301029996 (uM) Interaction | -4.119487454 | 19.0858749 | -0.215839592 | 0.829114829 |
| MeWo:Nilotinib:Ldose: 0.301029996 (uM) Interaction | -62.35150932 | 19.0858749 | -3.266892907 | 0.001089049 |
| SKMEL2:Nilotinib:Ldose: 0.301029996 (uM) Interaction | -38.7374316 | 19.0858749 | -2.029638768 | 0.042405539 |
| UACC0257:Nilotinib:Ldose: 0.301029996 (uM) Interaction | -35.47584377 | 19.0858749 | -1.858748627 | 0.063076406 |
| MeWo:Linsitinib:Ldose: 0.301029996 (uM) Interaction | -45.81599447 | 19.0858749 | -2.400518432 | 0.016380295 |
| SKMEL2:Linsitinib:Ldose: 0.301029996 (uM) Interaction | -29.78026346 | 19.0858749 | -1.560330015 | 0.118696605 |
| UACC0257:Linsitinib:Ldose: 0.301029996 (uM) Interaction | -31.11457914 | 19.0858749 | -1.630241176 | 0.103065156 |
| MeWo:Aphrocallistin.analogue:Ldose: 0.301029996 (uM) Interaction | -20.43698572 | 19.0858749 | -1.070791139 | 0.28427535 |
| SKMEL2:Aphrocallistin.analogue:Ldose: 0.301029996 (uM) Interaction | -3.960909011 | 19.0858749 | -0.207530911 | 0.835597216 |
| UACC0257:Aphrocallistin.analogue:Ldose: 0.301029996 (uM) Interaction | 24.61385503 | 19.0858749 | 1.28963724 | 0.197190486 |
| MeWo:Mitotane..o.p..DDD..Lysodren.:Ldose: 0.301029996 (uM) Interaction | -35.99828561 | 19.0858749 | -1.886121847 | 0.059291978 |
| SKMEL2:Mitotane..o.p..DDD..Lysodren.:Ldose: 0.301029996 (uM) Interaction | -43.60845277 | 19.0858749 | -2.284854795 | 0.02233102 |
| UACC0257:Mitotane..o.p..DDD..Lysodren.:Ldose: 0.301029996 (uM) Interaction | -9.585939287 | 19.0858749 | -0.502253071 | 0.61549465 |
| MeWo:Etoposide:Ldose: 0.301029996 (uM) Interaction | -14.64777816 | 19.0858749 | -0.767466948 | 0.442812349 |
| SKMEL2:Etoposide:Ldose: 0.301029996 (uM) Interaction | 5.631987363 | 19.0858749 | 0.295086675 | 0.767930513 |
| UACC0257:Etoposide:Ldose: 0.301029996 (uM) Interaction | 10.60861266 | 19.0858749 | 0.555835806 | 0.578328879 |
| MeWo:Vandetanib:Ldose: 0.301029996 (uM) Interaction | -67.90957845 | 19.0858749 | -3.558106653 | 0.00037434 |
| SKMEL2:Vandetanib:Ldose: 0.301029996 (uM) Interaction | -46.0444784 | 19.0858749 | -2.412489794 | 0.015852282 |
| UACC0257:Vandetanib:Ldose: 0.301029996 (uM) Interaction | -32.23315902 | 19.0858749 | -1.68884891 | 0.091262862 |
| MeWo:Carboplatin:Ldose: 0.301029996 (uM) Interaction | -64.97189339 | 19.0858749 | -3.404187324 | 0.000664821 |
| SKMEL2:Carboplatin:Ldose: 0.301029996 (uM) Interaction | -49.18369644 | 19.0858749 | -2.576968397 | 0.009973697 |
| UACC0257:Carboplatin:Ldose: 0.301029996 (uM) Interaction | -41.35861133 | 19.0858749 | -2.166974873 | 0.030247694 |
| MeWo:Gefitinib:Ldose: 0.301029996 (uM) Interaction | -62.71714292 | 19.0858749 | -3.286050195 | 0.001017653 |
| SKMEL2:Gefitinib:Ldose: 0.301029996 (uM) Interaction | -50.53372564 | 19.0858749 | -2.647702865 | 0.008109973 |
| UACC0257:Gefitinib:Ldose: 0.301029996 (uM) Interaction | -12.9511179 | 19.0858749 | -0.678570827 | 0.497417123 |
| MeWo:Vincristine.Sulfate:Ldose: 0.301029996 (uM) Interaction | -20.9779821 | 19.0858749 | -1.099136519 | 0.271720808 |
| SKMEL2:Vincristine.Sulfate:Ldose: 0.301029996 (uM) Interaction | 14.1139862 | 19.0858749 | 0.739499042 | 0.45961208 |
| UACC0257:Vincristine.Sulfate:Ldose: 0.301029996 (uM) Interaction | 26.1387174 | 19.0858749 | 1.369532051 | 0.1708473 |
| MeWo:Trametinib..GSK1120212.:Ldose: 0.301029996 (uM) Interaction | -34.59984278 | 19.0858749 | -1.812850758 | 0.069868757 |
| SKMEL2:Trametinib..GSK1120212.:Ldose: 0.301029996 (uM) Interaction | -22.19679609 | 19.0858749 | -1.162995996 | 0.244844011 |
| UACC0257:Trametinib..GSK1120212.:Ldose: 0.301029996 (uM) Interaction | -16.89643545 | 19.0858749 | -0.885284827 | 0.376012914 |
| MeWo:MLN4924:Ldose: 0.301029996 (uM) Interaction | -30.32052249 | 19.0858749 | -1.588636762 | 0.1121571 |
| SKMEL2:MLN4924:Ldose: 0.301029996 (uM) Interaction | -1.728363332 | 19.0858749 | -0.090557197 | 0.927845286 |
| UACC0257:MLN4924:Ldose: 0.301029996 (uM) Interaction | 17.2373545 | 19.0858749 | 0.903147202 | 0.366457875 |
| MeWo:Bortezomib:Ldose: 0.301029996 (uM) Interaction | -44.59634483 | 19.0858749 | -2.336615171 | 0.019468321 |
| SKMEL2:Bortezomib:Ldose: 0.301029996 (uM) Interaction | -25.90281641 | 19.0858749 | -1.357172074 | 0.174740762 |
| UACC0257:Bortezomib:Ldose: 0.301029996 (uM) Interaction | -4.053245344 | 19.0858749 | -0.212368852 | 0.831821293 |
| MeWo:Fluorouracil...5.FU.:Ldose: 0.301029996 (uM) Interaction | -65.51565189 | 19.0858749 | -3.432677425 | 0.000598776 |
| SKMEL2:Fluorouracil...5.FU.:Ldose: 0.301029996 (uM) Interaction | -39.28131315 | 19.0858749 | -2.058135315 | 0.039589149 |
| UACC0257:Fluorouracil...5.FU.:Ldose: 0.301029996 (uM) Interaction | -25.42417766 | 19.0858749 | -1.332093907 | 0.182843426 |
| MeWo:Lapatinib:Ldose: 0.301029996 (uM) Interaction | -52.72297086 | 19.0858749 | -2.762407861 | 0.00574254 |
| SKMEL2:Lapatinib:Ldose: 0.301029996 (uM) Interaction | -39.4791321 | 19.0858749 | -2.068499994 | 0.038604936 |
| UACC0257:Lapatinib:Ldose: 0.301029996 (uM) Interaction | -21.06412632 | 19.0858749 | -1.103650026 | 0.269757311 |
| MeWo:Mitoxantrone:Ldose: 0.301029996 (uM) Interaction | -27.31595409 | 19.0858749 | -1.431213095 | 0.152383659 |
| SKMEL2:Mitoxantrone:Ldose: 0.301029996 (uM) Interaction | 9.240410893 | 19.0858749 | 0.484149191 | 0.628284915 |
| UACC0257:Mitoxantrone:Ldose: 0.301029996 (uM) Interaction | 2.730719523 | 19.0858749 | 0.143075418 | 0.886231957 |
| MeWo:Imatinib:Ldose: 0.301029996 (uM) Interaction | -49.73873962 | 19.0858749 | -2.606049756 | 0.009165618 |
| SKMEL2:Imatinib:Ldose: 0.301029996 (uM) Interaction | -38.11516835 | 19.0858749 | -1.997035428 | 0.045833893 |
| UACC0257:Imatinib:Ldose: 0.301029996 (uM) Interaction | -9.827319774 | 19.0858749 | -0.514900146 | 0.606628082 |
| MeWo:Imiquimod:Ldose: 0.301029996 (uM) Interaction | -45.99623615 | 19.0858749 | -2.409962153 | 0.015962503 |
| SKMEL2:Imiquimod:Ldose: 0.301029996 (uM) Interaction | -27.7004277 | 19.0858749 | -1.451357501 | 0.146694901 |
| UACC0257:Imiquimod:Ldose: 0.301029996 (uM) Interaction | 6.173387528 | 19.0858749 | 0.323453211 | 0.746355177 |
| MeWo:Dacomitinib..PF299804.:Ldose: 0.301029996 (uM) Interaction | -51.19105673 | 19.0858749 | -2.682143575 | 0.007320785 |
| SKMEL2:Dacomitinib..PF299804.:Ldose: 0.301029996 (uM) Interaction | -33.51691603 | 19.0858749 | -1.756111062 | 0.079083657 |
| UACC0257:Dacomitinib..PF299804.:Ldose: 0.301029996 (uM) Interaction | -8.417069367 | 19.0858749 | -0.441010402 | 0.659209892 |
| MeWo:PD325901:Ldose: 0.301029996 (uM) Interaction | -85.04262931 | 19.0858749 | -4.45578889 | 8.40E-06 |
| SKMEL2:PD325901:Ldose: 0.301029996 (uM) Interaction | -76.19175002 | 19.0858749 | -3.992049116 | 6.57E-05 |
| UACC0257:PD325901:Ldose: 0.301029996 (uM) Interaction | -21.16350559 | 19.0858749 | -1.10885698 | 0.267504265 |
| MeWo:Vismodegib:Ldose: 0.301029996 (uM) Interaction | -37.11584512 | 19.0858749 | -1.944676119 | 0.05182697 |
| SKMEL2:Vismodegib:Ldose: 0.301029996 (uM) Interaction | -42.13583406 | 19.0858749 | -2.207697278 | 0.027275882 |
| UACC0257:Vismodegib:Ldose: 0.301029996 (uM) Interaction | -0.23755953 | 19.0858749 | -0.012446877 | 0.990069201 |
| MeWo:Temozolomide:Ldose: 0.301029996 (uM) Interaction | -57.27455307 | 19.0858749 | -3.000886958 | 0.002695026 |
| SKMEL2:Temozolomide:Ldose: 0.301029996 (uM) Interaction | -46.21303937 | 19.0858749 | -2.421321507 | 0.0154724 |
| UACC0257:Temozolomide:Ldose: 0.301029996 (uM) Interaction | -30.64372295 | 19.0858749 | -1.605570775 | 0.10838291 |
| MeWo:Mercaptopurine:Ldose: 0.301029996 (uM) Interaction | -62.80025793 | 19.0858749 | -3.290404987 | 0.00100204 |
| SKMEL2:Mercaptopurine:Ldose: 0.301029996 (uM) Interaction | -29.13789073 | 19.0858749 | -1.526673044 | 0.126857016 |
| UACC0257:Mercaptopurine:Ldose: 0.301029996 (uM) Interaction | -19.63745108 | 19.0858749 | -1.028899707 | 0.303538359 |
| MeWo:Dasatinib:Ldose: 0.301029996 (uM) Interaction | -61.37680019 | 19.0858749 | -3.215823248 | 0.001302629 |
| SKMEL2:Dasatinib:Ldose: 0.301029996 (uM) Interaction | -50.32892898 | 19.0858749 | -2.636972591 | 0.008370963 |
| UACC0257:Dasatinib:Ldose: 0.301029996 (uM) Interaction | -22.67853895 | 19.0858749 | -1.188236802 | 0.234753229 |
| MeWo:Daunorubicin.HCl:Ldose: 0.301029996 (uM) Interaction | -56.39880527 | 19.0858749 | -2.955002355 | 0.00313005 |
| SKMEL2:Daunorubicin.HCl:Ldose: 0.301029996 (uM) Interaction | -16.56199104 | 19.0858749 | -0.867761689 | 0.385534474 |
| UACC0257:Daunorubicin.HCl:Ldose: 0.301029996 (uM) Interaction | -39.93509928 | 19.0858749 | -2.092390289 | 0.036415291 |
| MeWo:Sirolimus..Rapamycin.:Ldose: 0.301029996 (uM) Interaction | -51.03022987 | 19.0858749 | -2.673717089 | 0.007507228 |
| SKMEL2:Sirolimus..Rapamycin.:Ldose: 0.301029996 (uM) Interaction | -47.22405091 | 19.0858749 | -2.474293223 | 0.013357652 |
| UACC0257:Sirolimus..Rapamycin.:Ldose: 0.301029996 (uM) Interaction | -19.18878264 | 19.0858749 | -1.005391827 | 0.314719507 |
| MeWo:INK.128..MLN0128.:Ldose: 0.301029996 (uM) Interaction | -25.25200988 | 19.0858749 | -1.323073216 | 0.185825075 |
| SKMEL2:INK.128..MLN0128.:Ldose: 0.301029996 (uM) Interaction | -36.30122476 | 19.0858749 | -1.901994274 | 0.05718523 |
| UACC0257:INK.128..MLN0128.:Ldose: 0.301029996 (uM) Interaction | -14.90267051 | 19.0858749 | -0.780821974 | 0.434915789 |
| MeWo:Quizartinib:Ldose: 0.301029996 (uM) Interaction | -57.68028918 | 19.0858749 | -3.022145408 | 0.002512848 |
| SKMEL2:Quizartinib:Ldose: 0.301029996 (uM) Interaction | -31.13250185 | 19.0858749 | -1.631180233 | 0.102866914 |
| UACC0257:Quizartinib:Ldose: 0.301029996 (uM) Interaction | -31.06050287 | 19.0858749 | -1.627407862 | 0.103665129 |
| MeWo:Sorafenib:Ldose: 0.301029996 (uM) Interaction | -48.57502457 | 19.0858749 | -2.545077175 | 0.010932271 |
| SKMEL2:Sorafenib:Ldose: 0.301029996 (uM) Interaction | -42.30357291 | 19.0858749 | -2.216485917 | 0.026668648 |
| UACC0257:Sorafenib:Ldose: 0.301029996 (uM) Interaction | -7.1768571 | 19.0858749 | -0.376029768 | 0.706898469 |
| MeWo:Carmustine:Ldose: 0.301029996 (uM) Interaction | -56.5430559 | 19.0858749 | -2.962560334 | 0.003054253 |
| SKMEL2:Carmustine:Ldose: 0.301029996 (uM) Interaction | -41.3102657 | 19.0858749 | -2.164441815 | 0.030441408 |
| UACC0257:Carmustine:Ldose: 0.301029996 (uM) Interaction | -33.06004417 | 19.0858749 | -1.732173366 | 0.083257015 |
| MeWo:Uracil.mustard:Ldose: 0.301029996 (uM) Interaction | -53.25239334 | 19.0858749 | -2.79014683 | 0.005273045 |
| SKMEL2:Uracil.mustard:Ldose: 0.301029996 (uM) Interaction | -39.39837394 | 19.0858749 | -2.064268689 | 0.039004193 |
| UACC0257:Uracil.mustard:Ldose: 0.301029996 (uM) Interaction | -37.09518117 | 19.0858749 | -1.943593436 | 0.051957505 |
| MeWo:Ixabepilone:Ldose: 0.301029996 (uM) Interaction | -25.55101372 | 19.0858749 | -1.338739453 | 0.180669636 |
| SKMEL2:Ixabepilone:Ldose: 0.301029996 (uM) Interaction | -17.17871382 | 19.0858749 | -0.900074737 | 0.368090542 |
| UACC0257:Ixabepilone:Ldose: 0.301029996 (uM) Interaction | -5.429167504 | 19.0858749 | -0.284459975 | 0.776060637 |
| MeWo:Valrubicin:Ldose: 0.301029996 (uM) Interaction | 2.923359783 | 19.0858749 | 0.15316876 | 0.878266628 |
| SKMEL2:Valrubicin:Ldose: 0.301029996 (uM) Interaction | 9.162942179 | 19.0858749 | 0.480090236 | 0.631168106 |
| UACC0257:Valrubicin:Ldose: 0.301029996 (uM) Interaction | 12.53702396 | 19.0858749 | 0.65687447 | 0.511268653 |
| MeWo:Triethylenemelamine:Ldose: 0.301029996 (uM) Interaction | -58.51740299 | 19.0858749 | -3.066005793 | 0.002172077 |
| SKMEL2:Triethylenemelamine:Ldose: 0.301029996 (uM) Interaction | -43.65455805 | 19.0858749 | -2.28727047 | 0.022189692 |
| UACC0257:Triethylenemelamine:Ldose: 0.301029996 (uM) Interaction | -15.40498979 | 19.0858749 | -0.807140876 | 0.419594236 |
| MeWo:Palbociclib..PD.0332991..Isethionate:Ldose: 0.301029996 (uM) Interaction | -32.8460574 | 19.0858749 | -1.720961579 | 0.085272156 |
| SKMEL2:Palbociclib..PD.0332991..Isethionate:Ldose: 0.301029996 (uM) Interaction | -14.05576589 | 19.0858749 | -0.736448602 | 0.461465756 |
| UACC0257:Palbociclib..PD.0332991..Isethionate:Ldose: 0.301029996 (uM) Interaction | -20.34098134 | 19.0858749 | -1.065761012 | 0.286543635 |
| MeWo:Afatinib:Ldose: 0.301029996 (uM) Interaction | -46.7036118 | 19.0858749 | -2.447024936 | 0.01441202 |
| SKMEL2:Afatinib:Ldose: 0.301029996 (uM) Interaction | -34.70835827 | 19.0858749 | -1.818536402 | 0.068996069 |
| UACC0257:Afatinib:Ldose: 0.301029996 (uM) Interaction | -23.48774755 | 19.0858749 | -1.2306351 | 0.21847281 |
| MeWo:Doxorubicin.HCl:Ldose: 0.301029996 (uM) Interaction | -52.30934321 | 19.0858749 | -2.740735937 | 0.006135227 |
| SKMEL2:Doxorubicin.HCl:Ldose: 0.301029996 (uM) Interaction | -48.72703854 | 19.0858749 | -2.553041912 | 0.010685495 |
| UACC0257:Doxorubicin.HCl:Ldose: 0.301029996 (uM) Interaction | -41.27489684 | 19.0858749 | -2.162588672 | 0.0305838 |
| MeWo:Exemestane:Ldose: 0.301029996 (uM) Interaction | -56.08334847 | 19.0858749 | -2.938474069 | 0.00330182 |
| SKMEL2:Exemestane:Ldose: 0.301029996 (uM) Interaction | -46.28868572 | 19.0858749 | -2.42528498 | 0.015304538 |
| UACC0257:Exemestane:Ldose: 0.301029996 (uM) Interaction | -37.3762669 | 19.0858749 | -1.958320858 | 0.050205262 |
| MeWo:Tretinoin:Ldose: 0.301029996 (uM) Interaction | -54.6595212 | 19.0858749 | -2.863872969 | 0.004189 |
| SKMEL2:Tretinoin:Ldose: 0.301029996 (uM) Interaction | -41.12554005 | 19.0858749 | -2.154763157 | 0.031191422 |
| UACC0257:Tretinoin:Ldose: 0.301029996 (uM) Interaction | -32.32307808 | 19.0858749 | -1.693560199 | 0.090363345 |
| MeWo:Fulvestrant:Ldose: 0.301029996 (uM) Interaction | -46.72022345 | 19.0858749 | -2.4478953 | 0.014377264 |
| SKMEL2:Fulvestrant:Ldose: 0.301029996 (uM) Interaction | -35.74921767 | 19.0858749 | -1.87307199 | 0.061071977 |
| UACC0257:Fulvestrant:Ldose: 0.301029996 (uM) Interaction | -6.776715284 | 19.0858749 | -0.35506443 | 0.7225448 |
| MeWo:Docetaxel:Ldose: 0.301029996 (uM) Interaction | -23.82730405 | 19.0858749 | -1.248426084 | 0.211888631 |
| SKMEL2:Docetaxel:Ldose: 0.301029996 (uM) Interaction | 5.976824034 | 19.0858749 | 0.313154313 | 0.754166459 |
| UACC0257:Docetaxel:Ldose: 0.301029996 (uM) Interaction | -5.013900742 | 19.0858749 | -0.26270217 | 0.792782657 |
| MeWo:Everolimus:Ldose: 0.301029996 (uM) Interaction | -28.20605394 | 19.0858749 | -1.477849671 | 0.139462641 |
| SKMEL2:Everolimus:Ldose: 0.301029996 (uM) Interaction | -22.09655965 | 19.0858749 | -1.157744131 | 0.246981306 |
| UACC0257:Everolimus:Ldose: 0.301029996 (uM) Interaction | -25.57614893 | 19.0858749 | -1.340056407 | 0.180241143 |
| MeWo:MLN.2480:Ldose: 0.301029996 (uM) Interaction | -19.48655137 | 19.0858749 | -1.020993351 | 0.307269056 |
| SKMEL2:MLN.2480:Ldose: 0.301029996 (uM) Interaction | 0.689449691 | 19.0858749 | 0.036123557 | 0.971184173 |
| UACC0257:MLN.2480:Ldose: 0.301029996 (uM) Interaction | -27.52270099 | 19.0858749 | -1.442045551 | 0.149304063 |
| MeWo:LY2157299:Ldose: 0.301029996 (uM) Interaction | -57.38254172 | 19.0858749 | -3.006544999 | 0.002645395 |
| SKMEL2:LY2157299:Ldose: 0.301029996 (uM) Interaction | -34.19371596 | 19.0858749 | -1.791571838 | 0.07321561 |
| UACC0257:LY2157299:Ldose: 0.301029996 (uM) Interaction | -15.20448703 | 19.0858749 | -0.796635581 | 0.425671487 |
| MeWo:Allopurinol:Ldose: 0.301029996 (uM) Interaction | -53.67394474 | 19.0858749 | -2.812233918 | 0.00492432 |
| SKMEL2:Allopurinol:Ldose: 0.301029996 (uM) Interaction | -48.26052531 | 19.0858749 | -2.528599059 | 0.011458957 |
| UACC0257:Allopurinol:Ldose: 0.301029996 (uM) Interaction | -20.16992413 | 19.0858749 | -1.056798509 | 0.290615407 |
| MeWo:Pipobroman:Ldose: 0.301029996 (uM) Interaction | -52.164087 | 19.0858749 | -2.733125271 | 0.006278765 |
| SKMEL2:Pipobroman:Ldose: 0.301029996 (uM) Interaction | -38.36756212 | 19.0858749 | -2.010259541 | 0.044416169 |
| UACC0257:Pipobroman:Ldose: 0.301029996 (uM) Interaction | -13.44457239 | 19.0858749 | -0.70442526 | 0.481175595 |
| MeWo:Letrozole:Ldose: 0.301029996 (uM) Interaction | -42.16111452 | 19.0858749 | -2.209021842 | 0.027183607 |
| SKMEL2:Letrozole:Ldose: 0.301029996 (uM) Interaction | -28.45100253 | 19.0858749 | -1.490683696 | 0.136059202 |
| UACC0257:Letrozole:Ldose: 0.301029996 (uM) Interaction | -29.28183065 | 19.0858749 | -1.534214743 | 0.124991519 |
| MeWo:Thiotepa:Ldose: 0.301029996 (uM) Interaction | -48.51278787 | 19.0858749 | -2.541816298 | 0.011034757 |
| SKMEL2:Thiotepa:Ldose: 0.301029996 (uM) Interaction | -36.63764266 | 19.0858749 | -1.919620812 | 0.054919008 |
| UACC0257:Thiotepa:Ldose: 0.301029996 (uM) Interaction | -4.768191607 | 19.0858749 | -0.249828296 | 0.802722519 |
| MeWo:Plicamycin:Ldose: 0.301029996 (uM) Interaction | -53.59321298 | 19.0858749 | -2.808003996 | 0.004989444 |
| SKMEL2:Plicamycin:Ldose: 0.301029996 (uM) Interaction | -36.15087271 | 19.0858749 | -1.894116613 | 0.058222917 |
| UACC0257:Plicamycin:Ldose: 0.301029996 (uM) Interaction | -27.20845948 | 19.0858749 | -1.42558094 | 0.154003832 |
| MeWo:Erlotinib.HCl:Ldose: 0.301029996 (uM) Interaction | -57.06282658 | 19.0858749 | -2.989793598 | 0.002794813 |
| SKMEL2:Erlotinib.HCl:Ldose: 0.301029996 (uM) Interaction | -35.35791815 | 19.0858749 | -1.852569942 | 0.063957694 |
| UACC0257:Erlotinib.HCl:Ldose: 0.301029996 (uM) Interaction | -29.41001242 | 19.0858749 | -1.540930797 | 0.123348319 |
| MeWo:MEK.162..ARRY.438162.:Ldose: 0.301029996 (uM) Interaction | -31.13031843 | 19.0858749 | -1.631065833 | 0.102891049 |
| SKMEL2:MEK.162..ARRY.438162.:Ldose: 0.301029996 (uM) Interaction | -11.27246631 | 19.0858749 | -0.590618264 | 0.554782421 |
| UACC0257:MEK.162..ARRY.438162.:Ldose: 0.301029996 (uM) Interaction | 6.876338961 | 19.0858749 | 0.360284189 | 0.718638154 |
| MeWo:Baricitinib..LY3009104..INCB028050.:Ldose: 0.301029996 (uM) Interaction | -38.63660485 | 19.0858749 | -2.024355973 | 0.042945854 |
| SKMEL2:Baricitinib..LY3009104..INCB028050.:Ldose: 0.301029996 (uM) Interaction | -17.61942328 | 19.0858749 | -0.923165607 | 0.35593125 |
| UACC0257:Baricitinib..LY3009104..INCB028050.:Ldose: 0.301029996 (uM) Interaction | -19.64747757 | 19.0858749 | -1.029425042 | 0.303291545 |
| MeWo:Arsenic.Trioxide:Ldose: 0.301029996 (uM) Interaction | -38.75061109 | 19.0858749 | -2.030329304 | 0.042335339 |
| SKMEL2:Arsenic.Trioxide:Ldose: 0.301029996 (uM) Interaction | -41.22194089 | 19.0858749 | -2.159814057 | 0.030798065 |
| UACC0257:Arsenic.Trioxide:Ldose: 0.301029996 (uM) Interaction | -14.35897399 | 19.0858749 | -0.75233512 | 0.451857774 |
| MeWo:Celecoxib:Ldose: 0.301029996 (uM) Interaction | -52.91422231 | 19.0858749 | -2.772428437 | 0.005568754 |
| SKMEL2:Celecoxib:Ldose: 0.301029996 (uM) Interaction | -38.51135259 | 19.0858749 | -2.01779341 | 0.043625156 |
| UACC0257:Celecoxib:Ldose: 0.301029996 (uM) Interaction | -13.73641729 | 19.0858749 | -0.719716406 | 0.471707431 |
| MeWo:Bendamustine.HCl:Ldose: 0.301029996 (uM) Interaction | -58.07082371 | 19.0858749 | -3.042607374 | 0.002348205 |
| SKMEL2:Bendamustine.HCl:Ldose: 0.301029996 (uM) Interaction | -36.95007393 | 19.0858749 | -1.935990576 | 0.052881922 |
| UACC0257:Bendamustine.HCl:Ldose: 0.301029996 (uM) Interaction | -40.65533214 | 19.0858749 | -2.130126723 | 0.033172453 |
| MeWo:Chlorambucil:Ldose: 0.301029996 (uM) Interaction | -47.99273493 | 19.0858749 | -2.514568244 | 0.011925048 |
| SKMEL2:Chlorambucil:Ldose: 0.301029996 (uM) Interaction | -29.91491523 | 19.0858749 | -1.567385063 | 0.117039403 |
| UACC0257:Chlorambucil:Ldose: 0.301029996 (uM) Interaction | -35.42535485 | 19.0858749 | -1.856103272 | 0.063452486 |
| MeWo:Zoledronic.Acid:Ldose: 0.301029996 (uM) Interaction | -43.44954333 | 19.0858749 | -2.276528772 | 0.022824147 |
| SKMEL2:Zoledronic.Acid:Ldose: 0.301029996 (uM) Interaction | -35.8131758 | 19.0858749 | -1.876423061 | 0.06061072 |
| UACC0257:Zoledronic.Acid:Ldose: 0.301029996 (uM) Interaction | -9.378346917 | 19.0858749 | -0.491376317 | 0.623165318 |
| MeWo:Actinomycin.D:Ldose: 0.301029996 (uM) Interaction | -61.50977162 | 19.0858749 | -3.222790255 | 0.001271373 |
| SKMEL2:Actinomycin.D:Ldose: 0.301029996 (uM) Interaction | -77.77850795 | 19.0858749 | -4.075186932 | 4.61E-05 |
| UACC0257:Actinomycin.D:Ldose: 0.301029996 (uM) Interaction | -47.02006305 | 19.0858749 | -2.463605326 | 0.013762504 |
| MeWo:Temsirolimus..CCI.779..Torisel.:Ldose: 0.301029996 (uM) Interaction | -55.67640674 | 19.0858749 | -2.917152452 | 0.003536078 |
| SKMEL2:Temsirolimus..CCI.779..Torisel.:Ldose: 0.301029996 (uM) Interaction | -41.99524504 | 19.0858749 | -2.200331149 | 0.027793986 |
| UACC0257:Temsirolimus..CCI.779..Torisel.:Ldose: 0.301029996 (uM) Interaction | -11.83781025 | 19.0858749 | -0.620239329 | 0.535106793 |
| MeWo:Foretinib..GSK1363089.:Ldose: 0.301029996 (uM) Interaction | -31.36736317 | 19.0858749 | -1.643485737 | 0.100297069 |
| SKMEL2:Foretinib..GSK1363089.:Ldose: 0.301029996 (uM) Interaction | 17.12052356 | 19.0858749 | 0.897025871 | 0.369715137 |
| UACC0257:Foretinib..GSK1363089.:Ldose: 0.301029996 (uM) Interaction | -16.77303758 | 19.0858749 | -0.878819424 | 0.379508996 |
| MeWo:Decitabine:Ldose: 0.301029996 (uM) Interaction | -50.64691848 | 19.0858749 | -2.653633577 | 0.00796887 |
| SKMEL2:Decitabine:Ldose: 0.301029996 (uM) Interaction | -38.7863036 | 19.0858749 | -2.032199405 | 0.042145717 |
| UACC0257:Decitabine:Ldose: 0.301029996 (uM) Interaction | -2.615629327 | 19.0858749 | -0.137045294 | 0.890996275 |
| MeWo:Methotrexate:Ldose: 0.301029996 (uM) Interaction | -51.873398 | 19.0858749 | -2.717894688 | 0.006575129 |
| SKMEL2:Methotrexate:Ldose: 0.301029996 (uM) Interaction | -35.92849275 | 19.0858749 | -1.882465066 | 0.059786366 |
| UACC0257:Methotrexate:Ldose: 0.301029996 (uM) Interaction | -21.79372633 | 19.0858749 | -1.14187725 | 0.25351774 |
| MeWo:Axitinib:Ldose: 0.301029996 (uM) Interaction | -52.66562399 | 19.0858749 | -2.759403186 | 0.005795594 |
| SKMEL2:Axitinib:Ldose: 0.301029996 (uM) Interaction | -40.32293542 | 19.0858749 | -2.112710873 | 0.034636996 |
| UACC0257:Axitinib:Ldose: 0.301029996 (uM) Interaction | -38.31585781 | 19.0858749 | -2.007550506 | 0.044703545 |
| MeWo:Oxaliplatin:Ldose: 0.301029996 (uM) Interaction | -54.88379133 | 19.0858749 | -2.87562355 | 0.004036256 |
| SKMEL2:Oxaliplatin:Ldose: 0.301029996 (uM) Interaction | -37.09336269 | 19.0858749 | -1.943498157 | 0.051969005 |
| UACC0257:Oxaliplatin:Ldose: 0.301029996 (uM) Interaction | -31.89890072 | 19.0858749 | -1.671335524 | 0.09466995 |
| MeWo:Cabazitaxel:Ldose: 0.301029996 (uM) Interaction | -47.66887411 | 19.0858749 | -2.497599632 | 0.012511133 |
| SKMEL2:Cabazitaxel:Ldose: 0.301029996 (uM) Interaction | -44.66907245 | 19.0858749 | -2.340425718 | 0.019270846 |
| UACC0257:Cabazitaxel:Ldose: 0.301029996 (uM) Interaction | -28.01824256 | 19.0858749 | -1.468009338 | 0.142116288 |
| MeWo:Amifostine:Ldose: 0.301029996 (uM) Interaction | -55.07690142 | 19.0858749 | -2.885741509 | 0.003908804 |
| SKMEL2:Amifostine:Ldose: 0.301029996 (uM) Interaction | -39.12589556 | 19.0858749 | -2.049992247 | 0.040377267 |
| UACC0257:Amifostine:Ldose: 0.301029996 (uM) Interaction | -24.77636663 | 19.0858749 | -1.298151998 | 0.194249009 |
| MeWo:Flutamide..Eulexin.:Ldose: 0.301029996 (uM) Interaction | -48.73627425 | 19.0858749 | -2.553525815 | 0.010670663 |
| SKMEL2:Flutamide..Eulexin.:Ldose: 0.301029996 (uM) Interaction | -29.59003797 | 19.0858749 | -1.550363194 | 0.121069059 |
| UACC0257:Flutamide..Eulexin.:Ldose: 0.301029996 (uM) Interaction | -15.00246207 | 19.0858749 | -0.786050529 | 0.431846532 |
| MeWo:LDK378:Ldose: 0.301029996 (uM) Interaction | -59.88749105 | 19.0858749 | -3.13779124 | 0.001704555 |
| SKMEL2:LDK378:Ldose: 0.301029996 (uM) Interaction | -35.10379231 | 19.0858749 | -1.839255077 | 0.065891432 |
| UACC0257:LDK378:Ldose: 0.301029996 (uM) Interaction | -18.01409977 | 19.0858749 | -0.94384459 | 0.345259657 |
| MeWo:Pralatrexate:Ldose: 0.301029996 (uM) Interaction | -46.50469127 | 19.0858749 | -2.436602541 | 0.014834008 |
| SKMEL2:Pralatrexate:Ldose: 0.301029996 (uM) Interaction | -37.90903157 | 19.0858749 | -1.986234939 | 0.047019896 |
| UACC0257:Pralatrexate:Ldose: 0.301029996 (uM) Interaction | -25.19155027 | 19.0858749 | -1.319905449 | 0.186880615 |
| MeWo:Topotecan.HCl:Ldose: 0.301029996 (uM) Interaction | -2.162957367 | 19.0858749 | -0.113327651 | 0.909771852 |
| SKMEL2:Topotecan.HCl:Ldose: 0.301029996 (uM) Interaction | 25.81730834 | 19.0858749 | 1.352691898 | 0.176168278 |
| UACC0257:Topotecan.HCl:Ldose: 0.301029996 (uM) Interaction | 14.34311912 | 19.0858749 | 0.751504408 | 0.452357359 |
| MeWo:Pemetrexed:Ldose: 0.301029996 (uM) Interaction | -43.3492402 | 19.0858749 | -2.271273412 | 0.023140254 |
| SKMEL2:Pemetrexed:Ldose: 0.301029996 (uM) Interaction | -30.61558417 | 19.0858749 | -1.60409645 | 0.108707455 |
| UACC0257:Pemetrexed:Ldose: 0.301029996 (uM) Interaction | -11.68133175 | 19.0858749 | -0.612040675 | 0.540517307 |
| MeWo:Bleomycin.Sulfate:Ldose: 0.301029996 (uM) Interaction | -24.60615222 | 19.0858749 | -1.289233653 | 0.197330713 |
| SKMEL2:Bleomycin.Sulfate:Ldose: 0.301029996 (uM) Interaction | -6.393556217 | 19.0858749 | -0.334988899 | 0.737636722 |
| UACC0257:Bleomycin.Sulfate:Ldose: 0.301029996 (uM) Interaction | 32.90384268 | 19.0858749 | 1.723989225 | 0.084724137 |
| MeWo:Axitinib.1:Ldose: 0.301029996 (uM) Interaction | -49.693606 | 19.0858749 | -2.603684991 | 0.009229074 |
| SKMEL2:Axitinib.1:Ldose: 0.301029996 (uM) Interaction | -29.7417703 | 19.0858749 | -1.558313174 | 0.119173719 |
| UACC0257:Axitinib.1:Ldose: 0.301029996 (uM) Interaction | -25.14872757 | 19.0858749 | -1.317661763 | 0.187630914 |
| MeWo:Ibrutinib..PCI.32765.:Ldose: 0.301029996 (uM) Interaction | -55.27998292 | 19.0858749 | -2.896381917 | 0.003778725 |
| SKMEL2:Ibrutinib..PCI.32765.:Ldose: 0.301029996 (uM) Interaction | -37.27964403 | 19.0858749 | -1.953258325 | 0.050801922 |
| UACC0257:Ibrutinib..PCI.32765.:Ldose: 0.301029996 (uM) Interaction | -13.36394707 | 19.0858749 | -0.700200915 | 0.483809403 |
| MeWo:Tamoxifen.Citrate:Ldose: 0.301029996 (uM) Interaction | -50.48816393 | 19.0858749 | -2.64531567 | 0.008167398 |
| SKMEL2:Tamoxifen.Citrate:Ldose: 0.301029996 (uM) Interaction | -36.29061457 | 19.0858749 | -1.901438355 | 0.05725795 |
| UACC0257:Tamoxifen.Citrate:Ldose: 0.301029996 (uM) Interaction | -29.7502037 | 19.0858749 | -1.558755041 | 0.11906906 |
| MeWo:Vemurafenib:Ldose: 0.301029996 (uM) Interaction | 21.92422558 | 19.0858749 | 1.148714728 | 0.250686387 |
| SKMEL2:Vemurafenib:Ldose: 0.301029996 (uM) Interaction | 41.64885679 | 19.0858749 | 2.182182217 | 0.029106854 |
| UACC0257:Vemurafenib:Ldose: 0.301029996 (uM) Interaction | 15.71871844 | 19.0858749 | 0.823578616 | 0.410188149 |
| MeWo:Pazopanib.HCl:Ldose: 0.301029996 (uM) Interaction | -40.3538126 | 19.0858749 | -2.114328676 | 0.034498663 |
| SKMEL2:Pazopanib.HCl:Ldose: 0.301029996 (uM) Interaction | -32.83793979 | 19.0858749 | -1.720536259 | 0.085349371 |
| UACC0257:Pazopanib.HCl:Ldose: 0.301029996 (uM) Interaction | -21.61829942 | 19.0858749 | -1.132685797 | 0.257358832 |
| MeWo:Abiraterone:Ldose: 0.301029996 (uM) Interaction | -56.79142787 | 19.0858749 | -2.975573725 | 0.002927656 |
| SKMEL2:Abiraterone:Ldose: 0.301029996 (uM) Interaction | -45.85343375 | 19.0858749 | -2.402480054 | 0.016292731 |
| UACC0257:Abiraterone:Ldose: 0.301029996 (uM) Interaction | -30.51935558 | 19.0858749 | -1.599054576 | 0.109823143 |
| MeWo:Bosutinib..SKI.606.:Ldose: 0.301029996 (uM) Interaction | -30.38918313 | 19.0858749 | -1.59223422 | 0.111346761 |
| SKMEL2:Bosutinib..SKI.606.:Ldose: 0.301029996 (uM) Interaction | -5.451180369 | 19.0858749 | -0.285613334 | 0.775177036 |
| UACC0257:Bosutinib..SKI.606.:Ldose: 0.301029996 (uM) Interaction | -13.09119007 | 19.0858749 | -0.685909875 | 0.492777325 |
| MeWo:Sabutoclax..BI.97C1.:Ldose: 0.301029996 (uM) Interaction | -53.2047202 | 19.0858749 | -2.787649007 | 0.005313854 |
| SKMEL2:Sabutoclax..BI.97C1.:Ldose: 0.301029996 (uM) Interaction | -45.42835081 | 19.0858749 | -2.380207931 | 0.017311519 |
| UACC0257:Sabutoclax..BI.97C1.:Ldose: 0.301029996 (uM) Interaction | 4.471959183 | 19.0858749 | 0.234307267 | 0.814748672 |
| MeWo:Thioguanine:Ldose: 0.602059991 (uM) Interaction | -20.26182756 | 19.0858749 | -1.061613768 | 0.288422963 |
| SKMEL2:Thioguanine:Ldose: 0.602059991 (uM) Interaction | 28.83012602 | 19.0858749 | 1.510547784 | 0.130918375 |
| UACC0257:Thioguanine:Ldose: 0.602059991 (uM) Interaction | 5.412478503 | 19.0858749 | 0.283585559 | 0.776730731 |
| MeWo:Irinotecan.HCl:Ldose: 0.602059991 (uM) Interaction | 0.551485046 | 19.0858749 | 0.028894931 | 0.976948656 |
| SKMEL2:Irinotecan.HCl:Ldose: 0.602059991 (uM) Interaction | -4.024889444 | 19.0858749 | -0.210883151 | 0.832980447 |
| UACC0257:Irinotecan.HCl:Ldose: 0.602059991 (uM) Interaction | 13.14497261 | 19.0858749 | 0.688727799 | 0.491001997 |
| MeWo:Romidepsin:Ldose: 0.602059991 (uM) Interaction | -40.56712459 | 19.0858749 | -2.125505108 | 0.033555835 |
| SKMEL2:Romidepsin:Ldose: 0.602059991 (uM) Interaction | -33.0503043 | 19.0858749 | -1.731663048 | 0.083347891 |
| UACC0257:Romidepsin:Ldose: 0.602059991 (uM) Interaction | -29.27544756 | 19.0858749 | -1.533880302 | 0.12507379 |
| MeWo:Paclitaxel:Ldose: 0.602059991 (uM) Interaction | -6.204966047 | 19.0858749 | -0.325107761 | 0.745102673 |
| SKMEL2:Paclitaxel:Ldose: 0.602059991 (uM) Interaction | 14.13530265 | 19.0858749 | 0.740615912 | 0.45893443 |
| UACC0257:Paclitaxel:Ldose: 0.602059991 (uM) Interaction | 4.339110508 | 19.0858749 | 0.227346691 | 0.820156327 |
| MeWo:Alisertib..MLN8237.:Ldose: 0.602059991 (uM) Interaction | -24.64975857 | 19.0858749 | -1.291518398 | 0.196537838 |
| SKMEL2:Alisertib..MLN8237.:Ldose: 0.602059991 (uM) Interaction | -11.22166976 | 19.0858749 | -0.587956791 | 0.55656746 |
| UACC0257:Alisertib..MLN8237.:Ldose: 0.602059991 (uM) Interaction | -2.63108172 | 19.0858749 | -0.137854918 | 0.890356369 |
| MeWo:Vorinostat:Ldose: 0.602059991 (uM) Interaction | -43.68252184 | 19.0858749 | -2.288735627 | 0.022104352 |
| SKMEL2:Vorinostat:Ldose: 0.602059991 (uM) Interaction | -45.84863588 | 19.0858749 | -2.402228671 | 0.016303929 |
| UACC0257:Vorinostat:Ldose: 0.602059991 (uM) Interaction | 39.19679997 | 19.0858749 | 2.053707267 | 0.040016078 |
| MeWo:Busulfan:Ldose: 0.602059991 (uM) Interaction | -42.17429899 | 19.0858749 | -2.209712639 | 0.02713559 |
| SKMEL2:Busulfan:Ldose: 0.602059991 (uM) Interaction | -29.97252956 | 19.0858749 | -1.570403753 | 0.116335898 |
| UACC0257:Busulfan:Ldose: 0.602059991 (uM) Interaction | 6.822731843 | 19.0858749 | 0.357475457 | 0.720739399 |
| MeWo:Mechlorethamine.HCl:Ldose: 0.602059991 (uM) Interaction | -56.78156336 | 19.0858749 | -2.975056877 | 0.002932591 |
| SKMEL2:Mechlorethamine.HCl:Ldose: 0.602059991 (uM) Interaction | -39.83612856 | 19.0858749 | -2.08720474 | 0.036881356 |
| UACC0257:Mechlorethamine.HCl:Ldose: 0.602059991 (uM) Interaction | -19.25890055 | 19.0858749 | -1.009065639 | 0.31295449 |
| MeWo:Teniposide:Ldose: 0.602059991 (uM) Interaction | -78.77840846 | 19.0858749 | -4.127576487 | 3.68E-05 |
| SKMEL2:Teniposide:Ldose: 0.602059991 (uM) Interaction | -49.33445864 | 19.0858749 | -2.584867548 | 0.009748153 |
| UACC0257:Teniposide:Ldose: 0.602059991 (uM) Interaction | -5.51253525 | 19.0858749 | -0.288828009 | 0.772715776 |
[truncated: 77,547 more chars]
