# Supplementary material for: Analysis of variability in high throughput screening data: applications to melanoma cell lines and drug responses
Source: Oncotarget. 2017 Feb 15;8(17):27786–99. doi: 10.18632/oncotarget.15347 (PMC5438608; doi:10.18632/oncotarget.15347)
Supplement: Supplementary file 6 [file oncotarget-08-27786-s006.docx]

**Supplemental Table 8:** Factor Significance in analysis with site, dose, cell line, plate, and drug-site interaction

| Covariate | Estimate | StErr | Tstat | Pval | Estimate_SiteInter | StErr_SiteInter | Tstat_SiteInter | Pval_SiteInter |
| --- | --- | --- | --- | --- | --- | --- | --- | --- |
| Intercept | 106.940216 | 1.242718011 | 86.05348527 | 0 |  |  |  |  |
| SBP | -3.274173459 | 1.699575526 | -1.926465408 | 0.054057343 |  |  |  |  |
| MeWo | 14.42875812 | 1.597811363 | 9.030326388 | 1.83E-19 |  |  |  |  |
| SKMEL2 | -9.808993905 | 1.60007289 | -6.130341917 | 8.90E-10 |  |  |  |  |
| UACC0257 | 3.881016734 | 1.598866232 | 2.427355496 | 0.015216194 |  |  |  |  |
| Abiraterone | 3.456631256 | 2.421952302 | 1.427208642 | 0.153531959 | -2.232658439 | 3.425157793 | -0.651841046 | 0.51450957 |
| ABT737 | -0.343332153 | 2.104058141 | -0.163176172 | 0.870381014 | -7.39932701 | 2.975587558 | -2.486677627 | 0.01290054 |
| Actinomycin | -67.11747472 | 2.104058141 | -31.89905898 | 5.32E-219 | -18.91926315 | 2.975587558 | -6.358160458 | 2.08E-10 |
| Afatinib | -12.13799063 | 2.104058141 | -5.768847541 | 8.07E-09 | -3.536870384 | 2.975587558 | -1.188629242 | 0.23459662 |
| Alisertib | -4.255281055 | 2.421952302 | -1.756963195 | 0.078936034 | -31.12499921 | 3.425157793 | -9.087172357 | 1.09E-19 |
| Allopurinol | 2.621282851 | 2.104058141 | 1.245822442 | 0.212840988 | 2.413975579 | 2.975587558 | 0.81126014 | 0.417223837 |
| Amifostine | 9.699484616 | 2.948772381 | 3.289329715 | 0.001005618 | -5.061705918 | 4.170193893 | -1.213781912 | 0.224842211 |
| Aphrocallistin | -36.08282355 | 2.104058141 | -17.14915708 | 1.48E-65 | 10.0099968 | 2.975587558 | 3.364040413 | 0.000769229 |
| Arsenic | 0.685475676 | 2.421952302 | 0.283026084 | 0.77715913 | 3.279797141 | 3.425157793 | 0.957560889 | 0.33829327 |
| Axitinib | 2.898927879 | 2.104058141 | 1.37777936 | 0.168283462 | -0.967321301 | 2.975587558 | -0.325085813 | 0.745118782 |
| Axitinib | 1.187386102 | 2.104058141 | 0.564331412 | 0.572533551 | 4.296027883 | 2.975587558 | 1.443757846 | 0.148819339 |
| Azacitidine | -4.078934265 | 2.104058141 | -1.9386034 | 0.052560625 | 1.720501954 | 2.975587558 | 0.57820579 | 0.563130276 |
| Baricitinib | 3.738070053 | 2.417898193 | 1.545999771 | 0.122116982 | 4.775481809 | 3.419424418 | 1.396574752 | 0.162553593 |
| Bendamustine | -4.588951657 | 2.947646233 | -1.556818998 | 0.119525825 | 7.56394167 | 4.16860128 | 1.814503514 | 0.06961194 |
| BGJ398 | 5.331248407 | 4.150028819 | 1.284629249 | 0.198933495 | -3.526728402 | 5.86902704 | -0.600905121 | 0.547908502 |
| Bioymifi | 1.586443387 | 2.104058141 | 0.753992181 | 0.45086085 | 2.131703006 | 2.975587558 | 0.716397338 | 0.473752572 |
| Bleomycin | -9.121390293 | 2.104058141 | -4.335141752 | 1.46E-05 | -28.47313368 | 2.975587558 | -9.568911392 | 1.17E-21 |
| Bortezomib | -92.18894525 | 2.104058141 | -43.81482787 | 0 | -0.945467616 | 2.975587558 | -0.317741487 | 0.75068363 |
| Bosutinib | -8.06478354 | 2.104058141 | -3.832966107 | 0.00012691 | 0.072530858 | 2.975587558 | 0.024375306 | 0.980553435 |
| Busulfan | -3.754045343 | 2.951980335 | -1.271704049 | 0.203489843 | 17.76360254 | 4.174730626 | 4.255029637 | 2.10E-05 |
| Cabazitaxel | -38.4378771 | 2.104058141 | -18.26844818 | 4.37E-74 | -16.98050243 | 2.975587558 | -5.706604863 | 1.17E-08 |
| Cabozantinib | -10.46635905 | 2.104058141 | -4.974367792 | 6.59E-07 | 9.927062847 | 2.975587558 | 3.336168959 | 0.000850623 |
| Capecitabine | -6.682220624 | 2.952788428 | -2.263020459 | 0.023642766 | 20.81513096 | 4.175873442 | 4.98461729 | 6.25E-07 |
| Carboplatin | -21.35377901 | 4.155699867 | -5.138431478 | 2.79E-07 | 35.39574007 | 5.877047113 | 6.022708238 | 1.74E-09 |
| Carfilzomib | -70.47302092 | 2.104058141 | -33.49385626 | 9.28E-241 | -9.795050277 | 2.975587558 | -3.291803748 | 0.000996818 |
| Carmustine | -10.29802408 | 2.952788428 | -3.487559076 | 0.000488269 | 19.0102159 | 4.175873442 | 4.55239273 | 5.33E-06 |
| Celecoxib | -7.126036069 | 2.952788428 | -2.413324301 | 0.015814723 | 10.64167233 | 4.175873442 | 2.548370413 | 0.010828535 |
| Chlorambucil | 12.25875443 | 2.944229642 | 4.163654307 | 3.14E-05 | -7.739007165 | 4.163769491 | -1.858654083 | 0.063087652 |
| Cisplatin | -4.724858761 | 2.421952302 | -1.950847156 | 0.051086123 | 16.43690071 | 3.425157793 | 4.79887401 | 1.60E-06 |
| Cladribine | -21.08072535 | 2.104058141 | -10.0190793 | 1.39E-23 | -25.48511066 | 2.975587558 | -8.564732229 | 1.14E-17 |
| Clofarabine | -27.55027271 | 2.104058141 | -13.09387425 | 4.77E-39 | -22.43695682 | 2.975587558 | -7.540345018 | 4.84E-14 |
| Crizotinib | -0.635447349 | 2.104058141 | -0.302010356 | 0.762646621 | -1.705122925 | 2.975587558 | -0.573037389 | 0.566624411 |
| Cytarabine | -9.540469524 | 2.104058141 | -4.534318392 | 5.81E-06 | -16.55346556 | 2.975587558 | -5.563091403 | 2.68E-08 |
| Dacarbazine | -0.785634901 | 2.421952302 | -0.324380831 | 0.74565238 | 2.313894137 | 3.425157793 | 0.675558405 | 0.499327159 |
| Dacomitinib | -0.467749309 | 2.104058141 | -0.222308167 | 0.824075768 | 5.285294776 | 2.975587558 | 1.776218872 | 0.075708722 |
| Dasatinib | -9.522964723 | 2.104058141 | -4.525998849 | 6.04E-06 | -2.635627773 | 2.975587558 | -0.885750367 | 0.375760379 |
| Daunorubicin | -45.86243859 | 2.104058141 | -21.79713465 | 2.20E-104 | -10.09447705 | 2.975587558 | -3.392431529 | 0.000693801 |
| Decitabine | -2.448059442 | 2.421952302 | -1.010779378 | 0.312131587 | -0.849892433 | 3.425157793 | -0.248132344 | 0.804034001 |
| Dexrazoxane | -19.14621688 | 4.155699867 | -4.607218397 | 4.10E-06 | 33.21970757 | 5.877047113 | 5.652448743 | 1.60E-08 |
| Docetaxel | -49.95389955 | 2.948772381 | -16.94057496 | 5.05E-64 | -0.522621756 | 4.170193893 | -0.125323131 | 0.900268725 |
| Doxorubicin | -23.75742479 | 2.104058141 | -11.29123969 | 1.70E-29 | -26.07682326 | 2.975587558 | -8.763587947 | 2.01E-18 |
| Erlotinib | -2.443910129 | 2.104058141 | -1.161522147 | 0.24544045 | -0.254110145 | 2.975587558 | -0.085398309 | 0.931945405 |
| Etoposide | -9.068639714 | 2.104058141 | -4.310070876 | 1.64E-05 | -10.23464537 | 2.975587558 | -3.439537629 | 0.000583637 |
| Everolimus | -1.394591819 | 2.952788428 | -0.472296561 | 0.63671915 | -35.04145155 | 4.175873442 | -8.391406502 | 5.05E-17 |
| Exemestane | -2.533367238 | 2.418480477 | -1.047503696 | 0.294877217 | 8.438284155 | 3.420247891 | 2.467155722 | 0.013625606 |
| Floxuridine | -26.17586386 | 2.104058141 | -12.44065616 | 1.99E-35 | -2.47249345 | 2.975587558 | -0.830926128 | 0.40602311 |
| Fludarabine | 4.055620898 | 2.421952302 | 1.674525504 | 0.094039579 | 2.598393894 | 3.425157793 | 0.758620201 | 0.448086768 |
| Fluorouracil | 3.569157246 | 2.104058141 | 1.696320637 | 0.08983733 | 2.667170628 | 2.975587558 | 0.896350914 | 0.370073802 |
| Flutamide | 3.093206748 | 2.104058141 | 1.470114675 | 0.141542986 | -2.707553874 | 2.975587558 | -0.909922434 | 0.362871983 |
| Foretinib | -17.21054418 | 2.104058141 | -8.179690402 | 2.98E-16 | 2.476047051 | 2.975587558 | 0.83212038 | 0.40534876 |
| Fulvestrant | 1.140277545 | 2.417898193 | 0.471598659 | 0.637217303 | 9.613031108 | 3.419424418 | 2.811300948 | 0.00493791 |
| Gefitinib | -8.199301215 | 2.952788428 | -2.776799427 | 0.005493694 | 16.65486896 | 4.175873442 | 3.988355775 | 6.67E-05 |
| Gemcitabine | -23.55221004 | 2.104058141 | -11.19370686 | 5.11E-29 | -40.87208524 | 2.975587558 | -13.73580324 | 8.79E-43 |
| Ibrutinib | -2.40810317 | 2.418480477 | -0.995709162 | 0.319400887 | 3.941776878 | 3.420247891 | 1.152482803 | 0.249133492 |
| Imatinib | -20.63926234 | 4.155699867 | -4.966494935 | 6.86E-07 | 33.10494192 | 5.877047113 | 5.632920968 | 1.79E-08 |
| Imiquimod | -0.483309035 | 2.418480477 | -0.199839957 | 0.841607337 | 10.32683583 | 3.420247891 | 3.019323791 | 0.002535891 |
| INK128 | -42.1624857 | 2.104058141 | -20.0386505 | 1.21E-88 | -15.22337994 | 2.975587558 | -5.11609208 | 3.14E-07 |
| Irinotecan | -6.791649748 | 2.104058141 | -3.227881215 | 0.001248677 | -12.27843904 | 2.975587558 | -4.126391445 | 3.70E-05 |
| Ixabepilone | -34.52999771 | 2.104058141 | -16.41114238 | 3.23E-60 | -13.95368089 | 2.975587558 | -4.689386758 | 2.75E-06 |
| Lapatinib | -1.365771564 | 2.418480477 | -0.564723006 | 0.572267131 | 6.156441568 | 3.420247891 | 1.799998644 | 0.071872619 |
| LDK378 | -2.935506044 | 2.104058141 | -1.395163939 | 0.16297851 | -17.99753645 | 2.975587558 | -6.048397535 | 1.48E-09 |
| Lenalidomide | -1.435617985 | 2.421952302 | -0.592752377 | 0.553352118 | 15.7034513 | 3.425157793 | 4.58473806 | 4.57E-06 |
| Letrozole | -5.030388974 | 2.951980335 | -1.704072657 | 0.088379648 | 21.54434802 | 4.174730626 | 5.160655849 | 2.48E-07 |
| Linsitinib | -3.66549344 | 2.104058141 | -1.742106537 | 0.081501812 | 1.221224092 | 2.975587558 | 0.410414437 | 0.681505392 |
| Lomustine | -6.512767652 | 2.951980335 | -2.206236801 | 0.02737627 | 15.76399892 | 4.174730626 | 3.776051759 | 0.000159694 |
| LY2157299 | -0.07427373 | 2.421952302 | -0.030666884 | 0.975535441 | 6.378683584 | 3.425157793 | 1.86230357 | 0.062571759 |
| Mechlorethamine | -2.576394926 | 2.104058141 | -1.224488466 | 0.220779281 | 4.612767244 | 2.975587558 | 1.550203835 | 0.121104959 |
| Megestrol | -1.227354488 | 2.421952302 | -0.506762452 | 0.612325869 | 16.99823595 | 3.425157793 | 4.962759959 | 6.99E-07 |
| MEK162 | -39.33668876 | 2.104058141 | -18.69562822 | 1.76E-77 | -16.7352917 | 2.975587558 | -5.624197364 | 1.88E-08 |
| Melphalan | -4.749676995 | 2.104058141 | -2.257388664 | 0.023992188 | 6.688418758 | 2.975587558 | 2.247764056 | 0.024599714 |
| Mercaptopurine | -16.32664642 | 2.104058141 | -7.759598512 | 8.84E-15 | 3.125911329 | 2.975587558 | 1.050519021 | 0.293489465 |
| Methotrexate | 9.758813221 | 4.150028819 | 2.351504928 | 0.018705177 | -13.70421914 | 5.86902704 | -2.335006987 | 0.019550741 |
| MitomycinC | -9.383612013 | 2.104058141 | -4.459768403 | 8.24E-06 | -8.491754557 | 2.975587558 | -2.853807657 | 0.004323333 |
| Mitotane | -0.70404208 | 2.421952302 | -0.290691967 | 0.77128927 | 7.002039588 | 3.425157793 | 2.044296938 | 0.040934428 |
| Mitoxantrone | -25.60037117 | 2.104058141 | -12.16714057 | 5.77E-34 | -14.46272966 | 2.975587558 | -4.860461799 | 1.18E-06 |
| MLN2480 | -6.668038588 | 2.944229642 | -2.264782099 | 0.023534376 | -4.928019702 | 4.163769491 | -1.183547675 | 0.236603152 |
| MLN4924 | -27.4732978 | 2.104058141 | -13.05729023 | 7.69E-39 | -3.262002108 | 2.975587558 | -1.096254788 | 0.2729776 |
| MLN9708 | -35.56390926 | 2.104058141 | -16.90253162 | 9.56E-64 | -24.7874425 | 2.975587558 | -8.330268228 | 8.46E-17 |
| Navitoclax | -4.187237632 | 2.104058141 | -1.990076962 | 0.046593075 | -2.048079864 | 2.975587558 | -0.688294269 | 0.491273726 |
| Nelarabine | 1.525431322 | 2.104058141 | 0.724994853 | 0.468461813 | 2.655416922 | 2.975587558 | 0.892400869 | 0.372186492 |
| OSI27 | -9.772162383 | 2.104058141 | -4.644435529 | 3.43E-06 | -7.838640298 | 2.975587558 | -2.634316801 | 0.008435751 |
| Oxaliplatin | 1.155828609 | 2.418480477 | 0.477915212 | 0.632714634 | -2.714638853 | 3.420247891 | -0.793696521 | 0.427379481 |
| Paclitaxel | -46.78872721 | 2.421952302 | -19.31859978 | 1.45E-82 | -8.56382837 | 3.425157793 | -2.500272655 | 0.012415978 |
| Palbociclib | -5.79570724 | 2.104058141 | -2.75453759 | 0.005881663 | -3.627997293 | 2.975587558 | -1.219254087 | 0.222759001 |
| Pazopanib | -3.874736982 | 2.104058141 | -1.841554141 | 0.065551954 | -4.145158292 | 2.975587558 | -1.393055392 | 0.163615135 |
| PD325901 | -54.42804746 | 2.104058141 | -25.86812903 | 1.13E-145 | -13.430958 | 2.975587558 | -4.513716279 | 6.40E-06 |
| Pemetrexed | -3.819122584 | 2.951980335 | -1.293749331 | 0.19576369 | 15.96688904 | 4.174730626 | 3.82465133 | 0.000131268 |
| Pipobroman | -20.21102651 | 4.155699867 | -4.863447109 | 1.16E-06 | 39.22735052 | 5.877047113 | 6.674670079 | 2.53E-11 |
| Plicamycin | -32.62983219 | 2.104058141 | -15.50804683 | 5.39E-54 | -36.36066031 | 2.975587558 | -12.21965733 | 3.04E-34 |
| Pralatrexate | -70.89656702 | 2.418480477 | -29.31450871 | 7.97E-186 | 27.00480616 | 3.420247891 | 7.895569859 | 3.01E-15 |
| Quinacrine | -2.975451791 | 2.104058141 | -1.414149036 | 0.157330307 | 8.995061147 | 2.975587558 | 3.022952936 | 0.002505688 |
| Quizartinib | 3.09005628 | 2.104058141 | 1.468617345 | 0.141948893 | -0.40384785 | 2.975587558 | -0.135720372 | 0.892043409 |
| Raloxifene | -3.998803923 | 2.418480477 | -1.653436511 | 0.098254317 | 11.22299615 | 3.420247891 | 3.28133998 | 0.001034533 |
| Romidepsin | -29.89293444 | 2.104058141 | -14.20727587 | 1.23E-45 | -63.42276361 | 2.975587558 | -21.31436644 | 6.18E-100 |
| Sabutoclax | -18.53884622 | 2.104058141 | -8.810995222 | 1.32E-18 | -0.224352754 | 2.975587558 | -0.075397799 | 0.939898799 |
| Sirolimus | -2.980208887 | 2.421952302 | -1.230498588 | 0.21852176 | -17.48614052 | 3.425157793 | -5.105207286 | 3.33E-07 |
| Sorafenib | 0.045225675 | 2.415533257 | 0.018722853 | 0.985062343 | -4.412337256 | 3.416079893 | -1.291637606 | 0.196494334 |
| Streptozocin | 1.625293457 | 2.104058141 | 0.772456533 | 0.439851212 | 2.788931639 | 2.975587558 | 0.937270903 | 0.348628067 |
| Sunitinib | 3.779314304 | 2.415533257 | 1.564587982 | 0.117691907 | 1.040351893 | 3.416079893 | 0.304545539 | 0.760714779 |
| Tamoxifen | -0.026875217 | 2.104058141 | -0.012773039 | 0.989808966 | -1.663390427 | 2.975587558 | -0.559012429 | 0.576158127 |
| Temozolomide | -2.084795412 | 2.952788428 | -0.706042936 | 0.480167861 | 13.10714278 | 4.175873442 | 3.138778741 | 0.001698461 |
| Temsirolimus | 5.608753962 | 2.951980335 | 1.899997061 | 0.057444729 | -27.07404677 | 4.174730626 | -6.485220052 | 9.02E-11 |
| Teniposide | -31.05360484 | 2.104058141 | -14.75891005 | 4.29E-49 | -15.42228025 | 2.975587558 | -5.182936124 | 2.20E-07 |
| Thioguanine | -21.59984535 | 2.104058141 | -10.26580252 | 1.12E-24 | -1.098939735 | 2.975587558 | -0.369318568 | 0.711893341 |
| Thiotepa | -0.718867047 | 2.104058141 | -0.341657406 | 0.732611534 | 11.29171529 | 2.975587558 | 3.794785087 | 0.000148112 |
| Topotecan | -31.88326937 | 2.104058141 | -15.1532264 | 1.21E-51 | -18.9469185 | 2.975587558 | -6.367454538 | 1.95E-10 |
| Trametinib | -60.04175348 | 2.415533257 | -24.85652114 | 8.72E-135 | 1.194182639 | 3.416079893 | 0.349576906 | 0.72665912 |
| Tretinoin | -22.43499864 | 4.155699867 | -5.398608984 | 6.78E-08 | 36.0439708 | 5.877047113 | 6.133006952 | 8.75E-10 |
| Triethylenemelamine | -1.350577825 | 2.104058141 | -0.641891875 | 0.520949137 | 9.605489032 | 2.975587558 | 3.228098264 | 0.00124773 |
| Uracil | -4.654629893 | 2.421952302 | -1.921850356 | 0.054635676 | 17.35963851 | 3.425157793 | 5.068274095 | 4.04E-07 |
| Valrubicin | -8.577712659 | 2.104058141 | -4.076746974 | 4.58E-05 | -13.57597243 | 2.975587558 | -4.562451 | 5.08E-06 |
| Vandetanib | -2.822537792 | 2.418480477 | -1.167070737 | 0.243192673 | 8.693167055 | 3.420247891 | 2.541677484 | 0.011038036 |
| Vemurafenib | -19.65705624 | 2.417898193 | -8.129811375 | 4.49E-16 | -1.278089432 | 3.419424418 | -0.373773266 | 0.708576106 |
| Vinblastine | -0.727970734 | 2.417898193 | -0.301075842 | 0.763359107 | -53.83463913 | 3.419424418 | -15.74377221 | 1.38E-55 |
| Vincristine | -36.16226039 | 2.104058141 | -17.18691119 | 7.80E-66 | -18.43444044 | 2.975587558 | -6.195227018 | 5.91E-10 |
| Vinorelbine | -25.87822801 | 2.104058141 | -12.29919816 | 1.15E-34 | -7.226264489 | 2.975587558 | -2.428516838 | 0.01516756 |
| Vismodegib | 2.320720574 | 2.104058141 | 1.102973596 | 0.270048998 | 2.481866559 | 2.975587558 | 0.834076131 | 0.404245866 |
| Vorinostat | -29.11985266 | 2.104058141 | -13.83985171 | 2.10E-43 | 10.9218898 | 2.975587558 | 3.670498543 | 0.000242569 |
| Zoledronic | -5.733254176 | 2.952788428 | -1.941640695 | 0.052191568 | 8.808666362 | 4.175873442 | 2.1094189 | 0.03491812 |
| Ldose: -1.397940009 (uM) | -3.290650486 | 1.657746209 | -1.985014635 | 0.047153496 |  |  |  |  |
| Ldose: -1 (uM) | -5.043578603 | 1.657746209 | -3.042431088 | 0.002349132 |  |  |  |  |
| Ldose: -0.698970004 (uM) | -6.169655059 | 0.563108388 | -10.95642542 | 7.14E-28 |  |  |  |  |
| Ldose: -0.397940009 (uM) | -9.698519416 | 1.657746209 | -5.850424729 | 4.96E-09 |  |  |  |  |
| Ldose: 0 (uM) | -11.54557581 | 1.657746209 | -6.964622055 | 3.37E-12 |  |  |  |  |
| Ldose: 0.301029996 (uM) | -13.39789184 | 0.563108388 | -23.79274065 | 8.77E-124 |  |  |  |  |
| Ldose: 0.602059991 (uM) | -17.20397433 | 1.657746209 | -10.37793013 | 3.51E-25 |  |  |  |  |
| Ldose: 1 (uM) | -22.4686118 | 1.657746209 | -13.55371026 | 1.05E-41 |  |  |  |  |
| Plate: -35 | 2.542300278 | 1.592711039 | 1.596209366 | 0.110454399 |  |  |  |  |
| Plate: -34 | 3.4980325 | 1.592711039 | 2.196275667 | 0.028081152 |  |  |  |  |
| Plate: -33 | 6.157534063 | 2.252433553 | 2.733725066 | 0.006266533 |  |  |  |  |
| Plate: -32 | 5.129357119 | 2.252433553 | 2.277251248 | 0.022779436 |  |  |  |  |
| Plate: -31 | 4.968404619 | 2.252433553 | 2.20579409 | 0.02740727 |  |  |  |  |
| Plate: -30 | 3.561967451 | 2.252433553 | 1.581386251 | 0.113802101 |  |  |  |  |
| Plate: -29 | 4.511690229 | 2.252433553 | 2.003029223 | 0.045184673 |  |  |  |  |
| Plate: -28 | 2.864528284 | 2.252433553 | 1.271748186 | 0.203474155 |  |  |  |  |
| Plate: -27 | -33.34313542 | 2.26085903 | -14.74799401 | 5.04E-49 |  |  |  |  |
| Plate: -26 | 4.277811806 | 2.26085903 | 1.892117885 | 0.058486548 |  |  |  |  |
| Plate: -25 | 6.897628473 | 2.26085903 | 3.050888349 | 0.002283988 |  |  |  |  |
| Plate: -24 | 21.01004587 | 2.765540093 | 7.59708598 | 3.13E-14 |  |  |  |  |
| Plate: -23 | 8.997037259 | 2.765540093 | 3.253265892 | 0.001142345 |  |  |  |  |
| Plate: -22 | 6.650188092 | 2.765540093 | 2.40466161 | 0.016194501 |  |  |  |  |
| Plate: -21 | 1.136493424 | 2.26085903 | 0.502682126 | 0.615192102 |  |  |  |  |
| Plate: -20 | 5.745969535 | 2.26085903 | 2.541498368 | 0.011043691 |  |  |  |  |
| Plate: -19 | -1.252186298 | 2.26085903 | -0.553854213 | 0.57968346 |  |  |  |  |
| Plate: -18 | 0.718686429 | 2.2596465 | 0.318052593 | 0.750447637 |  |  |  |  |
| Plate: -17 | 4.372170318 | 2.2596465 | 1.934891284 | 0.053014638 |  |  |  |  |
| Plate: -16 | 3.936521707 | 2.2596465 | 1.742096256 | 0.08150361 |  |  |  |  |
| Plate: -15 | 7.158114659 | 2.764548925 | 2.589252299 | 0.009623901 |  |  |  |  |
| Plate: -14 | 11.28071633 | 2.764548925 | 4.080490753 | 4.51E-05 |  |  |  |  |
| Plate: -13 | 11.25353744 | 2.764548925 | 4.070659533 | 4.70E-05 |  |  |  |  |
| Plate: -12 | 11.19677416 | 2.2596465 | 4.955099906 | 7.28E-07 |  |  |  |  |
| Plate: -11 | 9.823111658 | 2.2596465 | 4.347189553 | 1.38E-05 |  |  |  |  |
| Plate: -10 | 1.710328047 | 2.2596465 | 0.756900713 | 0.449116313 |  |  |  |  |
| Plate: -9 | 7.755303389 | 2.259381216 | 3.432489981 | 0.000599011 |  |  |  |  |
| Plate: -8 | 7.945430889 | 2.259381216 | 3.516640234 | 0.000437802 |  |  |  |  |
| Plate: -7 | 4.915765056 | 2.259381216 | 2.175712987 | 0.029585843 |  |  |  |  |
| Plate: -6 | 9.228562731 | 2.764332096 | 3.33844213 | 0.000843697 |  |  |  |  |
| Plate: -5 | 10.81270829 | 2.764332096 | 3.911508426 | 9.20E-05 |  |  |  |  |
| Plate: -4 | 11.12952634 | 2.764332096 | 4.026117686 | 5.69E-05 |  |  |  |  |
| Plate: -3 | 11.02101473 | 2.259381216 | 4.877890747 | 1.08E-06 |  |  |  |  |
| Plate: -2 | 11.88822473 | 2.259381216 | 5.261717077 | 1.44E-07 |  |  |  |  |
| Plate: -1 | 13.00523362 | 2.259381216 | 5.75610416 | 8.71E-09 |  |  |  |  |
| Plate: 1 | 2.08809055 | 2.252433553 | 0.92703758 | 0.353915761 |  |  |  |  |
| Plate: 2 | -1.369852595 | 2.252433553 | -0.608165596 | 0.543083044 |  |  |  |  |
| Plate: 3 | -6.020651782 | 2.252433553 | -2.672954225 | 0.007523416 |  |  |  |  |
| Plate: 4 | 3.151328017 | 2.252433553 | 1.399077017 | 0.161802002 |  |  |  |  |
| Plate: 5 | 3.175641904 | 2.252433553 | 1.409871514 | 0.158589772 |  |  |  |  |
| Plate: 6 | 2.382005383 | 2.252433553 | 1.057525262 | 0.290281909 |  |  |  |  |
| Plate: 7 | 1.098031908 | 1.592711039 | 0.689410622 | 0.490571149 |  |  |  |  |
| Plate: 8 | -0.005717464 | 1.592711039 | -0.003589769 | 0.997135813 |  |  |  |  |
| Plate: 10 | -2.135263217 | 2.252433553 | -0.947980558 | 0.343148299 |  |  |  |  |
| Plate: 11 | -2.254600746 | 2.252433553 | -1.000962156 | 0.316854564 |  |  |  |  |
| Plate: 12 | -2.415679551 | 2.252433553 | -1.072475389 | 0.28351667 |  |  |  |  |
| Plate: 13 | 1.074382663 | 2.252433553 | 0.476987506 | 0.63337509 |  |  |  |  |
| Plate: 14 | -0.116427445 | 2.252433553 | -0.051689625 | 0.958776408 |  |  |  |  |
| Plate: 15 | -0.334813242 | 2.252433553 | -0.148645114 | 0.881834843 |  |  |  |  |
| Plate: 16 | -5.626774521 | 1.592711039 | -3.532828229 | 0.000411863 |  |  |  |  |
| Plate: 17 | 0.366361283 | 1.592711039 | 0.230023698 | 0.818075193 |  |  |  |  |
| Plate: 19 | -0.72874536 | 2.252433553 | -0.323536896 | 0.746291312 |  |  |  |  |
| Plate: 20 | -1.485489878 | 2.252433553 | -0.659504418 | 0.509577842 |  |  |  |  |
| Plate: 21 | 3.705302017 | 2.252433553 | 1.645021676 | 0.099977617 |  |  |  |  |
| Plate: 22 | 0.835383545 | 2.252433553 | 0.370880439 | 0.710729653 |  |  |  |  |
| Plate: 23 | 0.919318518 | 2.252433553 | 0.408144567 | 0.683170955 |  |  |  |  |
| Plate: 24 | 1.136611096 | 2.252433553 | 0.504614706 | 0.613833821 |  |  |  |  |
| Plate: 25 | 22.87469134 | 1.592711039 | 14.36211012 | 1.36E-46 |  |  |  |  |
| Plate: 26 | -0.095872012 | 1.592711039 | -0.060194228 | 0.952001412 |  |  |  |  |
| Plate: 28 | -0.525180938 | 1.592711039 | -0.329740251 | 0.741598934 |  |  |  |  |
| Plate: 29 | -0.413642737 | 1.592711039 | -0.259709845 | 0.795089686 |  |  |  |  |
| Plate: 31 | 0.969504552 | 1.592711039 | 0.6087134 | 0.542719824 |  |  |  |  |
| Plate: 32 | 0.557125075 | 1.592711039 | 0.349796706 | 0.726494149 |  |  |  |  |
| Plate: 34 | -1.622432408 | 1.592711039 | -1.018660867 | 0.308373622 |  |  |  |  |
| Plate: 35 | 0.043810796 | 1.592711039 | 0.027507059 | 0.978055524 |  |  |  |  |

Supplemental Table 8: ANOVA analysis for site, dose, cell line, plate, and drug-site interaction effects using an AIC derived set of control drugs as baseline. Signif. codes: 0 ‘***’ 0.001 ‘**’ 0.01 ‘*’ 0.05 ‘.’ 0.1 ‘ ’ 1
