# Supplementary material for: Analysis of variability in high throughput screening data: applications to melanoma cell lines and drug responses
Source: Oncotarget. 2017 Feb 15;8(17):27786–99. doi: 10.18632/oncotarget.15347 (PMC5438608; doi:10.18632/oncotarget.15347)
Supplement: Supplementary file 7 [file oncotarget-08-27786-s007.docx]

**Supplemental Table 10:** Factor Significance in analysis for site, dose, cell line, plate, and drug-dose-cell line interaction effects using an AIC derived set of control drugs as baseline.

| Covariate | Estimate | StErr | Tstat | Pval |
| --- | --- | --- | --- | --- |
| Intercept | 104.3575175 | 1.801281539 | 57.93515074 | 0 |
| SBP | -6.033357009 | 1.350002686 | -4.469144449 | 7.89E-06 |
| MeWo | 10.02569102 | 2.595718413 | 3.862395462 | 0.000112598 |
| SKMEL2 | -27.64519862 | 3.077582648 | -8.982764 | 2.85E-19 |
| UACC0257 | -0.344748565 | 2.350053031 | -0.146698207 | 0.883371561 |
| Abiraterone | 4.570514585 | 7.56045334 | 0.60452917 | 0.545498033 |
| ABT737 | -7.732933699 | 7.56045334 | -1.022813494 | 0.306407134 |
| Actinomycin | -60.46907713 | 7.56045334 | -7.998075567 | 1.32E-15 |
| Afatinib | 9.053865518 | 7.56045334 | 1.197529448 | 0.231112987 |
| Alisertib | -20.67393992 | 7.56045334 | -2.734484162 | 0.006252744 |
| Allopurinol | -1.539927815 | 7.56045334 | -0.203681942 | 0.838603914 |
| Amifostine | 6.291126585 | 7.56045334 | 0.832109703 | 0.405355937 |
| Aphrocallistin | -1.729803204 | 7.56045334 | -0.228796228 | 0.819029397 |
| Arsenic | 8.915879635 | 7.56045334 | 1.179278442 | 0.238299903 |
| Axitinib | 9.990091028 | 7.56045334 | 1.321361376 | 0.186394457 |
| Axitinib | 11.98525522 | 7.56045334 | 1.585256159 | 0.112922328 |
| Azacitidine | -10.75386033 | 7.56045334 | -1.422383004 | 0.154929084 |
| Baricitinib | 10.97630153 | 7.56045334 | 1.451804679 | 0.146569993 |
| Bendamustine | 14.12173245 | 7.496263975 | 1.883836067 | 0.059600154 |
| BGJ398 | 3.078701701 | 7.56045334 | 0.407211256 | 0.683856744 |
| Bioymifi | -3.971531125 | 7.56045334 | -0.525303305 | 0.599377584 |
| Bleomycin | 2.308963185 | 7.56045334 | 0.305400097 | 0.760064299 |
| Bortezomib | -81.61159381 | 7.56045334 | -10.79453707 | 4.26E-27 |
| Bosutinib | 11.830481 | 7.56045334 | 1.564784606 | 0.117647614 |
| Busulfan | 5.431716901 | 7.56045334 | 0.71843799 | 0.472494798 |
| Cabazitaxel | -57.7200639 | 7.56045334 | -7.634471282 | 2.36E-14 |
| Cabozantinib | 4.289094201 | 7.56045334 | 0.567306484 | 0.570511684 |
| Capecitabine | 12.47082866 | 7.755175001 | 1.60806541 | 0.107835013 |
| Carboplatin | 10.07319145 | 7.755175001 | 1.298899309 | 0.193991919 |
| Carfilzomib | -44.67230908 | 7.56045334 | -5.908681275 | 3.50E-09 |
| Carmustine | 9.72321005 | 7.755175001 | 1.253770553 | 0.209938561 |
| Celecoxib | 9.121454413 | 7.755175001 | 1.176176477 | 0.23953692 |
| Chlorambucil | 6.104315951 | 7.56045334 | 0.807400784 | 0.419444229 |
| Cisplatin | 6.860950885 | 7.56045334 | 0.907478768 | 0.36416348 |
| Cladribine | -13.91415167 | 7.56045334 | -1.840385892 | 0.065724885 |
| Clofarabine | -12.97922291 | 7.56045334 | -1.716725482 | 0.086043231 |
| Crizotinib | 5.417632338 | 7.56045334 | 0.716575064 | 0.473643841 |
| Cytarabine | 0.632596376 | 7.56045334 | 0.083671752 | 0.933318168 |
| Dacarbazine | -8.932131199 | 7.56045334 | -1.181427991 | 0.237445345 |
| Dacomitinib | 2.916259866 | 7.56045334 | 0.385725529 | 0.699703634 |
| Dasatinib | -18.61058798 | 7.56045334 | -2.461570377 | 0.01384054 |
| Daunorubicin | -8.189207644 | 7.56045334 | -1.083163572 | 0.278747539 |
| Decitabine | -3.005777015 | 7.56045334 | -0.397565712 | 0.690954145 |
| Dexrazoxane | 12.44890524 | 7.755175001 | 1.605238468 | 0.108455497 |
| Docetaxel | -56.03041341 | 7.56045334 | -7.410985941 | 1.30E-13 |
| Doxorubicin | -5.552500015 | 7.56045334 | -0.734413634 | 0.462704403 |
| Erlotinib | -1.927051992 | 7.56045334 | -0.254885773 | 0.798813676 |
| Etoposide | 0.444107838 | 7.56045334 | 0.058740901 | 0.953159008 |
| Everolimus | -0.887136427 | 7.755175001 | -0.114392831 | 0.908927426 |
| Exemestane | 13.71593441 | 7.496263975 | 1.829702697 | 0.067307753 |
| Floxuridine | -29.49198674 | 7.56045334 | -3.900822531 | 9.61E-05 |
| Fludarabine | -0.502230087 | 7.56045334 | -0.066428568 | 0.947037222 |
| Fluorouracil | 3.918485583 | 7.56045334 | 0.518287119 | 0.604263102 |
| Flutamide | 7.206856118 | 7.56045334 | 0.953230685 | 0.340483536 |
| Foretinib | 7.039009368 | 7.56045334 | 0.931030066 | 0.351848093 |
| Fulvestrant | 3.616536801 | 7.56045334 | 0.478349199 | 0.632406375 |
| Gefitinib | 12.30900031 | 7.755175001 | 1.587198266 | 0.112481928 |
| Gemcitabine | -47.17630053 | 7.56045334 | -6.239877216 | 4.46E-10 |
| Ibrutinib | 5.45943403 | 7.496263975 | 0.728287324 | 0.466445369 |
| Imatinib | 11.66058095 | 7.755175001 | 1.503587082 | 0.132701883 |
| Imiquimod | -3.79487092 | 7.496263975 | -0.506234964 | 0.612696718 |
| INK128 | -15.66509703 | 7.56045334 | -2.071978535 | 0.038278912 |
| Irinotecan | -1.290175065 | 7.56045334 | -0.170647844 | 0.864502224 |
| Ixabepilone | 4.950572451 | 7.56045334 | 0.65479836 | 0.512604341 |
| Lapatinib | -2.579518903 | 7.496263975 | -0.344107266 | 0.730768864 |
| LDK378 | 9.189966701 | 7.56045334 | 1.215531171 | 0.224176438 |
| Lenalidomide | 0.612365861 | 7.56045334 | 0.080995918 | 0.935445926 |
| Letrozole | 11.81326102 | 7.56045334 | 1.562506968 | 0.118182798 |
| Linsitinib | -1.79183364 | 7.56045334 | -0.23700082 | 0.812658332 |
| Lomustine | -1.086286632 | 7.56045334 | -0.143680092 | 0.885754391 |
| LY2157299 | 6.356221651 | 7.56045334 | 0.840719645 | 0.400514028 |
| Mechlorethamine | -5.340339997 | 7.56045334 | -0.706351823 | 0.479976754 |
| Megestrol | 9.5918563 | 7.56045334 | 1.268687983 | 0.204565615 |
| MEK162 | -20.5358201 | 7.56045334 | -2.716215441 | 0.006608384 |
| Melphalan | 4.262416335 | 7.56045334 | 0.563777877 | 0.57291098 |
| Mercaptopurine | 10.45629678 | 7.56045334 | 1.383025106 | 0.166671017 |
| Methotrexate | 6.173097768 | 7.56045334 | 0.816498362 | 0.414223883 |
| MitomycinC | -1.600870589 | 7.56045334 | -0.211742672 | 0.832309731 |
| Mitotane | 2.168582245 | 7.56045334 | 0.286832303 | 0.774243393 |
| Mitoxantrone | 5.875896646 | 7.56045334 | 0.777188402 | 0.43705587 |
| MLN2480 | 9.513499696 | 7.56045334 | 1.258323975 | 0.208287791 |
| MLN4924 | 7.266444918 | 7.56045334 | 0.961112329 | 0.336506114 |
| MLN9708 | -7.695855394 | 7.56045334 | -1.017909251 | 0.30873213 |
| Navitoclax | -3.914856339 | 7.56045334 | -0.517807089 | 0.604598009 |
| Nelarabine | -6.183538649 | 7.56045334 | -0.817879348 | 0.413434824 |
| OSI27 | -10.16458007 | 7.56045334 | -1.344440553 | 0.178819646 |
| Oxaliplatin | 10.1735251 | 7.496263975 | 1.357146057 | 0.174748545 |
| Paclitaxel | -46.21858851 | 7.56045334 | -6.113203326 | 9.93E-10 |
| Palbociclib | 2.683903335 | 7.56045334 | 0.354992381 | 0.722598657 |
| Pazopanib | 2.524724685 | 7.56045334 | 0.333938267 | 0.738429284 |
| PD325901 | -67.8177328 | 7.56045334 | -8.970061682 | 3.19E-19 |
| Pemetrexed | 8.444373951 | 7.56045334 | 1.116913705 | 0.26404326 |
| Pipobroman | 15.93981029 | 7.755175001 | 2.055377253 | 0.039854202 |
| Plicamycin | 3.537821093 | 7.56045334 | 0.467937693 | 0.639833721 |
| Pralatrexate | -78.35753716 | 7.496263975 | -10.45287858 | 1.63E-25 |
| Quinacrine | 1.716819315 | 7.56045334 | 0.227078885 | 0.820364484 |
| Quizartinib | 1.442750368 | 7.56045334 | 0.190828553 | 0.848661655 |
| Raloxifene | 16.6196971 | 7.496263975 | 2.217064014 | 0.026628765 |
| Romidepsin | -89.23507232 | 7.56045334 | -11.80287323 | 4.70E-32 |
| Sabutoclax | 6.039180385 | 7.56045334 | 0.798785485 | 0.424423317 |
| Sirolimus | -7.517640127 | 7.56045334 | -0.994337216 | 0.320069514 |
| Sorafenib | -1.687293065 | 7.56045334 | -0.223173531 | 0.823402496 |
| Streptozocin | -3.249439882 | 7.56045334 | -0.429794317 | 0.66734942 |
| Sunitinib | -2.891224382 | 7.56045334 | -0.382414156 | 0.702157837 |
| Tamoxifen | 0.043557168 | 7.56045334 | 0.005761185 | 0.995403317 |
| Temozolomide | 13.22457358 | 7.755175001 | 1.705257919 | 0.088160262 |
| Temsirolimus | -3.530329552 | 7.56045334 | -0.466946808 | 0.640542498 |
| Teniposide | -26.80511301 | 7.56045334 | -3.545437265 | 0.000392765 |
| Thioguanine | -3.15609874 | 7.56045334 | -0.417448346 | 0.676354518 |
| Thiotepa | -0.140008747 | 7.56045334 | -0.018518565 | 0.985225333 |
| Topotecan | 2.82680964 | 7.56045334 | 0.373894198 | 0.708486588 |
| Trametinib | -75.45950467 | 7.56045334 | -9.980817456 | 2.07E-23 |
| Tretinoin | 9.344295916 | 7.755175001 | 1.204911032 | 0.228250438 |
| Triethylenemelamine | 5.165813918 | 7.56045334 | 0.683267747 | 0.494444771 |
| Uracil | 10.10913235 | 7.56045334 | 1.337106639 | 0.181201472 |
| Valrubicin | -3.452050599 | 7.56045334 | -0.45659307 | 0.647968009 |
| Vandetanib | 0.96323308 | 7.496263975 | 0.128495085 | 0.897758346 |
| Vemurafenib | -5.24172385 | 7.56045334 | -0.693308141 | 0.488123417 |
| Vinblastine | -22.52218735 | 7.56045334 | -2.978946676 | 0.002895525 |
| Vincristine | -28.78342247 | 7.56045334 | -3.80710272 | 0.000140981 |
| Vinorelbine | 1.75112046 | 7.56045334 | 0.231615801 | 0.816838559 |
| Vismodegib | 0.471743885 | 7.56045334 | 0.062396243 | 0.950247846 |
| Vorinostat | -4.740804332 | 7.56045334 | -0.627052919 | 0.530630968 |
| Zoledronic | 8.463115198 | 7.755175001 | 1.091286167 | 0.275158775 |
| Ldose: -1.397940009 (uM) | -0.340177951 | 3.457853687 | -0.098378353 | 0.921632747 |
| Ldose: -1 (uM) | 2.831645534 | 3.457853687 | 0.818902646 | 0.412850713 |
| Ldose: -0.698970004 (uM) | -0.256183726 | 2.22945606 | -0.114908623 | 0.908518585 |
| Ldose: -0.397940009 (uM) | -1.141213575 | 3.457853687 | -0.330035241 | 0.741376435 |
| Ldose: 0 (uM) | 3.422214927 | 3.457853687 | 0.989693387 | 0.322334748 |
| Ldose: 0.301029996 (uM) | 2.412285718 | 2.22945606 | 1.082006397 | 0.279261388 |
| Ldose: 0.602059991 (uM) | -0.337351038 | 2.643156786 | -0.127631868 | 0.898441462 |
| Ldose: 1 (uM) | 1.003194749 | 2.643156786 | 0.37954417 | 0.704287425 |
| Plate: -35 | 2.542300278 | 1.350002686 | 1.883181645 | 0.059688761 |
| Plate: -34 | 3.4980325 | 1.350002686 | 2.59113003 | 0.009572365 |
| Plate: -33 | 6.157534063 | 1.909192107 | 3.225204022 | 0.001260643 |
| Plate: -32 | 5.129357119 | 1.909192107 | 2.686663694 | 0.007222307 |
| Plate: -31 | 4.968404619 | 1.909192107 | 2.60235971 | 0.009264593 |
| Plate: -30 | 3.561967451 | 1.909192107 | 1.865693577 | 0.062097371 |
| Plate: -29 | 4.511690229 | 1.909192107 | 2.363141044 | 0.018129304 |
| Plate: -28 | 2.864528284 | 1.909192107 | 1.500387663 | 0.133528173 |
| Plate: -27 | -38.28931547 | 3.397432255 | -11.27007475 | 2.21E-29 |
| Plate: -26 | -0.668368245 | 3.397432255 | -0.196727468 | 0.844042607 |
| Plate: -25 | 1.951448422 | 3.397432255 | 0.574389208 | 0.565710249 |
| Plate: -24 | 17.42954122 | 3.655824555 | 4.767608773 | 1.88E-06 |
| Plate: -23 | 5.416532611 | 3.655824555 | 1.481617219 | 0.138456307 |
| Plate: -22 | 3.069683444 | 3.655824555 | 0.839669245 | 0.401102866 |
| Plate: -21 | 0.119387301 | 1.909192107 | 0.062532891 | 0.950139031 |
| Plate: -20 | 4.728863412 | 1.909192107 | 2.476892396 | 0.013260544 |
| Plate: -19 | -2.269292421 | 1.909192107 | -1.188613976 | 0.234604266 |
| Plate: -18 | -2.034460582 | 3.670900184 | -0.554212994 | 0.579438645 |
| Plate: -17 | 1.619023306 | 3.670900184 | 0.441042585 | 0.659186444 |
| Plate: -16 | 1.183374695 | 3.670900184 | 0.32236635 | 0.747178196 |
| Plate: -15 | 8.164323403 | 3.91126775 | 2.087385453 | 0.036864631 |
| Plate: -14 | 12.28692507 | 3.91126775 | 3.141417529 | 0.00168352 |
| Plate: -13 | 12.25974618 | 3.91126775 | 3.134468659 | 0.00172389 |
| Plate: -12 | 9.91574655 | 1.909192107 | 5.193687168 | 2.08E-07 |
| Plate: -11 | 8.54208405 | 1.909192107 | 4.474187808 | 7.71E-06 |
| Plate: -10 | 0.429300439 | 1.909192107 | 0.224859739 | 0.822090452 |
| Plate: -9 | 6.127099769 | 4.409090308 | 1.389651683 | 0.164648549 |
| Plate: -8 | 6.317227269 | 4.409090308 | 1.43277339 | 0.151936629 |
| Plate: -7 | 3.287561435 | 4.409090308 | 0.745632592 | 0.45589725 |
| Plate: -6 | 9.624091547 | 4.611137018 | 2.087140657 | 0.03688675 |
| Plate: -5 | 11.2082371 | 4.611137018 | 2.430688366 | 0.015078002 |
| Plate: -4 | 11.52505516 | 4.611137018 | 2.49939551 | 0.01244767 |
| Plate: -3 | 12.65066772 | 1.909192107 | 6.62618899 | 3.52E-11 |
| Plate: -2 | 13.51787772 | 1.909192107 | 7.080417769 | 1.48E-12 |
| Plate: -1 | 14.63488661 | 1.909192107 | 7.66548665 | 1.85E-14 |
| Plate: 1 | -1.169766063 | 4.409090308 | -0.265307803 | 0.790774807 |
| Plate: 2 | -4.627709208 | 4.409090308 | -1.049583675 | 0.293920943 |
| Plate: 3 | -9.278508395 | 4.409090308 | -2.104404253 | 0.035354363 |
| Plate: 4 | 1.917203841 | 4.409090308 | 0.434829796 | 0.663690184 |
| Plate: 5 | 1.941517729 | 4.409090308 | 0.440344287 | 0.659692038 |
| Plate: 6 | 1.147881208 | 4.409090308 | 0.260344227 | 0.794600664 |
| Plate: 7 | 1.098031908 | 1.350002686 | 0.813355351 | 0.416023036 |
| Plate: 8 | -0.005717464 | 1.350002686 | -0.00423515 | 0.996620887 |
| Plate: 10 | -3.607382621 | 3.670900184 | -0.982697006 | 0.325767248 |
| Plate: 11 | -3.72672015 | 3.670900184 | -1.01520607 | 0.310018624 |
| Plate: 12 | -3.887798955 | 3.670900184 | -1.05908599 | 0.289572093 |
| Plate: 13 | 3.361619015 | 3.670900184 | 0.915747867 | 0.359809057 |
| Plate: 14 | 2.170808907 | 3.670900184 | 0.591356016 | 0.5542879 |
| Plate: 15 | 1.95242311 | 3.670900184 | 0.531864941 | 0.59482484 |
| Plate: 16 | -5.626774521 | 1.350002686 | -4.167972835 | 3.08E-05 |
| Plate: 17 | 0.366361283 | 1.350002686 | 0.271378188 | 0.786102689 |
| Plate: 19 | -4.657819289 | 3.397432255 | -1.370982242 | 0.170394291 |
| Plate: 20 | -5.414563807 | 3.397432255 | -1.593722377 | 0.111012405 |
| Plate: 21 | -0.223771912 | 3.397432255 | -0.065865011 | 0.947485888 |
| Plate: 22 | -1.72801498 | 3.397432255 | -0.508623823 | 0.61102096 |
| Plate: 23 | -1.644080008 | 3.397432255 | -0.483918408 | 0.628448529 |
| Plate: 24 | -1.42678743 | 3.397432255 | -0.419960524 | 0.674518339 |
| Plate: 25 | 22.87469134 | 1.350002686 | 16.9441821 | 5.35E-64 |
| Plate: 26 | -0.095872012 | 1.350002686 | -0.071016164 | 0.943385528 |
| Plate: 28 | -0.525180938 | 1.350002686 | -0.389022143 | 0.697263481 |
| Plate: 29 | -0.413642737 | 1.350002686 | -0.306401418 | 0.75930189 |
| Plate: 31 | 0.969504552 | 1.350002686 | 0.718150091 | 0.472672272 |
| Plate: 32 | 0.557125075 | 1.350002686 | 0.41268442 | 0.67984181 |
| Plate: 34 | -1.622432408 | 1.350002686 | -1.201799393 | 0.229454025 |
| Plate: 35 | 0.043810796 | 1.350002686 | 0.032452377 | 0.974111584 |
| MeWo:Abiraterone Interaction | -12.0754606 | 10.70371118 | -1.12815643 | 0.259266 |
| SKMEL2:Abiraterone Interaction | 16.46217021 | 10.83065991 | 1.519960035 | 0.1285352 |
| MeWo:ABT.737 Interaction | 5.915724188 | 10.70371118 | 0.552679728 | 0.580488278 |
| SKMEL2:ABT.737 Interaction | 16.16697768 | 10.83065991 | 1.492704768 | 0.135528634 |
| UACC0257:ABT.737 Interaction | 15.41778936 | 10.64680367 | 1.448114368 | 0.147599129 |
| MeWo:Actinomycin.D Interaction | 25.88192036 | 10.70371118 | 2.418032392 | 0.015612649 |
| SKMEL2:Actinomycin.D Interaction | 60.93161182 | 10.83065991 | 5.62584481 | 1.87E-08 |
| UACC0257:Actinomycin.D Interaction | 26.85137765 | 10.64680367 | 2.522013037 | 0.011675444 |
| MeWo:Afatinib Interaction | -10.17022265 | 10.70371118 | -0.950158545 | 0.342041998 |
| SKMEL2:Afatinib Interaction | -0.768023 | 10.83065991 | -0.07091193 | 0.943468484 |
| UACC0257:Afatinib Interaction | -0.927018738 | 10.64680367 | -0.087070145 | 0.930616538 |
| MeWo:Alisertib..MLN8237. Interaction | 0.216726898 | 10.70371118 | 0.020247827 | 0.983845856 |
| SKMEL2:Alisertib..MLN8237. Interaction | 35.22047622 | 10.83065991 | 3.251923385 | 0.001147972 |
| MeWo:Allopurinol Interaction | -2.688224546 | 10.70371118 | -0.25114883 | 0.801701359 |
| SKMEL2:Allopurinol Interaction | 18.75790664 | 10.83065991 | 1.731926476 | 0.083300485 |
| UACC0257:Allopurinol Interaction | 1.882904887 | 10.64680367 | 0.176851659 | 0.859626517 |
| MeWo:Amifostine Interaction | -3.486750329 | 10.70371118 | -0.325751533 | 0.744615408 |
| MeWo:Aphrocallistin.analogue Interaction | -4.295713561 | 10.70371118 | -0.40132936 | 0.688181496 |
| SKMEL2:Aphrocallistin.analogue Interaction | 23.47433289 | 10.83065991 | 2.167396363 | 0.030215194 |
| UACC0257:Aphrocallistin.analogue Interaction | 2.198255222 | 10.64680367 | 0.206470908 | 0.836424972 |
| MeWo:Arsenic.Trioxide Interaction | -15.77417383 | 10.70371118 | -1.473710712 | 0.140573615 |
| SKMEL2:Arsenic.Trioxide Interaction | 7.36086931 | 10.83065991 | 0.679632578 | 0.496744192 |
| MeWo:Axitinib Interaction | -12.91254619 | 10.70371118 | -1.206361604 | 0.227690894 |
| SKMEL2:Axitinib Interaction | 3.54240314 | 10.83065991 | 0.327071773 | 0.743616671 |
| UACC0257:Axitinib Interaction | 2.840327769 | 10.64680367 | 0.26677751 | 0.789642939 |
| MeWo:Axitinib.1 Interaction | -16.98360461 | 10.70371118 | -1.586702436 | 0.112594235 |
| SKMEL2:Axitinib.1 Interaction | -2.91468472 | 10.83065991 | -0.269114232 | 0.787844269 |
| UACC0257:Axitinib.1 Interaction | 3.416102579 | 10.64680367 | 0.320857103 | 0.748321691 |
| MeWo:Azacitidine Interaction | 2.619508111 | 10.70371118 | 0.24472896 | 0.806668555 |
| SKMEL2:Azacitidine Interaction | 28.19563879 | 10.83065991 | 2.603316791 | 0.009238775 |
| UACC0257:Azacitidine Interaction | 8.064243617 | 10.64680367 | 0.757433298 | 0.448798318 |
| SKMEL2:Baricitinib..LY3009104..INCB028050. Interaction | 0.502421007 | 10.83065991 | 0.046388771 | 0.963000801 |
| UACC0257:Baricitinib..LY3009104..INCB028050. Interaction | 5.659824729 | 10.64680367 | 0.531598488 | 0.595009408 |
| SKMEL2:Bendamustine.HCl Interaction | -6.202021099 | 10.78594979 | -0.575009269 | 0.565290831 |
| MeWo:Bioymifi Interaction | 6.492993714 | 10.70371118 | 0.606611446 | 0.544114979 |
| SKMEL2:Bioymifi Interaction | 17.20005452 | 10.83065991 | 1.588089246 | 0.11228034 |
| UACC0257:Bioymifi Interaction | 16.06726371 | 10.64680367 | 1.509116183 | 0.131283265 |
| MeWo:Bleomycin.Sulfate Interaction | -10.0789116 | 10.70371118 | -0.941627761 | 0.346393454 |
| SKMEL2:Bleomycin.Sulfate Interaction | 6.654295105 | 10.83065991 | 0.614394244 | 0.538961108 |
| UACC0257:Bleomycin.Sulfate Interaction | 0.839129679 | 10.64680367 | 0.078815174 | 0.937180342 |
| MeWo:Bortezomib Interaction | -12.41516162 | 10.70371118 | -1.159893181 | 0.24610472 |
| SKMEL2:Bortezomib Interaction | 16.42745945 | 10.83065991 | 1.516755174 | 0.129342686 |
| UACC0257:Bortezomib Interaction | -15.97840887 | 10.64680367 | -1.500770501 | 0.133429091 |
| MeWo:Bosutinib..SKI.606. Interaction | -16.8106507 | 10.70371118 | -1.570544123 | 0.116302768 |
| SKMEL2:Bosutinib..SKI.606. Interaction | 5.658334745 | 10.83065991 | 0.522436748 | 0.601371466 |
| UACC0257:Bosutinib..SKI.606. Interaction | -1.576422021 | 10.64680367 | -0.148065285 | 0.882292585 |
| SKMEL2:Busulfan Interaction | 6.508833968 | 10.83065991 | 0.600963748 | 0.547870241 |
| MeWo:Cabazitaxel Interaction | 17.37281855 | 10.70371118 | 1.623064958 | 0.104589657 |
| SKMEL2:Cabazitaxel Interaction | 18.50135992 | 10.83065991 | 1.708239395 | 0.087605851 |
| UACC0257:Cabazitaxel Interaction | 12.27802929 | 10.64680367 | 1.153212708 | 0.248835451 |
| MeWo:Cabozantinib..XL.184. Interaction | -11.06293172 | 10.70371118 | -1.033560373 | 0.301352943 |
| SKMEL2:Cabozantinib..XL.184. Interaction | 8.183858002 | 10.83065991 | 0.755619517 | 0.449885334 |
| UACC0257:Cabozantinib..XL.184. Interaction | 2.761845899 | 10.64680367 | 0.259406108 | 0.795324313 |
| MeWo:Capecitabine Interaction | -5.715998526 | 10.84212699 | -0.527202691 | 0.598058086 |
| MeWo:Carfilzomib Interaction | 29.82996915 | 10.70371118 | 2.786880983 | 0.005326302 |
| SKMEL2:Carfilzomib Interaction | 2.300176747 | 10.83065991 | 0.212376417 | 0.831815324 |
| UACC0257:Carfilzomib Interaction | 43.70768367 | 10.64680367 | 4.105239943 | 4.05E-05 |
| MeWo:Carmustine Interaction | -7.954733927 | 10.84212699 | -0.733687581 | 0.463146884 |
| MeWo:Celecoxib Interaction | -10.55574691 | 10.84212699 | -0.973586356 | 0.330272526 |
| UACC0257:Chlorambucil Interaction | 10.74673743 | 10.64680367 | 1.009386269 | 0.312800375 |
| MeWo:Cisplatin Interaction | -4.020371529 | 10.70371118 | -0.375605382 | 0.707213862 |
| SKMEL2:Cisplatin Interaction | 3.747481577 | 10.83065991 | 0.346006763 | 0.729340897 |
| MeWo:Cladribine Interaction | -3.496384656 | 10.70371118 | -0.326651625 | 0.743934458 |
| SKMEL2:Cladribine Interaction | 39.79196019 | 10.83065991 | 3.674010682 | 0.000239332 |
| UACC0257:Cladribine Interaction | 5.321993384 | 10.64680367 | 0.499867712 | 0.617173162 |
| MeWo:Clofarabine Interaction | 8.023725678 | 10.70371118 | 0.749620906 | 0.453490965 |
| SKMEL2:Clofarabine Interaction | 39.58314231 | 10.83065991 | 3.654730428 | 0.000258043 |
| UACC0257:Clofarabine Interaction | 22.43517095 | 10.64680367 | 2.107221252 | 0.035109543 |
| MeWo:Crizotinib Interaction | 1.928057001 | 10.70371118 | 0.180129767 | 0.857052331 |
| SKMEL2:Crizotinib Interaction | 7.468948753 | 10.83065991 | 0.689611604 | 0.490445645 |
| UACC0257:Crizotinib Interaction | 13.09474951 | 10.64680367 | 1.229923076 | 0.21873889 |
| MeWo:Cytarabine.HCl...Ara.C Interaction | 3.544039546 | 10.70371118 | 0.331103809 | 0.740569186 |
| SKMEL2:Cytarabine.HCl...Ara.C Interaction | 13.94884609 | 10.83065991 | 1.28790362 | 0.197792882 |
| UACC0257:Cytarabine.HCl...Ara.C Interaction | 19.0422363 | 10.64680367 | 1.788540195 | 0.073702469 |
| MeWo:Dacarbazine Interaction | -1.894090576 | 10.70371118 | -0.176956435 | 0.859544216 |
| SKMEL2:Dacarbazine Interaction | 24.87879423 | 10.83065991 | 2.29707095 | 0.021623944 |
| MeWo:Dacomitinib..PF299804. Interaction | 3.900492089 | 10.70371118 | 0.364405581 | 0.715558625 |
| SKMEL2:Dacomitinib..PF299804. Interaction | 5.310807205 | 10.83065991 | 0.490349365 | 0.623891526 |
| UACC0257:Dacomitinib..PF299804. Interaction | -3.247030353 | 10.64680367 | -0.30497701 | 0.760386508 |
| MeWo:Dasatinib Interaction | 17.56958171 | 10.70371118 | 1.641447664 | 0.100718627 |
| SKMEL2:Dasatinib Interaction | 19.90088935 | 10.83065991 | 1.837458615 | 0.06615552 |
| UACC0257:Dasatinib Interaction | 8.369321672 | 10.64680367 | 0.786087725 | 0.431824449 |
| MeWo:Daunorubicin.HCl Interaction | 15.1389901 | 10.70371118 | 1.414368329 | 0.157267748 |
| SKMEL2:Daunorubicin.HCl Interaction | 6.425736487 | 10.83065991 | 0.593291318 | 0.552992226 |
| UACC0257:Daunorubicin.HCl Interaction | 21.97445082 | 10.64680367 | 2.063948159 | 0.039034175 |
| MeWo:Decitabine Interaction | -6.950286462 | 10.70371118 | -0.649334268 | 0.51612903 |
| SKMEL2:Decitabine Interaction | 23.22782914 | 10.83065991 | 2.144636554 | 0.031992692 |
| MeWo:Docetaxel Interaction | 8.580893138 | 10.70371118 | 0.801674577 | 0.422749769 |
| MeWo:Doxorubicin.HCl Interaction | 1.852397254 | 10.70371118 | 0.173061214 | 0.862604882 |
| SKMEL2:Doxorubicin.HCl Interaction | 20.77399223 | 10.83065991 | 1.918072621 | 0.055114562 |
| UACC0257:Doxorubicin.HCl Interaction | 7.147415791 | 10.64680367 | 0.671320334 | 0.502023439 |
| MeWo:Erlotinib.HCl Interaction | 5.560691181 | 10.70371118 | 0.519510578 | 0.603409896 |
| SKMEL2:Erlotinib.HCl Interaction | 11.44584588 | 10.83065991 | 1.056800414 | 0.290614135 |
| UACC0257:Erlotinib.HCl Interaction | 11.03893397 | 10.64680367 | 1.036830801 | 0.299825963 |
| MeWo:Etoposide Interaction | 8.290733951 | 10.70371118 | 0.774566298 | 0.438604192 |
| SKMEL2:Etoposide Interaction | 9.368964028 | 10.83065991 | 0.865040922 | 0.387025636 |
| UACC0257:Etoposide Interaction | 11.09425418 | 10.64680367 | 1.042026746 | 0.297410578 |
| MeWo:Everolimus Interaction | -22.84888491 | 10.84212699 | -2.1074172 | 0.035092568 |
| MeWo:Exemestane Interaction | -10.88313612 | 10.65846856 | -1.021078785 | 0.307228192 |
| SKMEL2:Exemestane Interaction | -4.077218592 | 10.78594979 | -0.378012013 | 0.705425268 |
| MeWo:Floxuridine Interaction | 28.15456639 | 10.70371118 | 2.630355575 | 0.008535422 |
| SKMEL2:Floxuridine Interaction | 38.06283669 | 10.83065991 | 3.514359884 | 0.000441689 |
| UACC0257:Floxuridine Interaction | 38.8317766 | 10.64680367 | 3.647270845 | 0.000265644 |
| MeWo:Fludarabine.Phosphate Interaction | 12.07121314 | 10.70371118 | 1.127759609 | 0.259433591 |
| SKMEL2:Fludarabine.Phosphate Interaction | 11.70813771 | 10.83065991 | 1.081017945 | 0.279700824 |
| MeWo:Fluorouracil...5.FU. Interaction | 2.911621806 | 10.70371118 | 0.27201984 | 0.785609283 |
| SKMEL2:Fluorouracil...5.FU. Interaction | 8.200774692 | 10.83065991 | 0.757181443 | 0.448949168 |
| UACC0257:Fluorouracil...5.FU. Interaction | 2.059618364 | 10.64680367 | 0.193449455 | 0.84660875 |
| MeWo:Flutamide..Eulexin. Interaction | -6.575213046 | 10.70371118 | -0.614292831 | 0.539028107 |
| SKMEL2:Flutamide..Eulexin. Interaction | 4.240672563 | 10.83065991 | 0.391543322 | 0.695399415 |
| UACC0257:Flutamide..Eulexin. Interaction | 10.17084361 | 10.64680367 | 0.955295498 | 0.339438641 |
| MeWo:Foretinib..GSK1363089. Interaction | -13.87445088 | 10.70371118 | -1.296228069 | 0.194910339 |
| SKMEL2:Foretinib..GSK1363089. Interaction | 3.58795825 | 10.83065991 | 0.331277898 | 0.740437698 |
| UACC0257:Foretinib..GSK1363089. Interaction | -2.537848571 | 10.64680367 | -0.238367181 | 0.811598511 |
| SKMEL2:Fulvestrant Interaction | 6.639323357 | 10.83065991 | 0.613011895 | 0.539874727 |
| UACC0257:Fulvestrant Interaction | -5.867252571 | 10.64680367 | -0.551081128 | 0.581583584 |
| MeWo:Gefitinib Interaction | -6.144907422 | 10.84212699 | -0.56676217 | 0.570881481 |
| MeWo:Gemcitabine.HCl Interaction | 26.97812256 | 10.70371118 | 2.520445675 | 0.011727554 |
| SKMEL2:Gemcitabine.HCl Interaction | 6.25961381 | 10.83065991 | 0.577953132 | 0.563301599 |
| UACC0257:Gemcitabine.HCl Interaction | 29.20833948 | 10.64680367 | 2.743390448 | 0.006085692 |
| MeWo:Ibrutinib..PCI.32765. Interaction | -8.258466741 | 10.65846856 | -0.774826768 | 0.438450247 |
| SKMEL2:Ibrutinib..PCI.32765. Interaction | 5.214365209 | 10.78594979 | 0.483440523 | 0.628787728 |
| MeWo:Imiquimod Interaction | 13.11123311 | 10.65846856 | 1.230123543 | 0.218663824 |
| SKMEL2:Imiquimod Interaction | 12.37389938 | 10.78594979 | 1.147223899 | 0.251301407 |
| MeWo:INK.128..MLN0128. Interaction | -23.08248489 | 10.70371118 | -2.156493621 | 0.031055799 |
| SKMEL2:INK.128..MLN0128. Interaction | 35.61292377 | 10.83065991 | 3.288158255 | 0.001010012 |
| UACC0257:INK.128..MLN0128. Interaction | -1.292659879 | 10.64680367 | -0.121412954 | 0.903365051 |
| MeWo:Irinotecan.HCl Interaction | -10.87602903 | 10.70371118 | -1.016098887 | 0.309593325 |
| SKMEL2:Irinotecan.HCl Interaction | 19.49629951 | 10.83065991 | 1.800102642 | 0.071857944 |
| UACC0257:Irinotecan.HCl Interaction | -5.433619706 | 10.64680367 | -0.510352203 | 0.609809789 |
| MeWo:Ixabepilone Interaction | -2.182456279 | 10.70371118 | -0.203897157 | 0.838435728 |
| SKMEL2:Ixabepilone Interaction | 2.613327708 | 10.83065991 | 0.241289795 | 0.809332739 |
| UACC0257:Ixabepilone Interaction | -9.151114871 | 10.64680367 | -0.859517575 | 0.390064269 |
| MeWo:Lapatinib Interaction | 10.86479413 | 10.65846856 | 1.0193579 | 0.308044146 |
| SKMEL2:Lapatinib Interaction | 16.59173427 | 10.78594979 | 1.5382729 | 0.123996094 |
| MeWo:LDK378 Interaction | -10.19845526 | 10.70371118 | -0.952796193 | 0.340703673 |
| SKMEL2:LDK378 Interaction | 1.963654713 | 10.83065991 | 0.181305177 | 0.856129691 |
| UACC0257:LDK378 Interaction | 4.502966929 | 10.64680367 | 0.422940731 | 0.672342583 |
| MeWo:Lenalidomide Interaction | 4.731710178 | 10.70371118 | 0.44206258 | 0.65844821 |
| SKMEL2:Lenalidomide Interaction | 11.26130509 | 10.83065991 | 1.039761676 | 0.298461913 |
| SKMEL2:Letrozole Interaction | -1.859193307 | 10.83065991 | -0.171660206 | 0.863706231 |
| MeWo:Linsitinib Interaction | 7.309179229 | 10.70371118 | 0.682864018 | 0.494699867 |
| SKMEL2:Linsitinib Interaction | 10.65915539 | 10.83065991 | 0.984164907 | 0.325045117 |
| UACC0257:Linsitinib Interaction | 14.56374389 | 10.64680367 | 1.36789823 | 0.17135772 |
| SKMEL2:Lomustine..CCNU. Interaction | 9.740118565 | 10.83065991 | 0.899309797 | 0.368497382 |
| MeWo:LY2157299 Interaction | -3.707464112 | 10.70371118 | -0.346371838 | 0.729066556 |
| SKMEL2:LY2157299 Interaction | 4.616159783 | 10.83065991 | 0.426212237 | 0.669957316 |
| MeWo:Mechlorethamine.HCl Interaction | 4.275007586 | 10.70371118 | 0.399394893 | 0.689606082 |
| SKMEL2:Mechlorethamine.HCl Interaction | 18.82769784 | 10.83065991 | 1.73837033 | 0.082159386 |
| UACC0257:Mechlorethamine.HCl Interaction | 2.215022111 | 10.64680367 | 0.208045737 | 0.835195156 |
| MeWo:Megestrol.acetate Interaction | -2.733323794 | 10.70371118 | -0.255362252 | 0.798445678 |
| SKMEL2:Megestrol.acetate Interaction | 3.406962823 | 10.83065991 | 0.314566504 | 0.753093758 |
| MeWo:MEK.162..ARRY.438162. Interaction | 5.272126826 | 10.70371118 | 0.492551297 | 0.622334518 |
| SKMEL2:MEK.162..ARRY.438162. Interaction | 6.456391078 | 10.83065991 | 0.596121671 | 0.5511 |
| UACC0257:MEK.162..ARRY.438162. Interaction | -11.84373544 | 10.64680367 | -1.112421701 | 0.265968872 |
| MeWo:Melphalan Interaction | -4.307506696 | 10.70371118 | -0.40243114 | 0.687370614 |
| SKMEL2:Melphalan Interaction | 5.095377472 | 10.83065991 | 0.470458635 | 0.638031983 |
| UACC0257:Melphalan Interaction | -5.425209663 | 10.64680367 | -0.509562291 | 0.610363191 |
| MeWo:Mercaptopurine Interaction | -5.627171209 | 10.70371118 | -0.52572151 | 0.599086945 |
| SKMEL2:Mercaptopurine Interaction | -1.023425593 | 10.83065991 | -0.094493374 | 0.924718095 |
| UACC0257:Mercaptopurine Interaction | -15.0064407 | 10.64680367 | -1.409478485 | 0.158707678 |
| MeWo:Mitomycin.C Interaction | 10.48957513 | 10.70371118 | 0.979994223 | 0.327099603 |
| SKMEL2:Mitomycin.C Interaction | 15.59245169 | 10.83065991 | 1.439658509 | 0.149978077 |
| UACC0257:Mitomycin.C Interaction | 12.94319712 | 10.64680367 | 1.215688532 | 0.224116466 |
| MeWo:Mitotane..o.p..DDD..Lysodren. Interaction | -10.42779167 | 10.70371118 | -0.97422207 | 0.329956858 |
| SKMEL2:Mitotane..o.p..DDD..Lysodren. Interaction | 18.0235985 | 10.83065991 | 1.664127455 | 0.096101014 |
| MeWo:Mitoxantrone Interaction | -0.858522941 | 10.70371118 | -0.08020797 | 0.936072572 |
| SKMEL2:Mitoxantrone Interaction | -16.4398993 | 10.83065991 | -1.517903751 | 0.129052842 |
| UACC0257:Mitoxantrone Interaction | 8.003224601 | 10.64680367 | 0.751702093 | 0.452238166 |
| UACC0257:MLN.2480 Interaction | 2.851995301 | 10.64680367 | 0.267873382 | 0.788799262 |
| MeWo:MLN4924 Interaction | -5.361899796 | 10.70371118 | -0.500938385 | 0.616419432 |
| SKMEL2:MLN4924 Interaction | -0.129124653 | 10.83065991 | -0.011922141 | 0.99048784 |
| UACC0257:MLN4924 Interaction | 2.895713896 | 10.64680367 | 0.271979646 | 0.785640188 |
| MeWo:MLN9708..MLN2238. Interaction | 12.93699115 | 10.70371118 | 1.208645387 | 0.226811929 |
| SKMEL2:MLN9708..MLN2238. Interaction | 17.75661241 | 10.83065991 | 1.639476501 | 0.101128169 |
| UACC0257:MLN9708..MLN2238. Interaction | 7.330719957 | 10.64680367 | 0.68853716 | 0.491121742 |
| MeWo:Navitoclax..ABT.263..5uM Interaction | -1.575555556 | 10.70371118 | -0.147197129 | 0.882977758 |
| SKMEL2:Navitoclax..ABT.263..5uM Interaction | 11.98918626 | 10.83065991 | 1.106967292 | 0.268320013 |
| UACC0257:Navitoclax..ABT.263..5uM Interaction | -5.262262673 | 10.64680367 | -0.49425751 | 0.621129198 |
| MeWo:Nelarabine Interaction | -1.508597982 | 10.70371118 | -0.140941582 | 0.887917366 |
| SKMEL2:Nelarabine Interaction | 22.51184609 | 10.83065991 | 2.078529498 | 0.037672025 |
| UACC0257:Nelarabine Interaction | 2.863804272 | 10.64680367 | 0.268982538 | 0.787945609 |
| MeWo:OSI.027 Interaction | 14.51848747 | 10.70371118 | 1.356397535 | 0.174986452 |
| SKMEL2:OSI.027 Interaction | 17.58897985 | 10.83065991 | 1.623998907 | 0.104390178 |
| UACC0257:OSI.027 Interaction | 21.5447067 | 10.64680367 | 2.023584484 | 0.043024828 |
| MeWo:Oxaliplatin Interaction | -4.747171458 | 10.65846856 | -0.445389638 | 0.656042523 |
| SKMEL2:Oxaliplatin Interaction | -0.957277087 | 10.78594979 | -0.088752229 | 0.929279621 |
| MeWo:Paclitaxel Interaction | -4.982972577 | 10.70371118 | -0.465536905 | 0.641551562 |
| SKMEL2:Paclitaxel Interaction | 13.03024544 | 10.83065991 | 1.203088783 | 0.22895474 |
| MeWo:Palbociclib..PD.0332991..Isethionate Interaction | -3.282147612 | 10.70371118 | -0.306636414 | 0.759122997 |
| SKMEL2:Palbociclib..PD.0332991..Isethionate Interaction | 4.733302273 | 10.83065991 | 0.437028059 | 0.662095232 |
| UACC0257:Palbociclib..PD.0332991..Isethionate Interaction | -3.288784454 | 10.64680367 | -0.30889876 | 0.757401426 |
| MeWo:Pazopanib.HCl Interaction | -11.48157198 | 10.70371118 | -1.072672065 | 0.283429888 |
| SKMEL2:Pazopanib.HCl Interaction | 16.66255773 | 10.83065991 | 1.538461911 | 0.123949905 |
| UACC0257:Pazopanib.HCl Interaction | -10.96583527 | 10.64680367 | -1.029965013 | 0.303037603 |
| MeWo:PD325901 Interaction | 42.64997326 | 10.70371118 | 3.984596794 | 6.78E-05 |
| SKMEL2:PD325901 Interaction | 55.55896967 | 10.83065991 | 5.129786196 | 2.93E-07 |
| UACC0257:PD325901 Interaction | 13.09264743 | 10.64680367 | 1.229725638 | 0.218812839 |
| SKMEL2:Pemetrexed Interaction | 2.325097988 | 10.83065991 | 0.214677407 | 0.830020802 |
| MeWo:Plicamycin Interaction | -0.697185987 | 10.70371118 | -0.065134978 | 0.948067115 |
| SKMEL2:Plicamycin Interaction | 5.903481123 | 10.83065991 | 0.545071231 | 0.585709981 |
| UACC0257:Plicamycin Interaction | -0.998095879 | 10.64680367 | -0.093746059 | 0.925311724 |
| MeWo:Pralatrexate Interaction | 14.30501039 | 10.65846856 | 1.342126246 | 0.179568732 |
| SKMEL2:Pralatrexate Interaction | 56.2845597 | 10.78594979 | 5.218322058 | 1.82E-07 |
| MeWo:Quinacrine.HCl Interaction | 6.712399641 | 10.70371118 | 0.627109563 | 0.530593841 |
| SKMEL2:Quinacrine.HCl Interaction | 8.938667968 | 10.83065991 | 0.825311481 | 0.409203605 |
| UACC0257:Quinacrine.HCl Interaction | -1.976581084 | 10.64680367 | -0.185650186 | 0.852720783 |
| MeWo:Quizartinib Interaction | -1.708408662 | 10.70371118 | -0.159609002 | 0.873190533 |
| SKMEL2:Quizartinib Interaction | 12.0687457 | 10.83065991 | 1.114313053 | 0.265156921 |
| UACC0257:Quizartinib Interaction | 13.89415606 | 10.64680367 | 1.305007258 | 0.191903844 |
| MeWo:Raloxifene Interaction | -10.08421234 | 10.65846856 | -0.946122071 | 0.344096581 |
| SKMEL2:Raloxifene Interaction | -5.457154641 | 10.78594979 | -0.50595031 | 0.612896535 |
| MeWo:Romidepsin Interaction | -15.94514725 | 10.70371118 | -1.489683996 | 0.13632149 |
| SKMEL2:Romidepsin Interaction | 14.99448357 | 10.83065991 | 1.384447827 | 0.166235228 |
| UACC0257:Romidepsin Interaction | 14.09319433 | 10.64680367 | 1.323701909 | 0.185615636 |
| MeWo:Sabutoclax..BI.97C1. Interaction | -14.94171625 | 10.70371118 | -1.395937912 | 0.162747092 |
| SKMEL2:Sabutoclax..BI.97C1. Interaction | 16.07417345 | 10.83065991 | 1.484136109 | 0.137786952 |
| UACC0257:Sabutoclax..BI.97C1. Interaction | -9.817861338 | 10.64680367 | -0.922141672 | 0.356464661 |
| MeWo:Sirolimus..Rapamycin. Interaction | -17.05384195 | 10.70371118 | -1.593264397 | 0.111115065 |
| SKMEL2:Sirolimus..Rapamycin. Interaction | 6.446291033 | 10.83065991 | 0.595189129 | 0.551723097 |
| MeWo:Sorafenib Interaction | -7.392648759 | 10.70371118 | -0.690662204 | 0.489785036 |
| UACC0257:Sorafenib Interaction | -4.601878621 | 10.64680367 | -0.432231002 | 0.665577709 |
| MeWo:Streptozocin Interaction | -4.982359496 | 10.70371118 | -0.465479628 | 0.641592569 |
| SKMEL2:Streptozocin Interaction | 19.68009449 | 10.83065991 | 1.81707252 | 0.069219428 |
| UACC0257:Streptozocin Interaction | -3.220607123 | 10.64680367 | -0.302495211 | 0.762277405 |
| MeWo:Sunitinib Interaction | 7.838798288 | 10.70371118 | 0.732343965 | 0.463966349 |
| UACC0257:Sunitinib Interaction | -0.083584321 | 10.64680367 | -0.007850649 | 0.993736223 |
| MeWo:Tamoxifen.Citrate Interaction | -10.60468125 | 10.70371118 | -0.990748074 | 0.321819362 |
| SKMEL2:Tamoxifen.Citrate Interaction | 18.76494201 | 10.83065991 | 1.732576055 | 0.083184877 |
| UACC0257:Tamoxifen.Citrate Interaction | 3.842866696 | 10.64680367 | 0.360940881 | 0.718147062 |
| MeWo:Temozolomide Interaction | -11.15126786 | 10.84212699 | -1.028512935 | 0.303719767 |
| SKMEL2:Temsirolimus..CCI.779..Torisel. Interaction | 3.747595225 | 10.83065991 | 0.346017256 | 0.729333011 |
| MeWo:Teniposide Interaction | 20.84072367 | 10.70371118 | 1.947055868 | 0.051540576 |
| SKMEL2:Teniposide Interaction | 23.623041 | 10.83065991 | 2.181126654 | 0.029184462 |
| UACC0257:Teniposide Interaction | 17.68469134 | 10.64680367 | 1.661032916 | 0.096720896 |
| MeWo:Thioguanine Interaction | -8.935725471 | 10.70371118 | -0.83482498 | 0.403825204 |
| SKMEL2:Thioguanine Interaction | 23.07618942 | 10.83065991 | 2.130635587 | 0.033130087 |
| UACC0257:Thioguanine Interaction | 1.988272336 | 10.64680367 | 0.186748286 | 0.851859694 |
| MeWo:Thiotepa Interaction | 3.890974336 | 10.70371118 | 0.363516379 | 0.716222626 |
| SKMEL2:Thiotepa Interaction | 9.639853538 | 10.83065991 | 0.89005228 | 0.373447418 |
| UACC0257:Thiotepa Interaction | -2.380227689 | 10.64680367 | -0.223562655 | 0.823099674 |
| MeWo:Topotecan.HCl Interaction | -7.877592882 | 10.70371118 | -0.735968371 | 0.461757689 |
| SKMEL2:Topotecan.HCl Interaction | 5.549336998 | 10.83065991 | 0.512372934 | 0.608395105 |
| UACC0257:Topotecan.HCl Interaction | 6.634718407 | 10.64680367 | 0.623165282 | 0.533182288 |
| MeWo:Trametinib..GSK1120212. Interaction | 28.92257116 | 10.70371118 | 2.702106836 | 0.006895375 |
| UACC0257:Trametinib..GSK1120212. Interaction | 9.706321806 | 10.64680367 | 0.911665333 | 0.361954775 |
| MeWo:Triethylenemelamine Interaction | -2.403147896 | 10.70371118 | -0.22451539 | 0.82235835 |
| SKMEL2:Triethylenemelamine Interaction | 2.46990973 | 10.83065991 | 0.228047945 | 0.819611059 |
| UACC0257:Triethylenemelamine Interaction | -7.265381988 | 10.64680367 | -0.682400297 | 0.494992957 |
| MeWo:Uracil.mustard Interaction | -10.40465093 | 10.70371118 | -0.972060134 | 0.331031179 |
| SKMEL2:Uracil.mustard Interaction | -0.413746533 | 10.83065991 | -0.038201415 | 0.969527435 |
| MeWo:Valrubicin Interaction | 2.869858038 | 10.70371118 | 0.268118038 | 0.788610942 |
| SKMEL2:Valrubicin Interaction | 3.298316115 | 10.83065991 | 0.304535102 | 0.760723097 |
| UACC0257:Valrubicin Interaction | 3.786817996 | 10.64680367 | 0.355676512 | 0.722086202 |
| MeWo:Vandetanib Interaction | 8.983421609 | 10.65846856 | 0.842843562 | 0.399324982 |
| SKMEL2:Vandetanib Interaction | 9.532341373 | 10.78594979 | 0.883773943 | 0.376827785 |
| SKMEL2:Vemurafenib Interaction | 22.65823235 | 10.83065991 | 2.092045411 | 0.036445735 |
| UACC0257:Vemurafenib Interaction | -13.05776373 | 10.64680367 | -1.22644919 | 0.220042637 |
| SKMEL2:Vinblastine.Sulfate Interaction | -9.649932387 | 10.83065991 | -0.890982864 | 0.372947978 |
| UACC0257:Vinblastine.Sulfate Interaction | -0.871620021 | 10.64680367 | -0.081866826 | 0.93475335 |
| MeWo:Vincristine.Sulfate Interaction | -10.36579234 | 10.70371118 | -0.968429749 | 0.332840293 |
| SKMEL2:Vincristine.Sulfate Interaction | -4.565184508 | 10.83065991 | -0.421505665 | 0.673389939 |
| UACC0257:Vincristine.Sulfate Interaction | -22.48169082 | 10.64680367 | -2.111590627 | 0.034732673 |
| MeWo:Vinorelbine.Tartrate Interaction | -5.077908606 | 10.70371118 | -0.474406355 | 0.635214808 |
| SKMEL2:Vinorelbine.Tartrate Interaction | 6.815604817 | 10.83065991 | 0.629288047 | 0.529166943 |
| UACC0257:Vinorelbine.Tartrate Interaction | -5.526994113 | 10.64680367 | -0.519122385 | 0.603680552 |
| MeWo:Vismodegib Interaction | -8.848086212 | 10.70371118 | -0.826637235 | 0.408451552 |
| SKMEL2:Vismodegib Interaction | 18.56248252 | 10.83065991 | 1.713882873 | 0.086564138 |
| UACC0257:Vismodegib Interaction | -6.071714441 | 10.64680367 | -0.570285189 | 0.568490031 |
| MeWo:Vorinostat Interaction | -0.926038194 | 10.70371118 | -0.086515619 | 0.931057318 |
| SKMEL2:Vorinostat Interaction | 15.66988571 | 10.83065991 | 1.446808029 | 0.147964756 |
| UACC0257:Vorinostat Interaction | -18.42126694 | 10.64680367 | -1.730215708 | 0.083605581 |
| MeWo:Zoledronic.Acid Interaction | -9.689047892 | 10.84212699 | -0.893648257 | 0.371519764 |
| MeWo:Ldose: -1.397940009 (uM) Interaction | -0.447566913 | 3.23078877 | -0.138531778 | 0.889821407 |
| SKMEL2:Ldose: -1.397940009 (uM) Interaction | -1.193165768 | 3.988172242 | -0.299176088 | 0.764808482 |
| UACC0257:Ldose: -1.397940009 (uM) Interaction | -0.658994365 | 2.829838583 | -0.232873482 | 0.815861788 |
| MeWo:Ldose: -1 (uM) Interaction | -3.644685808 | 3.23078877 | -1.128110213 | 0.259285515 |
| SKMEL2:Ldose: -1 (uM) Interaction | -3.219720377 | 3.988172242 | -0.807317283 | 0.419492322 |
| UACC0257:Ldose: -1 (uM) Interaction | -2.386747654 | 2.829838583 | -0.843421836 | 0.399001612 |
| MeWo:Ldose: -0.698970004 (uM) Interaction | 0.204904992 | 3.23078877 | 0.06342259 | 0.949430568 |
| SKMEL2:Ldose: -0.698970004 (uM) Interaction | -0.346686838 | 3.988172242 | -0.086928753 | 0.930728925 |
| UACC0257:Ldose: -0.698970004 (uM) Interaction | -1.202774587 | 2.829838583 | -0.425032931 | 0.670816771 |
| MeWo:Ldose: -0.397940009 (uM) Interaction | 0.436712794 | 3.23078877 | 0.13517219 | 0.892476955 |
| SKMEL2:Ldose: -0.397940009 (uM) Interaction | -0.814262803 | 3.988172242 | -0.204169417 | 0.838222973 |
| UACC0257:Ldose: -0.397940009 (uM) Interaction | 1.056996686 | 2.829838583 | 0.373518367 | 0.708766229 |
| MeWo:Ldose: 0 (uM) Interaction | -3.865722384 | 3.23078877 | -1.196525883 | 0.231504127 |
| SKMEL2:Ldose: 0 (uM) Interaction | -2.811814302 | 3.988172242 | -0.705038331 | 0.480793747 |
| UACC0257:Ldose: 0 (uM) Interaction | -1.36946239 | 2.829838583 | -0.483936575 | 0.628435636 |
| MeWo:Ldose: 0.301029996 (uM) Interaction | -3.623806326 | 3.23078877 | -1.121647556 | 0.262024403 |
| SKMEL2:Ldose: 0.301029996 (uM) Interaction | 4.444371734 | 3.988172242 | 1.114388112 | 0.265124734 |
| UACC0257:Ldose: 0.301029996 (uM) Interaction | -4.345045694 | 2.829838583 | -1.535439413 | 0.124690118 |
| Abiraterone:Ldose: -1.397940009 (uM) Interaction | -3.41690003 | 10.69209565 | -0.319572527 | 0.749295397 |
| ABT.737:Ldose: -1.397940009 (uM) Interaction | 4.934124104 | 10.69209565 | 0.461473996 | 0.644463076 |
| Actinomycin.D:Ldose: -1.397940009 (uM) Interaction | -18.11748461 | 10.69209565 | -1.69447461 | 0.090189098 |
| Afatinib:Ldose: -1.397940009 (uM) Interaction | -10.74260485 | 10.69209565 | -1.004723975 | 0.315040684 |
| Alisertib..MLN8237.:Ldose: -1.397940009 (uM) Interaction | -9.625929511 | 10.69209565 | -0.900284643 | 0.367978515 |
| Allopurinol:Ldose: -1.397940009 (uM) Interaction | -3.640892063 | 10.69209565 | -0.340521838 | 0.733466788 |
| Amifostine:Ldose: -1.397940009 (uM) Interaction | -1.292212913 | 10.69209565 | -0.120856842 | 0.903805516 |
| Aphrocallistin.analogue:Ldose: -1.397940009 (uM) Interaction | -8.941094303 | 10.69209565 | -0.836234036 | 0.403032216 |
| Arsenic.Trioxide:Ldose: -1.397940009 (uM) Interaction | -12.37980236 | 10.69209565 | -1.157846204 | 0.246939207 |
| Axitinib:Ldose: -1.397940009 (uM) Interaction | -6.950928706 | 10.69209565 | -0.65009975 | 0.515634489 |
| Axitinib.1:Ldose: -1.397940009 (uM) Interaction | -7.470474563 | 10.69209565 | -0.698691333 | 0.484752239 |
| Azacitidine:Ldose: -1.397940009 (uM) Interaction | 2.16936902 | 10.69209565 | 0.202894651 | 0.839219226 |
| Baricitinib..LY3009104..INCB028050.:Ldose: -1.397940009 (uM) Interaction | -9.34098153 | 10.69209565 | -0.873634303 | 0.382326848 |
| Bendamustine.HCl:Ldose: -1.397940009 (uM) Interaction | -8.25885754 | 10.60131818 | -0.779040625 | 0.435964052 |
| BGJ398..NVPBGJ398.:Ldose: -1.397940009 (uM) Interaction | -6.83755604 | 10.69209565 | -0.63949634 | 0.522506664 |
| Bioymifi:Ldose: -1.397940009 (uM) Interaction | 2.219038064 | 10.69209565 | 0.20754005 | 0.835590014 |
| Bleomycin.Sulfate:Ldose: -1.397940009 (uM) Interaction | -4.629351896 | 10.69209565 | -0.432969555 | 0.665041076 |
| Bortezomib:Ldose: -1.397940009 (uM) Interaction | -21.62894529 | 10.69209565 | -2.022891115 | 0.043096286 |
| Bosutinib..SKI.606.:Ldose: -1.397940009 (uM) Interaction | -11.5025521 | 10.69209565 | -1.075799588 | 0.282028537 |
| Busulfan:Ldose: -1.397940009 (uM) Interaction | -5.195839313 | 10.69209565 | -0.485951443 | 0.627006369 |
| Cabazitaxel:Ldose: -1.397940009 (uM) Interaction | -3.964258246 | 10.69209565 | -0.370765318 | 0.710815858 |
| Cabozantinib..XL.184.:Ldose: -1.397940009 (uM) Interaction | -6.055461746 | 10.69209565 | -0.56634938 | 0.571161999 |
| Capecitabine:Ldose: -1.397940009 (uM) Interaction | -0.637506866 | 10.96747366 | -0.058127048 | 0.953647951 |
| Carboplatin:Ldose: -1.397940009 (uM) Interaction | 1.458037891 | 10.96747366 | 0.132942001 | 0.89424045 |
| Carfilzomib:Ldose: -1.397940009 (uM) Interaction | -36.0331522 | 10.69209565 | -3.370073873 | 0.000752754 |
| Carmustine:Ldose: -1.397940009 (uM) Interaction | 3.692487038 | 10.96747366 | 0.336676171 | 0.736364197 |
| Celecoxib:Ldose: -1.397940009 (uM) Interaction | 2.735210786 | 10.96747366 | 0.249392966 | 0.803059123 |
| Chlorambucil:Ldose: -1.397940009 (uM) Interaction | 2.115490854 | 10.69209565 | 0.197855586 | 0.843159858 |
| Cisplatin:Ldose: -1.397940009 (uM) Interaction | -8.835005396 | 10.69209565 | -0.826311856 | 0.408636052 |
| Cladribine:Ldose: -1.397940009 (uM) Interaction | -9.186913143 | 10.69209565 | -0.859224744 | 0.390225772 |
| Clofarabine:Ldose: -1.397940009 (uM) Interaction | -28.32901991 | 10.69209565 | -2.649529226 | 0.008066084 |
| Crizotinib:Ldose: -1.397940009 (uM) Interaction | -2.355678033 | 10.69209565 | -0.220319581 | 0.825624289 |
| Cytarabine.HCl...Ara.C:Ldose: -1.397940009 (uM) Interaction | -5.300239105 | 10.69209565 | -0.495715646 | 0.620099932 |
| Dacarbazine:Ldose: -1.397940009 (uM) Interaction | -10.79633433 | 10.69209565 | -1.009749135 | 0.312626453 |
| Dacomitinib..PF299804.:Ldose: -1.397940009 (uM) Interaction | 3.543156005 | 10.69209565 | 0.331380874 | 0.740359924 |
| Dasatinib:Ldose: -1.397940009 (uM) Interaction | 5.891430267 | 10.69209565 | 0.551008003 | 0.58163371 |
| Daunorubicin.HCl:Ldose: -1.397940009 (uM) Interaction | -17.57119918 | 10.69209565 | -1.643382154 | 0.10031799 |
| Decitabine:Ldose: -1.397940009 (uM) Interaction | -1.521920045 | 10.69209565 | -0.142340669 | 0.886812211 |
| Dexrazoxane:Ldose: -1.397940009 (uM) Interaction | 0.65595115 | 10.96747366 | 0.059808774 | 0.952308474 |
| Docetaxel:Ldose: -1.397940009 (uM) Interaction | -7.133362026 | 10.69209565 | -0.667162197 | 0.504675439 |
| Doxorubicin.HCl:Ldose: -1.397940009 (uM) Interaction | -13.06466781 | 10.69209565 | -1.221899638 | 0.2217585 |
| Erlotinib.HCl:Ldose: -1.397940009 (uM) Interaction | 6.658556347 | 10.69209565 | 0.62275503 | 0.533451883 |
| Etoposide:Ldose: -1.397940009 (uM) Interaction | -7.851616911 | 10.69209565 | -0.734338447 | 0.462750214 |
| Everolimus:Ldose: -1.397940009 (uM) Interaction | 1.184598204 | 10.96747366 | 0.108010125 | 0.913988632 |
| Exemestane:Ldose: -1.397940009 (uM) Interaction | -4.441484157 | 10.60131818 | -0.418955839 | 0.675252442 |
| Floxuridine:Ldose: -1.397940009 (uM) Interaction | -20.94790476 | 10.69209565 | -1.959195413 | 0.050102349 |
| Fludarabine.Phosphate:Ldose: -1.397940009 (uM) Interaction | 9.896828575 | 10.69209565 | 0.925621029 | 0.354652979 |
| Fluorouracil...5.FU.:Ldose: -1.397940009 (uM) Interaction | -2.945214761 | 10.69209565 | -0.275457203 | 0.782967554 |
| Flutamide..Eulexin.:Ldose: -1.397940009 (uM) Interaction | -5.550994813 | 10.69209565 | -0.519168084 | 0.603648687 |
| Foretinib..GSK1363089.:Ldose: -1.397940009 (uM) Interaction | -6.132181496 | 10.69209565 | -0.573524751 | 0.56629523 |
| Fulvestrant:Ldose: -1.397940009 (uM) Interaction | 5.279189387 | 10.69209565 | 0.493746929 | 0.621489782 |
| Gefitinib:Ldose: -1.397940009 (uM) Interaction | -3.324510447 | 10.96747366 | -0.303124543 | 0.761797779 |
| Gemcitabine.HCl:Ldose: -1.397940009 (uM) Interaction | -1.657572345 | 10.69209565 | -0.155027826 | 0.876800784 |
| Ibrutinib..PCI.32765.:Ldose: -1.397940009 (uM) Interaction | 0.456673343 | 10.60131818 | 0.043077034 | 0.965640512 |
| Imatinib:Ldose: -1.397940009 (uM) Interaction | -0.779975256 | 10.96747366 | -0.07111713 | 0.943305172 |
| Imiquimod:Ldose: -1.397940009 (uM) Interaction | 11.33717613 | 10.60131818 | 1.069411929 | 0.284895672 |
| INK.128..MLN0128.:Ldose: -1.397940009 (uM) Interaction | -16.96690178 | 10.69209565 | -1.58686401 | 0.112557628 |
| Irinotecan.HCl:Ldose: -1.397940009 (uM) Interaction | -6.16138243 | 10.69209565 | -0.576255828 | 0.564448092 |
| Ixabepilone:Ldose: -1.397940009 (uM) Interaction | -20.82228221 | 10.69209565 | -1.947446309 | 0.051493786 |
| Lapatinib:Ldose: -1.397940009 (uM) Interaction | 12.83833631 | 10.60131818 | 1.211013205 | 0.22590318 |
| LDK378:Ldose: -1.397940009 (uM) Interaction | -11.0216785 | 10.69209565 | -1.030824906 | 0.302634118 |
| Lenalidomide:Ldose: -1.397940009 (uM) Interaction | 7.53167566 | 10.69209565 | 0.70441529 | 0.481181544 |
| Letrozole:Ldose: -1.397940009 (uM) Interaction | -4.7411576 | 10.69209565 | -0.443426411 | 0.657461639 |
| Linsitinib:Ldose: -1.397940009 (uM) Interaction | 2.266097637 | 10.69209565 | 0.211941392 | 0.832154695 |
| Lomustine..CCNU.:Ldose: -1.397940009 (uM) Interaction | 1.978969004 | 10.69209565 | 0.185087103 | 0.853162401 |
| LY2157299:Ldose: -1.397940009 (uM) Interaction | -3.438687113 | 10.69209565 | -0.321610209 | 0.747751024 |
| Mechlorethamine.HCl:Ldose: -1.397940009 (uM) Interaction | 0.859839519 | 10.69209565 | 0.08041824 | 0.935905342 |
| Megestrol.acetate:Ldose: -1.397940009 (uM) Interaction | -2.917524928 | 10.69209565 | -0.272867455 | 0.784957632 |
| MEK.162..ARRY.438162.:Ldose: -1.397940009 (uM) Interaction | -20.03603565 | 10.69209565 | -1.873911 | 0.060955761 |
| Melphalan:Ldose: -1.397940009 (uM) Interaction | -4.346682013 | 10.69209565 | -0.406532279 | 0.684355446 |
| Mercaptopurine:Ldose: -1.397940009 (uM) Interaction | -3.167732276 | 10.69209565 | -0.296268606 | 0.767027718 |
| Methotrexate:Ldose: -1.397940009 (uM) Interaction | -33.5948651 | 10.69209565 | -3.142028111 | 0.001680015 |
| Mitomycin.C:Ldose: -1.397940009 (uM) Interaction | 8.405777327 | 10.69209565 | 0.786167427 | 0.431777761 |
| Mitotane..o.p..DDD..Lysodren.:Ldose: -1.397940009 (uM) Interaction | -4.60963574 | 10.69209565 | -0.431125561 | 0.666381244 |
| Mitoxantrone:Ldose: -1.397940009 (uM) Interaction | -31.71664521 | 10.69209565 | -2.966363774 | 0.003016633 |
| MLN.2480:Ldose: -1.397940009 (uM) Interaction | -2.431475775 | 10.69209565 | -0.227408719 | 0.820108026 |
| MLN4924:Ldose: -1.397940009 (uM) Interaction | -7.414586663 | 10.69209565 | -0.693464303 | 0.488025444 |
| MLN9708..MLN2238.:Ldose: -1.397940009 (uM) Interaction | 9.683891015 | 10.69209565 | 0.90570561 | 0.365101479 |
| Navitoclax..ABT.263..5uM:Ldose: -1.397940009 (uM) Interaction | -2.996312896 | 10.69209565 | -0.28023626 | 0.779298849 |
| Nelarabine:Ldose: -1.397940009 (uM) Interaction | 0.755704487 | 10.69209565 | 0.0706788 | 0.943654028 |
| OSI.027:Ldose: -1.397940009 (uM) Interaction | 12.68802599 | 10.69209565 | 1.186673446 | 0.235369097 |
| Oxaliplatin:Ldose: -1.397940009 (uM) Interaction | -5.902167174 | 10.60131818 | -0.55673899 | 0.577711362 |
| Paclitaxel:Ldose: -1.397940009 (uM) Interaction | -18.09284044 | 10.69209565 | -1.692169714 | 0.090627592 |
| Palbociclib..PD.0332991..Isethionate:Ldose: -1.397940009 (uM) Interaction | -1.610107923 | 10.69209565 | -0.150588619 | 0.880301602 |
| Pazopanib.HCl:Ldose: -1.397940009 (uM) Interaction | -2.744734746 | 10.69209565 | -0.256706902 | 0.797407412 |
| PD325901:Ldose: -1.397940009 (uM) Interaction | -11.15025031 | 10.69209565 | -1.042849847 | 0.297029148 |
| Pemetrexed:Ldose: -1.397940009 (uM) Interaction | -5.74173658 | 10.69209565 | -0.537007596 | 0.591267726 |
| Pipobroman:Ldose: -1.397940009 (uM) Interaction | -4.473278662 | 10.96747366 | -0.407867737 | 0.683374697 |
| Plicamycin:Ldose: -1.397940009 (uM) Interaction | -13.48967257 | 10.69209565 | -1.261649074 | 0.207088288 |
| Pralatrexate:Ldose: -1.397940009 (uM) Interaction | -1.393939177 | 10.60131818 | -0.131487345 | 0.895390984 |
| Quinacrine.HCl:Ldose: -1.397940009 (uM) Interaction | 4.89471739 | 10.69209565 | 0.457788403 | 0.64710893 |
| Quizartinib:Ldose: -1.397940009 (uM) Interaction | 0.868151804 | 10.69209565 | 0.081195664 | 0.935287077 |
| Raloxifene:Ldose: -1.397940009 (uM) Interaction | -11.00179426 | 10.60131818 | -1.037776064 | 0.299385577 |
| Romidepsin:Ldose: -1.397940009 (uM) Interaction | -9.415837015 | 10.69209565 | -0.880635314 | 0.37852473 |
| Sabutoclax..BI.97C1.:Ldose: -1.397940009 (uM) Interaction | -6.901297516 | 10.69209565 | -0.645457892 | 0.51863714 |
| Sirolimus..Rapamycin.:Ldose: -1.397940009 (uM) Interaction | 2.335794822 | 10.69209565 | 0.218459963 | 0.827072749 |
| Sorafenib:Ldose: -1.397940009 (uM) Interaction | -4.014509426 | 10.69209565 | -0.375465162 | 0.707318123 |
| Streptozocin:Ldose: -1.397940009 (uM) Interaction | -2.143542713 | 10.69209565 | -0.200479194 | 0.841107657 |
| Sunitinib:Ldose: -1.397940009 (uM) Interaction | 9.826378687 | 10.69209565 | 0.91903206 | 0.358088746 |
| Tamoxifen.Citrate:Ldose: -1.397940009 (uM) Interaction | -7.19594963 | 10.69209565 | -0.67301583 | 0.500944195 |
| Temozolomide:Ldose: -1.397940009 (uM) Interaction | -2.883677357 | 10.96747366 | -0.262929955 | 0.792606997 |
| Temsirolimus..CCI.779..Torisel.:Ldose: -1.397940009 (uM) Interaction | -5.458372116 | 10.69209565 | -0.510505358 | 0.609702516 |
| Teniposide:Ldose: -1.397940009 (uM) Interaction | -9.81964733 | 10.69209565 | -0.918402496 | 0.358418119 |
| Thioguanine:Ldose: -1.397940009 (uM) Interaction | -4.501447971 | 10.69209565 | -0.42100708 | 0.67375397 |
| Thiotepa:Ldose: -1.397940009 (uM) Interaction | 8.241341485 | 10.69209565 | 0.77078823 | 0.440840632 |
| Topotecan.HCl:Ldose: -1.397940009 (uM) Interaction | -22.60060062 | 10.69209565 | -2.113767156 | 0.034546234 |
| Trametinib..GSK1120212.:Ldose: -1.397940009 (uM) Interaction | -3.571245775 | 10.69209565 | -0.334008027 | 0.738376643 |
| Tretinoin:Ldose: -1.397940009 (uM) Interaction | 2.175453563 | 10.96747366 | 0.19835503 | 0.842769108 |
| Triethylenemelamine:Ldose: -1.397940009 (uM) Interaction | 0.517617454 | 10.69209565 | 0.048411226 | 0.961388947 |
| Uracil.mustard:Ldose: -1.397940009 (uM) Interaction | -6.002571896 | 10.69209565 | -0.56140275 | 0.574528653 |
| Valrubicin:Ldose: -1.397940009 (uM) Interaction | 3.703478589 | 10.69209565 | 0.34637537 | 0.729063901 |
| Vandetanib:Ldose: -1.397940009 (uM) Interaction | 7.774647276 | 10.60131818 | 0.733366091 | 0.463342885 |
| Vemurafenib:Ldose: -1.397940009 (uM) Interaction | -13.73423293 | 10.69209565 | -1.284522079 | 0.198972689 |
| Vinblastine.Sulfate:Ldose: -1.397940009 (uM) Interaction | -8.984957613 | 10.69209565 | -0.840336442 | 0.400728786 |
| Vincristine.Sulfate:Ldose: -1.397940009 (uM) Interaction | -25.72043286 | 10.69209565 | -2.405555815 | 0.016155976 |
| Vinorelbine.Tartrate:Ldose: -1.397940009 (uM) Interaction | -2.572131321 | 10.69209565 | -0.240563815 | 0.80989541 |
| Vismodegib:Ldose: -1.397940009 (uM) Interaction | -1.90333353 | 10.69209565 | -0.178013141 | 0.858714273 |
| Vorinostat:Ldose: -1.397940009 (uM) Interaction | 2.788031587 | 10.69209565 | 0.260756327 | 0.794282834 |
| Zoledronic.Acid:Ldose: -1.397940009 (uM) Interaction | 0.803472616 | 10.96747366 | 0.073259589 | 0.941600205 |
| Abiraterone:Ldose: -1 (uM) Interaction | -1.485314499 | 10.69209565 | -0.138917061 | 0.889516944 |
| ABT.737:Ldose: -1 (uM) Interaction | 5.386807717 | 10.69209565 | 0.503812152 | 0.614398365 |
| Actinomycin.D:Ldose: -1 (uM) Interaction | -31.33467047 | 10.69209565 | -2.93063881 | 0.00338609 |
| Afatinib:Ldose: -1 (uM) Interaction | -7.302797523 | 10.69209565 | -0.683008997 | 0.494608254 |
| Alisertib..MLN8237.:Ldose: -1 (uM) Interaction | -12.3431063 | 10.69209565 | -1.15441413 | 0.248342798 |
| Allopurinol:Ldose: -1 (uM) Interaction | 5.080187384 | 10.69209565 | 0.475134861 | 0.634695506 |
| Amifostine:Ldose: -1 (uM) Interaction | 1.814716967 | 10.69209565 | 0.169725097 | 0.865227876 |
| Aphrocallistin.analogue:Ldose: -1 (uM) Interaction | -17.05905127 | 10.69209565 | -1.595482479 | 0.110618558 |
| Arsenic.Trioxide:Ldose: -1 (uM) Interaction | -6.480218116 | 10.69209565 | -0.606075584 | 0.544470733 |
| Axitinib:Ldose: -1 (uM) Interaction | -3.229835493 | 10.69209565 | -0.302076936 | 0.762596232 |
| Axitinib.1:Ldose: -1 (uM) Interaction | -6.237052299 | 10.69209565 | -0.583333006 | 0.559675048 |
| Azacitidine:Ldose: -1 (uM) Interaction | 3.513719091 | 10.69209565 | 0.328627727 | 0.742440174 |
| Baricitinib..LY3009104..INCB028050.:Ldose: -1 (uM) Interaction | -3.934737266 | 10.69209565 | -0.368004308 | 0.712873515 |
| Bendamustine.HCl:Ldose: -1 (uM) Interaction | -14.08893981 | 10.60131818 | -1.328979998 | 0.183868169 |
| BGJ398..NVPBGJ398.:Ldose: -1 (uM) Interaction | -3.326614266 | 10.69209565 | -0.311128368 | 0.755705945 |
| Bioymifi:Ldose: -1 (uM) Interaction | 6.093214277 | 10.69209565 | 0.569880263 | 0.568764654 |
| Bleomycin.Sulfate:Ldose: -1 (uM) Interaction | -14.48134193 | 10.69209565 | -1.354396968 | 0.17562349 |
| Bortezomib:Ldose: -1 (uM) Interaction | -17.63219075 | 10.69209565 | -1.649086515 | 0.099144 |
| Bosutinib..SKI.606.:Ldose: -1 (uM) Interaction | -11.21762962 | 10.69209565 | -1.049151634 | 0.294119707 |
| Busulfan:Ldose: -1 (uM) Interaction | -4.554871033 | 10.69209565 | -0.426003581 | 0.670109349 |
| Cabazitaxel:Ldose: -1 (uM) Interaction | -4.936191759 | 10.69209565 | -0.461667378 | 0.644324373 |
| Cabozantinib..XL.184.:Ldose: -1 (uM) Interaction | -5.948883033 | 10.69209565 | -0.556381389 | 0.577955742 |
| Capecitabine:Ldose: -1 (uM) Interaction | 1.783057642 | 10.96747366 | 0.162576879 | 0.870853066 |
| Carboplatin:Ldose: -1 (uM) Interaction | 1.62044957 | 10.96747366 | 0.147750487 | 0.882541022 |
| Carfilzomib:Ldose: -1 (uM) Interaction | -51.2748979 | 10.69209565 | -4.79558915 | 1.63E-06 |
| Carmustine:Ldose: -1 (uM) Interaction | 2.28024796 | 10.96747366 | 0.207910047 | 0.835301104 |
| Celecoxib:Ldose: -1 (uM) Interaction | 4.3233086 | 10.96747366 | 0.394193661 | 0.693441835 |
| Chlorambucil:Ldose: -1 (uM) Interaction | -2.614912883 | 10.69209565 | -0.244565048 | 0.806795481 |
| Cisplatin:Ldose: -1 (uM) Interaction | -5.5404824 | 10.69209565 | -0.518184889 | 0.604334419 |
| Cladribine:Ldose: -1 (uM) Interaction | -41.05394993 | 10.69209565 | -3.839654196 | 0.000123548 |
| Clofarabine:Ldose: -1 (uM) Interaction | -67.6245699 | 10.69209565 | -6.32472549 | 2.58E-10 |
| Crizotinib:Ldose: -1 (uM) Interaction | -1.587975419 | 10.69209565 | -0.148518632 | 0.881934826 |
| Cytarabine.HCl...Ara.C:Ldose: -1 (uM) Interaction | -18.33424852 | 10.69209565 | -1.714747896 | 0.086405354 |
| Dacarbazine:Ldose: -1 (uM) Interaction | -0.386442468 | 10.69209565 | -0.036142818 | 0.971168804 |
| Dacomitinib..PF299804.:Ldose: -1 (uM) Interaction | 3.392838536 | 10.69209565 | 0.317322127 | 0.751002161 |
| Dasatinib:Ldose: -1 (uM) Interaction | 3.917243586 | 10.69209565 | 0.366368176 | 0.714093839 |
| Daunorubicin.HCl:Ldose: -1 (uM) Interaction | -33.99526417 | 10.69209565 | -3.179476249 | 0.001477432 |
| Decitabine:Ldose: -1 (uM) Interaction | 2.794306896 | 10.69209565 | 0.261343238 | 0.79383024 |
| Dexrazoxane:Ldose: -1 (uM) Interaction | -3.080904035 | 10.96747366 | -0.280912827 | 0.778779869 |
| Docetaxel:Ldose: -1 (uM) Interaction | -8.233191323 | 10.69209565 | -0.77002597 | 0.441292646 |
| Doxorubicin.HCl:Ldose: -1 (uM) Interaction | -20.73353711 | 10.69209565 | -1.939146243 | 0.052496133 |
| Erlotinib.HCl:Ldose: -1 (uM) Interaction | 4.557660394 | 10.69209565 | 0.426264461 | 0.669919266 |
| Etoposide:Ldose: -1 (uM) Interaction | -8.240194733 | 10.69209565 | -0.770680978 | 0.440904216 |
| Everolimus:Ldose: -1 (uM) Interaction | -1.769943418 | 10.96747366 | -0.161381141 | 0.871794681 |
| Exemestane:Ldose: -1 (uM) Interaction | -12.36053444 | 10.60131818 | -1.165943162 | 0.243649898 |
| Floxuridine:Ldose: -1 (uM) Interaction | -34.0935669 | 10.69209565 | -3.188670212 | 0.001431263 |
| Fludarabine.Phosphate:Ldose: -1 (uM) Interaction | 7.682871739 | 10.69209565 | 0.718556211 | 0.472421932 |
| Fluorouracil...5.FU.:Ldose: -1 (uM) Interaction | -0.642296014 | 10.69209565 | -0.060072042 | 0.952098796 |
| Flutamide..Eulexin.:Ldose: -1 (uM) Interaction | -0.968942666 | 10.69209565 | -0.090622334 | 0.927793499 |
| Foretinib..GSK1363089.:Ldose: -1 (uM) Interaction | -3.283394616 | 10.69209565 | -0.307086162 | 0.758780658 |
| Fulvestrant:Ldose: -1 (uM) Interaction | 5.910819767 | 10.69209565 | 0.552821445 | 0.580391225 |
| Gefitinib:Ldose: -1 (uM) Interaction | -2.343362628 | 10.96747366 | -0.21366476 | 0.830810448 |
| Gemcitabine.HCl:Ldose: -1 (uM) Interaction | 2.660076586 | 10.69209565 | 0.248789075 | 0.803526234 |
| Ibrutinib..PCI.32765.:Ldose: -1 (uM) Interaction | 5.084568173 | 10.60131818 | 0.479616599 | 0.631504745 |
| Imatinib:Ldose: -1 (uM) Interaction | -0.768722257 | 10.96747366 | -0.070091097 | 0.944121782 |
| Imiquimod:Ldose: -1 (uM) Interaction | 24.36391032 | 10.60131818 | 2.29819631 | 0.021559828 |
| INK.128..MLN0128.:Ldose: -1 (uM) Interaction | -34.31695969 | 10.69209565 | -3.209563477 | 0.001331248 |
| Irinotecan.HCl:Ldose: -1 (uM) Interaction | -3.994303349 | 10.69209565 | -0.373575348 | 0.70872383 |
| Ixabepilone:Ldose: -1 (uM) Interaction | -32.05543505 | 10.69209565 | -2.998049783 | 0.002720127 |
| Lapatinib:Ldose: -1 (uM) Interaction | 15.96698261 | 10.60131818 | 1.506131816 | 0.132047505 |
| LDK378:Ldose: -1 (uM) Interaction | -7.438058583 | 10.69209565 | -0.695659563 | 0.486649306 |
| Lenalidomide:Ldose: -1 (uM) Interaction | 7.485053957 | 10.69209565 | 0.700054901 | 0.483900323 |
| Letrozole:Ldose: -1 (uM) Interaction | -7.146803036 | 10.69209565 | -0.668419295 | 0.503872902 |
| Linsitinib:Ldose: -1 (uM) Interaction | -0.027820666 | 10.69209565 | -0.002601984 | 0.997923942 |
| Lomustine..CCNU.:Ldose: -1 (uM) Interaction | 2.610118617 | 10.69209565 | 0.244116654 | 0.807142721 |
| LY2157299:Ldose: -1 (uM) Interaction | 0.571878084 | 10.69209565 | 0.053486061 | 0.957345115 |
| Mechlorethamine.HCl:Ldose: -1 (uM) Interaction | 4.408972432 | 10.69209565 | 0.412358117 | 0.680080921 |
| Megestrol.acetate:Ldose: -1 (uM) Interaction | -4.141303864 | 10.69209565 | -0.38732387 | 0.698520153 |
| MEK.162..ARRY.438162.:Ldose: -1 (uM) Interaction | -37.94373466 | 10.69209565 | -3.548764985 | 0.000387838 |
| Melphalan:Ldose: -1 (uM) Interaction | -3.945509316 | 10.69209565 | -0.369011786 | 0.712122444 |
| Mercaptopurine:Ldose: -1 (uM) Interaction | -4.777868779 | 10.69209565 | -0.446859899 | 0.65498056 |
| Methotrexate:Ldose: -1 (uM) Interaction | -45.000576 | 10.69209565 | -4.208770429 | 2.58E-05 |
| Mitomycin.C:Ldose: -1 (uM) Interaction | -0.666587709 | 10.69209565 | -0.062343972 | 0.950289471 |
| Mitotane..o.p..DDD..Lysodren.:Ldose: -1 (uM) Interaction | -3.568275281 | 10.69209565 | -0.333730206 | 0.738586293 |
| Mitoxantrone:Ldose: -1 (uM) Interaction | -18.92246989 | 10.69209565 | -1.769762496 | 0.076780379 |
| MLN.2480:Ldose: -1 (uM) Interaction | -0.959503094 | 10.69209565 | -0.089739479 | 0.928495049 |
| MLN4924:Ldose: -1 (uM) Interaction | -3.263474799 | 10.69209565 | -0.305223121 | 0.760199074 |
| MLN9708..MLN2238.:Ldose: -1 (uM) Interaction | -5.304145223 | 10.69209565 | -0.496080974 | 0.619842172 |
| Navitoclax..ABT.263..5uM:Ldose: -1 (uM) Interaction | -0.342473273 | 10.69209565 | -0.03203051 | 0.974448007 |
| Nelarabine:Ldose: -1 (uM) Interaction | 5.643252171 | 10.69209565 | 0.527796641 | 0.597645741 |
| OSI.027:Ldose: -1 (uM) Interaction | 10.68243467 | 10.69209565 | 0.999096437 | 0.31775882 |
| Oxaliplatin:Ldose: -1 (uM) Interaction | -10.38227668 | 10.60131818 | -0.979338277 | 0.32742349 |
| Paclitaxel:Ldose: -1 (uM) Interaction | -18.35032716 | 10.69209565 | -1.716251683 | 0.086129878 |
| Palbociclib..PD.0332991..Isethionate:Ldose: -1 (uM) Interaction | -0.581663433 | 10.69209565 | -0.054401256 | 0.956615966 |
| Pazopanib.HCl:Ldose: -1 (uM) Interaction | 2.424775501 | 10.69209565 | 0.226782062 | 0.820595292 |
| PD325901:Ldose: -1 (uM) Interaction | -8.722243711 | 10.69209565 | -0.815765589 | 0.414642932 |
| Pemetrexed:Ldose: -1 (uM) Interaction | -4.671005933 | 10.69209565 | -0.436865334 | 0.662213245 |
| Pipobroman:Ldose: -1 (uM) Interaction | -1.515472738 | 10.96747366 | -0.138178835 | 0.890100329 |
| Plicamycin:Ldose: -1 (uM) Interaction | -52.32281662 | 10.69209565 | -4.893597881 | 9.97E-07 |
| Pralatrexate:Ldose: -1 (uM) Interaction | 2.602034873 | 10.60131818 | 0.245444465 | 0.806114562 |
| Quinacrine.HCl:Ldose: -1 (uM) Interaction | 5.266301221 | 10.69209565 | 0.492541537 | 0.622341416 |
| Quizartinib:Ldose: -1 (uM) Interaction | 1.914321451 | 10.69209565 | 0.179040809 | 0.857907286 |
| Raloxifene:Ldose: -1 (uM) Interaction | -16.18261499 | 10.60131818 | -1.526471965 | 0.126906552 |
| Romidepsin:Ldose: -1 (uM) Interaction | -9.74129692 | 10.69209565 | -0.911074614 | 0.362265911 |
| Sabutoclax..BI.97C1.:Ldose: -1 (uM) Interaction | -3.153865033 | 10.69209565 | -0.294971644 | 0.768018288 |
| Sirolimus..Rapamycin.:Ldose: -1 (uM) Interaction | 1.708503774 | 10.69209565 | 0.159791292 | 0.873046932 |
| Sorafenib:Ldose: -1 (uM) Interaction | 3.357325056 | 10.69209565 | 0.314000657 | 0.753523478 |
| Streptozocin:Ldose: -1 (uM) Interaction | 2.936059934 | 10.69209565 | 0.274600979 | 0.783625358 |
| Sunitinib:Ldose: -1 (uM) Interaction | 8.759294667 | 10.69209565 | 0.819230856 | 0.41266347 |
| Tamoxifen.Citrate:Ldose: -1 (uM) Interaction | 0.471233267 | 10.69209565 | 0.04407305 | 0.964846569 |
| Temozolomide:Ldose: -1 (uM) Interaction | -1.678506297 | 10.96747366 | -0.153044023 | 0.878364943 |
| Temsirolimus..CCI.779..Torisel.:Ldose: -1 (uM) Interaction | -4.531032198 | 10.69209565 | -0.423774005 | 0.671734726 |
| Teniposide:Ldose: -1 (uM) Interaction | -13.50486286 | 10.69209565 | -1.263069776 | 0.206577313 |
| Thioguanine:Ldose: -1 (uM) Interaction | -2.785883923 | 10.69209565 | -0.260555462 | 0.794437746 |
| Thiotepa:Ldose: -1 (uM) Interaction | 6.595836332 | 10.69209565 | 0.616889013 | 0.537314233 |
| Topotecan.HCl:Ldose: -1 (uM) Interaction | -45.38860654 | 10.69209565 | -4.245061775 | 2.19E-05 |
| Trametinib..GSK1120212.:Ldose: -1 (uM) Interaction | 1.318883856 | 10.69209565 | 0.123351296 | 0.901830034 |
| Tretinoin:Ldose: -1 (uM) Interaction | -1.544459368 | 10.96747366 | -0.140821799 | 0.888011994 |
| Triethylenemelamine:Ldose: -1 (uM) Interaction | 1.431228317 | 10.69209565 | 0.13385854 | 0.893515643 |
| Uracil.mustard:Ldose: -1 (uM) Interaction | -3.340281733 | 10.69209565 | -0.312406645 | 0.754734422 |
| Valrubicin:Ldose: -1 (uM) Interaction | -4.846164133 | 10.69209565 | -0.453247361 | 0.650375043 |
| Vandetanib:Ldose: -1 (uM) Interaction | 6.662553923 | 10.60131818 | 0.628464669 | 0.529706022 |
| Vemurafenib:Ldose: -1 (uM) Interaction | -37.74750566 | 10.69209565 | -3.530412268 | 0.000415749 |
| Vinblastine.Sulfate:Ldose: -1 (uM) Interaction | -5.586437866 | 10.69209565 | -0.522482968 | 0.601339294 |
| Vincristine.Sulfate:Ldose: -1 (uM) Interaction | -31.78357185 | 10.69209565 | -2.972623224 | 0.002955821 |
| Vinorelbine.Tartrate:Ldose: -1 (uM) Interaction | -4.242195674 | 10.69209565 | -0.396759982 | 0.691548261 |
| Vismodegib:Ldose: -1 (uM) Interaction | -2.104567483 | 10.69209565 | -0.196833956 | 0.843959272 |
| Vorinostat:Ldose: -1 (uM) Interaction | 1.649198866 | 10.69209565 | 0.154244679 | 0.877418211 |
| Zoledronic.Acid:Ldose: -1 (uM) Interaction | 1.151923275 | 10.96747366 | 0.105030868 | 0.91635226 |
| Abiraterone:Ldose: -0.698970004 (uM) Interaction | -2.070832024 | 10.69209565 | -0.193678778 | 0.846429174 |
| ABT.737:Ldose: -0.698970004 (uM) Interaction | -2.627734242 | 10.69209565 | -0.245764191 | 0.80586704 |
| Actinomycin.D:Ldose: -0.698970004 (uM) Interaction | -19.49814424 | 10.69209565 | -1.823603611 | 0.068225402 |
| Afatinib:Ldose: -0.698970004 (uM) Interaction | -3.576592524 | 10.69209565 | -0.334508093 | 0.737999332 |
| Alisertib..MLN8237.:Ldose: -0.698970004 (uM) Interaction | -17.5848984 | 10.69209565 | -1.644663401 | 0.100053342 |
| Allopurinol:Ldose: -0.698970004 (uM) Interaction | 5.294429933 | 10.69209565 | 0.495172332 | 0.620483359 |
| Amifostine:Ldose: -0.698970004 (uM) Interaction | 5.573673091 | 10.69209565 | 0.521289116 | 0.602170559 |
| Aphrocallistin.analogue:Ldose: -0.698970004 (uM) Interaction | -34.5694749 | 10.69209565 | -3.233180475 | 0.001225982 |
| Arsenic.Trioxide:Ldose: -0.698970004 (uM) Interaction | -1.644419991 | 10.69209565 | -0.153797725 | 0.877770619 |
| Axitinib:Ldose: -0.698970004 (uM) Interaction | 5.392960816 | 10.69209565 | 0.504387633 | 0.613993991 |
| Axitinib.1:Ldose: -0.698970004 (uM) Interaction | 1.818676009 | 10.69209565 | 0.170095374 | 0.864936675 |
| Azacitidine:Ldose: -0.698970004 (uM) Interaction | 17.05040958 | 10.69209565 | 1.594674247 | 0.110799274 |
| Baricitinib..LY3009104..INCB028050.:Ldose: -0.698970004 (uM) Interaction | -10.16356786 | 10.69209565 | -0.950568363 | 0.341833839 |
| Bendamustine.HCl:Ldose: -0.698970004 (uM) Interaction | -0.516206387 | 10.60131818 | -0.04869266 | 0.961164661 |
| BGJ398..NVPBGJ398.:Ldose: -0.698970004 (uM) Interaction | -3.373882257 | 10.69209565 | -0.315549203 | 0.752347653 |
| Bioymifi:Ldose: -0.698970004 (uM) Interaction | 1.274893998 | 10.69209565 | 0.119237055 | 0.905088625 |
| Bleomycin.Sulfate:Ldose: -0.698970004 (uM) Interaction | -32.1664285 | 10.69209565 | -3.008430672 | 0.002628938 |
| Bortezomib:Ldose: -0.698970004 (uM) Interaction | -17.52467563 | 10.69209565 | -1.639030944 | 0.101220924 |
| Bosutinib..SKI.606.:Ldose: -0.698970004 (uM) Interaction | -6.360330041 | 10.69209565 | -0.594862808 | 0.551941217 |
| Busulfan:Ldose: -0.698970004 (uM) Interaction | 1.878640609 | 10.69209565 | 0.175703685 | 0.860528336 |
| Cabazitaxel:Ldose: -0.698970004 (uM) Interaction | -5.963266754 | 10.69209565 | -0.557726656 | 0.577036652 |
| Cabozantinib..XL.184.:Ldose: -0.698970004 (uM) Interaction | -11.1977798 | 10.69209565 | -1.047295139 | 0.29497483 |
| Capecitabine:Ldose: -0.698970004 (uM) Interaction | 0.276613209 | 10.96747366 | 0.025221233 | 0.979878727 |
| Carboplatin:Ldose: -0.698970004 (uM) Interaction | -0.314025721 | 10.96747366 | -0.028632457 | 0.977157982 |
| Carfilzomib:Ldose: -0.698970004 (uM) Interaction | -54.65775705 | 10.69209565 | -5.111977935 | 3.21E-07 |
| Carmustine:Ldose: -0.698970004 (uM) Interaction | -0.097913143 | 10.96747366 | -0.008927593 | 0.992876986 |
| Celecoxib:Ldose: -0.698970004 (uM) Interaction | 0.509428872 | 10.96747366 | 0.046449063 | 0.962952748 |
| Chlorambucil:Ldose: -0.698970004 (uM) Interaction | -3.150138261 | 10.69209565 | -0.29462309 | 0.768284564 |
| Cisplatin:Ldose: -0.698970004 (uM) Interaction | 2.235909476 | 10.69209565 | 0.209117983 | 0.834358047 |
| Cladribine:Ldose: -0.698970004 (uM) Interaction | -63.33466726 | 10.69209565 | -5.923503617 | 3.20E-09 |
| Clofarabine:Ldose: -0.698970004 (uM) Interaction | -77.24067632 | 10.69209565 | -7.224091407 | 5.21E-13 |
| Crizotinib:Ldose: -0.698970004 (uM) Interaction | -3.199042884 | 10.69209565 | -0.299196995 | 0.764792531 |
| Cytarabine.HCl...Ara.C:Ldose: -0.698970004 (uM) Interaction | -45.93028695 | 10.69209565 | -4.295723537 | 1.75E-05 |
| Dacarbazine:Ldose: -0.698970004 (uM) Interaction | 5.921300028 | 10.69209565 | 0.553801633 | 0.579720165 |
| Dacomitinib..PF299804.:Ldose: -0.698970004 (uM) Interaction | -3.116882289 | 10.69209565 | -0.291512758 | 0.7706619 |
| Dasatinib:Ldose: -0.698970004 (uM) Interaction | 4.496978788 | 10.69209565 | 0.420589091 | 0.674059215 |
| Daunorubicin.HCl:Ldose: -0.698970004 (uM) Interaction | -55.39641207 | 10.69209565 | -5.181062149 | 2.23E-07 |
| Decitabine:Ldose: -0.698970004 (uM) Interaction | 8.057884753 | 10.69209565 | 0.753630066 | 0.451079344 |
| Dexrazoxane:Ldose: -0.698970004 (uM) Interaction | 0.082229121 | 10.96747366 | 0.007497544 | 0.994017948 |
| Docetaxel:Ldose: -0.698970004 (uM) Interaction | -9.696295141 | 10.69209565 | -0.906865731 | 0.364487606 |
| Doxorubicin.HCl:Ldose: -0.698970004 (uM) Interaction | -33.62094305 | 10.69209565 | -3.144467104 | 0.00166608 |
| Erlotinib.HCl:Ldose: -0.698970004 (uM) Interaction | 9.261970243 | 10.69209565 | 0.866244611 | 0.386365355 |
| Etoposide:Ldose: -0.698970004 (uM) Interaction | -12.39400092 | 10.69209565 | -1.159174153 | 0.246397619 |
| Everolimus:Ldose: -0.698970004 (uM) Interaction | 0.679727697 | 10.96747366 | 0.061976688 | 0.950581952 |
| Exemestane:Ldose: -0.698970004 (uM) Interaction | 3.990260396 | 10.60131818 | 0.376392853 | 0.70662844 |
| Floxuridine:Ldose: -0.698970004 (uM) Interaction | -37.97456994 | 10.69209565 | -3.551648918 | 0.000383615 |
| Fludarabine.Phosphate:Ldose: -0.698970004 (uM) Interaction | -1.543280386 | 10.69209565 | -0.144338438 | 0.885234531 |
| Fluorouracil...5.FU.:Ldose: -0.698970004 (uM) Interaction | -0.352795747 | 10.69209565 | -0.03299594 | 0.973678121 |
| Flutamide..Eulexin.:Ldose: -0.698970004 (uM) Interaction | -6.419410691 | 10.69209565 | -0.600388446 | 0.548253489 |
| Foretinib..GSK1363089.:Ldose: -0.698970004 (uM) Interaction | -7.554235186 | 10.69209565 | -0.706525216 | 0.47986896 |
| Fulvestrant:Ldose: -0.698970004 (uM) Interaction | 8.327642443 | 10.69209565 | 0.778859703 | 0.43607063 |
| Gefitinib:Ldose: -0.698970004 (uM) Interaction | -3.241766126 | 10.96747366 | -0.295580024 | 0.767553584 |
| Gemcitabine.HCl:Ldose: -0.698970004 (uM) Interaction | -39.45814648 | 10.69209565 | -3.690403432 | 0.000224432 |
| Ibrutinib..PCI.32765.:Ldose: -0.698970004 (uM) Interaction | 6.128003513 | 10.60131818 | 0.578041656 | 0.563241834 |
| Imatinib:Ldose: -0.698970004 (uM) Interaction | 0.074164784 | 10.96747366 | 0.006762249 | 0.994604608 |
| Imiquimod:Ldose: -0.698970004 (uM) Interaction | 2.318243013 | 10.60131818 | 0.218674977 | 0.826905244 |
| INK.128..MLN0128.:Ldose: -0.698970004 (uM) Interaction | -44.23503999 | 10.69209565 | -4.137172116 | 3.53E-05 |
| Irinotecan.HCl:Ldose: -0.698970004 (uM) Interaction | -9.820415107 | 10.69209565 | -0.918474304 | 0.358380541 |
| Ixabepilone:Ldose: -0.698970004 (uM) Interaction | -40.21830849 | 10.69209565 | -3.76149913 | 0.00016933 |
| Lapatinib:Ldose: -0.698970004 (uM) Interaction | 0.523513913 | 10.60131818 | 0.049381964 | 0.960615344 |
| LDK378:Ldose: -0.698970004 (uM) Interaction | -9.464777174 | 10.69209565 | -0.885212542 | 0.376051555 |
| Lenalidomide:Ldose: -0.698970004 (uM) Interaction | 2.160002673 | 10.69209565 | 0.202018645 | 0.839903991 |
| Letrozole:Ldose: -0.698970004 (uM) Interaction | -1.837366541 | 10.69209565 | -0.171843444 | 0.86356217 |
| Linsitinib:Ldose: -0.698970004 (uM) Interaction | -12.37003659 | 10.69209565 | -1.15693284 | 0.247312195 |
| Lomustine..CCNU.:Ldose: -0.698970004 (uM) Interaction | 2.090452976 | 10.69209565 | 0.195513868 | 0.844992464 |
| LY2157299:Ldose: -0.698970004 (uM) Interaction | 4.143837243 | 10.69209565 | 0.387560809 | 0.698344776 |
| Mechlorethamine.HCl:Ldose: -0.698970004 (uM) Interaction | -0.284038759 | 10.69209565 | -0.026565303 | 0.978806686 |
| Megestrol.acetate:Ldose: -0.698970004 (uM) Interaction | -5.558335886 | 10.69209565 | -0.519854673 | 0.603170031 |
| MEK.162..ARRY.438162.:Ldose: -0.698970004 (uM) Interaction | -47.22998023 | 10.69209565 | -4.417279996 | 1.00E-05 |
| Melphalan:Ldose: -0.698970004 (uM) Interaction | 2.679489676 | 10.69209565 | 0.250604724 | 0.802122038 |
| Mercaptopurine:Ldose: -0.698970004 (uM) Interaction | -8.498853646 | 10.69209565 | -0.794872579 | 0.426696087 |
| Methotrexate:Ldose: -0.698970004 (uM) Interaction | 2.496540309 | 10.69209565 | 0.233494012 | 0.815379962 |
| Mitomycin.C:Ldose: -0.698970004 (uM) Interaction | -0.955204742 | 10.69209565 | -0.089337467 | 0.928814521 |
| Mitotane..o.p..DDD..Lysodren.:Ldose: -0.698970004 (uM) Interaction | 3.564282919 | 10.69209565 | 0.333356812 | 0.738868094 |
| Mitoxantrone:Ldose: -0.698970004 (uM) Interaction | -50.77985382 | 10.69209565 | -4.749289146 | 2.05E-06 |
| MLN.2480:Ldose: -0.698970004 (uM) Interaction | -8.906591684 | 10.69209565 | -0.833007109 | 0.404849643 |
| MLN4924:Ldose: -0.698970004 (uM) Interaction | -17.29218392 | 10.69209565 | -1.617286684 | 0.105830559 |
| MLN9708..MLN2238.:Ldose: -0.698970004 (uM) Interaction | -20.46664755 | 10.69209565 | -1.914184854 | 0.055609327 |
| Navitoclax..ABT.263..5uM:Ldose: -0.698970004 (uM) Interaction | -3.016269022 | 10.69209565 | -0.282102697 | 0.777867383 |
| Nelarabine:Ldose: -0.698970004 (uM) Interaction | 4.987390659 | 10.69209565 | 0.466455859 | 0.640893794 |
| OSI.027:Ldose: -0.698970004 (uM) Interaction | 12.25006726 | 10.69209565 | 1.145712465 | 0.251926442 |
| Oxaliplatin:Ldose: -0.698970004 (uM) Interaction | -14.34934777 | 10.60131818 | -1.353543732 | 0.17589571 |
| Paclitaxel:Ldose: -0.698970004 (uM) Interaction | -12.97342384 | 10.69209565 | -1.213365861 | 0.225002827 |
| Palbociclib..PD.0332991..Isethionate:Ldose: -0.698970004 (uM) Interaction | -12.06819605 | 10.69209565 | -1.12870259 | 0.259035461 |
| Pazopanib.HCl:Ldose: -0.698970004 (uM) Interaction | -0.171018541 | 10.69209565 | -0.015994857 | 0.987238638 |
| PD325901:Ldose: -0.698970004 (uM) Interaction | -8.033795354 | 10.69209565 | -0.751377056 | 0.452433697 |
| Pemetrexed:Ldose: -0.698970004 (uM) Interaction | -5.273577741 | 10.69209565 | -0.493222088 | 0.62186053 |
| Pipobroman:Ldose: -0.698970004 (uM) Interaction | -4.665572488 | 10.96747366 | -0.425400838 | 0.670548601 |
| Plicamycin:Ldose: -0.698970004 (uM) Interaction | -101.0141463 | 10.69209565 | -9.447553558 | 3.80E-21 |
| Pralatrexate:Ldose: -0.698970004 (uM) Interaction | -5.55063972 | 10.60131818 | -0.523580146 | 0.600575797 |
| Quinacrine.HCl:Ldose: -0.698970004 (uM) Interaction | 7.409220819 | 10.69209565 | 0.692962452 | 0.488340333 |
| Quizartinib:Ldose: -0.698970004 (uM) Interaction | -2.013828157 | 10.69209565 | -0.188347376 | 0.850606063 |
| Raloxifene:Ldose: -0.698970004 (uM) Interaction | -5.926805787 | 10.60131818 | -0.559063098 | 0.576124273 |
| Romidepsin:Ldose: -0.698970004 (uM) Interaction | 38.01256298 | 10.69209565 | 3.555202293 | 0.00037847 |
| Sabutoclax..BI.97C1.:Ldose: -0.698970004 (uM) Interaction | -7.113290957 | 10.69209565 | -0.66528501 | 0.505875098 |
| Sirolimus..Rapamycin.:Ldose: -0.698970004 (uM) Interaction | 6.517649534 | 10.69209565 | 0.609576434 | 0.542148638 |
| Sorafenib:Ldose: -0.698970004 (uM) Interaction | 7.836454076 | 10.69209565 | 0.732920312 | 0.463614739 |
| Streptozocin:Ldose: -0.698970004 (uM) Interaction | 6.497241754 | 10.69209565 | 0.607667754 | 0.543414043 |
| Sunitinib:Ldose: -0.698970004 (uM) Interaction | 2.270136973 | 10.69209565 | 0.212319179 | 0.831859975 |
| Tamoxifen.Citrate:Ldose: -0.698970004 (uM) Interaction | -7.367443607 | 10.69209565 | -0.689055153 | 0.490795731 |
| Temozolomide:Ldose: -0.698970004 (uM) Interaction | 3.093542807 | 10.96747366 | 0.282065214 | 0.777896124 |
| Temsirolimus..CCI.779..Torisel.:Ldose: -0.698970004 (uM) Interaction | -8.546508414 | 10.69209565 | -0.799329588 | 0.424107842 |
| Teniposide:Ldose: -0.698970004 (uM) Interaction | -19.42743177 | 10.69209565 | -1.816990083 | 0.069232051 |
| Thioguanine:Ldose: -0.698970004 (uM) Interaction | -0.595718697 | 10.69209565 | -0.055715803 | 0.955568709 |
| Thiotepa:Ldose: -0.698970004 (uM) Interaction | 3.134366944 | 10.69209565 | 0.293148046 | 0.769411722 |
| Topotecan.HCl:Ldose: -0.698970004 (uM) Interaction | -68.10714334 | 10.69209565 | -6.369859152 | 1.93E-10 |
| Trametinib..GSK1120212.:Ldose: -0.698970004 (uM) Interaction | 0.344767719 | 10.69209565 | 0.032245102 | 0.974276877 |
| Tretinoin:Ldose: -0.698970004 (uM) Interaction | 1.531746724 | 10.96747366 | 0.139662676 | 0.888927779 |
| Triethylenemelamine:Ldose: -0.698970004 (uM) Interaction | 3.927341976 | 10.69209565 | 0.367312649 | 0.713389306 |
| Uracil.mustard:Ldose: -0.698970004 (uM) Interaction | -6.403100917 | 10.69209565 | -0.598863041 | 0.549270303 |
| Valrubicin:Ldose: -0.698970004 (uM) Interaction | -17.52220318 | 10.69209565 | -1.638799703 | 0.10126909 |
| Vandetanib:Ldose: -0.698970004 (uM) Interaction | 12.34078363 | 10.60131818 | 1.16408011 | 0.244404005 |
| Vemurafenib:Ldose: -0.698970004 (uM) Interaction | -38.52322014 | 10.69209565 | -3.602962544 | 0.000315295 |
| Vinblastine.Sulfate:Ldose: -0.698970004 (uM) Interaction | -10.3354153 | 10.69209565 | -0.966640745 | 0.333734143 |
| Vincristine.Sulfate:Ldose: -0.698970004 (uM) Interaction | -36.38660747 | 10.69209565 | -3.403131497 | 0.000667353 |
| Vinorelbine.Tartrate:Ldose: -0.698970004 (uM) Interaction | -2.294769279 | 10.69209565 | -0.214622966 | 0.83006325 |
| Vismodegib:Ldose: -0.698970004 (uM) Interaction | 6.714817294 | 10.69209565 | 0.62801695 | 0.529999269 |
| Vorinostat:Ldose: -0.698970004 (uM) Interaction | -3.623470651 | 10.69209565 | -0.338892465 | 0.734693932 |
| Zoledronic.Acid:Ldose: -0.698970004 (uM) Interaction | -0.718745373 | 10.96747366 | -0.065534269 | 0.94774921 |
| Abiraterone:Ldose: -0.397940009 (uM) Interaction | -3.704945406 | 10.69209565 | -0.346512557 | 0.728960819 |
| ABT.737:Ldose: -0.397940009 (uM) Interaction | 3.666058711 | 10.69209565 | 0.3428756 | 0.731695281 |
| Actinomycin.D:Ldose: -0.397940009 (uM) Interaction | -34.96616724 | 10.69209565 | -3.270281934 | 0.001076033 |
| Afatinib:Ldose: -0.397940009 (uM) Interaction | -14.64303861 | 10.69209565 | -1.369519979 | 0.170850587 |
| Alisertib..MLN8237.:Ldose: -0.397940009 (uM) Interaction | -24.88384222 | 10.69209565 | -2.327311972 | 0.019957573 |
| Allopurinol:Ldose: -0.397940009 (uM) Interaction | 0.991146894 | 10.69209565 | 0.09269903 | 0.926143501 |
| Amifostine:Ldose: -0.397940009 (uM) Interaction | -2.856668139 | 10.69209565 | -0.2671757 | 0.789336356 |
| Aphrocallistin.analogue:Ldose: -0.397940009 (uM) Interaction | -63.83926615 | 10.69209565 | -5.970697255 | 2.40E-09 |
| Arsenic.Trioxide:Ldose: -0.397940009 (uM) Interaction | -6.557775256 | 10.69209565 | -0.613329273 | 0.539664898 |
| Axitinib:Ldose: -0.397940009 (uM) Interaction | -2.896728899 | 10.69209565 | -0.270922464 | 0.786453175 |
| Axitinib.1:Ldose: -0.397940009 (uM) Interaction | -6.305914722 | 10.69209565 | -0.589773504 | 0.555348485 |
| Azacitidine:Ldose: -0.397940009 (uM) Interaction | 5.762904261 | 10.69209565 | 0.538987346 | 0.589900967 |
| Baricitinib..LY3009104..INCB028050.:Ldose: -0.397940009 (uM) Interaction | -8.592092456 | 10.69209565 | -0.803592928 | 0.421640675 |
| Bendamustine.HCl:Ldose: -0.397940009 (uM) Interaction | -11.052287 | 10.60131818 | -1.042538938 | 0.297173186 |
| BGJ398..NVPBGJ398.:Ldose: -0.397940009 (uM) Interaction | -6.557302211 | 10.69209565 | -0.613285031 | 0.539694145 |
| Bioymifi:Ldose: -0.397940009 (uM) Interaction | 2.015679771 | 10.69209565 | 0.188520552 | 0.850470322 |
| Bleomycin.Sulfate:Ldose: -0.397940009 (uM) Interaction | -44.53475333 | 10.69209565 | -4.165203416 | 3.12E-05 |
| Bortezomib:Ldose: -0.397940009 (uM) Interaction | -21.1440111 | 10.69209565 | -1.977536658 | 0.047993296 |
| Bosutinib..SKI.606.:Ldose: -0.397940009 (uM) Interaction | -20.7717772 | 10.69209565 | -1.942722725 | 0.052062241 |
| Busulfan:Ldose: -0.397940009 (uM) Interaction | -4.081960939 | 10.69209565 | -0.381773702 | 0.702632865 |
| Cabazitaxel:Ldose: -0.397940009 (uM) Interaction | -7.123450722 | 10.69209565 | -0.666235222 | 0.505267656 |
| Cabozantinib..XL.184.:Ldose: -0.397940009 (uM) Interaction | -10.08916384 | 10.69209565 | -0.943609576 | 0.345379422 |
| Capecitabine:Ldose: -0.397940009 (uM) Interaction | 0.189691511 | 10.96747366 | 0.017295826 | 0.986200771 |
| Carboplatin:Ldose: -0.397940009 (uM) Interaction | -2.041870523 | 10.96747366 | -0.186175101 | 0.852309142 |
| Carfilzomib:Ldose: -0.397940009 (uM) Interaction | -58.68554333 | 10.69209565 | -5.488684842 | 4.09E-08 |
| Carmustine:Ldose: -0.397940009 (uM) Interaction | 2.812015131 | 10.96747366 | 0.256395886 | 0.79764753 |
| Celecoxib:Ldose: -0.397940009 (uM) Interaction | 1.553036951 | 10.96747366 | 0.141603891 | 0.887394172 |
| Chlorambucil:Ldose: -0.397940009 (uM) Interaction | 3.151366394 | 10.69209565 | 0.294737954 | 0.768196812 |
| Cisplatin:Ldose: -0.397940009 (uM) Interaction | -3.761581989 | 10.69209565 | -0.351809609 | 0.724984377 |
| Cladribine:Ldose: -0.397940009 (uM) Interaction | -81.04069212 | 10.69209565 | -7.579495617 | 3.60E-14 |
| Clofarabine:Ldose: -0.397940009 (uM) Interaction | -81.21869818 | 10.69209565 | -7.596143996 | 3.17E-14 |
| Crizotinib:Ldose: -0.397940009 (uM) Interaction | -0.433685209 | 10.69209565 | -0.040561292 | 0.967646007 |
| Cytarabine.HCl...Ara.C:Ldose: -0.397940009 (uM) Interaction | -61.03329532 | 10.69209565 | -5.708263124 | 1.16E-08 |
| Dacarbazine:Ldose: -0.397940009 (uM) Interaction | 4.829276299 | 10.69209565 | 0.451667892 | 0.651512646 |
| Dacomitinib..PF299804.:Ldose: -0.397940009 (uM) Interaction | 0.199126346 | 10.69209565 | 0.018623697 | 0.985141466 |
| Dasatinib:Ldose: -0.397940009 (uM) Interaction | 3.103958099 | 10.69209565 | 0.290303997 | 0.771586381 |
| Daunorubicin.HCl:Ldose: -0.397940009 (uM) Interaction | -74.56799126 | 10.69209565 | -6.974123099 | 3.16E-12 |
| Decitabine:Ldose: -0.397940009 (uM) Interaction | -3.836943492 | 10.69209565 | -0.358857947 | 0.719704759 |
| Dexrazoxane:Ldose: -0.397940009 (uM) Interaction | -1.893590906 | 10.96747366 | -0.172655159 | 0.86292406 |
| Docetaxel:Ldose: -0.397940009 (uM) Interaction | -13.77684355 | 10.69209565 | -1.288507323 | 0.197582791 |
| Doxorubicin.HCl:Ldose: -0.397940009 (uM) Interaction | -53.84550322 | 10.69209565 | -5.036010243 | 4.79E-07 |
| Erlotinib.HCl:Ldose: -0.397940009 (uM) Interaction | 6.644286088 | 10.69209565 | 0.621420375 | 0.534329423 |
| Etoposide:Ldose: -0.397940009 (uM) Interaction | -27.51172264 | 10.69209565 | -2.573089835 | 0.010085908 |
| Everolimus:Ldose: -0.397940009 (uM) Interaction | 0.368222498 | 10.96747366 | 0.033574049 | 0.973217117 |
| Exemestane:Ldose: -0.397940009 (uM) Interaction | -2.818092468 | 10.60131818 | -0.265824723 | 0.79037666 |
| Floxuridine:Ldose: -0.397940009 (uM) Interaction | -48.65225078 | 10.69209565 | -4.550300743 | 5.39E-06 |
| Fludarabine.Phosphate:Ldose: -0.397940009 (uM) Interaction | 9.512319466 | 10.69209565 | 0.889659032 | 0.373658597 |
| Fluorouracil...5.FU.:Ldose: -0.397940009 (uM) Interaction | 5.323887013 | 10.69209565 | 0.497927365 | 0.61854015 |
| Flutamide..Eulexin.:Ldose: -0.397940009 (uM) Interaction | -5.273467306 | 10.69209565 | -0.493211759 | 0.621867827 |
| Foretinib..GSK1363089.:Ldose: -0.397940009 (uM) Interaction | -15.13281797 | 10.69209565 | -1.41532759 | 0.156986436 |
| Fulvestrant:Ldose: -0.397940009 (uM) Interaction | 4.515695561 | 10.69209565 | 0.422339615 | 0.672781219 |
| Gefitinib:Ldose: -0.397940009 (uM) Interaction | -3.728711944 | 10.96747366 | -0.339979111 | 0.733875462 |
| Gemcitabine.HCl:Ldose: -0.397940009 (uM) Interaction | -42.35993143 | 10.69209565 | -3.961798773 | 7.46E-05 |
| Ibrutinib..PCI.32765.:Ldose: -0.397940009 (uM) Interaction | -5.578996604 | 10.60131818 | -0.526254991 | 0.598716285 |
| Imatinib:Ldose: -0.397940009 (uM) Interaction | -1.377902361 | 10.96747366 | -0.125635347 | 0.900021714 |
| Imiquimod:Ldose: -0.397940009 (uM) Interaction | 10.61002028 | 10.60131818 | 1.000820851 | 0.316924289 |
| INK.128..MLN0128.:Ldose: -0.397940009 (uM) Interaction | -54.98492397 | 10.69209565 | -5.142576887 | 2.73E-07 |
| Irinotecan.HCl:Ldose: -0.397940009 (uM) Interaction | -10.8727837 | 10.69209565 | -1.016899217 | 0.30921241 |
| Ixabepilone:Ldose: -0.397940009 (uM) Interaction | -67.44797989 | 10.69209565 | -6.30820955 | 2.88E-10 |
| Lapatinib:Ldose: -0.397940009 (uM) Interaction | 9.647947099 | 10.60131818 | 0.910070515 | 0.362795161 |
| LDK378:Ldose: -0.397940009 (uM) Interaction | -13.72188074 | 10.69209565 | -1.283366815 | 0.199376933 |
| Lenalidomide:Ldose: -0.397940009 (uM) Interaction | 8.549886334 | 10.69209565 | 0.799645515 | 0.423924728 |
| Letrozole:Ldose: -0.397940009 (uM) Interaction | -3.578117992 | 10.69209565 | -0.334650765 | 0.737891693 |
| Linsitinib:Ldose: -0.397940009 (uM) Interaction | -6.854971601 | 10.69209565 | -0.641125166 | 0.521447955 |
| Lomustine..CCNU.:Ldose: -0.397940009 (uM) Interaction | -0.264880872 | 10.69209565 | -0.024773523 | 0.980235832 |
| LY2157299:Ldose: -0.397940009 (uM) Interaction | -4.040620539 | 10.69209565 | -0.377907257 | 0.705503089 |
| Mechlorethamine.HCl:Ldose: -0.397940009 (uM) Interaction | 7.064578143 | 10.69209565 | 0.660729044 | 0.50879292 |
| Megestrol.acetate:Ldose: -0.397940009 (uM) Interaction | -0.607287871 | 10.69209565 | -0.056797834 | 0.954706749 |
| MEK.162..ARRY.438162.:Ldose: -0.397940009 (uM) Interaction | -55.52424568 | 10.69209565 | -5.193018048 | 2.09E-07 |
| Melphalan:Ldose: -0.397940009 (uM) Interaction | -4.273354406 | 10.69209565 | -0.399674165 | 0.689400351 |
| Mercaptopurine:Ldose: -0.397940009 (uM) Interaction | -4.958031652 | 10.69209565 | -0.463709998 | 0.64286006 |
| Methotrexate:Ldose: -0.397940009 (uM) Interaction | -5.124198756 | 10.69209565 | -0.479251114 | 0.631764695 |
| Mitomycin.C:Ldose: -0.397940009 (uM) Interaction | -0.590793862 | 10.69209565 | -0.055255198 | 0.95593565 |
| Mitotane..o.p..DDD..Lysodren.:Ldose: -0.397940009 (uM) Interaction | -0.480194749 | 10.69209565 | -0.044911191 | 0.964178499 |
| Mitoxantrone:Ldose: -0.397940009 (uM) Interaction | -65.99312488 | 10.69209565 | -6.172141275 | 6.85E-10 |
| MLN.2480:Ldose: -0.397940009 (uM) Interaction | -9.560606554 | 10.69209565 | -0.89417518 | 0.371237822 |
| MLN4924:Ldose: -0.397940009 (uM) Interaction | -55.42678559 | 10.69209565 | -5.183902894 | 2.19E-07 |
| MLN9708..MLN2238.:Ldose: -0.397940009 (uM) Interaction | -42.13406761 | 10.69209565 | -3.9406744 | 8.15E-05 |
| Navitoclax..ABT.263..5uM:Ldose: -0.397940009 (uM) Interaction | -4.212457831 | 10.69209565 | -0.393978689 | 0.693600541 |
| Nelarabine:Ldose: -0.397940009 (uM) Interaction | 6.998546944 | 10.69209565 | 0.654553342 | 0.512762124 |
| OSI.027:Ldose: -0.397940009 (uM) Interaction | 7.640899294 | 10.69209565 | 0.714630653 | 0.474844781 |
| Oxaliplatin:Ldose: -0.397940009 (uM) Interaction | -4.157160001 | 10.60131818 | -0.392136141 | 0.694961373 |
| Paclitaxel:Ldose: -0.397940009 (uM) Interaction | -27.92712822 | 10.69209565 | -2.611941487 | 0.009009 |
| Palbociclib..PD.0332991..Isethionate:Ldose: -0.397940009 (uM) Interaction | -14.17026552 | 10.69209565 | -1.325302914 | 0.185084283 |
| Pazopanib.HCl:Ldose: -0.397940009 (uM) Interaction | -4.095827139 | 10.69209565 | -0.383070567 | 0.701671095 |
| PD325901:Ldose: -0.397940009 (uM) Interaction | -12.56888114 | 10.69209565 | -1.175530181 | 0.239795222 |
| Pemetrexed:Ldose: -0.397940009 (uM) Interaction | -4.395442489 | 10.69209565 | -0.411092702 | 0.681008512 |
| Pipobroman:Ldose: -0.397940009 (uM) Interaction | -3.041068199 | 10.96747366 | -0.277280647 | 0.781567189 |
| Plicamycin:Ldose: -0.397940009 (uM) Interaction | -106.3562211 | 10.69209565 | -9.947181975 | 2.90E-23 |
| Pralatrexate:Ldose: -0.397940009 (uM) Interaction | -5.880621396 | 10.60131818 | -0.554706622 | 0.57910091 |
| Quinacrine.HCl:Ldose: -0.397940009 (uM) Interaction | 7.330598764 | 10.69209565 | 0.685609164 | 0.492966729 |
| Quizartinib:Ldose: -0.397940009 (uM) Interaction | -2.727769422 | 10.69209565 | -0.255120185 | 0.798632628 |
| Raloxifene:Ldose: -0.397940009 (uM) Interaction | -14.89658775 | 10.60131818 | -1.405163726 | 0.159986531 |
| Romidepsin:Ldose: -0.397940009 (uM) Interaction | 35.29511356 | 10.69209565 | 3.301047308 | 0.000964759 |
| Sabutoclax..BI.97C1.:Ldose: -0.397940009 (uM) Interaction | -33.91443512 | 10.69209565 | -3.171916547 | 0.001516417 |
| Sirolimus..Rapamycin.:Ldose: -0.397940009 (uM) Interaction | 3.338455543 | 10.69209565 | 0.312235847 | 0.754864211 |
| Sorafenib:Ldose: -0.397940009 (uM) Interaction | 1.993467413 | 10.69209565 | 0.186443096 | 0.852098996 |
| Streptozocin:Ldose: -0.397940009 (uM) Interaction | 3.989616594 | 10.69209565 | 0.373137009 | 0.709050022 |
| Sunitinib:Ldose: -0.397940009 (uM) Interaction | 12.89909246 | 10.69209565 | 1.206413867 | 0.227670752 |
| Tamoxifen.Citrate:Ldose: -0.397940009 (uM) Interaction | -4.245142772 | 10.69209565 | -0.397035615 | 0.691344998 |
| Temozolomide:Ldose: -0.397940009 (uM) Interaction | 0.330203201 | 10.96747366 | 0.030107499 | 0.97598159 |
| Temsirolimus..CCI.779..Torisel.:Ldose: -0.397940009 (uM) Interaction | -7.945629482 | 10.69209565 | -0.743131164 | 0.457410104 |
| Teniposide:Ldose: -0.397940009 (uM) Interaction | -45.39728721 | 10.69209565 | -4.245873652 | 2.19E-05 |
| Thioguanine:Ldose: -0.397940009 (uM) Interaction | -11.7515528 | 10.69209565 | -1.099087886 | 0.271741601 |
| Thiotepa:Ldose: -0.397940009 (uM) Interaction | 8.200975043 | 10.69209565 | 0.767012877 | 0.443081981 |
| Topotecan.HCl:Ldose: -0.397940009 (uM) Interaction | -82.72626175 | 10.69209565 | -7.737141946 | 1.06E-14 |
| Trametinib..GSK1120212.:Ldose: -0.397940009 (uM) Interaction | -3.623044384 | 10.69209565 | -0.338852597 | 0.734723967 |
| Tretinoin:Ldose: -0.397940009 (uM) Interaction | 1.544648657 | 10.96747366 | 0.140839058 | 0.887998359 |
| Triethylenemelamine:Ldose: -0.397940009 (uM) Interaction | 2.014205828 | 10.69209565 | 0.188382698 | 0.850578376 |
| Uracil.mustard:Ldose: -0.397940009 (uM) Interaction | -3.588577956 | 10.69209565 | -0.335629055 | 0.737153769 |
| Valrubicin:Ldose: -0.397940009 (uM) Interaction | -28.12211363 | 10.69209565 | -2.630177895 | 0.008539883 |
| Vandetanib:Ldose: -0.397940009 (uM) Interaction | 2.509089716 | 10.60131818 | 0.236677145 | 0.812909442 |
| Vemurafenib:Ldose: -0.397940009 (uM) Interaction | -48.81644557 | 10.69209565 | -4.565657395 | 5.01E-06 |
| Vinblastine.Sulfate:Ldose: -0.397940009 (uM) Interaction | -14.62198687 | 10.69209565 | -1.367551072 | 0.171466425 |
| Vincristine.Sulfate:Ldose: -0.397940009 (uM) Interaction | -41.998434 | 10.69209565 | -3.92798899 | 8.59E-05 |
| Vinorelbine.Tartrate:Ldose: -0.397940009 (uM) Interaction | -27.54128578 | 10.69209565 | -2.575854788 | 0.010005641 |
| Vismodegib:Ldose: -0.397940009 (uM) Interaction | 1.642959278 | 10.69209565 | 0.153661109 | 0.877878342 |
| Vorinostat:Ldose: -0.397940009 (uM) Interaction | 0.023603906 | 10.69209565 | 0.002207603 | 0.998238609 |
| Zoledronic.Acid:Ldose: -0.397940009 (uM) Interaction | -3.784456579 | 10.96747366 | -0.345061834 | 0.73005114 |
| Abiraterone:Ldose: 0 (uM) Interaction | -0.177040659 | 10.69209565 | -0.016558088 | 0.986789309 |
| ABT.737:Ldose: 0 (uM) Interaction | 5.446665108 | 10.69209565 | 0.509410436 | 0.610469604 |
| Actinomycin.D:Ldose: 0 (uM) Interaction | -38.53135731 | 10.69209565 | -3.603723589 | 0.000314374 |
| Afatinib:Ldose: 0 (uM) Interaction | -16.15392883 | 10.69209565 | -1.510829061 | 0.13084618 |
| Alisertib..MLN8237.:Ldose: 0 (uM) Interaction | -28.25667505 | 10.69209565 | -2.642763026 | 0.008229003 |
| Allopurinol:Ldose: 0 (uM) Interaction | 6.749461074 | 10.69209565 | 0.63125708 | 0.527878917 |
| Amifostine:Ldose: 0 (uM) Interaction | -0.132112409 | 10.69209565 | -0.012356082 | 0.990141634 |
| Aphrocallistin.analogue:Ldose: 0 (uM) Interaction | -78.07340437 | 10.69209565 | -7.301973993 | 2.93E-13 |
| Arsenic.Trioxide:Ldose: 0 (uM) Interaction | -8.274645209 | 10.69209565 | -0.773903029 | 0.438996344 |
| Axitinib:Ldose: 0 (uM) Interaction | -2.719966852 | 10.69209565 | -0.254390434 | 0.799196287 |
| Axitinib.1:Ldose: 0 (uM) Interaction | -3.916147976 | 10.69209565 | -0.366265707 | 0.714170291 |
| Azacitidine:Ldose: 0 (uM) Interaction | 6.542615124 | 10.69209565 | 0.611911391 | 0.540602624 |
| Baricitinib..LY3009104..INCB028050.:Ldose: 0 (uM) Interaction | -7.257057709 | 10.69209565 | -0.678731088 | 0.49731531 |
| Bendamustine.HCl:Ldose: 0 (uM) Interaction | -15.17580877 | 10.60131818 | -1.431502056 | 0.152300395 |
| BGJ398..NVPBGJ398.:Ldose: 0 (uM) Interaction | -3.855010389 | 10.69209565 | -0.36054769 | 0.718441015 |
| Bioymifi:Ldose: 0 (uM) Interaction | 3.312049318 | 10.69209565 | 0.309766151 | 0.756741689 |
| Bleomycin.Sulfate:Ldose: 0 (uM) Interaction | -52.30251416 | 10.69209565 | -4.891699052 | 1.01E-06 |
| Bortezomib:Ldose: 0 (uM) Interaction | -17.63659259 | 10.69209565 | -1.649498206 | 0.099059698 |
| Bosutinib..SKI.606.:Ldose: 0 (uM) Interaction | -22.61086118 | 10.69209565 | -2.114726797 | 0.034464305 |
| Busulfan:Ldose: 0 (uM) Interaction | -3.453018442 | 10.69209565 | -0.322950575 | 0.746735702 |
| Cabazitaxel:Ldose: 0 (uM) Interaction | -0.618273012 | 10.69209565 | -0.057825241 | 0.95388835 |
| Cabozantinib..XL.184.:Ldose: 0 (uM) Interaction | -8.227485032 | 10.69209565 | -0.769492277 | 0.441609279 |
| Capecitabine:Ldose: 0 (uM) Interaction | 0.212481123 | 10.96747366 | 0.019373753 | 0.984543122 |
| Carboplatin:Ldose: 0 (uM) Interaction | -0.741780992 | 10.96747366 | -0.067634627 | 0.946077097 |
| Carfilzomib:Ldose: 0 (uM) Interaction | -54.94656536 | 10.69209565 | -5.13898932 | 2.79E-07 |
| Carmustine:Ldose: 0 (uM) Interaction | 0.33374407 | 10.96747366 | 0.030430351 | 0.975724112 |
| Celecoxib:Ldose: 0 (uM) Interaction | -0.806875158 | 10.96747366 | -0.073569829 | 0.941353339 |
| Chlorambucil:Ldose: 0 (uM) Interaction | 0.689649008 | 10.69209565 | 0.064500827 | 0.948572027 |
| Cisplatin:Ldose: 0 (uM) Interaction | -6.071642193 | 10.69209565 | -0.56786269 | 0.570133926 |
| Cladribine:Ldose: 0 (uM) Interaction | -80.88008276 | 10.69209565 | -7.5644743 | 4.04E-14 |
| Clofarabine:Ldose: 0 (uM) Interaction | -78.68567289 | 10.69209565 | -7.359237652 | 1.91E-13 |
| Crizotinib:Ldose: 0 (uM) Interaction | -2.382525262 | 10.69209565 | -0.222830523 | 0.823669453 |
| Cytarabine.HCl...Ara.C:Ldose: 0 (uM) Interaction | -77.01212397 | 10.69209565 | -7.202715584 | 6.09E-13 |
| Dacarbazine:Ldose: 0 (uM) Interaction | 2.345913589 | 10.69209565 | 0.219406341 | 0.826335539 |
| Dacomitinib..PF299804.:Ldose: 0 (uM) Interaction | -1.129607674 | 10.69209565 | -0.105648856 | 0.915861911 |
| Dasatinib:Ldose: 0 (uM) Interaction | 1.105609584 | 10.69209565 | 0.103404386 | 0.917642961 |
| Daunorubicin.HCl:Ldose: 0 (uM) Interaction | -89.8512055 | 10.69209565 | -8.403516807 | 4.59E-17 |
| Decitabine:Ldose: 0 (uM) Interaction | -5.989211561 | 10.69209565 | -0.560153197 | 0.575380576 |
| Dexrazoxane:Ldose: 0 (uM) Interaction | -4.518157883 | 10.96747366 | -0.411959766 | 0.680372874 |
| Docetaxel:Ldose: 0 (uM) Interaction | -15.27211784 | 10.69209565 | -1.428355894 | 0.153203455 |
| Doxorubicin.HCl:Ldose: 0 (uM) Interaction | -73.89593107 | 10.69209565 | -6.911267303 | 4.93E-12 |
| Erlotinib.HCl:Ldose: 0 (uM) Interaction | 5.650806034 | 10.69209565 | 0.528503132 | 0.597155435 |
| Etoposide:Ldose: 0 (uM) Interaction | -39.74889534 | 10.69209565 | -3.71759631 | 0.000201619 |
| Everolimus:Ldose: 0 (uM) Interaction | -1.367079192 | 10.96747366 | -0.124648505 | 0.90080295 |
| Exemestane:Ldose: 0 (uM) Interaction | -13.34273125 | 10.60131818 | -1.258591717 | 0.208191019 |
| Floxuridine:Ldose: 0 (uM) Interaction | -50.38162405 | 10.69209565 | -4.712043896 | 2.47E-06 |
| Fludarabine.Phosphate:Ldose: 0 (uM) Interaction | 6.513019329 | 10.69209565 | 0.609143384 | 0.54243561 |
| Fluorouracil...5.FU.:Ldose: 0 (uM) Interaction | 4.515508826 | 10.69209565 | 0.42232215 | 0.672793965 |
| Flutamide..Eulexin.:Ldose: 0 (uM) Interaction | -3.358367026 | 10.69209565 | -0.314098109 | 0.753449464 |
| Foretinib..GSK1363089.:Ldose: 0 (uM) Interaction | -30.04459302 | 10.69209565 | -2.809981691 | 0.004958747 |
| Fulvestrant:Ldose: 0 (uM) Interaction | 2.943127358 | 10.69209565 | 0.275261974 | 0.783117527 |
| Gefitinib:Ldose: 0 (uM) Interaction | -8.057330545 | 10.96747366 | -0.734656931 | 0.462556183 |
| Gemcitabine.HCl:Ldose: 0 (uM) Interaction | -38.86582563 | 10.69209565 | -3.635005419 | 0.0002786 |
| Ibrutinib..PCI.32765.:Ldose: 0 (uM) Interaction | 1.330094116 | 10.60131818 | 0.125464975 | 0.900156583 |
| Imatinib:Ldose: 0 (uM) Interaction | -2.563484825 | 10.96747366 | -0.233735216 | 0.815192692 |
| Imiquimod:Ldose: 0 (uM) Interaction | 20.23221273 | 10.60131818 | 1.908461984 | 0.05634436 |
| INK.128..MLN0128.:Ldose: 0 (uM) Interaction | -53.98421402 | 10.69209565 | -5.048983453 | 4.48E-07 |
| Irinotecan.HCl:Ldose: 0 (uM) Interaction | -18.19571135 | 10.69209565 | -1.701790925 | 0.08880851 |
| Ixabepilone:Ldose: 0 (uM) Interaction | -77.04472233 | 10.69209565 | -7.205764412 | 5.96E-13 |
| Lapatinib:Ldose: 0 (uM) Interaction | 9.473398083 | 10.60131818 | 0.893605674 | 0.371542554 |
| LDK378:Ldose: 0 (uM) Interaction | -15.84790158 | 10.69209565 | -1.48220724 | 0.138299294 |
| Lenalidomide:Ldose: 0 (uM) Interaction | 6.189804331 | 10.69209565 | 0.578914044 | 0.562653022 |
| Letrozole:Ldose: 0 (uM) Interaction | -10.442397 | 10.69209565 | -0.976646425 | 0.328754823 |
| Linsitinib:Ldose: 0 (uM) Interaction | -14.52933538 | 10.69209565 | -1.358885653 | 0.174196572 |
| Lomustine..CCNU.:Ldose: 0 (uM) Interaction | 2.177678591 | 10.69209565 | 0.203671821 | 0.838611823 |
| LY2157299:Ldose: 0 (uM) Interaction | -1.303075259 | 10.69209565 | -0.121872765 | 0.903000884 |
| Mechlorethamine.HCl:Ldose: 0 (uM) Interaction | 4.917700439 | 10.69209565 | 0.459937939 | 0.645565252 |
| Megestrol.acetate:Ldose: 0 (uM) Interaction | -2.870535807 | 10.69209565 | -0.268472702 | 0.788337968 |
| MEK.162..ARRY.438162.:Ldose: 0 (uM) Interaction | -56.05580507 | 10.69209565 | -5.242733221 | 1.60E-07 |
| Melphalan:Ldose: 0 (uM) Interaction | -1.806113709 | 10.69209565 | -0.168920459 | 0.865860739 |
| Mercaptopurine:Ldose: 0 (uM) Interaction | -13.99840055 | 10.69209565 | -1.309228893 | 0.19047032 |
| Methotrexate:Ldose: 0 (uM) Interaction | -2.472349842 | 10.69209565 | -0.231231549 | 0.817137043 |
| Mitomycin.C:Ldose: 0 (uM) Interaction | -29.07165883 | 10.69209565 | -2.718986042 | 0.006553304 |
| Mitotane..o.p..DDD..Lysodren.:Ldose: 0 (uM) Interaction | -0.107697969 | 10.69209565 | -0.010072672 | 0.991963397 |
| Mitoxantrone:Ldose: 0 (uM) Interaction | -68.87140206 | 10.69209565 | -6.441338004 | 1.21E-10 |
| MLN.2480:Ldose: 0 (uM) Interaction | -29.26716425 | 10.69209565 | -2.737271084 | 0.006200032 |
| MLN4924:Ldose: 0 (uM) Interaction | -72.94399299 | 10.69209565 | -6.822235358 | 9.19E-12 |
| MLN9708..MLN2238.:Ldose: 0 (uM) Interaction | -39.36422733 | 10.69209565 | -3.681619452 | 0.000232304 |
| Navitoclax..ABT.263..5uM:Ldose: 0 (uM) Interaction | -0.333671097 | 10.69209565 | -0.031207268 | 0.975104523 |
| Nelarabine:Ldose: 0 (uM) Interaction | 8.322247408 | 10.69209565 | 0.778355121 | 0.436367948 |
| OSI.027:Ldose: 0 (uM) Interaction | 7.455380041 | 10.69209565 | 0.697279587 | 0.485635111 |
| Oxaliplatin:Ldose: 0 (uM) Interaction | -6.032326567 | 10.60131818 | -0.569016651 | 0.569350572 |
| Paclitaxel:Ldose: 0 (uM) Interaction | -29.23733386 | 10.69209565 | -2.734481136 | 0.006252801 |
| Palbociclib..PD.0332991..Isethionate:Ldose: 0 (uM) Interaction | -15.45698534 | 10.69209565 | -1.445646003 | 0.148290572 |
| Pazopanib.HCl:Ldose: 0 (uM) Interaction | -4.221431417 | 10.69209565 | -0.394817962 | 0.692981014 |
| PD325901:Ldose: 0 (uM) Interaction | -9.378600137 | 10.69209565 | -0.87715266 | 0.380413176 |
| Pemetrexed:Ldose: 0 (uM) Interaction | -1.967804309 | 10.69209565 | -0.184042902 | 0.853981473 |
| Pipobroman:Ldose: 0 (uM) Interaction | -2.950241552 | 10.96747366 | -0.268999192 | 0.787932794 |
| Plicamycin:Ldose: 0 (uM) Interaction | -103.1545143 | 10.69209565 | -9.647735827 | 5.55E-22 |
| Pralatrexate:Ldose: 0 (uM) Interaction | 0.585957763 | 10.60131818 | 0.055272161 | 0.955922136 |
| Quinacrine.HCl:Ldose: 0 (uM) Interaction | 2.673622144 | 10.69209565 | 0.250055951 | 0.802546384 |
| Quizartinib:Ldose: 0 (uM) Interaction | -3.250067259 | 10.69209565 | -0.303969153 | 0.761154229 |
| Raloxifene:Ldose: 0 (uM) Interaction | -18.02762267 | 10.60131818 | -1.70050765 | 0.089049424 |
| Romidepsin:Ldose: 0 (uM) Interaction | 39.30989109 | 10.69209565 | 3.676537545 | 0.000236976 |
| Sabutoclax..BI.97C1.:Ldose: 0 (uM) Interaction | -47.01634956 | 10.69209565 | -4.397299753 | 1.10E-05 |
| Sirolimus..Rapamycin.:Ldose: 0 (uM) Interaction | 3.485225428 | 10.69209565 | 0.325962799 | 0.74445556 |
| Sorafenib:Ldose: 0 (uM) Interaction | -1.431666649 | 10.69209565 | -0.133899536 | 0.893483226 |
| Streptozocin:Ldose: 0 (uM) Interaction | 0.078963574 | 10.69209565 | 0.007385229 | 0.994107559 |
| Sunitinib:Ldose: 0 (uM) Interaction | 5.703461754 | 10.69209565 | 0.533427865 | 0.59374275 |
| Tamoxifen.Citrate:Ldose: 0 (uM) Interaction | 1.812972308 | 10.69209565 | 0.169561924 | 0.865356208 |
| Temozolomide:Ldose: 0 (uM) Interaction | -3.554476317 | 10.96747366 | -0.324092533 | 0.74587102 |
| Temsirolimus..CCI.779..Torisel.:Ldose: 0 (uM) Interaction | -5.486319564 | 10.69209565 | -0.5131192 | 0.607873026 |
| Teniposide:Ldose: 0 (uM) Interaction | -48.49919595 | 10.69209565 | -4.535985978 | 5.76E-06 |
| Thioguanine:Ldose: 0 (uM) Interaction | -33.07681336 | 10.69209565 | -3.093576268 | 0.001980042 |
| Thiotepa:Ldose: 0 (uM) Interaction | 4.465949339 | 10.69209565 | 0.417686998 | 0.676180002 |
| Topotecan.HCl:Ldose: 0 (uM) Interaction | -87.99271831 | 10.69209565 | -8.229698011 | 1.98E-16 |
| Trametinib..GSK1120212.:Ldose: 0 (uM) Interaction | -0.211169241 | 10.69209565 | -0.019750033 | 0.984242955 |
| Tretinoin:Ldose: 0 (uM) Interaction | -3.617628137 | 10.96747366 | -0.329850634 | 0.741515925 |
| Triethylenemelamine:Ldose: 0 (uM) Interaction | 2.560862441 | 10.69209565 | 0.23950987 | 0.810712446 |
| Uracil.mustard:Ldose: 0 (uM) Interaction | -2.688882176 | 10.69209565 | -0.251483176 | 0.801442885 |
| Valrubicin:Ldose: 0 (uM) Interaction | -37.13176055 | 10.69209565 | -3.472823454 | 0.00051599 |
| Vandetanib:Ldose: 0 (uM) Interaction | 2.624704349 | 10.60131818 | 0.247582829 | 0.804459477 |
| Vemurafenib:Ldose: 0 (uM) Interaction | -55.22520088 | 10.69209565 | -5.165049274 | 2.42E-07 |
| Vinblastine.Sulfate:Ldose: 0 (uM) Interaction | -10.78104423 | 10.69209565 | -1.008319096 | 0.313312241 |
| Vincristine.Sulfate:Ldose: 0 (uM) Interaction | -41.86581616 | 10.69209565 | -3.915585637 | 9.05E-05 |
| Vinorelbine.Tartrate:Ldose: 0 (uM) Interaction | -57.34456553 | 10.69209565 | -5.363267166 | 8.25E-08 |
| Vismodegib:Ldose: 0 (uM) Interaction | 3.134050908 | 10.69209565 | 0.293118488 | 0.769434314 |
| Vorinostat:Ldose: 0 (uM) Interaction | -9.165882841 | 10.69209565 | -0.857257842 | 0.391311616 |
| Zoledronic.Acid:Ldose: 0 (uM) Interaction | 3.260650612 | 10.96747366 | 0.297301887 | 0.766238811 |
| Abiraterone:Ldose: 0.301029996 (uM) Interaction | 0.523947282 | 10.69209565 | 0.049003236 | 0.960917156 |
| ABT.737:Ldose: 0.301029996 (uM) Interaction | -9.327106788 | 10.69209565 | -0.872336639 | 0.383034149 |
| Actinomycin.D:Ldose: 0.301029996 (uM) Interaction | -35.54005085 | 10.69209565 | -3.323955566 | 0.000888933 |
| Afatinib:Ldose: 0.301029996 (uM) Interaction | -29.94866351 | 10.69209565 | -2.801009689 | 0.005098686 |
| Alisertib..MLN8237.:Ldose: 0.301029996 (uM) Interaction | -24.48204383 | 10.69209565 | -2.28973296 | 0.022046099 |
| Allopurinol:Ldose: 0.301029996 (uM) Interaction | 4.390310755 | 10.69209565 | 0.410612746 | 0.681360462 |
| Amifostine:Ldose: 0.301029996 (uM) Interaction | 1.992772548 | 10.69209565 | 0.186378107 | 0.852149955 |
| Aphrocallistin.analogue:Ldose: 0.301029996 (uM) Interaction | -94.78639888 | 10.69209565 | -8.865090809 | 8.21E-19 |
| Arsenic.Trioxide:Ldose: 0.301029996 (uM) Interaction | -5.437726232 | 10.69209565 | -0.508574409 | 0.611055602 |
| Axitinib:Ldose: 0.301029996 (uM) Interaction | -0.558086228 | 10.69209565 | -0.05219615 | 0.958372868 |
| Axitinib.1:Ldose: 0.301029996 (uM) Interaction | -1.085509468 | 10.69209565 | -0.101524482 | 0.919135037 |
| Azacitidine:Ldose: 0.301029996 (uM) Interaction | 1.335336105 | 10.69209565 | 0.124890026 | 0.90061174 |
| Baricitinib..LY3009104..INCB028050.:Ldose: 0.301029996 (uM) Interaction | -16.2677456 | 10.69209565 | -1.521474006 | 0.128155111 |
| Bendamustine.HCl:Ldose: 0.301029996 (uM) Interaction | -18.31364209 | 10.60131818 | -1.72748726 | 0.08409404 |
| BGJ398..NVPBGJ398.:Ldose: 0.301029996 (uM) Interaction | -15.0294023 | 10.69209565 | -1.40565543 | 0.159840402 |
| Bioymifi:Ldose: 0.301029996 (uM) Interaction | -7.82724692 | 10.69209565 | -0.732059194 | 0.464140134 |
| Bleomycin.Sulfate:Ldose: 0.301029996 (uM) Interaction | -63.29294878 | 10.69209565 | -5.919601812 | 3.27E-09 |
| Bortezomib:Ldose: 0.301029996 (uM) Interaction | -19.45619288 | 10.69209565 | -1.819680025 | 0.068821151 |
| Bosutinib..SKI.606.:Ldose: 0.301029996 (uM) Interaction | -37.26056805 | 10.69209565 | -3.484870438 | 0.000493321 |
| Busulfan:Ldose: 0.301029996 (uM) Interaction | -2.868893677 | 10.69209565 | -0.268319118 | 0.788456174 |
| Cabazitaxel:Ldose: 0.301029996 (uM) Interaction | 2.812947168 | 10.69209565 | 0.263086607 | 0.792486257 |
| Cabozantinib..XL.184.:Ldose: 0.301029996 (uM) Interaction | -16.59537063 | 10.69209565 | -1.552115803 | 0.120648711 |
| Capecitabine:Ldose: 0.301029996 (uM) Interaction | -7.358253654 | 10.96747366 | -0.670916008 | 0.502280988 |
| Carboplatin:Ldose: 0.301029996 (uM) Interaction | -6.11939378 | 10.96747366 | -0.557958375 | 0.576878411 |
| Carfilzomib:Ldose: 0.301029996 (uM) Interaction | -57.25896987 | 10.69209565 | -5.355261657 | 8.63E-08 |
| Carmustine:Ldose: 0.301029996 (uM) Interaction | -7.781546869 | 10.96747366 | -0.709511334 | 0.47801464 |
| Celecoxib:Ldose: 0.301029996 (uM) Interaction | -9.406084187 | 10.96747366 | -0.857634536 | 0.391103517 |
| Chlorambucil:Ldose: 0.301029996 (uM) Interaction | -5.089324818 | 10.69209565 | -0.475989458 | 0.634086552 |
| Cisplatin:Ldose: 0.301029996 (uM) Interaction | -12.54358638 | 10.69209565 | -1.173164438 | 0.240742403 |
| Cladribine:Ldose: 0.301029996 (uM) Interaction | -82.58895331 | 10.69209565 | -7.724299895 | 1.17E-14 |
| Clofarabine:Ldose: 0.301029996 (uM) Interaction | -80.38746726 | 10.69209565 | -7.518401433 | 5.75E-14 |
| Crizotinib:Ldose: 0.301029996 (uM) Interaction | -7.860446087 | 10.69209565 | -0.735164213 | 0.462247224 |
| Cytarabine.HCl...Ara.C:Ldose: 0.301029996 (uM) Interaction | -87.9035206 | 10.69209565 | -8.221355614 | 2.12E-16 |
| Dacarbazine:Ldose: 0.301029996 (uM) Interaction | -0.35803019 | 10.69209565 | -0.033485502 | 0.973287727 |
| Dacomitinib..PF299804.:Ldose: 0.301029996 (uM) Interaction | -6.425361637 | 10.69209565 | -0.60094502 | 0.547882715 |
| Dasatinib:Ldose: 0.301029996 (uM) Interaction | 2.354851492 | 10.69209565 | 0.220242277 | 0.825684489 |
| Daunorubicin.HCl:Ldose: 0.301029996 (uM) Interaction | -93.28130748 | 10.69209565 | -8.724324074 | 2.86E-18 |
| Decitabine:Ldose: 0.301029996 (uM) Interaction | -7.755572175 | 10.69209565 | -0.725355667 | 0.468241476 |
| Dexrazoxane:Ldose: 0.301029996 (uM) Interaction | -5.431884779 | 10.96747366 | -0.495272197 | 0.620412875 |
| Docetaxel:Ldose: 0.301029996 (uM) Interaction | -10.56248802 | 10.69209565 | -0.987878183 | 0.323223032 |
| Doxorubicin.HCl:Ldose: 0.301029996 (uM) Interaction | -77.53893533 | 10.69209565 | -7.251986689 | 4.24E-13 |
| Erlotinib.HCl:Ldose: 0.301029996 (uM) Interaction | -3.181389135 | 10.69209565 | -0.297545892 | 0.766052549 |
| Etoposide:Ldose: 0.301029996 (uM) Interaction | -57.48745699 | 10.69209565 | -5.376631379 | 7.67E-08 |
| Everolimus:Ldose: 0.301029996 (uM) Interaction | 2.474472031 | 10.96747366 | 0.225619145 | 0.821499717 |
| Exemestane:Ldose: 0.301029996 (uM) Interaction | -10.48044371 | 10.60131818 | -0.988598166 | 0.322870512 |
| Floxuridine:Ldose: 0.301029996 (uM) Interaction | -55.38474004 | 10.69209565 | -5.179970499 | 2.24E-07 |
| Fludarabine.Phosphate:Ldose: 0.301029996 (uM) Interaction | -5.400066815 | 10.69209565 | -0.505052236 | 0.61352714 |
| Fluorouracil...5.FU.:Ldose: 0.301029996 (uM) Interaction | 7.376561072 | 10.69209565 | 0.689907883 | 0.490259299 |
| Flutamide..Eulexin.:Ldose: 0.301029996 (uM) Interaction | -8.868056268 | 10.69209565 | -0.829403006 | 0.406885281 |
| Foretinib..GSK1363089.:Ldose: 0.301029996 (uM) Interaction | -52.65476675 | 10.69209565 | -4.924644193 | 8.51E-07 |
| Fulvestrant:Ldose: 0.301029996 (uM) Interaction | -4.869031835 | 10.69209565 | -0.455386109 | 0.648835922 |
| Gefitinib:Ldose: 0.301029996 (uM) Interaction | -9.66964459 | 10.96747366 | -0.881665631 | 0.377967156 |
| Gemcitabine.HCl:Ldose: 0.301029996 (uM) Interaction | -40.8336304 | 10.69209565 | -3.819048364 | 0.00013433 |
| Ibrutinib..PCI.32765.:Ldose: 0.301029996 (uM) Interaction | 10.60420674 | 10.60131818 | 1.000272472 | 0.317189521 |
| Imatinib:Ldose: 0.301029996 (uM) Interaction | -9.937495114 | 10.96747366 | -0.906087894 | 0.364899124 |
| Imiquimod:Ldose: 0.301029996 (uM) Interaction | 21.80724451 | 10.60131818 | 2.057031412 | 0.039694811 |
| INK.128..MLN0128.:Ldose: 0.301029996 (uM) Interaction | -57.89280746 | 10.69209565 | -5.414542608 | 6.21E-08 |
| Irinotecan.HCl:Ldose: 0.301029996 (uM) Interaction | -47.05996976 | 10.69209565 | -4.401379421 | 1.08E-05 |
| Ixabepilone:Ldose: 0.301029996 (uM) Interaction | -81.82411578 | 10.69209565 | -7.652766908 | 2.05E-14 |
| Lapatinib:Ldose: 0.301029996 (uM) Interaction | -3.144473541 | 10.60131818 | -0.296611562 | 0.766765846 |
| LDK378:Ldose: 0.301029996 (uM) Interaction | -18.25291998 | 10.69209565 | -1.707141479 | 0.087809682 |
| Lenalidomide:Ldose: 0.301029996 (uM) Interaction | -4.314159205 | 10.69209565 | -0.403490517 | 0.686591279 |
| Letrozole:Ldose: 0.301029996 (uM) Interaction | -11.57201183 | 10.69209565 | -1.082295951 | 0.279132749 |
| Linsitinib:Ldose: 0.301029996 (uM) Interaction | -11.55215268 | 10.69209565 | -1.080438583 | 0.279958609 |
| Lomustine..CCNU.:Ldose: 0.301029996 (uM) Interaction | 4.223941265 | 10.69209565 | 0.395052701 | 0.692807774 |
| LY2157299:Ldose: 0.301029996 (uM) Interaction | -3.317901352 | 10.69209565 | -0.310313475 | 0.756325486 |
| Mechlorethamine.HCl:Ldose: 0.301029996 (uM) Interaction | -2.855375303 | 10.69209565 | -0.267054785 | 0.78942945 |
| Megestrol.acetate:Ldose: 0.301029996 (uM) Interaction | -18.36504981 | 10.69209565 | -1.717628649 | 0.085878257 |
| MEK.162..ARRY.438162.:Ldose: 0.301029996 (uM) Interaction | -56.86271584 | 10.69209565 | -5.318201192 | 1.06E-07 |
| Melphalan:Ldose: 0.301029996 (uM) Interaction | -2.642898268 | 10.69209565 | -0.247182438 | 0.804769312 |
| Mercaptopurine:Ldose: 0.301029996 (uM) Interaction | -35.12444229 | 10.69209565 | -3.28508493 | 0.001021089 |
| Methotrexate:Ldose: 0.301029996 (uM) Interaction | 1.575686882 | 10.69209565 | 0.147369322 | 0.882841851 |
| Mitomycin.C:Ldose: 0.301029996 (uM) Interaction | -38.12407362 | 10.69209565 | -3.565631553 | 0.00036374 |
| Mitotane..o.p..DDD..Lysodren.:Ldose: 0.301029996 (uM) Interaction | -6.241755383 | 10.69209565 | -0.583772872 | 0.559379038 |
| Mitoxantrone:Ldose: 0.301029996 (uM) Interaction | -76.26081896 | 10.69209565 | -7.132448255 | 1.02E-12 |
| MLN.2480:Ldose: 0.301029996 (uM) Interaction | -36.03381548 | 10.69209565 | -3.370135908 | 0.000752585 |
| MLN4924:Ldose: 0.301029996 (uM) Interaction | -88.19208704 | 10.69209565 | -8.248344377 | 1.69E-16 |
| MLN9708..MLN2238.:Ldose: 0.301029996 (uM) Interaction | -92.95287735 | 10.69209565 | -8.693606978 | 3.75E-18 |
| Navitoclax..ABT.263..5uM:Ldose: 0.301029996 (uM) Interaction | -4.041770483 | 10.69209565 | -0.378014808 | 0.705423192 |
| Nelarabine:Ldose: 0.301029996 (uM) Interaction | 7.91302073 | 10.69209565 | 0.740081364 | 0.459258419 |
| OSI.027:Ldose: 0.301029996 (uM) Interaction | -15.47248068 | 10.69209565 | -1.447095236 | 0.147884311 |
| Oxaliplatin:Ldose: 0.301029996 (uM) Interaction | -11.84276372 | 10.60131818 | -1.117102942 | 0.263962351 |
| Paclitaxel:Ldose: 0.301029996 (uM) Interaction | -24.37753265 | 10.69209565 | -2.27995834 | 0.022619559 |
| Palbociclib..PD.0332991..Isethionate:Ldose: 0.301029996 (uM) Interaction | -23.27880019 | 10.69209565 | -2.177197151 | 0.029476312 |
| Pazopanib.HCl:Ldose: 0.301029996 (uM) Interaction | -9.355340387 | 10.69209565 | -0.874977244 | 0.381595713 |
| PD325901:Ldose: 0.301029996 (uM) Interaction | -12.29635942 | 10.69209565 | -1.150042034 | 0.250138894 |
| Pemetrexed:Ldose: 0.301029996 (uM) Interaction | -10.14541172 | 10.69209565 | -0.948870273 | 0.34269688 |
| Pipobroman:Ldose: 0.301029996 (uM) Interaction | -9.722358022 | 10.96747366 | -0.886471973 | 0.375372811 |
| Plicamycin:Ldose: 0.301029996 (uM) Interaction | -53.88868406 | 10.69209565 | -5.040048819 | 4.69E-07 |
| Pralatrexate:Ldose: 0.301029996 (uM) Interaction | -4.346560332 | 10.60131818 | -0.410001875 | 0.681808511 |
| Quinacrine.HCl:Ldose: 0.301029996 (uM) Interaction | 9.600978137 | 10.69209565 | 0.897951015 | 0.369221362 |
| Quizartinib:Ldose: 0.301029996 (uM) Interaction | -3.161859968 | 10.69209565 | -0.295719387 | 0.767447144 |
| Raloxifene:Ldose: 0.301029996 (uM) Interaction | -13.00697212 | 10.60131818 | -1.226920266 | 0.219865516 |
| Romidepsin:Ldose: 0.301029996 (uM) Interaction | 34.50748465 | 10.69209565 | 3.227382711 | 0.001251087 |
| Sabutoclax..BI.97C1.:Ldose: 0.301029996 (uM) Interaction | -18.7706324 | 10.69209565 | -1.755561586 | 0.079177027 |
| Sirolimus..Rapamycin.:Ldose: 0.301029996 (uM) Interaction | 3.261211905 | 10.69209565 | 0.305011479 | 0.760360257 |
| Sorafenib:Ldose: 0.301029996 (uM) Interaction | 1.914774382 | 10.69209565 | 0.17908317 | 0.857874024 |
| Streptozocin:Ldose: 0.301029996 (uM) Interaction | 5.26458284 | 10.69209565 | 0.492380822 | 0.622455003 |
| Sunitinib:Ldose: 0.301029996 (uM) Interaction | 4.51297507 | 10.69209565 | 0.422085176 | 0.672966918 |
| Tamoxifen.Citrate:Ldose: 0.301029996 (uM) Interaction | -3.53120338 | 10.69209565 | -0.330262981 | 0.741204365 |
| Temozolomide:Ldose: 0.301029996 (uM) Interaction | -6.362943154 | 10.96747366 | -0.58016489 | 0.561809291 |
| Temsirolimus..CCI.779..Torisel.:Ldose: 0.301029996 (uM) Interaction | -0.300423943 | 10.69209565 | -0.028097761 | 0.977584432 |
| Teniposide:Ldose: 0.301029996 (uM) Interaction | -45.10284582 | 10.69209565 | -4.218335423 | 2.47E-05 |
| Thioguanine:Ldose: 0.301029996 (uM) Interaction | -84.97898078 | 10.69209565 | -7.947832077 | 1.99E-15 |
| Thiotepa:Ldose: 0.301029996 (uM) Interaction | -1.633306512 | 10.69209565 | -0.152758315 | 0.878590254 |
| Topotecan.HCl:Ldose: 0.301029996 (uM) Interaction | -102.6703879 | 10.69209565 | -9.602456922 | 8.60E-22 |
| Trametinib..GSK1120212.:Ldose: 0.301029996 (uM) Interaction | -2.069697645 | 10.69209565 | -0.193572683 | 0.846512253 |
| Tretinoin:Ldose: 0.301029996 (uM) Interaction | -9.27686001 | 10.96747366 | -0.845852043 | 0.397644366 |
| Triethylenemelamine:Ldose: 0.301029996 (uM) Interaction | 2.870715757 | 10.69209565 | 0.268489532 | 0.788325015 |
| Uracil.mustard:Ldose: 0.301029996 (uM) Interaction | 0.344280815 | 10.69209565 | 0.032199564 | 0.974313192 |
| Valrubicin:Ldose: 0.301029996 (uM) Interaction | -57.70566948 | 10.69209565 | -5.397040147 | 6.84E-08 |
| Vandetanib:Ldose: 0.301029996 (uM) Interaction | -2.308818024 | 10.60131818 | -0.217785938 | 0.827597894 |
| Vemurafenib:Ldose: 0.301029996 (uM) Interaction | -70.62902496 | 10.69209565 | -6.605723262 | 4.04E-11 |
| Vinblastine.Sulfate:Ldose: 0.301029996 (uM) Interaction | -13.56207165 | 10.69209565 | -1.268420345 | 0.204661123 |
| Vincristine.Sulfate:Ldose: 0.301029996 (uM) Interaction | -43.18401265 | 10.69209565 | -4.038872646 | 5.39E-05 |
| Vinorelbine.Tartrate:Ldose: 0.301029996 (uM) Interaction | -63.25613912 | 10.69209565 | -5.916159113 | 3.34E-09 |
| Vismodegib:Ldose: 0.301029996 (uM) Interaction | -5.589067885 | 10.69209565 | -0.522728946 | 0.601168086 |
| Vorinostat:Ldose: 0.301029996 (uM) Interaction | -32.15749964 | 10.69209565 | -3.007595582 | 0.002636169 |
| Zoledronic.Acid:Ldose: 0.301029996 (uM) Interaction | -9.68318769 | 10.96747366 | -0.882900473 | 0.377299567 |
| Abiraterone:Ldose: 0.602059991 (uM) Interaction | -7.12359946 | 10.69209565 | -0.666249133 | 0.505258766 |
| ABT.737:Ldose: 0.602059991 (uM) Interaction | -3.997570523 | 10.69209565 | -0.373880917 | 0.70849647 |
| Actinomycin.D:Ldose: 0.602059991 (uM) Interaction | -42.38570207 | 10.69209565 | -3.964209025 | 7.39E-05 |
| Afatinib:Ldose: 0.602059991 (uM) Interaction | -38.18847557 | 10.69209565 | -3.571654876 | 0.00035548 |
| Alisertib..MLN8237.:Ldose: 0.602059991 (uM) Interaction | -26.57959366 | 10.69209565 | -2.485910576 | 0.012929311 |
| Allopurinol:Ldose: 0.602059991 (uM) Interaction | 4.118301957 | 10.69209565 | 0.38517257 | 0.700113239 |
| Amifostine:Ldose: 0.602059991 (uM) Interaction | -2.808075877 | 10.69209565 | -0.26263101 | 0.792837423 |
| Aphrocallistin.analogue:Ldose: 0.602059991 (uM) Interaction | -86.9026738 | 10.69209565 | -8.127749381 | 4.60E-16 |
| Arsenic.Trioxide:Ldose: 0.602059991 (uM) Interaction | -6.534829743 | 10.69209565 | -0.611183248 | 0.541084503 |
| Axitinib:Ldose: 0.602059991 (uM) Interaction | -5.309581487 | 10.69209565 | -0.496589412 | 0.619483517 |
| Axitinib.1:Ldose: 0.602059991 (uM) Interaction | -22.64679883 | 10.69209565 | -2.118087938 | 0.034178654 |
| Azacitidine:Ldose: 0.602059991 (uM) Interaction | -13.2933979 | 10.69209565 | -1.243292086 | 0.213773293 |
| Baricitinib..LY3009104..INCB028050.:Ldose: 0.602059991 (uM) Interaction | -21.24380168 | 10.69209565 | -1.986869775 | 0.046949048 |
| Bendamustine.HCl:Ldose: 0.602059991 (uM) Interaction | -12.84582579 | 10.60131818 | -1.211719672 | 0.225632548 |
| BGJ398..NVPBGJ398.:Ldose: 0.602059991 (uM) Interaction | -6.896451218 | 10.69209565 | -0.645004632 | 0.518930821 |
| Bioymifi:Ldose: 0.602059991 (uM) Interaction | -1.350125573 | 10.69209565 | -0.126273241 | 0.899516776 |
| Bleomycin.Sulfate:Ldose: 0.602059991 (uM) Interaction | -74.41698634 | 10.69209565 | -6.960000057 | 3.50E-12 |
| Bortezomib:Ldose: 0.602059991 (uM) Interaction | -21.12626531 | 10.69209565 | -1.975876947 | 0.048181025 |
| Bosutinib..SKI.606.:Ldose: 0.602059991 (uM) Interaction | -40.19709877 | 10.69209565 | -3.759515448 | 0.000170677 |
| Busulfan:Ldose: 0.602059991 (uM) Interaction | -8.026082577 | 10.69209565 | -0.750655703 | 0.45286781 |
| Cabazitaxel:Ldose: 0.602059991 (uM) Interaction | 1.394952008 | 10.69209565 | 0.130465725 | 0.896199147 |
| Cabozantinib..XL.184.:Ldose: 0.602059991 (uM) Interaction | -20.36084852 | 10.69209565 | -1.904289784 | 0.056885312 |
| Capecitabine:Ldose: 0.602059991 (uM) Interaction | -0.723528022 | 10.96747366 | -0.065970345 | 0.947402027 |
| Carboplatin:Ldose: 0.602059991 (uM) Interaction | -4.110324665 | 10.96747366 | -0.374774063 | 0.70783207 |
| Carfilzomib:Ldose: 0.602059991 (uM) Interaction | -58.81719052 | 10.69209565 | -5.500997414 | 3.82E-08 |
| Carmustine:Ldose: 0.602059991 (uM) Interaction | -0.214897869 | 10.96747366 | -0.019594108 | 0.984367339 |
| Celecoxib:Ldose: 0.602059991 (uM) Interaction | -1.56002394 | 10.96747366 | -0.142240956 | 0.886890968 |
| Chlorambucil:Ldose: 0.602059991 (uM) Interaction | -6.042677777 | 10.69209565 | -0.565153734 | 0.571974889 |
| Cisplatin:Ldose: 0.602059991 (uM) Interaction | -6.231492627 | 10.69209565 | -0.582813027 | 0.560025069 |
| Cladribine:Ldose: 0.602059991 (uM) Interaction | -84.87392102 | 10.69209565 | -7.938006149 | 2.15E-15 |
| Clofarabine:Ldose: 0.602059991 (uM) Interaction | -82.53496946 | 10.69209565 | -7.719250946 | 1.22E-14 |
| Crizotinib:Ldose: 0.602059991 (uM) Interaction | -20.31898415 | 10.69209565 | -1.900374333 | 0.057396899 |
| Cytarabine.HCl...Ara.C:Ldose: 0.602059991 (uM) Interaction | -92.67071805 | 10.69209565 | -8.667217454 | 4.72E-18 |
| Dacarbazine:Ldose: 0.602059991 (uM) Interaction | -8.1647594 | 10.69209565 | -0.763625735 | 0.445098389 |
| Dacomitinib..PF299804.:Ldose: 0.602059991 (uM) Interaction | -1.871832157 | 10.69209565 | -0.175066911 | 0.861028648 |
| Dasatinib:Ldose: 0.602059991 (uM) Interaction | 1.062265678 | 10.69209565 | 0.099350559 | 0.92086083 |
| Daunorubicin.HCl:Ldose: 0.602059991 (uM) Interaction | -94.82357367 | 10.69209565 | -8.868567656 | 7.95E-19 |
| Decitabine:Ldose: 0.602059991 (uM) Interaction | -13.28111018 | 10.69209565 | -1.242142852 | 0.214196926 |
| Dexrazoxane:Ldose: 0.602059991 (uM) Interaction | -4.493014309 | 10.96747366 | -0.409667207 | 0.682054024 |
| Docetaxel:Ldose: 0.602059991 (uM) Interaction | -8.703501249 | 10.69209565 | -0.814012663 | 0.41564639 |
| Doxorubicin.HCl:Ldose: 0.602059991 (uM) Interaction | -44.31274381 | 10.69209565 | -4.144439524 | 3.42E-05 |
| Erlotinib.HCl:Ldose: 0.602059991 (uM) Interaction | -10.63183707 | 10.69209565 | -0.994364193 | 0.320056385 |
| Etoposide:Ldose: 0.602059991 (uM) Interaction | -74.2635437 | 10.69209565 | -6.945649022 | 3.87E-12 |
| Everolimus:Ldose: 0.602059991 (uM) Interaction | 0.311949773 | 10.96747366 | 0.028443175 | 0.977308944 |
| Exemestane:Ldose: 0.602059991 (uM) Interaction | -11.82227387 | 10.60131818 | -1.115170177 | 0.264789523 |
| Floxuridine:Ldose: 0.602059991 (uM) Interaction | -61.20470651 | 10.69209565 | -5.724294704 | 1.05E-08 |
| Fludarabine.Phosphate:Ldose: 0.602059991 (uM) Interaction | -10.38046893 | 10.69209565 | -0.970854477 | 0.331631282 |
| Fluorouracil...5.FU.:Ldose: 0.602059991 (uM) Interaction | 2.533201292 | 10.69209565 | 0.236922805 | 0.812718855 |
| Flutamide..Eulexin.:Ldose: 0.602059991 (uM) Interaction | -11.09424658 | 10.69209565 | -1.037611984 | 0.299461989 |
| Foretinib..GSK1363089.:Ldose: 0.602059991 (uM) Interaction | -71.09559255 | 10.69209565 | -6.649359945 | 3.01E-11 |
| Fulvestrant:Ldose: 0.602059991 (uM) Interaction | -0.016835193 | 10.69209565 | -0.001574546 | 0.998743709 |
| Gefitinib:Ldose: 0.602059991 (uM) Interaction | -8.382734974 | 10.96747366 | -0.764326884 | 0.444680557 |
| Gemcitabine.HCl:Ldose: 0.602059991 (uM) Interaction | -43.84131187 | 10.69209565 | -4.100347893 | 4.14E-05 |
| Ibrutinib..PCI.32765.:Ldose: 0.602059991 (uM) Interaction | -6.228927766 | 10.60131818 | -0.587561628 | 0.556832524 |
| Imatinib:Ldose: 0.602059991 (uM) Interaction | -5.426587649 | 10.96747366 | -0.494789212 | 0.620753796 |
| Imiquimod:Ldose: 0.602059991 (uM) Interaction | 7.359848447 | 10.60131818 | 0.694238992 | 0.487539577 |
| INK.128..MLN0128.:Ldose: 0.602059991 (uM) Interaction | -64.13329247 | 10.69209565 | -5.998196665 | 2.03E-09 |
| Irinotecan.HCl:Ldose: 0.602059991 (uM) Interaction | -61.18728708 | 10.69209565 | -5.722665517 | 1.06E-08 |
| Ixabepilone:Ldose: 0.602059991 (uM) Interaction | -85.93644035 | 10.69209565 | -8.037380431 | 9.63E-16 |
| Lapatinib:Ldose: 0.602059991 (uM) Interaction | 8.040882814 | 10.60131818 | 0.758479528 | 0.448171981 |
| LDK378:Ldose: 0.602059991 (uM) Interaction | -52.45446144 | 10.69209565 | -4.905910231 | 9.37E-07 |
| Lenalidomide:Ldose: 0.602059991 (uM) Interaction | 2.42305893 | 10.69209565 | 0.226621516 | 0.820720137 |
| Letrozole:Ldose: 0.602059991 (uM) Interaction | -6.397213847 | 10.69209565 | -0.598312441 | 0.549637555 |
| Linsitinib:Ldose: 0.602059991 (uM) Interaction | -13.62132017 | 10.69209565 | -1.273961683 | 0.202690275 |
| Lomustine..CCNU.:Ldose: 0.602059991 (uM) Interaction | 1.775276957 | 10.69209565 | 0.166036389 | 0.868129819 |
| LY2157299:Ldose: 0.602059991 (uM) Interaction | -10.81634685 | 10.69209565 | -1.011620846 | 0.311730353 |
| Mechlorethamine.HCl:Ldose: 0.602059991 (uM) Interaction | -0.619393262 | 10.69209565 | -0.057930015 | 0.953804894 |
| Megestrol.acetate:Ldose: 0.602059991 (uM) Interaction | -3.283120125 | 10.69209565 | -0.30706049 | 0.758800198 |
| MEK.162..ARRY.438162.:Ldose: 0.602059991 (uM) Interaction | -58.7426772 | 10.69209565 | -5.494028403 | 3.97E-08 |
| Melphalan:Ldose: 0.602059991 (uM) Interaction | -12.22552057 | 10.69209565 | -1.143416686 | 0.252877905 |
| Mercaptopurine:Ldose: 0.602059991 (uM) Interaction | -87.59885103 | 10.69209565 | -8.192860772 | 2.69E-16 |
| Methotrexate:Ldose: 0.602059991 (uM) Interaction | -4.473199693 | 10.69209565 | -0.418365103 | 0.675684227 |
| Mitomycin.C:Ldose: 0.602059991 (uM) Interaction | -67.10074376 | 10.69209565 | -6.275733584 | 3.54E-10 |
| Mitotane..o.p..DDD..Lysodren.:Ldose: 0.602059991 (uM) Interaction | -0.462118453 | 10.69209565 | -0.043220569 | 0.965526096 |
| Mitoxantrone:Ldose: 0.602059991 (uM) Interaction | -87.98482821 | 10.69209565 | -8.228960074 | 1.99E-16 |
| MLN.2480:Ldose: 0.602059991 (uM) Interaction | -35.06655866 | 10.69209565 | -3.279671245 | 0.001040874 |
| MLN4924:Ldose: 0.602059991 (uM) Interaction | -93.45302585 | 10.69209565 | -8.740384384 | 2.48E-18 |
| MLN9708..MLN2238.:Ldose: 0.602059991 (uM) Interaction | -94.93185509 | 10.69209565 | -8.878694897 | 7.26E-19 |
| Navitoclax..ABT.263..5uM:Ldose: 0.602059991 (uM) Interaction | -9.345537195 | 10.69209565 | -0.874060381 | 0.382094786 |
| Nelarabine:Ldose: 0.602059991 (uM) Interaction | 8.667570623 | 10.69209565 | 0.810652178 | 0.417574096 |
| OSI.027:Ldose: 0.602059991 (uM) Interaction | -35.53058109 | 10.69209565 | -3.323069888 | 0.000891759 |
| Oxaliplatin:Ldose: 0.602059991 (uM) Interaction | -4.947656436 | 10.60131818 | -0.466702004 | 0.640717656 |
| Paclitaxel:Ldose: 0.602059991 (uM) Interaction | -26.55115977 | 10.69209565 | -2.483251239 | 0.013026217 |
| Palbociclib..PD.0332991..Isethionate:Ldose: 0.602059991 (uM) Interaction | -28.427231 | 10.69209565 | -2.658714618 | 0.007849538 |
| Pazopanib.HCl:Ldose: 0.602059991 (uM) Interaction | -19.27067147 | 10.69209565 | -1.802328758 | 0.071507197 |
| PD325901:Ldose: 0.602059991 (uM) Interaction | -14.81704695 | 10.69209565 | -1.385794463 | 0.165823534 |
| Pemetrexed:Ldose: 0.602059991 (uM) Interaction | -4.317115577 | 10.69209565 | -0.403767018 | 0.686387924 |
| Pipobroman:Ldose: 0.602059991 (uM) Interaction | -7.933490505 | 10.96747366 | -0.723365357 | 0.469463037 |
| Plicamycin:Ldose: 0.602059991 (uM) Interaction | -54.17795312 | 10.69209565 | -5.067103297 | 4.07E-07 |
| Pralatrexate:Ldose: 0.602059991 (uM) Interaction | -5.372004135 | 10.60131818 | -0.506729828 | 0.61234941 |
| Quinacrine.HCl:Ldose: 0.602059991 (uM) Interaction | 7.459718527 | 10.69209565 | 0.697685353 | 0.485381266 |
| Quizartinib:Ldose: 0.602059991 (uM) Interaction | -7.217494027 | 10.69209565 | -0.675030814 | 0.499663188 |
| Raloxifene:Ldose: 0.602059991 (uM) Interaction | -12.3700456 | 10.60131818 | -1.166840329 | 0.243287337 |
| Romidepsin:Ldose: 0.602059991 (uM) Interaction | 34.68581331 | 10.69209565 | 3.244061262 | 0.001180119 |
| Sabutoclax..BI.97C1.:Ldose: 0.602059991 (uM) Interaction | -85.14018407 | 10.69209565 | -7.962908942 | 1.76E-15 |
| Sirolimus..Rapamycin.:Ldose: 0.602059991 (uM) Interaction | 4.313760372 | 10.69209565 | 0.403453216 | 0.686618714 |
| Sorafenib:Ldose: 0.602059991 (uM) Interaction | -7.913001942 | 10.69209565 | -0.740079607 | 0.459259485 |
| Streptozocin:Ldose: 0.602059991 (uM) Interaction | 5.20540554 | 10.69209565 | 0.486846144 | 0.626372153 |
| Sunitinib:Ldose: 0.602059991 (uM) Interaction | -0.579637125 | 10.69209565 | -0.054211741 | 0.956766952 |
| Tamoxifen.Citrate:Ldose: 0.602059991 (uM) Interaction | -6.176608577 | 10.69209565 | -0.577679884 | 0.563486096 |
| Temozolomide:Ldose: 0.602059991 (uM) Interaction | 7.16470727 | 10.96747366 | 0.653268701 | 0.513589803 |
| Temsirolimus..CCI.779..Torisel.:Ldose: 0.602059991 (uM) Interaction | -7.351593755 | 10.69209565 | -0.687572764 | 0.491729017 |
| Teniposide:Ldose: 0.602059991 (uM) Interaction | -46.27578132 | 10.69209565 | -4.328036601 | 1.51E-05 |
| Thioguanine:Ldose: 0.602059991 (uM) Interaction | -89.45357881 | 10.69209565 | -8.366327962 | 6.29E-17 |
| Thiotepa:Ldose: 0.602059991 (uM) Interaction | 3.520491658 | 10.69209565 | 0.329261145 | 0.741961403 |
| Topotecan.HCl:Ldose: 0.602059991 (uM) Interaction | -106.37325 | 10.69209565 | -9.948774631 | 2.85E-23 |
| Trametinib..GSK1120212.:Ldose: 0.602059991 (uM) Interaction | -3.523248615 | 10.69209565 | -0.329518995 | 0.741766534 |
| Tretinoin:Ldose: 0.602059991 (uM) Interaction | -1.387451577 | 10.96747366 | -0.126506032 | 0.899332515 |
| Triethylenemelamine:Ldose: 0.602059991 (uM) Interaction | -3.123575293 | 10.69209565 | -0.292138735 | 0.77018327 |
| Uracil.mustard:Ldose: 0.602059991 (uM) Interaction | -7.08930981 | 10.69209565 | -0.663042124 | 0.50731043 |
| Valrubicin:Ldose: 0.602059991 (uM) Interaction | -63.53541392 | 10.69209565 | -5.942278857 | 2.85E-09 |
| Vandetanib:Ldose: 0.602059991 (uM) Interaction | 4.835347114 | 10.60131818 | 0.456108102 | 0.648316687 |
| Vemurafenib:Ldose: 0.602059991 (uM) Interaction | -73.74712943 | 10.69209565 | -6.897350326 | 5.44E-12 |
| Vinblastine.Sulfate:Ldose: 0.602059991 (uM) Interaction | -15.58270489 | 10.69209565 | -1.45740418 | 0.145018928 |
| Vincristine.Sulfate:Ldose: 0.602059991 (uM) Interaction | -45.9040003 | 10.69209565 | -4.293265025 | 1.77E-05 |
| Vinorelbine.Tartrate:Ldose: 0.602059991 (uM) Interaction | -68.36453802 | 10.69209565 | -6.393932514 | 1.65E-10 |
| Vismodegib:Ldose: 0.602059991 (uM) Interaction | 1.897320007 | 10.69209565 | 0.177450714 | 0.859155987 |
| Vorinostat:Ldose: 0.602059991 (uM) Interaction | -70.18782354 | 10.69209565 | -6.564459002 | 5.34E-11 |
| Zoledronic.Acid:Ldose: 0.602059991 (uM) Interaction | -1.013521142 | 10.96747366 | -0.092411541 | 0.926371901 |
| Abiraterone:Ldose: 1 (uM) Interaction | 0.919805085 | 10.69209565 | 0.086026642 | 0.931446011 |
| ABT.737:Ldose: 1 (uM) Interaction | 2.961637447 | 10.69209565 | 0.276993168 | 0.78178792 |
| Actinomycin.D:Ldose: 1 (uM) Interaction | -35.92482173 | 10.69209565 | -3.359942046 | 0.000780895 |
| Afatinib:Ldose: 1 (uM) Interaction | -68.70192048 | 10.69209565 | -6.425486894 | 1.34E-10 |
| Alisertib..MLN8237.:Ldose: 1 (uM) Interaction | -25.02401701 | 10.69209565 | -2.340422105 | 0.019270725 |
| Allopurinol:Ldose: 1 (uM) Interaction | 3.812740435 | 10.69209565 | 0.356594307 | 0.721398915 |
| Amifostine:Ldose: 1 (uM) Interaction | 1.088776619 | 10.69209565 | 0.101830049 | 0.91889249 |
| Aphrocallistin.analogue:Ldose: 1 (uM) Interaction | -51.70301463 | 10.69209565 | -4.835629639 | 1.34E-06 |
| Arsenic.Trioxide:Ldose: 1 (uM) Interaction | -7.046183598 | 10.69209565 | -0.659008657 | 0.509897015 |
| Axitinib:Ldose: 1 (uM) Interaction | -29.53462726 | 10.69209565 | -2.762286106 | 0.005744516 |
| Axitinib.1:Ldose: 1 (uM) Interaction | -28.22284537 | 10.69209565 | -2.639599036 | 0.008306192 |
| Azacitidine:Ldose: 1 (uM) Interaction | -38.63648325 | 10.69209565 | -3.613555707 | 0.000302691 |
| Baricitinib..LY3009104..INCB028050.:Ldose: 1 (uM) Interaction | -18.31856968 | 10.69209565 | -1.713281501 | 0.086674666 |
| Bendamustine.HCl:Ldose: 1 (uM) Interaction | -18.39058918 | 10.60131818 | -1.734745516 | 0.08279971 |
| BGJ398..NVPBGJ398.:Ldose: 1 (uM) Interaction | -7.154124408 | 10.69209565 | -0.669104041 | 0.50343604 |
| Bioymifi:Ldose: 1 (uM) Interaction | 2.807325347 | 10.69209565 | 0.262560815 | 0.792891532 |
| Bleomycin.Sulfate:Ldose: 1 (uM) Interaction | -76.0135001 | 10.69209565 | -7.109317255 | 1.20E-12 |
| Bortezomib:Ldose: 1 (uM) Interaction | -14.36643189 | 10.69209565 | -1.343649773 | 0.17907534 |
| Bosutinib..SKI.606.:Ldose: 1 (uM) Interaction | -49.69772723 | 10.69209565 | -4.648081055 | 3.37E-06 |
| Busulfan:Ldose: 1 (uM) Interaction | -2.456286998 | 10.69209565 | -0.229729239 | 0.81830428 |
| Cabazitaxel:Ldose: 1 (uM) Interaction | 9.021899655 | 10.69209565 | 0.843791521 | 0.398794967 |
| Cabozantinib..XL.184.:Ldose: 1 (uM) Interaction | -35.22930412 | 10.69209565 | -3.294892346 | 0.000986129 |
| Capecitabine:Ldose: 1 (uM) Interaction | -3.231520871 | 10.96747366 | -0.294645875 | 0.768267157 |
| Carboplatin:Ldose: 1 (uM) Interaction | -3.455629944 | 10.96747366 | -0.315079849 | 0.752703977 |
| Carfilzomib:Ldose: 1 (uM) Interaction | -19.18889581 | 10.69209565 | -1.794680523 | 0.072718156 |
| Carmustine:Ldose: 1 (uM) Interaction | -4.829118259 | 10.96747366 | -0.440312729 | 0.659714891 |
| Celecoxib:Ldose: 1 (uM) Interaction | -3.727166107 | 10.96747366 | -0.339838163 | 0.733981607 |
| Chlorambucil:Ldose: 1 (uM) Interaction | -4.163061214 | 10.69209565 | -0.38935877 | 0.697014486 |
| Cisplatin:Ldose: 1 (uM) Interaction | -3.253148315 | 10.69209565 | -0.304257315 | 0.760934702 |
| Cladribine:Ldose: 1 (uM) Interaction | -78.03985087 | 10.69209565 | -7.298835833 | 3.00E-13 |
| Clofarabine:Ldose: 1 (uM) Interaction | -76.58821027 | 10.69209565 | -7.163068193 | 8.13E-13 |
| Crizotinib:Ldose: 1 (uM) Interaction | -64.35628682 | 10.69209565 | -6.019052665 | 1.78E-09 |
| Cytarabine.HCl...Ara.C:Ldose: 1 (uM) Interaction | -84.8460214 | 10.69209565 | -7.93539678 | 2.20E-15 |
| Dacarbazine:Ldose: 1 (uM) Interaction | -23.84703612 | 10.69209565 | -2.230342573 | 0.025734621 |
| Dacomitinib..PF299804.:Ldose: 1 (uM) Interaction | -4.510869296 | 10.69209565 | -0.421888229 | 0.67311067 |
| Dasatinib:Ldose: 1 (uM) Interaction | -28.80396757 | 10.69209565 | -2.693949672 | 0.007066368 |
| Daunorubicin.HCl:Ldose: 1 (uM) Interaction | -87.9064729 | 10.69209565 | -8.221631733 | 2.12E-16 |
| Decitabine:Ldose: 1 (uM) Interaction | -9.408733496 | 10.69209565 | -0.879970943 | 0.378884535 |
| Dexrazoxane:Ldose: 1 (uM) Interaction | -6.330677717 | 10.96747366 | -0.57722297 | 0.563794671 |
| Docetaxel:Ldose: 1 (uM) Interaction | 3.895463917 | 10.69209565 | 0.364331189 | 0.715614168 |
| Doxorubicin.HCl:Ldose: 1 (uM) Interaction | -90.69232939 | 10.69209565 | -8.48218463 | 2.35E-17 |
| Erlotinib.HCl:Ldose: 1 (uM) Interaction | -21.23366562 | 10.69209565 | -1.98592178 | 0.047054236 |
| Etoposide:Ldose: 1 (uM) Interaction | -75.17991928 | 10.69209565 | -7.031354913 | 2.10E-12 |
| Everolimus:Ldose: 1 (uM) Interaction | -3.043525954 | 10.96747366 | -0.277504742 | 0.781395138 |
| Exemestane:Ldose: 1 (uM) Interaction | -20.20437364 | 10.60131818 | -1.905835982 | 0.056684336 |
| Floxuridine:Ldose: 1 (uM) Interaction | -56.87461359 | 10.69209565 | -5.319313954 | 1.05E-07 |
| Fludarabine.Phosphate:Ldose: 1 (uM) Interaction | -51.07547823 | 10.69209565 | -4.776938021 | 1.79E-06 |
| Fluorouracil...5.FU.:Ldose: 1 (uM) Interaction | -11.93642244 | 10.69209565 | -1.116378195 | 0.264272314 |
| Flutamide..Eulexin.:Ldose: 1 (uM) Interaction | -12.1479249 | 10.69209565 | -1.136159392 | 0.255902074 |
| Foretinib..GSK1363089.:Ldose: 1 (uM) Interaction | -94.18061459 | 10.69209565 | -8.808433601 | 1.36E-18 |
| Fulvestrant:Ldose: 1 (uM) Interaction | 3.211846535 | 10.69209565 | 0.300394482 | 0.763879073 |
| Gefitinib:Ldose: 1 (uM) Interaction | -20.24134046 | 10.96747366 | -1.845579126 | 0.06496659 |
| Gemcitabine.HCl:Ldose: 1 (uM) Interaction | -40.56072616 | 10.69209565 | -3.79352444 | 0.000148916 |
| Ibrutinib..PCI.32765.:Ldose: 1 (uM) Interaction | -22.16623906 | 10.60131818 | -2.090894612 | 0.036548805 |
| Imatinib:Ldose: 1 (uM) Interaction | -11.08898268 | 10.96747366 | -1.011079034 | 0.311989576 |
| Imiquimod:Ldose: 1 (uM) Interaction | 18.69300359 | 10.60131818 | 1.76327163 | 0.077868383 |
| INK.128..MLN0128.:Ldose: 1 (uM) Interaction | -63.03011839 | 10.69209565 | -5.895020064 | 3.80E-09 |
| Irinotecan.HCl:Ldose: 1 (uM) Interaction | -72.04406373 | 10.69209565 | -6.738067642 | 1.64E-11 |
| Ixabepilone:Ldose: 1 (uM) Interaction | -82.9005905 | 10.69209565 | -7.753446397 | 9.32E-15 |
| Lapatinib:Ldose: 1 (uM) Interaction | -0.93401743 | 10.60131818 | -0.088103896 | 0.929794891 |
| LDK378:Ldose: 1 (uM) Interaction | -102.1049183 | 10.69209565 | -9.549570228 | 1.43E-21 |
| Lenalidomide:Ldose: 1 (uM) Interaction | 1.921469025 | 10.69209565 | 0.1797093 | 0.857382425 |
| Letrozole:Ldose: 1 (uM) Interaction | -3.511321901 | 10.69209565 | -0.328403525 | 0.742609662 |
| Linsitinib:Ldose: 1 (uM) Interaction | -15.07216989 | 10.69209565 | -1.409655355 | 0.158655422 |
| Lomustine..CCNU.:Ldose: 1 (uM) Interaction | 3.397093602 | 10.69209565 | 0.317720091 | 0.750700245 |
| LY2157299:Ldose: 1 (uM) Interaction | -3.247873006 | 10.69209565 | -0.303763931 | 0.761310582 |
| Mechlorethamine.HCl:Ldose: 1 (uM) Interaction | -17.24807362 | 10.69209565 | -1.613161178 | 0.106723648 |
| Megestrol.acetate:Ldose: 1 (uM) Interaction | -2.457190779 | 10.69209565 | -0.229813767 | 0.818238595 |
| MEK.162..ARRY.438162.:Ldose: 1 (uM) Interaction | -52.68443366 | 10.69209565 | -4.927418851 | 8.39E-07 |
| Melphalan:Ldose: 1 (uM) Interaction | -29.01874918 | 10.69209565 | -2.714037559 | 0.006651972 |
| Mercaptopurine:Ldose: 1 (uM) Interaction | -95.05680026 | 10.69209565 | -8.890380648 | 6.54E-19 |
| Methotrexate:Ldose: 1 (uM) Interaction | -0.237570498 | 10.69209565 | -0.022219264 | 0.98227325 |
| Mitomycin.C:Ldose: 1 (uM) Interaction | -79.52251957 | 10.69209565 | -7.43750544 | 1.06E-13 |
| Mitotane..o.p..DDD..Lysodren.:Ldose: 1 (uM) Interaction | 0.737951725 | 10.69209565 | 0.069018437 | 0.944975563 |
| Mitoxantrone:Ldose: 1 (uM) Interaction | -96.07132964 | 10.69209565 | -8.985266573 | 2.78E-19 |
| MLN.2480:Ldose: 1 (uM) Interaction | -44.55358665 | 10.69209565 | -4.166964841 | 3.10E-05 |
| MLN4924:Ldose: 1 (uM) Interaction | -86.74477831 | 10.69209565 | -8.112981883 | 5.19E-16 |
| MLN9708..MLN2238.:Ldose: 1 (uM) Interaction | -88.65901104 | 10.69209565 | -8.292014394 | 1.18E-16 |
| Navitoclax..ABT.263..5uM:Ldose: 1 (uM) Interaction | -1.746265915 | 10.69209565 | -0.163323073 | 0.870265549 |
| Nelarabine:Ldose: 1 (uM) Interaction | 10.18800714 | 10.69209565 | 0.952854096 | 0.340674331 |
| OSI.027:Ldose: 1 (uM) Interaction | -52.87409762 | 10.69209565 | -4.945157558 | 7.66E-07 |
| Oxaliplatin:Ldose: 1 (uM) Interaction | -10.72305878 | 10.60131818 | -1.011483534 | 0.311796034 |
| Paclitaxel:Ldose: 1 (uM) Interaction | -25.57155188 | 10.69209565 | -2.391631417 | 0.016781913 |
| Palbociclib..PD.0332991..Isethionate:Ldose: 1 (uM) Interaction | -34.77868904 | 10.69209565 | -3.252747654 | 0.001144649 |
| Pazopanib.HCl:Ldose: 1 (uM) Interaction | -29.94674939 | 10.69209565 | -2.800830667 | 0.005101514 |
| PD325901:Ldose: 1 (uM) Interaction | -8.699483108 | 10.69209565 | -0.813636858 | 0.415861705 |
| Pemetrexed:Ldose: 1 (uM) Interaction | -12.72698993 | 10.69209565 | -1.190317628 | 0.233934248 |
| Pipobroman:Ldose: 1 (uM) Interaction | -4.675605934 | 10.96747366 | -0.426315675 | 0.669881953 |
| Plicamycin:Ldose: 1 (uM) Interaction | -100.2193914 | 10.69209565 | -9.373222493 | 7.68E-21 |
| Pralatrexate:Ldose: 1 (uM) Interaction | 1.841601734 | 10.60131818 | 0.17371441 | 0.862091489 |
| Quinacrine.HCl:Ldose: 1 (uM) Interaction | -10.77406902 | 10.69209565 | -1.007666726 | 0.313625419 |
| Quizartinib:Ldose: 1 (uM) Interaction | -2.535971615 | 10.69209565 | -0.237181905 | 0.812517853 |
| Raloxifene:Ldose: 1 (uM) Interaction | -29.64851283 | 10.60131818 | -2.796681726 | 0.005167458 |
| Romidepsin:Ldose: 1 (uM) Interaction | 24.60356831 | 10.69209565 | 2.301098785 | 0.021395227 |
| Sabutoclax..BI.97C1.:Ldose: 1 (uM) Interaction | -98.00524652 | 10.69209565 | -9.166140083 | 5.32E-20 |
| Sirolimus..Rapamycin.:Ldose: 1 (uM) Interaction | 9.110601425 | 10.69209565 | 0.852087535 | 0.394174662 |
| Sorafenib:Ldose: 1 (uM) Interaction | -43.15232736 | 10.69209565 | -4.035909214 | 5.46E-05 |
| Streptozocin:Ldose: 1 (uM) Interaction | 8.263952982 | 10.69209565 | 0.772903017 | 0.439587973 |
| Sunitinib:Ldose: 1 (uM) Interaction | -12.29088022 | 10.69209565 | -1.149529579 | 0.250350007 |
| Tamoxifen.Citrate:Ldose: 1 (uM) Interaction | -11.13860757 | 10.69209565 | -1.041760936 | 0.297533825 |
| Temozolomide:Ldose: 1 (uM) Interaction | 13.28519593 | 10.96747366 | 1.211326905 | 0.22578298 |
| Temsirolimus..CCI.779..Torisel.:Ldose: 1 (uM) Interaction | -6.064989118 | 10.69209565 | -0.567240447 | 0.570556542 |
| Teniposide:Ldose: 1 (uM) Interaction | -52.2174243 | 10.69209565 | -4.88374085 | 1.05E-06 |
| Thioguanine:Ldose: 1 (uM) Interaction | -84.37953067 | 10.69209565 | -7.891767286 | 3.12E-15 |
| Thiotepa:Ldose: 1 (uM) Interaction | -6.697716895 | 10.69209565 | -0.6264176 | 0.531047482 |
| Topotecan.HCl:Ldose: 1 (uM) Interaction | -99.34521273 | 10.69209565 | -9.29146315 | 1.66E-20 |
| Trametinib..GSK1120212.:Ldose: 1 (uM) Interaction | 2.320459795 | 10.69209565 | 0.217025724 | 0.828190284 |
| Tretinoin:Ldose: 1 (uM) Interaction | -3.383866551 | 10.96747366 | -0.308536556 | 0.757676971 |
| Triethylenemelamine:Ldose: 1 (uM) Interaction | 1.614059252 | 10.69209565 | 0.150958176 | 0.880010074 |
| Uracil.mustard:Ldose: 1 (uM) Interaction | -14.27733236 | 10.69209565 | -1.335316558 | 0.181786394 |
| Valrubicin:Ldose: 1 (uM) Interaction | -17.75739036 | 10.69209565 | -1.660796063 | 0.096768473 |
| Vandetanib:Ldose: 1 (uM) Interaction | -27.49514468 | 10.60131818 | -2.593559047 | 0.009505031 |
| Vemurafenib:Ldose: 1 (uM) Interaction | -28.01300713 | 10.69209565 | -2.619973488 | 0.008799619 |
| Vinblastine.Sulfate:Ldose: 1 (uM) Interaction | -7.867443398 | 10.69209565 | -0.735818651 | 0.46184881 |
| Vincristine.Sulfate:Ldose: 1 (uM) Interaction | -40.52540089 | 10.69209565 | -3.790220572 | 0.00015091 |
| Vinorelbine.Tartrate:Ldose: 1 (uM) Interaction | -67.55768861 | 10.69209565 | -6.318470281 | 2.69E-10 |
| Vismodegib:Ldose: 1 (uM) Interaction | 3.469574869 | 10.69209565 | 0.324499049 | 0.745563286 |
| Vorinostat:Ldose: 1 (uM) Interaction | -83.46368982 | 10.69209565 | -7.806111407 | 6.16E-15 |
| Zoledronic.Acid:Ldose: 1 (uM) Interaction | 0.494356851 | 10.96747366 | 0.045074815 | 0.964048079 |
| MeWo:Abiraterone:Ldose: -1.397940009 (uM) Interaction | 0.625300365 | 15.13733352 | 0.041308488 | 0.967050336 |
| SKMEL2:Abiraterone:Ldose: -1.397940009 (uM) Interaction | 4.579596357 | 15.31686613 | 0.298990428 | 0.764950136 |
| MeWo:ABT.737:Ldose: -1.397940009 (uM) Interaction | -6.561152468 | 15.13733352 | -0.433441759 | 0.664698062 |
| SKMEL2:ABT.737:Ldose: -1.397940009 (uM) Interaction | -4.926241031 | 15.31686613 | -0.321621994 | 0.747742094 |
| UACC0257:ABT.737:Ldose: -1.397940009 (uM) Interaction | -14.47450299 | 15.05685414 | -0.961323186 | 0.336400118 |
| MeWo:Actinomycin.D:Ldose: -1.397940009 (uM) Interaction | 6.520776837 | 15.13733352 | 0.430774471 | 0.666636528 |
| SKMEL2:Actinomycin.D:Ldose: -1.397940009 (uM) Interaction | -29.24995206 | 15.31686613 | -1.909656441 | 0.056190283 |
| UACC0257:Actinomycin.D:Ldose: -1.397940009 (uM) Interaction | 6.972178189 | 15.05685414 | 0.463056766 | 0.643328198 |
| MeWo:Afatinib:Ldose: -1.397940009 (uM) Interaction | 7.774772715 | 15.13733352 | 0.513615737 | 0.607525764 |
| SKMEL2:Afatinib:Ldose: -1.397940009 (uM) Interaction | 10.01116052 | 15.31686613 | 0.653603709 | 0.513373894 |
| UACC0257:Afatinib:Ldose: -1.397940009 (uM) Interaction | 0.243309706 | 15.05685414 | 0.016159398 | 0.987107371 |
| MeWo:Alisertib..MLN8237.:Ldose: -1.397940009 (uM) Interaction | 1.59264534 | 15.13733352 | 0.105213071 | 0.916207686 |
| SKMEL2:Alisertib..MLN8237.:Ldose: -1.397940009 (uM) Interaction | -4.497941468 | 15.31686613 | -0.293659384 | 0.769020927 |
| MeWo:Allopurinol:Ldose: -1.397940009 (uM) Interaction | -2.75121451 | 15.13733352 | -0.181750274 | 0.855780363 |
| SKMEL2:Allopurinol:Ldose: -1.397940009 (uM) Interaction | 8.636982264 | 15.31686613 | 0.563887037 | 0.572836684 |
| UACC0257:Allopurinol:Ldose: -1.397940009 (uM) Interaction | 3.906346248 | 15.05685414 | 0.259439735 | 0.79529837 |
| MeWo:Amifostine:Ldose: -1.397940009 (uM) Interaction | 0.177781882 | 15.13733352 | 0.011744597 | 0.990629488 |
| MeWo:Aphrocallistin.analogue:Ldose: -1.397940009 (uM) Interaction | 5.244512798 | 15.13733352 | 0.346462129 | 0.72899871 |
| SKMEL2:Aphrocallistin.analogue:Ldose: -1.397940009 (uM) Interaction | 4.766574789 | 15.31686613 | 0.311197783 | 0.755653177 |
| UACC0257:Aphrocallistin.analogue:Ldose: -1.397940009 (uM) Interaction | 0.923800224 | 15.05685414 | 0.061354133 | 0.95107773 |
| MeWo:Arsenic.Trioxide:Ldose: -1.397940009 (uM) Interaction | 4.126097732 | 15.13733352 | 0.272577579 | 0.785180473 |
| SKMEL2:Arsenic.Trioxide:Ldose: -1.397940009 (uM) Interaction | 17.99980627 | 15.31686613 | 1.175162472 | 0.23994227 |
| MeWo:Axitinib:Ldose: -1.397940009 (uM) Interaction | 9.837652958 | 15.13733352 | 0.649893387 | 0.515767785 |
| SKMEL2:Axitinib:Ldose: -1.397940009 (uM) Interaction | 5.523441951 | 15.31686613 | 0.360611753 | 0.718393118 |
| UACC0257:Axitinib:Ldose: -1.397940009 (uM) Interaction | -2.538434367 | 15.05685414 | -0.168589955 | 0.866120711 |
| MeWo:Axitinib.1:Ldose: -1.397940009 (uM) Interaction | 7.212264615 | 15.13733352 | 0.476455421 | 0.633754628 |
| SKMEL2:Axitinib.1:Ldose: -1.397940009 (uM) Interaction | 9.974808819 | 15.31686613 | 0.651230398 | 0.514904482 |
| UACC0257:Axitinib.1:Ldose: -1.397940009 (uM) Interaction | -3.852343594 | 15.05685414 | -0.255853152 | 0.79806659 |
| MeWo:Azacitidine:Ldose: -1.397940009 (uM) Interaction | -4.982892502 | 15.13733352 | -0.329179013 | 0.742023477 |
| SKMEL2:Azacitidine:Ldose: -1.397940009 (uM) Interaction | 4.485716514 | 15.31686613 | 0.292861247 | 0.769630937 |
| UACC0257:Azacitidine:Ldose: -1.397940009 (uM) Interaction | 3.105301258 | 15.05685414 | 0.206238383 | 0.83660659 |
| SKMEL2:Baricitinib..LY3009104..INCB028050.:Ldose: -1.397940009 (uM) Interaction | 9.560759006 | 15.31686613 | 0.624198118 | 0.532503869 |
| UACC0257:Baricitinib..LY3009104..INCB028050.:Ldose: -1.397940009 (uM) Interaction | -3.409571944 | 15.05685414 | -0.226446502 | 0.82085624 |
| SKMEL2:Bendamustine.HCl:Ldose: -1.397940009 (uM) Interaction | 11.34025625 | 15.25363648 | 0.743446081 | 0.457219488 |
| MeWo:Bioymifi:Ldose: -1.397940009 (uM) Interaction | -3.563825178 | 15.13733352 | -0.235432824 | 0.813874974 |
| SKMEL2:Bioymifi:Ldose: -1.397940009 (uM) Interaction | -4.285915578 | 15.31686613 | -0.279816742 | 0.779620702 |
| UACC0257:Bioymifi:Ldose: -1.397940009 (uM) Interaction | -8.375714504 | 15.05685414 | -0.556272541 | 0.578030138 |
| MeWo:Bleomycin.Sulfate:Ldose: -1.397940009 (uM) Interaction | -2.500900652 | 15.13733352 | -0.165214081 | 0.86877698 |
| SKMEL2:Bleomycin.Sulfate:Ldose: -1.397940009 (uM) Interaction | -3.035357451 | 15.31686613 | -0.19817092 | 0.842913146 |
| UACC0257:Bleomycin.Sulfate:Ldose: -1.397940009 (uM) Interaction | 3.452409489 | 15.05685414 | 0.229291554 | 0.81864442 |
| MeWo:Bortezomib:Ldose: -1.397940009 (uM) Interaction | 10.55696884 | 15.13733352 | 0.697412713 | 0.48555182 |
| SKMEL2:Bortezomib:Ldose: -1.397940009 (uM) Interaction | 20.67115729 | 15.31686613 | 1.34956832 | 0.177168194 |
| UACC0257:Bortezomib:Ldose: -1.397940009 (uM) Interaction | 21.84054013 | 15.05685414 | 1.450538069 | 0.146922599 |
| MeWo:Bosutinib..SKI.606.:Ldose: -1.397940009 (uM) Interaction | 9.551379948 | 15.13733352 | 0.630981667 | 0.528058979 |
| SKMEL2:Bosutinib..SKI.606.:Ldose: -1.397940009 (uM) Interaction | 9.100019289 | 15.31686613 | 0.59411757 | 0.552439508 |
| UACC0257:Bosutinib..SKI.606.:Ldose: -1.397940009 (uM) Interaction | 4.953812206 | 15.05685414 | 0.329007119 | 0.742153397 |
| SKMEL2:Busulfan:Ldose: -1.397940009 (uM) Interaction | 7.219231059 | 15.31686613 | 0.471325596 | 0.637412851 |
| MeWo:Cabazitaxel:Ldose: -1.397940009 (uM) Interaction | 0.234700312 | 15.13733352 | 0.015504733 | 0.987629647 |
| SKMEL2:Cabazitaxel:Ldose: -1.397940009 (uM) Interaction | 0.536353162 | 15.31686613 | 0.035017161 | 0.97206637 |
| UACC0257:Cabazitaxel:Ldose: -1.397940009 (uM) Interaction | 21.29591716 | 15.05685414 | 1.41436697 | 0.157268147 |
| MeWo:Cabozantinib..XL.184.:Ldose: -1.397940009 (uM) Interaction | 2.86248496 | 15.13733352 | 0.189101004 | 0.850015376 |
| SKMEL2:Cabozantinib..XL.184.:Ldose: -1.397940009 (uM) Interaction | 4.916176547 | 15.31686613 | 0.320964909 | 0.748239992 |
| UACC0257:Cabozantinib..XL.184.:Ldose: -1.397940009 (uM) Interaction | -3.970276726 | 15.05685414 | -0.263685674 | 0.792024573 |
| MeWo:Capecitabine:Ldose: -1.397940009 (uM) Interaction | -2.219208499 | 15.33308304 | -0.144733352 | 0.884922714 |
| MeWo:Carfilzomib:Ldose: -1.397940009 (uM) Interaction | -7.754759363 | 15.13733352 | -0.512293618 | 0.608450605 |
| SKMEL2:Carfilzomib:Ldose: -1.397940009 (uM) Interaction | 36.08564898 | 15.31686613 | 2.35594205 | 0.018484407 |
| UACC0257:Carfilzomib:Ldose: -1.397940009 (uM) Interaction | 27.18957947 | 15.05685414 | 1.805794173 | 0.070963977 |
| MeWo:Carmustine:Ldose: -1.397940009 (uM) Interaction | -4.737863736 | 15.33308304 | -0.308996157 | 0.757327337 |
| MeWo:Celecoxib:Ldose: -1.397940009 (uM) Interaction | -0.849363784 | 15.33308304 | -0.055394195 | 0.955824917 |
| UACC0257:Chlorambucil:Ldose: -1.397940009 (uM) Interaction | -11.70329118 | 15.05685414 | -0.777273331 | 0.437005772 |
| MeWo:Cisplatin:Ldose: -1.397940009 (uM) Interaction | 7.302805598 | 15.13733352 | 0.482436724 | 0.629500474 |
| SKMEL2:Cisplatin:Ldose: -1.397940009 (uM) Interaction | 9.465183068 | 15.31686613 | 0.617958203 | 0.536609203 |
| MeWo:Cladribine:Ldose: -1.397940009 (uM) Interaction | 10.4835212 | 15.13733352 | 0.692560627 | 0.488592539 |
| SKMEL2:Cladribine:Ldose: -1.397940009 (uM) Interaction | 12.82451435 | 15.31686613 | 0.837280566 | 0.402443855 |
| UACC0257:Cladribine:Ldose: -1.397940009 (uM) Interaction | 15.26658035 | 15.05685414 | 1.013928953 | 0.310627658 |
| MeWo:Clofarabine:Ldose: -1.397940009 (uM) Interaction | 24.96684913 | 15.13733352 | 1.649355819 | 0.099088848 |
| SKMEL2:Clofarabine:Ldose: -1.397940009 (uM) Interaction | 28.77091791 | 15.31686613 | 1.878381496 | 0.060342033 |
| UACC0257:Clofarabine:Ldose: -1.397940009 (uM) Interaction | 22.581986 | 15.05685414 | 1.499781149 | 0.133685261 |
| MeWo:Crizotinib:Ldose: -1.397940009 (uM) Interaction | -0.177248232 | 15.13733352 | -0.011709343 | 0.990657614 |
| SKMEL2:Crizotinib:Ldose: -1.397940009 (uM) Interaction | 1.294076356 | 15.31686613 | 0.084487019 | 0.932669982 |
| UACC0257:Crizotinib:Ldose: -1.397940009 (uM) Interaction | -7.417119374 | 15.05685414 | -0.492607507 | 0.622294794 |
| MeWo:Cytarabine.HCl...Ara.C:Ldose: -1.397940009 (uM) Interaction | 5.010241773 | 15.13733352 | 0.330985756 | 0.740658356 |
| SKMEL2:Cytarabine.HCl...Ara.C:Ldose: -1.397940009 (uM) Interaction | -0.378873344 | 15.31686613 | -0.024735696 | 0.980266004 |
| UACC0257:Cytarabine.HCl...Ara.C:Ldose: -1.397940009 (uM) Interaction | -2.125614019 | 15.05685414 | -0.141172518 | 0.887734931 |
| MeWo:Dacarbazine:Ldose: -1.397940009 (uM) Interaction | 10.13393153 | 15.13733352 | 0.669466093 | 0.503205135 |
| SKMEL2:Dacarbazine:Ldose: -1.397940009 (uM) Interaction | 15.84059066 | 15.31686613 | 1.034192669 | 0.301057318 |
| MeWo:Dacomitinib..PF299804.:Ldose: -1.397940009 (uM) Interaction | -6.326758937 | 15.13733352 | -0.417957293 | 0.675982368 |
| SKMEL2:Dacomitinib..PF299804.:Ldose: -1.397940009 (uM) Interaction | -3.366560298 | 15.31686613 | -0.219794328 | 0.826033349 |
| UACC0257:Dacomitinib..PF299804.:Ldose: -1.397940009 (uM) Interaction | 1.780872121 | 15.05685414 | 0.118276507 | 0.905849637 |
| MeWo:Dasatinib:Ldose: -1.397940009 (uM) Interaction | -5.622764915 | 15.13733352 | -0.371450157 | 0.710305803 |
| SKMEL2:Dasatinib:Ldose: -1.397940009 (uM) Interaction | -7.200765511 | 15.31686613 | -0.470120027 | 0.638273865 |
| UACC0257:Dasatinib:Ldose: -1.397940009 (uM) Interaction | 0.872712443 | 15.05685414 | 0.057961141 | 0.953780101 |
| MeWo:Daunorubicin.HCl:Ldose: -1.397940009 (uM) Interaction | 12.82975061 | 15.13733352 | 0.847556843 | 0.396693916 |
| SKMEL2:Daunorubicin.HCl:Ldose: -1.397940009 (uM) Interaction | 5.641063616 | 15.31686613 | 0.368290979 | 0.712659775 |
| UACC0257:Daunorubicin.HCl:Ldose: -1.397940009 (uM) Interaction | 9.158133121 | 15.05685414 | 0.608236823 | 0.543036611 |
| MeWo:Decitabine:Ldose: -1.397940009 (uM) Interaction | 3.666927227 | 15.13733352 | 0.242243934 | 0.808593382 |
| SKMEL2:Decitabine:Ldose: -1.397940009 (uM) Interaction | 5.284242749 | 15.31686613 | 0.344995034 | 0.730101358 |
| MeWo:Docetaxel:Ldose: -1.397940009 (uM) Interaction | 2.419044397 | 15.13733352 | 0.159806507 | 0.873034946 |
| MeWo:Doxorubicin.HCl:Ldose: -1.397940009 (uM) Interaction | 4.658518295 | 15.13733352 | 0.307750258 | 0.758275249 |
| SKMEL2:Doxorubicin.HCl:Ldose: -1.397940009 (uM) Interaction | 13.21035021 | 15.31686613 | 0.862470828 | 0.388437756 |
| UACC0257:Doxorubicin.HCl:Ldose: -1.397940009 (uM) Interaction | 12.87832358 | 15.05685414 | 0.855313033 | 0.392387065 |
| MeWo:Erlotinib.HCl:Ldose: -1.397940009 (uM) Interaction | -8.639112178 | 15.13733352 | -0.570715586 | 0.568198203 |
| SKMEL2:Erlotinib.HCl:Ldose: -1.397940009 (uM) Interaction | -8.762008094 | 15.31686613 | -0.572049662 | 0.567294098 |
| UACC0257:Erlotinib.HCl:Ldose: -1.397940009 (uM) Interaction | -6.746333954 | 15.05685414 | -0.448057336 | 0.654116171 |
| MeWo:Etoposide:Ldose: -1.397940009 (uM) Interaction | 0.999146947 | 15.13733352 | 0.066005479 | 0.947374055 |
| SKMEL2:Etoposide:Ldose: -1.397940009 (uM) Interaction | 8.626140204 | 15.31686613 | 0.563179186 | 0.573318538 |
| UACC0257:Etoposide:Ldose: -1.397940009 (uM) Interaction | 1.757106621 | 15.05685414 | 0.116698123 | 0.907100329 |
| MeWo:Everolimus:Ldose: -1.397940009 (uM) Interaction | -4.203238704 | 15.33308304 | -0.274128738 | 0.783988229 |
| MeWo:Exemestane:Ldose: -1.397940009 (uM) Interaction | 6.070694076 | 15.07335079 | 0.402743501 | 0.68714079 |
| SKMEL2:Exemestane:Ldose: -1.397940009 (uM) Interaction | 12.49125934 | 15.25363648 | 0.818903699 | 0.412850112 |
| MeWo:Floxuridine:Ldose: -1.397940009 (uM) Interaction | 19.83075304 | 15.13733352 | 1.310055897 | 0.190190424 |
| SKMEL2:Floxuridine:Ldose: -1.397940009 (uM) Interaction | 23.21448466 | 15.31686613 | 1.515615823 | 0.1296307 |
| UACC0257:Floxuridine:Ldose: -1.397940009 (uM) Interaction | 18.27202062 | 15.05685414 | 1.213535075 | 0.224938168 |
| MeWo:Fludarabine.Phosphate:Ldose: -1.397940009 (uM) Interaction | -6.38013414 | 15.13733352 | -0.421483356 | 0.673406225 |
| SKMEL2:Fludarabine.Phosphate:Ldose: -1.397940009 (uM) Interaction | -8.374629458 | 15.31686613 | -0.546758677 | 0.584550008 |
| MeWo:Fluorouracil...5.FU.:Ldose: -1.397940009 (uM) Interaction | 3.223868613 | 15.13733352 | 0.21297467 | 0.831348667 |
| SKMEL2:Fluorouracil...5.FU.:Ldose: -1.397940009 (uM) Interaction | 3.538404716 | 15.31686613 | 0.231013622 | 0.817306339 |
| UACC0257:Fluorouracil...5.FU.:Ldose: -1.397940009 (uM) Interaction | 4.360362154 | 15.05685414 | 0.289593172 | 0.772130184 |
| MeWo:Flutamide..Eulexin.:Ldose: -1.397940009 (uM) Interaction | 2.884573515 | 15.13733352 | 0.190560214 | 0.848871899 |
| SKMEL2:Flutamide..Eulexin.:Ldose: -1.397940009 (uM) Interaction | 2.999615327 | 15.31686613 | 0.195837406 | 0.844739216 |
| UACC0257:Flutamide..Eulexin.:Ldose: -1.397940009 (uM) Interaction | -10.25485394 | 15.05685414 | -0.681075466 | 0.495830814 |
| MeWo:Foretinib..GSK1363089.:Ldose: -1.397940009 (uM) Interaction | -4.888181955 | 15.13733352 | -0.32292226 | 0.746757146 |
| SKMEL2:Foretinib..GSK1363089.:Ldose: -1.397940009 (uM) Interaction | 9.856948841 | 15.31686613 | 0.643535614 | 0.519883233 |
| UACC0257:Foretinib..GSK1363089.:Ldose: -1.397940009 (uM) Interaction | 3.152991106 | 15.05685414 | 0.209405702 | 0.834133455 |
| SKMEL2:Fulvestrant:Ldose: -1.397940009 (uM) Interaction | -6.393011141 | 15.31686613 | -0.417383758 | 0.676401752 |
| UACC0257:Fulvestrant:Ldose: -1.397940009 (uM) Interaction | 7.850863606 | 15.05685414 | 0.521414602 | 0.602083161 |
| MeWo:Gefitinib:Ldose: -1.397940009 (uM) Interaction | 6.345868183 | 15.33308304 | 0.413867724 | 0.678974963 |
| MeWo:Gemcitabine.HCl:Ldose: -1.397940009 (uM) Interaction | 2.172225397 | 15.13733352 | 0.143501191 | 0.885895667 |
| SKMEL2:Gemcitabine.HCl:Ldose: -1.397940009 (uM) Interaction | 2.578898481 | 15.31686613 | 0.168369852 | 0.866293851 |
| UACC0257:Gemcitabine.HCl:Ldose: -1.397940009 (uM) Interaction | 8.619268571 | 15.05685414 | 0.572448168 | 0.567024163 |
| MeWo:Ibrutinib..PCI.32765.:Ldose: -1.397940009 (uM) Interaction | -2.002585424 | 15.07335079 | -0.132856022 | 0.894308447 |
| SKMEL2:Ibrutinib..PCI.32765.:Ldose: -1.397940009 (uM) Interaction | 0.894555991 | 15.25363648 | 0.058645425 | 0.953235055 |
| MeWo:Imiquimod:Ldose: -1.397940009 (uM) Interaction | -11.77227096 | 15.07335079 | -0.780998938 | 0.434811411 |
| SKMEL2:Imiquimod:Ldose: -1.397940009 (uM) Interaction | -9.883018189 | 15.25363648 | -0.647912267 | 0.517048368 |
| MeWo:INK.128..MLN0128.:Ldose: -1.397940009 (uM) Interaction | 2.731425645 | 15.13733352 | 0.180442985 | 0.856806451 |
| SKMEL2:INK.128..MLN0128.:Ldose: -1.397940009 (uM) Interaction | 8.200595401 | 15.31686613 | 0.535396427 | 0.5923811 |
| UACC0257:INK.128..MLN0128.:Ldose: -1.397940009 (uM) Interaction | 6.095112144 | 15.05685414 | 0.404806481 | 0.685623648 |
| MeWo:Irinotecan.HCl:Ldose: -1.397940009 (uM) Interaction | 9.428941345 | 15.13733352 | 0.622893149 | 0.533361111 |
| SKMEL2:Irinotecan.HCl:Ldose: -1.397940009 (uM) Interaction | 4.840331306 | 15.31686613 | 0.316013163 | 0.751995475 |
| UACC0257:Irinotecan.HCl:Ldose: -1.397940009 (uM) Interaction | 7.074388491 | 15.05685414 | 0.469845057 | 0.638470316 |
| MeWo:Ixabepilone:Ldose: -1.397940009 (uM) Interaction | 17.99133786 | 15.13733352 | 1.188540758 | 0.234633091 |
| SKMEL2:Ixabepilone:Ldose: -1.397940009 (uM) Interaction | 8.035515856 | 15.31686613 | 0.524618795 | 0.599853434 |
| UACC0257:Ixabepilone:Ldose: -1.397940009 (uM) Interaction | 15.20795166 | 15.05685414 | 1.010035132 | 0.312489419 |
| MeWo:Lapatinib:Ldose: -1.397940009 (uM) Interaction | -12.99083079 | 15.07335079 | -0.861840939 | 0.388784322 |
| SKMEL2:Lapatinib:Ldose: -1.397940009 (uM) Interaction | -13.60597928 | 15.25363648 | -0.891982663 | 0.372411851 |
| MeWo:LDK378:Ldose: -1.397940009 (uM) Interaction | 10.75194838 | 15.13733352 | 0.710293419 | 0.477529629 |
| SKMEL2:LDK378:Ldose: -1.397940009 (uM) Interaction | 13.02011554 | 15.31686613 | 0.850050881 | 0.395305927 |
| UACC0257:LDK378:Ldose: -1.397940009 (uM) Interaction | -0.927322244 | 15.05685414 | -0.061588047 | 0.950891448 |
| MeWo:Lenalidomide:Ldose: -1.397940009 (uM) Interaction | -2.373667508 | 15.13733352 | -0.156808827 | 0.875396936 |
| SKMEL2:Lenalidomide:Ldose: -1.397940009 (uM) Interaction | -8.263125153 | 15.31686613 | -0.539478839 | 0.589561882 |
| SKMEL2:Letrozole:Ldose: -1.397940009 (uM) Interaction | 7.822112477 | 15.31686613 | 0.510686221 | 0.609575847 |
| MeWo:Linsitinib:Ldose: -1.397940009 (uM) Interaction | -0.295927118 | 15.13733352 | -0.019549488 | 0.984402934 |
| SKMEL2:Linsitinib:Ldose: -1.397940009 (uM) Interaction | -2.262615813 | 15.31686613 | -0.147720545 | 0.882564653 |
| UACC0257:Linsitinib:Ldose: -1.397940009 (uM) Interaction | -10.96801695 | 15.05685414 | -0.728440141 | 0.466351849 |
| SKMEL2:Lomustine..CCNU.:Ldose: -1.397940009 (uM) Interaction | -2.115767974 | 15.31686613 | -0.138133216 | 0.890136382 |
| MeWo:LY2157299:Ldose: -1.397940009 (uM) Interaction | 1.034990782 | 15.13733352 | 0.068373388 | 0.945489018 |
| SKMEL2:LY2157299:Ldose: -1.397940009 (uM) Interaction | 7.399099457 | 15.31686613 | 0.483068755 | 0.629051661 |
| MeWo:Mechlorethamine.HCl:Ldose: -1.397940009 (uM) Interaction | -0.526482533 | 15.13733352 | -0.034780401 | 0.97225516 |
| SKMEL2:Mechlorethamine.HCl:Ldose: -1.397940009 (uM) Interaction | -0.448798798 | 15.31686613 | -0.029300955 | 0.976624828 |
| UACC0257:Mechlorethamine.HCl:Ldose: -1.397940009 (uM) Interaction | 4.092619691 | 15.05685414 | 0.271811074 | 0.785769807 |
| MeWo:Megestrol.acetate:Ldose: -1.397940009 (uM) Interaction | 1.92944198 | 15.13733352 | 0.127462474 | 0.898575522 |
| SKMEL2:Megestrol.acetate:Ldose: -1.397940009 (uM) Interaction | 6.686797737 | 15.31686613 | 0.436564352 | 0.662431547 |
| MeWo:MEK.162..ARRY.438162.:Ldose: -1.397940009 (uM) Interaction | 16.7173527 | 15.13733352 | 1.104378963 | 0.269440701 |
| SKMEL2:MEK.162..ARRY.438162.:Ldose: -1.397940009 (uM) Interaction | 15.6450359 | 15.31686613 | 1.021425386 | 0.307064025 |
| UACC0257:MEK.162..ARRY.438162.:Ldose: -1.397940009 (uM) Interaction | 15.23268579 | 15.05685414 | 1.011677848 | 0.311703089 |
| MeWo:Melphalan:Ldose: -1.397940009 (uM) Interaction | 6.784212282 | 15.13733352 | 0.448177499 | 0.654029455 |
| SKMEL2:Melphalan:Ldose: -1.397940009 (uM) Interaction | 3.992621689 | 15.31686613 | 0.260668315 | 0.79435071 |
| UACC0257:Melphalan:Ldose: -1.397940009 (uM) Interaction | 4.687874631 | 15.05685414 | 0.311344892 | 0.755541354 |
| MeWo:Mercaptopurine:Ldose: -1.397940009 (uM) Interaction | 2.573680745 | 15.13733352 | 0.170022068 | 0.864994324 |
| SKMEL2:Mercaptopurine:Ldose: -1.397940009 (uM) Interaction | 3.636346436 | 15.31686613 | 0.237407992 | 0.812342471 |
| UACC0257:Mercaptopurine:Ldose: -1.397940009 (uM) Interaction | 14.12850385 | 15.05685414 | 0.938343675 | 0.348077986 |
| MeWo:Mitomycin.C:Ldose: -1.397940009 (uM) Interaction | -7.300872192 | 15.13733352 | -0.482309 | 0.62959119 |
| SKMEL2:Mitomycin.C:Ldose: -1.397940009 (uM) Interaction | -11.6930579 | 15.31686613 | -0.763410596 | 0.44522664 |
| UACC0257:Mitomycin.C:Ldose: -1.397940009 (uM) Interaction | -13.16828917 | 15.05685414 | -0.874571079 | 0.38181675 |
| MeWo:Mitotane..o.p..DDD..Lysodren.:Ldose: -1.397940009 (uM) Interaction | 3.5514203 | 15.13733352 | 0.234613335 | 0.814511013 |
| SKMEL2:Mitotane..o.p..DDD..Lysodren.:Ldose: -1.397940009 (uM) Interaction | 5.332119456 | 15.31686613 | 0.348120785 | 0.727752762 |
| MeWo:Mitoxantrone:Ldose: -1.397940009 (uM) Interaction | 23.31329203 | 15.13733352 | 1.540118806 | 0.123545588 |
| SKMEL2:Mitoxantrone:Ldose: -1.397940009 (uM) Interaction | 30.74464331 | 15.31686613 | 2.007241106 | 0.044736043 |
| UACC0257:Mitoxantrone:Ldose: -1.397940009 (uM) Interaction | 22.37184435 | 15.05685414 | 1.485824605 | 0.137339659 |
| UACC0257:MLN.2480:Ldose: -1.397940009 (uM) Interaction | -3.455892432 | 15.05685414 | -0.229522874 | 0.81846465 |
| MeWo:MLN4924:Ldose: -1.397940009 (uM) Interaction | -0.666642368 | 15.13733352 | -0.044039617 | 0.964873219 |
| SKMEL2:MLN4924:Ldose: -1.397940009 (uM) Interaction | 8.235554724 | 15.31686613 | 0.537678834 | 0.59080416 |
| UACC0257:MLN4924:Ldose: -1.397940009 (uM) Interaction | -4.691486966 | 15.05685414 | -0.311584805 | 0.755358997 |
| MeWo:MLN9708..MLN2238.:Ldose: -1.397940009 (uM) Interaction | -24.14087566 | 15.13733352 | -1.594790497 | 0.110773267 |
| SKMEL2:MLN9708..MLN2238.:Ldose: -1.397940009 (uM) Interaction | -12.08731549 | 15.31686613 | -0.789150691 | 0.430032329 |
| UACC0257:MLN9708..MLN2238.:Ldose: -1.397940009 (uM) Interaction | -17.68464348 | 15.05685414 | -1.17452446 | 0.240197564 |
| MeWo:Navitoclax..ABT.263..5uM:Ldose: -1.397940009 (uM) Interaction | 0.599778715 | 15.13733352 | 0.039622481 | 0.968394459 |
| SKMEL2:Navitoclax..ABT.263..5uM:Ldose: -1.397940009 (uM) Interaction | -2.240690863 | 15.31686613 | -0.14628912 | 0.883694477 |
| UACC0257:Navitoclax..ABT.263..5uM:Ldose: -1.397940009 (uM) Interaction | 10.66685925 | 15.05685414 | 0.708438772 | 0.478680229 |
| MeWo:Nelarabine:Ldose: -1.397940009 (uM) Interaction | -1.379850648 | 15.13733352 | -0.091155463 | 0.927369882 |
| SKMEL2:Nelarabine:Ldose: -1.397940009 (uM) Interaction | 5.824445451 | 15.31686613 | 0.380263521 | 0.703753433 |
| UACC0257:Nelarabine:Ldose: -1.397940009 (uM) Interaction | 3.989075246 | 15.05685414 | 0.264934176 | 0.791062619 |
| MeWo:OSI.027:Ldose: -1.397940009 (uM) Interaction | -15.152637 | 15.13733352 | -1.001010976 | 0.316832366 |
| SKMEL2:OSI.027:Ldose: -1.397940009 (uM) Interaction | -13.27604373 | 15.31686613 | -0.866759794 | 0.386082963 |
| UACC0257:OSI.027:Ldose: -1.397940009 (uM) Interaction | -13.03353031 | 15.05685414 | -0.865621078 | 0.386707307 |
| MeWo:Oxaliplatin:Ldose: -1.397940009 (uM) Interaction | 4.180494759 | 15.07335079 | 0.277343427 | 0.781518988 |
| SKMEL2:Oxaliplatin:Ldose: -1.397940009 (uM) Interaction | 8.822741358 | 15.25363648 | 0.578402493 | 0.562998254 |
| MeWo:Paclitaxel:Ldose: -1.397940009 (uM) Interaction | 11.3949177 | 15.13733352 | 0.752769151 | 0.451596595 |
| SKMEL2:Paclitaxel:Ldose: -1.397940009 (uM) Interaction | 13.4060701 | 15.31686613 | 0.875248891 | 0.381447925 |
| MeWo:Palbociclib..PD.0332991..Isethionate:Ldose: -1.397940009 (uM) Interaction | 1.590836958 | 15.13733352 | 0.105093606 | 0.916302479 |
| SKMEL2:Palbociclib..PD.0332991..Isethionate:Ldose: -1.397940009 (uM) Interaction | -0.243881568 | 15.31686613 | -0.015922419 | 0.987296427 |
| UACC0257:Palbociclib..PD.0332991..Isethionate:Ldose: -1.397940009 (uM) Interaction | 4.591656899 | 15.05685414 | 0.304954598 | 0.760403578 |
| MeWo:Pazopanib.HCl:Ldose: -1.397940009 (uM) Interaction | 3.305581232 | 15.13733352 | 0.218372755 | 0.82714069 |
| SKMEL2:Pazopanib.HCl:Ldose: -1.397940009 (uM) Interaction | 8.094862896 | 15.31686613 | 0.528493416 | 0.597162176 |
| UACC0257:Pazopanib.HCl:Ldose: -1.397940009 (uM) Interaction | 13.58081032 | 15.05685414 | 0.901968645 | 0.367083268 |
| MeWo:PD325901:Ldose: -1.397940009 (uM) Interaction | 1.644764683 | 15.13733352 | 0.10865617 | 0.913476185 |
| SKMEL2:PD325901:Ldose: -1.397940009 (uM) Interaction | -0.249551644 | 15.31686613 | -0.016292605 | 0.987001103 |
| UACC0257:PD325901:Ldose: -1.397940009 (uM) Interaction | 9.408888986 | 15.05685414 | 0.624890757 | 0.532049154 |
| SKMEL2:Pemetrexed:Ldose: -1.397940009 (uM) Interaction | 6.577988916 | 15.31686613 | 0.429460495 | 0.667592287 |
| MeWo:Plicamycin:Ldose: -1.397940009 (uM) Interaction | 6.457511023 | 15.13733352 | 0.426595015 | 0.669678448 |
| SKMEL2:Plicamycin:Ldose: -1.397940009 (uM) Interaction | -5.511209303 | 15.31686613 | -0.359813114 | 0.718990303 |
| UACC0257:Plicamycin:Ldose: -1.397940009 (uM) Interaction | 22.54714056 | 15.05685414 | 1.497466892 | 0.13428597 |
| MeWo:Pralatrexate:Ldose: -1.397940009 (uM) Interaction | 1.672130539 | 15.07335079 | 0.110932902 | 0.911670553 |
| SKMEL2:Pralatrexate:Ldose: -1.397940009 (uM) Interaction | 4.655071208 | 15.25363648 | 0.305177799 | 0.760233589 |
| MeWo:Quinacrine.HCl:Ldose: -1.397940009 (uM) Interaction | -2.642096555 | 15.13733352 | -0.174541742 | 0.861441314 |
| SKMEL2:Quinacrine.HCl:Ldose: -1.397940009 (uM) Interaction | -5.419972679 | 15.31686613 | -0.353856503 | 0.723449772 |
| UACC0257:Quinacrine.HCl:Ldose: -1.397940009 (uM) Interaction | 6.468844619 | 15.05685414 | 0.4296279 | 0.66747049 |
| MeWo:Quizartinib:Ldose: -1.397940009 (uM) Interaction | 2.060585948 | 15.13733352 | 0.136126085 | 0.891722836 |
| SKMEL2:Quizartinib:Ldose: -1.397940009 (uM) Interaction | -2.717867254 | 15.31686613 | -0.177442777 | 0.859162221 |
| UACC0257:Quizartinib:Ldose: -1.397940009 (uM) Interaction | -13.12649068 | 15.05685414 | -0.871795035 | 0.383329591 |
| MeWo:Raloxifene:Ldose: -1.397940009 (uM) Interaction | 9.188352159 | 15.07335079 | 0.609575952 | 0.542148957 |
| SKMEL2:Raloxifene:Ldose: -1.397940009 (uM) Interaction | 10.49103264 | 15.25363648 | 0.687772562 | 0.491603172 |
| MeWo:Romidepsin:Ldose: -1.397940009 (uM) Interaction | 8.15810545 | 15.13733352 | 0.5389394 | 0.589934051 |
| SKMEL2:Romidepsin:Ldose: -1.397940009 (uM) Interaction | 11.20672417 | 15.31686613 | 0.731659079 | 0.464384368 |
| UACC0257:Romidepsin:Ldose: -1.397940009 (uM) Interaction | 18.77674339 | 15.05685414 | 1.247056206 | 0.212389989 |
| MeWo:Sabutoclax..BI.97C1.:Ldose: -1.397940009 (uM) Interaction | 6.684845902 | 15.13733352 | 0.441613174 | 0.658773432 |
| SKMEL2:Sabutoclax..BI.97C1.:Ldose: -1.397940009 (uM) Interaction | 6.889224864 | 15.31686613 | 0.449780314 | 0.652873226 |
| UACC0257:Sabutoclax..BI.97C1.:Ldose: -1.397940009 (uM) Interaction | 12.13002531 | 15.05685414 | 0.805614851 | 0.420473551 |
| MeWo:Sirolimus..Rapamycin.:Ldose: -1.397940009 (uM) Interaction | -5.93960336 | 15.13733352 | -0.392381086 | 0.69478041 |
| SKMEL2:Sirolimus..Rapamycin.:Ldose: -1.397940009 (uM) Interaction | -0.457415018 | 15.31686613 | -0.029863486 | 0.976176194 |
| MeWo:Sorafenib:Ldose: -1.397940009 (uM) Interaction | 6.396630592 | 15.13733352 | 0.422573142 | 0.672610801 |
| UACC0257:Sorafenib:Ldose: -1.397940009 (uM) Interaction | 7.174609766 | 15.05685414 | 0.476501247 | 0.633721989 |
| MeWo:Streptozocin:Ldose: -1.397940009 (uM) Interaction | -0.662875817 | 15.13733352 | -0.043790792 | 0.965071559 |
| SKMEL2:Streptozocin:Ldose: -1.397940009 (uM) Interaction | 6.385508659 | 15.31686613 | 0.416893939 | 0.67676 |
| UACC0257:Streptozocin:Ldose: -1.397940009 (uM) Interaction | 10.33757011 | 15.05685414 | 0.686569055 | 0.492361475 |
| MeWo:Sunitinib:Ldose: -1.397940009 (uM) Interaction | -4.517559168 | 15.13733352 | -0.29843824 | 0.765371488 |
| UACC0257:Sunitinib:Ldose: -1.397940009 (uM) Interaction | 5.495273623 | 15.05685414 | 0.364968244 | 0.715138573 |
| MeWo:Tamoxifen.Citrate:Ldose: -1.397940009 (uM) Interaction | 6.455523765 | 15.13733352 | 0.426463733 | 0.669774087 |
| SKMEL2:Tamoxifen.Citrate:Ldose: -1.397940009 (uM) Interaction | 12.53138949 | 15.31686613 | 0.818143175 | 0.413284181 |
| UACC0257:Tamoxifen.Citrate:Ldose: -1.397940009 (uM) Interaction | 1.587767373 | 15.05685414 | 0.105451468 | 0.916018527 |
| MeWo:Temozolomide:Ldose: -1.397940009 (uM) Interaction | 2.773784943 | 15.33308304 | 0.180901971 | 0.856446166 |
| SKMEL2:Temsirolimus..CCI.779..Torisel.:Ldose: -1.397940009 (uM) Interaction | 6.274883587 | 15.31686613 | 0.409671504 | 0.682050872 |
| MeWo:Teniposide:Ldose: -1.397940009 (uM) Interaction | 7.903756232 | 15.13733352 | 0.522136625 | 0.601580395 |
| SKMEL2:Teniposide:Ldose: -1.397940009 (uM) Interaction | 11.48874819 | 15.31686613 | 0.750071724 | 0.453219423 |
| UACC0257:Teniposide:Ldose: -1.397940009 (uM) Interaction | 16.38911357 | 15.05685414 | 1.088481925 | 0.276394177 |
| MeWo:Thioguanine:Ldose: -1.397940009 (uM) Interaction | 4.922591787 | 15.13733352 | 0.325195437 | 0.745036213 |
| SKMEL2:Thioguanine:Ldose: -1.397940009 (uM) Interaction | 3.428114737 | 15.31686613 | 0.223813064 | 0.822904815 |
| UACC0257:Thioguanine:Ldose: -1.397940009 (uM) Interaction | -1.185758016 | 15.05685414 | -0.078752042 | 0.937230557 |
| MeWo:Thiotepa:Ldose: -1.397940009 (uM) Interaction | -5.472586183 | 15.13733352 | -0.361529075 | 0.7177074 |
| SKMEL2:Thiotepa:Ldose: -1.397940009 (uM) Interaction | -6.193262823 | 15.31686613 | -0.404342688 | 0.685964617 |
| UACC0257:Thiotepa:Ldose: -1.397940009 (uM) Interaction | 6.858514441 | 15.05685414 | 0.455507796 | 0.648748396 |
| MeWo:Topotecan.HCl:Ldose: -1.397940009 (uM) Interaction | 18.92266812 | 15.13733352 | 1.250066142 | 0.211288507 |
| SKMEL2:Topotecan.HCl:Ldose: -1.397940009 (uM) Interaction | 16.03182469 | 15.31686613 | 1.046677862 | 0.295259524 |
| UACC0257:Topotecan.HCl:Ldose: -1.397940009 (uM) Interaction | 15.26278063 | 15.05685414 | 1.013676595 | 0.310748096 |
| MeWo:Trametinib..GSK1120212.:Ldose: -1.397940009 (uM) Interaction | -7.877214015 | 15.13733352 | -0.520383197 | 0.602801686 |
| UACC0257:Trametinib..GSK1120212.:Ldose: -1.397940009 (uM) Interaction | 9.735097476 | 15.05685414 | 0.646555873 | 0.51792608 |
| MeWo:Triethylenemelamine:Ldose: -1.397940009 (uM) Interaction | 0.449239798 | 15.13733352 | 0.029677605 | 0.976324439 |
| SKMEL2:Triethylenemelamine:Ldose: -1.397940009 (uM) Interaction | -0.643193486 | 15.31686613 | -0.041992499 | 0.966505054 |
| UACC0257:Triethylenemelamine:Ldose: -1.397940009 (uM) Interaction | -1.958743577 | 15.05685414 | -0.130089829 | 0.896496531 |
| MeWo:Uracil.mustard:Ldose: -1.397940009 (uM) Interaction | 6.505004248 | 15.13733352 | 0.429732505 | 0.667394389 |
| SKMEL2:Uracil.mustard:Ldose: -1.397940009 (uM) Interaction | 6.500322372 | 15.31686613 | 0.42438984 | 0.671285624 |
| MeWo:Valrubicin:Ldose: -1.397940009 (uM) Interaction | -1.881095953 | 15.13733352 | -0.124268647 | 0.901103691 |
| SKMEL2:Valrubicin:Ldose: -1.397940009 (uM) Interaction | -10.25044582 | 15.31686613 | -0.66922605 | 0.50335822 |
| UACC0257:Valrubicin:Ldose: -1.397940009 (uM) Interaction | 2.226203904 | 15.05685414 | 0.147853189 | 0.882459969 |
| MeWo:Vandetanib:Ldose: -1.397940009 (uM) Interaction | -9.143030958 | 15.07335079 | -0.606569242 | 0.544142994 |
| SKMEL2:Vandetanib:Ldose: -1.397940009 (uM) Interaction | -7.464357275 | 15.25363648 | -0.489349362 | 0.624599194 |
| SKMEL2:Vemurafenib:Ldose: -1.397940009 (uM) Interaction | 17.50586723 | 15.31686613 | 1.142914424 | 0.253086395 |
| UACC0257:Vemurafenib:Ldose: -1.397940009 (uM) Interaction | 14.86331194 | 15.05685414 | 0.987145907 | 0.323581827 |
| SKMEL2:Vinblastine.Sulfate:Ldose: -1.397940009 (uM) Interaction | 5.736741081 | 15.31686613 | 0.374537522 | 0.708008008 |
| UACC0257:Vinblastine.Sulfate:Ldose: -1.397940009 (uM) Interaction | 14.06462147 | 15.05685414 | 0.934100931 | 0.350261948 |
| MeWo:Vincristine.Sulfate:Ldose: -1.397940009 (uM) Interaction | 11.83212778 | 15.13733352 | 0.78165205 | 0.434427389 |
| SKMEL2:Vincristine.Sulfate:Ldose: -1.397940009 (uM) Interaction | 32.55575782 | 15.31686613 | 2.125484257 | 0.033557188 |
| UACC0257:Vincristine.Sulfate:Ldose: -1.397940009 (uM) Interaction | 41.09764979 | 15.05685414 | 2.72949777 | 0.006348064 |
| MeWo:Vinorelbine.Tartrate:Ldose: -1.397940009 (uM) Interaction | 1.282463992 | 15.13733352 | 0.084721922 | 0.932483228 |
| SKMEL2:Vinorelbine.Tartrate:Ldose: -1.397940009 (uM) Interaction | 3.158399626 | 15.31686613 | 0.206204037 | 0.836633418 |
| UACC0257:Vinorelbine.Tartrate:Ldose: -1.397940009 (uM) Interaction | 2.270175588 | 15.05685414 | 0.150773566 | 0.880155703 |
| MeWo:Vismodegib:Ldose: -1.397940009 (uM) Interaction | 0.735184282 | 15.13733352 | 0.048567621 | 0.961264309 |
| SKMEL2:Vismodegib:Ldose: -1.397940009 (uM) Interaction | 8.092038742 | 15.31686613 | 0.528309034 | 0.597290121 |
| UACC0257:Vismodegib:Ldose: -1.397940009 (uM) Interaction | 6.799352691 | 15.05685414 | 0.451578572 | 0.651577002 |
| MeWo:Vorinostat:Ldose: -1.397940009 (uM) Interaction | -1.63983462 | 15.13733352 | -0.108330481 | 0.913734519 |
| SKMEL2:Vorinostat:Ldose: -1.397940009 (uM) Interaction | -1.655677141 | 15.31686613 | -0.108095032 | 0.913921281 |
| UACC0257:Vorinostat:Ldose: -1.397940009 (uM) Interaction | 19.85767906 | 15.05685414 | 1.318846478 | 0.187233988 |
| MeWo:Zoledronic.Acid:Ldose: -1.397940009 (uM) Interaction | 0.216560669 | 15.33308304 | 0.014123752 | 0.988731377 |
| MeWo:Abiraterone:Ldose: -1 (uM) Interaction | 0.875035499 | 15.13733352 | 0.057806449 | 0.953903319 |
| SKMEL2:Abiraterone:Ldose: -1 (uM) Interaction | 3.125961276 | 15.31686613 | 0.204086218 | 0.838287987 |
| MeWo:ABT.737:Ldose: -1 (uM) Interaction | -8.850883568 | 15.13733352 | -0.584705593 | 0.558751609 |
| SKMEL2:ABT.737:Ldose: -1 (uM) Interaction | -10.22534095 | 15.31686613 | -0.667587016 | 0.504404158 |
| UACC0257:ABT.737:Ldose: -1 (uM) Interaction | -6.330861627 | 15.05685414 | -0.420463768 | 0.674150745 |
| MeWo:Actinomycin.D:Ldose: -1 (uM) Interaction | -9.468736001 | 15.13733352 | -0.625522057 | 0.531634879 |
| SKMEL2:Actinomycin.D:Ldose: -1 (uM) Interaction | -36.8529351 | 15.31686613 | -2.406036247 | 0.01613475 |
| UACC0257:Actinomycin.D:Ldose: -1 (uM) Interaction | -23.43419062 | 15.05685414 | -1.556380264 | 0.119631889 |
| MeWo:Afatinib:Ldose: -1 (uM) Interaction | 5.172758989 | 15.13733352 | 0.341721941 | 0.732563379 |
| SKMEL2:Afatinib:Ldose: -1 (uM) Interaction | 4.428899883 | 15.31686613 | 0.289151831 | 0.77246788 |
| UACC0257:Afatinib:Ldose: -1 (uM) Interaction | -4.773324921 | 15.05685414 | -0.317020068 | 0.751231343 |
| MeWo:Alisertib..MLN8237.:Ldose: -1 (uM) Interaction | 5.610280127 | 15.13733352 | 0.370625389 | 0.71092009 |
| SKMEL2:Alisertib..MLN8237.:Ldose: -1 (uM) Interaction | -5.678264489 | 15.31686613 | -0.370719731 | 0.710849815 |
| MeWo:Allopurinol:Ldose: -1 (uM) Interaction | -14.8873816 | 15.13733352 | -0.983487717 | 0.325378129 |
| SKMEL2:Allopurinol:Ldose: -1 (uM) Interaction | 2.664508206 | 15.31686613 | 0.173959097 | 0.861899187 |
| UACC0257:Allopurinol:Ldose: -1 (uM) Interaction | 3.916293964 | 15.05685414 | 0.260100412 | 0.794788722 |
| MeWo:Amifostine:Ldose: -1 (uM) Interaction | -5.780384418 | 15.13733352 | -0.38186279 | 0.702566781 |
| MeWo:Aphrocallistin.analogue:Ldose: -1 (uM) Interaction | -1.094195289 | 15.13733352 | -0.072284547 | 0.942376111 |
| SKMEL2:Aphrocallistin.analogue:Ldose: -1 (uM) Interaction | 8.085421393 | 15.31686613 | 0.527877003 | 0.59758996 |
| UACC0257:Aphrocallistin.analogue:Ldose: -1 (uM) Interaction | -5.544831291 | 15.05685414 | -0.368259614 | 0.712683159 |
| MeWo:Arsenic.Trioxide:Ldose: -1 (uM) Interaction | 1.421123332 | 15.13733352 | 0.093882012 | 0.925203726 |
| SKMEL2:Arsenic.Trioxide:Ldose: -1 (uM) Interaction | 20.0010335 | 15.31686613 | 1.305817609 | 0.191628063 |
| MeWo:Axitinib:Ldose: -1 (uM) Interaction | 8.110921726 | 15.13733352 | 0.535822357 | 0.592086674 |
| SKMEL2:Axitinib:Ldose: -1 (uM) Interaction | 5.065185545 | 15.31686613 | 0.330693335 | 0.740879245 |
| UACC0257:Axitinib:Ldose: -1 (uM) Interaction | -11.07344525 | 15.05685414 | -0.735442155 | 0.462077993 |
| MeWo:Axitinib.1:Ldose: -1 (uM) Interaction | 7.493896516 | 15.13733352 | 0.49506054 | 0.620562265 |
| SKMEL2:Axitinib.1:Ldose: -1 (uM) Interaction | 12.53385368 | 15.31686613 | 0.818304056 | 0.413192336 |
| UACC0257:Axitinib.1:Ldose: -1 (uM) Interaction | 1.651838856 | 15.05685414 | 0.109706771 | 0.912642917 |
| MeWo:Azacitidine:Ldose: -1 (uM) Interaction | -3.204355638 | 15.13733352 | -0.211685607 | 0.832354252 |
| SKMEL2:Azacitidine:Ldose: -1 (uM) Interaction | 0.872768603 | 15.31686613 | 0.056980886 | 0.954560932 |
| UACC0257:Azacitidine:Ldose: -1 (uM) Interaction | 7.229231474 | 15.05685414 | 0.480128944 | 0.631140419 |
| SKMEL2:Baricitinib..LY3009104..INCB028050.:Ldose: -1 (uM) Interaction | 7.020351865 | 15.31686613 | 0.458341269 | 0.646711746 |
| UACC0257:Baricitinib..LY3009104..INCB028050.:Ldose: -1 (uM) Interaction | -14.20247788 | 15.05685414 | -0.943256655 | 0.345559861 |
| SKMEL2:Bendamustine.HCl:Ldose: -1 (uM) Interaction | 16.06328051 | 15.25363648 | 1.053078755 | 0.292316311 |
| MeWo:Bioymifi:Ldose: -1 (uM) Interaction | -4.258497528 | 15.13733352 | -0.281324153 | 0.778464398 |
| SKMEL2:Bioymifi:Ldose: -1 (uM) Interaction | -7.728334159 | 15.31686613 | -0.504563668 | 0.61387032 |
| UACC0257:Bioymifi:Ldose: -1 (uM) Interaction | -19.70718624 | 15.05685414 | -1.308851507 | 0.190598145 |
| MeWo:Bleomycin.Sulfate:Ldose: -1 (uM) Interaction | 5.910636482 | 15.13733352 | 0.39046748 | 0.696194628 |
| SKMEL2:Bleomycin.Sulfate:Ldose: -1 (uM) Interaction | 4.824821343 | 15.31686613 | 0.315000556 | 0.75276418 |
| UACC0257:Bleomycin.Sulfate:Ldose: -1 (uM) Interaction | 20.10033089 | 15.05685414 | 1.334962184 | 0.181902354 |
| MeWo:Bortezomib:Ldose: -1 (uM) Interaction | 7.539019339 | 15.13733352 | 0.498041437 | 0.618459749 |
| SKMEL2:Bortezomib:Ldose: -1 (uM) Interaction | 20.2403329 | 15.31686613 | 1.32144087 | 0.186367966 |
| UACC0257:Bortezomib:Ldose: -1 (uM) Interaction | 24.84364359 | 15.05685414 | 1.649988992 | 0.098959273 |
| MeWo:Bosutinib..SKI.606.:Ldose: -1 (uM) Interaction | 9.308830601 | 15.13733352 | 0.614958413 | 0.538588461 |
| SKMEL2:Bosutinib..SKI.606.:Ldose: -1 (uM) Interaction | 14.47011528 | 15.31686613 | 0.944717749 | 0.344813231 |
| UACC0257:Bosutinib..SKI.606.:Ldose: -1 (uM) Interaction | 10.97925082 | 15.05685414 | 0.729186238 | 0.465895408 |
| SKMEL2:Busulfan:Ldose: -1 (uM) Interaction | 8.021072423 | 15.31686613 | 0.52367582 | 0.600509242 |
| MeWo:Cabazitaxel:Ldose: -1 (uM) Interaction | 1.069182481 | 15.13733352 | 0.070632155 | 0.943691152 |
| SKMEL2:Cabazitaxel:Ldose: -1 (uM) Interaction | 4.007125865 | 15.31686613 | 0.261615257 | 0.793620497 |
| UACC0257:Cabazitaxel:Ldose: -1 (uM) Interaction | 18.41825723 | 15.05685414 | 1.22324737 | 0.221249207 |
| MeWo:Cabozantinib..XL.184.:Ldose: -1 (uM) Interaction | 0.963465987 | 15.13733352 | 0.063648329 | 0.949250819 |
| SKMEL2:Cabozantinib..XL.184.:Ldose: -1 (uM) Interaction | 8.374362518 | 15.31686613 | 0.546741249 | 0.584561983 |
| UACC0257:Cabozantinib..XL.184.:Ldose: -1 (uM) Interaction | 5.616396119 | 15.05685414 | 0.373012587 | 0.709142622 |
| MeWo:Capecitabine:Ldose: -1 (uM) Interaction | -1.114641359 | 15.33308304 | -0.072695188 | 0.94204933 |
| MeWo:Carfilzomib:Ldose: -1 (uM) Interaction | -33.10357604 | 15.13733352 | -2.186882913 | 0.028761427 |
| SKMEL2:Carfilzomib:Ldose: -1 (uM) Interaction | 57.83544275 | 15.31686613 | 3.775931856 | 0.000159824 |
| UACC0257:Carfilzomib:Ldose: -1 (uM) Interaction | 7.214569928 | 15.05685414 | 0.479155198 | 0.631832923 |
| MeWo:Carmustine:Ldose: -1 (uM) Interaction | -1.274316177 | 15.33308304 | -0.083108933 | 0.933765669 |
| MeWo:Celecoxib:Ldose: -1 (uM) Interaction | -3.636297134 | 15.33308304 | -0.237153684 | 0.812539745 |
| UACC0257:Chlorambucil:Ldose: -1 (uM) Interaction | -16.62574554 | 15.05685414 | -1.104197822 | 0.269519251 |
| MeWo:Cisplatin:Ldose: -1 (uM) Interaction | 7.569714783 | 15.13733352 | 0.500069234 | 0.617031264 |
| SKMEL2:Cisplatin:Ldose: -1 (uM) Interaction | 4.080250775 | 15.31686613 | 0.2663894 | 0.789941792 |
| MeWo:Cladribine:Ldose: -1 (uM) Interaction | 36.85982164 | 15.13733352 | 2.435027383 | 0.014898449 |
| SKMEL2:Cladribine:Ldose: -1 (uM) Interaction | 53.05272239 | 15.31686613 | 3.463679969 | 0.00053384 |
| UACC0257:Cladribine:Ldose: -1 (uM) Interaction | 57.5490578 | 15.05685414 | 3.822116975 | 0.00013267 |
| MeWo:Clofarabine:Ldose: -1 (uM) Interaction | 54.6759238 | 15.13733352 | 3.61199175 | 0.000304521 |
| SKMEL2:Clofarabine:Ldose: -1 (uM) Interaction | 51.77200189 | 15.31686613 | 3.380064921 | 0.000725929 |
| UACC0257:Clofarabine:Ldose: -1 (uM) Interaction | 48.37280406 | 15.05685414 | 3.212676673 | 0.00131691 |
| MeWo:Crizotinib:Ldose: -1 (uM) Interaction | 1.279336586 | 15.13733352 | 0.08451532 | 0.932647482 |
| SKMEL2:Crizotinib:Ldose: -1 (uM) Interaction | 4.457225056 | 15.31686613 | 0.291001111 | 0.771053177 |
| UACC0257:Crizotinib:Ldose: -1 (uM) Interaction | -14.22539752 | 15.05685414 | -0.944778862 | 0.344782023 |
| MeWo:Cytarabine.HCl...Ara.C:Ldose: -1 (uM) Interaction | 24.52301712 | 15.13733352 | 1.620035463 | 0.105238802 |
| SKMEL2:Cytarabine.HCl...Ara.C:Ldose: -1 (uM) Interaction | 13.32753714 | 15.31686613 | 0.87012167 | 0.384243284 |
| UACC0257:Cytarabine.HCl...Ara.C:Ldose: -1 (uM) Interaction | 0.128440831 | 15.05685414 | 0.00853039 | 0.993193893 |
| MeWo:Dacarbazine:Ldose: -1 (uM) Interaction | 2.878344734 | 15.13733352 | 0.190148729 | 0.849194317 |
| SKMEL2:Dacarbazine:Ldose: -1 (uM) Interaction | 10.98840293 | 15.31686613 | 0.71740543 | 0.473131486 |
| MeWo:Dacomitinib..PF299804.:Ldose: -1 (uM) Interaction | -5.471700769 | 15.13733352 | -0.361470583 | 0.717751117 |
| SKMEL2:Dacomitinib..PF299804.:Ldose: -1 (uM) Interaction | -5.898892545 | 15.31686613 | -0.385123987 | 0.700149231 |
| UACC0257:Dacomitinib..PF299804.:Ldose: -1 (uM) Interaction | 11.11711914 | 15.05685414 | 0.738342753 | 0.460313964 |
| MeWo:Dasatinib:Ldose: -1 (uM) Interaction | -4.300321436 | 15.13733352 | -0.284087117 | 0.776346258 |
| SKMEL2:Dasatinib:Ldose: -1 (uM) Interaction | -11.95798471 | 15.31686613 | -0.780707007 | 0.434983127 |
| UACC0257:Dasatinib:Ldose: -1 (uM) Interaction | 3.452618521 | 15.05685414 | 0.229305437 | 0.818633631 |
| MeWo:Daunorubicin.HCl:Ldose: -1 (uM) Interaction | 22.33349152 | 15.13733352 | 1.475391388 | 0.14012147 |
| SKMEL2:Daunorubicin.HCl:Ldose: -1 (uM) Interaction | 18.69701777 | 15.31686613 | 1.220681673 | 0.222219479 |
| UACC0257:Daunorubicin.HCl:Ldose: -1 (uM) Interaction | 19.76513275 | 15.05685414 | 1.312700021 | 0.189297562 |
| MeWo:Decitabine:Ldose: -1 (uM) Interaction | -7.677825074 | 15.13733352 | -0.507211198 | 0.612011655 |
| SKMEL2:Decitabine:Ldose: -1 (uM) Interaction | 3.95237153 | 15.31686613 | 0.258040483 | 0.796378046 |
| MeWo:Docetaxel:Ldose: -1 (uM) Interaction | 7.567451486 | 15.13733352 | 0.499919717 | 0.617136542 |
| MeWo:Doxorubicin.HCl:Ldose: -1 (uM) Interaction | 10.32822424 | 15.13733352 | 0.682301426 | 0.49505546 |
| SKMEL2:Doxorubicin.HCl:Ldose: -1 (uM) Interaction | 11.14573019 | 15.31686613 | 0.727676934 | 0.466819014 |
| UACC0257:Doxorubicin.HCl:Ldose: -1 (uM) Interaction | 20.71397677 | 15.05685414 | 1.375717436 | 0.168922951 |
| MeWo:Erlotinib.HCl:Ldose: -1 (uM) Interaction | -10.98783533 | 15.13733352 | -0.725876543 | 0.467922077 |
| SKMEL2:Erlotinib.HCl:Ldose: -1 (uM) Interaction | -1.559920324 | 15.31686613 | -0.101843309 | 0.918881965 |
| UACC0257:Erlotinib.HCl:Ldose: -1 (uM) Interaction | -12.50728684 | 15.05685414 | -0.830670652 | 0.406168605 |
| MeWo:Etoposide:Ldose: -1 (uM) Interaction | 2.240577766 | 15.13733352 | 0.148016674 | 0.882330948 |
| SKMEL2:Etoposide:Ldose: -1 (uM) Interaction | 3.84848826 | 15.31686613 | 0.251258203 | 0.801616803 |
| UACC0257:Etoposide:Ldose: -1 (uM) Interaction | -5.659424244 | 15.05685414 | -0.375870297 | 0.707016899 |
| MeWo:Everolimus:Ldose: -1 (uM) Interaction | -4.923663982 | 15.33308304 | -0.321113762 | 0.748127191 |
| MeWo:Exemestane:Ldose: -1 (uM) Interaction | 8.225532893 | 15.07335079 | 0.545700356 | 0.585277387 |
| SKMEL2:Exemestane:Ldose: -1 (uM) Interaction | 15.79490954 | 15.25363648 | 1.035484854 | 0.300453767 |
| MeWo:Floxuridine:Ldose: -1 (uM) Interaction | 30.82691119 | 15.13733352 | 2.03648226 | 0.041713744 |
| SKMEL2:Floxuridine:Ldose: -1 (uM) Interaction | 39.44968137 | 15.31686613 | 2.575571337 | 0.010013843 |
| UACC0257:Floxuridine:Ldose: -1 (uM) Interaction | 14.59213355 | 15.05685414 | 0.969135612 | 0.332488044 |
| MeWo:Fludarabine.Phosphate:Ldose: -1 (uM) Interaction | -0.306746356 | 15.13733352 | -0.020264227 | 0.983832774 |
| SKMEL2:Fludarabine.Phosphate:Ldose: -1 (uM) Interaction | -3.472678569 | 15.31686613 | -0.226722525 | 0.820641589 |
| MeWo:Fluorouracil...5.FU.:Ldose: -1 (uM) Interaction | 0.246098147 | 15.13733352 | 0.016257695 | 0.987028953 |
| SKMEL2:Fluorouracil...5.FU.:Ldose: -1 (uM) Interaction | 2.556644 | 15.31686613 | 0.166916912 | 0.867436939 |
| UACC0257:Fluorouracil...5.FU.:Ldose: -1 (uM) Interaction | -6.871951996 | 15.05685414 | -0.45640025 | 0.648106632 |
| MeWo:Flutamide..Eulexin.:Ldose: -1 (uM) Interaction | -1.052938018 | 15.13733352 | -0.069559016 | 0.944545283 |
| SKMEL2:Flutamide..Eulexin.:Ldose: -1 (uM) Interaction | 3.061677191 | 15.31686613 | 0.19988927 | 0.841569005 |
| UACC0257:Flutamide..Eulexin.:Ldose: -1 (uM) Interaction | -18.10500743 | 15.05685414 | -1.202442905 | 0.229204744 |
| MeWo:Foretinib..GSK1363089.:Ldose: -1 (uM) Interaction | -15.92462742 | 15.13733352 | -1.052010078 | 0.292806327 |
| SKMEL2:Foretinib..GSK1363089.:Ldose: -1 (uM) Interaction | 7.855298856 | 15.31686613 | 0.512852877 | 0.60805932 |
| UACC0257:Foretinib..GSK1363089.:Ldose: -1 (uM) Interaction | -2.285537861 | 15.05685414 | -0.15179385 | 0.879350906 |
| SKMEL2:Fulvestrant:Ldose: -1 (uM) Interaction | -5.826794689 | 15.31686613 | -0.380416897 | 0.703639598 |
| UACC0257:Fulvestrant:Ldose: -1 (uM) Interaction | 1.995737306 | 15.05685414 | 0.132546765 | 0.894553032 |
| MeWo:Gefitinib:Ldose: -1 (uM) Interaction | 3.996317411 | 15.33308304 | 0.260633651 | 0.794377444 |
| MeWo:Gemcitabine.HCl:Ldose: -1 (uM) Interaction | 28.07113351 | 15.13733352 | 1.854430536 | 0.063690784 |
| SKMEL2:Gemcitabine.HCl:Ldose: -1 (uM) Interaction | 47.71429892 | 15.31686613 | 3.11514761 | 0.00184086 |
| UACC0257:Gemcitabine.HCl:Ldose: -1 (uM) Interaction | -10.0256467 | 15.05685414 | -0.665852681 | 0.505512157 |
| MeWo:Ibrutinib..PCI.32765.:Ldose: -1 (uM) Interaction | -6.237829607 | 15.07335079 | -0.413831649 | 0.679001385 |
| SKMEL2:Ibrutinib..PCI.32765.:Ldose: -1 (uM) Interaction | -2.507076491 | 15.25363648 | -0.164359266 | 0.869449819 |
| MeWo:Imiquimod:Ldose: -1 (uM) Interaction | -25.78365827 | 15.07335079 | -1.710545892 | 0.087178886 |
| SKMEL2:Imiquimod:Ldose: -1 (uM) Interaction | -22.23665539 | 15.25363648 | -1.457793715 | 0.144911496 |
| MeWo:INK.128..MLN0128.:Ldose: -1 (uM) Interaction | 13.92755307 | 15.13733352 | 0.920079686 | 0.357541073 |
| SKMEL2:INK.128..MLN0128.:Ldose: -1 (uM) Interaction | 5.646574743 | 15.31686613 | 0.368650786 | 0.712391536 |
| UACC0257:INK.128..MLN0128.:Ldose: -1 (uM) Interaction | 7.833751314 | 15.05685414 | 0.52027809 | 0.60287493 |
| MeWo:Irinotecan.HCl:Ldose: -1 (uM) Interaction | 10.87094129 | 15.13733352 | 0.718154308 | 0.472669672 |
| SKMEL2:Irinotecan.HCl:Ldose: -1 (uM) Interaction | 9.039726273 | 15.31686613 | 0.59018119 | 0.555075163 |
| UACC0257:Irinotecan.HCl:Ldose: -1 (uM) Interaction | 16.73683005 | 15.05685414 | 1.111575492 | 0.266332701 |
| MeWo:Ixabepilone:Ldose: -1 (uM) Interaction | 18.37594672 | 15.13733352 | 1.213948724 | 0.224780164 |
| SKMEL2:Ixabepilone:Ldose: -1 (uM) Interaction | 2.419634033 | 15.31686613 | 0.157971873 | 0.874480393 |
| UACC0257:Ixabepilone:Ldose: -1 (uM) Interaction | 13.23270897 | 15.05685414 | 0.878849516 | 0.379492345 |
| MeWo:Lapatinib:Ldose: -1 (uM) Interaction | -19.61936417 | 15.07335079 | -1.301592755 | 0.193069086 |
| SKMEL2:Lapatinib:Ldose: -1 (uM) Interaction | -19.37467225 | 15.25363648 | -1.27016743 | 0.204038251 |
| MeWo:LDK378:Ldose: -1 (uM) Interaction | 5.837183632 | 15.13733352 | 0.385615051 | 0.699785464 |
| SKMEL2:LDK378:Ldose: -1 (uM) Interaction | 12.46910233 | 15.31686613 | 0.814076602 | 0.415609763 |
| UACC0257:LDK378:Ldose: -1 (uM) Interaction | 1.633634823 | 15.05685414 | 0.108497752 | 0.91360184 |
| MeWo:Lenalidomide:Ldose: -1 (uM) Interaction | -2.423109408 | 15.13733352 | -0.160075049 | 0.872823406 |
| SKMEL2:Lenalidomide:Ldose: -1 (uM) Interaction | -9.48525081 | 15.31686613 | -0.619268376 | 0.535745902 |
| SKMEL2:Letrozole:Ldose: -1 (uM) Interaction | 10.31774586 | 15.31686613 | 0.673619902 | 0.50055998 |
| MeWo:Linsitinib:Ldose: -1 (uM) Interaction | 2.007621149 | 15.13733352 | 0.132627133 | 0.89448947 |
| SKMEL2:Linsitinib:Ldose: -1 (uM) Interaction | -1.547610937 | 15.31686613 | -0.101039659 | 0.919519886 |
| UACC0257:Linsitinib:Ldose: -1 (uM) Interaction | -4.102336064 | 15.05685414 | -0.272456386 | 0.785273645 |
| SKMEL2:Lomustine..CCNU.:Ldose: -1 (uM) Interaction | -2.257798774 | 15.31686613 | -0.147406053 | 0.882812861 |
| MeWo:LY2157299:Ldose: -1 (uM) Interaction | -3.422719268 | 15.13733352 | -0.226111109 | 0.821117077 |
| SKMEL2:LY2157299:Ldose: -1 (uM) Interaction | -0.256162927 | 15.31686613 | -0.016724239 | 0.98665676 |
| MeWo:Mechlorethamine.HCl:Ldose: -1 (uM) Interaction | -0.651571716 | 15.13733352 | -0.043044022 | 0.965666827 |
| SKMEL2:Mechlorethamine.HCl:Ldose: -1 (uM) Interaction | -5.986248669 | 15.31686613 | -0.39082725 | 0.695928665 |
| UACC0257:Mechlorethamine.HCl:Ldose: -1 (uM) Interaction | 10.01461843 | 15.05685414 | 0.66512024 | 0.50598047 |
| MeWo:Megestrol.acetate:Ldose: -1 (uM) Interaction | 8.264873864 | 15.13733352 | 0.545992717 | 0.585076406 |
| SKMEL2:Megestrol.acetate:Ldose: -1 (uM) Interaction | 3.399984568 | 15.31686613 | 0.221976515 | 0.824334198 |
| MeWo:MEK.162..ARRY.438162.:Ldose: -1 (uM) Interaction | 25.61409642 | 15.13733352 | 1.692114161 | 0.090638182 |
| SKMEL2:MEK.162..ARRY.438162.:Ldose: -1 (uM) Interaction | 37.91027202 | 15.31686613 | 2.475067139 | 0.01332849 |
| UACC0257:MEK.162..ARRY.438162.:Ldose: -1 (uM) Interaction | 21.23467509 | 15.05685414 | 1.410299581 | 0.158465192 |
| MeWo:Melphalan:Ldose: -1 (uM) Interaction | 9.283190766 | 15.13733352 | 0.613264598 | 0.539707653 |
| SKMEL2:Melphalan:Ldose: -1 (uM) Interaction | 0.333856911 | 15.31686613 | 0.021796685 | 0.982610333 |
| UACC0257:Melphalan:Ldose: -1 (uM) Interaction | 7.598638089 | 15.05685414 | 0.50466306 | 0.613800498 |
| MeWo:Mercaptopurine:Ldose: -1 (uM) Interaction | 5.182269446 | 15.13733352 | 0.342350219 | 0.732090574 |
| SKMEL2:Mercaptopurine:Ldose: -1 (uM) Interaction | 3.265708176 | 15.31686613 | 0.213209944 | 0.831165161 |
| UACC0257:Mercaptopurine:Ldose: -1 (uM) Interaction | 22.11276235 | 15.05685414 | 1.468617691 | 0.141950622 |
| MeWo:Mitomycin.C:Ldose: -1 (uM) Interaction | 10.04741493 | 15.13733352 | 0.663750647 | 0.506856781 |
| SKMEL2:Mitomycin.C:Ldose: -1 (uM) Interaction | -3.186729214 | 15.31686613 | -0.208053605 | 0.835189013 |
| UACC0257:Mitomycin.C:Ldose: -1 (uM) Interaction | -11.69487965 | 15.05685414 | -0.77671468 | 0.437335364 |
| MeWo:Mitotane..o.p..DDD..Lysodren.:Ldose: -1 (uM) Interaction | 3.409637312 | 15.13733352 | 0.225246891 | 0.821789277 |
| SKMEL2:Mitotane..o.p..DDD..Lysodren.:Ldose: -1 (uM) Interaction | 5.600842306 | 15.31686613 | 0.36566503 | 0.714618513 |
| MeWo:Mitoxantrone:Ldose: -1 (uM) Interaction | 6.684318639 | 15.13733352 | 0.441578342 | 0.658798642 |
| SKMEL2:Mitoxantrone:Ldose: -1 (uM) Interaction | 16.61525899 | 15.31686613 | 1.084768832 | 0.278035782 |
| UACC0257:Mitoxantrone:Ldose: -1 (uM) Interaction | 4.709110224 | 15.05685414 | 0.312755253 | 0.754469539 |
| UACC0257:MLN.2480:Ldose: -1 (uM) Interaction | -18.35855165 | 15.05685414 | -1.219282028 | 0.222750066 |
| MeWo:MLN4924:Ldose: -1 (uM) Interaction | -20.50556809 | 15.13733352 | -1.354635415 | 0.17554747 |
| SKMEL2:MLN4924:Ldose: -1 (uM) Interaction | -1.789617785 | 15.31686613 | -0.116839683 | 0.906988149 |
| UACC0257:MLN4924:Ldose: -1 (uM) Interaction | -22.67946629 | 15.05685414 | -1.506255296 | 0.132015816 |
| MeWo:MLN9708..MLN2238.:Ldose: -1 (uM) Interaction | -47.77887491 | 15.13733352 | -3.156360057 | 0.001599641 |
| SKMEL2:MLN9708..MLN2238.:Ldose: -1 (uM) Interaction | -45.41235721 | 15.31686613 | -2.964859576 | 0.003031416 |
| UACC0257:MLN9708..MLN2238.:Ldose: -1 (uM) Interaction | -32.36565737 | 15.05685414 | -2.149563054 | 0.03160052 |
| MeWo:Navitoclax..ABT.263..5uM:Ldose: -1 (uM) Interaction | -4.121965511 | 15.13733352 | -0.272304597 | 0.785390343 |
| SKMEL2:Navitoclax..ABT.263..5uM:Ldose: -1 (uM) Interaction | -5.979461824 | 15.31686613 | -0.390384154 | 0.696256233 |
| UACC0257:Navitoclax..ABT.263..5uM:Ldose: -1 (uM) Interaction | 10.95766055 | 15.05685414 | 0.727752321 | 0.466772858 |
| MeWo:Nelarabine:Ldose: -1 (uM) Interaction | -7.618008858 | 15.13733352 | -0.503259629 | 0.614786718 |
| SKMEL2:Nelarabine:Ldose: -1 (uM) Interaction | 0.947935611 | 15.31686613 | 0.061888353 | 0.950652298 |
| UACC0257:Nelarabine:Ldose: -1 (uM) Interaction | 2.081400758 | 15.05685414 | 0.138236098 | 0.890055076 |
| MeWo:OSI.027:Ldose: -1 (uM) Interaction | -11.16120043 | 15.13733352 | -0.73732936 | 0.460929841 |
| SKMEL2:OSI.027:Ldose: -1 (uM) Interaction | -11.18859469 | 15.31686613 | -0.730475451 | 0.465107289 |
| UACC0257:OSI.027:Ldose: -1 (uM) Interaction | -9.880983511 | 15.05685414 | -0.656244885 | 0.511673346 |
| MeWo:Oxaliplatin:Ldose: -1 (uM) Interaction | 6.24936441 | 15.07335079 | 0.414596893 | 0.678441012 |
| SKMEL2:Oxaliplatin:Ldose: -1 (uM) Interaction | 15.49672681 | 15.25363648 | 1.015936549 | 0.309670627 |
| MeWo:Paclitaxel:Ldose: -1 (uM) Interaction | 11.63434203 | 15.13733352 | 0.768585961 | 0.442147283 |
| SKMEL2:Paclitaxel:Ldose: -1 (uM) Interaction | 7.152556923 | 15.31686613 | 0.466972608 | 0.640524039 |
| MeWo:Palbociclib..PD.0332991..Isethionate:Ldose: -1 (uM) Interaction | 0.780792066 | 15.13733352 | 0.051580555 | 0.958863376 |
| SKMEL2:Palbociclib..PD.0332991..Isethionate:Ldose: -1 (uM) Interaction | 1.15400889 | 15.31686613 | 0.075342363 | 0.939942992 |
| UACC0257:Palbociclib..PD.0332991..Isethionate:Ldose: -1 (uM) Interaction | 2.448729784 | 15.05685414 | 0.162632231 | 0.870809482 |
| MeWo:Pazopanib.HCl:Ldose: -1 (uM) Interaction | -0.199151218 | 15.13733352 | -0.013156294 | 0.989503216 |
| SKMEL2:Pazopanib.HCl:Ldose: -1 (uM) Interaction | 2.572900705 | 15.31686613 | 0.167978272 | 0.866601896 |
| UACC0257:Pazopanib.HCl:Ldose: -1 (uM) Interaction | 11.72569826 | 15.05685414 | 0.778761496 | 0.436128488 |
| MeWo:PD325901:Ldose: -1 (uM) Interaction | -9.988532724 | 15.13733352 | -0.659860781 | 0.50934999 |
| SKMEL2:PD325901:Ldose: -1 (uM) Interaction | -15.82603896 | 15.31686613 | -1.033242624 | 0.301501577 |
| UACC0257:PD325901:Ldose: -1 (uM) Interaction | 2.879256019 | 15.05685414 | 0.191225603 | 0.848350586 |
| SKMEL2:Pemetrexed:Ldose: -1 (uM) Interaction | 10.14503895 | 15.31686613 | 0.662344298 | 0.507757439 |
| MeWo:Plicamycin:Ldose: -1 (uM) Interaction | 28.35151834 | 15.13733352 | 1.872953271 | 0.061087912 |
| SKMEL2:Plicamycin:Ldose: -1 (uM) Interaction | 16.97179818 | 15.31686613 | 1.108046388 | 0.267853736 |
| UACC0257:Plicamycin:Ldose: -1 (uM) Interaction | 39.28942183 | 15.05685414 | 2.609404426 | 0.009076055 |
| MeWo:Pralatrexate:Ldose: -1 (uM) Interaction | 4.725023987 | 15.07335079 | 0.313468721 | 0.753927514 |
| SKMEL2:Pralatrexate:Ldose: -1 (uM) Interaction | 5.024790164 | 15.25363648 | 0.329415885 | 0.741844457 |
| MeWo:Quinacrine.HCl:Ldose: -1 (uM) Interaction | -4.393017221 | 15.13733352 | -0.29021077 | 0.771657696 |
| SKMEL2:Quinacrine.HCl:Ldose: -1 (uM) Interaction | -6.349806599 | 15.31686613 | -0.414563041 | 0.678465797 |
| UACC0257:Quinacrine.HCl:Ldose: -1 (uM) Interaction | -0.316223831 | 15.05685414 | -0.021001985 | 0.98324426 |
| MeWo:Quizartinib:Ldose: -1 (uM) Interaction | 0.382261516 | 15.13733352 | 0.025252896 | 0.979853471 |
| SKMEL2:Quizartinib:Ldose: -1 (uM) Interaction | -6.942718844 | 15.31686613 | -0.453272803 | 0.650356725 |
| UACC0257:Quizartinib:Ldose: -1 (uM) Interaction | -19.31308979 | 15.05685414 | -1.282677618 | 0.199618379 |
| MeWo:Raloxifene:Ldose: -1 (uM) Interaction | 15.87690621 | 15.07335079 | 1.053309674 | 0.292210501 |
| SKMEL2:Raloxifene:Ldose: -1 (uM) Interaction | 22.36433952 | 15.25363648 | 1.466164449 | 0.142617594 |
| MeWo:Romidepsin:Ldose: -1 (uM) Interaction | 9.220925242 | 15.13733352 | 0.609151224 | 0.542430414 |
| SKMEL2:Romidepsin:Ldose: -1 (uM) Interaction | 15.05971865 | 15.31686613 | 0.983211482 | 0.325514034 |
| UACC0257:Romidepsin:Ldose: -1 (uM) Interaction | 13.57484597 | 15.05685414 | 0.901572522 | 0.367293732 |
| MeWo:Sabutoclax..BI.97C1.:Ldose: -1 (uM) Interaction | 3.094126916 | 15.13733352 | 0.204403696 | 0.838039908 |
| SKMEL2:Sabutoclax..BI.97C1.:Ldose: -1 (uM) Interaction | 2.994675991 | 15.31686613 | 0.195514929 | 0.844991633 |
| UACC0257:Sabutoclax..BI.97C1.:Ldose: -1 (uM) Interaction | 15.52471801 | 15.05685414 | 1.031073149 | 0.302517703 |
| MeWo:Sirolimus..Rapamycin.:Ldose: -1 (uM) Interaction | -3.590870536 | 15.13733352 | -0.23721949 | 0.812488697 |
| SKMEL2:Sirolimus..Rapamycin.:Ldose: -1 (uM) Interaction | -0.480072039 | 15.31686613 | -0.031342706 | 0.974996513 |
| MeWo:Sorafenib:Ldose: -1 (uM) Interaction | 3.848412084 | 15.13733352 | 0.254233157 | 0.799317781 |
| UACC0257:Sorafenib:Ldose: -1 (uM) Interaction | 11.42410685 | 15.05685414 | 0.758731322 | 0.448021316 |
| MeWo:Streptozocin:Ldose: -1 (uM) Interaction | 0.672058216 | 15.13733352 | 0.044397398 | 0.964588034 |
| SKMEL2:Streptozocin:Ldose: -1 (uM) Interaction | 5.14927342 | 15.31686613 | 0.336183223 | 0.736735867 |
| UACC0257:Streptozocin:Ldose: -1 (uM) Interaction | 6.876856089 | 15.05685414 | 0.456725955 | 0.647872482 |
| MeWo:Sunitinib:Ldose: -1 (uM) Interaction | -2.939979268 | 15.13733352 | -0.19422042 | 0.846005064 |
| UACC0257:Sunitinib:Ldose: -1 (uM) Interaction | -8.486362894 | 15.05685414 | -0.563621246 | 0.573017593 |
| MeWo:Tamoxifen.Citrate:Ldose: -1 (uM) Interaction | 0.510966249 | 15.13733352 | 0.033755367 | 0.97307253 |
| SKMEL2:Tamoxifen.Citrate:Ldose: -1 (uM) Interaction | 8.927695563 | 15.31686613 | 0.582866984 | 0.559988743 |
| UACC0257:Tamoxifen.Citrate:Ldose: -1 (uM) Interaction | 3.559619839 | 15.05685414 | 0.236411923 | 0.813115217 |
| MeWo:Temozolomide:Ldose: -1 (uM) Interaction | 8.937640213 | 15.33308304 | 0.582899094 | 0.559967126 |
| SKMEL2:Temsirolimus..CCI.779..Torisel.:Ldose: -1 (uM) Interaction | 2.488746018 | 15.31686613 | 0.162484022 | 0.870926182 |
| MeWo:Teniposide:Ldose: -1 (uM) Interaction | 2.915837036 | 15.13733352 | 0.192625539 | 0.847253996 |
| SKMEL2:Teniposide:Ldose: -1 (uM) Interaction | 13.55720717 | 15.31686613 | 0.885116254 | 0.376103479 |
| UACC0257:Teniposide:Ldose: -1 (uM) Interaction | 20.38595714 | 15.05685414 | 1.353932034 | 0.175771786 |
| MeWo:Thioguanine:Ldose: -1 (uM) Interaction | 6.659322907 | 15.13733352 | 0.439927078 | 0.659994188 |
| SKMEL2:Thioguanine:Ldose: -1 (uM) Interaction | 1.695249013 | 15.31686613 | 0.110678581 | 0.911872227 |
| UACC0257:Thioguanine:Ldose: -1 (uM) Interaction | 8.693025473 | 15.05685414 | 0.577346728 | 0.563711083 |
| MeWo:Thiotepa:Ldose: -1 (uM) Interaction | -8.873053833 | 15.13733352 | -0.586170201 | 0.557767078 |
| SKMEL2:Thiotepa:Ldose: -1 (uM) Interaction | -5.044729167 | 15.31686613 | -0.329357789 | 0.741888363 |
| UACC0257:Thiotepa:Ldose: -1 (uM) Interaction | 5.791169208 | 15.05685414 | 0.384620131 | 0.700522546 |
| MeWo:Topotecan.HCl:Ldose: -1 (uM) Interaction | 36.36918366 | 15.13733352 | 2.402614939 | 0.01628644 |
| SKMEL2:Topotecan.HCl:Ldose: -1 (uM) Interaction | 19.87659861 | 15.31686613 | 1.297693565 | 0.194406081 |
| UACC0257:Topotecan.HCl:Ldose: -1 (uM) Interaction | 18.61070435 | 15.05685414 | 1.236028734 | 0.216460905 |
| MeWo:Trametinib..GSK1120212.:Ldose: -1 (uM) Interaction | -20.61969033 | 15.13733352 | -1.36217454 | 0.173156569 |
| UACC0257:Trametinib..GSK1120212.:Ldose: -1 (uM) Interaction | 9.737017583 | 15.05685414 | 0.646683397 | 0.517843528 |
| MeWo:Triethylenemelamine:Ldose: -1 (uM) Interaction | 3.235061432 | 15.13733352 | 0.213714088 | 0.830771979 |
| SKMEL2:Triethylenemelamine:Ldose: -1 (uM) Interaction | 1.457818491 | 15.31686613 | 0.095177335 | 0.924174828 |
| UACC0257:Triethylenemelamine:Ldose: -1 (uM) Interaction | 10.32848466 | 15.05685414 | 0.685965645 | 0.492741906 |
| MeWo:Uracil.mustard:Ldose: -1 (uM) Interaction | 8.652327149 | 15.13733352 | 0.571588592 | 0.567606488 |
| SKMEL2:Uracil.mustard:Ldose: -1 (uM) Interaction | 4.232013781 | 15.31686613 | 0.276297628 | 0.782322038 |
| MeWo:Valrubicin:Ldose: -1 (uM) Interaction | 5.894782182 | 15.13733352 | 0.389420116 | 0.696969113 |
| SKMEL2:Valrubicin:Ldose: -1 (uM) Interaction | -1.849821352 | 15.31686613 | -0.120770224 | 0.903874124 |
| UACC0257:Valrubicin:Ldose: -1 (uM) Interaction | 9.608951286 | 15.05685414 | 0.638177882 | 0.523364448 |
| MeWo:Vandetanib:Ldose: -1 (uM) Interaction | -7.26849304 | 15.07335079 | -0.482208179 | 0.629662801 |
| SKMEL2:Vandetanib:Ldose: -1 (uM) Interaction | -6.08199135 | 15.25363648 | -0.398724026 | 0.690100381 |
| SKMEL2:Vemurafenib:Ldose: -1 (uM) Interaction | 46.42156993 | 15.31686613 | 3.030748558 | 0.00244228 |
| UACC0257:Vemurafenib:Ldose: -1 (uM) Interaction | 29.51713823 | 15.05685414 | 1.960378839 | 0.049963961 |
| SKMEL2:Vinblastine.Sulfate:Ldose: -1 (uM) Interaction | 1.616524426 | 15.31686613 | 0.105538849 | 0.915949194 |
| UACC0257:Vinblastine.Sulfate:Ldose: -1 (uM) Interaction | 17.04675821 | 15.05685414 | 1.132159351 | 0.257579622 |
| MeWo:Vincristine.Sulfate:Ldose: -1 (uM) Interaction | 28.38985651 | 15.13733352 | 1.875485961 | 0.060738957 |
| SKMEL2:Vincristine.Sulfate:Ldose: -1 (uM) Interaction | 38.05983185 | 15.31686613 | 2.484831527 | 0.012968554 |
| UACC0257:Vincristine.Sulfate:Ldose: -1 (uM) Interaction | 53.42488458 | 15.05685414 | 3.548210276 | 0.000388655 |
| MeWo:Vinorelbine.Tartrate:Ldose: -1 (uM) Interaction | -4.636192303 | 15.13733352 | -0.306275362 | 0.759397857 |
| SKMEL2:Vinorelbine.Tartrate:Ldose: -1 (uM) Interaction | -9.694915537 | 15.31686613 | -0.632956863 | 0.526768305 |
| UACC0257:Vinorelbine.Tartrate:Ldose: -1 (uM) Interaction | 1.063839939 | 15.05685414 | 0.070654861 | 0.94367308 |
| MeWo:Vismodegib:Ldose: -1 (uM) Interaction | 3.953708332 | 15.13733352 | 0.26118922 | 0.793949003 |
| SKMEL2:Vismodegib:Ldose: -1 (uM) Interaction | 7.779436963 | 15.31686613 | 0.507900043 | 0.611528469 |
| UACC0257:Vismodegib:Ldose: -1 (uM) Interaction | 9.056827366 | 15.05685414 | 0.601508607 | 0.547507396 |
| MeWo:Vorinostat:Ldose: -1 (uM) Interaction | 1.331725682 | 15.13733352 | 0.08797624 | 0.929896351 |
| SKMEL2:Vorinostat:Ldose: -1 (uM) Interaction | 5.558879166 | 15.31686613 | 0.362925361 | 0.716664082 |
| UACC0257:Vorinostat:Ldose: -1 (uM) Interaction | 26.66619794 | 15.05685414 | 1.771033823 | 0.076568737 |
| MeWo:Zoledronic.Acid:Ldose: -1 (uM) Interaction | 2.091186575 | 15.33308304 | 0.136383959 | 0.891518985 |
| MeWo:Abiraterone:Ldose: -0.698970004 (uM) Interaction | 1.154351608 | 15.13733352 | 0.076258583 | 0.93921406 |
| SKMEL2:Abiraterone:Ldose: -0.698970004 (uM) Interaction | -1.096701729 | 15.31686613 | -0.071600921 | 0.942920149 |
| MeWo:ABT.737:Ldose: -0.698970004 (uM) Interaction | -4.35352449 | 15.13733352 | -0.287601808 | 0.773654234 |
| SKMEL2:ABT.737:Ldose: -0.698970004 (uM) Interaction | -0.461949994 | 15.31686613 | -0.030159563 | 0.975940068 |
| UACC0257:ABT.737:Ldose: -0.698970004 (uM) Interaction | 4.45360395 | 15.05685414 | 0.29578582 | 0.767396407 |
| MeWo:Actinomycin.D:Ldose: -0.698970004 (uM) Interaction | 6.856877143 | 15.13733352 | 0.452977873 | 0.650569083 |
| SKMEL2:Actinomycin.D:Ldose: -0.698970004 (uM) Interaction | -43.98476139 | 15.31686613 | -2.871655404 | 0.004087127 |
| UACC0257:Actinomycin.D:Ldose: -0.698970004 (uM) Interaction | -18.04148909 | 15.05685414 | -1.198224339 | 0.230842429 |
| MeWo:Afatinib:Ldose: -0.698970004 (uM) Interaction | -0.637841075 | 15.13733352 | -0.042136951 | 0.966389901 |
| SKMEL2:Afatinib:Ldose: -0.698970004 (uM) Interaction | 1.557047396 | 15.31686613 | 0.101655742 | 0.919030847 |
| UACC0257:Afatinib:Ldose: -0.698970004 (uM) Interaction | -1.905266368 | 15.05685414 | -0.126538143 | 0.899307099 |
| MeWo:Alisertib..MLN8237.:Ldose: -0.698970004 (uM) Interaction | 9.255483446 | 15.13733352 | 0.611434202 | 0.5409184 |
| SKMEL2:Alisertib..MLN8237.:Ldose: -0.698970004 (uM) Interaction | 1.004325911 | 15.31686613 | 0.065569935 | 0.947720814 |
| MeWo:Allopurinol:Ldose: -0.698970004 (uM) Interaction | -3.536779132 | 15.13733352 | -0.233646113 | 0.815261871 |
| SKMEL2:Allopurinol:Ldose: -0.698970004 (uM) Interaction | -8.633295842 | 15.31686613 | -0.56364636 | 0.573000498 |
| UACC0257:Allopurinol:Ldose: -0.698970004 (uM) Interaction | 13.11081374 | 15.05685414 | 0.870753852 | 0.383897943 |
| MeWo:Amifostine:Ldose: -0.698970004 (uM) Interaction | -8.38147494 | 15.13733352 | -0.553695598 | 0.579792741 |
| MeWo:Aphrocallistin.analogue:Ldose: -0.698970004 (uM) Interaction | -20.33834422 | 15.13733352 | -1.3435883 | 0.179095229 |
| SKMEL2:Aphrocallistin.analogue:Ldose: -0.698970004 (uM) Interaction | -3.861844752 | 15.31686613 | -0.252130215 | 0.80094274 |
| UACC0257:Aphrocallistin.analogue:Ldose: -0.698970004 (uM) Interaction | 16.40939414 | 15.05685414 | 1.089828857 | 0.275800318 |
| MeWo:Arsenic.Trioxide:Ldose: -0.698970004 (uM) Interaction | 3.834336341 | 15.13733352 | 0.253303287 | 0.800036192 |
| SKMEL2:Arsenic.Trioxide:Ldose: -0.698970004 (uM) Interaction | -0.105928525 | 15.31686613 | -0.006915809 | 0.994482088 |
| MeWo:Axitinib:Ldose: -0.698970004 (uM) Interaction | 1.044853385 | 15.13733352 | 0.06902493 | 0.944970395 |
| SKMEL2:Axitinib:Ldose: -0.698970004 (uM) Interaction | -6.89924729 | 15.31686613 | -0.450434654 | 0.652401442 |
| UACC0257:Axitinib:Ldose: -0.698970004 (uM) Interaction | -1.309421953 | 15.05685414 | -0.086965175 | 0.930699975 |
| MeWo:Axitinib.1:Ldose: -0.698970004 (uM) Interaction | 2.479687125 | 15.13733352 | 0.163812677 | 0.869880097 |
| SKMEL2:Axitinib.1:Ldose: -0.698970004 (uM) Interaction | 5.765752248 | 15.31686613 | 0.376431589 | 0.706599648 |
| UACC0257:Axitinib.1:Ldose: -0.698970004 (uM) Interaction | 1.817061554 | 15.05685414 | 0.120680026 | 0.903945567 |
| MeWo:Azacitidine:Ldose: -0.698970004 (uM) Interaction | -8.175258932 | 15.13733352 | -0.540072591 | 0.589152366 |
| SKMEL2:Azacitidine:Ldose: -0.698970004 (uM) Interaction | -21.19517587 | 15.31686613 | -1.383780187 | 0.166439624 |
| UACC0257:Azacitidine:Ldose: -0.698970004 (uM) Interaction | -11.33143385 | 15.05685414 | -0.752576451 | 0.451712418 |
| SKMEL2:Baricitinib..LY3009104..INCB028050.:Ldose: -0.698970004 (uM) Interaction | 16.65094277 | 15.31686613 | 1.087098537 | 0.277005016 |
| UACC0257:Baricitinib..LY3009104..INCB028050.:Ldose: -0.698970004 (uM) Interaction | 6.355083987 | 15.05685414 | 0.422072495 | 0.672976173 |
| SKMEL2:Bendamustine.HCl:Ldose: -0.698970004 (uM) Interaction | 4.996038564 | 15.25363648 | 0.327530984 | 0.743269387 |
| MeWo:Bioymifi:Ldose: -0.698970004 (uM) Interaction | -3.515408997 | 15.13733352 | -0.232234362 | 0.816358121 |
| SKMEL2:Bioymifi:Ldose: -0.698970004 (uM) Interaction | 0.242584005 | 15.31686613 | 0.015837705 | 0.98736401 |
| UACC0257:Bioymifi:Ldose: -0.698970004 (uM) Interaction | 3.483244682 | 15.05685414 | 0.231339472 | 0.817053207 |
| MeWo:Bleomycin.Sulfate:Ldose: -0.698970004 (uM) Interaction | 22.27710132 | 15.13733352 | 1.471666148 | 0.141125167 |
| SKMEL2:Bleomycin.Sulfate:Ldose: -0.698970004 (uM) Interaction | 12.52624821 | 15.31686613 | 0.817807514 | 0.413475846 |
| UACC0257:Bleomycin.Sulfate:Ldose: -0.698970004 (uM) Interaction | 26.21288971 | 15.05685414 | 1.740927386 | 0.081710102 |
| MeWo:Bortezomib:Ldose: -0.698970004 (uM) Interaction | 7.723524222 | 15.13733352 | 0.510230168 | 0.60989527 |
| SKMEL2:Bortezomib:Ldose: -0.698970004 (uM) Interaction | 17.06736939 | 15.31686613 | 1.114285994 | 0.265168525 |
| UACC0257:Bortezomib:Ldose: -0.698970004 (uM) Interaction | 15.499299 | 15.05685414 | 1.029384947 | 0.303309986 |
| MeWo:Bosutinib..SKI.606.:Ldose: -0.698970004 (uM) Interaction | 7.769816691 | 15.13733352 | 0.513288333 | 0.60775473 |
| SKMEL2:Bosutinib..SKI.606.:Ldose: -0.698970004 (uM) Interaction | 11.10466158 | 15.31686613 | 0.724995667 | 0.468462297 |
| UACC0257:Bosutinib..SKI.606.:Ldose: -0.698970004 (uM) Interaction | 1.882032987 | 15.05685414 | 0.1249951 | 0.900528557 |
| SKMEL2:Busulfan:Ldose: -0.698970004 (uM) Interaction | 3.59388988 | 15.31686613 | 0.23463611 | 0.814493335 |
| MeWo:Cabazitaxel:Ldose: -0.698970004 (uM) Interaction | 6.06114324 | 15.13733352 | 0.400410233 | 0.688858225 |
| SKMEL2:Cabazitaxel:Ldose: -0.698970004 (uM) Interaction | 3.465512386 | 15.31686613 | 0.226254663 | 0.821005432 |
| UACC0257:Cabazitaxel:Ldose: -0.698970004 (uM) Interaction | -6.638268808 | 15.05685414 | -0.440880196 | 0.659304006 |
| MeWo:Cabozantinib..XL.184.:Ldose: -0.698970004 (uM) Interaction | -0.144167695 | 15.13733352 | -0.009523982 | 0.992401162 |
| SKMEL2:Cabozantinib..XL.184.:Ldose: -0.698970004 (uM) Interaction | 13.82597118 | 15.31686613 | 0.902663186 | 0.366714432 |
| UACC0257:Cabozantinib..XL.184.:Ldose: -0.698970004 (uM) Interaction | -7.453050916 | 15.05685414 | -0.494993898 | 0.620609306 |
| MeWo:Capecitabine:Ldose: -0.698970004 (uM) Interaction | -1.189292425 | 15.33308304 | -0.077563816 | 0.938175723 |
| MeWo:Carfilzomib:Ldose: -0.698970004 (uM) Interaction | -34.51048102 | 15.13733352 | -2.279825635 | 0.022627433 |
| SKMEL2:Carfilzomib:Ldose: -0.698970004 (uM) Interaction | 44.72429373 | 15.31686613 | 2.919937627 | 0.003504519 |
| UACC0257:Carfilzomib:Ldose: -0.698970004 (uM) Interaction | -42.11068131 | 15.05685414 | -2.796778193 | 0.005165916 |
| MeWo:Carmustine:Ldose: -0.698970004 (uM) Interaction | 0.89350726 | 15.33308304 | 0.058273164 | 0.953531566 |
| MeWo:Celecoxib:Ldose: -0.698970004 (uM) Interaction | 4.597311545 | 15.33308304 | 0.29982956 | 0.764309961 |
| UACC0257:Chlorambucil:Ldose: -0.698970004 (uM) Interaction | 5.72448354 | 15.05685414 | 0.380191206 | 0.703807108 |
| MeWo:Cisplatin:Ldose: -0.698970004 (uM) Interaction | -1.646918059 | 15.13733352 | -0.108798426 | 0.913363351 |
| SKMEL2:Cisplatin:Ldose: -0.698970004 (uM) Interaction | -0.196996975 | 15.31686613 | -0.012861441 | 0.989738453 |
| MeWo:Cladribine:Ldose: -0.698970004 (uM) Interaction | 58.51279656 | 15.13733352 | 3.865462597 | 0.000111193 |
| SKMEL2:Cladribine:Ldose: -0.698970004 (uM) Interaction | 65.67748049 | 15.31686613 | 4.287918948 | 1.81E-05 |
| UACC0257:Cladribine:Ldose: -0.698970004 (uM) Interaction | 62.86197551 | 15.05685414 | 4.174974064 | 2.99E-05 |
| MeWo:Clofarabine:Ldose: -0.698970004 (uM) Interaction | 56.37884312 | 15.13733352 | 3.724489722 | 0.000196193 |
| SKMEL2:Clofarabine:Ldose: -0.698970004 (uM) Interaction | 47.75898941 | 15.31686613 | 3.118065341 | 0.001822741 |
| UACC0257:Clofarabine:Ldose: -0.698970004 (uM) Interaction | 37.44494024 | 15.05685414 | 2.4869033 | 0.012893299 |
| MeWo:Crizotinib:Ldose: -0.698970004 (uM) Interaction | -0.316705832 | 15.13733352 | -0.020922168 | 0.98330793 |
| SKMEL2:Crizotinib:Ldose: -0.698970004 (uM) Interaction | 6.141371225 | 15.31686613 | 0.400954815 | 0.688457234 |
| UACC0257:Crizotinib:Ldose: -0.698970004 (uM) Interaction | -18.09214675 | 15.05685414 | -1.201588764 | 0.22953566 |
| MeWo:Cytarabine.HCl...Ara.C:Ldose: -0.698970004 (uM) Interaction | 41.46786861 | 15.13733352 | 2.739443413 | 0.006159222 |
| SKMEL2:Cytarabine.HCl...Ara.C:Ldose: -0.698970004 (uM) Interaction | 63.43317138 | 15.31686613 | 4.141393602 | 3.46E-05 |
| UACC0257:Cytarabine.HCl...Ara.C:Ldose: -0.698970004 (uM) Interaction | 29.12824748 | 15.05685414 | 1.934550684 | 0.053058092 |
| MeWo:Dacarbazine:Ldose: -0.698970004 (uM) Interaction | 4.397282353 | 15.13733352 | 0.290492533 | 0.771442164 |
| SKMEL2:Dacarbazine:Ldose: -0.698970004 (uM) Interaction | -11.21725007 | 15.31686613 | -0.732346289 | 0.463964931 |
| MeWo:Dacomitinib..PF299804.:Ldose: -0.698970004 (uM) Interaction | -3.13842516 | 15.13733352 | -0.207330119 | 0.835753947 |
| SKMEL2:Dacomitinib..PF299804.:Ldose: -0.698970004 (uM) Interaction | 4.31159377 | 15.31686613 | 0.281493207 | 0.77833475 |
| UACC0257:Dacomitinib..PF299804.:Ldose: -0.698970004 (uM) Interaction | 4.840451019 | 15.05685414 | 0.321478243 | 0.747851011 |
| MeWo:Dasatinib:Ldose: -0.698970004 (uM) Interaction | -10.12446717 | 15.13733352 | -0.66884086 | 0.503603924 |
| SKMEL2:Dasatinib:Ldose: -0.698970004 (uM) Interaction | -15.68769493 | 15.31686613 | -1.024210488 | 0.305746978 |
| UACC0257:Dasatinib:Ldose: -0.698970004 (uM) Interaction | -3.210549271 | 15.05685414 | -0.213228424 | 0.831150749 |
| MeWo:Daunorubicin.HCl:Ldose: -0.698970004 (uM) Interaction | 36.39669644 | 15.13733352 | 2.404432483 | 0.016205701 |
| SKMEL2:Daunorubicin.HCl:Ldose: -0.698970004 (uM) Interaction | 40.6254924 | 15.31686613 | 2.652337107 | 0.007999328 |
| UACC0257:Daunorubicin.HCl:Ldose: -0.698970004 (uM) Interaction | 17.91570584 | 15.05685414 | 1.189870452 | 0.234109983 |
| MeWo:Decitabine:Ldose: -0.698970004 (uM) Interaction | -1.705109985 | 15.13733352 | -0.112642691 | 0.910314852 |
| SKMEL2:Decitabine:Ldose: -0.698970004 (uM) Interaction | -11.9231645 | 15.31686613 | -0.778433682 | 0.43632165 |
| MeWo:Docetaxel:Ldose: -0.698970004 (uM) Interaction | 12.64179452 | 15.13733352 | 0.835140119 | 0.403647769 |
| MeWo:Doxorubicin.HCl:Ldose: -0.698970004 (uM) Interaction | 29.63104288 | 15.13733352 | 1.957481008 | 0.0503034 |
| SKMEL2:Doxorubicin.HCl:Ldose: -0.698970004 (uM) Interaction | 10.27217977 | 15.31686613 | 0.670645005 | 0.502453651 |
| UACC0257:Doxorubicin.HCl:Ldose: -0.698970004 (uM) Interaction | 19.75976211 | 15.05685414 | 1.312343331 | 0.189417828 |
| MeWo:Erlotinib.HCl:Ldose: -0.698970004 (uM) Interaction | -19.62115238 | 15.13733352 | -1.296209292 | 0.194916806 |
| SKMEL2:Erlotinib.HCl:Ldose: -0.698970004 (uM) Interaction | -18.85416508 | 15.31686613 | -1.230941429 | 0.218357756 |
| UACC0257:Erlotinib.HCl:Ldose: -0.698970004 (uM) Interaction | -16.2808357 | 15.05685414 | -1.081290656 | 0.279579537 |
| MeWo:Etoposide:Ldose: -0.698970004 (uM) Interaction | 10.33220177 | 15.13733352 | 0.682564188 | 0.494889361 |
| SKMEL2:Etoposide:Ldose: -0.698970004 (uM) Interaction | 5.080520111 | 15.31686613 | 0.331694491 | 0.740123079 |
| UACC0257:Etoposide:Ldose: -0.698970004 (uM) Interaction | 17.79398876 | 15.05685414 | 1.18178662 | 0.237302982 |
| MeWo:Everolimus:Ldose: -0.698970004 (uM) Interaction | -5.304864237 | 15.33308304 | -0.345975054 | 0.729364727 |
| MeWo:Exemestane:Ldose: -0.698970004 (uM) Interaction | 0.043254404 | 15.07335079 | 0.002869594 | 0.997710424 |
| SKMEL2:Exemestane:Ldose: -0.698970004 (uM) Interaction | -3.379131864 | 15.25363648 | -0.221529592 | 0.824682127 |
| MeWo:Floxuridine:Ldose: -0.698970004 (uM) Interaction | 29.1746466 | 15.13733352 | 1.927330633 | 0.053951128 |
| SKMEL2:Floxuridine:Ldose: -0.698970004 (uM) Interaction | 40.93172671 | 15.31686613 | 2.672330382 | 0.007538122 |
| UACC0257:Floxuridine:Ldose: -0.698970004 (uM) Interaction | 15.92520378 | 15.05685414 | 1.057671386 | 0.290216744 |
| MeWo:Fludarabine.Phosphate:Ldose: -0.698970004 (uM) Interaction | 4.778635136 | 15.13733352 | 0.315685397 | 0.752244267 |
| SKMEL2:Fludarabine.Phosphate:Ldose: -0.698970004 (uM) Interaction | 5.246907732 | 15.31686613 | 0.342557524 | 0.731934591 |
| MeWo:Fluorouracil...5.FU.:Ldose: -0.698970004 (uM) Interaction | 3.006914581 | 15.13733352 | 0.198642289 | 0.842544382 |
| SKMEL2:Fluorouracil...5.FU.:Ldose: -0.698970004 (uM) Interaction | 0.41881921 | 15.31686613 | 0.027343662 | 0.978185877 |
| UACC0257:Fluorouracil...5.FU.:Ldose: -0.698970004 (uM) Interaction | 1.94322826 | 15.05685414 | 0.12905938 | 0.897311827 |
| MeWo:Flutamide..Eulexin.:Ldose: -0.698970004 (uM) Interaction | 8.359040591 | 15.13733352 | 0.552213544 | 0.580807591 |
| SKMEL2:Flutamide..Eulexin.:Ldose: -0.698970004 (uM) Interaction | 5.060354253 | 15.31686613 | 0.330377912 | 0.741117533 |
| UACC0257:Flutamide..Eulexin.:Ldose: -0.698970004 (uM) Interaction | -1.10034308 | 15.05685414 | -0.073079215 | 0.941743737 |
| MeWo:Foretinib..GSK1363089.:Ldose: -0.698970004 (uM) Interaction | -12.11650871 | 15.13733352 | -0.800438776 | 0.423465151 |
| SKMEL2:Foretinib..GSK1363089.:Ldose: -0.698970004 (uM) Interaction | 12.57535062 | 15.31686613 | 0.821013288 | 0.411647478 |
| UACC0257:Foretinib..GSK1363089.:Ldose: -0.698970004 (uM) Interaction | 13.52384364 | 15.05685414 | 0.898185206 | 0.369096518 |
| SKMEL2:Fulvestrant:Ldose: -0.698970004 (uM) Interaction | -5.1197724 | 15.31686613 | -0.334257175 | 0.738188647 |
| UACC0257:Fulvestrant:Ldose: -0.698970004 (uM) Interaction | -1.730471613 | 15.05685414 | -0.114929161 | 0.908502306 |
| MeWo:Gefitinib:Ldose: -0.698970004 (uM) Interaction | 5.03700916 | 15.33308304 | 0.32850596 | 0.742532224 |
| MeWo:Gemcitabine.HCl:Ldose: -0.698970004 (uM) Interaction | 30.09222398 | 15.13733352 | 1.987947476 | 0.046829708 |
| SKMEL2:Gemcitabine.HCl:Ldose: -0.698970004 (uM) Interaction | 68.58116116 | 15.31686613 | 4.477493019 | 7.59E-06 |
| UACC0257:Gemcitabine.HCl:Ldose: -0.698970004 (uM) Interaction | -21.02940296 | 15.05685414 | -1.396666446 | 0.162527801 |
| MeWo:Ibrutinib..PCI.32765.:Ldose: -0.698970004 (uM) Interaction | -6.321552829 | 15.07335079 | -0.419386036 | 0.674938069 |
| SKMEL2:Ibrutinib..PCI.32765.:Ldose: -0.698970004 (uM) Interaction | -5.972162011 | 15.25363648 | -0.391523819 | 0.695413827 |
| MeWo:Imiquimod:Ldose: -0.698970004 (uM) Interaction | -2.536631496 | 15.07335079 | -0.16828584 | 0.86635994 |
| SKMEL2:Imiquimod:Ldose: -0.698970004 (uM) Interaction | 1.126742736 | 15.25363648 | 0.073867155 | 0.941116753 |
| MeWo:INK.128..MLN0128.:Ldose: -0.698970004 (uM) Interaction | 21.99947977 | 15.13733352 | 1.45332596 | 0.146147347 |
| SKMEL2:INK.128..MLN0128.:Ldose: -0.698970004 (uM) Interaction | 8.631955021 | 15.31686613 | 0.563558821 | 0.573060086 |
| UACC0257:INK.128..MLN0128.:Ldose: -0.698970004 (uM) Interaction | 1.615169265 | 15.05685414 | 0.107271363 | 0.914574668 |
| MeWo:Irinotecan.HCl:Ldose: -0.698970004 (uM) Interaction | 12.05146766 | 15.13733352 | 0.796142044 | 0.425957955 |
| SKMEL2:Irinotecan.HCl:Ldose: -0.698970004 (uM) Interaction | 11.64401696 | 15.31686613 | 0.760208835 | 0.447137803 |
| UACC0257:Irinotecan.HCl:Ldose: -0.698970004 (uM) Interaction | 9.730705645 | 15.05685414 | 0.64626419 | 0.518114927 |
| MeWo:Ixabepilone:Ldose: -0.698970004 (uM) Interaction | 5.983572653 | 15.13733352 | 0.395285778 | 0.692635776 |
| SKMEL2:Ixabepilone:Ldose: -0.698970004 (uM) Interaction | 10.23364333 | 15.31686613 | 0.668129058 | 0.504058131 |
| UACC0257:Ixabepilone:Ldose: -0.698970004 (uM) Interaction | -14.52487842 | 15.05685414 | -0.964668867 | 0.334721156 |
| MeWo:Lapatinib:Ldose: -0.698970004 (uM) Interaction | -4.077480746 | 15.07335079 | -0.270509245 | 0.786771009 |
| SKMEL2:Lapatinib:Ldose: -0.698970004 (uM) Interaction | -3.443721179 | 15.25363648 | -0.225763947 | 0.821387088 |
| MeWo:LDK378:Ldose: -0.698970004 (uM) Interaction | 11.65476419 | 15.13733352 | 0.769935086 | 0.441346557 |
| SKMEL2:LDK378:Ldose: -0.698970004 (uM) Interaction | 6.86244236 | 15.31686613 | 0.448031752 | 0.654134635 |
| UACC0257:LDK378:Ldose: -0.698970004 (uM) Interaction | -4.24174768 | 15.05685414 | -0.2817154 | 0.77816436 |
| MeWo:Lenalidomide:Ldose: -0.698970004 (uM) Interaction | 0.440141095 | 15.13733352 | 0.029076528 | 0.976803816 |
| SKMEL2:Lenalidomide:Ldose: -0.698970004 (uM) Interaction | -3.053576802 | 15.31686613 | -0.199360416 | 0.84198264 |
| SKMEL2:Letrozole:Ldose: -0.698970004 (uM) Interaction | 4.553652178 | 15.31686613 | 0.297296597 | 0.766242849 |
| MeWo:Linsitinib:Ldose: -0.698970004 (uM) Interaction | 10.39122887 | 15.13733352 | 0.686463627 | 0.492427932 |
| SKMEL2:Linsitinib:Ldose: -0.698970004 (uM) Interaction | 10.73884285 | 15.31686613 | 0.701112275 | 0.483240268 |
| UACC0257:Linsitinib:Ldose: -0.698970004 (uM) Interaction | -8.186172026 | 15.05685414 | -0.543684089 | 0.586664319 |
| SKMEL2:Lomustine..CCNU.:Ldose: -0.698970004 (uM) Interaction | -1.941699297 | 15.31686613 | -0.126768706 | 0.899124608 |
| MeWo:LY2157299:Ldose: -0.698970004 (uM) Interaction | -4.942520125 | 15.13733352 | -0.326511939 | 0.744040122 |
| SKMEL2:LY2157299:Ldose: -0.698970004 (uM) Interaction | -3.353699517 | 15.31686613 | -0.21895468 | 0.826687356 |
| MeWo:Mechlorethamine.HCl:Ldose: -0.698970004 (uM) Interaction | -2.32321824 | 15.13733352 | -0.153476056 | 0.87802426 |
| SKMEL2:Mechlorethamine.HCl:Ldose: -0.698970004 (uM) Interaction | -1.503463447 | 15.31686613 | -0.09815738 | 0.921808207 |
| UACC0257:Mechlorethamine.HCl:Ldose: -0.698970004 (uM) Interaction | 8.028086289 | 15.05685414 | 0.533184835 | 0.593910953 |
| MeWo:Megestrol.acetate:Ldose: -0.698970004 (uM) Interaction | 7.019733536 | 15.13733352 | 0.463736465 | 0.642841095 |
| SKMEL2:Megestrol.acetate:Ldose: -0.698970004 (uM) Interaction | 6.132816253 | 15.31686613 | 0.400396282 | 0.688868498 |
| MeWo:MEK.162..ARRY.438162.:Ldose: -0.698970004 (uM) Interaction | 30.98151815 | 15.13733352 | 2.04669588 | 0.040699654 |
| SKMEL2:MEK.162..ARRY.438162.:Ldose: -0.698970004 (uM) Interaction | 47.49524851 | 15.31686613 | 3.100846355 | 0.00193209 |
| UACC0257:MEK.162..ARRY.438162.:Ldose: -0.698970004 (uM) Interaction | 27.29297604 | 15.05685414 | 1.81266125 | 0.069897528 |
| MeWo:Melphalan:Ldose: -0.698970004 (uM) Interaction | -0.443098025 | 15.13733352 | -0.029271868 | 0.976648026 |
| SKMEL2:Melphalan:Ldose: -0.698970004 (uM) Interaction | -0.82948752 | 15.31686613 | -0.054155172 | 0.956812021 |
| UACC0257:Melphalan:Ldose: -0.698970004 (uM) Interaction | 11.46160832 | 15.05685414 | 0.76122198 | 0.446532543 |
| MeWo:Mercaptopurine:Ldose: -0.698970004 (uM) Interaction | 4.95156643 | 15.13733352 | 0.327109555 | 0.743588096 |
| SKMEL2:Mercaptopurine:Ldose: -0.698970004 (uM) Interaction | 9.781454068 | 15.31686613 | 0.638606748 | 0.52308535 |
| UACC0257:Mercaptopurine:Ldose: -0.698970004 (uM) Interaction | 27.89396253 | 15.05685414 | 1.852575728 | 0.0639564 |
| MeWo:Mitomycin.C:Ldose: -0.698970004 (uM) Interaction | -0.26650899 | 15.13733352 | -0.017606072 | 0.98595327 |
| SKMEL2:Mitomycin.C:Ldose: -0.698970004 (uM) Interaction | -1.071477815 | 15.31686613 | -0.069954115 | 0.944230808 |
| UACC0257:Mitomycin.C:Ldose: -0.698970004 (uM) Interaction | -14.79571026 | 15.05685414 | -0.982656146 | 0.325787364 |
| MeWo:Mitotane..o.p..DDD..Lysodren.:Ldose: -0.698970004 (uM) Interaction | 6.082222976 | 15.13733352 | 0.401802799 | 0.687833014 |
| SKMEL2:Mitotane..o.p..DDD..Lysodren.:Ldose: -0.698970004 (uM) Interaction | -10.59217809 | 15.31686613 | -0.691536898 | 0.489235402 |
| MeWo:Mitoxantrone:Ldose: -0.698970004 (uM) Interaction | 37.34571552 | 15.13733352 | 2.467126424 | 0.013627684 |
| SKMEL2:Mitoxantrone:Ldose: -0.698970004 (uM) Interaction | 47.81138813 | 15.31686613 | 3.121486323 | 0.001801705 |
| UACC0257:Mitoxantrone:Ldose: -0.698970004 (uM) Interaction | 29.38932392 | 15.05685414 | 1.95189006 | 0.05096376 |
| UACC0257:MLN.2480:Ldose: -0.698970004 (uM) Interaction | 0.462078289 | 15.05685414 | 0.0306889 | 0.975517918 |
| MeWo:MLN4924:Ldose: -0.698970004 (uM) Interaction | -20.81468158 | 15.13733352 | -1.37505602 | 0.169127895 |
| SKMEL2:MLN4924:Ldose: -0.698970004 (uM) Interaction | 11.6049549 | 15.31686613 | 0.757658571 | 0.448663414 |
| UACC0257:MLN4924:Ldose: -0.698970004 (uM) Interaction | 6.434836994 | 15.05685414 | 0.427369285 | 0.669114502 |
| MeWo:MLN9708..MLN2238.:Ldose: -0.698970004 (uM) Interaction | 0.529094321 | 15.13733352 | 0.034952941 | 0.972117579 |
| SKMEL2:MLN9708..MLN2238.:Ldose: -0.698970004 (uM) Interaction | -24.58896994 | 15.31686613 | -1.60535254 | 0.108430405 |
| UACC0257:MLN9708..MLN2238.:Ldose: -0.698970004 (uM) Interaction | -7.727282533 | 15.05685414 | -0.513206973 | 0.607811634 |
| MeWo:Navitoclax..ABT.263..5uM:Ldose: -0.698970004 (uM) Interaction | -1.679898794 | 15.13733352 | -0.110977194 | 0.911635431 |
| SKMEL2:Navitoclax..ABT.263..5uM:Ldose: -0.698970004 (uM) Interaction | 4.173391143 | 15.31686613 | 0.272470302 | 0.785262946 |
| UACC0257:Navitoclax..ABT.263..5uM:Ldose: -0.698970004 (uM) Interaction | 13.68741755 | 15.05685414 | 0.909048957 | 0.36333411 |
| MeWo:Nelarabine:Ldose: -0.698970004 (uM) Interaction | 3.419771195 | 15.13733352 | 0.225916354 | 0.821268549 |
| SKMEL2:Nelarabine:Ldose: -0.698970004 (uM) Interaction | -10.05838227 | 15.31686613 | -0.6566867 | 0.511389166 |
| UACC0257:Nelarabine:Ldose: -0.698970004 (uM) Interaction | 1.891097294 | 15.05685414 | 0.125597105 | 0.900051987 |
| MeWo:OSI.027:Ldose: -0.698970004 (uM) Interaction | -20.25062289 | 15.13733352 | -1.337793268 | 0.180977482 |
| SKMEL2:OSI.027:Ldose: -0.698970004 (uM) Interaction | -11.2024081 | 15.31686613 | -0.731377294 | 0.464556417 |
| UACC0257:OSI.027:Ldose: -0.698970004 (uM) Interaction | -17.70687451 | 15.05685414 | -1.176000933 | 0.23960706 |
| MeWo:Oxaliplatin:Ldose: -0.698970004 (uM) Interaction | 9.682214021 | 15.07335079 | 0.642339859 | 0.520659144 |
| SKMEL2:Oxaliplatin:Ldose: -0.698970004 (uM) Interaction | 15.68273239 | 15.25363648 | 1.028130729 | 0.303899491 |
| MeWo:Paclitaxel:Ldose: -0.698970004 (uM) Interaction | 7.502986066 | 15.13733352 | 0.495661013 | 0.620138484 |
| SKMEL2:Paclitaxel:Ldose: -0.698970004 (uM) Interaction | -0.879729069 | 15.31686613 | -0.057435317 | 0.954198945 |
| MeWo:Palbociclib..PD.0332991..Isethionate:Ldose: -0.698970004 (uM) Interaction | 7.963062766 | 15.13733352 | 0.526054523 | 0.598855557 |
| SKMEL2:Palbociclib..PD.0332991..Isethionate:Ldose: -0.698970004 (uM) Interaction | 14.56308687 | 15.31686613 | 0.950787632 | 0.341722499 |
| UACC0257:Palbociclib..PD.0332991..Isethionate:Ldose: -0.698970004 (uM) Interaction | 1.574824677 | 15.05685414 | 0.10459188 | 0.916700599 |
| MeWo:Pazopanib.HCl:Ldose: -0.698970004 (uM) Interaction | 3.168601375 | 15.13733352 | 0.209323615 | 0.83419753 |
| SKMEL2:Pazopanib.HCl:Ldose: -0.698970004 (uM) Interaction | 2.18749237 | 15.31686613 | 0.142815923 | 0.886436852 |
| UACC0257:Pazopanib.HCl:Ldose: -0.698970004 (uM) Interaction | 6.579092004 | 15.05685414 | 0.436949973 | 0.662151861 |
| MeWo:PD325901:Ldose: -0.698970004 (uM) Interaction | -16.62514326 | 15.13733352 | -1.098287438 | 0.272090855 |
| SKMEL2:PD325901:Ldose: -0.698970004 (uM) Interaction | -23.01137152 | 15.31686613 | -1.502355073 | 0.133019595 |
| UACC0257:PD325901:Ldose: -0.698970004 (uM) Interaction | -4.747434126 | 15.05685414 | -0.315300532 | 0.752536432 |
| SKMEL2:Pemetrexed:Ldose: -0.698970004 (uM) Interaction | 7.463902281 | 15.31686613 | 0.48729957 | 0.626050843 |
| MeWo:Plicamycin:Ldose: -0.698970004 (uM) Interaction | 22.78023664 | 15.13733352 | 1.50490419 | 0.132362876 |
| SKMEL2:Plicamycin:Ldose: -0.698970004 (uM) Interaction | 16.07437704 | 15.31686613 | 1.049455998 | 0.293979672 |
| UACC0257:Plicamycin:Ldose: -0.698970004 (uM) Interaction | 3.701707727 | 15.05685414 | 0.245848681 | 0.805801634 |
| MeWo:Pralatrexate:Ldose: -0.698970004 (uM) Interaction | 6.454819586 | 15.07335079 | 0.428227252 | 0.668489814 |
| SKMEL2:Pralatrexate:Ldose: -0.698970004 (uM) Interaction | 6.900637343 | 15.25363648 | 0.452392933 | 0.650990339 |
| MeWo:Quinacrine.HCl:Ldose: -0.698970004 (uM) Interaction | -8.942821885 | 15.13733352 | -0.590779206 | 0.554674357 |
| SKMEL2:Quinacrine.HCl:Ldose: -0.698970004 (uM) Interaction | -3.77549789 | 15.31686613 | -0.246492844 | 0.805303013 |
| UACC0257:Quinacrine.HCl:Ldose: -0.698970004 (uM) Interaction | -6.151896923 | 15.05685414 | -0.408577839 | 0.682853421 |
| MeWo:Quizartinib:Ldose: -0.698970004 (uM) Interaction | 2.624512341 | 15.13733352 | 0.173380096 | 0.862354243 |
| SKMEL2:Quizartinib:Ldose: -0.698970004 (uM) Interaction | 5.15973734 | 15.31686613 | 0.336866386 | 0.736220796 |
| UACC0257:Quizartinib:Ldose: -0.698970004 (uM) Interaction | 1.566155937 | 15.05685414 | 0.104016146 | 0.917157471 |
| MeWo:Raloxifene:Ldose: -0.698970004 (uM) Interaction | 8.417386804 | 15.07335079 | 0.558428376 | 0.576557508 |
| SKMEL2:Raloxifene:Ldose: -0.698970004 (uM) Interaction | 7.422608483 | 15.25363648 | 0.486612389 | 0.626537826 |
| MeWo:Romidepsin:Ldose: -0.698970004 (uM) Interaction | 5.661713471 | 15.13733352 | 0.37402317 | 0.708390635 |
| SKMEL2:Romidepsin:Ldose: -0.698970004 (uM) Interaction | 6.091719613 | 15.31686613 | 0.397713185 | 0.690845425 |
| UACC0257:Romidepsin:Ldose: -0.698970004 (uM) Interaction | -2.860387471 | 15.05685414 | -0.18997245 | 0.849332448 |
| MeWo:Sabutoclax..BI.97C1.:Ldose: -0.698970004 (uM) Interaction | 0.428874333 | 15.13733352 | 0.028332225 | 0.977397433 |
| SKMEL2:Sabutoclax..BI.97C1.:Ldose: -0.698970004 (uM) Interaction | 5.53627053 | 15.31686613 | 0.361449299 | 0.717767025 |
| UACC0257:Sabutoclax..BI.97C1.:Ldose: -0.698970004 (uM) Interaction | 12.45361247 | 15.05685414 | 0.827105872 | 0.408185908 |
| MeWo:Sirolimus..Rapamycin.:Ldose: -0.698970004 (uM) Interaction | -6.083454675 | 15.13733352 | -0.401884167 | 0.687773128 |
| SKMEL2:Sirolimus..Rapamycin.:Ldose: -0.698970004 (uM) Interaction | -6.17084397 | 15.31686613 | -0.402879017 | 0.687041091 |
| MeWo:Sorafenib:Ldose: -0.698970004 (uM) Interaction | 3.001340588 | 15.13733352 | 0.198274061 | 0.842832453 |
| UACC0257:Sorafenib:Ldose: -0.698970004 (uM) Interaction | 6.335642437 | 15.05685414 | 0.420781285 | 0.673918854 |
| MeWo:Streptozocin:Ldose: -0.698970004 (uM) Interaction | 3.49713508 | 15.13733352 | 0.231027154 | 0.817295827 |
| SKMEL2:Streptozocin:Ldose: -0.698970004 (uM) Interaction | -11.40534886 | 15.31686613 | -0.74462679 | 0.456505216 |
| UACC0257:Streptozocin:Ldose: -0.698970004 (uM) Interaction | 6.165230194 | 15.05685414 | 0.409463367 | 0.682203579 |
| MeWo:Sunitinib:Ldose: -0.698970004 (uM) Interaction | 1.533250428 | 15.13733352 | 0.101289334 | 0.919321694 |
| UACC0257:Sunitinib:Ldose: -0.698970004 (uM) Interaction | 7.46814969 | 15.05685414 | 0.495996682 | 0.619901641 |
| MeWo:Tamoxifen.Citrate:Ldose: -0.698970004 (uM) Interaction | 8.332628091 | 15.13733352 | 0.550468686 | 0.582003464 |
| SKMEL2:Tamoxifen.Citrate:Ldose: -0.698970004 (uM) Interaction | 7.222384383 | 15.31686613 | 0.471531469 | 0.637265867 |
| UACC0257:Tamoxifen.Citrate:Ldose: -0.698970004 (uM) Interaction | 0.47938162 | 15.05685414 | 0.0318381 | 0.974601448 |
| MeWo:Temozolomide:Ldose: -0.698970004 (uM) Interaction | -0.944951957 | 15.33308304 | -0.061628308 | 0.950859386 |
| SKMEL2:Temsirolimus..CCI.779..Torisel.:Ldose: -0.698970004 (uM) Interaction | 9.427698645 | 15.31686613 | 0.615510938 | 0.53822363 |
| MeWo:Teniposide:Ldose: -0.698970004 (uM) Interaction | 5.594580665 | 15.13733352 | 0.369588254 | 0.711692815 |
| SKMEL2:Teniposide:Ldose: -0.698970004 (uM) Interaction | 16.32072843 | 15.31686613 | 1.065539667 | 0.286643324 |
| UACC0257:Teniposide:Ldose: -0.698970004 (uM) Interaction | 10.61922971 | 15.05685414 | 0.705275459 | 0.480646198 |
| MeWo:Thioguanine:Ldose: -0.698970004 (uM) Interaction | -0.19925521 | 15.13733352 | -0.013163164 | 0.989497735 |
| SKMEL2:Thioguanine:Ldose: -0.698970004 (uM) Interaction | 0.357017533 | 15.31686613 | 0.023308785 | 0.981404173 |
| UACC0257:Thioguanine:Ldose: -0.698970004 (uM) Interaction | 3.352242189 | 15.05685414 | 0.22263895 | 0.823818559 |
| MeWo:Thiotepa:Ldose: -0.698970004 (uM) Interaction | 3.179359023 | 15.13733352 | 0.210034285 | 0.833642833 |
| SKMEL2:Thiotepa:Ldose: -0.698970004 (uM) Interaction | -1.641601485 | 15.31686613 | -0.107176068 | 0.914650265 |
| UACC0257:Thiotepa:Ldose: -0.698970004 (uM) Interaction | 11.97591014 | 15.05685414 | 0.795379302 | 0.426401363 |
| MeWo:Topotecan.HCl:Ldose: -0.698970004 (uM) Interaction | 53.65445354 | 15.13733352 | 3.544511552 | 0.000394146 |
| SKMEL2:Topotecan.HCl:Ldose: -0.698970004 (uM) Interaction | 39.1700602 | 15.31686613 | 2.557315568 | 0.010554905 |
| UACC0257:Topotecan.HCl:Ldose: -0.698970004 (uM) Interaction | 21.06024784 | 15.05685414 | 1.398715007 | 0.161912372 |
| MeWo:Trametinib..GSK1120212.:Ldose: -0.698970004 (uM) Interaction | -5.043734997 | 15.13733352 | -0.333198379 | 0.738987674 |
| UACC0257:Trametinib..GSK1120212.:Ldose: -0.698970004 (uM) Interaction | 5.51345736 | 15.05685414 | 0.366175916 | 0.714237286 |
| MeWo:Triethylenemelamine:Ldose: -0.698970004 (uM) Interaction | -3.296118342 | 15.13733352 | -0.21774762 | 0.827627751 |
| SKMEL2:Triethylenemelamine:Ldose: -0.698970004 (uM) Interaction | -0.778023082 | 15.31686613 | -0.050795187 | 0.959489181 |
| UACC0257:Triethylenemelamine:Ldose: -0.698970004 (uM) Interaction | -3.83964153 | 15.05685414 | -0.255009545 | 0.798718079 |
| MeWo:Uracil.mustard:Ldose: -0.698970004 (uM) Interaction | 5.721987485 | 15.13733352 | 0.378004982 | 0.705430492 |
| SKMEL2:Uracil.mustard:Ldose: -0.698970004 (uM) Interaction | 4.822064778 | 15.31686613 | 0.314820587 | 0.752900826 |
| MeWo:Valrubicin:Ldose: -0.698970004 (uM) Interaction | 15.73010574 | 15.13733352 | 1.039159619 | 0.298741776 |
| SKMEL2:Valrubicin:Ldose: -0.698970004 (uM) Interaction | 7.037296935 | 15.31686613 | 0.459447571 | 0.645917273 |
| UACC0257:Valrubicin:Ldose: -0.698970004 (uM) Interaction | 7.64174418 | 15.05685414 | 0.507525949 | 0.611790854 |
| MeWo:Vandetanib:Ldose: -0.698970004 (uM) Interaction | -15.7687827 | 15.07335079 | -1.046136517 | 0.295509348 |
| SKMEL2:Vandetanib:Ldose: -0.698970004 (uM) Interaction | -9.334194786 | 15.25363648 | -0.611932427 | 0.540588706 |
| SKMEL2:Vemurafenib:Ldose: -0.698970004 (uM) Interaction | 41.44918881 | 15.31686613 | 2.706114192 | 0.006812741 |
| UACC0257:Vemurafenib:Ldose: -0.698970004 (uM) Interaction | 29.21829042 | 15.05685414 | 1.94053088 | 0.052327794 |
| SKMEL2:Vinblastine.Sulfate:Ldose: -0.698970004 (uM) Interaction | 7.603625856 | 15.31686613 | 0.496421774 | 0.61960176 |
| UACC0257:Vinblastine.Sulfate:Ldose: -0.698970004 (uM) Interaction | 16.60532965 | 15.05685414 | 1.102841901 | 0.270107732 |
| MeWo:Vincristine.Sulfate:Ldose: -0.698970004 (uM) Interaction | 33.83929176 | 15.13733352 | 2.235485643 | 0.025395342 |
| SKMEL2:Vincristine.Sulfate:Ldose: -0.698970004 (uM) Interaction | 48.64319351 | 15.31686613 | 3.175792823 | 0.00149631 |
| UACC0257:Vincristine.Sulfate:Ldose: -0.698970004 (uM) Interaction | 42.95555296 | 15.05685414 | 2.852890289 | 0.004336344 |
| MeWo:Vinorelbine.Tartrate:Ldose: -0.698970004 (uM) Interaction | -33.06343815 | 15.13733352 | -2.184231331 | 0.028955635 |
| SKMEL2:Vinorelbine.Tartrate:Ldose: -0.698970004 (uM) Interaction | -23.72132155 | 15.31686613 | -1.54870594 | 0.121466619 |
| UACC0257:Vinorelbine.Tartrate:Ldose: -0.698970004 (uM) Interaction | -35.78544574 | 15.05685414 | -2.376688079 | 0.017477244 |
| MeWo:Vismodegib:Ldose: -0.698970004 (uM) Interaction | 0.977018223 | 15.13733352 | 0.064543615 | 0.948537959 |
| SKMEL2:Vismodegib:Ldose: -0.698970004 (uM) Interaction | -12.36814763 | 15.31686613 | -0.807485521 | 0.419395427 |
| UACC0257:Vismodegib:Ldose: -0.698970004 (uM) Interaction | 14.64216254 | 15.05685414 | 0.972458284 | 0.330833158 |
| MeWo:Vorinostat:Ldose: -0.698970004 (uM) Interaction | 5.013443701 | 15.13733352 | 0.331197281 | 0.740498587 |
| SKMEL2:Vorinostat:Ldose: -0.698970004 (uM) Interaction | 1.175407885 | 15.31686613 | 0.07673945 | 0.938831509 |
| UACC0257:Vorinostat:Ldose: -0.698970004 (uM) Interaction | 9.977686366 | 15.05685414 | 0.662667399 | 0.507550444 |
| MeWo:Zoledronic.Acid:Ldose: -0.698970004 (uM) Interaction | 4.952302006 | 15.33308304 | 0.32298149 | 0.746712289 |
| MeWo:Abiraterone:Ldose: -0.397940009 (uM) Interaction | 3.231079275 | 15.13733352 | 0.21345102 | 0.830977141 |
| SKMEL2:Abiraterone:Ldose: -0.397940009 (uM) Interaction | 1.698107405 | 15.31686613 | 0.110865199 | 0.911724241 |
| MeWo:ABT.737:Ldose: -0.397940009 (uM) Interaction | -14.44241701 | 15.13733352 | -0.954092541 | 0.340047145 |
| SKMEL2:ABT.737:Ldose: -0.397940009 (uM) Interaction | -6.788598045 | 15.31686613 | -0.44321064 | 0.657617684 |
| UACC0257:ABT.737:Ldose: -0.397940009 (uM) Interaction | -13.73993876 | 15.05685414 | -0.91253715 | 0.361495889 |
| MeWo:Actinomycin.D:Ldose: -0.397940009 (uM) Interaction | -7.108870162 | 15.13733352 | -0.469624994 | 0.638627557 |
| SKMEL2:Actinomycin.D:Ldose: -0.397940009 (uM) Interaction | -36.91924221 | 15.31686613 | -2.410365273 | 0.015944597 |
| UACC0257:Actinomycin.D:Ldose: -0.397940009 (uM) Interaction | -27.73297879 | 15.05685414 | -1.841884004 | 0.065505391 |
| MeWo:Afatinib:Ldose: -0.397940009 (uM) Interaction | 7.393376592 | 15.13733352 | 0.48842001 | 0.625257175 |
| SKMEL2:Afatinib:Ldose: -0.397940009 (uM) Interaction | 8.143176016 | 15.31686613 | 0.531647659 | 0.594975346 |
| UACC0257:Afatinib:Ldose: -0.397940009 (uM) Interaction | 5.532314671 | 15.05685414 | 0.367428323 | 0.713303035 |
| MeWo:Alisertib..MLN8237.:Ldose: -0.397940009 (uM) Interaction | 22.76240621 | 15.13733352 | 1.503726279 | 0.132666024 |
| SKMEL2:Alisertib..MLN8237.:Ldose: -0.397940009 (uM) Interaction | 20.93297273 | 15.31686613 | 1.366661597 | 0.171745181 |
| MeWo:Allopurinol:Ldose: -0.397940009 (uM) Interaction | -3.624259592 | 15.13733352 | -0.239425232 | 0.810778067 |
| SKMEL2:Allopurinol:Ldose: -0.397940009 (uM) Interaction | -3.553793677 | 15.31686613 | -0.232018329 | 0.816525908 |
| UACC0257:Allopurinol:Ldose: -0.397940009 (uM) Interaction | 4.748672863 | 15.05685414 | 0.315382803 | 0.752473974 |
| MeWo:Amifostine:Ldose: -0.397940009 (uM) Interaction | -1.504984292 | 15.13733352 | -0.099422021 | 0.920804093 |
| MeWo:Aphrocallistin.analogue:Ldose: -0.397940009 (uM) Interaction | 6.716677053 | 15.13733352 | 0.443715998 | 0.657252233 |
| SKMEL2:Aphrocallistin.analogue:Ldose: -0.397940009 (uM) Interaction | 42.43365304 | 15.31686613 | 2.770387407 | 0.005603599 |
| UACC0257:Aphrocallistin.analogue:Ldose: -0.397940009 (uM) Interaction | 43.85804746 | 15.05685414 | 2.912829403 | 0.003585256 |
| MeWo:Arsenic.Trioxide:Ldose: -0.397940009 (uM) Interaction | 10.45389686 | 15.13733352 | 0.690603589 | 0.489821881 |
| SKMEL2:Arsenic.Trioxide:Ldose: -0.397940009 (uM) Interaction | 4.824326068 | 15.31686613 | 0.314968221 | 0.752788731 |
| MeWo:Axitinib:Ldose: -0.397940009 (uM) Interaction | 7.765150518 | 15.13733352 | 0.512980077 | 0.607970339 |
| SKMEL2:Axitinib:Ldose: -0.397940009 (uM) Interaction | 1.581068131 | 15.31686613 | 0.103223996 | 0.917786124 |
| UACC0257:Axitinib:Ldose: -0.397940009 (uM) Interaction | -5.202067385 | 15.05685414 | -0.345494971 | 0.72972555 |
| MeWo:Axitinib.1:Ldose: -0.397940009 (uM) Interaction | 8.096828975 | 15.13733352 | 0.534891364 | 0.592730315 |
| SKMEL2:Axitinib.1:Ldose: -0.397940009 (uM) Interaction | 14.18800248 | 15.31686613 | 0.926299307 | 0.354300482 |
| UACC0257:Axitinib.1:Ldose: -0.397940009 (uM) Interaction | -2.067188479 | 15.05685414 | -0.13729219 | 0.890801083 |
| MeWo:Azacitidine:Ldose: -0.397940009 (uM) Interaction | 1.956426418 | 15.13733352 | 0.129245115 | 0.897164864 |
| SKMEL2:Azacitidine:Ldose: -0.397940009 (uM) Interaction | -9.62731256 | 15.31686613 | -0.628543233 | 0.529654573 |
| UACC0257:Azacitidine:Ldose: -0.397940009 (uM) Interaction | 0.283615683 | 15.05685414 | 0.018836317 | 0.98497185 |
| SKMEL2:Baricitinib..LY3009104..INCB028050.:Ldose: -0.397940009 (uM) Interaction | 14.31514166 | 15.31686613 | 0.934599907 | 0.350004649 |
| UACC0257:Baricitinib..LY3009104..INCB028050.:Ldose: -0.397940009 (uM) Interaction | -3.560569945 | 15.05685414 | -0.236475024 | 0.813066258 |
| SKMEL2:Bendamustine.HCl:Ldose: -0.397940009 (uM) Interaction | 14.37948968 | 15.25363648 | 0.942692564 | 0.345848392 |
| MeWo:Bioymifi:Ldose: -0.397940009 (uM) Interaction | -2.974919568 | 15.13733352 | -0.19652864 | 0.84419821 |
| SKMEL2:Bioymifi:Ldose: -0.397940009 (uM) Interaction | -3.851291514 | 15.31686613 | -0.251441221 | 0.801475319 |
| UACC0257:Bioymifi:Ldose: -0.397940009 (uM) Interaction | -12.20722386 | 15.05685414 | -0.810741988 | 0.417522509 |
| MeWo:Bleomycin.Sulfate:Ldose: -0.397940009 (uM) Interaction | 24.56837128 | 15.13733352 | 1.623031642 | 0.104596779 |
| SKMEL2:Bleomycin.Sulfate:Ldose: -0.397940009 (uM) Interaction | 19.01446726 | 15.31686613 | 1.241407159 | 0.214468436 |
| UACC0257:Bleomycin.Sulfate:Ldose: -0.397940009 (uM) Interaction | 41.19769308 | 15.05685414 | 2.736142138 | 0.006221336 |
| MeWo:Bortezomib:Ldose: -0.397940009 (uM) Interaction | 10.05590208 | 15.13733352 | 0.664311324 | 0.506497945 |
| SKMEL2:Bortezomib:Ldose: -0.397940009 (uM) Interaction | 20.48672969 | 15.31686613 | 1.337527502 | 0.181064155 |
| UACC0257:Bortezomib:Ldose: -0.397940009 (uM) Interaction | 18.6237134 | 15.05685414 | 1.236892728 | 0.216139938 |
| MeWo:Bosutinib..SKI.606.:Ldose: -0.397940009 (uM) Interaction | 23.59046463 | 15.13733352 | 1.558429336 | 0.1191457 |
| SKMEL2:Bosutinib..SKI.606.:Ldose: -0.397940009 (uM) Interaction | 25.8641321 | 15.31686613 | 1.688604698 | 0.091309194 |
| UACC0257:Bosutinib..SKI.606.:Ldose: -0.397940009 (uM) Interaction | 5.17667951 | 15.05685414 | 0.343808837 | 0.730993296 |
| SKMEL2:Busulfan:Ldose: -0.397940009 (uM) Interaction | 7.178841236 | 15.31686613 | 0.468688645 | 0.639296786 |
| MeWo:Cabazitaxel:Ldose: -0.397940009 (uM) Interaction | 8.272496437 | 15.13733352 | 0.546496278 | 0.584730314 |
| SKMEL2:Cabazitaxel:Ldose: -0.397940009 (uM) Interaction | 9.1977797 | 15.31686613 | 0.600500104 | 0.548179095 |
| UACC0257:Cabazitaxel:Ldose: -0.397940009 (uM) Interaction | 1.192865488 | 15.05685414 | 0.079224085 | 0.936855099 |
| MeWo:Cabozantinib..XL.184.:Ldose: -0.397940009 (uM) Interaction | -3.966872112 | 15.13733352 | -0.262058843 | 0.793278498 |
| SKMEL2:Cabozantinib..XL.184.:Ldose: -0.397940009 (uM) Interaction | 12.21265743 | 15.31686613 | 0.797333954 | 0.425265597 |
| UACC0257:Cabozantinib..XL.184.:Ldose: -0.397940009 (uM) Interaction | -5.781144625 | 15.05685414 | -0.383954349 | 0.701015945 |
| MeWo:Capecitabine:Ldose: -0.397940009 (uM) Interaction | 0.033729109 | 15.33308304 | 0.00219976 | 0.998244866 |
| MeWo:Carfilzomib:Ldose: -0.397940009 (uM) Interaction | -32.52701917 | 15.13733352 | -2.14879451 | 0.031661427 |
| SKMEL2:Carfilzomib:Ldose: -0.397940009 (uM) Interaction | 38.1793592 | 15.31686613 | 2.49263517 | 0.012687105 |
| UACC0257:Carfilzomib:Ldose: -0.397940009 (uM) Interaction | -39.88666907 | 15.05685414 | -2.649070563 | 0.008077036 |
| MeWo:Carmustine:Ldose: -0.397940009 (uM) Interaction | -4.537220495 | 15.33308304 | -0.295910515 | 0.767301177 |
| MeWo:Celecoxib:Ldose: -0.397940009 (uM) Interaction | 1.533858119 | 15.33308304 | 0.100035858 | 0.920316757 |
| UACC0257:Chlorambucil:Ldose: -0.397940009 (uM) Interaction | -15.37935678 | 15.05685414 | -1.021418992 | 0.307067053 |
| MeWo:Cisplatin:Ldose: -0.397940009 (uM) Interaction | 6.994726225 | 15.13733352 | 0.462084436 | 0.64402528 |
| SKMEL2:Cisplatin:Ldose: -0.397940009 (uM) Interaction | 5.776730271 | 15.31686613 | 0.377148316 | 0.706066976 |
| MeWo:Cladribine:Ldose: -0.397940009 (uM) Interaction | 71.88136551 | 15.13733352 | 4.748614767 | 2.06E-06 |
| SKMEL2:Cladribine:Ldose: -0.397940009 (uM) Interaction | 68.39171522 | 15.31686613 | 4.465124566 | 8.04E-06 |
| UACC0257:Cladribine:Ldose: -0.397940009 (uM) Interaction | 71.53111816 | 15.05685414 | 4.750734616 | 2.04E-06 |
| MeWo:Clofarabine:Ldose: -0.397940009 (uM) Interaction | 49.92442995 | 15.13733352 | 3.298099356 | 0.00097494 |
| SKMEL2:Clofarabine:Ldose: -0.397940009 (uM) Interaction | 34.75618016 | 15.31686613 | 2.269144345 | 0.023269061 |
| UACC0257:Clofarabine:Ldose: -0.397940009 (uM) Interaction | 25.12757056 | 15.05685414 | 1.668845984 | 0.095161947 |
| MeWo:Crizotinib:Ldose: -0.397940009 (uM) Interaction | -1.352219272 | 15.13733352 | -0.089330084 | 0.928820389 |
| SKMEL2:Crizotinib:Ldose: -0.397940009 (uM) Interaction | 4.256006461 | 15.31686613 | 0.27786405 | 0.781119298 |
| UACC0257:Crizotinib:Ldose: -0.397940009 (uM) Interaction | -10.17682643 | 15.05685414 | -0.675893273 | 0.499115419 |
| MeWo:Cytarabine.HCl...Ara.C:Ldose: -0.397940009 (uM) Interaction | 53.89721063 | 15.13733352 | 3.56054853 | 0.000370851 |
| SKMEL2:Cytarabine.HCl...Ara.C:Ldose: -0.397940009 (uM) Interaction | 65.95034642 | 15.31686613 | 4.305733684 | 1.67E-05 |
| UACC0257:Cytarabine.HCl...Ara.C:Ldose: -0.397940009 (uM) Interaction | 44.60467904 | 15.05685414 | 2.962416892 | 0.003055562 |
| MeWo:Dacarbazine:Ldose: -0.397940009 (uM) Interaction | -1.5116598 | 15.13733352 | -0.099863017 | 0.920453975 |
| SKMEL2:Dacarbazine:Ldose: -0.397940009 (uM) Interaction | -7.559151645 | 15.31686613 | -0.493518164 | 0.62165137 |
| MeWo:Dacomitinib..PF299804.:Ldose: -0.397940009 (uM) Interaction | -2.886630393 | 15.13733352 | -0.190696095 | 0.848765434 |
| SKMEL2:Dacomitinib..PF299804.:Ldose: -0.397940009 (uM) Interaction | -2.304687225 | 15.31686613 | -0.150467283 | 0.880397323 |
| UACC0257:Dacomitinib..PF299804.:Ldose: -0.397940009 (uM) Interaction | 2.980620186 | 15.05685414 | 0.197957698 | 0.843079965 |
| MeWo:Dasatinib:Ldose: -0.397940009 (uM) Interaction | -8.586883047 | 15.13733352 | -0.567265234 | 0.570539705 |
| SKMEL2:Dasatinib:Ldose: -0.397940009 (uM) Interaction | -11.57852885 | 15.31686613 | -0.75593328 | 0.449697186 |
| UACC0257:Dasatinib:Ldose: -0.397940009 (uM) Interaction | 6.6818784 | 15.05685414 | 0.443776524 | 0.657208469 |
| MeWo:Daunorubicin.HCl:Ldose: -0.397940009 (uM) Interaction | 27.63362656 | 15.13733352 | 1.825528024 | 0.067934756 |
| SKMEL2:Daunorubicin.HCl:Ldose: -0.397940009 (uM) Interaction | 51.92984899 | 15.31686613 | 3.390370364 | 0.000699193 |
| UACC0257:Daunorubicin.HCl:Ldose: -0.397940009 (uM) Interaction | 30.68061618 | 15.05685414 | 2.037651152 | 0.041596613 |
| MeWo:Decitabine:Ldose: -0.397940009 (uM) Interaction | -1.408600242 | 15.13733352 | -0.093054714 | 0.92586093 |
| SKMEL2:Decitabine:Ldose: -0.397940009 (uM) Interaction | 2.644299275 | 15.31686613 | 0.172639707 | 0.862936206 |
| MeWo:Docetaxel:Ldose: -0.397940009 (uM) Interaction | 16.45765954 | 15.13733352 | 1.087223157 | 0.276949952 |
| MeWo:Doxorubicin.HCl:Ldose: -0.397940009 (uM) Interaction | 39.78851629 | 15.13733352 | 2.62850232 | 0.008582056 |
| SKMEL2:Doxorubicin.HCl:Ldose: -0.397940009 (uM) Interaction | 31.57479619 | 15.31686613 | 2.061439718 | 0.039272675 |
| UACC0257:Doxorubicin.HCl:Ldose: -0.397940009 (uM) Interaction | 26.84888369 | 15.05685414 | 1.783166885 | 0.074572741 |
| MeWo:Erlotinib.HCl:Ldose: -0.397940009 (uM) Interaction | -20.79064882 | 15.13733352 | -1.373468371 | 0.169620597 |
| SKMEL2:Erlotinib.HCl:Ldose: -0.397940009 (uM) Interaction | -15.08066563 | 15.31686613 | -0.984579058 | 0.324841564 |
| UACC0257:Erlotinib.HCl:Ldose: -0.397940009 (uM) Interaction | -10.72186869 | 15.05685414 | -0.71209222 | 0.476415123 |
| MeWo:Etoposide:Ldose: -0.397940009 (uM) Interaction | 17.66821406 | 15.13733352 | 1.167194607 | 0.243144271 |
| SKMEL2:Etoposide:Ldose: -0.397940009 (uM) Interaction | 15.53300997 | 15.31686613 | 1.014111493 | 0.310540559 |
| UACC0257:Etoposide:Ldose: -0.397940009 (uM) Interaction | 15.31601967 | 15.05685414 | 1.017212462 | 0.309063407 |
| MeWo:Everolimus:Ldose: -0.397940009 (uM) Interaction | -10.26345432 | 15.33308304 | -0.669366643 | 0.503268556 |
| MeWo:Exemestane:Ldose: -0.397940009 (uM) Interaction | 3.697208204 | 15.07335079 | 0.245281109 | 0.806241035 |
| SKMEL2:Exemestane:Ldose: -0.397940009 (uM) Interaction | 4.533547874 | 15.25363648 | 0.297210956 | 0.766308227 |
| MeWo:Floxuridine:Ldose: -0.397940009 (uM) Interaction | 26.7929473 | 15.13733352 | 1.769991211 | 0.076742269 |
| SKMEL2:Floxuridine:Ldose: -0.397940009 (uM) Interaction | 55.74789181 | 15.31686613 | 3.63964086 | 0.000273635 |
| UACC0257:Floxuridine:Ldose: -0.397940009 (uM) Interaction | 21.06874686 | 15.05685414 | 1.399279469 | 0.161743106 |
| MeWo:Fludarabine.Phosphate:Ldose: -0.397940009 (uM) Interaction | -7.653964313 | 15.13733352 | -0.505634913 | 0.613117966 |
| SKMEL2:Fludarabine.Phosphate:Ldose: -0.397940009 (uM) Interaction | -5.996859349 | 15.31686613 | -0.391519995 | 0.695416654 |
| MeWo:Fluorouracil...5.FU.:Ldose: -0.397940009 (uM) Interaction | -5.710269543 | 15.13733352 | -0.377230873 | 0.706005629 |
| SKMEL2:Fluorouracil...5.FU.:Ldose: -0.397940009 (uM) Interaction | -8.94033156 | 15.31686613 | -0.583691957 | 0.559433484 |
| UACC0257:Fluorouracil...5.FU.:Ldose: -0.397940009 (uM) Interaction | -2.864829847 | 15.05685414 | -0.19026749 | 0.84910126 |
| MeWo:Flutamide..Eulexin.:Ldose: -0.397940009 (uM) Interaction | 3.216398742 | 15.13733352 | 0.212481197 | 0.831733588 |
| SKMEL2:Flutamide..Eulexin.:Ldose: -0.397940009 (uM) Interaction | 4.99549404 | 15.31686613 | 0.326143351 | 0.744318961 |
| UACC0257:Flutamide..Eulexin.:Ldose: -0.397940009 (uM) Interaction | -9.787672929 | 15.05685414 | -0.650047669 | 0.515668128 |
| MeWo:Foretinib..GSK1363089.:Ldose: -0.397940009 (uM) Interaction | -9.18168844 | 15.13733352 | -0.606559169 | 0.54414968 |
| SKMEL2:Foretinib..GSK1363089.:Ldose: -0.397940009 (uM) Interaction | 23.88084232 | 15.31686613 | 1.559120653 | 0.118982019 |
| UACC0257:Foretinib..GSK1363089.:Ldose: -0.397940009 (uM) Interaction | -0.384992427 | 15.05685414 | -0.025569247 | 0.979601144 |
| SKMEL2:Fulvestrant:Ldose: -0.397940009 (uM) Interaction | -2.556570665 | 15.31686613 | -0.166912124 | 0.867440707 |
| UACC0257:Fulvestrant:Ldose: -0.397940009 (uM) Interaction | 9.805402538 | 15.05685414 | 0.651225179 | 0.51490785 |
| MeWo:Gefitinib:Ldose: -0.397940009 (uM) Interaction | 6.240445497 | 15.33308304 | 0.406992219 | 0.68401761 |
| MeWo:Gemcitabine.HCl:Ldose: -0.397940009 (uM) Interaction | 32.36211988 | 15.13733352 | 2.13790096 | 0.032535623 |
| SKMEL2:Gemcitabine.HCl:Ldose: -0.397940009 (uM) Interaction | 75.27206909 | 15.31686613 | 4.914325715 | 8.97E-07 |
| UACC0257:Gemcitabine.HCl:Ldose: -0.397940009 (uM) Interaction | -15.77332841 | 15.05685414 | -1.047584593 | 0.294841395 |
| MeWo:Ibrutinib..PCI.32765.:Ldose: -0.397940009 (uM) Interaction | 4.011565574 | 15.07335079 | 0.266136284 | 0.790136712 |
| SKMEL2:Ibrutinib..PCI.32765.:Ldose: -0.397940009 (uM) Interaction | 7.945691515 | 15.25363648 | 0.520904738 | 0.602438308 |
| MeWo:Imiquimod:Ldose: -0.397940009 (uM) Interaction | -12.68418305 | 15.07335079 | -0.841497237 | 0.400078457 |
| SKMEL2:Imiquimod:Ldose: -0.397940009 (uM) Interaction | -6.385326481 | 15.25363648 | -0.418610113 | 0.67550513 |
| MeWo:INK.128..MLN0128.:Ldose: -0.397940009 (uM) Interaction | 28.83783901 | 15.13733352 | 1.905080506 | 0.05678246 |
| SKMEL2:INK.128..MLN0128.:Ldose: -0.397940009 (uM) Interaction | 12.03539339 | 15.31686613 | 0.785760827 | 0.43201597 |
| UACC0257:INK.128..MLN0128.:Ldose: -0.397940009 (uM) Interaction | 13.74449741 | 15.05685414 | 0.912839912 | 0.361336614 |
| MeWo:Irinotecan.HCl:Ldose: -0.397940009 (uM) Interaction | 11.6541139 | 15.13733352 | 0.769892127 | 0.441372041 |
| SKMEL2:Irinotecan.HCl:Ldose: -0.397940009 (uM) Interaction | 11.0211879 | 15.31686613 | 0.719545879 | 0.471812187 |
| UACC0257:Irinotecan.HCl:Ldose: -0.397940009 (uM) Interaction | 10.46256672 | 15.05685414 | 0.694870696 | 0.487143579 |
| MeWo:Ixabepilone:Ldose: -0.397940009 (uM) Interaction | 19.65192267 | 15.13733352 | 1.298242034 | 0.194217607 |
| SKMEL2:Ixabepilone:Ldose: -0.397940009 (uM) Interaction | 26.10068471 | 15.31686613 | 1.704048628 | 0.088385937 |
| UACC0257:Ixabepilone:Ldose: -0.397940009 (uM) Interaction | 11.73949136 | 15.05685414 | 0.779677564 | 0.435588964 |
| MeWo:Lapatinib:Ldose: -0.397940009 (uM) Interaction | -12.19985078 | 15.07335079 | -0.809365545 | 0.418313552 |
| SKMEL2:Lapatinib:Ldose: -0.397940009 (uM) Interaction | -10.65808266 | 15.25363648 | -0.69872405 | 0.484731789 |
| MeWo:LDK378:Ldose: -0.397940009 (uM) Interaction | 12.84747287 | 15.13733352 | 0.848727608 | 0.396041992 |
| SKMEL2:LDK378:Ldose: -0.397940009 (uM) Interaction | 16.46154844 | 15.31686613 | 1.074733454 | 0.282505712 |
| UACC0257:LDK378:Ldose: -0.397940009 (uM) Interaction | -5.207626819 | 15.05685414 | -0.3458642 | 0.729448037 |
| MeWo:Lenalidomide:Ldose: -0.397940009 (uM) Interaction | -4.991886282 | 15.13733352 | -0.329773158 | 0.741574469 |
| SKMEL2:Lenalidomide:Ldose: -0.397940009 (uM) Interaction | -6.603225297 | 15.31686613 | -0.431108116 | 0.666393928 |
| SKMEL2:Letrozole:Ldose: -0.397940009 (uM) Interaction | 6.592360301 | 15.31686613 | 0.430398767 | 0.666909752 |
| MeWo:Linsitinib:Ldose: -0.397940009 (uM) Interaction | 1.49848637 | 15.13733352 | 0.098992756 | 0.921144911 |
| SKMEL2:Linsitinib:Ldose: -0.397940009 (uM) Interaction | 8.94330427 | 15.31686613 | 0.583886037 | 0.559302894 |
| UACC0257:Linsitinib:Ldose: -0.397940009 (uM) Interaction | -15.53536141 | 15.05685414 | -1.03178003 | 0.302186368 |
| SKMEL2:Lomustine..CCNU.:Ldose: -0.397940009 (uM) Interaction | 3.055600018 | 15.31686613 | 0.199492507 | 0.841879324 |
| MeWo:LY2157299:Ldose: -0.397940009 (uM) Interaction | 3.970242525 | 15.13733352 | 0.262281499 | 0.793106848 |
| SKMEL2:LY2157299:Ldose: -0.397940009 (uM) Interaction | 4.944364286 | 15.31686613 | 0.322805216 | 0.746845789 |
| MeWo:Mechlorethamine.HCl:Ldose: -0.397940009 (uM) Interaction | -5.859458323 | 15.13733352 | -0.387086557 | 0.698695824 |
| SKMEL2:Mechlorethamine.HCl:Ldose: -0.397940009 (uM) Interaction | -6.078199999 | 15.31686613 | -0.396830523 | 0.691496239 |
| UACC0257:Mechlorethamine.HCl:Ldose: -0.397940009 (uM) Interaction | -2.23227075 | 15.05685414 | -0.148256118 | 0.882141986 |
| MeWo:Megestrol.acetate:Ldose: -0.397940009 (uM) Interaction | 4.659295257 | 15.13733352 | 0.307801585 | 0.75823619 |
| SKMEL2:Megestrol.acetate:Ldose: -0.397940009 (uM) Interaction | 5.517780561 | 15.31686613 | 0.360242135 | 0.71866948 |
| MeWo:MEK.162..ARRY.438162.:Ldose: -0.397940009 (uM) Interaction | 28.9756482 | 15.13733352 | 1.914184434 | 0.055609381 |
| SKMEL2:MEK.162..ARRY.438162.:Ldose: -0.397940009 (uM) Interaction | 47.01252963 | 15.31686613 | 3.069330844 | 0.002147965 |
| UACC0257:MEK.162..ARRY.438162.:Ldose: -0.397940009 (uM) Interaction | 34.00905585 | 15.05685414 | 2.258709259 | 0.023911096 |
| MeWo:Melphalan:Ldose: -0.397940009 (uM) Interaction | 6.904547425 | 15.13733352 | 0.456127059 | 0.648303056 |
| SKMEL2:Melphalan:Ldose: -0.397940009 (uM) Interaction | 7.245944891 | 15.31686613 | 0.473069676 | 0.636168102 |
| UACC0257:Melphalan:Ldose: -0.397940009 (uM) Interaction | 2.893999046 | 15.05685414 | 0.192204761 | 0.847583567 |
| MeWo:Mercaptopurine:Ldose: -0.397940009 (uM) Interaction | -4.490942945 | 15.13733352 | -0.296679923 | 0.76671365 |
| SKMEL2:Mercaptopurine:Ldose: -0.397940009 (uM) Interaction | 0.634669701 | 15.31686613 | 0.041436002 | 0.966948683 |
| UACC0257:Mercaptopurine:Ldose: -0.397940009 (uM) Interaction | 16.77358525 | 15.05685414 | 1.114016586 | 0.26528408 |
| MeWo:Mitomycin.C:Ldose: -0.397940009 (uM) Interaction | -1.914652218 | 15.13733352 | -0.126485435 | 0.899348818 |
| SKMEL2:Mitomycin.C:Ldose: -0.397940009 (uM) Interaction | -5.251008545 | 15.31686613 | -0.342825256 | 0.731733157 |
| UACC0257:Mitomycin.C:Ldose: -0.397940009 (uM) Interaction | -17.20019557 | 15.05685414 | -1.142349884 | 0.253320881 |
| MeWo:Mitotane..o.p..DDD..Lysodren.:Ldose: -0.397940009 (uM) Interaction | 8.122540347 | 15.13733352 | 0.536589904 | 0.591556273 |
| SKMEL2:Mitotane..o.p..DDD..Lysodren.:Ldose: -0.397940009 (uM) Interaction | -6.882724235 | 15.31686613 | -0.449355905 | 0.653179303 |
| MeWo:Mitoxantrone:Ldose: -0.397940009 (uM) Interaction | 44.65627115 | 15.13733352 | 2.950075129 | 0.003180268 |
| SKMEL2:Mitoxantrone:Ldose: -0.397940009 (uM) Interaction | 66.24777603 | 15.31686613 | 4.325152122 | 1.53E-05 |
| UACC0257:Mitoxantrone:Ldose: -0.397940009 (uM) Interaction | 42.11974024 | 15.05685414 | 2.797379841 | 0.005156308 |
| UACC0257:MLN.2480:Ldose: -0.397940009 (uM) Interaction | -11.28591205 | 15.05685414 | -0.749553124 | 0.453531801 |
| MeWo:MLN4924:Ldose: -0.397940009 (uM) Interaction | 7.193194848 | 15.13733352 | 0.475195637 | 0.634652191 |
| SKMEL2:MLN4924:Ldose: -0.397940009 (uM) Interaction | 38.46508154 | 15.31686613 | 2.511289269 | 0.01203612 |
| UACC0257:MLN4924:Ldose: -0.397940009 (uM) Interaction | 38.13121012 | 15.05685414 | 2.532481869 | 0.011332624 |
| MeWo:MLN9708..MLN2238.:Ldose: -0.397940009 (uM) Interaction | 0.20303113 | 15.13733352 | 0.013412609 | 0.989298728 |
| SKMEL2:MLN9708..MLN2238.:Ldose: -0.397940009 (uM) Interaction | -7.238378407 | 15.31686613 | -0.472575679 | 0.636520563 |
| UACC0257:MLN9708..MLN2238.:Ldose: -0.397940009 (uM) Interaction | 0.763510426 | 15.05685414 | 0.050708496 | 0.959558261 |
| MeWo:Navitoclax..ABT.263..5uM:Ldose: -0.397940009 (uM) Interaction | -2.80520835 | 15.13733352 | -0.185317206 | 0.852981929 |
| SKMEL2:Navitoclax..ABT.263..5uM:Ldose: -0.397940009 (uM) Interaction | 4.913859323 | 15.31686613 | 0.320813623 | 0.748354641 |
| UACC0257:Navitoclax..ABT.263..5uM:Ldose: -0.397940009 (uM) Interaction | 10.55129441 | 15.05685414 | 0.70076354 | 0.483457908 |
| MeWo:Nelarabine:Ldose: -0.397940009 (uM) Interaction | -2.182964005 | 15.13733352 | -0.144210604 | 0.885335471 |
| SKMEL2:Nelarabine:Ldose: -0.397940009 (uM) Interaction | -7.568166814 | 15.31686613 | -0.494106742 | 0.621235665 |
| UACC0257:Nelarabine:Ldose: -0.397940009 (uM) Interaction | -0.207068839 | 15.05685414 | -0.013752464 | 0.989027591 |
| MeWo:OSI.027:Ldose: -0.397940009 (uM) Interaction | -19.29893106 | 15.13733352 | -1.274922761 | 0.202349867 |
| SKMEL2:OSI.027:Ldose: -0.397940009 (uM) Interaction | -12.45673976 | 15.31686613 | -0.81326948 | 0.416072255 |
| UACC0257:OSI.027:Ldose: -0.397940009 (uM) Interaction | -16.58442598 | 15.05685414 | -1.101453586 | 0.270711185 |
| MeWo:Oxaliplatin:Ldose: -0.397940009 (uM) Interaction | -3.95370788 | 15.07335079 | -0.262297875 | 0.793094224 |
| SKMEL2:Oxaliplatin:Ldose: -0.397940009 (uM) Interaction | 4.396197472 | 15.25363648 | 0.288206519 | 0.773191338 |
| MeWo:Paclitaxel:Ldose: -0.397940009 (uM) Interaction | 19.53506421 | 15.13733352 | 1.290522151 | 0.196882809 |
| SKMEL2:Paclitaxel:Ldose: -0.397940009 (uM) Interaction | 12.61223189 | 15.31686613 | 0.823421174 | 0.410277333 |
| MeWo:Palbociclib..PD.0332991..Isethionate:Ldose: -0.397940009 (uM) Interaction | 10.89421864 | 15.13733352 | 0.719692053 | 0.471722164 |
| SKMEL2:Palbociclib..PD.0332991..Isethionate:Ldose: -0.397940009 (uM) Interaction | 15.5676899 | 15.31686613 | 1.016375659 | 0.309461561 |
| UACC0257:Palbociclib..PD.0332991..Isethionate:Ldose: -0.397940009 (uM) Interaction | 5.432486125 | 15.05685414 | 0.360798217 | 0.718253714 |
| MeWo:Pazopanib.HCl:Ldose: -0.397940009 (uM) Interaction | 6.338014558 | 15.13733352 | 0.41870086 | 0.675438801 |
| SKMEL2:Pazopanib.HCl:Ldose: -0.397940009 (uM) Interaction | 5.423766273 | 15.31686613 | 0.354104177 | 0.72326416 |
| UACC0257:Pazopanib.HCl:Ldose: -0.397940009 (uM) Interaction | 14.33728395 | 15.05685414 | 0.952209792 | 0.341000918 |
| MeWo:PD325901:Ldose: -0.397940009 (uM) Interaction | -20.32686225 | 15.13733352 | -1.34282978 | 0.179340768 |
| SKMEL2:PD325901:Ldose: -0.397940009 (uM) Interaction | -23.25569928 | 15.31686613 | -1.518306623 | 0.128951298 |
| UACC0257:PD325901:Ldose: -0.397940009 (uM) Interaction | 4.708966496 | 15.05685414 | 0.312745707 | 0.754476792 |
| SKMEL2:Pemetrexed:Ldose: -0.397940009 (uM) Interaction | 7.815021098 | 15.31686613 | 0.510223242 | 0.609900122 |
| MeWo:Plicamycin:Ldose: -0.397940009 (uM) Interaction | 7.924111054 | 15.13733352 | 0.523481301 | 0.600644563 |
| SKMEL2:Plicamycin:Ldose: -0.397940009 (uM) Interaction | 23.22451141 | 15.31686613 | 1.516270444 | 0.129465159 |
| UACC0257:Plicamycin:Ldose: -0.397940009 (uM) Interaction | 12.21467654 | 15.05685414 | 0.811236957 | 0.417238266 |
| MeWo:Pralatrexate:Ldose: -0.397940009 (uM) Interaction | 9.81448162 | 15.07335079 | 0.651114789 | 0.514979101 |
| SKMEL2:Pralatrexate:Ldose: -0.397940009 (uM) Interaction | 9.280047334 | 15.25363648 | 0.608382621 | 0.542939933 |
| MeWo:Quinacrine.HCl:Ldose: -0.397940009 (uM) Interaction | -6.930472228 | 15.13733352 | -0.457839699 | 0.647072074 |
| SKMEL2:Quinacrine.HCl:Ldose: -0.397940009 (uM) Interaction | -6.416477232 | 15.31686613 | -0.4189158 | 0.675281705 |
| UACC0257:Quinacrine.HCl:Ldose: -0.397940009 (uM) Interaction | -0.110300865 | 15.05685414 | -0.007325625 | 0.994155115 |
| MeWo:Quizartinib:Ldose: -0.397940009 (uM) Interaction | 2.513871742 | 15.13733352 | 0.166070975 | 0.868102601 |
| SKMEL2:Quizartinib:Ldose: -0.397940009 (uM) Interaction | 4.716143731 | 15.31686613 | 0.307905265 | 0.758157295 |
| UACC0257:Quizartinib:Ldose: -0.397940009 (uM) Interaction | -10.78744416 | 15.05685414 | -0.716447411 | 0.473722633 |
| MeWo:Raloxifene:Ldose: -0.397940009 (uM) Interaction | 15.42892402 | 15.07335079 | 1.023589528 | 0.306040299 |
| SKMEL2:Raloxifene:Ldose: -0.397940009 (uM) Interaction | 12.4477767 | 15.25363648 | 0.816053059 | 0.414478508 |
| MeWo:Romidepsin:Ldose: -0.397940009 (uM) Interaction | 9.040534196 | 15.13733352 | 0.597234261 | 0.550357053 |
| SKMEL2:Romidepsin:Ldose: -0.397940009 (uM) Interaction | 8.063333361 | 15.31686613 | 0.526434931 | 0.598591288 |
| UACC0257:Romidepsin:Ldose: -0.397940009 (uM) Interaction | 2.261063686 | 15.05685414 | 0.150168399 | 0.880633115 |
| MeWo:Sabutoclax..BI.97C1.:Ldose: -0.397940009 (uM) Interaction | 12.51989015 | 15.13733352 | 0.827086893 | 0.408196664 |
| SKMEL2:Sabutoclax..BI.97C1.:Ldose: -0.397940009 (uM) Interaction | 27.9778501 | 15.31686613 | 1.826604076 | 0.067772684 |
| UACC0257:Sabutoclax..BI.97C1.:Ldose: -0.397940009 (uM) Interaction | 33.76296573 | 15.05685414 | 2.242365199 | 0.024947567 |
| MeWo:Sirolimus..Rapamycin.:Ldose: -0.397940009 (uM) Interaction | -5.068819853 | 15.13733352 | -0.334855531 | 0.737737219 |
| SKMEL2:Sirolimus..Rapamycin.:Ldose: -0.397940009 (uM) Interaction | -2.283145445 | 15.31686613 | -0.149060874 | 0.881506947 |
| MeWo:Sorafenib:Ldose: -0.397940009 (uM) Interaction | 7.55294957 | 15.13733352 | 0.498961693 | 0.617811293 |
| UACC0257:Sorafenib:Ldose: -0.397940009 (uM) Interaction | 3.930519536 | 15.05685414 | 0.261045202 | 0.79406006 |
| MeWo:Streptozocin:Ldose: -0.397940009 (uM) Interaction | 3.934470925 | 15.13733352 | 0.259918361 | 0.794929148 |
| SKMEL2:Streptozocin:Ldose: -0.397940009 (uM) Interaction | -6.511794724 | 15.31686613 | -0.425138842 | 0.670739567 |
| UACC0257:Streptozocin:Ldose: -0.397940009 (uM) Interaction | 8.79979274 | 15.05685414 | 0.584437669 | 0.558931803 |
| MeWo:Sunitinib:Ldose: -0.397940009 (uM) Interaction | -9.829437558 | 15.13733352 | -0.649350663 | 0.516118435 |
| UACC0257:Sunitinib:Ldose: -0.397940009 (uM) Interaction | 12.91453642 | 15.05685414 | 0.857718106 | 0.391057359 |
| MeWo:Tamoxifen.Citrate:Ldose: -0.397940009 (uM) Interaction | 3.951932192 | 15.13733352 | 0.261071885 | 0.794039484 |
| SKMEL2:Tamoxifen.Citrate:Ldose: -0.397940009 (uM) Interaction | 8.828054441 | 15.31686613 | 0.576361664 | 0.564376569 |
| UACC0257:Tamoxifen.Citrate:Ldose: -0.397940009 (uM) Interaction | 1.146857038 | 15.05685414 | 0.076168436 | 0.939285777 |
| MeWo:Temozolomide:Ldose: -0.397940009 (uM) Interaction | 1.330511402 | 15.33308304 | 0.086773899 | 0.930852014 |
| SKMEL2:Temsirolimus..CCI.779..Torisel.:Ldose: -0.397940009 (uM) Interaction | 9.855464863 | 15.31686613 | 0.643438728 | 0.519946078 |
| MeWo:Teniposide:Ldose: -0.397940009 (uM) Interaction | 22.8476893 | 15.13733352 | 1.509360236 | 0.131220919 |
| SKMEL2:Teniposide:Ldose: -0.397940009 (uM) Interaction | 36.79413844 | 15.31686613 | 2.40219756 | 0.01630503 |
| UACC0257:Teniposide:Ldose: -0.397940009 (uM) Interaction | 26.9037834 | 15.05685414 | 1.786813046 | 0.073981291 |
| MeWo:Thioguanine:Ldose: -0.397940009 (uM) Interaction | 10.72784442 | 15.13733352 | 0.708701067 | 0.478517412 |
| SKMEL2:Thioguanine:Ldose: -0.397940009 (uM) Interaction | 5.090164668 | 15.31686613 | 0.33232416 | 0.739647623 |
| UACC0257:Thioguanine:Ldose: -0.397940009 (uM) Interaction | 5.002687788 | 15.05685414 | 0.332253188 | 0.739701208 |
| MeWo:Thiotepa:Ldose: -0.397940009 (uM) Interaction | -7.08082009 | 15.13733352 | -0.467771955 | 0.63995225 |
| SKMEL2:Thiotepa:Ldose: -0.397940009 (uM) Interaction | -8.759319839 | 15.31686613 | -0.571874153 | 0.567413001 |
| UACC0257:Thiotepa:Ldose: -0.397940009 (uM) Interaction | 2.845674556 | 15.05685414 | 0.188995293 | 0.850098226 |
| MeWo:Topotecan.HCl:Ldose: -0.397940009 (uM) Interaction | 60.70836292 | 15.13733352 | 4.010505735 | 6.08E-05 |
| SKMEL2:Topotecan.HCl:Ldose: -0.397940009 (uM) Interaction | 55.9835225 | 15.31686613 | 3.6550246 | 0.000257748 |
| UACC0257:Topotecan.HCl:Ldose: -0.397940009 (uM) Interaction | 29.23909176 | 15.05685414 | 1.9419124 | 0.052160285 |
| MeWo:Trametinib..GSK1120212.:Ldose: -0.397940009 (uM) Interaction | -8.76535609 | 15.13733352 | -0.579055491 | 0.562557581 |
| UACC0257:Trametinib..GSK1120212.:Ldose: -0.397940009 (uM) Interaction | 8.90168293 | 15.05685414 | 0.5912047 | 0.554389268 |
| MeWo:Triethylenemelamine:Ldose: -0.397940009 (uM) Interaction | -2.160101908 | 15.13733352 | -0.142700292 | 0.886528176 |
| SKMEL2:Triethylenemelamine:Ldose: -0.397940009 (uM) Interaction | 0.38603868 | 15.31686613 | 0.025203503 | 0.979892868 |
| UACC0257:Triethylenemelamine:Ldose: -0.397940009 (uM) Interaction | -1.930381614 | 15.05685414 | -0.128206171 | 0.897986973 |
| MeWo:Uracil.mustard:Ldose: -0.397940009 (uM) Interaction | 8.591821092 | 15.13733352 | 0.56759145 | 0.570318129 |
| SKMEL2:Uracil.mustard:Ldose: -0.397940009 (uM) Interaction | 6.634439943 | 15.31686613 | 0.433146042 | 0.664912866 |
| MeWo:Valrubicin:Ldose: -0.397940009 (uM) Interaction | 26.13067474 | 15.13733352 | 1.726240272 | 0.084318049 |
| SKMEL2:Valrubicin:Ldose: -0.397940009 (uM) Interaction | 8.13227965 | 15.31686613 | 0.530936262 | 0.595468237 |
| UACC0257:Valrubicin:Ldose: -0.397940009 (uM) Interaction | 22.52359704 | 15.05685414 | 1.49590325 | 0.134693023 |
| MeWo:Vandetanib:Ldose: -0.397940009 (uM) Interaction | -5.22996908 | 15.07335079 | -0.346967914 | 0.728618699 |
| SKMEL2:Vandetanib:Ldose: -0.397940009 (uM) Interaction | 1.049249819 | 15.25363648 | 0.068786864 | 0.945159891 |
| SKMEL2:Vemurafenib:Ldose: -0.397940009 (uM) Interaction | 53.21462174 | 15.31686613 | 3.474249974 | 0.000513256 |
| UACC0257:Vemurafenib:Ldose: -0.397940009 (uM) Interaction | 31.45574373 | 15.05685414 | 2.089131198 | 0.036707224 |
| SKMEL2:Vinblastine.Sulfate:Ldose: -0.397940009 (uM) Interaction | 12.63774015 | 15.31686613 | 0.825086545 | 0.409331286 |
| UACC0257:Vinblastine.Sulfate:Ldose: -0.397940009 (uM) Interaction | 31.75761527 | 15.05685414 | 2.109179977 | 0.034940168 |
| MeWo:Vincristine.Sulfate:Ldose: -0.397940009 (uM) Interaction | 31.23016077 | 15.13733352 | 2.063121667 | 0.039112621 |
| SKMEL2:Vincristine.Sulfate:Ldose: -0.397940009 (uM) Interaction | 52.71187844 | 15.31686613 | 3.441427117 | 0.000579715 |
| UACC0257:Vincristine.Sulfate:Ldose: -0.397940009 (uM) Interaction | 64.59341788 | 15.05685414 | 4.289967697 | 1.79E-05 |
| MeWo:Vinorelbine.Tartrate:Ldose: -0.397940009 (uM) Interaction | -27.91392756 | 15.13733352 | -1.844045222 | 0.065189809 |
| SKMEL2:Vinorelbine.Tartrate:Ldose: -0.397940009 (uM) Interaction | -18.56126785 | 15.31686613 | -1.211818899 | 0.225594554 |
| UACC0257:Vinorelbine.Tartrate:Ldose: -0.397940009 (uM) Interaction | -19.74009304 | 15.05685414 | -1.31103701 | 0.189858762 |
| MeWo:Vismodegib:Ldose: -0.397940009 (uM) Interaction | 5.647897958 | 15.13733352 | 0.373110492 | 0.709069757 |
| SKMEL2:Vismodegib:Ldose: -0.397940009 (uM) Interaction | -4.834009264 | 15.31686613 | -0.315600412 | 0.752308779 |
| UACC0257:Vismodegib:Ldose: -0.397940009 (uM) Interaction | 3.100339508 | 15.05685414 | 0.205908849 | 0.836863993 |
| MeWo:Vorinostat:Ldose: -0.397940009 (uM) Interaction | -4.049393002 | 15.13733352 | -0.267510324 | 0.78907874 |
| SKMEL2:Vorinostat:Ldose: -0.397940009 (uM) Interaction | -3.260559504 | 15.31686613 | -0.2128738 | 0.831427344 |
| UACC0257:Vorinostat:Ldose: -0.397940009 (uM) Interaction | 11.99162248 | 15.05685414 | 0.796422837 | 0.425794789 |
| MeWo:Zoledronic.Acid:Ldose: -0.397940009 (uM) Interaction | 11.5820041 | 15.33308304 | 0.755360424 | 0.450040732 |
| MeWo:Abiraterone:Ldose: 0 (uM) Interaction | -1.529473142 | 15.13733352 | -0.101039799 | 0.919519775 |
| SKMEL2:Abiraterone:Ldose: 0 (uM) Interaction | -3.588180017 | 15.31686613 | -0.234263327 | 0.814782706 |
| MeWo:ABT.737:Ldose: 0 (uM) Interaction | -20.93964756 | 15.13733352 | -1.383311501 | 0.166583224 |
| SKMEL2:ABT.737:Ldose: 0 (uM) Interaction | -16.78121013 | 15.31686613 | -1.095603369 | 0.273264221 |
| UACC0257:ABT.737:Ldose: 0 (uM) Interaction | -9.943898575 | 15.05685414 | -0.660423385 | 0.508988992 |
| MeWo:Actinomycin.D:Ldose: 0 (uM) Interaction | -29.90292613 | 15.13733352 | -1.975442113 | 0.04823031 |
| SKMEL2:Actinomycin.D:Ldose: 0 (uM) Interaction | -31.38970196 | 15.31686613 | -2.049355377 | 0.040439053 |
| UACC0257:Actinomycin.D:Ldose: 0 (uM) Interaction | -15.75187647 | 15.05685414 | -1.046159864 | 0.295498571 |
| MeWo:Afatinib:Ldose: 0 (uM) Interaction | 6.477454083 | 15.13733352 | 0.42791249 | 0.668718966 |
| SKMEL2:Afatinib:Ldose: 0 (uM) Interaction | -2.470403884 | 15.31686613 | -0.16128651 | 0.871869209 |
| UACC0257:Afatinib:Ldose: 0 (uM) Interaction | 6.878228315 | 15.05685414 | 0.456817092 | 0.64780697 |
| MeWo:Alisertib..MLN8237.:Ldose: 0 (uM) Interaction | 28.53644395 | 15.13733352 | 1.885169796 | 0.059419909 |
| SKMEL2:Alisertib..MLN8237.:Ldose: 0 (uM) Interaction | 37.64660049 | 15.31686613 | 2.457852682 | 0.013984602 |
| MeWo:Allopurinol:Ldose: 0 (uM) Interaction | -1.598688192 | 15.13733352 | -0.105612272 | 0.915890937 |
| SKMEL2:Allopurinol:Ldose: 0 (uM) Interaction | -7.905429744 | 15.31686613 | -0.516125797 | 0.60577167 |
| UACC0257:Allopurinol:Ldose: 0 (uM) Interaction | 1.925160867 | 15.05685414 | 0.127859435 | 0.898261367 |
| MeWo:Amifostine:Ldose: 0 (uM) Interaction | -6.181676358 | 15.13733352 | -0.408372872 | 0.683003869 |
| MeWo:Aphrocallistin.analogue:Ldose: 0 (uM) Interaction | 22.27672828 | 15.13733352 | 1.471641505 | 0.141131825 |
| SKMEL2:Aphrocallistin.analogue:Ldose: 0 (uM) Interaction | 64.01425896 | 15.31686613 | 4.179331361 | 2.93E-05 |
| UACC0257:Aphrocallistin.analogue:Ldose: 0 (uM) Interaction | 37.08314415 | 15.05685414 | 2.462874635 | 0.013790311 |
| MeWo:Arsenic.Trioxide:Ldose: 0 (uM) Interaction | 9.768890942 | 15.13733352 | 0.645350842 | 0.518706493 |
| SKMEL2:Arsenic.Trioxide:Ldose: 0 (uM) Interaction | 4.205652369 | 15.31686613 | 0.274576557 | 0.783644123 |
| MeWo:Axitinib:Ldose: 0 (uM) Interaction | 4.792445968 | 15.13733352 | 0.316597765 | 0.751551796 |
| SKMEL2:Axitinib:Ldose: 0 (uM) Interaction | 5.958765678 | 15.31686613 | 0.389032954 | 0.697255484 |
| UACC0257:Axitinib:Ldose: 0 (uM) Interaction | -16.27856266 | 15.05685414 | -1.081139693 | 0.279646673 |
| MeWo:Axitinib.1:Ldose: 0 (uM) Interaction | 7.463266125 | 15.13733352 | 0.493037041 | 0.621991271 |
| SKMEL2:Axitinib.1:Ldose: 0 (uM) Interaction | 12.78358513 | 15.31686613 | 0.8346084 | 0.403947173 |
| UACC0257:Axitinib.1:Ldose: 0 (uM) Interaction | 2.068537059 | 15.05685414 | 0.137381756 | 0.890730292 |
| MeWo:Azacitidine:Ldose: 0 (uM) Interaction | 4.889012052 | 15.13733352 | 0.322977098 | 0.746715615 |
| SKMEL2:Azacitidine:Ldose: 0 (uM) Interaction | -10.01825004 | 15.31686613 | -0.654066567 | 0.513075666 |
| UACC0257:Azacitidine:Ldose: 0 (uM) Interaction | 7.308274787 | 15.05685414 | 0.4853786 | 0.627412579 |
| SKMEL2:Baricitinib..LY3009104..INCB028050.:Ldose: 0 (uM) Interaction | 13.43029051 | 15.31686613 | 0.876830182 | 0.38058833 |
| UACC0257:Baricitinib..LY3009104..INCB028050.:Ldose: 0 (uM) Interaction | 7.762534275 | 15.05685414 | 0.515548215 | 0.606175098 |
| SKMEL2:Bendamustine.HCl:Ldose: 0 (uM) Interaction | 15.6776995 | 15.25363648 | 1.027800782 | 0.304054698 |
| MeWo:Bioymifi:Ldose: 0 (uM) Interaction | -0.974443702 | 15.13733352 | -0.064373537 | 0.948673378 |
| SKMEL2:Bioymifi:Ldose: 0 (uM) Interaction | -4.526895852 | 15.31686613 | -0.295549743 | 0.767576711 |
| UACC0257:Bioymifi:Ldose: 0 (uM) Interaction | -9.767323735 | 15.05685414 | -0.648696178 | 0.516541456 |
| MeWo:Bleomycin.Sulfate:Ldose: 0 (uM) Interaction | 22.72765387 | 15.13733352 | 1.501430475 | 0.133258418 |
| SKMEL2:Bleomycin.Sulfate:Ldose: 0 (uM) Interaction | 23.21238478 | 15.31686613 | 1.515478727 | 0.12966539 |
| UACC0257:Bleomycin.Sulfate:Ldose: 0 (uM) Interaction | 40.18935429 | 15.05685414 | 2.669173383 | 0.007609325 |
| MeWo:Bortezomib:Ldose: 0 (uM) Interaction | 7.846115392 | 15.13733352 | 0.518328765 | 0.60423405 |
| SKMEL2:Bortezomib:Ldose: 0 (uM) Interaction | 17.07468492 | 15.31686613 | 1.114763606 | 0.264963752 |
| UACC0257:Bortezomib:Ldose: 0 (uM) Interaction | 23.92512466 | 15.05685414 | 1.588985617 | 0.112077819 |
| MeWo:Bosutinib..SKI.606.:Ldose: 0 (uM) Interaction | 19.58041262 | 15.13733352 | 1.293517949 | 0.195845381 |
| SKMEL2:Bosutinib..SKI.606.:Ldose: 0 (uM) Interaction | 25.36701948 | 15.31686613 | 1.656149454 | 0.097705628 |
| UACC0257:Bosutinib..SKI.606.:Ldose: 0 (uM) Interaction | 12.17220946 | 15.05685414 | 0.808416509 | 0.418859477 |
| SKMEL2:Busulfan:Ldose: 0 (uM) Interaction | 10.90300057 | 15.31686613 | 0.711829723 | 0.476577672 |
| MeWo:Cabazitaxel:Ldose: 0 (uM) Interaction | 6.117935583 | 15.13733352 | 0.404162039 | 0.686097443 |
| SKMEL2:Cabazitaxel:Ldose: 0 (uM) Interaction | 4.929358119 | 15.31686613 | 0.321825501 | 0.747587911 |
| UACC0257:Cabazitaxel:Ldose: 0 (uM) Interaction | -7.667249601 | 15.05685414 | -0.50921989 | 0.610603143 |
| MeWo:Cabozantinib..XL.184.:Ldose: 0 (uM) Interaction | -7.499258268 | 15.13733352 | -0.495414748 | 0.620312269 |
| SKMEL2:Cabozantinib..XL.184.:Ldose: 0 (uM) Interaction | 10.14663991 | 15.31686613 | 0.662448821 | 0.507690471 |
| UACC0257:Cabozantinib..XL.184.:Ldose: 0 (uM) Interaction | -3.669646105 | 15.05685414 | -0.24371931 | 0.807450459 |
| MeWo:Capecitabine:Ldose: 0 (uM) Interaction | 1.280689209 | 15.33308304 | 0.083524573 | 0.933435189 |
| MeWo:Carfilzomib:Ldose: 0 (uM) Interaction | -34.84904573 | 15.13733352 | -2.302191841 | 0.021333524 |
| SKMEL2:Carfilzomib:Ldose: 0 (uM) Interaction | 31.05313799 | 15.31686613 | 2.027381954 | 0.042635238 |
| UACC0257:Carfilzomib:Ldose: 0 (uM) Interaction | -35.29912305 | 15.05685414 | -2.344388989 | 0.019067011 |
| MeWo:Carmustine:Ldose: 0 (uM) Interaction | -0.963943688 | 15.33308304 | -0.062866919 | 0.949873041 |
| MeWo:Celecoxib:Ldose: 0 (uM) Interaction | 0.171988041 | 15.33308304 | 0.011216794 | 0.991050581 |
| UACC0257:Chlorambucil:Ldose: 0 (uM) Interaction | -9.107861658 | 15.05685414 | -0.604898047 | 0.545252897 |
| MeWo:Cisplatin:Ldose: 0 (uM) Interaction | 9.437297659 | 15.13733352 | 0.623445182 | 0.532998392 |
| SKMEL2:Cisplatin:Ldose: 0 (uM) Interaction | 6.474071414 | 15.31686613 | 0.422675981 | 0.672535758 |
| MeWo:Cladribine:Ldose: 0 (uM) Interaction | 58.1045751 | 15.13733352 | 3.838494739 | 0.000124133 |
| SKMEL2:Cladribine:Ldose: 0 (uM) Interaction | 53.08844512 | 15.31686613 | 3.466012217 | 0.000529234 |
| UACC0257:Cladribine:Ldose: 0 (uM) Interaction | 38.3910806 | 15.05685414 | 2.549741151 | 0.010786924 |
| MeWo:Clofarabine:Ldose: 0 (uM) Interaction | 49.33590605 | 15.13733352 | 3.259220389 | 0.001118862 |
| SKMEL2:Clofarabine:Ldose: 0 (uM) Interaction | 31.931787 | 15.31686613 | 2.084746758 | 0.037103644 |
| UACC0257:Clofarabine:Ldose: 0 (uM) Interaction | 0.363643737 | 15.05685414 | 0.024151375 | 0.98073208 |
| MeWo:Crizotinib:Ldose: 0 (uM) Interaction | 3.868369545 | 15.13733352 | 0.255551583 | 0.798299466 |
| SKMEL2:Crizotinib:Ldose: 0 (uM) Interaction | 3.440248331 | 15.31686613 | 0.224605236 | 0.822288449 |
| UACC0257:Crizotinib:Ldose: 0 (uM) Interaction | -17.37003905 | 15.05685414 | -1.153630028 | 0.248664249 |
| MeWo:Cytarabine.HCl...Ara.C:Ldose: 0 (uM) Interaction | 60.9301585 | 15.13733352 | 4.025157958 | 5.71E-05 |
| SKMEL2:Cytarabine.HCl...Ara.C:Ldose: 0 (uM) Interaction | 56.71973871 | 15.31686613 | 3.703090321 | 0.000213503 |
| UACC0257:Cytarabine.HCl...Ara.C:Ldose: 0 (uM) Interaction | 37.35701891 | 15.05685414 | 2.48106401 | 0.013106401 |
| MeWo:Dacarbazine:Ldose: 0 (uM) Interaction | 4.773200323 | 15.13733352 | 0.315326363 | 0.752516822 |
| SKMEL2:Dacarbazine:Ldose: 0 (uM) Interaction | -5.305139157 | 15.31686613 | -0.346359308 | 0.72907597 |
| MeWo:Dacomitinib..PF299804.:Ldose: 0 (uM) Interaction | -6.13720051 | 15.13733352 | -0.405434715 | 0.685161888 |
| SKMEL2:Dacomitinib..PF299804.:Ldose: 0 (uM) Interaction | -2.629619719 | 15.31686613 | -0.171681315 | 0.863689634 |
| UACC0257:Dacomitinib..PF299804.:Ldose: 0 (uM) Interaction | 11.93772112 | 15.05685414 | 0.792842981 | 0.427877747 |
| MeWo:Dasatinib:Ldose: 0 (uM) Interaction | -3.883026952 | 15.13733352 | -0.256519878 | 0.7975518 |
| SKMEL2:Dasatinib:Ldose: 0 (uM) Interaction | -13.92610577 | 15.31686613 | -0.909200724 | 0.36325401 |
| UACC0257:Dasatinib:Ldose: 0 (uM) Interaction | 14.99899465 | 15.05685414 | 0.996157266 | 0.31918455 |
| MeWo:Daunorubicin.HCl:Ldose: 0 (uM) Interaction | 15.02588839 | 15.13733352 | 0.99263773 | 0.320897303 |
| SKMEL2:Daunorubicin.HCl:Ldose: 0 (uM) Interaction | 39.76373541 | 15.31686613 | 2.596075142 | 0.009435728 |
| UACC0257:Daunorubicin.HCl:Ldose: 0 (uM) Interaction | -4.574775408 | 15.05685414 | -0.303833415 | 0.761257643 |
| MeWo:Decitabine:Ldose: 0 (uM) Interaction | 1.132784948 | 15.13733352 | 0.07483385 | 0.94034758 |
| SKMEL2:Decitabine:Ldose: 0 (uM) Interaction | 2.650289693 | 15.31686613 | 0.173030806 | 0.862628784 |
| MeWo:Docetaxel:Ldose: 0 (uM) Interaction | 19.06413886 | 15.13733352 | 1.259411958 | 0.207894756 |
| MeWo:Doxorubicin.HCl:Ldose: 0 (uM) Interaction | 42.24087389 | 15.13733352 | 2.790509558 | 0.005266985 |
| SKMEL2:Doxorubicin.HCl:Ldose: 0 (uM) Interaction | 35.14688476 | 15.31686613 | 2.294652474 | 0.021762295 |
| UACC0257:Doxorubicin.HCl:Ldose: 0 (uM) Interaction | 26.65792428 | 15.05685414 | 1.770484328 | 0.076660155 |
| MeWo:Erlotinib.HCl:Ldose: 0 (uM) Interaction | -24.22801248 | 15.13733352 | -1.600546916 | 0.109491476 |
| SKMEL2:Erlotinib.HCl:Ldose: 0 (uM) Interaction | -16.8299996 | 15.31686613 | -1.098788711 | 0.271872102 |
| UACC0257:Erlotinib.HCl:Ldose: 0 (uM) Interaction | -16.58106887 | 15.05685414 | -1.101230623 | 0.270808185 |
| MeWo:Etoposide:Ldose: 0 (uM) Interaction | 21.27126954 | 15.13733352 | 1.405219057 | 0.159970082 |
| SKMEL2:Etoposide:Ldose: 0 (uM) Interaction | 26.47379253 | 15.31686613 | 1.728407907 | 0.083928964 |
| UACC0257:Etoposide:Ldose: 0 (uM) Interaction | 18.96939101 | 15.05685414 | 1.259850885 | 0.207736346 |
| MeWo:Everolimus:Ldose: 0 (uM) Interaction | -6.429172761 | 15.33308304 | -0.41930072 | 0.67500041 |
| MeWo:Exemestane:Ldose: 0 (uM) Interaction | 10.6714155 | 15.07335079 | 0.70796571 | 0.478973952 |
| SKMEL2:Exemestane:Ldose: 0 (uM) Interaction | 12.67308548 | 15.25363648 | 0.830823882 | 0.406082025 |
| MeWo:Floxuridine:Ldose: 0 (uM) Interaction | 15.77212381 | 15.13733352 | 1.04193541 | 0.297452923 |
| SKMEL2:Floxuridine:Ldose: 0 (uM) Interaction | 56.96382674 | 15.31686613 | 3.71902622 | 0.000200482 |
| UACC0257:Floxuridine:Ldose: 0 (uM) Interaction | 3.628765319 | 15.05685414 | 0.241004215 | 0.809554065 |
| MeWo:Fludarabine.Phosphate:Ldose: 0 (uM) Interaction | -5.402029863 | 15.13733352 | -0.356867995 | 0.721194008 |
| SKMEL2:Fludarabine.Phosphate:Ldose: 0 (uM) Interaction | -0.089793747 | 15.31686613 | -0.00586241 | 0.995322553 |
| MeWo:Fluorouracil...5.FU.:Ldose: 0 (uM) Interaction | -3.691154177 | 15.13733352 | -0.243844411 | 0.807353567 |
| SKMEL2:Fluorouracil...5.FU.:Ldose: 0 (uM) Interaction | -5.329756251 | 15.31686613 | -0.347966497 | 0.72786863 |
| UACC0257:Fluorouracil...5.FU.:Ldose: 0 (uM) Interaction | -4.870642977 | 15.05685414 | -0.32348344 | 0.746332181 |
| MeWo:Flutamide..Eulexin.:Ldose: 0 (uM) Interaction | 0.210435908 | 15.13733352 | 0.013901782 | 0.988908465 |
| SKMEL2:Flutamide..Eulexin.:Ldose: 0 (uM) Interaction | 3.924232233 | 15.31686613 | 0.256203338 | 0.797796195 |
| UACC0257:Flutamide..Eulexin.:Ldose: 0 (uM) Interaction | -19.85974122 | 15.05685414 | -1.318983437 | 0.187188196 |
| MeWo:Foretinib..GSK1363089.:Ldose: 0 (uM) Interaction | -0.441239402 | 15.13733352 | -0.029149084 | 0.97674595 |
| SKMEL2:Foretinib..GSK1363089.:Ldose: 0 (uM) Interaction | 34.36751148 | 15.31686613 | 2.243769136 | 0.024857033 |
| UACC0257:Foretinib..GSK1363089.:Ldose: 0 (uM) Interaction | 0.98872235 | 15.05685414 | 0.065665931 | 0.947644385 |
| SKMEL2:Fulvestrant:Ldose: 0 (uM) Interaction | -6.708390126 | 15.31686613 | -0.437974065 | 0.661409327 |
| UACC0257:Fulvestrant:Ldose: 0 (uM) Interaction | 15.93375756 | 15.05685414 | 1.058239484 | 0.289957739 |
| MeWo:Gefitinib:Ldose: 0 (uM) Interaction | 8.740870161 | 15.33308304 | 0.570066055 | 0.568638641 |
| MeWo:Gemcitabine.HCl:Ldose: 0 (uM) Interaction | 29.90256259 | 15.13733352 | 1.975418097 | 0.048233033 |
| SKMEL2:Gemcitabine.HCl:Ldose: 0 (uM) Interaction | 67.9315517 | 15.31686613 | 4.435081637 | 9.25E-06 |
| UACC0257:Gemcitabine.HCl:Ldose: 0 (uM) Interaction | -13.88151963 | 15.05685414 | -0.921940234 | 0.356569727 |
| MeWo:Ibrutinib..PCI.32765.:Ldose: 0 (uM) Interaction | -2.441418133 | 15.07335079 | -0.161969171 | 0.871331599 |
| SKMEL2:Ibrutinib..PCI.32765.:Ldose: 0 (uM) Interaction | 2.125905059 | 15.25363648 | 0.139370377 | 0.889158739 |
| MeWo:Imiquimod:Ldose: 0 (uM) Interaction | -21.29584627 | 15.07335079 | -1.412814348 | 0.157724277 |
| SKMEL2:Imiquimod:Ldose: 0 (uM) Interaction | -22.38898618 | 15.25363648 | -1.467780238 | 0.142178033 |
| MeWo:INK.128..MLN0128.:Ldose: 0 (uM) Interaction | 25.86590005 | 15.13733352 | 1.70874877 | 0.087511414 |
| SKMEL2:INK.128..MLN0128.:Ldose: 0 (uM) Interaction | 2.497539743 | 15.31686613 | 0.163058143 | 0.870474134 |
| UACC0257:INK.128..MLN0128.:Ldose: 0 (uM) Interaction | 10.76898819 | 15.05685414 | 0.715221658 | 0.474479577 |
| MeWo:Irinotecan.HCl:Ldose: 0 (uM) Interaction | 22.62653655 | 15.13733352 | 1.49475048 | 0.134993727 |
| SKMEL2:Irinotecan.HCl:Ldose: 0 (uM) Interaction | 15.58948228 | 15.31686613 | 1.017798429 | 0.308784803 |
| UACC0257:Irinotecan.HCl:Ldose: 0 (uM) Interaction | 20.56184818 | 15.05685414 | 1.365613826 | 0.172073981 |
| MeWo:Ixabepilone:Ldose: 0 (uM) Interaction | 24.61324239 | 15.13733352 | 1.625995909 | 0.103964658 |
| SKMEL2:Ixabepilone:Ldose: 0 (uM) Interaction | 22.26453006 | 15.31686613 | 1.453595656 | 0.146072517 |
| UACC0257:Ixabepilone:Ldose: 0 (uM) Interaction | 20.6689398 | 15.05685414 | 1.372726309 | 0.169851254 |
| MeWo:Lapatinib:Ldose: 0 (uM) Interaction | -10.05732008 | 15.07335079 | -0.667225239 | 0.504635177 |
| SKMEL2:Lapatinib:Ldose: 0 (uM) Interaction | -15.88868049 | 15.25363648 | -1.041632303 | 0.297593481 |
| MeWo:LDK378:Ldose: 0 (uM) Interaction | 6.9346765 | 15.13733352 | 0.458117441 | 0.646872534 |
| SKMEL2:LDK378:Ldose: 0 (uM) Interaction | 13.54019308 | 15.31686613 | 0.884005446 | 0.376702805 |
| UACC0257:LDK378:Ldose: 0 (uM) Interaction | -3.18909242 | 15.05685414 | -0.211803368 | 0.832262377 |
| MeWo:Lenalidomide:Ldose: 0 (uM) Interaction | -0.833724882 | 15.13733352 | -0.055077394 | 0.956077299 |
| SKMEL2:Lenalidomide:Ldose: 0 (uM) Interaction | -7.353014882 | 15.31686613 | -0.480060008 | 0.631189433 |
| SKMEL2:Letrozole:Ldose: 0 (uM) Interaction | 13.50957852 | 15.31686613 | 0.882006698 | 0.377782693 |
| MeWo:Linsitinib:Ldose: 0 (uM) Interaction | 14.4140214 | 15.13733352 | 0.952216675 | 0.340997428 |
| SKMEL2:Linsitinib:Ldose: 0 (uM) Interaction | 5.761576393 | 15.31686613 | 0.376158957 | 0.706802305 |
| UACC0257:Linsitinib:Ldose: 0 (uM) Interaction | -11.05477153 | 15.05685414 | -0.734201941 | 0.462833392 |
| SKMEL2:Lomustine..CCNU.:Ldose: 0 (uM) Interaction | -3.881245989 | 15.31686613 | -0.253396874 | 0.79996388 |
| MeWo:LY2157299:Ldose: 0 (uM) Interaction | -1.637801658 | 15.13733352 | -0.10819618 | 0.913841049 |
| SKMEL2:LY2157299:Ldose: 0 (uM) Interaction | 1.493436676 | 15.31686613 | 0.097502757 | 0.922328021 |
| MeWo:Mechlorethamine.HCl:Ldose: 0 (uM) Interaction | -3.138422873 | 15.13733352 | -0.207329968 | 0.835754065 |
| SKMEL2:Mechlorethamine.HCl:Ldose: 0 (uM) Interaction | -8.321943189 | 15.31686613 | -0.543318922 | 0.58691567 |
| UACC0257:Mechlorethamine.HCl:Ldose: 0 (uM) Interaction | 6.519348532 | 15.05685414 | 0.432982114 | 0.665031952 |
| MeWo:Megestrol.acetate:Ldose: 0 (uM) Interaction | 7.975896307 | 15.13733352 | 0.52690233 | 0.598266658 |
| SKMEL2:Megestrol.acetate:Ldose: 0 (uM) Interaction | -1.238144332 | 15.31686613 | -0.080835356 | 0.935573615 |
| MeWo:MEK.162..ARRY.438162.:Ldose: 0 (uM) Interaction | 23.52079622 | 15.13733352 | 1.553826913 | 0.120239903 |
| SKMEL2:MEK.162..ARRY.438162.:Ldose: 0 (uM) Interaction | 34.91684857 | 15.31686613 | 2.279633985 | 0.022638808 |
| UACC0257:MEK.162..ARRY.438162.:Ldose: 0 (uM) Interaction | 25.64299563 | 15.05685414 | 1.703077907 | 0.088567427 |
| MeWo:Melphalan:Ldose: 0 (uM) Interaction | 9.279210908 | 15.13733352 | 0.613001682 | 0.53988148 |
| SKMEL2:Melphalan:Ldose: 0 (uM) Interaction | -1.158054842 | 15.31686613 | -0.075606513 | 0.939732833 |
| UACC0257:Melphalan:Ldose: 0 (uM) Interaction | 11.1295107 | 15.05685414 | 0.739165738 | 0.459814144 |
| MeWo:Mercaptopurine:Ldose: 0 (uM) Interaction | -5.750041617 | 15.13733352 | -0.379858289 | 0.704054229 |
| SKMEL2:Mercaptopurine:Ldose: 0 (uM) Interaction | -3.460748551 | 15.31686613 | -0.225943644 | 0.821247323 |
| UACC0257:Mercaptopurine:Ldose: 0 (uM) Interaction | 16.07071627 | 15.05685414 | 1.067335588 | 0.28583188 |
| MeWo:Mitomycin.C:Ldose: 0 (uM) Interaction | 25.5512544 | 15.13733352 | 1.687962702 | 0.091432375 |
| SKMEL2:Mitomycin.C:Ldose: 0 (uM) Interaction | 10.1558061 | 15.31686613 | 0.663047259 | 0.507307142 |
| UACC0257:Mitomycin.C:Ldose: 0 (uM) Interaction | -13.45940745 | 15.05685414 | -0.893905681 | 0.371382007 |
| MeWo:Mitotane..o.p..DDD..Lysodren.:Ldose: 0 (uM) Interaction | 7.26184023 | 15.13733352 | 0.479730477 | 0.63142376 |
| SKMEL2:Mitotane..o.p..DDD..Lysodren.:Ldose: 0 (uM) Interaction | -6.856120036 | 15.31686613 | -0.447618983 | 0.65443255 |
| MeWo:Mitoxantrone:Ldose: 0 (uM) Interaction | 36.81249893 | 15.13733352 | 2.431901158 | 0.015027624 |
| SKMEL2:Mitoxantrone:Ldose: 0 (uM) Interaction | 48.6745454 | 15.31686613 | 3.177839709 | 0.001485792 |
| UACC0257:Mitoxantrone:Ldose: 0 (uM) Interaction | 26.03792218 | 15.05685414 | 1.729306928 | 0.083768019 |
| UACC0257:MLN.2480:Ldose: 0 (uM) Interaction | -18.56913177 | 15.05685414 | -1.233267693 | 0.217488909 |
| MeWo:MLN4924:Ldose: 0 (uM) Interaction | 27.63434344 | 15.13733352 | 1.825575383 | 0.067927617 |
| SKMEL2:MLN4924:Ldose: 0 (uM) Interaction | 43.17499454 | 15.31686613 | 2.818787745 | 0.004824785 |
| UACC0257:MLN4924:Ldose: 0 (uM) Interaction | 40.52285718 | 15.05685414 | 2.691322956 | 0.007122234 |
| MeWo:MLN9708..MLN2238.:Ldose: 0 (uM) Interaction | -12.87020967 | 15.13733352 | -0.850229642 | 0.395206554 |
| SKMEL2:MLN9708..MLN2238.:Ldose: 0 (uM) Interaction | -11.8918176 | 15.31686613 | -0.776387121 | 0.437528682 |
| UACC0257:MLN9708..MLN2238.:Ldose: 0 (uM) Interaction | 1.764588364 | 15.05685414 | 0.117195023 | 0.906706567 |
| MeWo:Navitoclax..ABT.263..5uM:Ldose: 0 (uM) Interaction | -9.194386853 | 15.13733352 | -0.607398049 | 0.543592969 |
| SKMEL2:Navitoclax..ABT.263..5uM:Ldose: 0 (uM) Interaction | -6.335988202 | 15.31686613 | -0.413660872 | 0.679126465 |
| UACC0257:Navitoclax..ABT.263..5uM:Ldose: 0 (uM) Interaction | 11.66489746 | 15.05685414 | 0.774723415 | 0.438511328 |
| MeWo:Nelarabine:Ldose: 0 (uM) Interaction | -3.057382838 | 15.13733352 | -0.201976315 | 0.839937083 |
| SKMEL2:Nelarabine:Ldose: 0 (uM) Interaction | -7.127249249 | 15.31686613 | -0.465320333 | 0.641706621 |
| UACC0257:Nelarabine:Ldose: 0 (uM) Interaction | 6.235302465 | 15.05685414 | 0.414117213 | 0.678792251 |
| MeWo:OSI.027:Ldose: 0 (uM) Interaction | -25.66198391 | 15.13733352 | -1.695277696 | 0.090036717 |
| SKMEL2:OSI.027:Ldose: 0 (uM) Interaction | -23.992782 | 15.31686613 | -1.566428916 | 0.11726243 |
| UACC0257:OSI.027:Ldose: 0 (uM) Interaction | -22.08893962 | 15.05685414 | -1.467035506 | 0.142380501 |
| MeWo:Oxaliplatin:Ldose: 0 (uM) Interaction | 0.251259083 | 15.07335079 | 0.016669093 | 0.986700753 |
| SKMEL2:Oxaliplatin:Ldose: 0 (uM) Interaction | 9.060398579 | 15.25363648 | 0.593982857 | 0.552529605 |
| MeWo:Paclitaxel:Ldose: 0 (uM) Interaction | 24.91104847 | 15.13733352 | 1.645669525 | 0.099845912 |
| SKMEL2:Paclitaxel:Ldose: 0 (uM) Interaction | 14.24495379 | 15.31686613 | 0.930017516 | 0.352372084 |
| MeWo:Palbociclib..PD.0332991..Isethionate:Ldose: 0 (uM) Interaction | 15.41046906 | 15.13733352 | 1.018043834 | 0.308668172 |
| SKMEL2:Palbociclib..PD.0332991..Isethionate:Ldose: 0 (uM) Interaction | 12.22884542 | 15.31686613 | 0.798390827 | 0.424652228 |
| UACC0257:Palbociclib..PD.0332991..Isethionate:Ldose: 0 (uM) Interaction | 5.249489565 | 15.05685414 | 0.348644512 | 0.727359498 |
| MeWo:Pazopanib.HCl:Ldose: 0 (uM) Interaction | 9.28174405 | 15.13733352 | 0.613169026 | 0.539770838 |
| SKMEL2:Pazopanib.HCl:Ldose: 0 (uM) Interaction | 6.085411178 | 15.31686613 | 0.397301323 | 0.691149075 |
| UACC0257:Pazopanib.HCl:Ldose: 0 (uM) Interaction | 9.861604514 | 15.05685414 | 0.65495783 | 0.512501662 |
| MeWo:PD325901:Ldose: 0 (uM) Interaction | -27.85621154 | 15.13733352 | -1.840232396 | 0.065747408 |
| SKMEL2:PD325901:Ldose: 0 (uM) Interaction | -32.09064252 | 15.31686613 | -2.095118038 | 0.036171753 |
| UACC0257:PD325901:Ldose: 0 (uM) Interaction | 2.431338239 | 15.05685414 | 0.161477173 | 0.871719052 |
| SKMEL2:Pemetrexed:Ldose: 0 (uM) Interaction | 5.908259801 | 15.31686613 | 0.385735551 | 0.699696211 |
| MeWo:Plicamycin:Ldose: 0 (uM) Interaction | 0.44290815 | 15.13733352 | 0.029259324 | 0.976658029 |
| SKMEL2:Plicamycin:Ldose: 0 (uM) Interaction | 23.51487361 | 15.31686613 | 1.535227469 | 0.124742153 |
| UACC0257:Plicamycin:Ldose: 0 (uM) Interaction | 18.28756374 | 15.05685414 | 1.21456737 | 0.224544003 |
| MeWo:Pralatrexate:Ldose: 0 (uM) Interaction | 4.837615435 | 15.07335079 | 0.320938291 | 0.748260164 |
| SKMEL2:Pralatrexate:Ldose: 0 (uM) Interaction | 4.402031774 | 15.25363648 | 0.288589005 | 0.772898593 |
| MeWo:Quinacrine.HCl:Ldose: 0 (uM) Interaction | -2.668186445 | 15.13733352 | -0.176265287 | 0.860087133 |
| SKMEL2:Quinacrine.HCl:Ldose: 0 (uM) Interaction | -3.363608191 | 15.31686613 | -0.219601592 | 0.826183462 |
| UACC0257:Quinacrine.HCl:Ldose: 0 (uM) Interaction | -3.037751278 | 15.05685414 | -0.201752056 | 0.840112405 |
| MeWo:Quizartinib:Ldose: 0 (uM) Interaction | 4.921883125 | 15.13733352 | 0.325148621 | 0.745071643 |
| SKMEL2:Quizartinib:Ldose: 0 (uM) Interaction | 1.097673893 | 15.31686613 | 0.071664392 | 0.942869637 |
| UACC0257:Quizartinib:Ldose: 0 (uM) Interaction | 0.006559309 | 15.05685414 | 0.000435636 | 0.999652417 |
| MeWo:Raloxifene:Ldose: 0 (uM) Interaction | 17.9904529 | 15.07335079 | 1.193527116 | 0.232675698 |
| SKMEL2:Raloxifene:Ldose: 0 (uM) Interaction | 17.97309163 | 15.25363648 | 1.178282415 | 0.238696611 |
| MeWo:Romidepsin:Ldose: 0 (uM) Interaction | 8.408466863 | 15.13733352 | 0.555478734 | 0.578572825 |
| SKMEL2:Romidepsin:Ldose: 0 (uM) Interaction | 4.121185221 | 15.31686613 | 0.269061908 | 0.787884533 |
| UACC0257:Romidepsin:Ldose: 0 (uM) Interaction | -2.254465365 | 15.05685414 | -0.149730172 | 0.880978857 |
| MeWo:Sabutoclax..BI.97C1.:Ldose: 0 (uM) Interaction | 5.440442222 | 15.13733352 | 0.359405586 | 0.7192951 |
| SKMEL2:Sabutoclax..BI.97C1.:Ldose: 0 (uM) Interaction | 21.80878949 | 15.31686613 | 1.42384149 | 0.154506356 |
| UACC0257:Sabutoclax..BI.97C1.:Ldose: 0 (uM) Interaction | 37.26111 | 15.05685414 | 2.474694226 | 0.01334241 |
| MeWo:Sirolimus..Rapamycin.:Ldose: 0 (uM) Interaction | -3.853434932 | 15.13733352 | -0.254564975 | 0.799061462 |
| SKMEL2:Sirolimus..Rapamycin.:Ldose: 0 (uM) Interaction | -6.162614571 | 15.31686613 | -0.40234174 | 0.687436397 |
| MeWo:Sorafenib:Ldose: 0 (uM) Interaction | 11.43268911 | 15.13733352 | 0.755264399 | 0.450098333 |
| UACC0257:Sorafenib:Ldose: 0 (uM) Interaction | 8.513341699 | 15.05685414 | 0.565413042 | 0.571798545 |
| MeWo:Streptozocin:Ldose: 0 (uM) Interaction | 10.20706901 | 15.13733352 | 0.674297689 | 0.500129064 |
| SKMEL2:Streptozocin:Ldose: 0 (uM) Interaction | 0.014205466 | 15.31686613 | 0.000927439 | 0.999260019 |
| UACC0257:Streptozocin:Ldose: 0 (uM) Interaction | 10.38327661 | 15.05685414 | 0.689604649 | 0.49045002 |
| MeWo:Sunitinib:Ldose: 0 (uM) Interaction | 2.143339828 | 15.13733352 | 0.141592958 | 0.887402809 |
| UACC0257:Sunitinib:Ldose: 0 (uM) Interaction | -1.490645121 | 15.05685414 | -0.0990011 | 0.921138287 |
| MeWo:Tamoxifen.Citrate:Ldose: 0 (uM) Interaction | 1.681404842 | 15.13733352 | 0.111076686 | 0.911556536 |
| SKMEL2:Tamoxifen.Citrate:Ldose: 0 (uM) Interaction | 6.400588591 | 15.31686613 | 0.417878471 | 0.676039998 |
| UACC0257:Tamoxifen.Citrate:Ldose: 0 (uM) Interaction | 1.736913492 | 15.05685414 | 0.115356998 | 0.908163201 |
| MeWo:Temozolomide:Ldose: 0 (uM) Interaction | 5.882913549 | 15.33308304 | 0.383674538 | 0.701223345 |
| SKMEL2:Temsirolimus..CCI.779..Torisel.:Ldose: 0 (uM) Interaction | 4.234881214 | 15.31686613 | 0.276484836 | 0.782178268 |
| MeWo:Teniposide:Ldose: 0 (uM) Interaction | 14.5310759 | 15.13733352 | 0.95994951 | 0.337091036 |
| SKMEL2:Teniposide:Ldose: 0 (uM) Interaction | 6.002156151 | 15.31686613 | 0.39186581 | 0.695161112 |
| UACC0257:Teniposide:Ldose: 0 (uM) Interaction | 23.16275617 | 15.05685414 | 1.538352962 | 0.123976527 |
| MeWo:Thioguanine:Ldose: 0 (uM) Interaction | 18.74473427 | 15.13733352 | 1.238311506 | 0.215613617 |
| SKMEL2:Thioguanine:Ldose: 0 (uM) Interaction | 21.21753852 | 15.31686613 | 1.385240188 | 0.165992894 |
| UACC0257:Thioguanine:Ldose: 0 (uM) Interaction | 31.10914276 | 15.05685414 | 2.066111717 | 0.038829455 |
| MeWo:Thiotepa:Ldose: 0 (uM) Interaction | -7.255568523 | 15.13733352 | -0.479316156 | 0.631718431 |
| SKMEL2:Thiotepa:Ldose: 0 (uM) Interaction | -0.855559462 | 15.31686613 | -0.055857344 | 0.955455953 |
| UACC0257:Thiotepa:Ldose: 0 (uM) Interaction | 12.41860886 | 15.05685414 | 0.824781109 | 0.409504697 |
| MeWo:Topotecan.HCl:Ldose: 0 (uM) Interaction | 59.54951634 | 15.13733352 | 3.933950206 | 8.38E-05 |
| SKMEL2:Topotecan.HCl:Ldose: 0 (uM) Interaction | 60.54683724 | 15.31686613 | 3.952952042 | 7.74E-05 |
| UACC0257:Topotecan.HCl:Ldose: 0 (uM) Interaction | 21.68619137 | 15.05685414 | 1.440287006 | 0.149800257 |
| MeWo:Trametinib..GSK1120212.:Ldose: 0 (uM) Interaction | -17.93696695 | 15.13733352 | -1.184948916 | 0.236050275 |
| UACC0257:Trametinib..GSK1120212.:Ldose: 0 (uM) Interaction | 9.0071104 | 15.05685414 | 0.598206658 | 0.549708126 |
| MeWo:Triethylenemelamine:Ldose: 0 (uM) Interaction | -0.312146558 | 15.13733352 | -0.020620974 | 0.983548194 |
| SKMEL2:Triethylenemelamine:Ldose: 0 (uM) Interaction | -3.714844412 | 15.31686613 | -0.242532929 | 0.808369475 |
| UACC0257:Triethylenemelamine:Ldose: 0 (uM) Interaction | 4.410999292 | 15.05685414 | 0.292956234 | 0.769558331 |
| MeWo:Uracil.mustard:Ldose: 0 (uM) Interaction | 7.642257175 | 15.13733352 | 0.504861518 | 0.613661094 |
| SKMEL2:Uracil.mustard:Ldose: 0 (uM) Interaction | 2.732291933 | 15.31686613 | 0.178384528 | 0.85842262 |
| MeWo:Valrubicin:Ldose: 0 (uM) Interaction | 29.03853407 | 15.13733352 | 1.918338789 | 0.055080823 |
| SKMEL2:Valrubicin:Ldose: 0 (uM) Interaction | 11.73182337 | 15.31686613 | 0.765941497 | 0.443719222 |
| UACC0257:Valrubicin:Ldose: 0 (uM) Interaction | 15.68888341 | 15.05685414 | 1.041976184 | 0.297434019 |
| MeWo:Vandetanib:Ldose: 0 (uM) Interaction | -9.39561865 | 15.07335079 | -0.623326477 | 0.533076378 |
| SKMEL2:Vandetanib:Ldose: 0 (uM) Interaction | -7.397911813 | 15.25363648 | -0.484993321 | 0.627685848 |
| SKMEL2:Vemurafenib:Ldose: 0 (uM) Interaction | 60.0232637 | 15.31686613 | 3.918769231 | 8.93E-05 |
| UACC0257:Vemurafenib:Ldose: 0 (uM) Interaction | 28.0614157 | 15.05685414 | 1.863697121 | 0.062377385 |
| SKMEL2:Vinblastine.Sulfate:Ldose: 0 (uM) Interaction | 5.441947711 | 15.31686613 | 0.355291198 | 0.72237481 |
| UACC0257:Vinblastine.Sulfate:Ldose: 0 (uM) Interaction | 21.91626159 | 15.05685414 | 1.455567105 | 0.145526407 |
| MeWo:Vincristine.Sulfate:Ldose: 0 (uM) Interaction | 28.02869973 | 15.13733352 | 1.851627282 | 0.064092575 |
| SKMEL2:Vincristine.Sulfate:Ldose: 0 (uM) Interaction | 48.64135804 | 15.31686613 | 3.175672989 | 0.001496928 |
| UACC0257:Vincristine.Sulfate:Ldose: 0 (uM) Interaction | 60.1778452 | 15.05685414 | 3.996707721 | 6.44E-05 |
| MeWo:Vinorelbine.Tartrate:Ldose: 0 (uM) Interaction | 9.314536762 | 15.13733352 | 0.615335372 | 0.538339543 |
| SKMEL2:Vinorelbine.Tartrate:Ldose: 0 (uM) Interaction | 14.82131651 | 15.31686613 | 0.967646801 | 0.333231291 |
| UACC0257:Vinorelbine.Tartrate:Ldose: 0 (uM) Interaction | 24.32195046 | 15.05685414 | 1.615340777 | 0.106251067 |
| MeWo:Vismodegib:Ldose: 0 (uM) Interaction | 6.474080625 | 15.13733352 | 0.427689633 | 0.668881229 |
| SKMEL2:Vismodegib:Ldose: 0 (uM) Interaction | -4.878124676 | 15.31686613 | -0.318480597 | 0.750123393 |
| UACC0257:Vismodegib:Ldose: 0 (uM) Interaction | 10.39041208 | 15.05685414 | 0.69007855 | 0.490151974 |
| MeWo:Vorinostat:Ldose: 0 (uM) Interaction | -14.18114704 | 15.13733352 | -0.936832568 | 0.348854837 |
| SKMEL2:Vorinostat:Ldose: 0 (uM) Interaction | 2.951964433 | 15.31686613 | 0.192726398 | 0.847175004 |
| UACC0257:Vorinostat:Ldose: 0 (uM) Interaction | 10.54438915 | 15.05685414 | 0.700304928 | 0.483744202 |
| MeWo:Zoledronic.Acid:Ldose: 0 (uM) Interaction | -2.230367213 | 15.33308304 | -0.145461106 | 0.884348137 |
| MeWo:Abiraterone:Ldose: 0.301029996 (uM) Interaction | -1.350389474 | 15.13733352 | -0.089209204 | 0.928916452 |
| SKMEL2:Abiraterone:Ldose: 0.301029996 (uM) Interaction | -13.37635968 | 15.31686613 | -0.873309172 | 0.382503988 |
| MeWo:ABT.737:Ldose: 0.301029996 (uM) Interaction | -2.284467887 | 15.13733352 | -0.150916136 | 0.880043237 |
| SKMEL2:ABT.737:Ldose: 0.301029996 (uM) Interaction | -0.328053504 | 15.31686613 | -0.021417795 | 0.98291257 |
| UACC0257:ABT.737:Ldose: 0.301029996 (uM) Interaction | -1.060978064 | 15.05685414 | -0.07046479 | 0.943824356 |
| MeWo:Actinomycin.D:Ldose: 0.301029996 (uM) Interaction | -6.068733224 | 15.13733352 | -0.400911641 | 0.688489021 |
| SKMEL2:Actinomycin.D:Ldose: 0.301029996 (uM) Interaction | -45.30143387 | 15.31686613 | -2.957617668 | 0.003103516 |
| UACC0257:Actinomycin.D:Ldose: 0.301029996 (uM) Interaction | -24.07206719 | 15.05685414 | -1.598744795 | 0.10989149 |
| MeWo:Afatinib:Ldose: 0.301029996 (uM) Interaction | 8.737426596 | 15.13733352 | 0.577210417 | 0.563803149 |
| SKMEL2:Afatinib:Ldose: 0.301029996 (uM) Interaction | -2.231284189 | 15.31686613 | -0.145674981 | 0.884179289 |
| UACC0257:Afatinib:Ldose: 0.301029996 (uM) Interaction | -0.539751691 | 15.05685414 | -0.035847574 | 0.97140422 |
| MeWo:Alisertib..MLN8237.:Ldose: 0.301029996 (uM) Interaction | 19.24466674 | 15.13733352 | 1.271337961 | 0.203621705 |
| SKMEL2:Alisertib..MLN8237.:Ldose: 0.301029996 (uM) Interaction | 2.770268391 | 15.31686613 | 0.180863916 | 0.856476036 |
| MeWo:Allopurinol:Ldose: 0.301029996 (uM) Interaction | 1.767093653 | 15.13733352 | 0.116737446 | 0.907069167 |
| SKMEL2:Allopurinol:Ldose: 0.301029996 (uM) Interaction | -15.78345123 | 15.31686613 | -1.030462178 | 0.302804276 |
| UACC0257:Allopurinol:Ldose: 0.301029996 (uM) Interaction | 2.778071729 | 15.05685414 | 0.184505455 | 0.853618626 |
| MeWo:Amifostine:Ldose: 0.301029996 (uM) Interaction | 0.364136976 | 15.13733352 | 0.024055556 | 0.980808509 |
| MeWo:Aphrocallistin.analogue:Ldose: 0.301029996 (uM) Interaction | 35.00405267 | 15.13733352 | 2.312431884 | 0.020762956 |
| SKMEL2:Aphrocallistin.analogue:Ldose: 0.301029996 (uM) Interaction | 28.51616507 | 15.31686613 | 1.861749318 | 0.062651581 |
| UACC0257:Aphrocallistin.analogue:Ldose: 0.301029996 (uM) Interaction | 47.56185089 | 15.05685414 | 3.15881727 | 0.001586222 |
| MeWo:Arsenic.Trioxide:Ldose: 0.301029996 (uM) Interaction | 16.69042731 | 15.13733352 | 1.102600222 | 0.270212715 |
| SKMEL2:Arsenic.Trioxide:Ldose: 0.301029996 (uM) Interaction | -8.744866809 | 15.31686613 | -0.57093055 | 0.568052475 |
| MeWo:Axitinib:Ldose: 0.301029996 (uM) Interaction | 2.775414403 | 15.13733352 | 0.183348963 | 0.854525886 |
| SKMEL2:Axitinib:Ldose: 0.301029996 (uM) Interaction | -7.845861342 | 15.31686613 | -0.512236725 | 0.608490417 |
| UACC0257:Axitinib:Ldose: 0.301029996 (uM) Interaction | -15.36786195 | 15.05685414 | -1.020655564 | 0.307428729 |
| MeWo:Axitinib.1:Ldose: 0.301029996 (uM) Interaction | 5.747432393 | 15.13733352 | 0.379685919 | 0.70418219 |
| SKMEL2:Axitinib.1:Ldose: 0.301029996 (uM) Interaction | 2.735303781 | 15.31686613 | 0.178581164 | 0.858268208 |
| UACC0257:Axitinib.1:Ldose: 0.301029996 (uM) Interaction | -2.200731706 | 15.05685414 | -0.146161455 | 0.883795255 |
| MeWo:Azacitidine:Ldose: 0.301029996 (uM) Interaction | 7.40174093 | 15.13733352 | 0.488972574 | 0.624865924 |
| SKMEL2:Azacitidine:Ldose: 0.301029996 (uM) Interaction | -17.68657554 | 15.31686613 | -1.154712419 | 0.248220588 |
| UACC0257:Azacitidine:Ldose: 0.301029996 (uM) Interaction | 14.09798153 | 15.05685414 | 0.936316537 | 0.349120377 |
| SKMEL2:Baricitinib..LY3009104..INCB028050.:Ldose: 0.301029996 (uM) Interaction | 14.8576508 | 15.31686613 | 0.970018976 | 0.332047556 |
| UACC0257:Baricitinib..LY3009104..INCB028050.:Ldose: 0.301029996 (uM) Interaction | 3.300518291 | 15.05685414 | 0.21920371 | 0.826493372 |
| SKMEL2:Bendamustine.HCl:Ldose: 0.301029996 (uM) Interaction | 13.23433642 | 15.25363648 | 0.867618449 | 0.385612581 |
| MeWo:Bioymifi:Ldose: 0.301029996 (uM) Interaction | 0.037782361 | 15.13733352 | 0.002495972 | 0.998008527 |
| SKMEL2:Bioymifi:Ldose: 0.301029996 (uM) Interaction | 7.354697351 | 15.31686613 | 0.480169853 | 0.631111332 |
| UACC0257:Bioymifi:Ldose: 0.301029996 (uM) Interaction | 6.152592064 | 15.05685414 | 0.408624007 | 0.682819535 |
| MeWo:Bleomycin.Sulfate:Ldose: 0.301029996 (uM) Interaction | 30.83488618 | 15.13733352 | 2.037009102 | 0.041660916 |
| SKMEL2:Bleomycin.Sulfate:Ldose: 0.301029996 (uM) Interaction | 26.08351786 | 15.31686613 | 1.702927847 | 0.08859551 |
| UACC0257:Bleomycin.Sulfate:Ldose: 0.301029996 (uM) Interaction | 55.85183854 | 15.05685414 | 3.709396267 | 0.000208258 |
| MeWo:Bortezomib:Ldose: 0.301029996 (uM) Interaction | 10.84469357 | 15.13733352 | 0.716420336 | 0.473739346 |
| SKMEL2:Bortezomib:Ldose: 0.301029996 (uM) Interaction | 6.574257668 | 15.31686613 | 0.429216892 | 0.667769539 |
| UACC0257:Bortezomib:Ldose: 0.301029996 (uM) Interaction | 18.89475052 | 15.05685414 | 1.254893641 | 0.209530526 |
| MeWo:Bosutinib..SKI.606.:Ldose: 0.301029996 (uM) Interaction | 25.05185526 | 15.13733352 | 1.65497148 | 0.097944358 |
| SKMEL2:Bosutinib..SKI.606.:Ldose: 0.301029996 (uM) Interaction | 27.02589371 | 15.31686613 | 1.764453217 | 0.077669395 |
| UACC0257:Bosutinib..SKI.606.:Ldose: 0.301029996 (uM) Interaction | 9.856805794 | 15.05685414 | 0.654639123 | 0.512706881 |
| SKMEL2:Busulfan:Ldose: 0.301029996 (uM) Interaction | -2.014164332 | 15.31686613 | -0.131499767 | 0.895381157 |
| MeWo:Cabazitaxel:Ldose: 0.301029996 (uM) Interaction | 7.772164283 | 15.13733352 | 0.513443419 | 0.607646268 |
| SKMEL2:Cabazitaxel:Ldose: 0.301029996 (uM) Interaction | -12.19199838 | 15.31686613 | -0.795985176 | 0.426049126 |
| UACC0257:Cabazitaxel:Ldose: 0.301029996 (uM) Interaction | -5.070246702 | 15.05685414 | -0.336740109 | 0.736315994 |
| MeWo:Cabozantinib..XL.184.:Ldose: 0.301029996 (uM) Interaction | 9.597577781 | 15.13733352 | 0.634033581 | 0.526065411 |
| SKMEL2:Cabozantinib..XL.184.:Ldose: 0.301029996 (uM) Interaction | 15.72936995 | 15.31686613 | 1.026931346 | 0.304463933 |
| UACC0257:Cabozantinib..XL.184.:Ldose: 0.301029996 (uM) Interaction | 5.981262171 | 15.05685414 | 0.397245143 | 0.691190499 |
| MeWo:Capecitabine:Ldose: 0.301029996 (uM) Interaction | 9.424184279 | 15.33308304 | 0.614630747 | 0.538804876 |
| MeWo:Carfilzomib:Ldose: 0.301029996 (uM) Interaction | -31.31413787 | 15.13733352 | -2.068669348 | 0.038588625 |
| SKMEL2:Carfilzomib:Ldose: 0.301029996 (uM) Interaction | 31.00334748 | 15.31686613 | 2.024131256 | 0.042968548 |
| UACC0257:Carfilzomib:Ldose: 0.301029996 (uM) Interaction | -40.17077263 | 15.05685414 | -2.667939283 | 0.007637322 |
| MeWo:Carmustine:Ldose: 0.301029996 (uM) Interaction | 7.73117411 | 15.33308304 | 0.504215238 | 0.614115116 |
| MeWo:Celecoxib:Ldose: 0.301029996 (uM) Interaction | 8.561094595 | 15.33308304 | 0.55834137 | 0.576616907 |
| UACC0257:Chlorambucil:Ldose: 0.301029996 (uM) Interaction | -12.47735899 | 15.05685414 | -0.828682995 | 0.407292681 |
| MeWo:Cisplatin:Ldose: 0.301029996 (uM) Interaction | 10.57889166 | 15.13733352 | 0.698860975 | 0.484646208 |
| SKMEL2:Cisplatin:Ldose: 0.301029996 (uM) Interaction | 7.212083721 | 15.31686613 | 0.470858964 | 0.63774606 |
| MeWo:Cladribine:Ldose: 0.301029996 (uM) Interaction | 55.44103839 | 15.13733352 | 3.662536622 | 0.000250308 |
| SKMEL2:Cladribine:Ldose: 0.301029996 (uM) Interaction | 32.47707408 | 15.31686613 | 2.120347192 | 0.033987788 |
| UACC0257:Cladribine:Ldose: 0.301029996 (uM) Interaction | 22.94799586 | 15.05685414 | 1.52408967 | 0.127500496 |
| MeWo:Clofarabine:Ldose: 0.301029996 (uM) Interaction | 49.85220532 | 15.13733352 | 3.293328065 | 0.00099163 |
| SKMEL2:Clofarabine:Ldose: 0.301029996 (uM) Interaction | 28.06061496 | 15.31686613 | 1.832007587 | 0.066963622 |
| UACC0257:Clofarabine:Ldose: 0.301029996 (uM) Interaction | -0.743731847 | 15.05685414 | -0.049394903 | 0.960605033 |
| MeWo:Crizotinib:Ldose: 0.301029996 (uM) Interaction | 3.250361728 | 15.13733352 | 0.214724854 | 0.829983808 |
| SKMEL2:Crizotinib:Ldose: 0.301029996 (uM) Interaction | -5.611715734 | 15.31686613 | -0.366374929 | 0.714088802 |
| UACC0257:Crizotinib:Ldose: 0.301029996 (uM) Interaction | -11.94866211 | 15.05685414 | -0.793569626 | 0.427454466 |
| MeWo:Cytarabine.HCl...Ara.C:Ldose: 0.301029996 (uM) Interaction | 67.09805034 | 15.13733352 | 4.432620199 | 9.35E-06 |
| SKMEL2:Cytarabine.HCl...Ara.C:Ldose: 0.301029996 (uM) Interaction | 58.21967885 | 15.31686613 | 3.801017673 | 0.000144486 |
| UACC0257:Cytarabine.HCl...Ara.C:Ldose: 0.301029996 (uM) Interaction | 38.90555592 | 15.05685414 | 2.583909996 | 0.009775028 |
| MeWo:Dacarbazine:Ldose: 0.301029996 (uM) Interaction | 16.26738551 | 15.13733352 | 1.074653306 | 0.282541606 |
| SKMEL2:Dacarbazine:Ldose: 0.301029996 (uM) Interaction | 12.06542749 | 15.31686613 | 0.787721678 | 0.430867896 |
| MeWo:Dacomitinib..PF299804.:Ldose: 0.301029996 (uM) Interaction | 4.249981661 | 15.13733352 | 0.280761579 | 0.778895879 |
| SKMEL2:Dacomitinib..PF299804.:Ldose: 0.301029996 (uM) Interaction | -1.039841954 | 15.31686613 | -0.067888689 | 0.945874852 |
| UACC0257:Dacomitinib..PF299804.:Ldose: 0.301029996 (uM) Interaction | 14.53092649 | 15.05685414 | 0.965070549 | 0.334519944 |
| MeWo:Dasatinib:Ldose: 0.301029996 (uM) Interaction | -5.9357618 | 15.13733352 | -0.392127305 | 0.694967901 |
| SKMEL2:Dasatinib:Ldose: 0.301029996 (uM) Interaction | -17.8518549 | 15.31686613 | -1.165503097 | 0.243827876 |
| UACC0257:Dasatinib:Ldose: 0.301029996 (uM) Interaction | 0.269456913 | 15.05685414 | 0.017895964 | 0.985722009 |
| MeWo:Daunorubicin.HCl:Ldose: 0.301029996 (uM) Interaction | -0.957766881 | 15.13733352 | -0.063271836 | 0.94955061 |
| SKMEL2:Daunorubicin.HCl:Ldose: 0.301029996 (uM) Interaction | 15.91508303 | 15.31686613 | 1.03905609 | 0.298789918 |
| UACC0257:Daunorubicin.HCl:Ldose: 0.301029996 (uM) Interaction | -16.98710342 | 15.05685414 | -1.128197382 | 0.259248709 |
| MeWo:Decitabine:Ldose: 0.301029996 (uM) Interaction | 4.794119913 | 15.13733352 | 0.316708349 | 0.751467878 |
| SKMEL2:Decitabine:Ldose: 0.301029996 (uM) Interaction | -6.309229525 | 15.31686613 | -0.411913865 | 0.680406518 |
| MeWo:Docetaxel:Ldose: 0.301029996 (uM) Interaction | 31.61373434 | 15.13733352 | 2.08846124 | 0.036767564 |
| MeWo:Doxorubicin.HCl:Ldose: 0.301029996 (uM) Interaction | 3.131695179 | 15.13733352 | 0.206885524 | 0.836101151 |
| SKMEL2:Doxorubicin.HCl:Ldose: 0.301029996 (uM) Interaction | -16.24996446 | 15.31686613 | -1.060919663 | 0.288737904 |
| UACC0257:Doxorubicin.HCl:Ldose: 0.301029996 (uM) Interaction | -18.32690098 | 15.05685414 | -1.217179951 | 0.22354864 |
| MeWo:Erlotinib.HCl:Ldose: 0.301029996 (uM) Interaction | -1.62178819 | 15.13733352 | -0.107138301 | 0.914680226 |
| SKMEL2:Erlotinib.HCl:Ldose: 0.301029996 (uM) Interaction | -2.880844074 | 15.31686613 | -0.188083127 | 0.8508132 |
| UACC0257:Erlotinib.HCl:Ldose: 0.301029996 (uM) Interaction | -6.462016556 | 15.05685414 | -0.429174414 | 0.667800448 |
| MeWo:Etoposide:Ldose: 0.301029996 (uM) Interaction | 40.79326023 | 15.13733352 | 2.694877546 | 0.007046727 |
| SKMEL2:Etoposide:Ldose: 0.301029996 (uM) Interaction | 38.10906144 | 15.31686613 | 2.488045604 | 0.012851972 |
| UACC0257:Etoposide:Ldose: 0.301029996 (uM) Interaction | 33.55660852 | 15.05685414 | 2.228659998 | 0.025846465 |
| MeWo:Everolimus:Ldose: 0.301029996 (uM) Interaction | 16.85447002 | 15.33308304 | 1.09922251 | 0.271682892 |
| MeWo:Exemestane:Ldose: 0.301029996 (uM) Interaction | 13.78596097 | 15.07335079 | 0.914591663 | 0.360415928 |
| SKMEL2:Exemestane:Ldose: 0.301029996 (uM) Interaction | 0.616659399 | 15.25363648 | 0.040427042 | 0.967753034 |
| MeWo:Floxuridine:Ldose: 0.301029996 (uM) Interaction | 21.30630819 | 15.13733352 | 1.407533774 | 0.15928311 |
| SKMEL2:Floxuridine:Ldose: 0.301029996 (uM) Interaction | 58.94252364 | 15.31686613 | 3.848210407 | 0.000119315 |
| UACC0257:Floxuridine:Ldose: 0.301029996 (uM) Interaction | -0.019843066 | 15.05685414 | -0.001317876 | 0.998948499 |
| MeWo:Fludarabine.Phosphate:Ldose: 0.301029996 (uM) Interaction | 7.123842073 | 15.13733352 | 0.470614066 | 0.637920965 |
| SKMEL2:Fludarabine.Phosphate:Ldose: 0.301029996 (uM) Interaction | 10.20898798 | 15.31686613 | 0.666519371 | 0.505086083 |
| MeWo:Fluorouracil...5.FU.:Ldose: 0.301029996 (uM) Interaction | -10.0746135 | 15.13733352 | -0.665547435 | 0.505707299 |
| SKMEL2:Fluorouracil...5.FU.:Ldose: 0.301029996 (uM) Interaction | -6.80423907 | 15.31686613 | -0.444231804 | 0.656879312 |
| UACC0257:Fluorouracil...5.FU.:Ldose: 0.301029996 (uM) Interaction | -2.476181796 | 15.05685414 | -0.164455455 | 0.869374102 |
| MeWo:Flutamide..Eulexin.:Ldose: 0.301029996 (uM) Interaction | 6.704764143 | 15.13733352 | 0.442929009 | 0.657821381 |
| SKMEL2:Flutamide..Eulexin.:Ldose: 0.301029996 (uM) Interaction | 2.887036113 | 15.31686613 | 0.18848739 | 0.850496315 |
| UACC0257:Flutamide..Eulexin.:Ldose: 0.301029996 (uM) Interaction | 7.945533794 | 15.05685414 | 0.527702116 | 0.597711355 |
| MeWo:Foretinib..GSK1363089.:Ldose: 0.301029996 (uM) Interaction | 24.07367522 | 15.13733352 | 1.590351113 | 0.111769861 |
| SKMEL2:Foretinib..GSK1363089.:Ldose: 0.301029996 (uM) Interaction | 49.59759764 | 15.31686613 | 3.238103488 | 0.001205031 |
| UACC0257:Foretinib..GSK1363089.:Ldose: 0.301029996 (uM) Interaction | 6.174958281 | 15.05685414 | 0.410109457 | 0.681729596 |
| SKMEL2:Fulvestrant:Ldose: 0.301029996 (uM) Interaction | -3.272143587 | 15.31686613 | -0.213630096 | 0.830837481 |
| UACC0257:Fulvestrant:Ldose: 0.301029996 (uM) Interaction | 16.17128058 | 15.05685414 | 1.07401456 | 0.282827779 |
| MeWo:Gefitinib:Ldose: 0.301029996 (uM) Interaction | 10.78054703 | 15.33308304 | 0.703090631 | 0.482006611 |
| MeWo:Gemcitabine.HCl:Ldose: 0.301029996 (uM) Interaction | 35.82551867 | 15.13733352 | 2.366699433 | 0.017955997 |
| SKMEL2:Gemcitabine.HCl:Ldose: 0.301029996 (uM) Interaction | 64.2407251 | 15.31686613 | 4.194116771 | 2.75E-05 |
| UACC0257:Gemcitabine.HCl:Ldose: 0.301029996 (uM) Interaction | -13.01199044 | 15.05685414 | -0.864190509 | 0.387492542 |
| MeWo:Ibrutinib..PCI.32765.:Ldose: 0.301029996 (uM) Interaction | -9.422993318 | 15.07335079 | -0.625142574 | 0.531883885 |
| SKMEL2:Ibrutinib..PCI.32765.:Ldose: 0.301029996 (uM) Interaction | -14.38661874 | 15.25363648 | -0.943159932 | 0.345609324 |
| MeWo:Imiquimod:Ldose: 0.301029996 (uM) Interaction | -19.67658115 | 15.07335079 | -1.305388658 | 0.191774009 |
| SKMEL2:Imiquimod:Ldose: 0.301029996 (uM) Interaction | -24.34473701 | 15.25363648 | -1.59599562 | 0.110503944 |
| MeWo:INK.128..MLN0128.:Ldose: 0.301029996 (uM) Interaction | 30.18902851 | 15.13733352 | 1.994342561 | 0.046126787 |
| SKMEL2:INK.128..MLN0128.:Ldose: 0.301029996 (uM) Interaction | -3.82415068 | 15.31686613 | -0.249669263 | 0.80284543 |
| UACC0257:INK.128..MLN0128.:Ldose: 0.301029996 (uM) Interaction | 8.045325353 | 15.05685414 | 0.534329766 | 0.593118729 |
| MeWo:Irinotecan.HCl:Ldose: 0.301029996 (uM) Interaction | 45.24839042 | 15.13733352 | 2.989191613 | 0.002800216 |
| SKMEL2:Irinotecan.HCl:Ldose: 0.301029996 (uM) Interaction | 24.29280475 | 15.31686613 | 1.586016653 | 0.112749713 |
| UACC0257:Irinotecan.HCl:Ldose: 0.301029996 (uM) Interaction | 21.27155259 | 15.05685414 | 1.412748798 | 0.157743556 |
| MeWo:Ixabepilone:Ldose: 0.301029996 (uM) Interaction | 29.89002467 | 15.13733352 | 1.97458982 | 0.048327035 |
| SKMEL2:Ixabepilone:Ldose: 0.301029996 (uM) Interaction | 15.29836025 | 15.31686613 | 0.998791798 | 0.3179064 |
| UACC0257:Ixabepilone:Ldose: 0.301029996 (uM) Interaction | 17.51882836 | 15.05685414 | 1.16351186 | 0.244634341 |
| MeWo:Lapatinib:Ldose: 0.301029996 (uM) Interaction | 0.834197999 | 15.07335079 | 0.055342572 | 0.955866043 |
| SKMEL2:Lapatinib:Ldose: 0.301029996 (uM) Interaction | -8.885927561 | 15.25363648 | -0.582544862 | 0.560205623 |
| MeWo:LDK378:Ldose: 0.301029996 (uM) Interaction | -4.446452657 | 15.13733352 | -0.293740813 | 0.768958699 |
| SKMEL2:LDK378:Ldose: 0.301029996 (uM) Interaction | -2.626718229 | 15.31686613 | -0.171491884 | 0.863838568 |
| UACC0257:LDK378:Ldose: 0.301029996 (uM) Interaction | 4.933896094 | 15.05685414 | 0.327684392 | 0.743153382 |
| MeWo:Lenalidomide:Ldose: 0.301029996 (uM) Interaction | 9.438795163 | 15.13733352 | 0.62354411 | 0.532933404 |
| SKMEL2:Lenalidomide:Ldose: 0.301029996 (uM) Interaction | -4.586225994 | 15.31686613 | -0.29942326 | 0.764619908 |
| SKMEL2:Letrozole:Ldose: 0.301029996 (uM) Interaction | 4.02607155 | 15.31686613 | 0.262852173 | 0.792666949 |
| MeWo:Linsitinib:Ldose: 0.301029996 (uM) Interaction | 9.625043918 | 15.13733352 | 0.635848044 | 0.524881996 |
| SKMEL2:Linsitinib:Ldose: 0.301029996 (uM) Interaction | 2.696810621 | 15.31686613 | 0.176068042 | 0.860242087 |
| UACC0257:Linsitinib:Ldose: 0.301029996 (uM) Interaction | -8.166583281 | 15.05685414 | -0.542383104 | 0.587560038 |
| SKMEL2:Lomustine..CCNU.:Ldose: 0.301029996 (uM) Interaction | -9.15571793 | 15.31686613 | -0.597753996 | 0.550010162 |
| MeWo:LY2157299:Ldose: 0.301029996 (uM) Interaction | -1.941503324 | 15.13733352 | -0.128259268 | 0.897944955 |
| SKMEL2:LY2157299:Ldose: 0.301029996 (uM) Interaction | -1.716641882 | 15.31686613 | -0.112075268 | 0.910764736 |
| MeWo:Mechlorethamine.HCl:Ldose: 0.301029996 (uM) Interaction | -0.640906584 | 15.13733352 | -0.042339464 | 0.966228465 |
| SKMEL2:Mechlorethamine.HCl:Ldose: 0.301029996 (uM) Interaction | -4.566106249 | 15.31686613 | -0.298109692 | 0.765622222 |
| UACC0257:Mechlorethamine.HCl:Ldose: 0.301029996 (uM) Interaction | 2.474372129 | 15.05685414 | 0.164335266 | 0.869468711 |
| MeWo:Megestrol.acetate:Ldose: 0.301029996 (uM) Interaction | 19.99725047 | 15.13733352 | 1.321055022 | 0.186496576 |
| SKMEL2:Megestrol.acetate:Ldose: 0.301029996 (uM) Interaction | 10.27832279 | 15.31686613 | 0.671046068 | 0.502198134 |
| MeWo:MEK.162..ARRY.438162.:Ldose: 0.301029996 (uM) Interaction | 24.31071996 | 15.13733352 | 1.606010723 | 0.108285716 |
| SKMEL2:MEK.162..ARRY.438162.:Ldose: 0.301029996 (uM) Interaction | 21.20460777 | 15.31686613 | 1.384395972 | 0.166251097 |
| UACC0257:MEK.162..ARRY.438162.:Ldose: 0.301029996 (uM) Interaction | 29.82433482 | 15.05685414 | 1.980781281 | 0.047628076 |
| MeWo:Melphalan:Ldose: 0.301029996 (uM) Interaction | 2.380853476 | 15.13733352 | 0.157283545 | 0.875022812 |
| SKMEL2:Melphalan:Ldose: 0.301029996 (uM) Interaction | -7.435345877 | 15.31686613 | -0.485435194 | 0.627372443 |
| UACC0257:Melphalan:Ldose: 0.301029996 (uM) Interaction | 13.66833236 | 15.05685414 | 0.907781415 | 0.364003531 |
| MeWo:Mercaptopurine:Ldose: 0.301029996 (uM) Interaction | -7.359219542 | 15.13733352 | -0.486163533 | 0.626856003 |
| SKMEL2:Mercaptopurine:Ldose: 0.301029996 (uM) Interaction | 3.339183351 | 15.31686613 | 0.218006955 | 0.827425687 |
| UACC0257:Mercaptopurine:Ldose: 0.301029996 (uM) Interaction | 3.310544779 | 15.05685414 | 0.219869619 | 0.825974711 |
| MeWo:Mitomycin.C:Ldose: 0.301029996 (uM) Interaction | 23.24770114 | 15.13733352 | 1.535785752 | 0.124605125 |
| SKMEL2:Mitomycin.C:Ldose: 0.301029996 (uM) Interaction | 9.161933305 | 15.31686613 | 0.598159782 | 0.5497394 |
| UACC0257:Mitomycin.C:Ldose: 0.301029996 (uM) Interaction | -16.01610952 | 15.05685414 | -1.063708884 | 0.287472119 |
| MeWo:Mitotane..o.p..DDD..Lysodren.:Ldose: 0.301029996 (uM) Interaction | 19.44275279 | 15.13733352 | 1.284423888 | 0.199007024 |
| SKMEL2:Mitotane..o.p..DDD..Lysodren.:Ldose: 0.301029996 (uM) Interaction | -11.13137869 | 15.31686613 | -0.726739961 | 0.467392899 |
| MeWo:Mitoxantrone:Ldose: 0.301029996 (uM) Interaction | 28.1250843 | 15.13733352 | 1.857994624 | 0.06318295 |
| SKMEL2:Mitoxantrone:Ldose: 0.301029996 (uM) Interaction | 41.71748497 | 15.31686613 | 2.72363058 | 0.006461895 |
| UACC0257:Mitoxantrone:Ldose: 0.301029996 (uM) Interaction | 25.67871538 | 15.05685414 | 1.705450232 | 0.088124416 |
| UACC0257:MLN.2480:Ldose: 0.301029996 (uM) Interaction | -4.574705127 | 15.05685414 | -0.303828747 | 0.7612612 |
| MeWo:MLN4924:Ldose: 0.301029996 (uM) Interaction | 25.1205159 | 15.13733352 | 1.659507327 | 0.097027669 |
| SKMEL2:MLN4924:Ldose: 0.301029996 (uM) Interaction | 30.74871075 | 15.31686613 | 2.007506659 | 0.044707786 |
| UACC0257:MLN4924:Ldose: 0.301029996 (uM) Interaction | 40.18535036 | 15.05685414 | 2.668907462 | 0.00761535 |
| MeWo:MLN9708..MLN2238.:Ldose: 0.301029996 (uM) Interaction | -12.84324113 | 15.13733352 | -0.848448052 | 0.3961976 |
| SKMEL2:MLN9708..MLN2238.:Ldose: 0.301029996 (uM) Interaction | 15.81356166 | 15.31686613 | 1.032428013 | 0.301882853 |
| UACC0257:MLN9708..MLN2238.:Ldose: 0.301029996 (uM) Interaction | -4.487228076 | 15.05685414 | -0.298018964 | 0.765691466 |
| MeWo:Navitoclax..ABT.263..5uM:Ldose: 0.301029996 (uM) Interaction | -1.910515332 | 15.13733352 | -0.126212145 | 0.899565136 |
| SKMEL2:Navitoclax..ABT.263..5uM:Ldose: 0.301029996 (uM) Interaction | 5.740852513 | 15.31686613 | 0.374805947 | 0.707808356 |
| UACC0257:Navitoclax..ABT.263..5uM:Ldose: 0.301029996 (uM) Interaction | 11.09051289 | 15.05685414 | 0.736575701 | 0.461388166 |
| MeWo:Nelarabine:Ldose: 0.301029996 (uM) Interaction | 6.944290315 | 15.13733352 | 0.458752547 | 0.646416346 |
| SKMEL2:Nelarabine:Ldose: 0.301029996 (uM) Interaction | -19.43055422 | 15.31686613 | -1.268572439 | 0.204606844 |
| UACC0257:Nelarabine:Ldose: 0.301029996 (uM) Interaction | 16.08727339 | 15.05685414 | 1.068435228 | 0.285335801 |
| MeWo:OSI.027:Ldose: 0.301029996 (uM) Interaction | -35.68641735 | 15.13733352 | -2.357510145 | 0.018406543 |
| SKMEL2:OSI.027:Ldose: 0.301029996 (uM) Interaction | 7.940795551 | 15.31686613 | 0.518434743 | 0.604160124 |
| UACC0257:OSI.027:Ldose: 0.301029996 (uM) Interaction | -31.67086621 | 15.05685414 | -2.103418544 | 0.035440373 |
| MeWo:Oxaliplatin:Ldose: 0.301029996 (uM) Interaction | 9.508151916 | 15.07335079 | 0.630792187 | 0.528182878 |
| SKMEL2:Oxaliplatin:Ldose: 0.301029996 (uM) Interaction | 4.334616245 | 15.25363648 | 0.284169369 | 0.776283227 |
| MeWo:Paclitaxel:Ldose: 0.301029996 (uM) Interaction | 45.268911 | 15.13733352 | 2.990547241 | 0.002787822 |
| SKMEL2:Paclitaxel:Ldose: 0.301029996 (uM) Interaction | 53.45568852 | 15.31686613 | 3.489988623 | 0.000483974 |
| MeWo:Palbociclib..PD.0332991..Isethionate:Ldose: 0.301029996 (uM) Interaction | 22.594981 | 15.13733352 | 1.492665862 | 0.135538823 |
| SKMEL2:Palbociclib..PD.0332991..Isethionate:Ldose: 0.301029996 (uM) Interaction | 18.42130818 | 15.31686613 | 1.202681282 | 0.229112452 |
| UACC0257:Palbociclib..PD.0332991..Isethionate:Ldose: 0.301029996 (uM) Interaction | 2.607014518 | 15.05685414 | 0.173144702 | 0.86253926 |
| MeWo:Pazopanib.HCl:Ldose: 0.301029996 (uM) Interaction | 15.08722579 | 15.13733352 | 0.996689792 | 0.318925923 |
| SKMEL2:Pazopanib.HCl:Ldose: 0.301029996 (uM) Interaction | -0.36086571 | 15.31686613 | -0.023560022 | 0.981203772 |
| UACC0257:Pazopanib.HCl:Ldose: 0.301029996 (uM) Interaction | 1.329696438 | 15.05685414 | 0.088311703 | 0.92962973 |
| MeWo:PD325901:Ldose: 0.301029996 (uM) Interaction | -29.60159092 | 15.13733352 | -1.955535357 | 0.050532387 |
| SKMEL2:PD325901:Ldose: 0.301029996 (uM) Interaction | -43.71467594 | 15.31686613 | -2.854022198 | 0.004320929 |
| UACC0257:PD325901:Ldose: 0.301029996 (uM) Interaction | 1.784490273 | 15.05685414 | 0.118516807 | 0.905659247 |
| SKMEL2:Pemetrexed:Ldose: 0.301029996 (uM) Interaction | 1.861489913 | 15.31686613 | 0.121532035 | 0.903270738 |
| MeWo:Plicamycin:Ldose: 0.301029996 (uM) Interaction | 1.847825416 | 15.13733352 | 0.122070734 | 0.902844099 |
| SKMEL2:Plicamycin:Ldose: 0.301029996 (uM) Interaction | -3.673798635 | 15.31686613 | -0.239853153 | 0.810446304 |
| UACC0257:Plicamycin:Ldose: 0.301029996 (uM) Interaction | -4.260463621 | 15.05685414 | -0.282958418 | 0.77721134 |
| MeWo:Pralatrexate:Ldose: 0.301029996 (uM) Interaction | 11.17990153 | 15.07335079 | 0.741699818 | 0.458277043 |
| SKMEL2:Pralatrexate:Ldose: 0.301029996 (uM) Interaction | -3.188403083 | 15.25363648 | -0.209025768 | 0.834430032 |
| MeWo:Quinacrine.HCl:Ldose: 0.301029996 (uM) Interaction | -13.23212363 | 15.13733352 | -0.874138342 | 0.382052334 |
| SKMEL2:Quinacrine.HCl:Ldose: 0.301029996 (uM) Interaction | -19.92518349 | 15.31686613 | -1.300865551 | 0.193317924 |
| UACC0257:Quinacrine.HCl:Ldose: 0.301029996 (uM) Interaction | -17.38743531 | 15.05685414 | -1.154785399 | 0.248190694 |
| MeWo:Quizartinib:Ldose: 0.301029996 (uM) Interaction | -2.23925079 | 15.13733352 | -0.147929012 | 0.88240013 |
| SKMEL2:Quizartinib:Ldose: 0.301029996 (uM) Interaction | 1.344572225 | 15.31686613 | 0.087783768 | 0.930049327 |
| UACC0257:Quizartinib:Ldose: 0.301029996 (uM) Interaction | -8.112507006 | 15.05685414 | -0.538791631 | 0.590036018 |
| MeWo:Raloxifene:Ldose: 0.301029996 (uM) Interaction | 14.12010248 | 15.07335079 | 0.936759363 | 0.348892499 |
| SKMEL2:Raloxifene:Ldose: 0.301029996 (uM) Interaction | 4.964845532 | 15.25363648 | 0.325486027 | 0.744816311 |
| MeWo:Romidepsin:Ldose: 0.301029996 (uM) Interaction | 10.41528776 | 15.13733352 | 0.688053001 | 0.491426564 |
| SKMEL2:Romidepsin:Ldose: 0.301029996 (uM) Interaction | 1.603316059 | 15.31686613 | 0.104676508 | 0.916633445 |
| UACC0257:Romidepsin:Ldose: 0.301029996 (uM) Interaction | -4.094324889 | 15.05685414 | -0.271924324 | 0.785682725 |
| MeWo:Sabutoclax..BI.97C1.:Ldose: 0.301029996 (uM) Interaction | 2.236318193 | 15.13733352 | 0.147735279 | 0.882553024 |
| SKMEL2:Sabutoclax..BI.97C1.:Ldose: 0.301029996 (uM) Interaction | -12.95127673 | 15.31686613 | -0.845556566 | 0.397809238 |
| UACC0257:Sabutoclax..BI.97C1.:Ldose: 0.301029996 (uM) Interaction | 27.41995504 | 15.05685414 | 1.821094552 | 0.068605881 |
| MeWo:Sirolimus..Rapamycin.:Ldose: 0.301029996 (uM) Interaction | 4.41080852 | 15.13733352 | 0.291386096 | 0.770758758 |
| SKMEL2:Sirolimus..Rapamycin.:Ldose: 0.301029996 (uM) Interaction | -14.74697683 | 15.31686613 | -0.962793349 | 0.335661681 |
| MeWo:Sorafenib:Ldose: 0.301029996 (uM) Interaction | 6.866013823 | 15.13733352 | 0.453581459 | 0.650134516 |
| UACC0257:Sorafenib:Ldose: 0.301029996 (uM) Interaction | 15.77113876 | 15.05685414 | 1.047439167 | 0.29490843 |
| MeWo:Streptozocin:Ldose: 0.301029996 (uM) Interaction | 8.541434318 | 15.13733352 | 0.564262808 | 0.572580965 |
| SKMEL2:Streptozocin:Ldose: 0.301029996 (uM) Interaction | -18.54116109 | 15.31686613 | -1.210506179 | 0.226097553 |
| UACC0257:Streptozocin:Ldose: 0.301029996 (uM) Interaction | 13.43621525 | 15.05685414 | 0.892365373 | 0.372206756 |
| MeWo:Sunitinib:Ldose: 0.301029996 (uM) Interaction | -4.045336179 | 15.13733352 | -0.267242323 | 0.789285063 |
| UACC0257:Sunitinib:Ldose: 0.301029996 (uM) Interaction | -16.46934723 | 15.05685414 | -1.093810638 | 0.274049855 |
| MeWo:Tamoxifen.Citrate:Ldose: 0.301029996 (uM) Interaction | 4.952874461 | 15.13733352 | 0.327195966 | 0.743522744 |
| SKMEL2:Tamoxifen.Citrate:Ldose: 0.301029996 (uM) Interaction | -3.813540489 | 15.31686613 | -0.24897655 | 0.803381214 |
| UACC0257:Tamoxifen.Citrate:Ldose: 0.301029996 (uM) Interaction | -6.802207841 | 15.05685414 | -0.451768197 | 0.651440378 |
| MeWo:Temozolomide:Ldose: 0.301029996 (uM) Interaction | 11.90245061 | 15.33308304 | 0.77625945 | 0.437604044 |
| SKMEL2:Temsirolimus..CCI.779..Torisel.:Ldose: 0.301029996 (uM) Interaction | -9.518170959 | 15.31686613 | -0.62141765 | 0.534331215 |
| MeWo:Teniposide:Ldose: 0.301029996 (uM) Interaction | 0.936852576 | 15.13733352 | 0.061890198 | 0.950650828 |
| SKMEL2:Teniposide:Ldose: 0.301029996 (uM) Interaction | -13.32111857 | 15.31686613 | -0.869702618 | 0.384472304 |
| UACC0257:Teniposide:Ldose: 0.301029996 (uM) Interaction | 10.77851814 | 15.05685414 | 0.715854589 | 0.474088638 |
| MeWo:Thioguanine:Ldose: 0.301029996 (uM) Interaction | 34.62040446 | 15.13733352 | 2.287087379 | 0.022200049 |
| SKMEL2:Thioguanine:Ldose: 0.301029996 (uM) Interaction | 57.81645017 | 15.31686613 | 3.774691878 | 0.000160621 |
| UACC0257:Thioguanine:Ldose: 0.301029996 (uM) Interaction | 43.59350922 | 15.05685414 | 2.895260113 | 0.003792122 |
| MeWo:Thiotepa:Ldose: 0.301029996 (uM) Interaction | 6.92825052 | 15.13733352 | 0.457692929 | 0.647177529 |
| SKMEL2:Thiotepa:Ldose: 0.301029996 (uM) Interaction | -4.160568579 | 15.31686613 | -0.271633149 | 0.785906623 |
| UACC0257:Thiotepa:Ldose: 0.301029996 (uM) Interaction | 18.17980425 | 15.05685414 | 1.207410531 | 0.22728689 |
| MeWo:Topotecan.HCl:Ldose: 0.301029996 (uM) Interaction | 53.27808102 | 15.13733352 | 3.519647693 | 0.000432982 |
| SKMEL2:Topotecan.HCl:Ldose: 0.301029996 (uM) Interaction | 58.29438242 | 15.31686613 | 3.805894883 | 0.00014167 |
| UACC0257:Topotecan.HCl:Ldose: 0.301029996 (uM) Interaction | 37.29111499 | 15.05685414 | 2.476687005 | 0.013268174 |
| MeWo:Trametinib..GSK1120212.:Ldose: 0.301029996 (uM) Interaction | 20.84119561 | 15.13733352 | 1.376807585 | 0.168585569 |
| UACC0257:Trametinib..GSK1120212.:Ldose: 0.301029996 (uM) Interaction | 6.051560408 | 15.05685414 | 0.401913996 | 0.687751175 |
| MeWo:Triethylenemelamine:Ldose: 0.301029996 (uM) Interaction | -3.076364599 | 15.13733352 | -0.203230284 | 0.838956898 |
| SKMEL2:Triethylenemelamine:Ldose: 0.301029996 (uM) Interaction | -11.17748397 | 15.31686613 | -0.72975006 | 0.465550642 |
| UACC0257:Triethylenemelamine:Ldose: 0.301029996 (uM) Interaction | 7.543006069 | 15.05685414 | 0.500968263 | 0.616398404 |
| MeWo:Uracil.mustard:Ldose: 0.301029996 (uM) Interaction | 2.188645053 | 15.13733352 | 0.144585904 | 0.885039134 |
| SKMEL2:Uracil.mustard:Ldose: 0.301029996 (uM) Interaction | -6.921299865 | 15.31686613 | -0.451874411 | 0.651363855 |
| MeWo:Valrubicin:Ldose: 0.301029996 (uM) Interaction | 58.36439817 | 15.13733352 | 3.855659128 | 0.000115742 |
| SKMEL2:Valrubicin:Ldose: 0.301029996 (uM) Interaction | 41.64001626 | 15.31686613 | 2.718572841 | 0.006561492 |
| UACC0257:Valrubicin:Ldose: 0.301029996 (uM) Interaction | 35.48501982 | 15.05685414 | 2.356735311 | 0.018444982 |
| MeWo:Vandetanib:Ldose: 0.301029996 (uM) Interaction | -3.183376901 | 15.07335079 | -0.211192385 | 0.832739083 |
| SKMEL2:Vandetanib:Ldose: 0.301029996 (uM) Interaction | -4.282241168 | 15.25363648 | -0.280735756 | 0.778915687 |
| SKMEL2:Vemurafenib:Ldose: 0.301029996 (uM) Interaction | 74.12593087 | 15.31686613 | 4.839497208 | 1.31E-06 |
| UACC0257:Vemurafenib:Ldose: 0.301029996 (uM) Interaction | 38.6667143 | 15.05685414 | 2.568047345 | 0.010233772 |
| SKMEL2:Vinblastine.Sulfate:Ldose: 0.301029996 (uM) Interaction | 1.988516618 | 15.31686613 | 0.129825292 | 0.896705823 |
| UACC0257:Vinblastine.Sulfate:Ldose: 0.301029996 (uM) Interaction | 30.08445418 | 15.05685414 | 1.998057091 | 0.045722595 |
| MeWo:Vincristine.Sulfate:Ldose: 0.301029996 (uM) Interaction | 34.4630563 | 15.13733352 | 2.276692671 | 0.022814018 |
| SKMEL2:Vincristine.Sulfate:Ldose: 0.301029996 (uM) Interaction | 46.59106028 | 15.31686613 | 3.04181416 | 0.002354303 |
| UACC0257:Vincristine.Sulfate:Ldose: 0.301029996 (uM) Interaction | 49.08671326 | 15.05685414 | 3.260090907 | 0.001115435 |
| MeWo:Vinorelbine.Tartrate:Ldose: 0.301029996 (uM) Interaction | 28.99532544 | 15.13733352 | 1.915484348 | 0.055443541 |
| SKMEL2:Vinorelbine.Tartrate:Ldose: 0.301029996 (uM) Interaction | 20.38589928 | 15.31686613 | 1.330944536 | 0.183220871 |
| UACC0257:Vinorelbine.Tartrate:Ldose: 0.301029996 (uM) Interaction | 26.47082096 | 15.05685414 | 1.758057873 | 0.078751382 |
| MeWo:Vismodegib:Ldose: 0.301029996 (uM) Interaction | 18.32519328 | 15.13733352 | 1.210595859 | 0.226063165 |
| SKMEL2:Vismodegib:Ldose: 0.301029996 (uM) Interaction | -9.658759982 | 15.31686613 | -0.630596357 | 0.528310945 |
| UACC0257:Vismodegib:Ldose: 0.301029996 (uM) Interaction | 22.71043633 | 15.05685414 | 1.508312169 | 0.131488819 |
| MeWo:Vorinostat:Ldose: 0.301029996 (uM) Interaction | -3.929725032 | 15.13733352 | -0.259604839 | 0.795171 |
| SKMEL2:Vorinostat:Ldose: 0.301029996 (uM) Interaction | -14.04681641 | 15.31686613 | -0.917081621 | 0.359109791 |
| UACC0257:Vorinostat:Ldose: 0.301029996 (uM) Interaction | 9.124715914 | 15.05685414 | 0.606017421 | 0.544509354 |
| MeWo:Zoledronic.Acid:Ldose: 0.301029996 (uM) Interaction | 15.32759678 | 15.33308304 | 0.999642195 | 0.317494544 |
| MeWo:Abiraterone:Ldose: 0.602059991 (uM) Interaction | 4.811930552 | 15.13733352 | 0.317884953 | 0.750575184 |
| SKMEL2:Abiraterone:Ldose: 0.602059991 (uM) Interaction | 1.749947571 | 15.31686613 | 0.114249714 | 0.909040873 |
| MeWo:ABT.737:Ldose: 0.602059991 (uM) Interaction | -5.426225885 | 15.13733352 | -0.358466428 | 0.719997681 |
| SKMEL2:ABT.737:Ldose: 0.602059991 (uM) Interaction | 11.50550684 | 15.31686613 | 0.751165855 | 0.452560775 |
| UACC0257:ABT.737:Ldose: 0.602059991 (uM) Interaction | -7.106169396 | 15.05685414 | -0.471955784 | 0.636962968 |
| MeWo:Actinomycin.D:Ldose: 0.602059991 (uM) Interaction | -25.52828219 | 15.13733352 | -1.686445116 | 0.091724088 |
| SKMEL2:Actinomycin.D:Ldose: 0.602059991 (uM) Interaction | -31.46509874 | 15.31686613 | -2.054277845 | 0.039960439 |
| UACC0257:Actinomycin.D:Ldose: 0.602059991 (uM) Interaction | -18.56626799 | 15.05685414 | -1.233077496 | 0.217559853 |
| MeWo:Afatinib:Ldose: 0.602059991 (uM) Interaction | 5.87865701 | 15.13733352 | 0.388354858 | 0.697757154 |
| SKMEL2:Afatinib:Ldose: 0.602059991 (uM) Interaction | 4.776295491 | 15.31686613 | 0.311832424 | 0.755170797 |
| UACC0257:Afatinib:Ldose: 0.602059991 (uM) Interaction | -2.776742471 | 15.05685414 | -0.184417173 | 0.853687876 |
| MeWo:Alisertib..MLN8237.:Ldose: 0.602059991 (uM) Interaction | 26.39978884 | 15.13733352 | 1.744018443 | 0.081169655 |
| SKMEL2:Alisertib..MLN8237.:Ldose: 0.602059991 (uM) Interaction | 28.16675426 | 15.31686613 | 1.838937158 | 0.06593772 |
| MeWo:Allopurinol:Ldose: 0.602059991 (uM) Interaction | 1.461287618 | 15.13733352 | 0.096535339 | 0.923096276 |
| SKMEL2:Allopurinol:Ldose: 0.602059991 (uM) Interaction | -6.356297854 | 15.31686613 | -0.414986839 | 0.678155531 |
| UACC0257:Allopurinol:Ldose: 0.602059991 (uM) Interaction | 4.247960499 | 15.05685414 | 0.282128024 | 0.777847965 |
| MeWo:Amifostine:Ldose: 0.602059991 (uM) Interaction | 2.808404268 | 15.13733352 | 0.185528334 | 0.852816346 |
| MeWo:Aphrocallistin.analogue:Ldose: 0.602059991 (uM) Interaction | 42.02555463 | 15.13733352 | 2.776285173 | 0.005502981 |
| SKMEL2:Aphrocallistin.analogue:Ldose: 0.602059991 (uM) Interaction | 55.10930196 | 15.31686613 | 3.597948921 | 0.000321431 |
| UACC0257:Aphrocallistin.analogue:Ldose: 0.602059991 (uM) Interaction | 49.76025476 | 15.05685414 | 3.304824122 | 0.000951859 |
| MeWo:Arsenic.Trioxide:Ldose: 0.602059991 (uM) Interaction | 8.396070635 | 15.13733352 | 0.554659817 | 0.57913293 |
| SKMEL2:Arsenic.Trioxide:Ldose: 0.602059991 (uM) Interaction | 0.903841518 | 15.31686613 | 0.059009559 | 0.952945023 |
| MeWo:Axitinib:Ldose: 0.602059991 (uM) Interaction | 4.858366645 | 15.13733352 | 0.320952606 | 0.748249315 |
| SKMEL2:Axitinib:Ldose: 0.602059991 (uM) Interaction | -4.525127214 | 15.31686613 | -0.295434273 | 0.767664906 |
| UACC0257:Axitinib:Ldose: 0.602059991 (uM) Interaction | -3.293178466 | 15.05685414 | -0.218716236 | 0.826873102 |
| MeWo:Axitinib.1:Ldose: 0.602059991 (uM) Interaction | 19.94024854 | 15.13733352 | 1.31728937 | 0.187755184 |
| SKMEL2:Axitinib.1:Ldose: 0.602059991 (uM) Interaction | 22.58946168 | 15.31686613 | 1.474809631 | 0.140277851 |
| UACC0257:Axitinib.1:Ldose: 0.602059991 (uM) Interaction | 1.588639691 | 15.05685414 | 0.105509403 | 0.915972558 |
| MeWo:Azacitidine:Ldose: 0.602059991 (uM) Interaction | 2.02621317 | 15.13733352 | 0.133855356 | 0.893518161 |
| SKMEL2:Azacitidine:Ldose: 0.602059991 (uM) Interaction | -6.33663434 | 15.31686613 | -0.413703057 | 0.679095567 |
| UACC0257:Azacitidine:Ldose: 0.602059991 (uM) Interaction | 17.00664298 | 15.05685414 | 1.1294951 | 0.258701187 |
| SKMEL2:Baricitinib..LY3009104..INCB028050.:Ldose: 0.602059991 (uM) Interaction | 31.89023415 | 15.31686613 | 2.082033876 | 0.037350751 |
| UACC0257:Baricitinib..LY3009104..INCB028050.:Ldose: 0.602059991 (uM) Interaction | 7.064645457 | 15.05685414 | 0.469197974 | 0.638932721 |
| SKMEL2:Bendamustine.HCl:Ldose: 0.602059991 (uM) Interaction | 12.70809249 | 15.25363648 | 0.833118877 | 0.404786613 |
| MeWo:Bioymifi:Ldose: 0.602059991 (uM) Interaction | -7.022033802 | 15.13733352 | -0.463888425 | 0.642732215 |
| SKMEL2:Bioymifi:Ldose: 0.602059991 (uM) Interaction | 0.435049566 | 15.31686613 | 0.028403301 | 0.977340745 |
| UACC0257:Bioymifi:Ldose: 0.602059991 (uM) Interaction | -10.06816762 | 15.05685414 | -0.668676706 | 0.503708653 |
| MeWo:Bleomycin.Sulfate:Ldose: 0.602059991 (uM) Interaction | 24.63480367 | 15.13733352 | 1.627420287 | 0.103661996 |
| SKMEL2:Bleomycin.Sulfate:Ldose: 0.602059991 (uM) Interaction | 40.13776613 | 15.31686613 | 2.620494676 | 0.008786183 |
| UACC0257:Bleomycin.Sulfate:Ldose: 0.602059991 (uM) Interaction | 54.64565783 | 15.05685414 | 3.629287853 | 0.00028484 |
| MeWo:Bortezomib:Ldose: 0.602059991 (uM) Interaction | 9.440852206 | 15.13733352 | 0.623680002 | 0.532844139 |
| SKMEL2:Bortezomib:Ldose: 0.602059991 (uM) Interaction | 15.74308283 | 15.31686613 | 1.027826626 | 0.304042539 |
| UACC0257:Bortezomib:Ldose: 0.602059991 (uM) Interaction | 19.95277594 | 15.05685414 | 1.325162332 | 0.185130895 |
| MeWo:Bosutinib..SKI.606.:Ldose: 0.602059991 (uM) Interaction | 14.70994155 | 15.13733352 | 0.971765703 | 0.331177664 |
| SKMEL2:Bosutinib..SKI.606.:Ldose: 0.602059991 (uM) Interaction | 26.34413925 | 15.31686613 | 1.719943168 | 0.08545665 |
| UACC0257:Bosutinib..SKI.606.:Ldose: 0.602059991 (uM) Interaction | 4.516736757 | 15.05685414 | 0.299978781 | 0.764196137 |
| SKMEL2:Busulfan:Ldose: 0.602059991 (uM) Interaction | 9.415894468 | 15.31686613 | 0.614740273 | 0.538732532 |
| MeWo:Cabazitaxel:Ldose: 0.602059991 (uM) Interaction | 8.705634017 | 15.13733352 | 0.575110141 | 0.565222613 |
| SKMEL2:Cabazitaxel:Ldose: 0.602059991 (uM) Interaction | -2.006088554 | 15.31686613 | -0.13097252 | 0.895798228 |
| UACC0257:Cabazitaxel:Ldose: 0.602059991 (uM) Interaction | 0.353376781 | 15.05685414 | 0.023469496 | 0.98127598 |
| MeWo:Cabozantinib..XL.184.:Ldose: 0.602059991 (uM) Interaction | 7.02095412 | 15.13733352 | 0.463817099 | 0.64278332 |
| SKMEL2:Cabozantinib..XL.184.:Ldose: 0.602059991 (uM) Interaction | 26.51061862 | 15.31686613 | 1.730812191 | 0.083499102 |
| UACC0257:Cabozantinib..XL.184.:Ldose: 0.602059991 (uM) Interaction | -9.414263536 | 15.05685414 | -0.625247708 | 0.531814894 |
| MeWo:Capecitabine:Ldose: 0.602059991 (uM) Interaction | 0.81840973 | 15.33308304 | 0.053375419 | 0.957433268 |
| MeWo:Carfilzomib:Ldose: 0.602059991 (uM) Interaction | 5.580845257 | 15.13733352 | 0.368680868 | 0.712369112 |
| SKMEL2:Carfilzomib:Ldose: 0.602059991 (uM) Interaction | 66.01789474 | 15.31686613 | 4.310143745 | 1.64E-05 |
| UACC0257:Carfilzomib:Ldose: 0.602059991 (uM) Interaction | -39.03665111 | 15.05685414 | -2.592616675 | 0.009531104 |
| MeWo:Carmustine:Ldose: 0.602059991 (uM) Interaction | -2.64917259 | 15.33308304 | -0.172774946 | 0.8628299 |
| MeWo:Celecoxib:Ldose: 0.602059991 (uM) Interaction | -1.026889701 | 15.33308304 | -0.066972161 | 0.946604466 |
| UACC0257:Chlorambucil:Ldose: 0.602059991 (uM) Interaction | -5.324589843 | 15.05685414 | -0.353632292 | 0.723617814 |
| MeWo:Cisplatin:Ldose: 0.602059991 (uM) Interaction | 5.275098352 | 15.13733352 | 0.348482667 | 0.727481019 |
| SKMEL2:Cisplatin:Ldose: 0.602059991 (uM) Interaction | 6.52803906 | 15.31686613 | 0.426199394 | 0.669966674 |
| MeWo:Cladribine:Ldose: 0.602059991 (uM) Interaction | 51.04954741 | 15.13733352 | 3.372426678 | 0.000746356 |
| SKMEL2:Cladribine:Ldose: 0.602059991 (uM) Interaction | 39.38842402 | 15.31686613 | 2.571571998 | 0.010130215 |
| UACC0257:Cladribine:Ldose: 0.602059991 (uM) Interaction | 20.4233163 | 15.05685414 | 1.35641324 | 0.174981458 |
| MeWo:Clofarabine:Ldose: 0.602059991 (uM) Interaction | 46.26984137 | 15.13733352 | 3.056670535 | 0.002240749 |
| SKMEL2:Clofarabine:Ldose: 0.602059991 (uM) Interaction | 37.83294068 | 15.31686613 | 2.470018369 | 0.01351804 |
| UACC0257:Clofarabine:Ldose: 0.602059991 (uM) Interaction | -0.165696548 | 15.05685414 | -0.011004726 | 0.991219775 |
| MeWo:Crizotinib:Ldose: 0.602059991 (uM) Interaction | 5.456627803 | 15.13733352 | 0.360474835 | 0.718495487 |
| SKMEL2:Crizotinib:Ldose: 0.602059991 (uM) Interaction | 5.538371253 | 15.31686613 | 0.36158645 | 0.717664518 |
| UACC0257:Crizotinib:Ldose: 0.602059991 (uM) Interaction | 8.096696942 | 15.05685414 | 0.537741607 | 0.590760817 |
| MeWo:Cytarabine.HCl...Ara.C:Ldose: 0.602059991 (uM) Interaction | 63.73750487 | 15.13733352 | 4.210616405 | 2.56E-05 |
| SKMEL2:Cytarabine.HCl...Ara.C:Ldose: 0.602059991 (uM) Interaction | 68.09739194 | 15.31686613 | 4.445908933 | 8.79E-06 |
| UACC0257:Cytarabine.HCl...Ara.C:Ldose: 0.602059991 (uM) Interaction | 44.74560182 | 15.05685414 | 2.97177627 | 0.002963984 |
| MeWo:Dacarbazine:Ldose: 0.602059991 (uM) Interaction | 22.20516889 | 15.13733352 | 1.46691416 | 0.142413512 |
| SKMEL2:Dacarbazine:Ldose: 0.602059991 (uM) Interaction | 25.88374198 | 15.31686613 | 1.689884978 | 0.091063942 |
| MeWo:Dacomitinib..PF299804.:Ldose: 0.602059991 (uM) Interaction | -6.406583118 | 15.13733352 | -0.423230624 | 0.672131087 |
| SKMEL2:Dacomitinib..PF299804.:Ldose: 0.602059991 (uM) Interaction | 0.403131175 | 15.31686613 | 0.026319429 | 0.979002794 |
| UACC0257:Dacomitinib..PF299804.:Ldose: 0.602059991 (uM) Interaction | 0.096266337 | 15.05685414 | 0.006393523 | 0.994898799 |
| MeWo:Dasatinib:Ldose: 0.602059991 (uM) Interaction | -8.383348286 | 15.13733352 | -0.553819355 | 0.579708035 |
| SKMEL2:Dasatinib:Ldose: 0.602059991 (uM) Interaction | -8.565115808 | 15.31686613 | -0.559195056 | 0.576034224 |
| UACC0257:Dasatinib:Ldose: 0.602059991 (uM) Interaction | 3.825571949 | 15.05685414 | 0.254075115 | 0.799439871 |
| MeWo:Daunorubicin.HCl:Ldose: 0.602059991 (uM) Interaction | -17.35394211 | 15.13733352 | -1.146433227 | 0.251628244 |
| SKMEL2:Daunorubicin.HCl:Ldose: 0.602059991 (uM) Interaction | 22.70031105 | 15.31686613 | 1.48204671 | 0.138342 |
| UACC0257:Daunorubicin.HCl:Ldose: 0.602059991 (uM) Interaction | -15.18794926 | 15.05685414 | -1.008706674 | 0.313126277 |
| MeWo:Decitabine:Ldose: 0.602059991 (uM) Interaction | 3.180992932 | 15.13733352 | 0.210142224 | 0.833558591 |
| SKMEL2:Decitabine:Ldose: 0.602059991 (uM) Interaction | 2.925583263 | 15.31686613 | 0.191004037 | 0.84852417 |
| MeWo:Docetaxel:Ldose: 0.602059991 (uM) Interaction | 9.170053602 | 15.13733352 | 0.60579055 | 0.544660012 |
| MeWo:Doxorubicin.HCl:Ldose: 0.602059991 (uM) Interaction | -2.878549832 | 15.13733352 | -0.190162278 | 0.8491837 |
| SKMEL2:Doxorubicin.HCl:Ldose: 0.602059991 (uM) Interaction | -9.246993825 | 15.31686613 | -0.603713171 | 0.546040498 |
| UACC0257:Doxorubicin.HCl:Ldose: 0.602059991 (uM) Interaction | -51.30896362 | 15.05685414 | -3.407681521 | 0.000656331 |
| MeWo:Erlotinib.HCl:Ldose: 0.602059991 (uM) Interaction | -3.21893351 | 15.13733352 | -0.212648648 | 0.831602967 |
| SKMEL2:Erlotinib.HCl:Ldose: 0.602059991 (uM) Interaction | 10.33763165 | 15.31686613 | 0.674918196 | 0.499734738 |
| UACC0257:Erlotinib.HCl:Ldose: 0.602059991 (uM) Interaction | 1.650294484 | 15.05685414 | 0.109604202 | 0.912724264 |
| MeWo:Etoposide:Ldose: 0.602059991 (uM) Interaction | 47.00746448 | 15.13733352 | 3.10539927 | 0.001902605 |
| SKMEL2:Etoposide:Ldose: 0.602059991 (uM) Interaction | 49.84082641 | 15.31686613 | 3.253983288 | 0.001139684 |
| UACC0257:Etoposide:Ldose: 0.602059991 (uM) Interaction | 34.49247252 | 15.05685414 | 2.290815346 | 0.021983382 |
| MeWo:Everolimus:Ldose: 0.602059991 (uM) Interaction | 3.006373652 | 15.33308304 | 0.196071047 | 0.844556344 |
| MeWo:Exemestane:Ldose: 0.602059991 (uM) Interaction | 5.407435695 | 15.07335079 | 0.358741448 | 0.719791915 |
| SKMEL2:Exemestane:Ldose: 0.602059991 (uM) Interaction | 12.25510143 | 15.25363648 | 0.803421627 | 0.421739644 |
| MeWo:Floxuridine:Ldose: 0.602059991 (uM) Interaction | 13.73230236 | 15.13733352 | 0.907181066 | 0.364320859 |
| SKMEL2:Floxuridine:Ldose: 0.602059991 (uM) Interaction | 75.92648721 | 15.31686613 | 4.957051043 | 7.21E-07 |
| UACC0257:Floxuridine:Ldose: 0.602059991 (uM) Interaction | 7.024515122 | 15.05685414 | 0.46653272 | 0.64083879 |
| MeWo:Fludarabine.Phosphate:Ldose: 0.602059991 (uM) Interaction | 2.861839375 | 15.13733352 | 0.189058355 | 0.850048802 |
| SKMEL2:Fludarabine.Phosphate:Ldose: 0.602059991 (uM) Interaction | 17.36148397 | 15.31686613 | 1.133488001 | 0.257021565 |
| MeWo:Fluorouracil...5.FU.:Ldose: 0.602059991 (uM) Interaction | -9.654020417 | 15.13733352 | -0.637762285 | 0.523634983 |
| SKMEL2:Fluorouracil...5.FU.:Ldose: 0.602059991 (uM) Interaction | 4.32692343 | 15.31686613 | 0.282494042 | 0.777567336 |
| UACC0257:Fluorouracil...5.FU.:Ldose: 0.602059991 (uM) Interaction | -8.567733944 | 15.05685414 | -0.569025499 | 0.569344567 |
| MeWo:Flutamide..Eulexin.:Ldose: 0.602059991 (uM) Interaction | 3.754963385 | 15.13733352 | 0.248059764 | 0.804090451 |
| SKMEL2:Flutamide..Eulexin.:Ldose: 0.602059991 (uM) Interaction | 8.437179968 | 15.31686613 | 0.550842444 | 0.581747205 |
| UACC0257:Flutamide..Eulexin.:Ldose: 0.602059991 (uM) Interaction | -5.617042659 | 15.05685414 | -0.373055527 | 0.709110664 |
| MeWo:Foretinib..GSK1363089.:Ldose: 0.602059991 (uM) Interaction | 43.48243431 | 15.13733352 | 2.872529316 | 0.004075844 |
| SKMEL2:Foretinib..GSK1363089.:Ldose: 0.602059991 (uM) Interaction | 87.77645973 | 15.31686613 | 5.730706203 | 1.01E-08 |
| UACC0257:Foretinib..GSK1363089.:Ldose: 0.602059991 (uM) Interaction | 15.96570161 | 15.05685414 | 1.060361046 | 0.288991863 |
| SKMEL2:Fulvestrant:Ldose: 0.602059991 (uM) Interaction | -1.683321419 | 15.31686613 | -0.109899858 | 0.912489784 |
| UACC0257:Fulvestrant:Ldose: 0.602059991 (uM) Interaction | 10.74879212 | 15.05685414 | 0.713880338 | 0.475308649 |
| MeWo:Gefitinib:Ldose: 0.602059991 (uM) Interaction | 7.330034215 | 15.33308304 | 0.478053513 | 0.632616805 |
| MeWo:Gemcitabine.HCl:Ldose: 0.602059991 (uM) Interaction | 33.59571389 | 15.13733352 | 2.219394442 | 0.026469926 |
| SKMEL2:Gemcitabine.HCl:Ldose: 0.602059991 (uM) Interaction | 73.57469851 | 15.31686613 | 4.803508622 | 1.57E-06 |
| UACC0257:Gemcitabine.HCl:Ldose: 0.602059991 (uM) Interaction | -10.27970855 | 15.05685414 | -0.682726183 | 0.494786975 |
| MeWo:Ibrutinib..PCI.32765.:Ldose: 0.602059991 (uM) Interaction | 4.905621508 | 15.07335079 | 0.325449966 | 0.744843598 |
| SKMEL2:Ibrutinib..PCI.32765.:Ldose: 0.602059991 (uM) Interaction | 5.003211419 | 15.25363648 | 0.328001223 | 0.742913818 |
| MeWo:Imiquimod:Ldose: 0.602059991 (uM) Interaction | -11.19143204 | 15.07335079 | -0.742464777 | 0.457813607 |
| SKMEL2:Imiquimod:Ldose: 0.602059991 (uM) Interaction | -8.660093276 | 15.25363648 | -0.567739587 | 0.570217523 |
| MeWo:INK.128..MLN0128.:Ldose: 0.602059991 (uM) Interaction | 28.00286807 | 15.13733352 | 1.849920795 | 0.064338189 |
| SKMEL2:INK.128..MLN0128.:Ldose: 0.602059991 (uM) Interaction | 6.481655075 | 15.31686613 | 0.423171099 | 0.672174513 |
| UACC0257:INK.128..MLN0128.:Ldose: 0.602059991 (uM) Interaction | 12.4911131 | 15.05685414 | 0.829596474 | 0.406775853 |
| MeWo:Irinotecan.HCl:Ldose: 0.602059991 (uM) Interaction | 51.60103246 | 15.13733352 | 3.408858791 | 0.000653506 |
| SKMEL2:Irinotecan.HCl:Ldose: 0.602059991 (uM) Interaction | 35.36353458 | 15.31686613 | 2.308797001 | 0.020963948 |
| UACC0257:Irinotecan.HCl:Ldose: 0.602059991 (uM) Interaction | 33.56828892 | 15.05685414 | 2.229435751 | 0.025794847 |
| MeWo:Ixabepilone:Ldose: 0.602059991 (uM) Interaction | 24.5680876 | 15.13733352 | 1.623012901 | 0.104600785 |
| SKMEL2:Ixabepilone:Ldose: 0.602059991 (uM) Interaction | 25.29788507 | 15.31686613 | 1.651635841 | 0.098622891 |
| UACC0257:Ixabepilone:Ldose: 0.602059991 (uM) Interaction | 26.55137637 | 15.05685414 | 1.763407956 | 0.077845404 |
| MeWo:Lapatinib:Ldose: 0.602059991 (uM) Interaction | -12.11931199 | 15.07335079 | -0.804022421 | 0.421392599 |
| SKMEL2:Lapatinib:Ldose: 0.602059991 (uM) Interaction | -16.60135691 | 15.25363648 | -1.088354042 | 0.276450606 |
| MeWo:LDK378:Ldose: 0.602059991 (uM) Interaction | 2.658056062 | 15.13733352 | 0.175596056 | 0.860612896 |
| SKMEL2:LDK378:Ldose: 0.602059991 (uM) Interaction | 24.65729979 | 15.31686613 | 1.609813626 | 0.107452707 |
| UACC0257:LDK378:Ldose: 0.602059991 (uM) Interaction | 23.03544782 | 15.05685414 | 1.529897787 | 0.126056218 |
| MeWo:Lenalidomide:Ldose: 0.602059991 (uM) Interaction | 3.553229345 | 15.13733352 | 0.234732844 | 0.81441825 |
| SKMEL2:Lenalidomide:Ldose: 0.602059991 (uM) Interaction | -3.846434634 | 15.31686613 | -0.251124127 | 0.801720457 |
| SKMEL2:Letrozole:Ldose: 0.602059991 (uM) Interaction | 6.767066766 | 15.31686613 | 0.441804917 | 0.658634665 |
| MeWo:Linsitinib:Ldose: 0.602059991 (uM) Interaction | 7.820531643 | 15.13733352 | 0.516638656 | 0.60541355 |
| SKMEL2:Linsitinib:Ldose: 0.602059991 (uM) Interaction | 4.439457036 | 15.31686613 | 0.289841081 | 0.771940513 |
| UACC0257:Linsitinib:Ldose: 0.602059991 (uM) Interaction | -4.104533284 | 15.05685414 | -0.272602314 | 0.785161457 |
| SKMEL2:Lomustine..CCNU.:Ldose: 0.602059991 (uM) Interaction | -2.395843995 | 15.31686613 | -0.156418681 | 0.875704429 |
| MeWo:LY2157299:Ldose: 0.602059991 (uM) Interaction | 2.974489475 | 15.13733352 | 0.196500227 | 0.844220446 |
| SKMEL2:LY2157299:Ldose: 0.602059991 (uM) Interaction | 9.944648748 | 15.31686613 | 0.649261322 | 0.516176169 |
| MeWo:Mechlorethamine.HCl:Ldose: 0.602059991 (uM) Interaction | -5.732015947 | 15.13733352 | -0.37866748 | 0.70493841 |
| SKMEL2:Mechlorethamine.HCl:Ldose: 0.602059991 (uM) Interaction | -0.447704534 | 15.31686613 | -0.029229513 | 0.976681805 |
| UACC0257:Mechlorethamine.HCl:Ldose: 0.602059991 (uM) Interaction | 1.164415752 | 15.05685414 | 0.077334597 | 0.938358063 |
| MeWo:Megestrol.acetate:Ldose: 0.602059991 (uM) Interaction | 6.4817918 | 15.13733352 | 0.428199048 | 0.668510346 |
| SKMEL2:Megestrol.acetate:Ldose: 0.602059991 (uM) Interaction | 3.293527528 | 15.31686613 | 0.215026201 | 0.829748858 |
| MeWo:MEK.162..ARRY.438162.:Ldose: 0.602059991 (uM) Interaction | 16.78524285 | 15.13733352 | 1.108863911 | 0.267500855 |
| SKMEL2:MEK.162..ARRY.438162.:Ldose: 0.602059991 (uM) Interaction | 24.84583795 | 15.31686613 | 1.622122812 | 0.104791194 |
| UACC0257:MEK.162..ARRY.438162.:Ldose: 0.602059991 (uM) Interaction | 28.59182483 | 15.05685414 | 1.898924209 | 0.057587338 |
| MeWo:Melphalan:Ldose: 0.602059991 (uM) Interaction | 8.007229563 | 15.13733352 | 0.528972263 | 0.596829957 |
| SKMEL2:Melphalan:Ldose: 0.602059991 (uM) Interaction | -5.544333662 | 15.31686613 | -0.361975721 | 0.717373604 |
| UACC0257:Melphalan:Ldose: 0.602059991 (uM) Interaction | 5.185162346 | 15.05685414 | 0.344372224 | 0.730569623 |
| MeWo:Mercaptopurine:Ldose: 0.602059991 (uM) Interaction | 28.42002328 | 15.13733352 | 1.877478833 | 0.06046554 |
| SKMEL2:Mercaptopurine:Ldose: 0.602059991 (uM) Interaction | 61.49729772 | 15.31686613 | 4.015005237 | 5.96E-05 |
| UACC0257:Mercaptopurine:Ldose: 0.602059991 (uM) Interaction | 26.53895848 | 15.05685414 | 1.762583222 | 0.077984508 |
| MeWo:Mitomycin.C:Ldose: 0.602059991 (uM) Interaction | 39.83169742 | 15.13733352 | 2.631354945 | 0.008510368 |
| SKMEL2:Mitomycin.C:Ldose: 0.602059991 (uM) Interaction | 48.79401961 | 15.31686613 | 3.185639883 | 0.001446331 |
| UACC0257:Mitomycin.C:Ldose: 0.602059991 (uM) Interaction | -8.048232463 | 15.05685414 | -0.534522842 | 0.59298518 |
| MeWo:Mitotane..o.p..DDD..Lysodren.:Ldose: 0.602059991 (uM) Interaction | 10.62360691 | 15.13733352 | 0.701814946 | 0.482801902 |
| SKMEL2:Mitotane..o.p..DDD..Lysodren.:Ldose: 0.602059991 (uM) Interaction | -8.164020904 | 15.31686613 | -0.533008569 | 0.594032961 |
| MeWo:Mitoxantrone:Ldose: 0.602059991 (uM) Interaction | 18.25423835 | 15.13733352 | 1.205908446 | 0.22786559 |
| SKMEL2:Mitoxantrone:Ldose: 0.602059991 (uM) Interaction | 52.49559075 | 15.31686613 | 3.427306233 | 0.000610702 |
| UACC0257:Mitoxantrone:Ldose: 0.602059991 (uM) Interaction | 39.5588917 | 15.05685414 | 2.62730125 | 0.0086124 |
| UACC0257:MLN.2480:Ldose: 0.602059991 (uM) Interaction | -6.671846626 | 15.05685414 | -0.443110265 | 0.65769028 |
| MeWo:MLN4924:Ldose: 0.602059991 (uM) Interaction | 33.5297972 | 15.13733352 | 2.215039864 | 0.026767397 |
| SKMEL2:MLN4924:Ldose: 0.602059991 (uM) Interaction | 41.49133898 | 15.31686613 | 2.708866072 | 0.006756513 |
| UACC0257:MLN4924:Ldose: 0.602059991 (uM) Interaction | 49.5158896 | 15.05685414 | 3.288594626 | 0.001008448 |
| MeWo:MLN9708..MLN2238.:Ldose: 0.602059991 (uM) Interaction | -14.69518368 | 15.13733352 | -0.970790771 | 0.331663011 |
| SKMEL2:MLN9708..MLN2238.:Ldose: 0.602059991 (uM) Interaction | 23.1704604 | 15.31686613 | 1.512741589 | 0.130359485 |
| UACC0257:MLN9708..MLN2238.:Ldose: 0.602059991 (uM) Interaction | -3.48845103 | 15.05685414 | -0.231685251 | 0.816784614 |
| MeWo:Navitoclax..ABT.263..5uM:Ldose: 0.602059991 (uM) Interaction | -4.548111053 | 15.13733352 | -0.300456553 | 0.763831733 |
| SKMEL2:Navitoclax..ABT.263..5uM:Ldose: 0.602059991 (uM) Interaction | 9.859973835 | 15.31686613 | 0.643733108 | 0.519755138 |
| UACC0257:Navitoclax..ABT.263..5uM:Ldose: 0.602059991 (uM) Interaction | 18.67917623 | 15.05685414 | 1.240576289 | 0.21477537 |
| MeWo:Nelarabine:Ldose: 0.602059991 (uM) Interaction | 1.640399672 | 15.13733352 | 0.108367809 | 0.91370491 |
| SKMEL2:Nelarabine:Ldose: 0.602059991 (uM) Interaction | -12.00709132 | 15.31686613 | -0.783913055 | 0.433099456 |
| UACC0257:Nelarabine:Ldose: 0.602059991 (uM) Interaction | -3.551907769 | 15.05685414 | -0.235899726 | 0.813512647 |
| MeWo:OSI.027:Ldose: 0.602059991 (uM) Interaction | -28.59088767 | 15.13733352 | -1.888766448 | 0.058936098 |
| SKMEL2:OSI.027:Ldose: 0.602059991 (uM) Interaction | 17.71559528 | 15.31686613 | 1.156607045 | 0.247445335 |
| UACC0257:OSI.027:Ldose: 0.602059991 (uM) Interaction | -13.83494502 | 15.05685414 | -0.918846985 | 0.358185553 |
| MeWo:Oxaliplatin:Ldose: 0.602059991 (uM) Interaction | -5.194925739 | 15.07335079 | -0.34464306 | 0.73036598 |
| SKMEL2:Oxaliplatin:Ldose: 0.602059991 (uM) Interaction | 6.243353156 | 15.25363648 | 0.409302606 | 0.682321535 |
| MeWo:Paclitaxel:Ldose: 0.602059991 (uM) Interaction | 44.84458136 | 15.13733352 | 2.962515247 | 0.003054587 |
| SKMEL2:Paclitaxel:Ldose: 0.602059991 (uM) Interaction | 53.52372668 | 15.31686613 | 3.494430664 | 0.000475996 |
| MeWo:Palbociclib..PD.0332991..Isethionate:Ldose: 0.602059991 (uM) Interaction | 14.58417755 | 15.13733352 | 0.963457503 | 0.33532843 |
| SKMEL2:Palbociclib..PD.0332991..Isethionate:Ldose: 0.602059991 (uM) Interaction | 32.77606672 | 15.31686613 | 2.139867676 | 0.032376284 |
| UACC0257:Palbociclib..PD.0332991..Isethionate:Ldose: 0.602059991 (uM) Interaction | 15.46591298 | 15.05685414 | 1.027167617 | 0.304352687 |
| MeWo:Pazopanib.HCl:Ldose: 0.602059991 (uM) Interaction | 12.29069657 | 15.13733352 | 0.811945945 | 0.416831318 |
| SKMEL2:Pazopanib.HCl:Ldose: 0.602059991 (uM) Interaction | 13.40461392 | 15.31686613 | 0.875153821 | 0.381499643 |
| UACC0257:Pazopanib.HCl:Ldose: 0.602059991 (uM) Interaction | 5.256405277 | 15.05685414 | 0.349103819 | 0.727014667 |
| MeWo:PD325901:Ldose: 0.602059991 (uM) Interaction | -33.36833285 | 15.13733352 | -2.204373234 | 0.027508284 |
| SKMEL2:PD325901:Ldose: 0.602059991 (uM) Interaction | -36.03788606 | 15.31686613 | -2.352823727 | 0.018640104 |
| UACC0257:PD325901:Ldose: 0.602059991 (uM) Interaction | 5.448163921 | 15.05685414 | 0.361839457 | 0.717475434 |
| SKMEL2:Pemetrexed:Ldose: 0.602059991 (uM) Interaction | 1.356241743 | 15.31686613 | 0.088545642 | 0.929443805 |
| MeWo:Plicamycin:Ldose: 0.602059991 (uM) Interaction | -0.29255629 | 15.13733352 | -0.019326805 | 0.984580574 |
| SKMEL2:Plicamycin:Ldose: 0.602059991 (uM) Interaction | 1.764031588 | 15.31686613 | 0.115169224 | 0.908312029 |
| UACC0257:Plicamycin:Ldose: 0.602059991 (uM) Interaction | 12.80879373 | 15.05685414 | 0.850695213 | 0.394947819 |
| MeWo:Pralatrexate:Ldose: 0.602059991 (uM) Interaction | 10.56079515 | 15.07335079 | 0.700626908 | 0.483543193 |
| SKMEL2:Pralatrexate:Ldose: 0.602059991 (uM) Interaction | 7.902108828 | 15.25363648 | 0.518047538 | 0.604430242 |
| MeWo:Quinacrine.HCl:Ldose: 0.602059991 (uM) Interaction | -11.74647662 | 15.13733352 | -0.775993777 | 0.437760891 |
| SKMEL2:Quinacrine.HCl:Ldose: 0.602059991 (uM) Interaction | -18.14696802 | 15.31686613 | -1.184770296 | 0.236120909 |
| UACC0257:Quinacrine.HCl:Ldose: 0.602059991 (uM) Interaction | -6.569229213 | 15.05685414 | -0.436294936 | 0.66262698 |
| MeWo:Quizartinib:Ldose: 0.602059991 (uM) Interaction | 0.279347102 | 15.13733352 | 0.018454182 | 0.985276694 |
| SKMEL2:Quizartinib:Ldose: 0.602059991 (uM) Interaction | 7.839345408 | 15.31686613 | 0.511811316 | 0.608788139 |
| UACC0257:Quizartinib:Ldose: 0.602059991 (uM) Interaction | -10.59923638 | 15.05685414 | -0.703947603 | 0.481472757 |
| MeWo:Raloxifene:Ldose: 0.602059991 (uM) Interaction | 7.468535321 | 15.07335079 | 0.495479434 | 0.620266619 |
| SKMEL2:Raloxifene:Ldose: 0.602059991 (uM) Interaction | 6.665825133 | 15.25363648 | 0.436999082 | 0.662116246 |
| MeWo:Romidepsin:Ldose: 0.602059991 (uM) Interaction | 10.48242282 | 15.13733352 | 0.692488066 | 0.48863809 |
| SKMEL2:Romidepsin:Ldose: 0.602059991 (uM) Interaction | 6.338119723 | 15.31686613 | 0.413800034 | 0.679024539 |
| UACC0257:Romidepsin:Ldose: 0.602059991 (uM) Interaction | -8.852131254 | 15.05685414 | -0.587913728 | 0.556596156 |
| MeWo:Sabutoclax..BI.97C1.:Ldose: 0.602059991 (uM) Interaction | 56.10875131 | 15.13733352 | 3.706646961 | 0.00021053 |
| SKMEL2:Sabutoclax..BI.97C1.:Ldose: 0.602059991 (uM) Interaction | 35.33029012 | 15.31686613 | 2.306626553 | 0.02108477 |
| UACC0257:Sabutoclax..BI.97C1.:Ldose: 0.602059991 (uM) Interaction | 41.47064216 | 15.05685414 | 2.754270034 | 0.005887088 |
| MeWo:Sirolimus..Rapamycin.:Ldose: 0.602059991 (uM) Interaction | 0.442637453 | 15.13733352 | 0.029241442 | 0.976672291 |
| SKMEL2:Sirolimus..Rapamycin.:Ldose: 0.602059991 (uM) Interaction | -7.678934159 | 15.31686613 | -0.501338465 | 0.616137888 |
| MeWo:Sorafenib:Ldose: 0.602059991 (uM) Interaction | 10.69694041 | 15.13733352 | 0.706659492 | 0.479785493 |
| UACC0257:Sorafenib:Ldose: 0.602059991 (uM) Interaction | 11.10209676 | 15.05685414 | 0.737345043 | 0.460920306 |
| MeWo:Streptozocin:Ldose: 0.602059991 (uM) Interaction | 6.923691885 | 15.13733352 | 0.457391777 | 0.64739393 |
| SKMEL2:Streptozocin:Ldose: 0.602059991 (uM) Interaction | -9.650089589 | 15.31686613 | -0.630030289 | 0.528681224 |
| UACC0257:Streptozocin:Ldose: 0.602059991 (uM) Interaction | 8.894268909 | 15.05685414 | 0.590712298 | 0.554719194 |
| MeWo:Sunitinib:Ldose: 0.602059991 (uM) Interaction | -3.2793534 | 15.13733352 | -0.216640097 | 0.828490817 |
| UACC0257:Sunitinib:Ldose: 0.602059991 (uM) Interaction | -1.066667359 | 15.05685414 | -0.070842644 | 0.943523627 |
| MeWo:Tamoxifen.Citrate:Ldose: 0.602059991 (uM) Interaction | 3.08307735 | 15.13733352 | 0.203673741 | 0.838610322 |
| SKMEL2:Tamoxifen.Citrate:Ldose: 0.602059991 (uM) Interaction | 8.95934611 | 15.31686613 | 0.584933369 | 0.558598439 |
| UACC0257:Tamoxifen.Citrate:Ldose: 0.602059991 (uM) Interaction | -0.119116073 | 15.05685414 | -0.007911086 | 0.993688003 |
| MeWo:Temozolomide:Ldose: 0.602059991 (uM) Interaction | -0.969054928 | 15.33308304 | -0.063200266 | 0.949607599 |
| SKMEL2:Temsirolimus..CCI.779..Torisel.:Ldose: 0.602059991 (uM) Interaction | 4.830037378 | 15.31686613 | 0.315341098 | 0.752505635 |
| MeWo:Teniposide:Ldose: 0.602059991 (uM) Interaction | -27.72886105 | 15.13733352 | -1.831819389 | 0.066991667 |
| SKMEL2:Teniposide:Ldose: 0.602059991 (uM) Interaction | -9.946034614 | 15.31686613 | -0.649351801 | 0.516117699 |
| UACC0257:Teniposide:Ldose: 0.602059991 (uM) Interaction | 14.91078105 | 15.05685414 | 0.990298565 | 0.322038954 |
| MeWo:Thioguanine:Ldose: 0.602059991 (uM) Interaction | 30.78771985 | 15.13733352 | 2.033893209 | 0.041974178 |
| SKMEL2:Thioguanine:Ldose: 0.602059991 (uM) Interaction | 68.21855005 | 15.31686613 | 4.453819044 | 8.48E-06 |
| UACC0257:Thioguanine:Ldose: 0.602059991 (uM) Interaction | 25.8357948 | 15.05685414 | 1.715882652 | 0.086197415 |
| MeWo:Thiotepa:Ldose: 0.602059991 (uM) Interaction | -3.582570883 | 15.13733352 | -0.2366712 | 0.812914054 |
| SKMEL2:Thiotepa:Ldose: 0.602059991 (uM) Interaction | -0.341953897 | 15.31686613 | -0.022325317 | 0.982188653 |
| UACC0257:Thiotepa:Ldose: 0.602059991 (uM) Interaction | 5.906827606 | 15.05685414 | 0.392301576 | 0.694839149 |
| MeWo:Topotecan.HCl:Ldose: 0.602059991 (uM) Interaction | 42.37272283 | 15.13733352 | 2.799219741 | 0.005127027 |
| SKMEL2:Topotecan.HCl:Ldose: 0.602059991 (uM) Interaction | 62.40531439 | 15.31686613 | 4.074287381 | 4.63E-05 |
| UACC0257:Topotecan.HCl:Ldose: 0.602059991 (uM) Interaction | 33.2508439 | 15.05685414 | 2.208352661 | 0.027229836 |
| MeWo:Trametinib..GSK1120212.:Ldose: 0.602059991 (uM) Interaction | 22.19956233 | 15.13733352 | 1.46654378 | 0.142514307 |
| UACC0257:Trametinib..GSK1120212.:Ldose: 0.602059991 (uM) Interaction | 8.738958097 | 15.05685414 | 0.58039734 | 0.561652564 |
| MeWo:Triethylenemelamine:Ldose: 0.602059991 (uM) Interaction | 1.533212652 | 15.13733352 | 0.101286838 | 0.919323675 |
| SKMEL2:Triethylenemelamine:Ldose: 0.602059991 (uM) Interaction | 1.761720515 | 15.31686613 | 0.11501834 | 0.908431621 |
| UACC0257:Triethylenemelamine:Ldose: 0.602059991 (uM) Interaction | 6.282316957 | 15.05685414 | 0.417239677 | 0.676507124 |
| MeWo:Uracil.mustard:Ldose: 0.602059991 (uM) Interaction | 10.10567007 | 15.13733352 | 0.667599089 | 0.504396449 |
| SKMEL2:Uracil.mustard:Ldose: 0.602059991 (uM) Interaction | 5.658446666 | 15.31686613 | 0.369425875 | 0.711813823 |
| MeWo:Valrubicin:Ldose: 0.602059991 (uM) Interaction | 55.94089909 | 15.13733352 | 3.695558336 | 0.000219929 |
| SKMEL2:Valrubicin:Ldose: 0.602059991 (uM) Interaction | 41.65783087 | 15.31686613 | 2.719735912 | 0.006538467 |
| UACC0257:Valrubicin:Ldose: 0.602059991 (uM) Interaction | 46.31661446 | 15.05685414 | 3.076114972 | 0.002099709 |
| MeWo:Vandetanib:Ldose: 0.602059991 (uM) Interaction | -11.81253342 | 15.07335079 | -0.78367004 | 0.433242071 |
| SKMEL2:Vandetanib:Ldose: 0.602059991 (uM) Interaction | -6.247280639 | 15.25363648 | -0.409560084 | 0.682132617 |
| SKMEL2:Vemurafenib:Ldose: 0.602059991 (uM) Interaction | 81.26496147 | 15.31686613 | 5.305586717 | 1.13E-07 |
| UACC0257:Vemurafenib:Ldose: 0.602059991 (uM) Interaction | 56.77365804 | 15.05685414 | 3.77061885 | 0.000163263 |
| SKMEL2:Vinblastine.Sulfate:Ldose: 0.602059991 (uM) Interaction | 10.50507701 | 15.31686613 | 0.685850285 | 0.492814654 |
| UACC0257:Vinblastine.Sulfate:Ldose: 0.602059991 (uM) Interaction | 33.68123072 | 15.05685414 | 2.236936774 | 0.025300317 |
| MeWo:Vincristine.Sulfate:Ldose: 0.602059991 (uM) Interaction | 30.48104216 | 15.13733352 | 2.013633518 | 0.044060016 |
| SKMEL2:Vincristine.Sulfate:Ldose: 0.602059991 (uM) Interaction | 58.8247814 | 15.31686613 | 3.84052331 | 0.000123112 |
| UACC0257:Vincristine.Sulfate:Ldose: 0.602059991 (uM) Interaction | 62.14256929 | 15.05685414 | 4.127194745 | 3.69E-05 |
| MeWo:Vinorelbine.Tartrate:Ldose: 0.602059991 (uM) Interaction | 30.58842639 | 15.13733352 | 2.020727517 | 0.043319909 |
| SKMEL2:Vinorelbine.Tartrate:Ldose: 0.602059991 (uM) Interaction | 31.77631979 | 15.31686613 | 2.074596691 | 0.038035374 |
| UACC0257:Vinorelbine.Tartrate:Ldose: 0.602059991 (uM) Interaction | 34.36859864 | 15.05685414 | 2.28258827 | 0.022464005 |
| MeWo:Vismodegib:Ldose: 0.602059991 (uM) Interaction | 9.435823635 | 15.13733352 | 0.623347806 | 0.533062365 |
| SKMEL2:Vismodegib:Ldose: 0.602059991 (uM) Interaction | -8.737152009 | 15.31686613 | -0.57042687 | 0.568393957 |
| UACC0257:Vismodegib:Ldose: 0.602059991 (uM) Interaction | 10.84006256 | 15.05685414 | 0.719942058 | 0.471568218 |
| MeWo:Vorinostat:Ldose: 0.602059991 (uM) Interaction | 7.367025568 | 15.13733352 | 0.486679213 | 0.626490462 |
| SKMEL2:Vorinostat:Ldose: 0.602059991 (uM) Interaction | -6.460211854 | 15.31686613 | -0.421771125 | 0.67319615 |
| UACC0257:Vorinostat:Ldose: 0.602059991 (uM) Interaction | 59.62011627 | 15.05685414 | 3.959666191 | 7.53E-05 |
| MeWo:Zoledronic.Acid:Ldose: 0.602059991 (uM) Interaction | -0.442373233 | 15.33308304 | -0.028850899 | 0.976983764 |
| MeWo:Abiraterone:Ldose: 1 (uM) Interaction | -4.356750209 | 15.13733352 | -0.287814905 | 0.773491103 |
| SKMEL2:Abiraterone:Ldose: 1 (uM) Interaction | -4.405053304 | 15.31686613 | -0.287594947 | 0.773659486 |
| MeWo:ABT.737:Ldose: 1 (uM) Interaction | -22.68341459 | 15.13733352 | -1.498507947 | 0.134015487 |
| SKMEL2:ABT.737:Ldose: 1 (uM) Interaction | -9.016365938 | 15.31686613 | -0.588656052 | 0.556097988 |
| UACC0257:ABT.737:Ldose: 1 (uM) Interaction | -7.19150594 | 15.05685414 | -0.477623405 | 0.632922952 |
| MeWo:Actinomycin.D:Ldose: 1 (uM) Interaction | -34.09894838 | 15.13733352 | -2.252639035 | 0.0242916 |
| SKMEL2:Actinomycin.D:Ldose: 1 (uM) Interaction | -34.10930762 | 15.31686613 | -2.22691165 | 0.025963126 |
| UACC0257:Actinomycin.D:Ldose: 1 (uM) Interaction | -16.50173707 | 15.05685414 | -1.095961807 | 0.273107327 |
| MeWo:Afatinib:Ldose: 1 (uM) Interaction | 0.664570089 | 15.13733352 | 0.043902718 | 0.964982342 |
| SKMEL2:Afatinib:Ldose: 1 (uM) Interaction | 29.31809441 | 15.31686613 | 1.914105285 | 0.055619492 |
| UACC0257:Afatinib:Ldose: 1 (uM) Interaction | -7.924008994 | 15.05685414 | -0.526272548 | 0.598704089 |
| MeWo:Alisertib..MLN8237.:Ldose: 1 (uM) Interaction | 25.03069837 | 15.13733352 | 1.653573816 | 0.098228214 |
| SKMEL2:Alisertib..MLN8237.:Ldose: 1 (uM) Interaction | 33.98454936 | 15.31686613 | 2.218766494 | 0.026512645 |
| MeWo:Allopurinol:Ldose: 1 (uM) Interaction | -4.483590226 | 15.13733352 | -0.296194189 | 0.767084545 |
| SKMEL2:Allopurinol:Ldose: 1 (uM) Interaction | -0.140596669 | 15.31686613 | -0.009179206 | 0.992676238 |
| UACC0257:Allopurinol:Ldose: 1 (uM) Interaction | 1.07064281 | 15.05685414 | 0.071106673 | 0.943313495 |
| MeWo:Amifostine:Ldose: 1 (uM) Interaction | -8.705175776 | 15.13733352 | -0.575079869 | 0.565243085 |
| MeWo:Aphrocallistin.analogue:Ldose: 1 (uM) Interaction | 21.28331688 | 15.13733352 | 1.406014926 | 0.159733628 |
| SKMEL2:Aphrocallistin.analogue:Ldose: 1 (uM) Interaction | 46.67881515 | 15.31686613 | 3.047543457 | 0.002309901 |
| UACC0257:Aphrocallistin.analogue:Ldose: 1 (uM) Interaction | 0.14448337 | 15.05685414 | 0.009595854 | 0.99234382 |
| MeWo:Arsenic.Trioxide:Ldose: 1 (uM) Interaction | 7.581827541 | 15.13733352 | 0.500869425 | 0.616467966 |
| SKMEL2:Arsenic.Trioxide:Ldose: 1 (uM) Interaction | 1.726250161 | 15.31686613 | 0.112702569 | 0.910267379 |
| MeWo:Axitinib:Ldose: 1 (uM) Interaction | 18.9311427 | 15.13733352 | 1.250625988 | 0.211084089 |
| SKMEL2:Axitinib:Ldose: 1 (uM) Interaction | 11.74912206 | 15.31686613 | 0.767070885 | 0.443047494 |
| UACC0257:Axitinib:Ldose: 1 (uM) Interaction | 8.121473511 | 15.05685414 | 0.539387141 | 0.589625138 |
| MeWo:Axitinib.1:Ldose: 1 (uM) Interaction | 17.02448266 | 15.13733352 | 1.124668531 | 0.260741629 |
| SKMEL2:Axitinib.1:Ldose: 1 (uM) Interaction | 15.36344328 | 15.31686613 | 1.003040906 | 0.31585201 |
| UACC0257:Axitinib.1:Ldose: 1 (uM) Interaction | 15.29097207 | 15.05685414 | 1.015548928 | 0.309855256 |
| MeWo:Azacitidine:Ldose: 1 (uM) Interaction | 20.64483926 | 15.13733352 | 1.363835925 | 0.172632979 |
| SKMEL2:Azacitidine:Ldose: 1 (uM) Interaction | 9.275886236 | 15.31686613 | 0.605599485 | 0.544786909 |
| UACC0257:Azacitidine:Ldose: 1 (uM) Interaction | 28.02814938 | 15.05685414 | 1.86148774 | 0.06268848 |
| SKMEL2:Baricitinib..LY3009104..INCB028050.:Ldose: 1 (uM) Interaction | 38.47087073 | 15.31686613 | 2.511667231 | 0.012023242 |
| UACC0257:Baricitinib..LY3009104..INCB028050.:Ldose: 1 (uM) Interaction | 12.09023204 | 15.05685414 | 0.802971984 | 0.421999487 |
| SKMEL2:Bendamustine.HCl:Ldose: 1 (uM) Interaction | 16.08388803 | 15.25363648 | 1.054429746 | 0.291697636 |
| MeWo:Bioymifi:Ldose: 1 (uM) Interaction | -7.133416338 | 15.13733352 | -0.471246559 | 0.637469284 |
| SKMEL2:Bioymifi:Ldose: 1 (uM) Interaction | -1.413967539 | 15.31686613 | -0.092314415 | 0.926449065 |
| UACC0257:Bioymifi:Ldose: 1 (uM) Interaction | -7.415828217 | 15.05685414 | -0.492521754 | 0.622355397 |
| MeWo:Bleomycin.Sulfate:Ldose: 1 (uM) Interaction | 19.9184916 | 15.13733352 | 1.315852067 | 0.188237229 |
| SKMEL2:Bleomycin.Sulfate:Ldose: 1 (uM) Interaction | 41.88772084 | 15.31686613 | 2.734744855 | 0.006247796 |
| UACC0257:Bleomycin.Sulfate:Ldose: 1 (uM) Interaction | 44.41870353 | 15.05685414 | 2.95006534 | 0.003180369 |
| MeWo:Bortezomib:Ldose: 1 (uM) Interaction | 3.333362662 | 15.13733352 | 0.220208048 | 0.825711145 |
| SKMEL2:Bortezomib:Ldose: 1 (uM) Interaction | 13.48237179 | 15.31686613 | 0.880230438 | 0.378743975 |
| UACC0257:Bortezomib:Ldose: 1 (uM) Interaction | 22.1728202 | 15.05685414 | 1.472606429 | 0.140871306 |
| MeWo:Bosutinib..SKI.606.:Ldose: 1 (uM) Interaction | -18.05281573 | 15.13733352 | -1.192602099 | 0.233037935 |
| SKMEL2:Bosutinib..SKI.606.:Ldose: 1 (uM) Interaction | 10.08790776 | 15.31686613 | 0.658614346 | 0.510150249 |
| UACC0257:Bosutinib..SKI.606.:Ldose: 1 (uM) Interaction | -39.82251097 | 15.05685414 | -2.644809507 | 0.008179419 |
| SKMEL2:Busulfan:Ldose: 1 (uM) Interaction | 1.572436212 | 15.31686613 | 0.10266044 | 0.918233395 |
| MeWo:Cabazitaxel:Ldose: 1 (uM) Interaction | -3.561727774 | 15.13733352 | -0.235294266 | 0.813982507 |
| SKMEL2:Cabazitaxel:Ldose: 1 (uM) Interaction | -5.572672291 | 15.31686613 | -0.36382588 | 0.715991486 |
| UACC0257:Cabazitaxel:Ldose: 1 (uM) Interaction | -13.79923218 | 15.05685414 | -0.916475118 | 0.359427664 |
| MeWo:Cabozantinib..XL.184.:Ldose: 1 (uM) Interaction | 18.51669534 | 15.13733352 | 1.223246836 | 0.221249409 |
| SKMEL2:Cabozantinib..XL.184.:Ldose: 1 (uM) Interaction | 48.97621978 | 15.31686613 | 3.197535277 | 0.001388009 |
| UACC0257:Cabozantinib..XL.184.:Ldose: 1 (uM) Interaction | 6.490819265 | 15.05685414 | 0.431087344 | 0.66640903 |
| MeWo:Capecitabine:Ldose: 1 (uM) Interaction | 5.64221413 | 15.33308304 | 0.367976493 | 0.712894255 |
| MeWo:Carfilzomib:Ldose: 1 (uM) Interaction | -70.99236493 | 15.13733352 | -4.68988576 | 2.75E-06 |
| SKMEL2:Carfilzomib:Ldose: 1 (uM) Interaction | -5.254808153 | 15.31686613 | -0.343073322 | 0.731546535 |
| UACC0257:Carfilzomib:Ldose: 1 (uM) Interaction | -69.81919942 | 15.05685414 | -4.637037643 | 3.55E-06 |
| MeWo:Carmustine:Ldose: 1 (uM) Interaction | -4.876992582 | 15.33308304 | -0.318069926 | 0.750434875 |
| MeWo:Celecoxib:Ldose: 1 (uM) Interaction | 1.4014453 | 15.33308304 | 0.091400099 | 0.927175505 |
| UACC0257:Chlorambucil:Ldose: 1 (uM) Interaction | -7.683804899 | 15.05685414 | -0.510319409 | 0.60983276 |
| MeWo:Cisplatin:Ldose: 1 (uM) Interaction | 1.124949991 | 15.13733352 | 0.074316258 | 0.940759407 |
| SKMEL2:Cisplatin:Ldose: 1 (uM) Interaction | -0.195640753 | 15.31686613 | -0.012772897 | 0.989809094 |
| MeWo:Cladribine:Ldose: 1 (uM) Interaction | 43.95866377 | 15.13733352 | 2.903989907 | 0.003688016 |
| SKMEL2:Cladribine:Ldose: 1 (uM) Interaction | 33.5395911 | 15.31686613 | 2.189716279 | 0.028555145 |
| UACC0257:Cladribine:Ldose: 1 (uM) Interaction | 11.48687602 | 15.05685414 | 0.762900133 | 0.445531028 |
| MeWo:Clofarabine:Ldose: 1 (uM) Interaction | 34.50988523 | 15.13733352 | 2.279786276 | 0.022629768 |
| SKMEL2:Clofarabine:Ldose: 1 (uM) Interaction | 30.71588732 | 15.31686613 | 2.005363699 | 0.044936242 |
| UACC0257:Clofarabine:Ldose: 1 (uM) Interaction | -6.37900891 | 15.05685414 | -0.423661467 | 0.671816808 |
| MeWo:Crizotinib:Ldose: 1 (uM) Interaction | 5.209510917 | 15.13733352 | 0.34414984 | 0.730736848 |
| SKMEL2:Crizotinib:Ldose: 1 (uM) Interaction | 18.1953872 | 15.31686613 | 1.187931463 | 0.234873067 |
| UACC0257:Crizotinib:Ldose: 1 (uM) Interaction | 27.63404774 | 15.05685414 | 1.835313505 | 0.066472563 |
| MeWo:Cytarabine.HCl...Ara.C:Ldose: 1 (uM) Interaction | 45.37918723 | 15.13733352 | 2.99783229 | 0.002722068 |
| SKMEL2:Cytarabine.HCl...Ara.C:Ldose: 1 (uM) Interaction | 57.62206593 | 15.31686613 | 3.762001016 | 0.000168991 |
| UACC0257:Cytarabine.HCl...Ara.C:Ldose: 1 (uM) Interaction | 13.81467959 | 15.05685414 | 0.917501057 | 0.358890063 |
| MeWo:Dacarbazine:Ldose: 1 (uM) Interaction | 38.25806915 | 15.13733352 | 2.527398177 | 0.011497966 |
| SKMEL2:Dacarbazine:Ldose: 1 (uM) Interaction | 39.84051943 | 15.31686613 | 2.601088179 | 0.009298993 |
| MeWo:Dacomitinib..PF299804.:Ldose: 1 (uM) Interaction | -2.201516828 | 15.13733352 | -0.145436237 | 0.88436777 |
| SKMEL2:Dacomitinib..PF299804.:Ldose: 1 (uM) Interaction | 0.085976841 | 15.31686613 | 0.005613214 | 0.995521377 |
| UACC0257:Dacomitinib..PF299804.:Ldose: 1 (uM) Interaction | 3.263981555 | 15.05685414 | 0.216777125 | 0.828384023 |
| MeWo:Dasatinib:Ldose: 1 (uM) Interaction | 11.12003181 | 15.13733352 | 0.734609685 | 0.462584964 |
| SKMEL2:Dasatinib:Ldose: 1 (uM) Interaction | 19.72598918 | 15.31686613 | 1.287860651 | 0.197807841 |
| UACC0257:Dasatinib:Ldose: 1 (uM) Interaction | 22.98938242 | 15.05685414 | 1.526838356 | 0.126815396 |
| MeWo:Daunorubicin.HCl:Ldose: 1 (uM) Interaction | -25.12079637 | 15.13733352 | -1.659525855 | 0.097023939 |
| SKMEL2:Daunorubicin.HCl:Ldose: 1 (uM) Interaction | 20.20084243 | 15.31686613 | 1.318862635 | 0.187228585 |
| UACC0257:Daunorubicin.HCl:Ldose: 1 (uM) Interaction | -14.76595543 | 15.05685414 | -0.980679981 | 0.32676122 |
| MeWo:Decitabine:Ldose: 1 (uM) Interaction | -12.07599237 | 15.13733352 | -0.797762192 | 0.425017002 |
| SKMEL2:Decitabine:Ldose: 1 (uM) Interaction | 3.019039161 | 15.31686613 | 0.197105539 | 0.843746745 |
| MeWo:Docetaxel:Ldose: 1 (uM) Interaction | -0.141067904 | 15.13733352 | -0.009319204 | 0.992564542 |
| MeWo:Doxorubicin.HCl:Ldose: 1 (uM) Interaction | 7.795045458 | 15.13733352 | 0.514954992 | 0.606589577 |
| SKMEL2:Doxorubicin.HCl:Ldose: 1 (uM) Interaction | 39.49876561 | 15.31686613 | 2.578775925 | 0.009921457 |
| UACC0257:Doxorubicin.HCl:Ldose: 1 (uM) Interaction | 3.303824511 | 15.05685414 | 0.219423293 | 0.826322336 |
| MeWo:Erlotinib.HCl:Ldose: 1 (uM) Interaction | -11.4939467 | 15.13733352 | -0.759311188 | 0.447674454 |
| SKMEL2:Erlotinib.HCl:Ldose: 1 (uM) Interaction | 11.76887009 | 15.31686613 | 0.768360185 | 0.442281366 |
| UACC0257:Erlotinib.HCl:Ldose: 1 (uM) Interaction | -3.90407707 | 15.05685414 | -0.259289028 | 0.795414638 |
| MeWo:Etoposide:Ldose: 1 (uM) Interaction | 29.35735931 | 15.13733352 | 1.939400969 | 0.052465131 |
| SKMEL2:Etoposide:Ldose: 1 (uM) Interaction | 22.88891992 | 15.31686613 | 1.494360513 | 0.135095568 |
| UACC0257:Etoposide:Ldose: 1 (uM) Interaction | 21.42482833 | 15.05685414 | 1.422928597 | 0.154770847 |
| MeWo:Everolimus:Ldose: 1 (uM) Interaction | -5.607715648 | 15.33308304 | -0.365726556 | 0.714572598 |
| MeWo:Exemestane:Ldose: 1 (uM) Interaction | 18.2309971 | 15.07335079 | 1.20948536 | 0.226489257 |
| SKMEL2:Exemestane:Ldose: 1 (uM) Interaction | 13.0628991 | 15.25363648 | 0.856379337 | 0.391797194 |
| MeWo:Floxuridine:Ldose: 1 (uM) Interaction | -9.991261846 | 15.13733352 | -0.660041072 | 0.509234291 |
| SKMEL2:Floxuridine:Ldose: 1 (uM) Interaction | 63.31697981 | 15.31686613 | 4.133807744 | 3.58E-05 |
| UACC0257:Floxuridine:Ldose: 1 (uM) Interaction | 1.538895892 | 15.05685414 | 0.102205672 | 0.918594345 |
| MeWo:Fludarabine.Phosphate:Ldose: 1 (uM) Interaction | 30.22259591 | 15.13733352 | 1.996560085 | 0.04588513 |
| SKMEL2:Fludarabine.Phosphate:Ldose: 1 (uM) Interaction | 50.62133442 | 15.31686613 | 3.30494071 | 0.000951463 |
| MeWo:Fluorouracil...5.FU.:Ldose: 1 (uM) Interaction | -6.021015299 | 15.13733352 | -0.397759307 | 0.690811424 |
| SKMEL2:Fluorouracil...5.FU.:Ldose: 1 (uM) Interaction | 20.16184616 | 15.31686613 | 1.316316666 | 0.188081311 |
| UACC0257:Fluorouracil...5.FU.:Ldose: 1 (uM) Interaction | -8.031028299 | 15.05685414 | -0.533380228 | 0.593775718 |
| MeWo:Flutamide..Eulexin.:Ldose: 1 (uM) Interaction | 1.265619272 | 15.13733352 | 0.083609129 | 0.933367959 |
| SKMEL2:Flutamide..Eulexin.:Ldose: 1 (uM) Interaction | 8.255844337 | 15.31686613 | 0.539003493 | 0.589889826 |
| UACC0257:Flutamide..Eulexin.:Ldose: 1 (uM) Interaction | -8.333657645 | 15.05685414 | -0.553479337 | 0.579940776 |
| MeWo:Foretinib..GSK1363089.:Ldose: 1 (uM) Interaction | 58.46732037 | 15.13733352 | 3.862458357 | 0.000112569 |
| SKMEL2:Foretinib..GSK1363089.:Ldose: 1 (uM) Interaction | 99.74098624 | 15.31686613 | 6.511840308 | 7.58E-11 |
| UACC0257:Foretinib..GSK1363089.:Ldose: 1 (uM) Interaction | 13.53884857 | 15.05685414 | 0.899181757 | 0.368565566 |
| SKMEL2:Fulvestrant:Ldose: 1 (uM) Interaction | -7.642402313 | 15.31686613 | -0.498953392 | 0.617817141 |
| UACC0257:Fulvestrant:Ldose: 1 (uM) Interaction | 21.15239677 | 15.05685414 | 1.404835072 | 0.160084259 |
| MeWo:Gefitinib:Ldose: 1 (uM) Interaction | 5.22914587 | 15.33308304 | 0.341036819 | 0.733079077 |
| MeWo:Gemcitabine.HCl:Ldose: 1 (uM) Interaction | 22.9554819 | 15.13733352 | 1.516481213 | 0.129411894 |
| SKMEL2:Gemcitabine.HCl:Ldose: 1 (uM) Interaction | 64.19816017 | 15.31686613 | 4.191337812 | 2.78E-05 |
| UACC0257:Gemcitabine.HCl:Ldose: 1 (uM) Interaction | -18.78296276 | 15.05685414 | -1.247469264 | 0.212238586 |
| MeWo:Ibrutinib..PCI.32765.:Ldose: 1 (uM) Interaction | 16.77410292 | 15.07335079 | 1.112831722 | 0.265792706 |
| SKMEL2:Ibrutinib..PCI.32765.:Ldose: 1 (uM) Interaction | 16.84375818 | 15.25363648 | 1.104245417 | 0.26949861 |
| MeWo:Imiquimod:Ldose: 1 (uM) Interaction | -22.15698501 | 15.07335079 | -1.469944229 | 0.14159097 |
| SKMEL2:Imiquimod:Ldose: 1 (uM) Interaction | -20.92412708 | 15.25363648 | -1.371746803 | 0.170156076 |
| MeWo:INK.128..MLN0128.:Ldose: 1 (uM) Interaction | 21.68607438 | 15.13733352 | 1.432621825 | 0.151979961 |
| SKMEL2:INK.128..MLN0128.:Ldose: 1 (uM) Interaction | -0.941511774 | 15.31686613 | -0.061468956 | 0.950986288 |
| UACC0257:INK.128..MLN0128.:Ldose: 1 (uM) Interaction | 9.02795294 | 15.05685414 | 0.599590914 | 0.548784997 |
| MeWo:Irinotecan.HCl:Ldose: 1 (uM) Interaction | 54.98856111 | 15.13733352 | 3.632645144 | 0.00028116 |
| SKMEL2:Irinotecan.HCl:Ldose: 1 (uM) Interaction | 48.05873655 | 15.31686613 | 3.137635085 | 0.001705385 |
| UACC0257:Irinotecan.HCl:Ldose: 1 (uM) Interaction | 24.35277184 | 15.05685414 | 1.617387776 | 0.105808749 |
| MeWo:Ixabepilone:Ldose: 1 (uM) Interaction | 21.25802522 | 15.13733352 | 1.404344113 | 0.160230334 |
| SKMEL2:Ixabepilone:Ldose: 1 (uM) Interaction | 21.27702496 | 15.31686613 | 1.38912391 | 0.164808948 |
| UACC0257:Ixabepilone:Ldose: 1 (uM) Interaction | 27.84099133 | 15.05685414 | 1.84905765 | 0.064462717 |
| MeWo:Lapatinib:Ldose: 1 (uM) Interaction | -6.704962061 | 15.07335079 | -0.444822266 | 0.65645252 |
| SKMEL2:Lapatinib:Ldose: 1 (uM) Interaction | -13.15705706 | 15.25363648 | -0.862552158 | 0.388393022 |
| MeWo:LDK378:Ldose: 1 (uM) Interaction | 13.3912566 | 15.13733352 | 0.884650958 | 0.376354454 |
| SKMEL2:LDK378:Ldose: 1 (uM) Interaction | 55.45783729 | 15.31686613 | 3.620703924 | 0.000294454 |
| UACC0257:LDK378:Ldose: 1 (uM) Interaction | 31.31223715 | 15.05685414 | 2.079600218 | 0.037573615 |
| MeWo:Lenalidomide:Ldose: 1 (uM) Interaction | 3.454174501 | 15.13733352 | 0.228189099 | 0.819501328 |
| SKMEL2:Lenalidomide:Ldose: 1 (uM) Interaction | -3.909693854 | 15.31686613 | -0.255254164 | 0.798529154 |
| SKMEL2:Letrozole:Ldose: 1 (uM) Interaction | 4.734743922 | 15.31686613 | 0.309119626 | 0.757233419 |
| MeWo:Linsitinib:Ldose: 1 (uM) Interaction | 4.210938599 | 15.13733352 | 0.278182323 | 0.780874984 |
| SKMEL2:Linsitinib:Ldose: 1 (uM) Interaction | -2.796225009 | 15.31686613 | -0.182558559 | 0.855146063 |
| UACC0257:Linsitinib:Ldose: 1 (uM) Interaction | -12.25935799 | 15.05685414 | -0.814204473 | 0.415536519 |
| SKMEL2:Lomustine..CCNU.:Ldose: 1 (uM) Interaction | -8.322181438 | 15.31686613 | -0.543334476 | 0.586904963 |
| MeWo:LY2157299:Ldose: 1 (uM) Interaction | -9.045761784 | 15.13733352 | -0.597579605 | 0.550126545 |
| SKMEL2:LY2157299:Ldose: 1 (uM) Interaction | 5.452385491 | 15.31686613 | 0.355972654 | 0.721864412 |
| MeWo:Mechlorethamine.HCl:Ldose: 1 (uM) Interaction | 1.454047952 | 15.13733352 | 0.096057073 | 0.923476108 |
| SKMEL2:Mechlorethamine.HCl:Ldose: 1 (uM) Interaction | 14.95975294 | 15.31686613 | 0.97668497 | 0.328735735 |
| UACC0257:Mechlorethamine.HCl:Ldose: 1 (uM) Interaction | 21.92493704 | 15.05685414 | 1.456143285 | 0.145367095 |
| MeWo:Megestrol.acetate:Ldose: 1 (uM) Interaction | 4.356791356 | 15.13733352 | 0.287817623 | 0.773489022 |
| SKMEL2:Megestrol.acetate:Ldose: 1 (uM) Interaction | -0.620481174 | 15.31686613 | -0.040509669 | 0.967687161 |
| MeWo:MEK.162..ARRY.438162.:Ldose: 1 (uM) Interaction | 1.160119236 | 15.13733352 | 0.076639603 | 0.93891094 |
| SKMEL2:MEK.162..ARRY.438162.:Ldose: 1 (uM) Interaction | 14.14863137 | 15.31686613 | 0.923728865 | 0.355637496 |
| UACC0257:MEK.162..ARRY.438162.:Ldose: 1 (uM) Interaction | 22.69583428 | 15.05685414 | 1.507342375 | 0.131737089 |
| MeWo:Melphalan:Ldose: 1 (uM) Interaction | 17.6569205 | 15.13733352 | 1.166448534 | 0.243445622 |
| SKMEL2:Melphalan:Ldose: 1 (uM) Interaction | -8.792617248 | 15.31686613 | -0.574048057 | 0.565941072 |
| UACC0257:Melphalan:Ldose: 1 (uM) Interaction | 8.198742283 | 15.05685414 | 0.544518942 | 0.586089863 |
| MeWo:Mercaptopurine:Ldose: 1 (uM) Interaction | 25.31127553 | 15.13733352 | 1.672109258 | 0.094516811 |
| SKMEL2:Mercaptopurine:Ldose: 1 (uM) Interaction | 70.76877864 | 15.31686613 | 4.62031711 | 3.85E-06 |
| UACC0257:Mercaptopurine:Ldose: 1 (uM) Interaction | 27.06731395 | 15.05685414 | 1.797673916 | 0.072242222 |
| MeWo:Mitomycin.C:Ldose: 1 (uM) Interaction | 30.9079764 | 15.13733352 | 2.041837577 | 0.041179389 |
| SKMEL2:Mitomycin.C:Ldose: 1 (uM) Interaction | 42.46876762 | 15.31686613 | 2.772679951 | 0.005564292 |
| UACC0257:Mitomycin.C:Ldose: 1 (uM) Interaction | -16.37356956 | 15.05685414 | -1.08744957 | 0.276849929 |
| MeWo:Mitotane..o.p..DDD..Lysodren.:Ldose: 1 (uM) Interaction | 8.250010996 | 15.13733352 | 0.545010849 | 0.585751507 |
| SKMEL2:Mitotane..o.p..DDD..Lysodren.:Ldose: 1 (uM) Interaction | -9.703938203 | 15.31686613 | -0.63354593 | 0.526383696 |
| MeWo:Mitoxantrone:Ldose: 1 (uM) Interaction | -3.743855858 | 15.13733352 | -0.247325981 | 0.804658231 |
| SKMEL2:Mitoxantrone:Ldose: 1 (uM) Interaction | 38.58824961 | 15.31686613 | 2.519330605 | 0.011764752 |
| UACC0257:Mitoxantrone:Ldose: 1 (uM) Interaction | 4.855786016 | 15.05685414 | 0.322496716 | 0.747079449 |
| UACC0257:MLN.2480:Ldose: 1 (uM) Interaction | -12.46819249 | 15.05685414 | -0.828074203 | 0.407637342 |
| MeWo:MLN4924:Ldose: 1 (uM) Interaction | 23.04547214 | 15.13733352 | 1.522426133 | 0.127916523 |
| SKMEL2:MLN4924:Ldose: 1 (uM) Interaction | 34.65730865 | 15.31686613 | 2.262689271 | 0.023664429 |
| UACC0257:MLN4924:Ldose: 1 (uM) Interaction | 36.87067133 | 15.05685414 | 2.448763267 | 0.014342408 |
| MeWo:MLN9708..MLN2238.:Ldose: 1 (uM) Interaction | -20.90816958 | 15.13733352 | -1.381232008 | 0.167221478 |
| SKMEL2:MLN9708..MLN2238.:Ldose: 1 (uM) Interaction | 16.16296339 | 15.31686613 | 1.055239581 | 0.2913272 |
| UACC0257:MLN9708..MLN2238.:Ldose: 1 (uM) Interaction | -0.845668773 | 15.05685414 | -0.056165037 | 0.955210837 |
| MeWo:Navitoclax..ABT.263..5uM:Ldose: 1 (uM) Interaction | -11.06312984 | 15.13733352 | -0.730850637 | 0.46487807 |
| SKMEL2:Navitoclax..ABT.263..5uM:Ldose: 1 (uM) Interaction | -1.834305308 | 15.31686613 | -0.11975722 | 0.90467655 |
| UACC0257:Navitoclax..ABT.263..5uM:Ldose: 1 (uM) Interaction | 2.713482625 | 15.05685414 | 0.180215774 | 0.856984813 |
| MeWo:Nelarabine:Ldose: 1 (uM) Interaction | -7.655526656 | 15.13733352 | -0.505738124 | 0.613045501 |
| SKMEL2:Nelarabine:Ldose: 1 (uM) Interaction | -10.71913074 | 15.31686613 | -0.69982532 | 0.484043701 |
| UACC0257:Nelarabine:Ldose: 1 (uM) Interaction | -7.403775542 | 15.05685414 | -0.491721277 | 0.622921236 |
| MeWo:OSI.027:Ldose: 1 (uM) Interaction | -16.7972426 | 15.13733352 | -1.109656636 | 0.267158984 |
| SKMEL2:OSI.027:Ldose: 1 (uM) Interaction | 17.74827962 | 15.31686613 | 1.158740924 | 0.246574215 |
| UACC0257:OSI.027:Ldose: 1 (uM) Interaction | -18.60432035 | 15.05685414 | -1.23560474 | 0.21661854 |
| MeWo:Oxaliplatin:Ldose: 1 (uM) Interaction | -9.513797527 | 15.07335079 | -0.63116673 | 0.527937983 |
| SKMEL2:Oxaliplatin:Ldose: 1 (uM) Interaction | 10.89198533 | 15.25363648 | 0.714058274 | 0.475198621 |
| MeWo:Paclitaxel:Ldose: 1 (uM) Interaction | 36.60541724 | 15.13733352 | 2.418220962 | 0.015604563 |
| SKMEL2:Paclitaxel:Ldose: 1 (uM) Interaction | 32.84013126 | 15.31686613 | 2.14405029 | 0.032039638 |
| MeWo:Palbociclib..PD.0332991..Isethionate:Ldose: 1 (uM) Interaction | -16.43510615 | 15.13733352 | -1.085733238 | 0.277608769 |
| SKMEL2:Palbociclib..PD.0332991..Isethionate:Ldose: 1 (uM) Interaction | 28.74865422 | 15.31686613 | 1.876927955 | 0.060541017 |
| UACC0257:Palbociclib..PD.0332991..Isethionate:Ldose: 1 (uM) Interaction | 1.967105205 | 15.05685414 | 0.130645166 | 0.89605719 |
| MeWo:Pazopanib.HCl:Ldose: 1 (uM) Interaction | -11.51980911 | 15.13733352 | -0.761019706 | 0.446653345 |
| SKMEL2:Pazopanib.HCl:Ldose: 1 (uM) Interaction | -52.06942427 | 15.31686613 | -3.399482886 | 0.000676316 |
| UACC0257:Pazopanib.HCl:Ldose: 1 (uM) Interaction | -16.68814448 | 15.05685414 | -1.108342043 | 0.26772608 |
| MeWo:PD325901:Ldose: 1 (uM) Interaction | -41.01709224 | 15.13733352 | -2.709664299 | 0.006740281 |
| SKMEL2:PD325901:Ldose: 1 (uM) Interaction | -41.0436532 | 15.31686613 | -2.679637783 | 0.007375598 |
| UACC0257:PD325901:Ldose: 1 (uM) Interaction | 4.158546163 | 15.05685414 | 0.276189576 | 0.782405023 |
| SKMEL2:Pemetrexed:Ldose: 1 (uM) Interaction | 11.80778772 | 15.31686613 | 0.77090102 | 0.440773771 |
| MeWo:Plicamycin:Ldose: 1 (uM) Interaction | 46.86258859 | 15.13733352 | 3.095828503 | 0.001965071 |
| SKMEL2:Plicamycin:Ldose: 1 (uM) Interaction | 39.92804332 | 15.31686613 | 2.606802396 | 0.009145291 |
| UACC0257:Plicamycin:Ldose: 1 (uM) Interaction | 65.31673063 | 15.05685414 | 4.338006467 | 1.44E-05 |
| MeWo:Pralatrexate:Ldose: 1 (uM) Interaction | 8.043415471 | 15.07335079 | 0.533618277 | 0.59361098 |
| SKMEL2:Pralatrexate:Ldose: 1 (uM) Interaction | 0.895916008 | 15.25363648 | 0.058734585 | 0.953164038 |
| MeWo:Quinacrine.HCl:Ldose: 1 (uM) Interaction | -5.674077953 | 15.13733352 | -0.37483999 | 0.707783036 |
| SKMEL2:Quinacrine.HCl:Ldose: 1 (uM) Interaction | -39.55459546 | 15.31686613 | -2.582420916 | 0.009817299 |
| UACC0257:Quinacrine.HCl:Ldose: 1 (uM) Interaction | -26.76007479 | 15.05685414 | -1.777268648 | 0.075537679 |
| MeWo:Quizartinib:Ldose: 1 (uM) Interaction | -3.349695159 | 15.13733352 | -0.221287002 | 0.824870996 |
| SKMEL2:Quizartinib:Ldose: 1 (uM) Interaction | 2.856372677 | 15.31686613 | 0.18648545 | 0.852065784 |
| UACC0257:Quizartinib:Ldose: 1 (uM) Interaction | -1.459399749 | 15.05685414 | -0.096925941 | 0.922786079 |
| MeWo:Raloxifene:Ldose: 1 (uM) Interaction | 17.97826647 | 15.07335079 | 1.192718641 | 0.232992275 |
| SKMEL2:Raloxifene:Ldose: 1 (uM) Interaction | 18.98057108 | 15.25363648 | 1.244330892 | 0.213390887 |
| MeWo:Romidepsin:Ldose: 1 (uM) Interaction | 21.89993736 | 15.13733352 | 1.446750005 | 0.147981012 |
| SKMEL2:Romidepsin:Ldose: 1 (uM) Interaction | 18.58839666 | 15.31686613 | 1.213590071 | 0.224917156 |
| UACC0257:Romidepsin:Ldose: 1 (uM) Interaction | 8.963932191 | 15.05685414 | 0.59533898 | 0.551622947 |
| MeWo:Sabutoclax..BI.97C1.:Ldose: 1 (uM) Interaction | 20.40825597 | 15.13733352 | 1.348206799 | 0.177605574 |
| SKMEL2:Sabutoclax..BI.97C1.:Ldose: 1 (uM) Interaction | 42.51146198 | 15.31686613 | 2.775467359 | 0.005516835 |
| UACC0257:Sabutoclax..BI.97C1.:Ldose: 1 (uM) Interaction | 24.82065444 | 15.05685414 | 1.648462169 | 0.099271957 |
| MeWo:Sirolimus..Rapamycin.:Ldose: 1 (uM) Interaction | -5.778407624 | 15.13733352 | -0.3817322 | 0.702663651 |
| SKMEL2:Sirolimus..Rapamycin.:Ldose: 1 (uM) Interaction | -14.61686809 | 15.31686613 | -0.954298873 | 0.339942724 |
| MeWo:Sorafenib:Ldose: 1 (uM) Interaction | 29.91213129 | 15.13733352 | 1.976050223 | 0.048161397 |
| UACC0257:Sorafenib:Ldose: 1 (uM) Interaction | 18.05466396 | 15.05685414 | 1.199099347 | 0.230502061 |
| MeWo:Streptozocin:Ldose: 1 (uM) Interaction | 4.883139544 | 15.13733352 | 0.322589149 | 0.747009437 |
| SKMEL2:Streptozocin:Ldose: 1 (uM) Interaction | -13.26921408 | 15.31686613 | -0.866313903 | 0.386327366 |
| UACC0257:Streptozocin:Ldose: 1 (uM) Interaction | -0.552875327 | 15.05685414 | -0.036719179 | 0.970709245 |
| MeWo:Sunitinib:Ldose: 1 (uM) Interaction | 8.885766226 | 15.13733352 | 0.587010005 | 0.557202931 |
| UACC0257:Sunitinib:Ldose: 1 (uM) Interaction | -12.64315103 | 15.05685414 | -0.839694063 | 0.401088947 |
| MeWo:Tamoxifen.Citrate:Ldose: 1 (uM) Interaction | -8.830100936 | 15.13733352 | -0.583332654 | 0.559675285 |
| SKMEL2:Tamoxifen.Citrate:Ldose: 1 (uM) Interaction | 2.380140729 | 15.31686613 | 0.155393454 | 0.876512551 |
| UACC0257:Tamoxifen.Citrate:Ldose: 1 (uM) Interaction | -11.705381 | 15.05685414 | -0.777412127 | 0.436923908 |
| MeWo:Temozolomide:Ldose: 1 (uM) Interaction | -5.271457935 | 15.33308304 | -0.343796347 | 0.73100269 |
| SKMEL2:Temsirolimus..CCI.779..Torisel.:Ldose: 1 (uM) Interaction | 3.309301812 | 15.31686613 | 0.216056064 | 0.828946023 |
| MeWo:Teniposide:Ldose: 1 (uM) Interaction | -36.29168173 | 15.13733352 | -2.397495019 | 0.01651578 |
| SKMEL2:Teniposide:Ldose: 1 (uM) Interaction | -5.214048288 | 15.31686613 | -0.340412213 | 0.73354933 |
| UACC0257:Teniposide:Ldose: 1 (uM) Interaction | 14.03382693 | 15.05685414 | 0.932055714 | 0.351317826 |
| MeWo:Thioguanine:Ldose: 1 (uM) Interaction | 27.04772982 | 15.13733352 | 1.786822612 | 0.073979745 |
| SKMEL2:Thioguanine:Ldose: 1 (uM) Interaction | 44.98836008 | 15.31686613 | 2.937177861 | 0.003315528 |
| UACC0257:Thioguanine:Ldose: 1 (uM) Interaction | 17.13636701 | 15.05685414 | 1.138110714 | 0.255086484 |
| MeWo:Thiotepa:Ldose: 1 (uM) Interaction | -2.631654429 | 15.13733352 | -0.173851916 | 0.861983421 |
| SKMEL2:Thiotepa:Ldose: 1 (uM) Interaction | 13.10148286 | 15.31686613 | 0.85536315 | 0.392359329 |
| UACC0257:Thiotepa:Ldose: 1 (uM) Interaction | 5.932413048 | 15.05685414 | 0.394000831 | 0.693584193 |
| MeWo:Topotecan.HCl:Ldose: 1 (uM) Interaction | 19.63307983 | 15.13733352 | 1.296997242 | 0.194645557 |
| SKMEL2:Topotecan.HCl:Ldose: 1 (uM) Interaction | 48.87672637 | 15.31686613 | 3.1910396 | 0.001419583 |
| UACC0257:Topotecan.HCl:Ldose: 1 (uM) Interaction | 1.85681524 | 15.05685414 | 0.123320265 | 0.901854606 |
| MeWo:Trametinib..GSK1120212.:Ldose: 1 (uM) Interaction | -16.98732766 | 15.13733352 | -1.122214004 | 0.261783545 |
| UACC0257:Trametinib..GSK1120212.:Ldose: 1 (uM) Interaction | 30.91081628 | 15.05685414 | 2.052939877 | 0.040090052 |
| MeWo:Triethylenemelamine:Ldose: 1 (uM) Interaction | -2.605686393 | 15.13733352 | -0.172136419 | 0.863331844 |
| SKMEL2:Triethylenemelamine:Ldose: 1 (uM) Interaction | -3.213598581 | 15.31686613 | -0.209807839 | 0.833819572 |
| UACC0257:Triethylenemelamine:Ldose: 1 (uM) Interaction | 9.644827935 | 15.05685414 | 0.640560627 | 0.52181477 |
| MeWo:Uracil.mustard:Ldose: 1 (uM) Interaction | 21.30675001 | 15.13733352 | 1.407562962 | 0.159274462 |
| SKMEL2:Uracil.mustard:Ldose: 1 (uM) Interaction | 10.10359124 | 15.31686613 | 0.659638281 | 0.509492796 |
| MeWo:Valrubicin:Ldose: 1 (uM) Interaction | 2.209505949 | 15.13733352 | 0.145964013 | 0.883951117 |
| SKMEL2:Valrubicin:Ldose: 1 (uM) Interaction | -4.020294801 | 15.31686613 | -0.262475024 | 0.792957664 |
| UACC0257:Valrubicin:Ldose: 1 (uM) Interaction | 0.665116366 | 15.05685414 | 0.044173661 | 0.964766373 |
| MeWo:Vandetanib:Ldose: 1 (uM) Interaction | 17.06306386 | 15.07335079 | 1.132002041 | 0.257645751 |
| SKMEL2:Vandetanib:Ldose: 1 (uM) Interaction | 22.0809788 | 15.25363648 | 1.447587848 | 0.147746412 |
| SKMEL2:Vemurafenib:Ldose: 1 (uM) Interaction | 42.17350194 | 15.31686613 | 2.753402791 | 0.005902703 |
| UACC0257:Vemurafenib:Ldose: 1 (uM) Interaction | 24.36415436 | 15.05685414 | 1.618143746 | 0.105645768 |
| SKMEL2:Vinblastine.Sulfate:Ldose: 1 (uM) Interaction | 49.29509113 | 15.31686613 | 3.218353592 | 0.001291131 |
| UACC0257:Vinblastine.Sulfate:Ldose: 1 (uM) Interaction | 21.01927402 | 15.05685414 | 1.395993733 | 0.162730282 |
| MeWo:Vincristine.Sulfate:Ldose: 1 (uM) Interaction | 22.7536374 | 15.13733352 | 1.503146995 | 0.132815306 |
| SKMEL2:Vincristine.Sulfate:Ldose: 1 (uM) Interaction | 47.17023345 | 15.31686613 | 3.079626932 | 0.00207512 |
| UACC0257:Vincristine.Sulfate:Ldose: 1 (uM) Interaction | 53.51436069 | 15.05685414 | 3.554152826 | 0.000379983 |
| MeWo:Vinorelbine.Tartrate:Ldose: 1 (uM) Interaction | 21.74724365 | 15.13733352 | 1.436662779 | 0.150827867 |
| SKMEL2:Vinorelbine.Tartrate:Ldose: 1 (uM) Interaction | 29.4062493 | 15.31686613 | 1.919860698 | 0.054888242 |
| UACC0257:Vinorelbine.Tartrate:Ldose: 1 (uM) Interaction | 27.69260433 | 15.05685414 | 1.839202537 | 0.065898691 |
| MeWo:Vismodegib:Ldose: 1 (uM) Interaction | 7.065072607 | 15.13733352 | 0.466731647 | 0.640696445 |
| SKMEL2:Vismodegib:Ldose: 1 (uM) Interaction | -13.11609191 | 15.31686613 | -0.856316939 | 0.391831697 |
| UACC0257:Vismodegib:Ldose: 1 (uM) Interaction | 9.452786155 | 15.05685414 | 0.627806185 | 0.530137344 |
| MeWo:Vorinostat:Ldose: 1 (uM) Interaction | -4.031515169 | 15.13733352 | -0.266329282 | 0.789988086 |
| SKMEL2:Vorinostat:Ldose: 1 (uM) Interaction | 6.186883697 | 15.31686613 | 0.403926211 | 0.686270855 |
| UACC0257:Vorinostat:Ldose: 1 (uM) Interaction | 56.96226227 | 15.05685414 | 3.783144988 | 0.000155264 |
| MeWo:Zoledronic.Acid:Ldose: 1 (uM) Interaction | -1.900241108 | 15.33308304 | -0.123930791 | 0.90137119 |

Supplemental Table 10: ANOVA analysis for site, dose, cell line, plate, and drug-dose-cell line interaction effects using an AIC derived set of control drugs as baseline. Signif. codes: 0 ‘***’ 0.001 ‘**’ 0.01 ‘*’ 0.05 ‘.’ 0.1 ‘ ’ 1
